# Whole-transcriptome analysis reveals a potential hsa_circ_0001955/hsa_circ_0000977-mediated miRNA-mRNA regulatory sub-network in colorectal cancer

**DOI:** 10.18632/aging.102945

**Published:** 2020-03-28

**Authors:** Bisha Ding, Minya Yao, Weimin Fan, Weiyang Lou

**Affiliations:** 1 Department of Breast Surgery, The First Affiliated Hospital, College of Medicine, Zhejiang University, Hangzhou, Zhejiang Province, China; 2 Program of Innovative Cancer Therapeutics, Division of Hepatobiliary and Pancreatic Surgery, Department of Surgery, First Affiliated Hospital, College of Medicine, Zhejiang University, Key Laboratory of Organ Transplantation, Hangzhou, Zhejiang Province, China; 3 Key Laboratory of Organ Transplantation, Hangzhou, Zhejiang Province, China; 4 Key Laboratory of Combined Multi-organ Transplantation, Ministry of Public Health, Hangzhou, Zhejiang Province, China

**Keywords:** colorectal cancer (CRC), circRNA, iRNA, competing endogenous RNA (ceRNA), hsa_circ_0001955/hsa_circ_0000977

## Abstract

Background: Circular RNAs (circRNAs), a novel class of non-coding RNAs, have been found to act as microRNA (miRNA) sponges and thus play key roles in biological processes and pathogenesis. However, studies regarding circRNAs in colorectal cancer (CRC) remain inadequate.

Results: By differential expression analysis, 10 candidate circRNAs (6 upregulated and 4 downregulated circRNAs) were chosen. 9 of 10 circRNAs were available on CSCD and their structure showed the binding potential of miRNA. Intersection analysis revealed that miR-145-5p, miR-3127-5p, miR-761, miR-4766-3p, miR-135a-5p, miR-135b-5p, miR-374a-3p and miR-330-3p were 8 miRNAs with the most potential in binding circRNAs. Further expression validation and correlation analysis demonstrated hsa_circ_0001955/miR-145-5p and hsa_circ_0000977/miR-135b-5p axes as key pathways in CRC. Subsequently, target gene prediction, differential expression analysis, intersection analysis and correlation analysis showed that CDK6, MMP12 and RAB3IP were the three potential downstream targets of hsa_circ_0001955/miR-145-5p axis and FOXO1, MBNL1, MEF2C, RECK, PPM1E, TTLL7 and PCP4L1 were the seven potential downstream targets of hsa_circ_0000977/miR-135b-5p axis in CRC. Finally, we also confirmed that expression of hsa_circ_0001955 or hsa_circ_0000977 was significantly positively correlated with their individual targets in CRC.

Conclusions: In the present work, we constructed a potential hsa_circ_0001955/hsa_circ_0000977-mediated circRNA-miRNA-mRNA regulatory network in CRC by a series of *in silico* analysis and experimental validation.

Methods: Whole-transcriptome microarrays from CRC and matched normal samples were obtained from GEO. The structure of circRNA was identified by CSCD. starBase and miRNet were successively used to predict miRNA of circRNA and target gene of miRNA. Expression correlation between RNA-RNA interactions was assessed using GEO and TCGA data. Finally, a potential circRNA-miRNA-mRNA network was established based on competing endogenous RNA (ceRNA) hypothesis.

## INTRODUCTION

Colorectal cancer (CRC) ranks as the third most common malignancy and is the third leading cause of cancer-associated deaths in the United States [1]. A lot of factors, including diet, polyps and chronic inflammation, have been documented to link to CRC pathogenesis, consisting of multiple processes. However, the alterations and roles of genomics and epigenetics in CRC initiation and progression are still rarely known. Moreover, despite received standard therapies, like surgery combined with chemotherapy and radiotherapy, many CRC patients will eventually recur or metastasize [2]. Therefore, it is urgent to further investigate molecular mechanism of CRC carcinogenesis, and seek and develop effective therapeutic targets for patients with CRC.

Circular RNAs (circRNAs) are a class of novel endogenous non-coding RNAs (ncRNAs) with a covalently closed continuous loop, and they are generated by the selective cleavage of premature RNAs [3]. The loop structure produced by linking 5’ and 3’ end renders circRNAs resistant to exonucleases and more stable than their corresponding linear transcripts [4]. In 2011, Salmena L et al*.* proposed a competing endogenous RNA (ceRNA) hypothesis, in which ncRNAs could positively regulate mRNA expression through competitively binding to shared microRNAs (miRNAs) [5]. Recent studies have well-demonstrated that circRNAs increase oncogene or tumor suppressor expression by ceRNA mechanism, and thereby participate in cancer development. For example, Xue D et al*.* found that circ-AKT3 inhibited clear cell renal cell carcinoma metastasis by modulating miR-296-3p/E-cadherin axis [6]; Han D et al*.* suggested that circMTO1 acted as the sponge of miR-9 to suppress progression of hepatocellular carcinoma [7]; Wang C et al*.* showed that circFOXO3 induced cell apoptosis in urothelial carcinoma by interaction with miR-191-5p [8]. circRNAs have also been confirmed to play critical roles in various biological behaviors of CRC, including proliferation, migration and invasion [9, 10]. Moreover, circRNAs can serve as diagnostic or prognostic biomarkers for CRC patients [11, 12].

Establishment of circRNA-miRNA-mRNA regulatory networks may provide key clues for us to explore the detailed molecular mechanism of human disorders including malignancies. For example, Liu K et al*.* uncovered the possible mechanism of Hsp90 inhibitor-induced cell death of CRC by identification of a circRNA-miRNA-mRNA regulatory network [13]. Yuan W et al*.* identified circRNAs as ceRNAs for miRNA-mRNA in CRC [14]; Song W et al*.* also constructed a circRNA-associated ceRNA network in CRC [15]. The two studies constructed circRNA-miRNA-mRNA network using RNA data from different CRC patients.

In this study, we obtained and analyzed the circRNA-miRNA-mRNA ceRNA regulatory network using 10 CRC patients’ circRNA, miRNA and mRNA expression profile data. Furthermore, TCGA data were employed to validate the analytic results. A hsa_circ_0001955/hsa_circ_0000977-mediated miRNA-mRNA regulatory sub-network in colorectal cancer was successfully established. Finally, experimental validation was given. Those findings from the present work may provide novel insight into the molecular mechanism of CRC carcinogenesis.

## RESULTS

### Selection of 9 potential circRNAs in CRC

**Figure 1:**
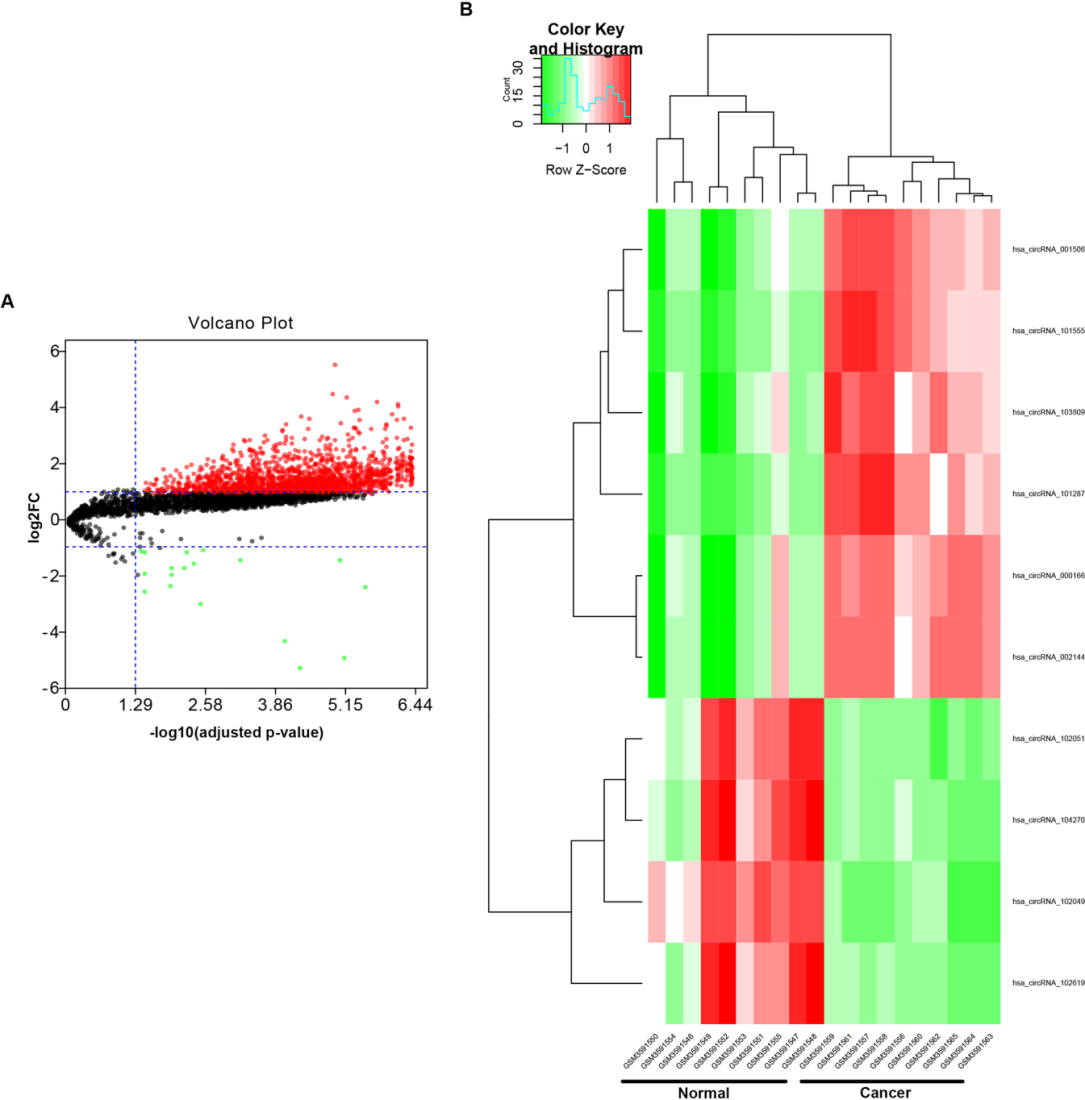
**Identification of potential circRNAs in colorectal cancer.** (**A**) The volcano plot of differentially expressed circRNAs (DECs) in colorectal cancer from GSE126094 dataset. The red dots and green dots represent upregulated DECs and downregulated DECs with significance (adjusted P-value < 0.05 and |log_2_FC| > 1), respectively. The black dots are those DECs without significance. (**B**) The heatmap of 10 potential DECs (|log_2_FC| > 4).

**Figure 2:**
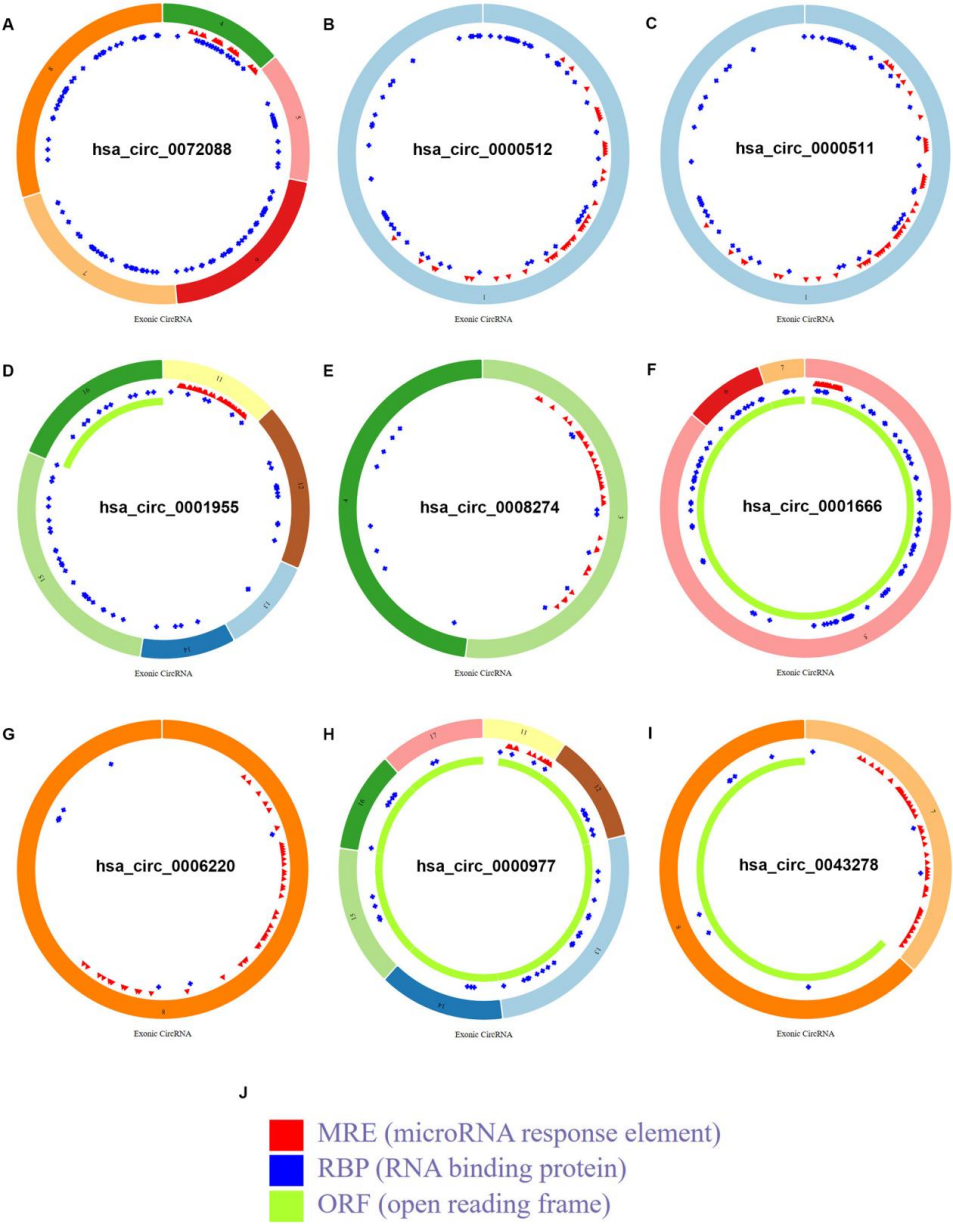
**Structural patterns of the 9 circRNAs in CSCD database.** (**A**) The structural pattern of hsa_circ_0072088. (**B**) The structural pattern of hsa_circ_0000512. (**C**) The structural pattern of hsa_circ_0000511. (**D**) The structural pattern of hsa_circ_0001955. (**E**) The structural pattern of hsa_circ_0008274. (**F**) The structural pattern of hsa_circ_0001666. (**G**) The structural pattern of hsa_circ_0006220. (**H**) The structural pattern of hsa_circ_0000977. (**I**) The structural pattern of hsa_circ_0043278. (**J**) The representation of MRE, RBP and ORF.

To find the potential circRNAs in CRC, circRNA dataset of 10 CRC patients (GSE126094) from GEO database was selected. GEO2R tool provided by GEO database was used to perform differential expression analysis for GSE126094. As listed in Figure 1A and Supplementary Table 1, a total of 1850 significant DECs, containing 1831 upregulated DECs and 19 downregulated DECs, were screened out. Next, we further selected some more potential DECs from the 1850 significant DECs based on the selection criterion of |log_2_FC| > 4, and 10 circRNAs, including 6 upregulated and 4 downregulated circRNAs, were finally identified (Figure 1B and Table 1). The circBase names and parental genes of the 10 circRNAs were presented in Table 2. Subsequently, the structure of 9 circRNAs, hsa_circ_0072088 (Figure 2A), hsa_circ_0000512 (Figure 2B), hsa_circ_0000511 (Figure 2C), hsa_circ_0001955 (Figure 2D), hsa_circ_0008274 (Figure 2E), hsa_circ_0001666 (Figure 2F), hsa_circ_0006220 (Figure 2G), hsa_circ_0000977 (Figure 2H) and hsa_circ_0043278 (Figure 2I) (hsa_circ_0000981 not available) was described in Figure 2 using the data from CSCD, indicating that all the 9 circRNAs had microRNA response elements (MREs). Taken together, the 9 circRNAs may be the key circRNAs in CRC by acting as miRNA sponges.

**Table 1:** The potential differentially expressed circRNAs (DECs) between colorectal cancer tissues and adjacent normal tissues.

**circBase ID**	**adj.P.Val**	**t**	**B**	**Log_2_FC**
hsa_circ_0072088	1.16E-05	6.617	5.369	5.586
hsa_circ_0000512	1.32E-05	6.511	5.135	4.524
hsa_circ_0000511	7.39E-06	6.923	6.035	4.438
hsa_circ_0000981	1.96E-06	7.963	8.201	4.272
hsa_circ_0001955	8.01E-07	8.851	9.929	4.180
hsa_circ_0008274	8.04E-07	8.826	9.883	4.113
hsa_circ_0001666	9.99E-05	-5.244	2.246	-4.272
hsa_circ_0006220	7.85E-06	-6.882	5.945	-4.872
hsa_circ_0000977	5.21E-05	-5.628	3.137	-5.209
hsa_circ_0043278	3.59E-07	-11.621	14.651	-6.443

**Table 2:** The parental genes of 10 potential circRNAs.

**circRNA ID**	**circBase ID**	**Parental gene**
hsa_circRNA_103809	hsa_circ_0072088	ZFR
hsa_circRNA_000166	hsa_circ_0000512	RPPH1
hsa_circRNA_002144	hsa_circ_0000511	RPPH1
hsa_circRNA_001506	hsa_circ_0000981	LAPTM4A
hsa_circRNA_101555	hsa_circ_0001955	CSNK1G1
hsa_circRNA_101287	hsa_circ_0008274	UGGT2
hsa_circRNA_104270	hsa_circ_0001666	FAM120B
hsa_circRNA_102051	hsa_circ_0006220	TADA2A
hsa_circRNA_102619	hsa_circ_0000977	NOL10
hsa_circRNA_102049	hsa_circ_0043278	TADA2A

### Prediction and analysis of binding miRNAs of potential circRNAs in CRC

**Figure 3:**
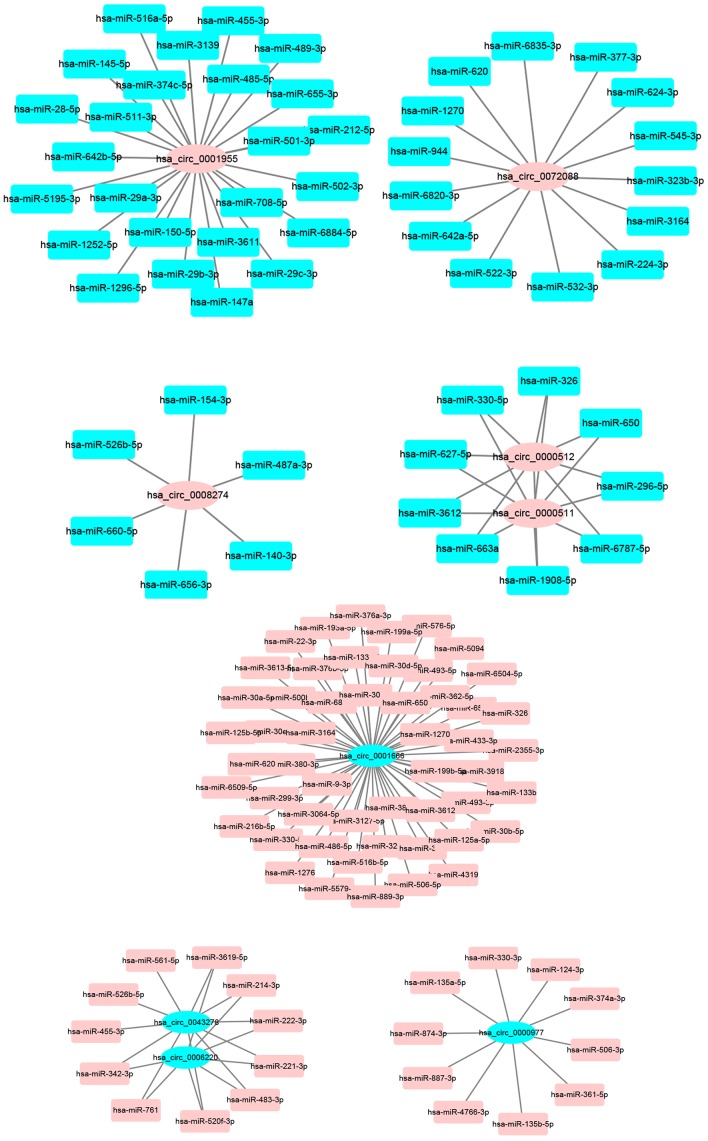
**The binding miRNAs of 9 circRNAs predicted by starBase.**

**Figure 4:**
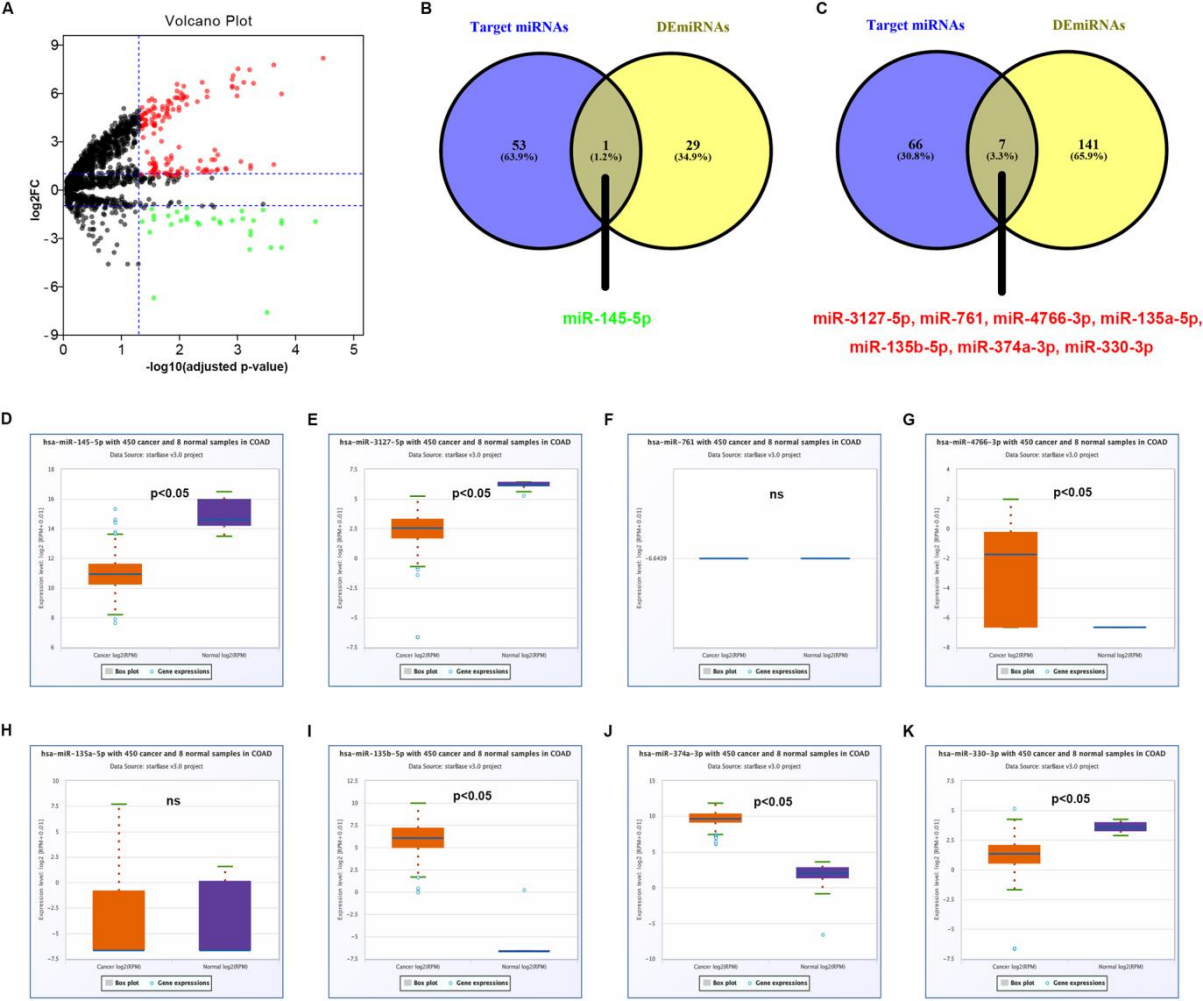
**Identification of potential miRNAs that bind to circRNAs in colorectal cancer.** (**A**) The volcano plot of differentially expressed miRNAs (DEmiRNAs) in colorectal cancer from GSE126093 dataset. The red dots and green dots represent upregulated DEmiRNAs and downregulated DEmiRNAs with significance (adjusted P-value < 0.05 and |log_2_FC| > 1), respectively. The black dots are those DEmiRNAs without significance. (**B**) The intersection analysis of target miRNAs of upregulated circRNAs and downregulated DEmiRNA. (**C**) The intersection analysis of target miRNAs of downregulated circRNAs and upregulated DEmiRNA. (**D**) The expression level of hsa-miR-145-5p in COAD. (**E**) The expression level of hsa-miR-3127-5p in COAD. (**F**) The expression level of hsa-miR-761 in COAD. (**G**) The expression level of hsa-miR-4766-3p in COAD. (**H**) The expression level of hsa-miR-135a-5p in COAD. (**I**) The expression level of hsa-miR-135b-5p in COAD. (**J**) The expression level of hsa-miR-374a-3p in COAD. (**K**) The expression level of hsa-miR-330-3p in COAD. “p<0.05” represents significant difference and “ns” represents no significance.

**Figure 5:**
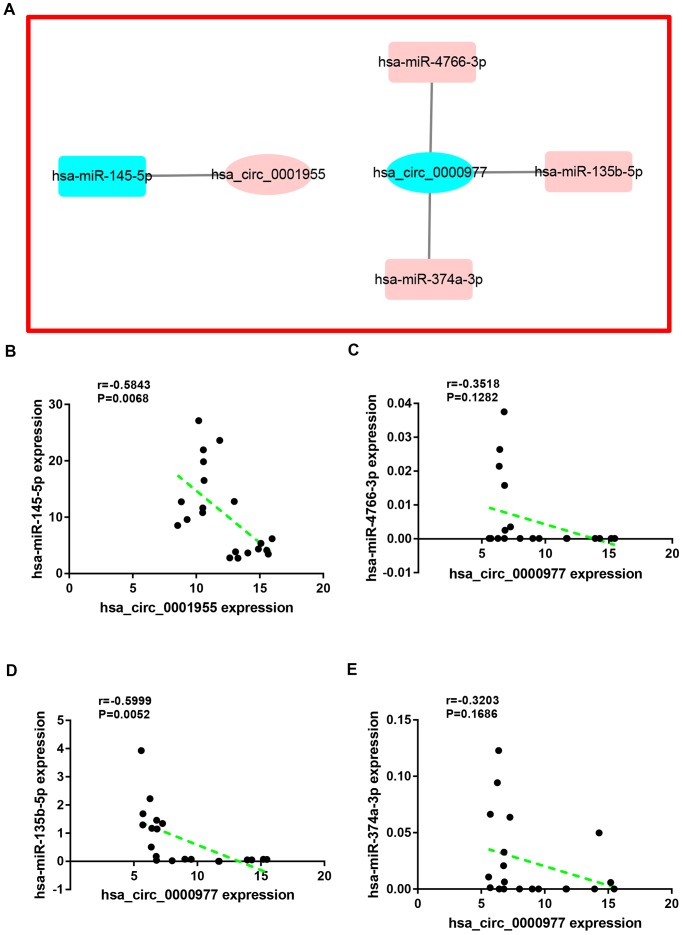
**Correlation analysis for potential circRNA-miRNA pairs in colorectal cancer.** (**A**) The circRNA-miRNA sub-network of interest. (**B**) The correlation of hsa_circ_0001955 with hsa-miR-145-5p in colorectal cancer. (**C**) The correlation of hsa_circ_0000977 with hsa-miR-4766-3p in colorectal cancer. (**D**) The correlation of hsa_circ_0000977 with hsa-miR-135b-5p in colorectal cancer. (**E**) The correlation of hsa_circ_0000977 with hsa-miR-374a-3p in colorectal cancer. P-value < 0.05 was considered as statistically significant.

Next, miRNAs that might bind to the 9 circRNAs were predicted through starBase database. As listed in Supplementary Table 2, 14, 9, 9, 25, 6, 52, 8, 10 and 11 miRNAs were found to potentially bind to hsa_circ_0072088, hsa_circ_0000512, hsa_circ_0000511, hsa_circ_0001955, hsa_circ_0008274, hsa_circ_0001666, hsa_circ_0006220, hsa_circ_0000977 and hsa_circ_0043278, respectively. For better visualization, the circRNA-miRNA connecting network was established by Cytoscape software as presented in Figure 3. According to ceRNA hypothesis, circRNA expression should be theoretically inversely linked to miRNA expression. Thus, we firstly screened out DEmiRNAs between CRC tissues and adjacent normal tissues using miRNA dataset GSE126093 from 10 CRC patients. Identically, GEO2R tool was employed to perform differential expression analysis for GSE126093, and 178 significant DEmiRNAs, containing 148 upregulated DEmiRNAs and 30 downregulated DEmiRNAs, were identified. The 178 significant DEmiRNAs were listed in Supplementary Table 3 and vividly presented in Figure 4A. Subsequently, by intersection of predicted targeting miRNAs of circRNAs and significant DEmiRNAs, we discovered that miR-145-5p were commonly appeared in target miRNAs of upregulated DECs set and downregulated DEmiRNAs set (Figure 4B), and 7 miRNAs (miR-3127-5p, miR-761, miR-4766-3p, miR-135a-5p, miR-135b-5p, miR-374a-3p and miR-330-3p) were collectively appeared in target miRNAs of downregulated DECs set and upregulated DEmiRNAs set (Figure 4C). Followingly, expression levels of the 8 miRNAs were also validated using TCGA data as shown in Figure 4D–4K. The results demonstrated that only miR-145-5p (Figure 4D), miR-4766-3p (Figure 4G), miR-135b-5p (Figure 4I) and miR-374a-3p (Figure 4J) expression were in accordance with the previous analytic findings. Thus, miR-145-5p, miR-4766-3p, miR-135b-5p and miR-374a-3p may be the most potential downstream miRNAs of the key circRNAs in CRC. By search of the predicted circRNA-miRNA binding pairs in Figure 3 or Supplementary Table 2, miR-145-5p potentially bound to upregulated hsa_circ_0001955, and miR-4766-3p, miR-135b-5p and miR-374a-3p were three binding miRNAs of one common downregulated circRNA, hsa_circ_0000977 (Figure 5A). The expression correlation of the four circRNA-miRNA pairs was also determined using data from GSE126094 and GSE126093 (Figure 5B–5E). All the 4 miRNAs were negatively correlated with their corresponding paired circRNA, which fitted the ceRNA hypothesis. However, among the four pairs, only hsa_circ_0001955/miR-145-5p and hsa_circ_0000977/miR-135b-5p possessed statistically significant. Altogether, hsa_circ_0001955/miR-145-5p and hsa_circ_0000977/miR-135b-5p might be two important axes in carcinogenesis of CRC.

### Identification of downstream target genes of miRNAs in CRC

**Figure 6:**
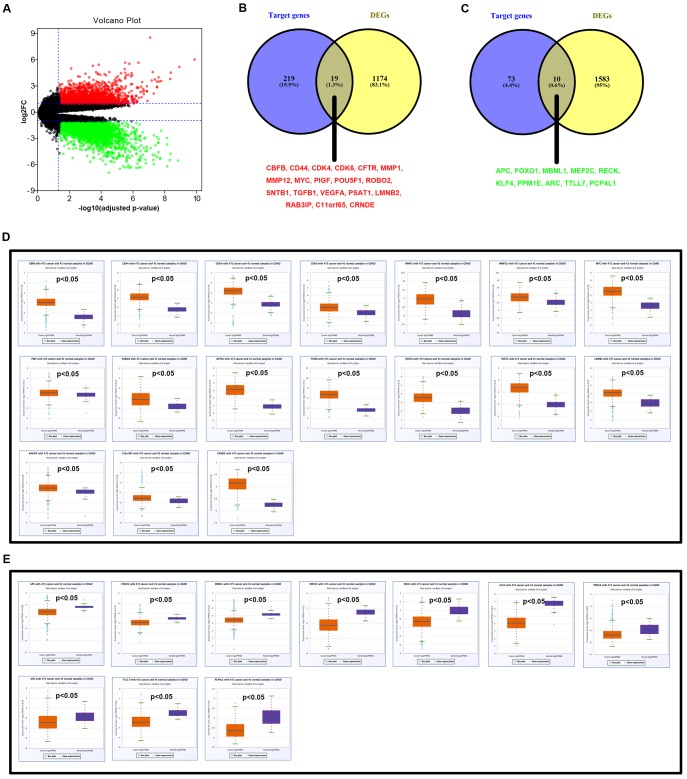
**Identification of potential targets of hsa-miR-145-5p and hsa-miR-135b-5p in colorectal cancer.** (**A**) The volcano plot of differentially expressed genes (DEGs) in colorectal cancer from GSE126092 dataset. The red dots and green dots represent upregulated DEGs and downregulated DEGs with significance (adjust P-value < 0.05 and |log_2_FC| > 1), respectively. The black dots are those DEGs without significance. (**B**) The intersection analysis of target genes of downregulated hsa-miR-145-5p and upregulated DEGs. (**C**) The intersection analysis of target genes of upregulated hsa-miR-135b-5p and downregulated DEGs. (**D**) Validation of expression of 17 potential upregulated DEGs using starBase. (**E**) Validation of expression of 10 potential downregulated DEGs using starBase. “p<0.05” represents significant difference.

It has been widely acknowledged that miRNAs exert their biological functions by negatively regulated expression of downstream target genes. Therefore, the next step was to ascertain the downstream target genes of hsa_circ_0001955/miR-145-5p and hsa_circ_0000977/miR-135b-5p axes. Target genes of miR-145-5p and miR-135b-5p were predicted through miRNet database, and 238 and 83 target genes were forecasted to potentially bind to miR-145-5p and miR-135b-5p, respectively (Supplementary Table 4). DEGs between CRC and normal controls were obtained by GEO2R analysis using GSE126092 from the same 10 CRC patients with GSE126093 and GSE126094. As presented in Figure 6A and Supplementary Table 5, a total of 2787 DEGs, including 1194 upregulated DEGs and 1593 downregulated DEGs, were finally found. According to action mechanism of miRNA, miRNA expression should be negatively associated with expression of target gene. Thus, we performed intersection analysis for target genes set and DEGs set. As shown in Figure 6B, 19 genes (CBFB, CD44, CDK4, CDK6, CFTR, MMP1, MMP12, MYC, PIGF, POU5F1, ROBO2, SNTB1, TGFBI, VEGFA, PSAT1, LMNB2, RAB3IP, C11orf65 and CRNDE) were commonly appeared in miR-145-5p’s target genes set and upregulated DEGs set. And 10 genes (APC, FOXO1, MBNL1, MEF2C, RECK, KLF4, PPM1E, ARC, TTLL7 and PCP4L1) were commonly appeared in miR-135b-5p’s target genes set and downregulated DEGs set (Figure 6C). Next, expression levels of the 19 upregulated genes and 10 downregulated genes were further validated using TCGA data by starBase database. The results demonstrated that 17 of 19 upregulated genes (except CFTR and POU5F1) and 10 of 10 downregulated genes fitted the previous analytic findings as shown in Figure 6D and 6E, respectively. The 27 genes were considered as the downstream target genes of miR-145-5p and miR-135b-5p in CRC and selected for subsequent analysis.

### Establishment of a potential circRNA-miRNA-mRNA regulatory sub-network in CRC

**Figure 7:**
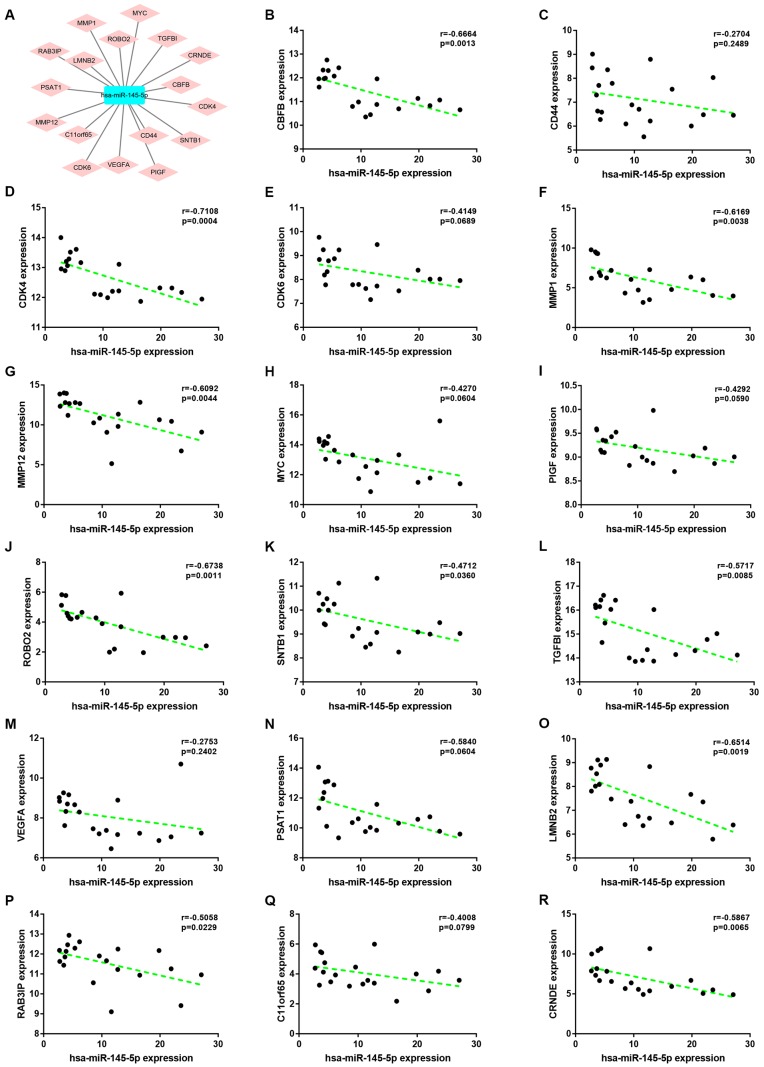
**Correlation analysis for potential hsa-miR-145-5p-target gene pairs in colorectal cancer.** (**A**) The visual hsa-miR-145-5p-target gene network. (**B**) The expression correlation of hsa-miR-145-5p with CBFB in colorectal cancer. (**C**) The expression correlation of hsa-miR-145-5p with CD44 in colorectal cancer. (**D**) The expression correlation of hsa-miR-145-5p with CDK4 in colorectal cancer. (**E**) The expression correlation of hsa-miR-145-5p with CDK6 in colorectal cancer. (**F**) The expression correlation of hsa-miR-145-5p with MMP1 in colorectal cancer. (**G**) The expression correlation of hsa-miR-145-5p with MMP12 in colorectal cancer. (**H**) The expression correlation of hsa-miR-145-5p with MYC in colorectal cancer. (**I**) The expression correlation of hsa-miR-145-5p with PIGF in colorectal cancer. (**J**) The expression correlation of hsa-miR-145-5p with ROBO2 in colorectal cancer. (**K**) The expression correlation of hsa-miR-145-5p with SNTB1 in colorectal cancer. (**L**) The expression correlation of hsa-miR-145-5p with TGFBI in colorectal cancer. (**M**) The expression correlation of hsa-miR-145-5p with VEGFA in colorectal cancer. (**N**) The expression correlation of hsa-miR-145-5p with PSAT1 in colorectal cancer. (**O**) The expression correlation of hsa-miR-145-5p with LMNB2 in colorectal cancer. (**P**) The expression correlation of hsa-miR-145-5p with RAB3IP in colorectal cancer. (**Q**) The expression correlation of hsa-miR-145-5p with C11orf65 in colorectal cancer. (**R**) The expression correlation of hsa-miR-145-5p with CRNDE in colorectal cancer. P-value < 0.05 was considered as statistically significant.

**Figure 8:**
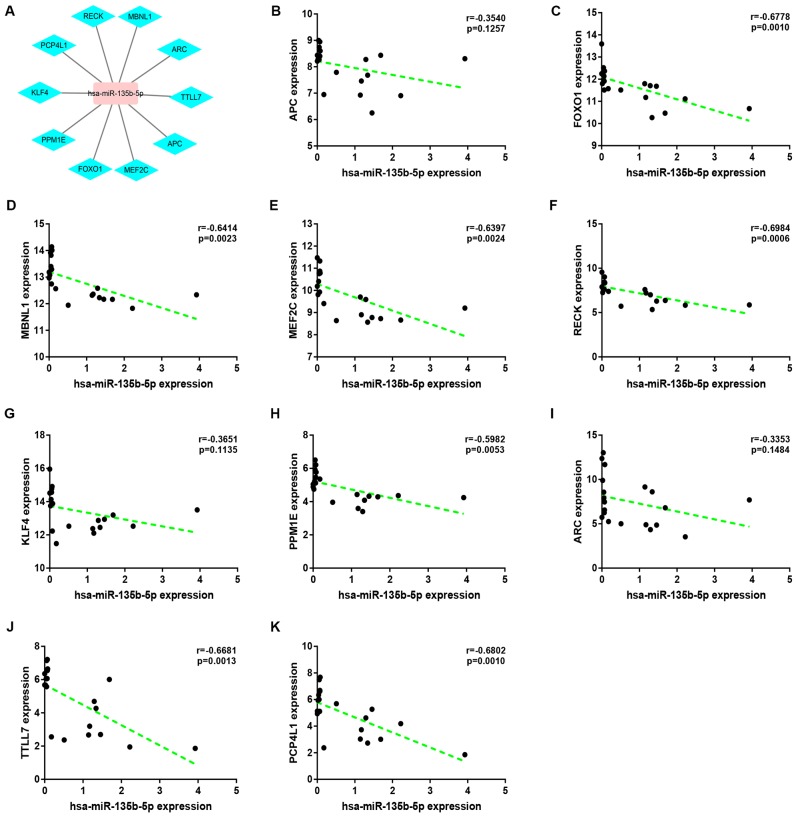
**Correlation analysis for potential hsa-miR-135b-5p-target gene pairs in colorectal cancer.** (**A**) The visual hsa-miR-145-5p-target gene network. (**B**) The expression correlation of hsa-miR-135b-5p with APC in colorectal cancer. (**C**) The expression correlation of hsa-miR-135b-5p with FOXO1 in colorectal cancer. (**D**) The expression correlation of hsa-miR-135b-5p with MBNL1 in colorectal cancer. (**E**) The expression correlation of hsa-miR-135b-5p with MEF2C in colorectal cancer. (**F**) The expression correlation of hsa-miR-135b-5p with RECK in colorectal cancer. (**G**) The expression correlation of hsa-miR-135b-5p with KLF4 in colorectal cancer. (**H**) The expression correlation of hsa-miR-135b-5p with PPM1E in colorectal cancer. (**I**) The expression correlation of hsa-miR-135b-5p with ARC in colorectal cancer. (**J**) The expression correlation of hsa-miR-135b-5p with TTLL7 in colorectal cancer. (**K**) The expression correlation of hsa-miR-135b-5p with PCP4L1 in colorectal cancer. P-value < 0.05 was considered as statistically significant.

**Figure 9:**
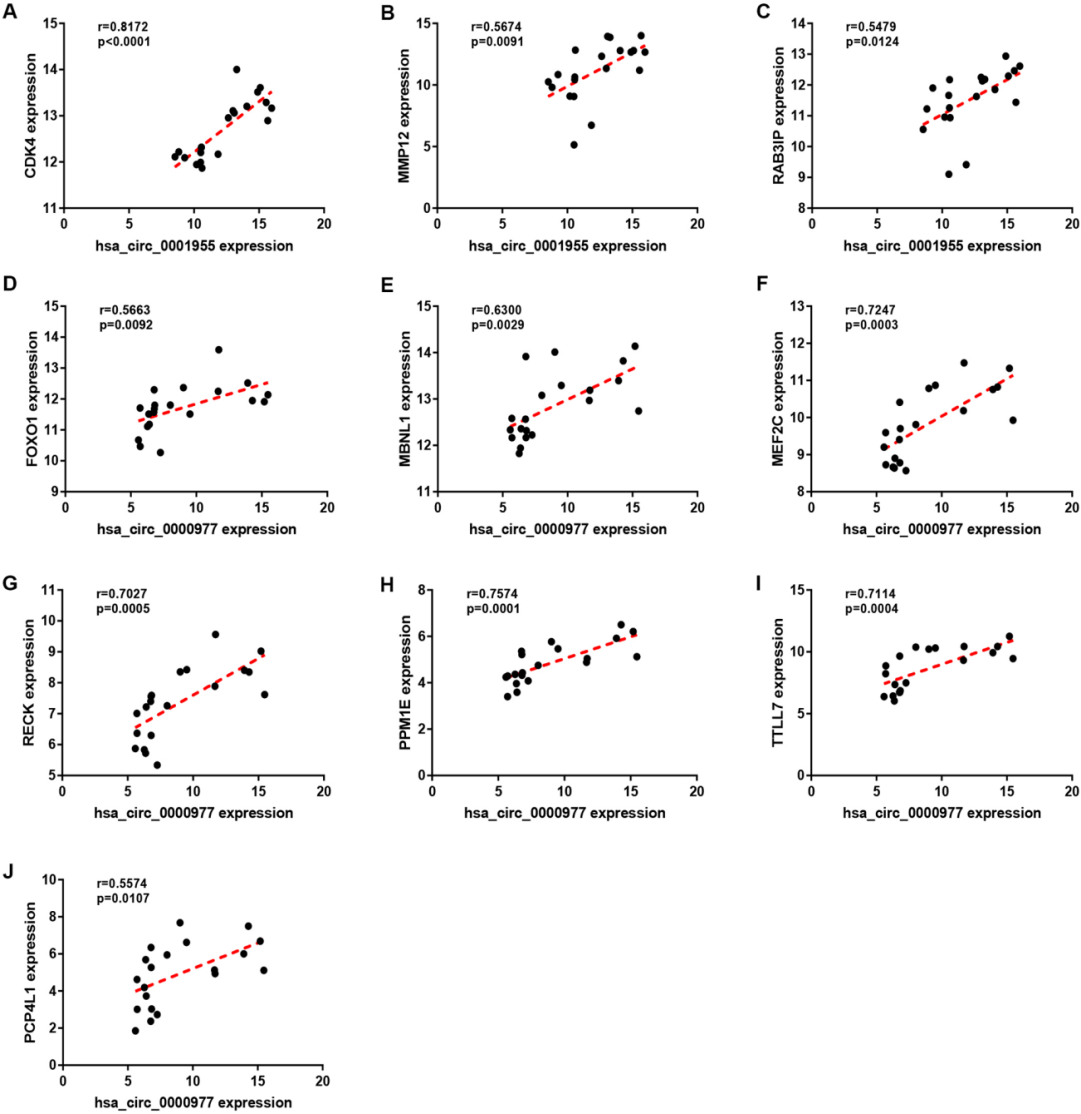
**Correlation analysis for potential circRNA-target gene pairs in colorectal cancer.** (**A**) The expression correlation of hsa_circ_0001955 with CDK4 in colorectal cancer. (**B**) The expression correlation of hsa_circ_0001955 with MMP12 in colorectal cancer. (**C**) The expression correlation of hsa_circ_0001955 with RAB3IP in colorectal cancer. (**D**) The expression correlation of hsa_circ_0000977 with FOXO1 in colorectal cancer. (**E**) The expression correlation of hsa_circ_0000977 with MBNL1 in colorectal cancer. (**F**) The expression correlation of hsa_circ_0000977 with MEF2C in colorectal cancer. (**G**) The expression correlation of hsa_circ_0000977 with RECK in colorectal cancer. (**H**) The expression correlation of hsa_circ_0000977 with PPM1E in colorectal cancer. (**I**) The expression correlation of hsa_circ_0000977 with TTLL7 in colorectal cancer. (**J**) The expression correlation of hsa_circ_0000977 with PCP4L1 in colorectal cancer. P-value < 0.05 was considered as statistically significant.

**Figure 10:**
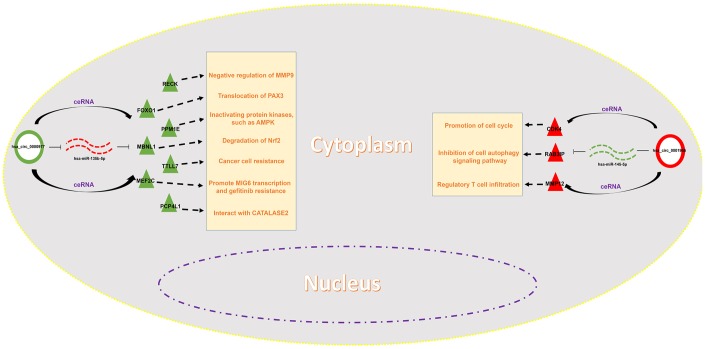
**Construction of a potential circRNA-mediated ceRNA regulatory network in colorectal cancer.**

**Figure 11:**
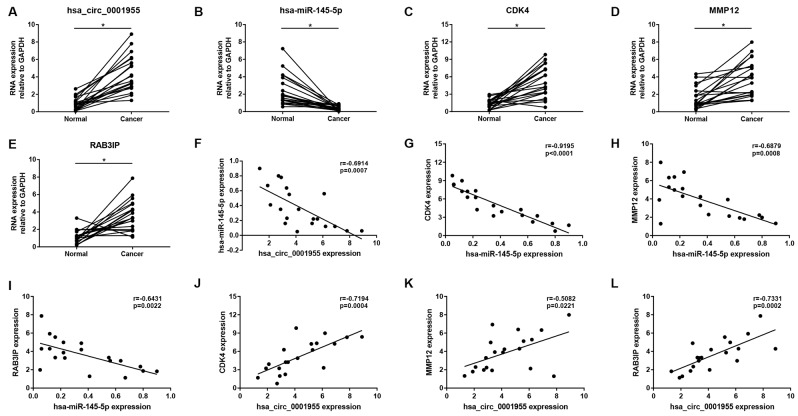
**Validation of hsa_circ_0001955-mediated miRNA-mRNA sub-network in CRC using clinical tissue samples.** Expression of hsa_circ_0001955 (**A**), hsa-miR-145-5p (**B**), CDK4 (**C**), MMP12 (**D**) and RAB3IP (**E**) in CRC cancer samples compared with normal samples. Expression correlation between hsa_circ_0001955/hsa-miR-145-5p (**F**), hsa-miR-145-5p/CDK4 (**G**), hsa-miR-145-5p/MMP12 (**H**), hsa-miR-145-5p/RAB3IP (**I**), hsa_circ_0001955/CDK4 (**J**), hsa_circ_0001955/MMP12 (**K**) and hsa_circ_0001955/RAB3IP (**L**) pairs in CRC. *p<0.05 represents significant difference.

**Figure 12:**
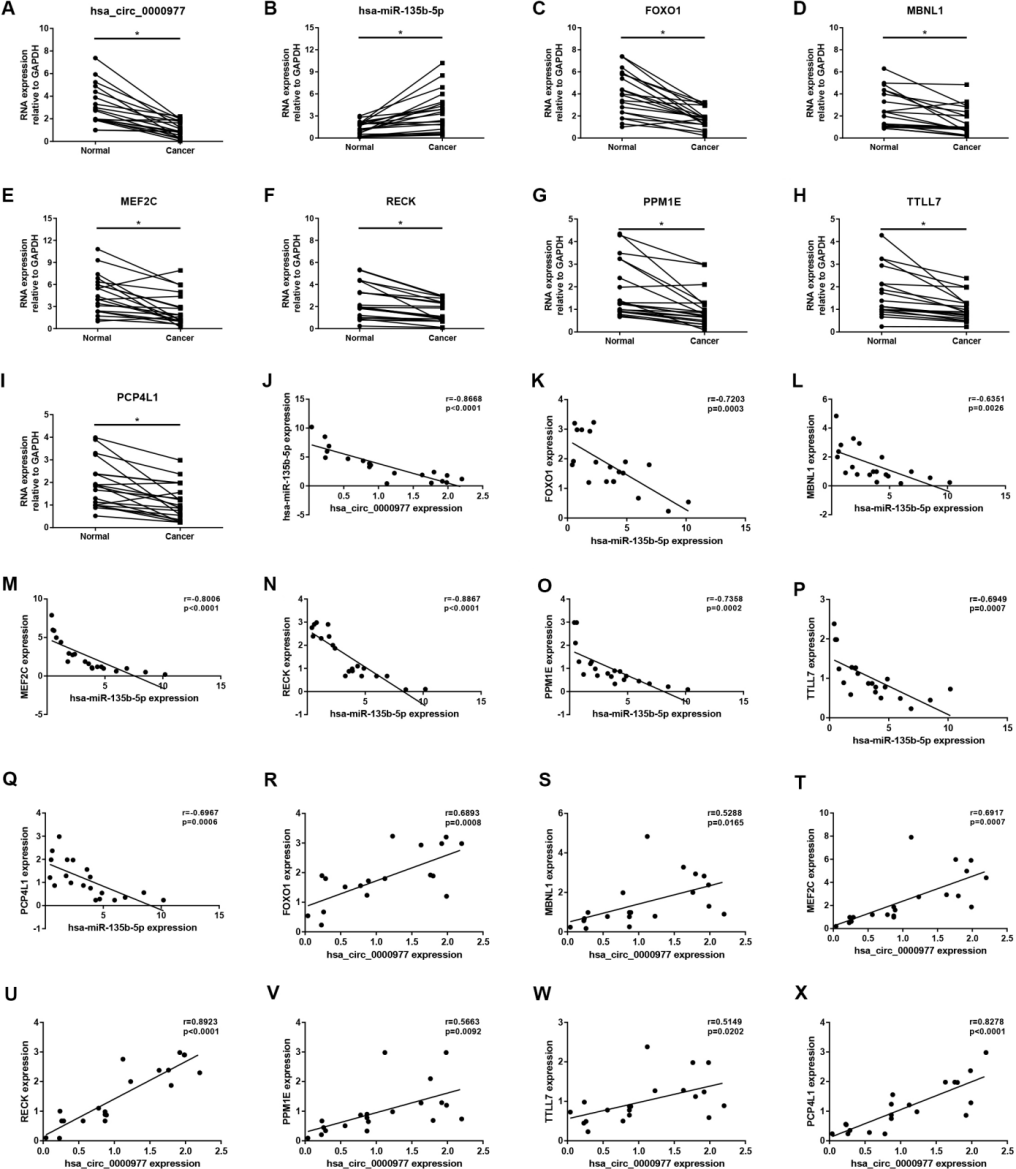
**Validation of hsa_circ_0000977-mediated miRNA-mRNA sub-network in CRC using clinical tissue samples.** Expression of hsa_circ_0000977 (**A**), hsa-miR-135b-5p (**B**), FOXO1 (**C**), MBNL1 (**D**), MEF2C (**E**), RECK (**F**), PPM1E (**G**), TTLL7 (**H**) and PCP4L1 (**I**) in CRC cancer samples compared with normal samples. Expression correlation between hsa_circ_0000977/ hsa-miR-135b-5p (**J**), hsa-miR-135b-5p/FOXO1 (**K**), hsa-miR-135b-5p/MBNL1 (**L**), hsa-miR-135b-5p/MEF2C (**M**), hsa-miR-135b-5p/RECK (**N**), hsa-miR-135b-5p/PPM1E (**O**), hsa-miR-135b-5p/TTLL7 (**P**), hsa-miR-135b-5p/PCP4L1 (**Q**), hsa_circ_0000977/FOXO1 (**R**), hsa_circ_0000977/MBNL1 (**S**), hsa_circ_0000977/MEF2C (**T**), hsa_circ_0000977/RECK (**U**), hsa_circ_0000977/PPM1E (**V**), hsa_circ_0000977/TTLL7 (**W**) and hsa_circ_0000977/PCP4L1 (**X**) pairs in CRC. *p<0.05 represents significant difference.

Expression correlation of between miR-145-5p or miR-135b-5p and their corresponding target genes in CRC was assessed using the data from GSE126093 and GSE126092. The miR-145-5p-target gene network, containing 17 miRNA-target gene pairs, was constructed in Figure 7A. As presented in Figure 7B–7R, miR-145-5p expression was inversely linked to all the 17 target gene expression whereas only miR-145-5p/CBFB, miR-145-5p/CDK4, miR-145-5p/MMP1, miR-145-5p/MMP12, miR-145-5p/ROBO2, miR-145-5p/SNTB1, miR-145-5p/TGFBI, miR-145-5p/LMNB2, miR-145-5p/RAB3IP and miR-145-5p/CRNDE pairs had statistically significant. The miR-135b-5p-target gene network, involving 10 miRNA-target gene pairs, was shown in Figure 8A. Next, the expression correlation of miR-135b-5p with 10 target genes was also detected (Figure 8B–8K). The results suggested that 7 of 10 pairs (miR-135b-5p/FOXO1, miR-135b-5p/MBNL1, miR-135b-5p/MEF2C, miR-135b-5p/RECK, miR-135b-5p/PPM1E, miR-135b-5p/TTLL7 and miR-135b-5p/PCP4L1) possessed statistically significant. We further determine the expression relationship between miR-145-5p or miR-135b-5p and their target genes using TCGA data and the results were listed in Tables 3, 4, respectively. By intersection of analytic findings from GEO and TCGA database, we found that three genes (CDK6, MMP12 and RAB3IP) and seven genes (FOXO1, MBNL1, MEF2C, RECK, PPM1E, TTLL7 and PCP4L1) were the potential downstream targets of miR-145-5p and miR-135b-5p in CRC, respectively. According to ceRNA mechanism, circRNA expression should be positively associated with target gene expression. Therefore, we finally evaluated the expression correlation of hsa_circ_0001955 with CDK6/MMP12/RAB3IP or hsa_circ_0000977 with FOXO1/MBNL1/MEF2C/RECK/PPM1E/TTLL7/PCP4L1 in CRC (Figure 9A–9J). Intriguingly, all the 10 genes were significantly positively correlated with their corresponding circRNAs. Taken all these findings together, a potential circRNA-miRNA-mRNA regulatory sub-network in CRC was successively established, containing two pathways hsa_circ_0001955-miR-145-5p-CDK4/MMP12/RAB3IP and hsa_circ_0000977-miR-135b-5p-FOXO1/MBNL1/MEF2C/RECK/PPM1E/TTLL7/PCP4 L1 (Figure 10). To further affirm the analytic results, we collected 20 CRC cancer samples and adjacent normal samples from the First Affiliated Hospital of Zhejiang University. The expression levels of all RNAs in the constructed network in CRC were determined by qRT-PCR and expression relationships among them were also assessed. As presented in Figure 11A–11E and Figure 12A–12I, hsa_circ_0001955, hsa-miR-135b-5p, CDK4, MMP12 and RAB3IP were significantly upregulated whereas hsa_circ_0000977, hsa-miR-145-5p, FOXO1, MBNL1, MEF2C, RECK, PPM1E, TTLL7 and PCP4L1 were markedly downregulated in CRC when compared with normal controls, which were in accordance with GEO and TCGA data. Moreover, Figure 11F–11L and Figure 12J–12X showed a similar pattern of results compared to previous *in silico* analytic results, partially supporting the accuracy of *in silico* analysis in this study.

**Table 3:** The correlation analysis of miR-145-5p and potential target gene in colorectal cancer using starBase.

**miRNA ID**	**gene ID**	**R**	**P-value**
hsa-miR-145-5p	CBFB	-0.024	6.14E-01
hsa-miR-145-5p	CD44	-0.044	3.53E-01
hsa-miR-145-5p	CDK4	-0.189	5.68E-05
hsa-miR-145-5p	CDK6	0.149	1.51E-03
hsa-miR-145-5p	MMP1	-0.058	2.18E-01
hsa-miR-145-5p	MMP12	-0.123	8.77E-03
hsa-miR-145-5p	MYC	-0.061	1.96E-01
hsa-miR-145-5p	PIGF	-0.166	4.14E-04
hsa-miR-145-5p	ROBO2	0.166	4.07E-04
hsa-miR-145-5p	SNTB1	0.031	5.12E-01
hsa-miR-145-5p	TGFBI	0.139	3.13E-03
hsa-miR-145-5p	VEGFA	-0.03	5.31E-01
hsa-miR-145-5p	PSAT1	-0.184	8.86E-05
hsa-miR-145-5p	LMNB2	-0.038	4.16E-01
hsa-miR-145-5p	RAB3IP	-0.123	9.23E-03
hsa-miR-145-5p	C11orf65	-0.043	3.63E-01
hsa-miR-145-5p	CRNDE	0.05	2.91E-01

**Table 4:** The correlation analysis of miR-135b-5p and potential target gene in colorectal cancer using starBase.

**miRNA ID**	**gene ID**	**R**	**P-value**
hsa-miR-135b-5p	APC	-0.185	8.20E-05
hsa-miR-135b-5p	FOXO1	-0.199	2.06E-05
hsa-miR-135b-5p	MBNL1	-0.309	2.06E-11
hsa-miR-135b-5p	MEF2C	-0.35	2.15E-14
hsa-miR-135b-5p	RECK	-0.326	1.24E-12
hsa-miR-135b-5p	KLF4	-0.126	7.59E-03
hsa-miR-135b-5p	PPM1E	-0.105	2.55E-02
hsa-miR-135b-5p	ARC	-0.024	6.13E-01
hsa-miR-135b-5p	TTLL7	-0.405	3.77E-19
hsa-miR-135b-5p	PCP4L1	-0.141	2.70E-03

## DISCUSSION

Increasing evidences have demonstrated that circRNAs serve as crucial players in initiation and progression of human cancers, including CRC. In spite of some studies have reported their constructed circRNA-miRNA-mRNA regulatory network in CRC [14, 15]. However, the current knowledge of circRNA-associated ceRNA network in CRC remain inadequate and need to be further investigated. In the present study, by a series of *in silico* analysis, we identified a potential circRNA-miRNA-mRNA ceRNA regulatory network in CRC pathogenesis.

After performing differential expression analysis, 10 circRNAs were selected for further research. Most of the 10 circRNAs have been found to be closely linked to cancer development and progression. For example, hsa_circRNA_103809 regulates CRC proliferation and migration via miR-532-3p/FOXO4 axis [16]; hsa_circRNA_101555 is involved in carcinogenesis of breast cancer [17] hsa_circRNA_102619 played a suppressive role in progression of pancreatic ductal adenocarcinoma [18]. By using circBase and CSCD databases, 9 of 10 circRNAs were finally identified.

Multiple studies have confirmed that circRNAs positively regulate expression of downstream genes by acting as miRNA sponges [19, 20]. Therefore, potential miRNAs of the 9 circRNAs were predicted. Followed by a series of analyses including miRNA expression analysis and correlation analysis between circRNA and miRNA in CRC, two pathways (hsa_circ_0001955/miR-145-5p and hsa_circ_0000977/miR-135b-5p) were regarded as the key axes in carcinogenesis of CRC. The functions of miR-145-5p and miR-135b-5p have been investigated, which were in accordance with ceRNA hypothesis. For example, miR-145-5p inhibited growth, migration and invasion of CRC [21, 22]. Silencing miR-135b-5p sensitized CRC cells to oxaliplatin-induced apoptosis by upregulating FOXO1 expression [23].

Next, we further explored the detailed downstream molecular mechanisms of hsa_circ_0001955/miR-145-5p and hsa_circ_0000977/miR-135b-5p axes in CRC. As is known to all, miRNAs exert their functions by negatively modulating gene expression [24–27]. Firstly, target genes of miR-145-5p and miR-135b-5p were predicted by a comprehensive database, namely miRNet. Then, the DEGs between CRC tissues and normal tissues were screened. 29 targets were selected by intersection of target gene set and DEG set, after which expression of the 29 targets were validated using TCGA data by starBase. Subsequently, correlation analysis of target gene with miRNA was performed through both GEO and TCGA data. Finally, we found that three targets of miR-145-5p (CDK4, MMP12 and RAB3IP) and seven targets of miR-135b-5p (FOXO1, MBNL1, MEF2C, RECK, PPM1E, TTLL7 and PCP4L1) possessed the most potential. These targets have been well-documented to closely link to cancer progression, including CRC. For instance, miR-143-3p-suppressed CDK4 enhanced CRC growth [28].

According to ceRNA mechanism, there should be positive expression relationship between circRNA and target gene. Intriguingly, hsa_circ_0001955 expression was positively correlated with expression of CDK4, MMP12 and RAB3IP, and hsa_circ_0000977 was also positively associated with expression of FOXO1, MBNL1, MEF2C, RECK, PPM1E, TTLL7 and PCP4L1 in CRC.

Taken all these data together, we successfully established a circRNA-miRNA-mRNA triple ceRNA sub-network mediated by two circRNAs (hsa_circ_0001955 and hsa_circ_0000977) in CRC. Moreover, the expression levels of RNAs in the network and expression correlation were followingly experimentally validated using clinical CRC samples, partially supporting the analytic accuracy of *in silico* analysis. The current work provides novel insight into the detailed molecular mechanism of CRC carcinogenesis, and the research route may also be applied to explore the ceRNA molecular interaction mechanism of circRNA-miRNA-mRNA network in initiation and progression of other types of human cancer. However, more efforts should be given to elucidate the function of the established network in CRC with *in vitro* and *in vivo* experiments. Meanwhile, the diagnostic and prognostic values of each RNA in the established network should be evaluated using a large number of clinical CRC data in the future, which will help to develop promising diagnostic and prognostic biomarkers for patients with CRC.

## CONCLUSIONS

In summary, this study reveals a potential hsa_circ_0001955/hsa_circ_0000977-mediated circRNA-miRNA-mRNA ceRNA sub-network in CRC by whole-transcriptome analysis, including differential expression analysis, intersection analysis and correlation analysis. Despite need more experimental and clinical validations, targeting the molecules in this sub-network may represent a promising treatment for patients with CRC.

## MATERIALS AND METHODS

### Inclusion of datasets

In this study, we intended to construct a key circRNA-miRNA-mRNA regulatory network in CRC. Firstly, we selected the datasets for analysis using NCBI GEO database (http://www.ncbi.nlm.nih.gov/geo/). Selection criteria: only datasets that focused on human CRC tissue samples were included; datasets regarding CRC cell lines or animal CRC tissue samples were excluded; only datasets with sample count more than ten were included; only datasets containing circRNA expression, miRNA expression and mRNA expression were included. Finally, only dataset GSE126095 met all these criteria mentioned above. GSE126095 had three sub-datasets (GSE126094, GSE126093 and GSE126092) from 10 CRC tissues and 10 matched normal tissues. GSE126094, based on the platform of GPL19978 Agilent-069978 Arraystar Human CircRNA microarray V1, investigated circRNA expression profile; GSE126093, based on the platform of GPL18058 Exiqon miRCURY LNA microRNA array G7, investigated miRNA expression profile; and GSE126092, based on the platform of GPL21047 Agilent-074348 Human LncRNA v6 4X180K.

### Differential analysis for circRNA, miRNA and mRNA

Differential analysis for GSE126094, GSE126093 and GSE126092 was successively conducted to obtain differentially expressed circRNAs (DECs), differentially expressed miRNAs (DEmiRNAs) and differentially expressed genes (DEGs) between CRC tissues and matched normal tissues by using an online tool GEO2R (http://www.ncbi.nlm.nih.gov/geo/geo2r/) which is provided by NCBI GEO database [29]. Adjust P-vlaue (adj.P.Val) < 0.05 and |log_2_FC (Fold change)| > 1 were set as the thresholds for identifying DECs, DEmiRNAs and DEGs.

### circBase and CSCD (Cancer-Specific CircRNA Database) analysis

circBase, a database for circRNA-associated studies, provides scripts to acquire known and novel circRNAs in sequencing data [30]. circBase was employed to identify parental genes of potential circRNAs. CSCD (http://gb.whu.edu.cn/CSCD/), which is an online tool to investigate cancer-specific circRNAs, was utilized to obtain structure of potential circRNAs.

### miRNA prediction

miRNAs that might bind to circRNAs were predicted by starBase database (http://starbase.sysu.edu.cn/). starBase database is an open-source platform that provides the ncRNA interactions from CLIP-seq, degradome-seq and RNA-RNA interactome data [31, 32]. The circBase ID of circRNA was entered into starBase database, after which potential miRNAs of circRNA were directly presented on the webpage.

### Validation of miRNA expression

The miRNAs that commonly appeared in target miRNAs set and DEmiRNAs set were regarded as potential miRNAs of circRNAs in CRC. Expression of these miRNAs was further validated using TCGA data by starBase database [31, 32]. P < 0.05 was considered as statistically significant.

### Target gene prediction

miRNet (https://www.mirnet.ca/), an integrated platform linking miRNAs, targets and functions, was introduced to perform miRNA’s target gene prediction [33]. The miRNA-gene interactions were directly downloaded from the webpage.

### Validation of gene expression

The genes that commonly appeared in target genes set and DEGs set were considered as potential target genes of miRNAs in CRC. Expression of these target genes were further confirmed using TCGA data using starBase database [31, 32]. P < 0.05 was considered as statistically significant.

### Correlation analysis for RNA-RNA interactions

The expression correlation of hsa_circ_0001955 or hsa_circ_0000977 with miRNA in CRC was evaluated using the data from GSE126094 and GSE126093; the expression correlation of miR-145-5p or miR-135b-5p with target gene in CRC was assessed using the data from GSE126093 and GSE126092; the expression correlation of hsa_circ_0001955 or hsa_circ_0000977 with target gene in CRC was determined using the data from GSE126094 and GSE126092. Then, starbase database was also employed to validate the expression correlation of miR-145-5p or miR-135b-5p with target gene. |R| > 0.1 and P-value < 0.05 were set as the thresholds for identifying significant RNA-RNA interactions.

### Clinical tissues and qRT-PCR

Fresh frozen CRC cancer tissues and adjacent normal tissues were obtained from 20 CRC patients who received surgical treatment at the First Affiliated Hospital of Zhejiang University (Hangzhou, China), and were frozen and stored in liquid nitrogen. This study was approved by the Ethical Committee of First Affiliated Hospital of Zhejiang University. Each informed consent was acquired from every patient. The expression of potential circRNAs, miRNAs and genes in these clinical samples was detected by the method of qRT-PCR as previously described [26]. The Primers used in this study were designed and purchased from RiboBio Co. Ltd (Guangzhou, China).

### Statistical analysis

Most of statistical analyses have been calculated by the online databases or tools. Expression correlation for RNA-RNA interactions was perform by Person correlation coefficient using Graphpad Prism software (version 7). Paired Student’s *t*-test was used to evaluate expression differences of circRNA, miRNA or gene between normal group and cancer group. Results with P-value < 0.05 were considered as statistically significant.

### Ethics approval

This study was approved by the Ethical Committee of First Affiliated Hospital of Zhejiang University. Each informed consent was acquired from every patient.

## Supplementary Material

Supplementary Table 1

Supplementary Table 2

Supplementary Table 3

Supplementary Table 4

Supplementary Table 5
