# Supplementary material for: Whole-transcriptome analysis reveals a potential hsa_circ_0001955/hsa_circ_0000977-mediated miRNA-mRNA regulatory sub-network in colorectal cancer
Source: Aging (Albany NY). 2020 Mar 28;12(6):5259–79. doi: 10.18632/aging.102945 (PMC7138558; doi:10.18632/aging.102945)
Supplement: Supplementary Table 1 [file aging-12-102945-s004..doc]

**Supplementary Table 1. Identification of differentially expressed circRNAs (DECs) between colorectal cancer tissues and adjacent normal tissues.**

| ID | adj.P.Val | t | B | Log_2_FC |
| --- | --- | --- | --- | --- |
| hsa_circRNA_103809 | 1.16E-05 | 6.61722 | 5.36852 | 5.58599 |
| hsa_circRNA_000166 | 1.32E-05 | 6.5112 | 5.13503 | 4.52354 |
| hsa_circRNA_002144 | 7.39E-06 | 6.92344 | 6.03471 | 4.4377 |
| hsa_circRNA_001506 | 1.96E-06 | 7.96299 | 8.20052 | 4.27232 |
| hsa_circRNA_101555 | 8.01E-07 | 8.8505 | 9.92934 | 4.18008 |
| hsa_circRNA_101287 | 8.04E-07 | 8.82587 | 9.88285 | 4.11314 |
| hsa_circRNA_104700 | 4.32E-06 | 7.32598 | 6.89123 | 3.98254 |
| hsa_circRNA_100146 | 5.19E-06 | 7.16715 | 6.55594 | 3.92307 |
| hsa_circRNA_002178 | 1.06E-06 | 8.58146 | 9.41694 | 3.81443 |
| hsa_circRNA_104852 | 5.02E-05 | 5.64916 | 3.18686 | 3.74701 |
| hsa_circRNA_104640 | 6.42E-07 | 9.12117 | 10.43475 | 3.67115 |
| hsa_circRNA_104475 | 3.59E-05 | 5.85569 | 3.66105 | 3.66986 |
| hsa_circRNA_400071 | 4.26E-06 | 7.33699 | 6.91433 | 3.63367 |
| hsa_circRNA_000167 | 8.69E-07 | 8.71391 | 9.67045 | 3.62587 |
| hsa_circRNA_100533 | 9.08E-06 | 6.78439 | 5.73375 | 3.49046 |
| hsa_circRNA_103188 | 1.32E-06 | 8.35604 | 8.97981 | 3.475 |
| hsa_circRNA_101145 | 9.30E-05 | 5.28628 | 2.34424 | 3.46612 |
| hsa_circRNA_001059 | 1.33E-06 | 8.32327 | 8.91566 | 3.40702 |
| hsa_circRNA_100367 | 5.41E-06 | 7.13013 | 6.47728 | 3.33543 |
| hsa_circRNA_104993 | 5.21E-06 | 7.16262 | 6.54632 | 3.33384 |
| hsa_circRNA_001846 | 4.87E-07 | 9.56946 | 11.24985 | 3.32774 |
| hsa_circRNA_102061 | 3.61E-05 | 5.84948 | 3.64686 | 3.30552 |
| hsa_circRNA_104499 | 2.59E-06 | 7.71961 | 7.70703 | 3.30358 |
| hsa_circRNA_104760 | 4.75E-06 | 7.24966 | 6.73055 | 3.30079 |
| hsa_circRNA_101707 | 1.42E-06 | 8.28721 | 8.84492 | 3.28444 |
| hsa_circRNA_101744 | 1.11E-04 | 5.18679 | 2.11145 | 3.26334 |
| hsa_circRNA_103348 | 1.08E-04 | 5.19918 | 2.14047 | 3.22954 |
| hsa_circRNA_101777 | 4.35E-06 | 7.3075 | 6.85239 | 3.19799 |
| hsa_circRNA_101164 | 4.29E-06 | 7.33085 | 6.90145 | 3.15301 |
| hsa_circRNA_102771 | 6.95E-06 | 6.96187 | 6.11745 | 3.1068 |
| hsa_circRNA_104922 | 2.30E-04 | 4.77827 | 1.14975 | 3.06373 |
| hsa_circRNA_100053 | 2.06E-05 | 6.20435 | 4.45132 | 3.03281 |
| hsa_circRNA_000585 | 9.50E-05 | 5.27237 | 2.31174 | 3.00467 |
| hsa_circRNA_001678 | 5.62E-06 | 7.10459 | 6.42291 | 2.97934 |
| hsa_circRNA_101144 | 7.49E-05 | 5.41742 | 2.65002 | 2.97055 |
| hsa_circRNA_101233 | 8.69E-07 | 8.72111 | 9.68417 | 2.96711 |
| hsa_circRNA_104854 | 1.62E-05 | 6.36768 | 4.81668 | 2.96082 |
| hsa_circRNA_101141 | 4.28E-05 | 5.7447 | 3.40675 | 2.94038 |
| hsa_circRNA_102482 | 1.68E-05 | 6.34653 | 4.76956 | 2.93925 |
| hsa_circRNA_101408 | 1.32E-06 | 8.34528 | 8.95878 | 2.93778 |
| hsa_circRNA_101303 | 5.59E-07 | 9.34107 | 10.83798 | 2.92363 |
| hsa_circRNA_101835 | 4.33E-04 | 4.44452 | 0.36038 | 2.91501 |
| hsa_circRNA_104650 | 2.23E-05 | 6.14457 | 4.3168 | 2.91344 |
| hsa_circRNA_101967 | 5.72E-04 | 4.30466 | 0.02965 | 2.91257 |
| hsa_circRNA_103114 | 2.08E-04 | 4.8277 | 1.26651 | 2.90642 |
| hsa_circRNA_100579 | 6.42E-07 | 9.14089 | 10.47118 | 2.90487 |
| hsa_circRNA_103951 | 1.19E-05 | 6.59092 | 5.31074 | 2.89529 |
| hsa_circRNA_002172 | 9.32E-05 | 5.28461 | 2.34034 | 2.89332 |
| hsa_circRNA_103598 | 6.48E-06 | 7.00146 | 6.20245 | 2.8846 |
| hsa_circRNA_101748 | 5.37E-04 | 4.33536 | 0.10222 | 2.86918 |
| hsa_circRNA_102483 | 1.56E-06 | 8.1425 | 8.55913 | 2.82783 |
| hsa_circRNA_001175 | 2.45E-04 | 4.74639 | 1.07444 | 2.81241 |
| hsa_circRNA_105027 | 7.55E-05 | 5.41076 | 2.63452 | 2.78482 |
| hsa_circRNA_103942 | 1.00E-04 | 5.24257 | 2.24205 | 2.76546 |
| hsa_circRNA_100191 | 1.22E-04 | 5.13073 | 1.97999 | 2.76183 |
| hsa_circRNA_101836 | 8.84E-04 | 4.08312 | -0.49291 | 2.73734 |
| hsa_circRNA_101903 | 1.39E-04 | 5.05635 | 1.80528 | 2.72681 |
| hsa_circRNA_100033 | 1.43E-05 | 6.46119 | 5.0244 | 2.72394 |
| hsa_circRNA_101002 | 4.44E-07 | 10.22198 | 12.38872 | 2.72327 |
| hsa_circRNA_101313 | 1.54E-04 | 5.00303 | 1.67986 | 2.71743 |
| hsa_circRNA_104762 | 1.76E-05 | 6.30918 | 4.6862 | 2.7148 |
| hsa_circRNA_100754 | 6.44E-06 | 7.00672 | 6.21374 | 2.7072 |
| hsa_circRNA_100257 | 1.33E-06 | 8.32562 | 8.92028 | 2.67538 |
| hsa_circRNA_000543 | 2.80E-04 | 4.67666 | 0.90956 | 2.62613 |
| hsa_circRNA_103948 | 7.85E-07 | 8.88715 | 9.99838 | 2.61989 |
| hsa_circRNA_104771 | 3.39E-06 | 7.49407 | 7.24225 | 2.61704 |
| hsa_circRNA_400070 | 1.50E-05 | 6.43025 | 4.9558 | 2.61138 |
| hsa_circRNA_100084 | 1.47E-04 | 5.02625 | 1.73449 | 2.60297 |
| hsa_circRNA_100980 | 1.12E-05 | 6.63848 | 5.41517 | 2.58869 |
| hsa_circRNA_103510 | 4.35E-04 | 4.44198 | 0.35437 | 2.58373 |
| hsa_circRNA_000481 | 9.15E-04 | 4.06639 | -0.53227 | 2.57943 |
| hsa_circRNA_101971 | 4.79E-06 | 7.23801 | 6.70595 | 2.56689 |
| hsa_circRNA_103066 | 8.01E-07 | 8.8456 | 9.92011 | 2.56618 |
| hsa_circRNA_001067 | 1.60E-04 | 4.97846 | 1.622 | 2.56505 |
| hsa_circRNA_101231 | 1.72E-05 | 6.32606 | 4.72388 | 2.56239 |
| hsa_circRNA_102034 | 4.85E-06 | 7.23026 | 6.68957 | 2.55662 |
| hsa_circRNA_100675 | 8.43E-06 | 6.83082 | 5.83453 | 2.5507 |
| hsa_circRNA_103017 | 3.91E-04 | 4.50104 | 0.4941 | 2.54809 |
| hsa_circRNA_101142 | 4.36E-04 | 4.44122 | 0.35257 | 2.54753 |
| hsa_circRNA_101592 | 1.42E-05 | 6.4698 | 5.04345 | 2.54427 |
| hsa_circRNA_102644 | 4.37E-04 | 4.43906 | 0.34746 | 2.54424 |
| hsa_circRNA_101201 | 6.28E-05 | 5.51528 | 2.87728 | 2.54015 |
| hsa_circRNA_102972 | 2.33E-05 | 6.11706 | 4.25475 | 2.53249 |
| hsa_circRNA_103548 | 6.07E-06 | 7.05133 | 6.30923 | 2.52209 |
| hsa_circRNA_000956 | 1.32E-04 | 5.08348 | 1.86904 | 2.51841 |
| hsa_circRNA_101837 | 3.56E-04 | 4.5501 | 0.61018 | 2.51829 |
| hsa_circRNA_000987 | 8.91E-04 | 4.07916 | -0.50224 | 2.50986 |
| hsa_circRNA_101873 | 1.56E-06 | 8.17244 | 8.61851 | 2.50896 |
| hsa_circRNA_102948 | 4.87E-07 | 9.53894 | 11.19522 | 2.50122 |
| hsa_circRNA_400048 | 4.27E-05 | 5.74837 | 3.41518 | 2.49833 |
| hsa_circRNA_104016 | 4.03E-04 | 4.48413 | 0.45408 | 2.4871 |
| hsa_circRNA_103506 | 5.61E-05 | 5.58039 | 3.02805 | 2.48536 |
| hsa_circRNA_101838 | 1.49E-04 | 5.02188 | 1.72422 | 2.48262 |
| hsa_circRNA_002044 | 6.32E-06 | 7.0236 | 6.24991 | 2.47575 |
| hsa_circRNA_103164 | 6.48E-04 | 4.23947 | -0.12432 | 2.47127 |
| hsa_circRNA_102559 | 3.28E-05 | 5.90595 | 3.77581 | 2.46522 |
| hsa_circRNA_400008 | 2.53E-04 | 4.73068 | 1.0373 | 2.45372 |
| hsa_circRNA_101094 | 2.32E-06 | 7.83249 | 7.93694 | 2.45339 |
| hsa_circRNA_104435 | 2.79E-04 | 4.6787 | 0.9144 | 2.45318 |
| hsa_circRNA_104939 | 1.51E-06 | 8.21695 | 8.70652 | 2.44881 |
| hsa_circRNA_103089 | 8.18E-05 | 5.35851 | 2.51281 | 2.43517 |
| hsa_circRNA_102404 | 3.23E-04 | 4.60192 | 0.7328 | 2.43402 |
| hsa_circRNA_104086 | 2.52E-04 | 4.73374 | 1.04453 | 2.41502 |
| hsa_circRNA_102395 | 6.87E-07 | 9.0646 | 10.32994 | 2.41492 |
| hsa_circRNA_104710 | 2.13E-05 | 6.17245 | 4.37959 | 2.39924 |
| hsa_circRNA_102234 | 1.51E-06 | 8.21349 | 8.69969 | 2.39816 |
| hsa_circRNA_000791 | 2.99E-05 | 5.96454 | 3.90922 | 2.39559 |
| hsa_circRNA_103026 | 1.43E-05 | 6.46093 | 5.02381 | 2.39444 |
| hsa_circRNA_100375 | 1.01E-04 | 5.23932 | 2.23445 | 2.38464 |
| hsa_circRNA_400091 | 3.45E-04 | 4.56624 | 0.64836 | 2.38153 |
| hsa_circRNA_104595 | 1.71E-05 | 6.33149 | 4.736 | 2.37353 |
| hsa_circRNA_102587 | 2.41E-05 | 6.0968 | 4.20901 | 2.36964 |
| hsa_circRNA_100213 | 7.07E-07 | 9.00007 | 10.20987 | 2.35753 |
| hsa_circRNA_102450 | 2.73E-06 | 7.66824 | 7.60179 | 2.34531 |
| hsa_circRNA_102541 | 2.48E-06 | 7.76835 | 7.80653 | 2.34056 |
| hsa_circRNA_102240 | 6.64E-05 | 5.48433 | 2.80549 | 2.33996 |
| hsa_circRNA_400087 | 6.20E-04 | 4.26419 | -0.06596 | 2.33885 |
| hsa_circRNA_400099 | 1.36E-03 | 3.87467 | -0.98189 | 2.33368 |
| hsa_circRNA_001046 | 2.48E-06 | 7.78608 | 7.84262 | 2.32854 |
| hsa_circRNA_103027 | 2.55E-05 | 6.06463 | 4.13626 | 2.32476 |
| hsa_circRNA_102430 | 4.07E-04 | 4.4782 | 0.44005 | 2.32225 |
| hsa_circRNA_000792 | 6.27E-06 | 7.02923 | 6.26195 | 2.32019 |
| hsa_circRNA_000996 | 1.02E-04 | 5.23286 | 2.21933 | 2.31845 |
| hsa_circRNA_105037 | 5.04E-07 | 9.46951 | 11.07047 | 2.31759 |
| hsa_circRNA_102721 | 1.31E-05 | 6.5276 | 5.17124 | 2.31618 |
| hsa_circRNA_104916 | 8.42E-05 | 5.34122 | 2.47251 | 2.30539 |
| hsa_circRNA_100849 | 5.66E-05 | 5.57491 | 3.01536 | 2.29438 |
| hsa_circRNA_101728 | 6.42E-07 | 9.15059 | 10.48907 | 2.29356 |
| hsa_circRNA_102513 | 1.61E-04 | 4.97595 | 1.6161 | 2.29286 |
| hsa_circRNA_101780 | 7.14E-06 | 6.94285 | 6.07653 | 2.29018 |
| hsa_circRNA_100974 | 8.96E-05 | 5.30687 | 2.39236 | 2.28877 |
| hsa_circRNA_101948 | 1.48E-04 | 5.02548 | 1.73269 | 2.28586 |
| hsa_circRNA_100956 | 4.44E-07 | 10.34488 | 12.59708 | 2.27914 |
| hsa_circRNA_100808 | 4.87E-07 | 9.53213 | 11.18301 | 2.2757 |
| hsa_circRNA_101003 | 5.13E-07 | 9.44366 | 11.02385 | 2.27209 |
| hsa_circRNA_101902 | 8.81E-07 | 8.6943 | 9.63308 | 2.26152 |
| hsa_circRNA_100833 | 1.01E-05 | 6.70213 | 5.55448 | 2.25991 |
| hsa_circRNA_104597 | 5.62E-05 | 5.57916 | 3.0252 | 2.25897 |
| hsa_circRNA_103829 | 2.09E-05 | 6.19156 | 4.42257 | 2.25689 |
| hsa_circRNA_104411 | 3.17E-04 | 4.61071 | 0.75358 | 2.25289 |
| hsa_circRNA_101670 | 1.91E-05 | 6.25646 | 4.56824 | 2.25157 |
| hsa_circRNA_104387 | 1.41E-03 | 3.85943 | -1.01748 | 2.2499 |
| hsa_circRNA_104326 | 5.47E-06 | 7.12114 | 6.45815 | 2.2472 |
| hsa_circRNA_103209 | 1.42E-06 | 8.2819 | 8.83449 | 2.24385 |
| hsa_circRNA_104833 | 4.44E-07 | 10.1424 | 12.25277 | 2.24332 |
| hsa_circRNA_101275 | 5.30E-05 | 5.61771 | 3.11428 | 2.24282 |
| hsa_circRNA_100960 | 4.16E-05 | 5.76426 | 3.45165 | 2.24024 |
| hsa_circRNA_100806 | 1.18E-06 | 8.45866 | 9.1797 | 2.23782 |
| hsa_circRNA_103994 | 1.34E-05 | 6.50397 | 5.11906 | 2.23728 |
| hsa_circRNA_103380 | 1.39E-05 | 6.48147 | 5.0693 | 2.237 |
| hsa_circRNA_102751 | 1.61E-05 | 6.37185 | 4.82598 | 2.23572 |
| hsa_circRNA_103812 | 4.87E-07 | 9.58543 | 11.27839 | 2.23419 |
| hsa_circRNA_101720 | 2.10E-06 | 7.89283 | 8.05912 | 2.22945 |
| hsa_circRNA_104268 | 1.34E-03 | 3.88284 | -0.9628 | 2.22346 |
| hsa_circRNA_101009 | 2.10E-06 | 7.89149 | 8.0564 | 2.22229 |
| hsa_circRNA_104084 | 7.09E-05 | 5.44569 | 2.71576 | 2.22212 |
| hsa_circRNA_102479 | 6.98E-05 | 5.45527 | 2.73801 | 2.22031 |
| hsa_circRNA_102962 | 8.08E-05 | 5.36717 | 2.53301 | 2.2156 |
| hsa_circRNA_104940 | 2.63E-03 | 3.56035 | -1.71076 | 2.21031 |
| hsa_circRNA_101470 | 5.19E-06 | 7.16759 | 6.55687 | 2.20995 |
| hsa_circRNA_102557 | 2.87E-06 | 7.62225 | 7.50728 | 2.20843 |
| hsa_circRNA_104056 | 1.22E-04 | 5.13161 | 1.98205 | 2.2082 |
| hsa_circRNA_103148 | 3.21E-05 | 5.91996 | 3.80774 | 2.20161 |
| hsa_circRNA_104620 | 4.87E-07 | 9.66137 | 11.41365 | 2.20104 |
| hsa_circRNA_104205 | 1.56E-06 | 8.15462 | 8.58318 | 2.19357 |
| hsa_circRNA_102011 | 2.12E-05 | 6.1822 | 4.40153 | 2.19065 |
| hsa_circRNA_100192 | 7.29E-04 | 4.17862 | -0.26792 | 2.18109 |
| hsa_circRNA_102402 | 3.30E-05 | 5.90041 | 3.76317 | 2.17862 |
| hsa_circRNA_103572 | 1.15E-03 | 3.95884 | -0.78486 | 2.17598 |
| hsa_circRNA_101227 | 2.88E-04 | 4.66301 | 0.87728 | 2.17525 |
| hsa_circRNA_102211 | 3.59E-07 | 11.27155 | 14.1081 | 2.1746 |
| hsa_circRNA_100920 | 1.71E-05 | 6.33284 | 4.73901 | 2.17278 |
| hsa_circRNA_100528 | 7.07E-07 | 8.98763 | 10.18666 | 2.16887 |
| hsa_circRNA_104917 | 2.08E-05 | 6.19424 | 4.4286 | 2.16739 |
| hsa_circRNA_101314 | 1.37E-05 | 6.48987 | 5.08788 | 2.16666 |
| hsa_circRNA_103243 | 1.18E-06 | 8.47397 | 9.20938 | 2.16271 |
| hsa_circRNA_100445 | 4.37E-05 | 5.73136 | 3.37609 | 2.16271 |
| hsa_circRNA_100177 | 3.82E-04 | 4.51346 | 0.52349 | 2.16088 |
| hsa_circRNA_100583 | 8.09E-04 | 4.12622 | -0.39143 | 2.16087 |
| hsa_circRNA_103547 | 2.06E-05 | 6.20238 | 4.44689 | 2.16019 |
| hsa_circRNA_101436 | 9.50E-05 | 5.27219 | 2.31132 | 2.15791 |
| hsa_circRNA_103123 | 6.14E-07 | 9.19972 | 10.57954 | 2.14529 |
| hsa_circRNA_100876 | 2.07E-03 | 3.6741 | -1.44845 | 2.13611 |
| hsa_circRNA_101915 | 8.08E-07 | 8.7909 | 9.81669 | 2.13347 |
| hsa_circRNA_101031 | 1.65E-04 | 4.9625 | 1.58442 | 2.13304 |
| hsa_circRNA_103998 | 6.70E-07 | 9.08804 | 10.37343 | 2.12982 |
| hsa_circRNA_400025 | 3.85E-04 | 4.51001 | 0.51532 | 2.12653 |
| hsa_circRNA_100620 | 2.48E-06 | 7.77347 | 7.81696 | 2.12588 |
| hsa_circRNA_100968 | 4.75E-06 | 7.24647 | 6.72381 | 2.11964 |
| hsa_circRNA_103177 | 2.24E-04 | 4.79283 | 1.18416 | 2.1132 |
| hsa_circRNA_102455 | 5.39E-06 | 7.1341 | 6.48573 | 2.11253 |
| hsa_circRNA_101325 | 8.97E-07 | 8.67312 | 9.59265 | 2.1084 |
| hsa_circRNA_104134 | 1.84E-04 | 4.89883 | 1.43434 | 2.10815 |
| hsa_circRNA_102888 | 8.06E-04 | 4.1286 | -0.38583 | 2.1034 |
| hsa_circRNA_100188 | 1.77E-06 | 8.0286 | 8.33211 | 2.10334 |
| hsa_circRNA_001012 | 7.95E-06 | 6.87383 | 5.92763 | 2.10262 |
| hsa_circRNA_102707 | 1.68E-05 | 6.34218 | 4.75984 | 2.1007 |
| hsa_circRNA_102139 | 2.10E-06 | 7.90777 | 8.08929 | 2.09711 |
| hsa_circRNA_103757 | 1.38E-04 | 5.06121 | 1.81671 | 2.0932 |
| hsa_circRNA_101367 | 1.22E-05 | 6.56772 | 5.25969 | 2.09067 |
| hsa_circRNA_102020 | 8.71E-07 | 8.70625 | 9.65586 | 2.08466 |
| hsa_circRNA_103140 | 4.87E-07 | 9.70396 | 11.48916 | 2.08085 |
| hsa_circRNA_101478 | 5.04E-07 | 9.47292 | 11.0766 | 2.07974 |
| hsa_circRNA_100910 | 4.75E-06 | 7.25062 | 6.73257 | 2.07841 |
| hsa_circRNA_100013 | 2.59E-05 | 6.05338 | 4.11081 | 2.07103 |
| hsa_circRNA_001498 | 2.27E-04 | 4.78717 | 1.17079 | 2.06958 |
| hsa_circRNA_104758 | 2.28E-05 | 6.1291 | 4.28192 | 2.06856 |
| hsa_circRNA_101486 | 5.20E-07 | 9.42092 | 10.98278 | 2.06718 |
| hsa_circRNA_103753 | 2.80E-06 | 7.6501 | 7.56455 | 2.06472 |
| hsa_circRNA_103987 | 4.04E-06 | 7.37485 | 6.99369 | 2.06062 |
| hsa_circRNA_101891 | 9.27E-06 | 6.76765 | 5.69733 | 2.0593 |
| hsa_circRNA_104821 | 4.00E-04 | 4.48825 | 0.46384 | 2.05868 |
| hsa_circRNA_100541 | 3.15E-04 | 4.61699 | 0.76845 | 2.05859 |
| hsa_circRNA_100593 | 4.29E-05 | 5.74292 | 3.40267 | 2.05787 |
| hsa_circRNA_100970 | 9.97E-06 | 6.71764 | 5.58835 | 2.05564 |
| hsa_circRNA_102140 | 1.19E-06 | 8.45139 | 9.16557 | 2.05394 |
| hsa_circRNA_102003 | 5.68E-06 | 7.09542 | 6.40336 | 2.05378 |
| hsa_circRNA_103285 | 9.96E-04 | 4.02679 | -0.62537 | 2.05376 |
| hsa_circRNA_101778 | 3.87E-05 | 5.80815 | 3.55228 | 2.05255 |
| hsa_circRNA_103827 | 2.69E-05 | 6.03072 | 4.05947 | 2.05241 |
| hsa_circRNA_103980 | 7.74E-06 | 6.89006 | 5.9627 | 2.05148 |
| hsa_circRNA_104507 | 1.02E-03 | 4.01485 | -0.65341 | 2.04415 |
| hsa_circRNA_104412 | 7.36E-04 | 4.17362 | -0.27972 | 2.04277 |
| hsa_circRNA_102018 | 8.43E-06 | 6.8346 | 5.84273 | 2.03532 |
| hsa_circRNA_104930 | 7.34E-07 | 8.93886 | 10.09545 | 2.03433 |
| hsa_circRNA_101850 | 4.87E-07 | 9.74765 | 11.56638 | 2.02655 |
| hsa_circRNA_001465 | 2.76E-05 | 6.01818 | 4.03104 | 2.02401 |
| hsa_circRNA_101425 | 5.85E-07 | 9.26206 | 10.69385 | 2.02202 |
| hsa_circRNA_100696 | 2.78E-03 | 3.53305 | -1.77343 | 2.02046 |
| hsa_circRNA_103514 | 5.18E-03 | 3.24368 | -2.42941 | 2.01976 |
| hsa_circRNA_104722 | 1.07E-05 | 6.66418 | 5.4715 | 2.01814 |
| hsa_circRNA_104230 | 7.07E-07 | 8.97527 | 10.16357 | 2.01286 |
| hsa_circRNA_102465 | 3.57E-04 | 4.54882 | 0.60716 | 2.00977 |
| hsa_circRNA_100840 | 1.56E-06 | 8.1429 | 8.55992 | 2.00546 |
| hsa_circRNA_103087 | 2.80E-05 | 6.01026 | 4.01306 | 2.00318 |
| hsa_circRNA_100875 | 4.87E-07 | 9.71669 | 11.51168 | 2.00122 |
| hsa_circRNA_104820 | 2.99E-04 | 4.64313 | 0.83029 | 2.00071 |
| hsa_circRNA_101050 | 5.10E-05 | 5.64124 | 3.16858 | 1.99995 |
| hsa_circRNA_400043 | 6.59E-04 | 4.23072 | -0.14499 | 1.99892 |
| hsa_circRNA_100834 | 6.48E-06 | 7.00053 | 6.20046 | 1.99838 |
| hsa_circRNA_104720 | 6.42E-07 | 9.12395 | 10.43989 | 1.99704 |
| hsa_circRNA_103213 | 3.82E-04 | 4.51354 | 0.52368 | 1.99335 |
| hsa_circRNA_100355 | 7.41E-04 | 4.16992 | -0.28844 | 1.98901 |
| hsa_circRNA_101067 | 1.24E-04 | 5.12232 | 1.96025 | 1.9823 |
| hsa_circRNA_101810 | 2.87E-06 | 7.62681 | 7.51667 | 1.9787 |
| hsa_circRNA_102745 | 5.74E-06 | 7.08738 | 6.38622 | 1.97729 |
| hsa_circRNA_101057 | 1.40E-04 | 5.05237 | 1.79593 | 1.97074 |
| hsa_circRNA_104250 | 4.87E-07 | 9.72802 | 11.53172 | 1.96661 |
| hsa_circRNA_102411 | 1.36E-03 | 3.87709 | -0.97624 | 1.96642 |
| hsa_circRNA_103830 | 7.14E-07 | 8.96223 | 10.1392 | 1.96634 |
| hsa_circRNA_100440 | 5.02E-06 | 7.20506 | 6.63628 | 1.96464 |
| hsa_circRNA_103579 | 7.07E-07 | 8.97594 | 10.16483 | 1.96249 |
| hsa_circRNA_101061 | 8.04E-07 | 8.81231 | 9.85723 | 1.96239 |
| hsa_circRNA_102092 | 1.32E-05 | 6.51627 | 5.14623 | 1.96179 |
| hsa_circRNA_000442 | 1.91E-05 | 6.25496 | 4.56487 | 1.96066 |
| hsa_circRNA_105024 | 3.15E-05 | 5.93527 | 3.84261 | 1.95928 |
| hsa_circRNA_001233 | 1.05E-05 | 6.6801 | 5.50632 | 1.9578 |
| hsa_circRNA_101251 | 7.50E-05 | 5.41604 | 2.6468 | 1.95558 |
| hsa_circRNA_001259 | 1.32E-06 | 8.34881 | 8.96568 | 1.95531 |
| hsa_circRNA_101458 | 4.44E-07 | 10.27966 | 12.48674 | 1.95278 |
| hsa_circRNA_104936 | 6.32E-06 | 7.02484 | 6.25255 | 1.9525 |
| hsa_circRNA_103605 | 1.44E-03 | 3.84864 | -1.04268 | 1.94927 |
| hsa_circRNA_400021 | 3.18E-06 | 7.54213 | 7.34188 | 1.94755 |
| hsa_circRNA_104645 | 3.50E-03 | 3.42635 | -2.01716 | 1.94624 |
| hsa_circRNA_104052 | 6.28E-03 | 3.15396 | -2.62918 | 1.94559 |
| hsa_circRNA_000638 | 4.04E-06 | 7.37354 | 6.99094 | 1.94407 |
| hsa_circRNA_100425 | 9.82E-06 | 6.72898 | 5.6131 | 1.94241 |
| hsa_circRNA_400011 | 5.02E-06 | 7.20811 | 6.64273 | 1.93859 |
| hsa_circRNA_101965 | 6.79E-06 | 6.97347 | 6.14238 | 1.93788 |
| hsa_circRNA_100655 | 5.85E-05 | 5.55669 | 2.97321 | 1.93687 |
| hsa_circRNA_104983 | 4.44E-07 | 10.04284 | 12.08155 | 1.93483 |
| hsa_circRNA_103417 | 3.49E-05 | 5.8693 | 3.69215 | 1.93442 |
| hsa_circRNA_000750 | 5.59E-06 | 7.10979 | 6.43399 | 1.93257 |
| hsa_circRNA_102914 | 7.46E-06 | 6.91308 | 6.01239 | 1.93001 |
| hsa_circRNA_103328 | 4.04E-06 | 7.37079 | 6.98519 | 1.92904 |
| hsa_circRNA_103797 | 3.72E-05 | 5.82927 | 3.60063 | 1.92136 |
| hsa_circRNA_102121 | 2.24E-04 | 4.79316 | 1.18493 | 1.9207 |
| hsa_circRNA_101571 | 1.99E-03 | 3.69212 | -1.40673 | 1.92064 |
| hsa_circRNA_104574 | 1.74E-05 | 6.31455 | 4.6982 | 1.9198 |
| hsa_circRNA_104838 | 1.17E-05 | 6.60594 | 5.34375 | 1.9175 |
| hsa_circRNA_104929 | 8.57E-07 | 8.74701 | 9.73342 | 1.91576 |
| hsa_circRNA_104285 | 8.04E-07 | 8.83378 | 9.89779 | 1.9157 |
| hsa_circRNA_101307 | 7.07E-07 | 9.00788 | 10.22443 | 1.91288 |
| hsa_circRNA_103387 | 1.08E-04 | 5.19904 | 2.14016 | 1.91152 |
| hsa_circRNA_100004 | 1.88E-05 | 6.26631 | 4.5903 | 1.90405 |
| hsa_circRNA_102546 | 3.59E-07 | 11.05463 | 13.76367 | 1.90394 |
| hsa_circRNA_103669 | 4.35E-06 | 7.30895 | 6.85545 | 1.9005 |
| hsa_circRNA_101839 | 2.21E-03 | 3.6407 | -1.52567 | 1.90014 |
| hsa_circRNA_104692 | 2.40E-05 | 6.09867 | 4.21324 | 1.89615 |
| hsa_circRNA_101080 | 2.55E-06 | 7.73792 | 7.74445 | 1.89226 |
| hsa_circRNA_103999 | 1.11E-03 | 3.97265 | -0.75247 | 1.89113 |
| hsa_circRNA_101969 | 3.24E-04 | 4.5991 | 0.72611 | 1.8897 |
| hsa_circRNA_102415 | 7.50E-05 | 5.41539 | 2.64529 | 1.88958 |
| hsa_circRNA_102804 | 1.06E-05 | 6.67184 | 5.48826 | 1.88337 |
| hsa_circRNA_103086 | 9.09E-06 | 6.78325 | 5.73128 | 1.88285 |
| hsa_circRNA_103519 | 1.87E-04 | 4.89068 | 1.41513 | 1.88167 |
| hsa_circRNA_000764 | 1.18E-03 | 3.94531 | -0.81658 | 1.88115 |
| hsa_circRNA_100139 | 4.65E-04 | 4.40705 | 0.27174 | 1.88102 |
| hsa_circRNA_103808 | 3.79E-04 | 4.51788 | 0.53394 | 1.88065 |
| hsa_circRNA_001100 | 1.51E-04 | 5.01257 | 1.7023 | 1.88009 |
| hsa_circRNA_400040 | 1.66E-02 | 2.69995 | -3.60555 | 1.87781 |
| hsa_circRNA_101924 | 1.00E-03 | 4.02302 | -0.63423 | 1.8769 |
| hsa_circRNA_101202 | 5.59E-07 | 9.31998 | 10.79958 | 1.87513 |
| hsa_circRNA_104928 | 6.97E-04 | 4.20073 | -0.21577 | 1.87501 |
| hsa_circRNA_101505 | 1.56E-06 | 8.14299 | 8.5601 | 1.87376 |
| hsa_circRNA_101346 | 8.01E-07 | 8.85918 | 9.9457 | 1.87178 |
| hsa_circRNA_101068 | 2.77E-04 | 4.68332 | 0.92533 | 1.87159 |
| hsa_circRNA_104161 | 3.48E-04 | 4.56273 | 0.64006 | 1.87106 |
| hsa_circRNA_102760 | 4.60E-07 | 9.93529 | 11.89517 | 1.86753 |
| hsa_circRNA_001379 | 4.68E-04 | 4.40337 | 0.26304 | 1.86742 |
| hsa_circRNA_103059 | 2.01E-06 | 7.9493 | 8.17298 | 1.86188 |
| hsa_circRNA_102070 | 8.83E-07 | 8.68679 | 9.61875 | 1.86028 |
| hsa_circRNA_100507 | 1.51E-05 | 6.42157 | 4.93651 | 1.85982 |
| hsa_circRNA_102079 | 2.73E-06 | 7.66965 | 7.60468 | 1.85751 |
| hsa_circRNA_103220 | 1.32E-06 | 8.35769 | 8.98304 | 1.8567 |
| hsa_circRNA_102839 | 2.48E-06 | 7.77906 | 7.82833 | 1.85452 |
| hsa_circRNA_100913 | 6.79E-05 | 5.46989 | 2.77197 | 1.85276 |
| hsa_circRNA_102215 | 1.42E-04 | 5.04767 | 1.78488 | 1.85214 |
| hsa_circRNA_102451 | 3.24E-03 | 3.46181 | -1.93639 | 1.85207 |
| hsa_circRNA_102554 | 7.09E-05 | 5.4469 | 2.71857 | 1.85199 |
| hsa_circRNA_102605 | 8.82E-06 | 6.80215 | 5.77232 | 1.85046 |
| hsa_circRNA_102417 | 7.98E-05 | 5.37796 | 2.55816 | 1.84973 |
| hsa_circRNA_102148 | 5.52E-05 | 5.59175 | 3.0543 | 1.84818 |
| hsa_circRNA_103371 | 1.21E-06 | 8.43636 | 9.13638 | 1.84794 |
| hsa_circRNA_103597 | 5.43E-07 | 9.38316 | 10.9144 | 1.84661 |
| hsa_circRNA_101888 | 1.15E-05 | 6.62084 | 5.37647 | 1.84617 |
| hsa_circRNA_102586 | 1.48E-06 | 8.23596 | 8.74404 | 1.84454 |
| hsa_circRNA_103910 | 8.43E-05 | 5.33972 | 2.469 | 1.84232 |
| hsa_circRNA_103221 | 1.13E-06 | 8.53142 | 9.32052 | 1.83977 |
| hsa_circRNA_102886 | 2.95E-04 | 4.65031 | 0.84727 | 1.83839 |
| hsa_circRNA_100832 | 2.03E-04 | 4.84174 | 1.29964 | 1.83671 |
| hsa_circRNA_102231 | 1.23E-06 | 8.4185 | 9.10164 | 1.8304 |
| hsa_circRNA_103668 | 6.98E-04 | 4.19971 | -0.21818 | 1.82899 |
| hsa_circRNA_100709 | 1.61E-05 | 6.3734 | 4.82942 | 1.82868 |
| hsa_circRNA_104975 | 2.87E-05 | 5.98978 | 3.96657 | 1.82854 |
| hsa_circRNA_101691 | 1.39E-03 | 3.86579 | -1.00264 | 1.82738 |
| hsa_circRNA_102008 | 2.97E-06 | 7.59561 | 7.45238 | 1.82729 |
| hsa_circRNA_001671 | 6.43E-05 | 5.5034 | 2.84973 | 1.8272 |
| hsa_circRNA_102954 | 2.96E-04 | 4.6492 | 0.84464 | 1.82617 |
| hsa_circRNA_104433 | 5.65E-07 | 9.29277 | 10.74996 | 1.82599 |
| hsa_circRNA_104819 | 9.02E-05 | 5.30376 | 2.38509 | 1.82519 |
| hsa_circRNA_100209 | 2.19E-05 | 6.15417 | 4.33843 | 1.82285 |
| hsa_circRNA_104708 | 9.65E-03 | 2.95438 | -3.06615 | 1.81982 |
| hsa_circRNA_000598 | 3.75E-03 | 3.39429 | -2.09001 | 1.81632 |
| hsa_circRNA_103696 | 5.92E-07 | 9.23157 | 10.638 | 1.81601 |
| hsa_circRNA_104168 | 5.32E-06 | 7.1428 | 6.50423 | 1.81545 |
| hsa_circRNA_101721 | 1.50E-05 | 6.43003 | 4.9553 | 1.81525 |
| hsa_circRNA_400056 | 2.88E-06 | 7.61664 | 7.49573 | 1.81487 |
| hsa_circRNA_002149 | 2.80E-06 | 7.65266 | 7.56981 | 1.81479 |
| hsa_circRNA_101138 | 2.64E-06 | 7.7058 | 7.67877 | 1.81424 |
| hsa_circRNA_104575 | 1.84E-05 | 6.28109 | 4.62339 | 1.81358 |
| hsa_circRNA_102961 | 3.56E-06 | 7.46096 | 7.17342 | 1.81351 |
| hsa_circRNA_103870 | 1.17E-04 | 5.15469 | 2.03619 | 1.8127 |
| hsa_circRNA_104970 | 1.68E-05 | 6.34734 | 4.77136 | 1.81252 |
| hsa_circRNA_104394 | 1.54E-04 | 5.0034 | 1.68073 | 1.81147 |
| hsa_circRNA_103038 | 2.18E-05 | 6.15936 | 4.35013 | 1.81113 |
| hsa_circRNA_104165 | 1.49E-03 | 3.83158 | -1.08249 | 1.81106 |
| hsa_circRNA_104434 | 4.87E-07 | 9.52027 | 11.16174 | 1.81074 |
| hsa_circRNA_102292 | 1.61E-05 | 6.37377 | 4.83024 | 1.81026 |
| hsa_circRNA_104540 | 1.04E-05 | 6.68583 | 5.51885 | 1.80757 |
| hsa_circRNA_103023 | 8.21E-05 | 5.35645 | 2.50802 | 1.80689 |
| hsa_circRNA_101820 | 1.62E-03 | 3.79062 | -1.17796 | 1.80421 |
| hsa_circRNA_104170 | 7.98E-05 | 5.37774 | 2.55764 | 1.80368 |
| hsa_circRNA_000031 | 5.93E-04 | 4.28734 | -0.01127 | 1.80019 |
| hsa_circRNA_100027 | 6.84E-05 | 5.46527 | 2.76123 | 1.79944 |
| hsa_circRNA_104497 | 6.54E-06 | 6.99451 | 6.18755 | 1.7978 |
| hsa_circRNA_101857 | 1.38E-05 | 6.48778 | 5.08325 | 1.79761 |
| hsa_circRNA_100011 | 2.05E-05 | 6.21043 | 4.46498 | 1.797 |
| hsa_circRNA_103041 | 4.03E-06 | 7.37738 | 6.99897 | 1.79491 |
| hsa_circRNA_103562 | 6.72E-06 | 6.98005 | 6.1565 | 1.79462 |
| hsa_circRNA_100641 | 5.32E-06 | 7.14319 | 6.50505 | 1.79415 |
| hsa_circRNA_103124 | 4.83E-04 | 4.38605 | 0.22207 | 1.79227 |
| hsa_circRNA_102984 | 1.35E-04 | 5.07043 | 1.83837 | 1.79224 |
| hsa_circRNA_102173 | 1.96E-06 | 7.96702 | 8.20861 | 1.79133 |
| hsa_circRNA_103322 | 2.48E-05 | 6.07896 | 4.1687 | 1.79126 |
| hsa_circRNA_102229 | 1.11E-05 | 6.64627 | 5.43226 | 1.79085 |
| hsa_circRNA_103490 | 1.56E-06 | 8.15091 | 8.57583 | 1.78969 |
| hsa_circRNA_101848 | 5.59E-07 | 9.32964 | 10.81718 | 1.78937 |
| hsa_circRNA_100385 | 1.16E-06 | 8.50546 | 9.27036 | 1.78626 |
| hsa_circRNA_103244 | 2.44E-06 | 7.80058 | 7.87214 | 1.78514 |
| hsa_circRNA_104443 | 3.72E-05 | 5.82983 | 3.60191 | 1.78466 |
| hsa_circRNA_101976 | 5.73E-04 | 4.30231 | 0.02409 | 1.7828 |
| hsa_circRNA_100919 | 7.07E-07 | 8.98362 | 10.17916 | 1.78235 |
| hsa_circRNA_102460 | 1.34E-04 | 5.07662 | 1.85292 | 1.78188 |
| hsa_circRNA_104669 | 3.08E-06 | 7.56711 | 7.39355 | 1.7812 |
| hsa_circRNA_102806 | 4.82E-04 | 4.3874 | 0.22526 | 1.78109 |
| hsa_circRNA_100987 | 1.96E-04 | 4.85969 | 1.34201 | 1.78102 |
| hsa_circRNA_102382 | 1.70E-06 | 8.0798 | 8.43439 | 1.78046 |
| hsa_circRNA_104942 | 1.01E-05 | 6.70749 | 5.56619 | 1.77691 |
| hsa_circRNA_100017 | 5.77E-05 | 5.56362 | 2.98924 | 1.7758 |
| hsa_circRNA_101514 | 5.61E-05 | 5.58082 | 3.02903 | 1.77517 |
| hsa_circRNA_101746 | 1.85E-05 | 6.277 | 4.61422 | 1.77456 |
| hsa_circRNA_102581 | 2.10E-06 | 7.89748 | 8.06851 | 1.77443 |
| hsa_circRNA_101517 | 4.87E-07 | 9.55748 | 11.22842 | 1.77391 |
| hsa_circRNA_102265 | 5.20E-06 | 7.1645 | 6.55031 | 1.77371 |
| hsa_circRNA_101695 | 3.95E-04 | 4.4963 | 0.48288 | 1.77371 |
| hsa_circRNA_104055 | 6.52E-05 | 5.49447 | 2.82901 | 1.77292 |
| hsa_circRNA_103988 | 2.15E-05 | 6.16652 | 4.36625 | 1.77222 |
| hsa_circRNA_103491 | 1.56E-06 | 8.16285 | 8.5995 | 1.771 |
| hsa_circRNA_102033 | 1.02E-04 | 5.23217 | 2.21773 | 1.77059 |
| hsa_circRNA_101683 | 1.14E-06 | 8.52131 | 9.301 | 1.76632 |
| hsa_circRNA_001205 | 1.11E-04 | 5.17994 | 2.0954 | 1.76284 |
| hsa_circRNA_100984 | 4.37E-05 | 5.73084 | 3.3749 | 1.76283 |
| hsa_circRNA_001108 | 1.84E-04 | 4.90054 | 1.43839 | 1.76084 |
| hsa_circRNA_103571 | 4.42E-04 | 4.43292 | 0.33293 | 1.75955 |
| hsa_circRNA_104490 | 3.51E-04 | 4.55789 | 0.62861 | 1.75873 |
| hsa_circRNA_102100 | 3.16E-04 | 4.61502 | 0.76378 | 1.75631 |
| hsa_circRNA_103290 | 8.65E-04 | 4.09478 | -0.46548 | 1.75541 |
| hsa_circRNA_400045 | 1.45E-05 | 6.45269 | 5.00555 | 1.75515 |
| hsa_circRNA_100715 | 3.74E-06 | 7.42761 | 7.10393 | 1.75411 |
| hsa_circRNA_101513 | 4.77E-05 | 5.68051 | 3.2591 | 1.75153 |
| hsa_circRNA_104103 | 5.31E-05 | 5.61614 | 3.11065 | 1.74665 |
| hsa_circRNA_100015 | 8.32E-05 | 5.35 | 2.49298 | 1.74648 |
| hsa_circRNA_103159 | 1.56E-06 | 8.18515 | 8.64367 | 1.74531 |
| hsa_circRNA_103219 | 1.43E-06 | 8.27154 | 8.81412 | 1.74435 |
| hsa_circRNA_101243 | 1.85E-05 | 6.27845 | 4.61748 | 1.74347 |
| hsa_circRNA_104106 | 1.88E-05 | 6.26652 | 4.59077 | 1.74331 |
| hsa_circRNA_100994 | 1.77E-06 | 8.02961 | 8.33414 | 1.74213 |
| hsa_circRNA_104506 | 2.00E-04 | 4.84875 | 1.31618 | 1.74175 |
| hsa_circRNA_001038 | 1.81E-03 | 3.73772 | -1.30095 | 1.74028 |
| hsa_circRNA_101798 | 2.13E-03 | 3.66022 | -1.48055 | 1.73849 |
| hsa_circRNA_104032 | 2.42E-06 | 7.80834 | 7.8879 | 1.73847 |
| hsa_circRNA_100010 | 5.21E-05 | 5.62839 | 3.13893 | 1.73733 |
| hsa_circRNA_102856 | 2.53E-04 | 4.73052 | 1.03691 | 1.73601 |
| hsa_circRNA_103532 | 1.75E-06 | 8.05542 | 8.38573 | 1.73585 |
| hsa_circRNA_101492 | 1.21E-04 | 5.13845 | 1.9981 | 1.73508 |
| hsa_circRNA_101338 | 4.87E-07 | 9.8105 | 11.67702 | 1.73468 |
| hsa_circRNA_101051 | 2.17E-03 | 3.65037 | -1.50331 | 1.72922 |
| hsa_circRNA_103570 | 7.51E-04 | 4.16303 | -0.30468 | 1.72916 |
| hsa_circRNA_100625 | 2.55E-06 | 7.73624 | 7.741 | 1.72792 |
| hsa_circRNA_103176 | 1.45E-03 | 3.84599 | -1.04887 | 1.72737 |
| hsa_circRNA_101741 | 1.88E-05 | 6.26897 | 4.59625 | 1.72723 |
| hsa_circRNA_100737 | 2.98E-06 | 7.5878 | 7.43628 | 1.72686 |
| hsa_circRNA_103359 | 2.85E-05 | 5.996 | 3.98071 | 1.72579 |
| hsa_circRNA_102254 | 1.01E-05 | 6.70828 | 5.56793 | 1.72409 |
| hsa_circRNA_104718 | 2.22E-05 | 6.14773 | 4.32393 | 1.72173 |
| hsa_circRNA_100917 | 5.14E-05 | 5.63527 | 3.15482 | 1.71748 |
| hsa_circRNA_100800 | 1.32E-06 | 8.38054 | 9.02766 | 1.71592 |
| hsa_circRNA_400051 | 2.48E-06 | 7.76764 | 7.80507 | 1.71504 |
| hsa_circRNA_103862 | 4.44E-07 | 10.03103 | 12.06117 | 1.71377 |
| hsa_circRNA_104689 | 4.15E-04 | 4.46597 | 0.41111 | 1.71355 |
| hsa_circRNA_100550 | 2.88E-05 | 5.98657 | 3.95929 | 1.71314 |
| hsa_circRNA_400029 | 1.61E-03 | 3.79433 | -1.16932 | 1.71073 |
| hsa_circRNA_104045 | 1.51E-06 | 8.21913 | 8.71084 | 1.71047 |
| hsa_circRNA_102673 | 3.42E-05 | 5.87967 | 3.71584 | 1.70931 |
| hsa_circRNA_102747 | 4.80E-03 | 3.2772 | -2.35427 | 1.70569 |
| hsa_circRNA_101161 | 9.74E-05 | 5.2581 | 2.27838 | 1.70501 |
| hsa_circRNA_000855 | 2.73E-06 | 7.66914 | 7.60365 | 1.7011 |
| hsa_circRNA_101719 | 3.53E-05 | 5.86471 | 3.68167 | 1.69894 |
| hsa_circRNA_001754 | 2.78E-06 | 7.65763 | 7.58002 | 1.6988 |
| hsa_circRNA_100969 | 1.76E-05 | 6.30889 | 4.68554 | 1.69831 |
| hsa_circRNA_101675 | 5.41E-05 | 5.6052 | 3.08539 | 1.69542 |
| hsa_circRNA_102299 | 1.73E-05 | 6.31966 | 4.70959 | 1.69531 |
| hsa_circRNA_101827 | 1.54E-05 | 6.4101 | 4.91104 | 1.69405 |
| hsa_circRNA_101639 | 2.55E-06 | 7.74538 | 7.75968 | 1.68961 |
| hsa_circRNA_104368 | 1.32E-06 | 8.36674 | 9.00073 | 1.68873 |
| hsa_circRNA_104704 | 8.40E-03 | 3.0205 | -2.92261 | 1.68862 |
| hsa_circRNA_100391 | 3.38E-04 | 4.57771 | 0.67551 | 1.68681 |
| hsa_circRNA_100731 | 8.95E-04 | 4.07673 | -0.50795 | 1.68507 |
| hsa_circRNA_001886 | 5.02E-06 | 7.2078 | 6.64206 | 1.68384 |
| hsa_circRNA_102437 | 8.14E-07 | 8.78 | 9.79604 | 1.68378 |
| hsa_circRNA_102474 | 4.35E-06 | 7.31204 | 6.86193 | 1.67936 |
| hsa_circRNA_102447 | 4.65E-06 | 7.26648 | 6.76603 | 1.67886 |
| hsa_circRNA_100269 | 3.39E-04 | 4.57613 | 0.67177 | 1.67871 |
| hsa_circRNA_100208 | 5.59E-06 | 7.1084 | 6.43103 | 1.67759 |
| hsa_circRNA_104361 | 1.69E-04 | 4.9466 | 1.54695 | 1.67745 |
| hsa_circRNA_102754 | 2.18E-06 | 7.87039 | 8.01374 | 1.67735 |
| hsa_circRNA_101316 | 9.75E-04 | 4.03681 | -0.60184 | 1.67701 |
| hsa_circRNA_104764 | 6.98E-04 | 4.19965 | -0.2183 | 1.67557 |
| hsa_circRNA_104885 | 8.95E-06 | 6.79328 | 5.75306 | 1.67553 |
| hsa_circRNA_103834 | 1.56E-06 | 8.12724 | 8.52883 | 1.67418 |
| hsa_circRNA_101252 | 2.09E-06 | 7.92294 | 8.11988 | 1.67365 |
| hsa_circRNA_101396 | 1.32E-05 | 6.50872 | 5.12956 | 1.67279 |
| hsa_circRNA_100222 | 4.49E-05 | 5.71658 | 3.34213 | 1.6723 |
| hsa_circRNA_101309 | 1.56E-06 | 8.14004 | 8.55425 | 1.67074 |
| hsa_circRNA_100270 | 2.04E-06 | 7.93828 | 8.15079 | 1.66799 |
| hsa_circRNA_104256 | 2.55E-06 | 7.73965 | 7.74797 | 1.66736 |
| hsa_circRNA_103383 | 9.27E-06 | 6.76775 | 5.69756 | 1.66613 |
| hsa_circRNA_104761 | 7.60E-04 | 4.15567 | -0.32204 | 1.66561 |
| hsa_circRNA_100805 | 2.10E-06 | 7.90256 | 8.07877 | 1.66483 |
| hsa_circRNA_103682 | 2.06E-04 | 4.8341 | 1.28161 | 1.66451 |
| hsa_circRNA_100735 | 1.75E-06 | 8.04781 | 8.37053 | 1.66367 |
| hsa_circRNA_103193 | 1.22E-05 | 6.56818 | 5.26071 | 1.66169 |
| hsa_circRNA_104826 | 4.35E-06 | 7.31497 | 6.86809 | 1.66094 |
| hsa_circRNA_104619 | 4.87E-07 | 9.64932 | 11.39223 | 1.66032 |
| hsa_circRNA_102264 | 1.22E-05 | 6.57334 | 5.27207 | 1.659 |
| hsa_circRNA_102424 | 5.68E-06 | 7.09429 | 6.40096 | 1.65842 |
| hsa_circRNA_105035 | 3.61E-05 | 5.84943 | 3.64675 | 1.658 |
| hsa_circRNA_104439 | 9.91E-06 | 6.72237 | 5.59868 | 1.6574 |
| hsa_circRNA_000945 | 4.71E-06 | 7.25842 | 6.74903 | 1.65582 |
| hsa_circRNA_100708 | 4.94E-04 | 4.37518 | 0.19637 | 1.65513 |
| hsa_circRNA_102952 | 2.66E-06 | 7.69413 | 7.65487 | 1.65508 |
| hsa_circRNA_101698 | 3.20E-06 | 7.53672 | 7.33068 | 1.655 |
| hsa_circRNA_103442 | 3.34E-05 | 5.89254 | 3.74522 | 1.65183 |
| hsa_circRNA_102960 | 8.84E-05 | 5.31416 | 2.40936 | 1.65058 |
| hsa_circRNA_102158 | 4.63E-04 | 4.41035 | 0.27954 | 1.65045 |
| hsa_circRNA_104569 | 1.76E-03 | 3.75071 | -1.27079 | 1.65004 |
| hsa_circRNA_103136 | 8.01E-07 | 8.85634 | 9.94035 | 1.64804 |
| hsa_circRNA_101102 | 7.44E-06 | 6.91676 | 6.02031 | 1.64779 |
| hsa_circRNA_102927 | 4.02E-04 | 4.48538 | 0.45705 | 1.64705 |
| hsa_circRNA_100454 | 1.56E-06 | 8.13008 | 8.53447 | 1.64572 |
| hsa_circRNA_102364 | 1.11E-03 | 3.97338 | -0.75075 | 1.64556 |
| hsa_circRNA_103546 | 6.15E-04 | 4.26862 | -0.0555 | 1.64549 |
| hsa_circRNA_103014 | 2.04E-05 | 6.21266 | 4.46998 | 1.64523 |
| hsa_circRNA_102592 | 2.75E-03 | 3.5382 | -1.76162 | 1.64412 |
| hsa_circRNA_104967 | 1.94E-04 | 4.86881 | 1.36354 | 1.64204 |
| hsa_circRNA_001950 | 1.21E-03 | 3.93113 | -0.8498 | 1.64142 |
| hsa_circRNA_102613 | 7.07E-07 | 8.98041 | 10.17318 | 1.63997 |
| hsa_circRNA_102414 | 1.18E-03 | 3.94605 | -0.81484 | 1.63954 |
| hsa_circRNA_001302 | 3.72E-03 | 3.39768 | -2.08231 | 1.63808 |
| hsa_circRNA_104876 | 2.59E-05 | 6.0532 | 4.11039 | 1.63757 |
| hsa_circRNA_100750 | 1.85E-03 | 3.72668 | -1.32658 | 1.63658 |
| hsa_circRNA_103469 | 7.55E-05 | 5.41057 | 2.63407 | 1.63596 |
| hsa_circRNA_100044 | 1.73E-04 | 4.93322 | 1.51543 | 1.63435 |
| hsa_circRNA_104705 | 1.24E-02 | 2.8367 | -3.31834 | 1.63024 |
| hsa_circRNA_102476 | 1.67E-03 | 3.7763 | -1.21128 | 1.6298 |
| hsa_circRNA_104867 | 5.01E-06 | 7.21281 | 6.65267 | 1.62913 |
| hsa_circRNA_103292 | 2.01E-05 | 6.22327 | 4.49381 | 1.62685 |
| hsa_circRNA_101469 | 5.59E-07 | 9.3114 | 10.78395 | 1.6243 |
| hsa_circRNA_101895 | 2.11E-03 | 3.66298 | -1.47417 | 1.62144 |
| hsa_circRNA_101900 | 1.76E-05 | 6.30557 | 4.67812 | 1.62077 |
| hsa_circRNA_101465 | 4.03E-06 | 7.37964 | 7.0037 | 1.61755 |
| hsa_circRNA_102219 | 3.20E-04 | 4.60549 | 0.74122 | 1.61747 |
| hsa_circRNA_101441 | 4.83E-04 | 4.38584 | 0.22158 | 1.61546 |
| hsa_circRNA_101644 | 2.36E-05 | 6.11003 | 4.23889 | 1.61438 |
| hsa_circRNA_103102 | 1.94E-06 | 7.97549 | 8.22563 | 1.61288 |
| hsa_circRNA_104824 | 6.41E-06 | 7.01043 | 6.22168 | 1.61107 |
| hsa_circRNA_103408 | 8.04E-07 | 8.80068 | 9.83522 | 1.60721 |
| hsa_circRNA_400024 | 3.78E-03 | 3.38917 | -2.10162 | 1.60499 |
| hsa_circRNA_102323 | 2.35E-03 | 3.61121 | -1.5937 | 1.60497 |
| hsa_circRNA_101087 | 3.09E-06 | 7.55753 | 7.37374 | 1.60278 |
| hsa_circRNA_000300 | 1.48E-05 | 6.43707 | 4.97092 | 1.60134 |
| hsa_circRNA_101725 | 2.08E-03 | 3.67149 | -1.45449 | 1.6009 |
| hsa_circRNA_104090 | 1.78E-04 | 4.91733 | 1.47796 | 1.60047 |
| hsa_circRNA_000410 | 1.56E-03 | 3.80974 | -1.13341 | 1.59914 |
| hsa_circRNA_102291 | 2.11E-05 | 6.18791 | 4.41437 | 1.59821 |
| hsa_circRNA_104467 | 2.53E-06 | 7.75025 | 7.76961 | 1.59778 |
| hsa_circRNA_103531 | 3.06E-06 | 7.57215 | 7.40396 | 1.59741 |
| hsa_circRNA_001826 | 1.02E-03 | 4.01717 | -0.64798 | 1.59693 |
| hsa_circRNA_101017 | 4.44E-07 | 10.17997 | 12.31705 | 1.59671 |
| hsa_circRNA_101262 | 1.27E-04 | 5.10407 | 1.91741 | 1.59613 |
| hsa_circRNA_100570 | 5.02E-06 | 7.2004 | 6.62642 | 1.59535 |
| hsa_circRNA_400012 | 5.93E-03 | 3.18147 | -2.56813 | 1.59438 |
| hsa_circRNA_100016 | 1.09E-04 | 5.19392 | 2.12816 | 1.59335 |
| hsa_circRNA_104353 | 1.22E-05 | 6.56883 | 5.26214 | 1.59182 |
| hsa_circRNA_104661 | 6.13E-05 | 5.52828 | 2.90741 | 1.59015 |
| hsa_circRNA_103775 | 5.37E-05 | 5.60836 | 3.09268 | 1.58942 |
| hsa_circRNA_100449 | 2.82E-06 | 7.64174 | 7.54736 | 1.58929 |
| hsa_circRNA_400092 | 1.94E-04 | 4.86969 | 1.3656 | 1.58862 |
| hsa_circRNA_104282 | 2.56E-06 | 7.73341 | 7.73523 | 1.58834 |
| hsa_circRNA_102179 | 5.50E-05 | 5.59391 | 3.05931 | 1.58471 |
| hsa_circRNA_105026 | 1.46E-05 | 6.44637 | 4.99155 | 1.58433 |
| hsa_circRNA_104707 | 1.55E-02 | 2.73252 | -3.53775 | 1.58407 |
| hsa_circRNA_103073 | 1.18E-06 | 8.49113 | 9.24264 | 1.58388 |
| hsa_circRNA_101901 | 2.26E-05 | 6.13483 | 4.29484 | 1.58205 |
| hsa_circRNA_103619 | 2.67E-03 | 3.55224 | -1.72939 | 1.58179 |
| hsa_circRNA_101174 | 4.44E-07 | 10.55657 | 12.95151 | 1.58114 |
| hsa_circRNA_400034 | 1.78E-04 | 4.91863 | 1.48104 | 1.58089 |
| hsa_circRNA_102325 | 4.32E-06 | 7.32366 | 6.88634 | 1.58023 |
| hsa_circRNA_103871 | 2.58E-03 | 3.56779 | -1.69367 | 1.57936 |
| hsa_circRNA_103246 | 3.36E-04 | 4.58062 | 0.68239 | 1.57925 |
| hsa_circRNA_102763 | 7.94E-05 | 5.38165 | 2.56674 | 1.57888 |
| hsa_circRNA_104615 | 6.05E-04 | 4.27587 | -0.03836 | 1.57796 |
| hsa_circRNA_103563 | 4.02E-04 | 4.48515 | 0.45651 | 1.57747 |
| hsa_circRNA_102580 | 6.57E-05 | 5.49024 | 2.8192 | 1.57741 |
| hsa_circRNA_104828 | 1.11E-04 | 5.18324 | 2.10313 | 1.57694 |
| hsa_circRNA_102328 | 1.04E-04 | 5.22122 | 2.19208 | 1.57679 |
| hsa_circRNA_103242 | 1.70E-06 | 8.07653 | 8.42788 | 1.57538 |
| hsa_circRNA_104128 | 1.17E-04 | 5.15555 | 2.03821 | 1.57505 |
| hsa_circRNA_102239 | 2.98E-04 | 4.64586 | 0.83672 | 1.57485 |
| hsa_circRNA_104756 | 1.77E-03 | 3.74772 | -1.27773 | 1.57373 |
| hsa_circRNA_103504 | 5.00E-04 | 4.36852 | 0.18062 | 1.57356 |
| hsa_circRNA_100083 | 2.58E-04 | 4.71869 | 1.00895 | 1.57306 |
| hsa_circRNA_100031 | 1.65E-04 | 4.95867 | 1.5754 | 1.5727 |
| hsa_circRNA_102829 | 1.62E-04 | 4.96933 | 1.60051 | 1.57118 |
| hsa_circRNA_001805 | 2.36E-05 | 6.10924 | 4.2371 | 1.56908 |
| hsa_circRNA_104111 | 1.18E-05 | 6.60202 | 5.33514 | 1.56855 |
| hsa_circRNA_100112 | 4.37E-05 | 5.7318 | 3.37712 | 1.56799 |
| hsa_circRNA_105055 | 3.00E-03 | 3.49711 | -1.85576 | 1.56731 |
| hsa_circRNA_101694 | 1.31E-05 | 6.52524 | 5.16604 | 1.56702 |
| hsa_circRNA_100405 | 1.25E-03 | 3.91419 | -0.88944 | 1.56622 |
| hsa_circRNA_100087 | 4.71E-04 | 4.39964 | 0.2542 | 1.56599 |
| hsa_circRNA_103513 | 3.61E-05 | 5.85128 | 3.65098 | 1.56554 |
| hsa_circRNA_102827 | 5.27E-06 | 7.15157 | 6.52286 | 1.56487 |
| hsa_circRNA_100691 | 5.83E-06 | 7.07671 | 6.36346 | 1.56404 |
| hsa_circRNA_104532 | 7.94E-03 | 3.04657 | -2.86568 | 1.56389 |
| hsa_circRNA_104307 | 6.08E-05 | 5.53483 | 2.92259 | 1.56285 |
| hsa_circRNA_101722 | 1.19E-03 | 3.93982 | -0.82944 | 1.56177 |
| hsa_circRNA_102805 | 8.07E-05 | 5.36873 | 2.53665 | 1.56128 |
| hsa_circRNA_104679 | 1.07E-06 | 8.56897 | 9.39289 | 1.56091 |
| hsa_circRNA_103358 | 9.23E-06 | 6.77152 | 5.70576 | 1.56091 |
| hsa_circRNA_100499 | 1.71E-03 | 3.76589 | -1.23549 | 1.5595 |
| hsa_circRNA_101288 | 2.09E-05 | 6.19351 | 4.42695 | 1.55843 |
| hsa_circRNA_104665 | 8.82E-06 | 6.80255 | 5.77321 | 1.55764 |
| hsa_circRNA_101995 | 1.18E-06 | 8.47897 | 9.21907 | 1.5561 |
| hsa_circRNA_102329 | 1.42E-04 | 5.04392 | 1.77606 | 1.55524 |
| hsa_circRNA_101085 | 4.86E-05 | 5.66574 | 3.22507 | 1.55437 |
| hsa_circRNA_103586 | 1.30E-04 | 5.09374 | 1.89315 | 1.55383 |
| hsa_circRNA_104283 | 1.73E-04 | 4.93382 | 1.51684 | 1.55368 |
| hsa_circRNA_100012 | 3.48E-04 | 4.56221 | 0.63884 | 1.55326 |
| hsa_circRNA_100944 | 4.26E-04 | 4.45325 | 0.38103 | 1.55258 |
| hsa_circRNA_101160 | 2.93E-06 | 7.6063 | 7.47443 | 1.55246 |
| hsa_circRNA_102844 | 3.94E-03 | 3.37087 | -2.14309 | 1.55167 |
| hsa_circRNA_000552 | 3.39E-06 | 7.4905 | 7.23484 | 1.55089 |
| hsa_circRNA_102477 | 4.79E-06 | 7.23748 | 6.70484 | 1.55017 |
| hsa_circRNA_102096 | 5.19E-06 | 7.17339 | 6.56916 | 1.55003 |
| hsa_circRNA_101224 | 5.46E-06 | 7.12349 | 6.46315 | 1.54988 |
| hsa_circRNA_100052 | 5.15E-06 | 7.1806 | 6.58446 | 1.54818 |
| hsa_circRNA_104300 | 3.76E-05 | 5.82395 | 3.58847 | 1.54724 |
| hsa_circRNA_104642 | 3.58E-03 | 3.41566 | -2.04147 | 1.54665 |
| hsa_circRNA_103276 | 1.44E-05 | 6.45561 | 5.01204 | 1.54601 |
| hsa_circRNA_100909 | 7.26E-04 | 4.18083 | -0.26271 | 1.54594 |
| hsa_circRNA_103475 | 6.58E-04 | 4.23214 | -0.14165 | 1.54584 |
| hsa_circRNA_103401 | 2.74E-04 | 4.68757 | 0.93538 | 1.54536 |
| hsa_circRNA_100001 | 3.76E-04 | 4.52262 | 0.54517 | 1.54472 |
| hsa_circRNA_102720 | 3.67E-05 | 5.84026 | 3.62579 | 1.54451 |
| hsa_circRNA_104634 | 2.12E-05 | 6.17836 | 4.39288 | 1.54361 |
| hsa_circRNA_100830 | 1.57E-05 | 6.39684 | 4.88156 | 1.54312 |
| hsa_circRNA_102885 | 3.42E-03 | 3.43705 | -1.99282 | 1.54312 |
| hsa_circRNA_101509 | 7.58E-06 | 6.90367 | 5.99209 | 1.54231 |
| hsa_circRNA_101622 | 4.44E-07 | 10.39633 | 12.68374 | 1.54141 |
| hsa_circRNA_101701 | 1.76E-04 | 4.92415 | 1.49406 | 1.54138 |
| hsa_circRNA_102448 | 3.60E-04 | 4.54445 | 0.59681 | 1.54124 |
| hsa_circRNA_002048 | 3.25E-05 | 5.91062 | 3.78645 | 1.53943 |
| hsa_circRNA_103920 | 3.98E-05 | 5.78945 | 3.50943 | 1.53926 |
| hsa_circRNA_101758 | 1.88E-05 | 6.26558 | 4.58866 | 1.53792 |
| hsa_circRNA_100637 | 4.15E-04 | 4.46568 | 0.41044 | 1.53665 |
| hsa_circRNA_102809 | 1.43E-06 | 8.26378 | 8.79885 | 1.53662 |
| hsa_circRNA_101862 | 7.52E-04 | 4.16189 | -0.30737 | 1.53561 |
| hsa_circRNA_102988 | 2.08E-05 | 6.19506 | 4.43043 | 1.53519 |
| hsa_circRNA_102413 | 1.81E-04 | 4.90722 | 1.45413 | 1.53285 |
| hsa_circRNA_102259 | 1.52E-05 | 6.41801 | 4.92861 | 1.53191 |
| hsa_circRNA_101989 | 9.23E-06 | 6.77185 | 5.70647 | 1.53159 |
| hsa_circRNA_102928 | 4.77E-04 | 4.39276 | 0.23795 | 1.53153 |
| hsa_circRNA_002042 | 4.50E-04 | 4.42375 | 0.31125 | 1.5304 |
| hsa_circRNA_400085 | 1.89E-05 | 6.26388 | 4.58487 | 1.52995 |
| hsa_circRNA_103433 | 9.95E-04 | 4.02761 | -0.62344 | 1.52989 |
| hsa_circRNA_100131 | 1.12E-05 | 6.63629 | 5.41037 | 1.52922 |
| hsa_circRNA_104266 | 6.28E-05 | 5.51506 | 2.87677 | 1.52841 |
| hsa_circRNA_100893 | 3.79E-05 | 5.81999 | 3.57939 | 1.52758 |
| hsa_circRNA_101263 | 8.04E-07 | 8.81073 | 9.85423 | 1.52716 |
| hsa_circRNA_102652 | 3.09E-06 | 7.55897 | 7.37673 | 1.52692 |
| hsa_circRNA_100537 | 1.10E-04 | 5.19234 | 2.12446 | 1.52621 |
| hsa_circRNA_103511 | 6.07E-05 | 5.53665 | 2.92681 | 1.52609 |
| hsa_circRNA_103551 | 3.27E-05 | 5.90785 | 3.78014 | 1.52526 |
| hsa_circRNA_104932 | 1.99E-05 | 6.22941 | 4.50758 | 1.5243 |
| hsa_circRNA_101099 | 2.15E-04 | 4.81222 | 1.22995 | 1.52376 |
| hsa_circRNA_101951 | 4.44E-07 | 10.21694 | 12.38013 | 1.5229 |
| hsa_circRNA_100552 | 2.81E-05 | 6.00591 | 4.0032 | 1.52249 |
| hsa_circRNA_102209 | 4.11E-03 | 3.35104 | -2.18795 | 1.52182 |
| hsa_circRNA_104651 | 5.25E-05 | 5.62308 | 3.12669 | 1.52124 |
| hsa_circRNA_101755 | 2.94E-05 | 5.97589 | 3.93503 | 1.52103 |
| hsa_circRNA_103704 | 1.86E-03 | 3.72386 | -1.33314 | 1.52083 |
| hsa_circRNA_102589 | 3.11E-05 | 5.94281 | 3.85978 | 1.51941 |
| hsa_circRNA_102746 | 2.61E-04 | 4.71223 | 0.99367 | 1.51927 |
| hsa_circRNA_101911 | 1.47E-05 | 6.44219 | 4.98227 | 1.51926 |
| hsa_circRNA_101981 | 2.46E-04 | 4.74424 | 1.06935 | 1.51914 |
| hsa_circRNA_105053 | 8.43E-06 | 6.83596 | 5.84567 | 1.51889 |
| hsa_circRNA_104232 | 2.43E-04 | 4.75077 | 1.08478 | 1.51771 |
| hsa_circRNA_400102 | 2.65E-04 | 4.70364 | 0.97338 | 1.51761 |
| hsa_circRNA_100646 | 2.24E-02 | 2.55537 | -3.90164 | 1.51754 |
| hsa_circRNA_104911 | 4.76E-05 | 5.68148 | 3.26135 | 1.51749 |
| hsa_circRNA_103106 | 2.95E-05 | 5.97218 | 3.92659 | 1.51748 |
| hsa_circRNA_102050 | 3.16E-05 | 5.93254 | 3.83639 | 1.5169 |
| hsa_circRNA_100626 | 1.32E-05 | 6.50941 | 5.13108 | 1.51678 |
| hsa_circRNA_101471 | 6.10E-05 | 5.53201 | 2.91605 | 1.51658 |
| hsa_circRNA_103443 | 2.23E-05 | 6.14322 | 4.31376 | 1.51495 |
| hsa_circRNA_103512 | 2.85E-05 | 5.99541 | 3.97937 | 1.51215 |
| hsa_circRNA_101727 | 4.72E-04 | 4.39815 | 0.25068 | 1.51195 |
| hsa_circRNA_102440 | 1.53E-05 | 6.41335 | 4.91827 | 1.51169 |
| hsa_circRNA_104156 | 4.14E-05 | 5.76741 | 3.45888 | 1.51046 |
| hsa_circRNA_104799 | 1.72E-05 | 6.3292 | 4.73089 | 1.51023 |
| hsa_circRNA_102261 | 9.53E-06 | 6.74845 | 5.65553 | 1.51001 |
| hsa_circRNA_100157 | 7.45E-07 | 8.92288 | 10.06548 | 1.50818 |
| hsa_circRNA_103316 | 5.89E-03 | 3.18428 | -2.56189 | 1.50805 |
| hsa_circRNA_102539 | 8.88E-06 | 6.79825 | 5.76385 | 1.50749 |
| hsa_circRNA_104207 | 1.11E-05 | 6.64509 | 5.42967 | 1.50723 |
| hsa_circRNA_103454 | 1.24E-04 | 5.11995 | 1.95468 | 1.50671 |
| hsa_circRNA_101905 | 9.62E-04 | 4.04362 | -0.58582 | 1.50583 |
| hsa_circRNA_100370 | 8.04E-06 | 6.86673 | 5.91228 | 1.50561 |
| hsa_circRNA_100892 | 5.73E-04 | 4.30293 | 0.02557 | 1.50522 |
| hsa_circRNA_101897 | 6.42E-07 | 9.14468 | 10.47817 | 1.50492 |
| hsa_circRNA_103271 | 1.53E-03 | 3.81928 | -1.11117 | 1.50397 |
| hsa_circRNA_101083 | 2.90E-04 | 4.66004 | 0.87026 | 1.50386 |
| hsa_circRNA_400097 | 2.69E-04 | 4.69627 | 0.95594 | 1.50285 |
| hsa_circRNA_104667 | 1.60E-05 | 6.37919 | 4.84232 | 1.50225 |
| hsa_circRNA_102916 | 2.31E-05 | 6.12309 | 4.26835 | 1.5014 |
| hsa_circRNA_101906 | 1.54E-03 | 3.81574 | -1.11944 | 1.50079 |
| hsa_circRNA_103103 | 8.93E-05 | 5.30886 | 2.397 | 1.50042 |
| hsa_circRNA_100855 | 2.64E-06 | 7.70079 | 7.66851 | 1.49995 |
| hsa_circRNA_101904 | 2.29E-03 | 3.62352 | -1.56533 | 1.49992 |
| hsa_circRNA_103165 | 5.93E-04 | 4.28723 | -0.01152 | 1.49977 |
| hsa_circRNA_000872 | 1.50E-03 | 3.8281 | -1.09061 | 1.49968 |
| hsa_circRNA_102054 | 6.12E-06 | 7.04503 | 6.29576 | 1.49959 |
| hsa_circRNA_400037 | 2.06E-05 | 6.20572 | 4.4544 | 1.49904 |
| hsa_circRNA_104706 | 2.32E-02 | 2.53973 | -3.93317 | 1.49896 |
| hsa_circRNA_103018 | 6.91E-04 | 4.20589 | -0.20359 | 1.49813 |
| hsa_circRNA_104754 | 2.41E-04 | 4.75543 | 1.09579 | 1.49602 |
| hsa_circRNA_103564 | 2.95E-05 | 5.97242 | 3.92714 | 1.49585 |
| hsa_circRNA_102238 | 4.08E-04 | 4.47584 | 0.43446 | 1.4952 |
| hsa_circRNA_103251 | 4.28E-06 | 7.33384 | 6.90772 | 1.4942 |
| hsa_circRNA_103311 | 5.41E-04 | 4.33086 | 0.09158 | 1.4889 |
| hsa_circRNA_103978 | 3.23E-05 | 5.91552 | 3.79762 | 1.48805 |
| hsa_circRNA_100791 | 5.59E-05 | 5.58364 | 3.03555 | 1.48668 |
| hsa_circRNA_104931 | 1.20E-04 | 5.14407 | 2.0113 | 1.48651 |
| hsa_circRNA_102226 | 1.55E-03 | 3.81146 | -1.12941 | 1.48519 |
| hsa_circRNA_100090 | 2.51E-03 | 3.58047 | -1.6645 | 1.48412 |
| hsa_circRNA_104396 | 7.34E-05 | 5.429 | 2.67694 | 1.48272 |
| hsa_circRNA_100986 | 1.19E-05 | 6.59413 | 5.31781 | 1.48202 |
| hsa_circRNA_104671 | 1.11E-02 | 2.88949 | -3.20575 | 1.48029 |
| hsa_circRNA_103238 | 2.48E-06 | 7.7627 | 7.79501 | 1.47816 |
| hsa_circRNA_101011 | 1.92E-06 | 7.9821 | 8.23891 | 1.4778 |
| hsa_circRNA_105023 | 7.07E-05 | 5.44908 | 2.72363 | 1.47707 |
| hsa_circRNA_104973 | 3.78E-04 | 4.51912 | 0.53689 | 1.47703 |
| hsa_circRNA_101666 | 3.65E-05 | 5.84414 | 3.63465 | 1.47567 |
| hsa_circRNA_102570 | 3.73E-06 | 7.4328 | 7.11476 | 1.4713 |
| hsa_circRNA_104545 | 1.84E-05 | 6.28096 | 4.62309 | 1.47064 |
| hsa_circRNA_100068 | 3.71E-05 | 5.83174 | 3.60628 | 1.4705 |
| hsa_circRNA_100382 | 5.88E-07 | 9.24698 | 10.66624 | 1.47005 |
| hsa_circRNA_101560 | 7.42E-06 | 6.91913 | 6.02544 | 1.46966 |
| hsa_circRNA_102939 | 1.62E-04 | 4.96948 | 1.60087 | 1.46913 |
| hsa_circRNA_102373 | 1.32E-06 | 8.34085 | 8.95009 | 1.46907 |
| hsa_circRNA_100122 | 3.60E-05 | 5.85239 | 3.65352 | 1.46834 |
| hsa_circRNA_103492 | 2.62E-05 | 6.04782 | 4.09823 | 1.46785 |
| hsa_circRNA_103343 | 1.61E-04 | 4.97477 | 1.61333 | 1.4672 |
| hsa_circRNA_103973 | 1.18E-05 | 6.59596 | 5.32181 | 1.46716 |
| hsa_circRNA_102350 | 2.98E-06 | 7.58708 | 7.43479 | 1.46565 |
| hsa_circRNA_000936 | 8.69E-07 | 8.71657 | 9.67552 | 1.46315 |
| hsa_circRNA_101947 | 9.49E-05 | 5.2741 | 2.31579 | 1.46143 |
| hsa_circRNA_101669 | 5.27E-06 | 7.15103 | 6.5217 | 1.46136 |
| hsa_circRNA_103997 | 1.56E-06 | 8.1282 | 8.53074 | 1.46064 |
| hsa_circRNA_102137 | 2.17E-03 | 3.65033 | -1.50341 | 1.46004 |
| hsa_circRNA_101415 | 4.60E-07 | 9.94175 | 11.90642 | 1.45961 |
| hsa_circRNA_101219 | 4.16E-05 | 5.76358 | 3.45009 | 1.45929 |
| hsa_circRNA_102594 | 2.10E-06 | 7.90192 | 8.07748 | 1.45855 |
| hsa_circRNA_104567 | 9.21E-06 | 6.77586 | 5.71519 | 1.45822 |
| hsa_circRNA_104709 | 1.45E-02 | 2.76453 | -3.47074 | 1.45813 |
| hsa_circRNA_102153 | 6.52E-04 | 4.23609 | -0.13232 | 1.45744 |
| hsa_circRNA_101463 | 2.50E-03 | 3.58373 | -1.65699 | 1.45693 |
| hsa_circRNA_101954 | 8.72E-06 | 6.81335 | 5.79663 | 1.45553 |
| hsa_circRNA_101312 | 2.88E-05 | 5.98722 | 3.96077 | 1.45499 |
| hsa_circRNA_103392 | 4.87E-07 | 9.63058 | 11.3589 | 1.45321 |
| hsa_circRNA_104818 | 9.74E-06 | 6.73455 | 5.62525 | 1.4525 |
| hsa_circRNA_100127 | 1.94E-03 | 3.70266 | -1.38231 | 1.45232 |
| hsa_circRNA_102478 | 6.08E-05 | 5.53485 | 2.92264 | 1.45193 |
| hsa_circRNA_102574 | 4.86E-05 | 5.66712 | 3.22826 | 1.45155 |
| hsa_circRNA_103452 | 8.04E-07 | 8.80569 | 9.8447 | 1.45145 |
| hsa_circRNA_104031 | 8.41E-05 | 5.34292 | 2.47648 | 1.45083 |
| hsa_circRNA_103138 | 1.37E-05 | 6.49132 | 5.09108 | 1.44977 |
| hsa_circRNA_105052 | 5.41E-06 | 7.12882 | 6.47449 | 1.44969 |
| hsa_circRNA_102339 | 2.37E-05 | 6.10565 | 4.229 | 1.44957 |
| hsa_circRNA_100414 | 2.48E-05 | 6.07869 | 4.16808 | 1.44742 |
| hsa_circRNA_101795 | 4.00E-06 | 7.386 | 7.01702 | 1.44736 |
| hsa_circRNA_102401 | 2.72E-06 | 7.67902 | 7.6239 | 1.44618 |
| hsa_circRNA_102357 | 4.21E-05 | 5.75737 | 3.43584 | 1.44523 |
| hsa_circRNA_102467 | 2.98E-06 | 7.58928 | 7.43933 | 1.44474 |
| hsa_circRNA_102268 | 3.12E-05 | 5.93938 | 3.85197 | 1.44374 |
| hsa_circRNA_103045 | 2.49E-03 | 3.58561 | -1.65268 | 1.4428 |
| hsa_circRNA_104311 | 6.17E-04 | 4.26614 | -0.06134 | 1.44278 |
| hsa_circRNA_103240 | 1.72E-05 | 6.32504 | 4.72161 | 1.44173 |
| hsa_circRNA_100404 | 3.05E-05 | 5.95349 | 3.88408 | 1.4404 |
| hsa_circRNA_101887 | 4.48E-05 | 5.71901 | 3.3477 | 1.44027 |
| hsa_circRNA_100034 | 1.07E-04 | 5.20783 | 2.16074 | 1.44003 |
| hsa_circRNA_102463 | 3.08E-06 | 7.56472 | 7.3886 | 1.4396 |
| hsa_circRNA_103804 | 4.62E-05 | 5.70047 | 3.30506 | 1.4394 |
| hsa_circRNA_104723 | 2.10E-06 | 7.90304 | 8.07974 | 1.43937 |
| hsa_circRNA_102196 | 1.75E-06 | 8.04416 | 8.36323 | 1.43813 |
| hsa_circRNA_101634 | 1.93E-05 | 6.24713 | 4.54732 | 1.43751 |
| hsa_circRNA_100488 | 2.05E-05 | 6.20935 | 4.46256 | 1.43671 |
| hsa_circRNA_102128 | 2.19E-05 | 6.15425 | 4.33862 | 1.43505 |
| hsa_circRNA_104341 | 1.32E-05 | 6.50916 | 5.13052 | 1.43492 |
| hsa_circRNA_104198 | 5.19E-06 | 7.16697 | 6.55555 | 1.43481 |
| hsa_circRNA_101296 | 1.57E-05 | 6.39756 | 4.88318 | 1.43477 |
| hsa_circRNA_104209 | 4.70E-05 | 5.68916 | 3.27902 | 1.43451 |
| hsa_circRNA_100075 | 3.39E-06 | 7.48911 | 7.23195 | 1.43394 |
| hsa_circRNA_101874 | 4.41E-04 | 4.43446 | 0.33659 | 1.43376 |
| hsa_circRNA_100371 | 8.74E-06 | 6.81073 | 5.79097 | 1.43227 |
| hsa_circRNA_100734 | 1.64E-05 | 6.36089 | 4.80155 | 1.42821 |
| hsa_circRNA_103461 | 1.98E-04 | 4.85607 | 1.33346 | 1.42817 |
| hsa_circRNA_100916 | 6.29E-04 | 4.25504 | -0.08756 | 1.42814 |
| hsa_circRNA_101972 | 4.26E-04 | 4.45378 | 0.38228 | 1.42795 |
| hsa_circRNA_101103 | 3.21E-05 | 5.92056 | 3.8091 | 1.42714 |
| hsa_circRNA_100207 | 3.77E-04 | 4.52065 | 0.54051 | 1.42697 |
| hsa_circRNA_103110 | 1.50E-03 | 3.83062 | -1.08473 | 1.42609 |
| hsa_circRNA_104851 | 3.11E-05 | 5.9417 | 3.85726 | 1.42599 |
| hsa_circRNA_100740 | 3.04E-05 | 5.9549 | 3.8873 | 1.4242 |
| hsa_circRNA_102855 | 3.88E-04 | 4.5057 | 0.50512 | 1.42412 |
| hsa_circRNA_100529 | 1.56E-06 | 8.13457 | 8.54338 | 1.4236 |
| hsa_circRNA_104962 | 4.18E-04 | 4.46263 | 0.40321 | 1.42327 |
| hsa_circRNA_000645 | 3.97E-04 | 4.49373 | 0.4768 | 1.4223 |
| hsa_circRNA_100848 | 6.65E-05 | 5.48273 | 2.80178 | 1.42169 |
| hsa_circRNA_104822 | 2.12E-05 | 6.18184 | 4.40072 | 1.42109 |
| hsa_circRNA_101554 | 3.93E-03 | 3.37134 | -2.14202 | 1.42104 |
| hsa_circRNA_102870 | 3.33E-05 | 5.89529 | 3.75148 | 1.42091 |
| hsa_circRNA_100175 | 7.50E-05 | 5.41444 | 2.64307 | 1.42084 |
| hsa_circRNA_103267 | 2.28E-03 | 3.62628 | -1.55896 | 1.41844 |
| hsa_circRNA_100912 | 1.91E-03 | 3.71085 | -1.36332 | 1.41801 |
| hsa_circRNA_002111 | 1.42E-03 | 3.85548 | -1.02671 | 1.41723 |
| hsa_circRNA_104697 | 2.56E-04 | 4.72471 | 1.02318 | 1.41699 |
| hsa_circRNA_102160 | 1.18E-05 | 6.59855 | 5.32751 | 1.41648 |
| hsa_circRNA_102490 | 1.56E-06 | 8.12454 | 8.52346 | 1.41568 |
| hsa_circRNA_100180 | 8.64E-05 | 5.32616 | 2.43737 | 1.41521 |
| hsa_circRNA_100334 | 2.50E-03 | 3.58388 | -1.65665 | 1.41514 |
| hsa_circRNA_104632 | 2.01E-04 | 4.84755 | 1.31335 | 1.41329 |
| hsa_circRNA_102547 | 1.72E-04 | 4.93553 | 1.52086 | 1.41286 |
| hsa_circRNA_100478 | 2.31E-06 | 7.83795 | 7.94802 | 1.41232 |
| hsa_circRNA_103686 | 4.87E-07 | 9.84565 | 11.73867 | 1.41052 |
| hsa_circRNA_101241 | 2.63E-04 | 4.70886 | 0.98571 | 1.41034 |
| hsa_circRNA_104298 | 1.57E-05 | 6.39879 | 4.8859 | 1.4102 |
| hsa_circRNA_103470 | 2.10E-06 | 7.89405 | 8.06158 | 1.4091 |
| hsa_circRNA_104479 | 1.81E-03 | 3.73864 | -1.29883 | 1.40856 |
| hsa_circRNA_102431 | 4.33E-03 | 3.32502 | -2.24668 | 1.4072 |
| hsa_circRNA_102378 | 3.39E-04 | 4.57518 | 0.66952 | 1.40672 |
| hsa_circRNA_103012 | 3.24E-04 | 4.5996 | 0.72729 | 1.40657 |
| hsa_circRNA_101671 | 1.05E-05 | 6.678 | 5.50173 | 1.40649 |
| hsa_circRNA_104405 | 8.43E-06 | 6.83377 | 5.84093 | 1.40611 |
| hsa_circRNA_101400 | 6.36E-04 | 4.24966 | -0.10028 | 1.40562 |
| hsa_circRNA_103813 | 5.12E-06 | 7.18465 | 6.59304 | 1.40555 |
| hsa_circRNA_100450 | 2.87E-05 | 5.99007 | 3.96723 | 1.40406 |
| hsa_circRNA_103135 | 7.68E-05 | 5.39917 | 2.60755 | 1.40381 |
| hsa_circRNA_104404 | 4.63E-04 | 4.41099 | 0.28106 | 1.40373 |
| hsa_circRNA_100670 | 1.32E-06 | 8.33738 | 8.94331 | 1.40329 |
| hsa_circRNA_100684 | 3.97E-04 | 4.49299 | 0.47504 | 1.40261 |
| hsa_circRNA_101332 | 1.65E-05 | 6.35417 | 4.78659 | 1.40236 |
| hsa_circRNA_104297 | 4.29E-05 | 5.74286 | 3.40251 | 1.40213 |
| hsa_circRNA_100536 | 9.11E-05 | 5.2983 | 2.37233 | 1.40102 |
| hsa_circRNA_101606 | 3.73E-04 | 4.52637 | 0.55404 | 1.40061 |
| hsa_circRNA_102511 | 2.97E-06 | 7.59784 | 7.45698 | 1.40016 |
| hsa_circRNA_101462 | 2.23E-03 | 3.63596 | -1.53662 | 1.4001 |
| hsa_circRNA_102599 | 1.90E-03 | 3.71483 | -1.35409 | 1.3979 |
| hsa_circRNA_101550 | 1.14E-02 | 2.87573 | -3.23519 | 1.39735 |
| hsa_circRNA_100298 | 6.93E-04 | 4.20378 | -0.20857 | 1.39664 |
| hsa_circRNA_103939 | 3.67E-05 | 5.83791 | 3.62041 | 1.39645 |
| hsa_circRNA_103587 | 4.05E-04 | 4.47982 | 0.44388 | 1.39641 |
| hsa_circRNA_100732 | 1.02E-04 | 5.23215 | 2.21768 | 1.3964 |
| hsa_circRNA_100002 | 7.74E-06 | 6.88948 | 5.96146 | 1.39605 |
| hsa_circRNA_100787 | 7.46E-06 | 6.91395 | 6.01425 | 1.39604 |
| hsa_circRNA_102712 | 4.50E-05 | 5.71511 | 3.33873 | 1.39564 |
| hsa_circRNA_102456 | 1.11E-04 | 5.18315 | 2.10292 | 1.39421 |
| hsa_circRNA_102765 | 5.19E-06 | 7.17363 | 6.56968 | 1.39418 |
| hsa_circRNA_102526 | 1.26E-03 | 3.91188 | -0.89486 | 1.39393 |
| hsa_circRNA_100073 | 4.83E-04 | 4.38544 | 0.22062 | 1.39387 |
| hsa_circRNA_103494 | 3.82E-05 | 5.81596 | 3.57016 | 1.39306 |
| hsa_circRNA_100542 | 6.13E-05 | 5.52812 | 2.90705 | 1.39303 |
| hsa_circRNA_400089 | 8.65E-04 | 4.09482 | -0.46537 | 1.39302 |
| hsa_circRNA_103582 | 1.55E-04 | 4.99825 | 1.6686 | 1.39293 |
| hsa_circRNA_102313 | 2.54E-05 | 6.06681 | 4.1412 | 1.39283 |
| hsa_circRNA_104925 | 4.16E-06 | 7.35381 | 6.9496 | 1.39221 |
| hsa_circRNA_100831 | 3.75E-04 | 4.52432 | 0.54918 | 1.39202 |
| hsa_circRNA_101289 | 5.25E-05 | 5.62266 | 3.12571 | 1.39184 |
| hsa_circRNA_101785 | 9.66E-06 | 6.74046 | 5.63814 | 1.39071 |
| hsa_circRNA_000041 | 1.96E-05 | 6.2401 | 4.53157 | 1.39061 |
| hsa_circRNA_100630 | 1.21E-05 | 6.57701 | 5.28013 | 1.39022 |
| hsa_circRNA_100045 | 4.67E-04 | 4.40508 | 0.26707 | 1.39008 |
| hsa_circRNA_104593 | 1.08E-04 | 5.20392 | 2.15158 | 1.38911 |
| hsa_circRNA_101819 | 3.54E-06 | 7.46517 | 7.18218 | 1.38826 |
| hsa_circRNA_102784 | 1.63E-04 | 4.96609 | 1.59288 | 1.38717 |
| hsa_circRNA_104599 | 2.43E-06 | 7.80394 | 7.87896 | 1.387 |
| hsa_circRNA_104022 | 5.69E-05 | 5.57202 | 3.00869 | 1.3869 |
| hsa_circRNA_101015 | 1.05E-05 | 6.67649 | 5.49842 | 1.38603 |
| hsa_circRNA_104359 | 2.59E-06 | 7.72001 | 7.70784 | 1.38539 |
| hsa_circRNA_104527 | 2.66E-05 | 6.03578 | 4.07095 | 1.38531 |
| hsa_circRNA_101637 | 5.61E-04 | 4.31446 | 0.05281 | 1.38472 |
| hsa_circRNA_103653 | 1.06E-03 | 3.99657 | -0.69635 | 1.38466 |
| hsa_circRNA_102966 | 1.08E-06 | 8.56087 | 9.37731 | 1.38464 |
| hsa_circRNA_002039 | 4.72E-04 | 4.39816 | 0.25071 | 1.38391 |
| hsa_circRNA_400028 | 4.59E-06 | 7.27648 | 6.7871 | 1.38376 |
| hsa_circRNA_102517 | 1.69E-04 | 4.94745 | 1.54895 | 1.38362 |
| hsa_circRNA_102563 | 8.33E-05 | 5.34853 | 2.48956 | 1.38277 |
| hsa_circRNA_102486 | 4.42E-04 | 4.43295 | 0.333 | 1.38137 |
| hsa_circRNA_103993 | 1.61E-02 | 2.71451 | -3.57529 | 1.38113 |
| hsa_circRNA_103560 | 3.86E-04 | 4.50795 | 0.51045 | 1.38108 |
| hsa_circRNA_103312 | 7.34E-04 | 4.17473 | -0.27709 | 1.38065 |
| hsa_circRNA_101711 | 5.52E-05 | 5.59044 | 3.05128 | 1.37888 |
| hsa_circRNA_104477 | 3.04E-05 | 5.95714 | 3.89238 | 1.37858 |
| hsa_circRNA_101608 | 2.55E-04 | 4.7276 | 1.03001 | 1.37834 |
| hsa_circRNA_103522 | 6.67E-06 | 6.98442 | 6.16589 | 1.37767 |
| hsa_circRNA_100387 | 1.02E-04 | 5.23383 | 2.22161 | 1.37732 |
| hsa_circRNA_104252 | 7.72E-05 | 5.39619 | 2.6006 | 1.37724 |
| hsa_circRNA_104503 | 2.08E-03 | 3.67047 | -1.45685 | 1.37676 |
| hsa_circRNA_400072 | 2.25E-05 | 6.13865 | 4.30346 | 1.37648 |
| hsa_circRNA_102514 | 1.91E-05 | 6.25391 | 4.56253 | 1.37598 |
| hsa_circRNA_102890 | 1.63E-03 | 3.78746 | -1.1853 | 1.37455 |
| hsa_circRNA_103888 | 2.02E-03 | 3.68525 | -1.42264 | 1.37394 |
| hsa_circRNA_104398 | 5.47E-05 | 5.59804 | 3.06883 | 1.37392 |
| hsa_circRNA_101740 | 6.15E-03 | 3.16453 | -2.60574 | 1.3737 |
| hsa_circRNA_100252 | 4.71E-05 | 5.68756 | 3.27534 | 1.37361 |
| hsa_circRNA_102687 | 8.17E-05 | 5.36182 | 2.52053 | 1.37332 |
| hsa_circRNA_102813 | 8.96E-03 | 2.98953 | -2.99 | 1.37248 |
| hsa_circRNA_100789 | 5.52E-05 | 5.59134 | 3.05335 | 1.37194 |
| hsa_circRNA_103740 | 2.31E-03 | 3.61973 | -1.57407 | 1.37183 |
| hsa_circRNA_101920 | 1.32E-05 | 6.50836 | 5.12875 | 1.37117 |
| hsa_circRNA_101523 | 4.10E-04 | 4.47342 | 0.42874 | 1.37115 |
| hsa_circRNA_100038 | 3.55E-02 | 2.33155 | -4.34258 | 1.37031 |
| hsa_circRNA_100486 | 1.02E-05 | 6.69777 | 5.54495 | 1.37023 |
| hsa_circRNA_103837 | 4.82E-05 | 5.67245 | 3.24053 | 1.3701 |
| hsa_circRNA_100223 | 5.39E-03 | 3.22506 | -2.47102 | 1.36777 |
| hsa_circRNA_102361 | 3.92E-05 | 5.79737 | 3.52759 | 1.36475 |
| hsa_circRNA_103647 | 8.79E-03 | 2.99826 | -2.97104 | 1.36464 |
| hsa_circRNA_100624 | 6.41E-06 | 7.01183 | 6.22469 | 1.36362 |
| hsa_circRNA_104766 | 3.09E-03 | 3.4836 | -1.88663 | 1.36291 |
| hsa_circRNA_103241 | 1.75E-06 | 8.054 | 8.38289 | 1.36219 |
| hsa_circRNA_104573 | 1.13E-03 | 3.96599 | -0.76809 | 1.3602 |
| hsa_circRNA_103064 | 1.71E-05 | 6.33396 | 4.74151 | 1.36005 |
| hsa_circRNA_101147 | 3.33E-05 | 5.8941 | 3.74877 | 1.35938 |
| hsa_circRNA_102545 | 5.46E-05 | 5.59915 | 3.07142 | 1.35931 |
| hsa_circRNA_101176 | 1.72E-04 | 4.93707 | 1.52449 | 1.35878 |
| hsa_circRNA_102334 | 7.14E-04 | 4.18942 | -0.24244 | 1.35806 |
| hsa_circRNA_104586 | 2.33E-03 | 3.61644 | -1.58166 | 1.3574 |
| hsa_circRNA_102262 | 2.11E-05 | 6.18571 | 4.40942 | 1.35735 |
| hsa_circRNA_101596 | 7.24E-06 | 6.93525 | 6.06015 | 1.35633 |
| hsa_circRNA_100610 | 1.20E-04 | 5.14206 | 2.00659 | 1.35577 |
| hsa_circRNA_104346 | 1.15E-03 | 3.95745 | -0.78811 | 1.35465 |
| hsa_circRNA_100051 | 4.44E-07 | 10.09019 | 12.16314 | 1.35429 |
| hsa_circRNA_104959 | 4.05E-04 | 4.48035 | 0.44514 | 1.35342 |
| hsa_circRNA_101572 | 7.22E-05 | 5.43672 | 2.69489 | 1.35338 |
| hsa_circRNA_104419 | 2.45E-04 | 4.74729 | 1.07656 | 1.35333 |
| hsa_circRNA_104539 | 3.24E-05 | 5.91442 | 3.7951 | 1.35312 |
| hsa_circRNA_100993 | 2.70E-03 | 3.54593 | -1.74388 | 1.352 |
| hsa_circRNA_102069 | 1.18E-06 | 8.46831 | 9.19841 | 1.34984 |
| hsa_circRNA_103800 | 1.20E-04 | 5.14148 | 2.00521 | 1.34842 |
| hsa_circRNA_101437 | 1.76E-05 | 6.30735 | 4.68211 | 1.34786 |
| hsa_circRNA_103677 | 1.90E-05 | 6.26058 | 4.57746 | 1.34776 |
| hsa_circRNA_101928 | 1.00E-04 | 5.24137 | 2.23925 | 1.34725 |
| hsa_circRNA_100097 | 2.98E-02 | 2.41793 | -4.17509 | 1.34673 |
| hsa_circRNA_104781 | 1.23E-03 | 3.92533 | -0.86338 | 1.34666 |
| hsa_circRNA_104185 | 2.15E-04 | 4.81236 | 1.23027 | 1.34578 |
| hsa_circRNA_102043 | 6.70E-04 | 4.22147 | -0.16683 | 1.34532 |
| hsa_circRNA_100546 | 6.72E-04 | 4.21992 | -0.17047 | 1.34528 |
| hsa_circRNA_103968 | 1.33E-04 | 5.07974 | 1.86025 | 1.34458 |
| hsa_circRNA_103212 | 7.53E-06 | 6.90792 | 6.00126 | 1.34441 |
| hsa_circRNA_100629 | 7.07E-06 | 6.9502 | 6.09235 | 1.34354 |
| hsa_circRNA_103037 | 6.81E-05 | 5.46775 | 2.76701 | 1.34326 |
| hsa_circRNA_100237 | 8.79E-06 | 6.80601 | 5.78071 | 1.34271 |
| hsa_circRNA_101285 | 7.95E-06 | 6.87374 | 5.92744 | 1.34244 |
| hsa_circRNA_104882 | 2.95E-03 | 3.50532 | -1.83697 | 1.34203 |
| hsa_circRNA_102360 | 1.59E-04 | 4.98277 | 1.63216 | 1.34115 |
| hsa_circRNA_101688 | 2.81E-05 | 6.00708 | 4.00586 | 1.34082 |
| hsa_circRNA_102593 | 2.48E-06 | 7.76429 | 7.79825 | 1.34048 |
| hsa_circRNA_102742 | 1.96E-04 | 4.86295 | 1.34971 | 1.33947 |
| hsa_circRNA_103905 | 1.44E-06 | 8.25626 | 8.78405 | 1.33812 |
| hsa_circRNA_101078 | 2.03E-05 | 6.21683 | 4.47936 | 1.33804 |
| hsa_circRNA_102488 | 8.43E-06 | 6.83531 | 5.84425 | 1.3376 |
| hsa_circRNA_100407 | 3.40E-04 | 4.57388 | 0.66646 | 1.33583 |
| hsa_circRNA_102392 | 2.87E-05 | 5.99159 | 3.97068 | 1.33554 |
| hsa_circRNA_103365 | 4.42E-06 | 7.29852 | 6.83351 | 1.33446 |
| hsa_circRNA_101917 | 1.32E-05 | 6.50991 | 5.13218 | 1.33389 |
| hsa_circRNA_100351 | 2.03E-05 | 6.21616 | 4.47785 | 1.33333 |
| hsa_circRNA_102383 | 5.52E-05 | 5.59042 | 3.05124 | 1.33303 |
| hsa_circRNA_103681 | 1.68E-04 | 4.95064 | 1.55647 | 1.3327 |
| hsa_circRNA_101434 | 3.30E-04 | 4.59072 | 0.70629 | 1.33236 |
| hsa_circRNA_104141 | 1.43E-04 | 5.04154 | 1.77046 | 1.3323 |
| hsa_circRNA_100494 | 3.78E-06 | 7.42133 | 7.09082 | 1.33221 |
| hsa_circRNA_104869 | 9.07E-05 | 5.30082 | 2.37821 | 1.33196 |
| hsa_circRNA_101372 | 3.97E-04 | 4.49288 | 0.4748 | 1.33098 |
| hsa_circRNA_102065 | 3.91E-06 | 7.40069 | 7.04773 | 1.33087 |
| hsa_circRNA_102614 | 1.87E-05 | 6.27062 | 4.59994 | 1.33083 |
| hsa_circRNA_101272 | 3.59E-06 | 7.45414 | 7.15922 | 1.33053 |
| hsa_circRNA_102678 | 2.37E-02 | 2.52975 | -3.95324 | 1.3303 |
| hsa_circRNA_100185 | 4.12E-04 | 4.47093 | 0.42286 | 1.32904 |
| hsa_circRNA_101735 | 3.33E-06 | 7.50981 | 7.27492 | 1.32829 |
| hsa_circRNA_104067 | 9.98E-05 | 5.2457 | 2.24937 | 1.32794 |
| hsa_circRNA_103639 | 2.26E-03 | 3.63127 | -1.54744 | 1.32793 |
| hsa_circRNA_102767 | 8.17E-05 | 5.36091 | 2.51841 | 1.32778 |
| hsa_circRNA_102207 | 1.18E-06 | 8.48902 | 9.23855 | 1.32632 |
| hsa_circRNA_101787 | 2.76E-04 | 4.68459 | 0.92833 | 1.32581 |
| hsa_circRNA_103255 | 2.53E-05 | 6.0685 | 4.14502 | 1.3258 |
| hsa_circRNA_103307 | 8.32E-04 | 4.11374 | -0.42082 | 1.3258 |
| hsa_circRNA_103104 | 1.58E-05 | 6.3925 | 4.87192 | 1.32553 |
| hsa_circRNA_103015 | 1.47E-05 | 6.4434 | 4.98496 | 1.3254 |
| hsa_circRNA_100736 | 2.19E-06 | 7.86698 | 8.00684 | 1.32528 |
| hsa_circRNA_103574 | 8.56E-03 | 3.01203 | -2.94106 | 1.32496 |
| hsa_circRNA_102515 | 1.77E-06 | 8.02614 | 8.32719 | 1.32448 |
| hsa_circRNA_103833 | 9.42E-05 | 5.27768 | 2.32416 | 1.32396 |
| hsa_circRNA_102288 | 4.75E-06 | 7.25032 | 6.73193 | 1.32352 |
| hsa_circRNA_103706 | 1.72E-05 | 6.32546 | 4.72255 | 1.32289 |
| hsa_circRNA_103426 | 1.18E-06 | 8.46619 | 9.1943 | 1.32254 |
| hsa_circRNA_100491 | 3.22E-06 | 7.53163 | 7.32015 | 1.32209 |
| hsa_circRNA_100141 | 6.38E-03 | 3.14669 | -2.64528 | 1.32187 |
| hsa_circRNA_101988 | 2.19E-05 | 6.15777 | 4.34653 | 1.32112 |
| hsa_circRNA_100889 | 4.21E-04 | 4.45938 | 0.39553 | 1.32065 |
| hsa_circRNA_104192 | 5.02E-06 | 7.19895 | 6.62333 | 1.32041 |
| hsa_circRNA_101084 | 1.92E-04 | 4.874 | 1.37578 | 1.3199 |
| hsa_circRNA_103239 | 4.87E-07 | 9.58543 | 11.27839 | 1.31968 |
| hsa_circRNA_101754 | 1.25E-05 | 6.55346 | 5.22829 | 1.31935 |
| hsa_circRNA_100359 | 1.25E-04 | 5.11682 | 1.94735 | 1.31842 |
| hsa_circRNA_103222 | 2.80E-06 | 7.64723 | 7.55865 | 1.31819 |
| hsa_circRNA_100028 | 7.69E-05 | 5.39791 | 2.60461 | 1.31794 |
| hsa_circRNA_101661 | 2.66E-06 | 7.6921 | 7.65071 | 1.31783 |
| hsa_circRNA_102421 | 4.83E-05 | 5.67019 | 3.23534 | 1.31721 |
| hsa_circRNA_104804 | 1.23E-03 | 3.92557 | -0.86282 | 1.31681 |
| hsa_circRNA_101863 | 1.30E-04 | 5.09321 | 1.89189 | 1.31603 |
| hsa_circRNA_103565 | 9.62E-05 | 5.26398 | 2.29213 | 1.31601 |
| hsa_circRNA_102449 | 5.19E-06 | 7.17173 | 6.56564 | 1.31535 |
| hsa_circRNA_102059 | 5.11E-06 | 7.18714 | 6.59833 | 1.31477 |
| hsa_circRNA_101854 | 4.79E-05 | 5.6772 | 3.25149 | 1.31473 |
| hsa_circRNA_102974 | 5.93E-04 | 4.28677 | -0.01263 | 1.31424 |
| hsa_circRNA_102641 | 7.87E-04 | 4.13973 | -0.3596 | 1.31418 |
| hsa_circRNA_102604 | 1.56E-06 | 8.16311 | 8.60002 | 1.31379 |
| hsa_circRNA_103441 | 2.02E-05 | 6.22 | 4.48646 | 1.31359 |
| hsa_circRNA_102266 | 2.19E-05 | 6.1569 | 4.34459 | 1.31229 |
| hsa_circRNA_102271 | 2.82E-05 | 6.00341 | 3.99754 | 1.31126 |
| hsa_circRNA_101013 | 1.27E-04 | 5.10488 | 1.9193 | 1.31118 |
| hsa_circRNA_100551 | 1.26E-04 | 5.11007 | 1.9315 | 1.31023 |
| hsa_circRNA_102989 | 1.15E-05 | 6.61981 | 5.37421 | 1.30967 |
| hsa_circRNA_104415 | 2.19E-05 | 6.15517 | 4.34067 | 1.30911 |
| hsa_circRNA_100357 | 4.58E-03 | 3.30001 | -2.30301 | 1.30869 |
| hsa_circRNA_103318 | 1.99E-05 | 6.22933 | 4.5074 | 1.30865 |
| hsa_circRNA_001535 | 6.13E-05 | 5.52781 | 2.90632 | 1.30827 |
| hsa_circRNA_101516 | 1.21E-04 | 5.13586 | 1.99204 | 1.30825 |
| hsa_circRNA_103805 | 2.13E-05 | 6.17221 | 4.37906 | 1.30772 |
| hsa_circRNA_104017 | 3.71E-03 | 3.39902 | -2.07927 | 1.30714 |
| hsa_circRNA_103573 | 1.22E-03 | 3.92939 | -0.85387 | 1.30707 |
| hsa_circRNA_100821 | 8.53E-04 | 4.10165 | -0.4493 | 1.30553 |
| hsa_circRNA_100080 | 8.36E-04 | 4.11116 | -0.4269 | 1.30405 |
| hsa_circRNA_100872 | 1.31E-05 | 6.52618 | 5.1681 | 1.30398 |
| hsa_circRNA_100654 | 1.53E-04 | 5.00456 | 1.68346 | 1.30371 |
| hsa_circRNA_103160 | 4.81E-05 | 5.67522 | 3.24692 | 1.30303 |
| hsa_circRNA_101354 | 7.15E-04 | 4.1885 | -0.24462 | 1.3025 |
| hsa_circRNA_001416 | 2.56E-04 | 4.72436 | 1.02235 | 1.30214 |
| hsa_circRNA_103615 | 8.67E-07 | 8.73423 | 9.70914 | 1.30101 |
| hsa_circRNA_104896 | 1.83E-04 | 4.90257 | 1.44317 | 1.30072 |
| hsa_circRNA_103735 | 5.36E-04 | 4.33652 | 0.10495 | 1.30046 |
| hsa_circRNA_102002 | 1.56E-04 | 4.99671 | 1.66498 | 1.30022 |
| hsa_circRNA_103493 | 9.09E-05 | 5.29954 | 2.37522 | 1.29963 |
| hsa_circRNA_103344 | 1.28E-03 | 3.90499 | -0.91098 | 1.29847 |
| hsa_circRNA_001408 | 8.10E-05 | 5.36561 | 2.52936 | 1.29845 |
| hsa_circRNA_100972 | 1.19E-04 | 5.14677 | 2.01763 | 1.29801 |
| hsa_circRNA_100238 | 1.15E-05 | 6.62511 | 5.38585 | 1.29776 |
| hsa_circRNA_104469 | 1.32E-06 | 8.36261 | 8.99266 | 1.29743 |
| hsa_circRNA_100851 | 1.28E-04 | 5.10015 | 1.90821 | 1.2964 |
| hsa_circRNA_104592 | 1.54E-04 | 5.00178 | 1.67692 | 1.29572 |
| hsa_circRNA_102441 | 2.95E-03 | 3.50612 | -1.83514 | 1.29553 |
| hsa_circRNA_104729 | 3.69E-05 | 5.83453 | 3.61267 | 1.2952 |
| hsa_circRNA_102053 | 2.57E-04 | 4.72128 | 1.01508 | 1.29468 |
| hsa_circRNA_101247 | 4.05E-04 | 4.48062 | 0.44578 | 1.29424 |
| hsa_circRNA_103342 | 2.86E-03 | 3.5197 | -1.80403 | 1.29318 |
| hsa_circRNA_103376 | 4.09E-04 | 4.47493 | 0.43233 | 1.29282 |
| hsa_circRNA_104522 | 1.73E-04 | 4.93261 | 1.51399 | 1.29259 |
| hsa_circRNA_100862 | 1.49E-03 | 3.8313 | -1.08314 | 1.29251 |
| hsa_circRNA_102562 | 6.50E-03 | 3.13837 | -2.66369 | 1.2923 |
| hsa_circRNA_102423 | 1.66E-04 | 4.95638 | 1.57 | 1.29222 |
| hsa_circRNA_104602 | 2.65E-04 | 4.70409 | 0.97444 | 1.29119 |
| hsa_circRNA_104880 | 1.75E-06 | 8.04566 | 8.36624 | 1.29112 |
| hsa_circRNA_104251 | 5.43E-05 | 5.60238 | 3.07886 | 1.29066 |
| hsa_circRNA_104046 | 2.06E-05 | 6.20556 | 4.45405 | 1.29049 |
| hsa_circRNA_100352 | 2.50E-02 | 2.50308 | -4.00664 | 1.28915 |
| hsa_circRNA_101875 | 5.68E-06 | 7.09805 | 6.40897 | 1.28814 |
| hsa_circRNA_101234 | 3.74E-06 | 7.42823 | 7.10522 | 1.28807 |
| hsa_circRNA_102598 | 5.37E-05 | 5.60804 | 3.09194 | 1.28807 |
| hsa_circRNA_104850 | 1.98E-04 | 4.85541 | 1.33191 | 1.28799 |
| hsa_circRNA_100623 | 2.18E-03 | 3.6467 | -1.51179 | 1.28758 |
| hsa_circRNA_104108 | 1.13E-04 | 5.17003 | 2.07218 | 1.28743 |
| hsa_circRNA_101779 | 5.96E-04 | 4.28381 | -0.01962 | 1.28707 |
| hsa_circRNA_102764 | 1.73E-05 | 6.31704 | 4.70374 | 1.28657 |
| hsa_circRNA_100484 | 1.49E-05 | 6.43337 | 4.96272 | 1.28596 |
| hsa_circRNA_102510 | 5.79E-06 | 7.08234 | 6.37547 | 1.28584 |
| hsa_circRNA_102484 | 1.53E-06 | 8.19946 | 8.67198 | 1.28568 |
| hsa_circRNA_102097 | 5.00E-05 | 5.65222 | 3.19391 | 1.28539 |
| hsa_circRNA_100111 | 1.01E-05 | 6.70912 | 5.56975 | 1.28502 |
| hsa_circRNA_104515 | 3.19E-05 | 5.92531 | 3.81993 | 1.28489 |
| hsa_circRNA_100194 | 1.26E-05 | 6.54767 | 5.21551 | 1.28441 |
| hsa_circRNA_104235 | 2.81E-05 | 6.00519 | 4.00156 | 1.28218 |
| hsa_circRNA_100549 | 1.58E-05 | 6.39123 | 4.86909 | 1.2816 |
| hsa_circRNA_103036 | 1.96E-03 | 3.69957 | -1.38946 | 1.28143 |
| hsa_circRNA_102224 | 2.64E-03 | 3.55721 | -1.71798 | 1.28127 |
| hsa_circRNA_104138 | 6.47E-05 | 5.49838 | 2.8381 | 1.28094 |
| hsa_circRNA_102573 | 4.35E-06 | 7.31268 | 6.86328 | 1.27991 |
| hsa_circRNA_102267 | 3.89E-05 | 5.80224 | 3.53874 | 1.27971 |
| hsa_circRNA_100354 | 6.49E-05 | 5.49695 | 2.83478 | 1.27959 |
| hsa_circRNA_103810 | 9.34E-06 | 6.76304 | 5.6873 | 1.27934 |
| hsa_circRNA_103418 | 1.72E-05 | 6.32496 | 4.72143 | 1.27898 |
| hsa_circRNA_101894 | 8.43E-05 | 5.33986 | 2.46933 | 1.27645 |
| hsa_circRNA_400065 | 9.44E-06 | 6.75707 | 5.6743 | 1.27618 |
| hsa_circRNA_102073 | 5.67E-03 | 3.20305 | -2.52011 | 1.27613 |
| hsa_circRNA_103542 | 5.82E-06 | 7.07875 | 6.3678 | 1.276 |
| hsa_circRNA_103400 | 3.36E-06 | 7.50376 | 7.26236 | 1.27588 |
| hsa_circRNA_100638 | 7.55E-04 | 4.15931 | -0.31345 | 1.27568 |
| hsa_circRNA_102540 | 3.21E-05 | 5.91984 | 3.80747 | 1.27565 |
| hsa_circRNA_104974 | 7.01E-04 | 4.19811 | -0.22196 | 1.27494 |
| hsa_circRNA_100517 | 4.63E-03 | 3.29456 | -2.31528 | 1.27417 |
| hsa_circRNA_103265 | 5.39E-06 | 7.1333 | 6.48401 | 1.27396 |
| hsa_circRNA_001676 | 1.39E-03 | 3.86691 | -1.00002 | 1.2739 |
| hsa_circRNA_103042 | 6.19E-05 | 5.52333 | 2.89594 | 1.27306 |
| hsa_circRNA_100347 | 8.82E-04 | 4.08436 | -0.49001 | 1.27272 |
| hsa_circRNA_100861 | 1.83E-03 | 3.73315 | -1.31158 | 1.27262 |
| hsa_circRNA_103798 | 3.92E-05 | 5.79704 | 3.52683 | 1.27227 |
| hsa_circRNA_102012 | 2.48E-06 | 7.77464 | 7.81934 | 1.27089 |
| hsa_circRNA_101535 | 1.90E-04 | 4.87984 | 1.38954 | 1.26924 |
| hsa_circRNA_101851 | 1.02E-05 | 6.69829 | 5.54611 | 1.26904 |
| hsa_circRNA_103649 | 6.22E-04 | 4.26063 | -0.07438 | 1.26891 |
| hsa_circRNA_104083 | 1.05E-03 | 4.00259 | -0.68222 | 1.26832 |
| hsa_circRNA_400093 | 1.96E-04 | 4.86279 | 1.34932 | 1.26807 |
| hsa_circRNA_104255 | 1.78E-05 | 6.3007 | 4.66724 | 1.2679 |
| hsa_circRNA_103340 | 6.81E-04 | 4.21338 | -0.18591 | 1.26653 |
| hsa_circRNA_101450 | 2.48E-06 | 7.77957 | 7.82937 | 1.2655 |
| hsa_circRNA_101115 | 3.48E-03 | 3.4283 | -2.01273 | 1.26547 |
| hsa_circRNA_100035 | 2.29E-03 | 3.62404 | -1.56412 | 1.26543 |
| hsa_circRNA_104072 | 4.35E-05 | 5.735 | 3.38446 | 1.26508 |
| hsa_circRNA_102403 | 2.01E-05 | 6.22349 | 4.49429 | 1.26439 |
| hsa_circRNA_104210 | 1.25E-05 | 6.55514 | 5.23197 | 1.26352 |
| hsa_circRNA_104997 | 6.93E-04 | 4.20386 | -0.20838 | 1.26304 |
| hsa_circRNA_103039 | 9.41E-05 | 5.27863 | 2.32639 | 1.26273 |
| hsa_circRNA_101079 | 1.31E-05 | 6.52509 | 5.16569 | 1.26212 |
| hsa_circRNA_103273 | 2.50E-03 | 3.58369 | -1.65709 | 1.26198 |
| hsa_circRNA_104253 | 4.14E-05 | 5.76788 | 3.45996 | 1.26182 |
| hsa_circRNA_101605 | 9.31E-04 | 4.05791 | -0.55221 | 1.26049 |
| hsa_circRNA_103644 | 1.11E-04 | 5.17982 | 2.09512 | 1.26019 |
| hsa_circRNA_103081 | 2.47E-05 | 6.08336 | 4.17863 | 1.26007 |
| hsa_circRNA_103627 | 4.81E-03 | 3.27625 | -2.35642 | 1.25975 |
| hsa_circRNA_103689 | 1.45E-02 | 2.7647 | -3.47039 | 1.25964 |
| hsa_circRNA_102068 | 2.10E-06 | 7.91579 | 8.10546 | 1.25873 |
| hsa_circRNA_102290 | 3.34E-04 | 4.58351 | 0.68924 | 1.25828 |
| hsa_circRNA_100279 | 7.67E-06 | 6.89706 | 5.97782 | 1.25765 |
| hsa_circRNA_101001 | 5.67E-04 | 4.30907 | 0.04008 | 1.25757 |
| hsa_circRNA_102480 | 9.48E-06 | 6.75413 | 5.6679 | 1.25668 |
| hsa_circRNA_103065 | 5.84E-03 | 3.18867 | -2.55211 | 1.256 |
| hsa_circRNA_104488 | 4.60E-05 | 5.70281 | 3.31044 | 1.2556 |
| hsa_circRNA_104966 | 9.22E-05 | 5.2911 | 2.35551 | 1.25536 |
| hsa_circRNA_104631 | 6.64E-05 | 5.48391 | 2.80452 | 1.25501 |
| hsa_circRNA_103826 | 1.68E-05 | 6.34208 | 4.75964 | 1.25453 |
| hsa_circRNA_103904 | 5.19E-06 | 7.16905 | 6.55996 | 1.25441 |
| hsa_circRNA_100999 | 1.91E-04 | 4.87754 | 1.38413 | 1.25401 |
| hsa_circRNA_103530 | 2.89E-05 | 5.98572 | 3.95736 | 1.25366 |
| hsa_circRNA_100172 | 4.64E-05 | 5.69757 | 3.29838 | 1.25323 |
| hsa_circRNA_104787 | 5.32E-05 | 5.61426 | 3.10632 | 1.2526 |
| hsa_circRNA_100878 | 7.09E-05 | 5.4464 | 2.71739 | 1.25221 |
| hsa_circRNA_104801 | 6.92E-04 | 4.20517 | -0.20528 | 1.25219 |
| hsa_circRNA_103025 | 2.15E-05 | 6.16636 | 4.36587 | 1.25087 |
| hsa_circRNA_103098 | 7.68E-05 | 5.39948 | 2.60826 | 1.25047 |
| hsa_circRNA_400100 | 8.77E-03 | 2.99973 | -2.96783 | 1.25011 |
| hsa_circRNA_101173 | 5.96E-05 | 5.54783 | 2.95271 | 1.25004 |
| hsa_circRNA_100346 | 3.59E-05 | 5.85482 | 3.65906 | 1.24954 |
| hsa_circRNA_103210 | 4.59E-05 | 5.70544 | 3.31649 | 1.24951 |
| hsa_circRNA_104730 | 1.28E-03 | 3.90462 | -0.91185 | 1.2495 |
| hsa_circRNA_101218 | 3.88E-05 | 5.80446 | 3.54383 | 1.24921 |
| hsa_circRNA_100384 | 1.72E-05 | 6.32477 | 4.72101 | 1.24822 |
| hsa_circRNA_100211 | 1.59E-05 | 6.38421 | 4.85347 | 1.24777 |
| hsa_circRNA_104831 | 1.01E-05 | 6.70684 | 5.56478 | 1.24769 |
| hsa_circRNA_104468 | 5.87E-06 | 7.06981 | 6.34873 | 1.24767 |
| hsa_circRNA_100976 | 2.11E-03 | 3.6636 | -1.47274 | 1.24734 |
| hsa_circRNA_100493 | 1.01E-05 | 6.70939 | 5.57035 | 1.24664 |
| hsa_circRNA_103357 | 7.50E-05 | 5.41578 | 2.64621 | 1.24621 |
| hsa_circRNA_103252 | 5.01E-06 | 7.2117 | 6.65032 | 1.24583 |
| hsa_circRNA_100461 | 1.20E-05 | 6.58288 | 5.29305 | 1.24574 |
| hsa_circRNA_000653 | 1.20E-04 | 5.14069 | 2.00336 | 1.24562 |
| hsa_circRNA_103143 | 1.17E-05 | 6.60967 | 5.35194 | 1.24552 |
| hsa_circRNA_100420 | 4.12E-04 | 4.46958 | 0.41966 | 1.24536 |
| hsa_circRNA_103602 | 8.07E-04 | 4.12756 | -0.38828 | 1.24525 |
| hsa_circRNA_105018 | 1.73E-05 | 6.32087 | 4.71229 | 1.24509 |
| hsa_circRNA_102368 | 7.07E-07 | 9.00959 | 10.22762 | 1.24481 |
| hsa_circRNA_103019 | 1.50E-05 | 6.42648 | 4.94743 | 1.24439 |
| hsa_circRNA_101696 | 1.88E-04 | 4.88684 | 1.40607 | 1.24361 |
| hsa_circRNA_102098 | 3.92E-05 | 5.79658 | 3.52578 | 1.24354 |
| hsa_circRNA_002106 | 2.66E-05 | 6.03854 | 4.07718 | 1.24351 |
| hsa_circRNA_101944 | 7.50E-05 | 5.41447 | 2.64315 | 1.24296 |
| hsa_circRNA_104536 | 5.02E-06 | 7.19839 | 6.62216 | 1.24254 |
| hsa_circRNA_102639 | 5.87E-03 | 3.18646 | -2.55704 | 1.24069 |
| hsa_circRNA_100277 | 1.53E-04 | 5.00705 | 1.68931 | 1.24044 |
| hsa_circRNA_103139 | 4.12E-05 | 5.77118 | 3.46753 | 1.24022 |
| hsa_circRNA_104383 | 4.82E-05 | 5.6735 | 3.24297 | 1.24011 |
| hsa_circRNA_103992 | 4.11E-03 | 3.35029 | -2.18964 | 1.24005 |
| hsa_circRNA_104947 | 3.23E-04 | 4.6011 | 0.73084 | 1.23998 |
| hsa_circRNA_102889 | 6.25E-03 | 3.15623 | -2.62415 | 1.23878 |
| hsa_circRNA_103339 | 8.76E-04 | 4.08792 | -0.48162 | 1.23809 |
| hsa_circRNA_102300 | 1.31E-05 | 6.52665 | 5.16915 | 1.23733 |
| hsa_circRNA_104114 | 7.68E-05 | 5.40042 | 2.61045 | 1.23705 |
| hsa_circRNA_101331 | 1.96E-04 | 4.8621 | 1.3477 | 1.23699 |
| hsa_circRNA_103595 | 6.97E-06 | 6.95783 | 6.10875 | 1.23696 |
| hsa_circRNA_102213 | 1.35E-03 | 3.88127 | -0.96646 | 1.23667 |
| hsa_circRNA_102023 | 1.87E-04 | 4.89065 | 1.41504 | 1.23646 |
| hsa_circRNA_100390 | 1.91E-05 | 6.25444 | 4.56371 | 1.236 |
| hsa_circRNA_400064 | 8.73E-04 | 4.08978 | -0.47724 | 1.23575 |
| hsa_circRNA_104500 | 4.32E-05 | 5.73819 | 3.39179 | 1.23564 |
| hsa_circRNA_104495 | 5.62E-04 | 4.31314 | 0.04969 | 1.23425 |
| hsa_circRNA_100751 | 2.49E-03 | 3.58626 | -1.65118 | 1.23395 |
| hsa_circRNA_100836 | 5.14E-05 | 5.63553 | 3.1554 | 1.23391 |
| hsa_circRNA_101585 | 8.35E-05 | 5.34678 | 2.48547 | 1.23282 |
| hsa_circRNA_101054 | 4.13E-06 | 7.35904 | 6.96058 | 1.23091 |
| hsa_circRNA_102773 | 1.25E-03 | 3.91408 | -0.8897 | 1.2304 |
| hsa_circRNA_100795 | 1.23E-03 | 3.92216 | -0.87079 | 1.23028 |
| hsa_circRNA_001830 | 8.42E-05 | 5.34051 | 2.47084 | 1.23004 |
| hsa_circRNA_103476 | 5.25E-04 | 4.34608 | 0.12755 | 1.22998 |
| hsa_circRNA_104946 | 4.08E-04 | 4.4762 | 0.43533 | 1.22933 |
| hsa_circRNA_103169 | 1.62E-04 | 4.97136 | 1.60529 | 1.22899 |
| hsa_circRNA_102824 | 1.26E-04 | 5.11159 | 1.93506 | 1.22806 |
| hsa_circRNA_100143 | 7.55E-05 | 5.41034 | 2.63355 | 1.22777 |
| hsa_circRNA_104437 | 1.11E-04 | 5.18614 | 2.10992 | 1.22775 |
| hsa_circRNA_103297 | 3.58E-05 | 5.85719 | 3.66449 | 1.22724 |
| hsa_circRNA_101512 | 1.12E-04 | 5.17614 | 2.08649 | 1.22717 |
| hsa_circRNA_102109 | 9.15E-05 | 5.29559 | 2.36601 | 1.22709 |
| hsa_circRNA_101856 | 1.20E-05 | 6.58419 | 5.29593 | 1.22708 |
| hsa_circRNA_103799 | 6.45E-05 | 5.50172 | 2.84584 | 1.22658 |
| hsa_circRNA_100946 | 1.26E-04 | 5.11128 | 1.93434 | 1.22576 |
| hsa_circRNA_102452 | 3.87E-05 | 5.80722 | 3.55015 | 1.22561 |
| hsa_circRNA_102381 | 9.78E-04 | 4.03548 | -0.60496 | 1.22546 |
| hsa_circRNA_102233 | 1.77E-06 | 8.02728 | 8.32947 | 1.22543 |
| hsa_circRNA_001363 | 2.59E-03 | 3.56637 | -1.69693 | 1.22539 |
| hsa_circRNA_103789 | 6.08E-05 | 5.53527 | 2.92362 | 1.22524 |
| hsa_circRNA_101545 | 1.11E-04 | 5.18195 | 2.10012 | 1.22444 |
| hsa_circRNA_103363 | 4.62E-05 | 5.69956 | 3.30297 | 1.22436 |
| hsa_circRNA_103105 | 3.67E-05 | 5.83985 | 3.62484 | 1.2227 |
| hsa_circRNA_103756 | 5.46E-05 | 5.59972 | 3.07271 | 1.22221 |
| hsa_circRNA_101053 | 2.06E-05 | 6.20124 | 4.44433 | 1.22192 |
| hsa_circRNA_101056 | 3.55E-04 | 4.55218 | 0.61511 | 1.2214 |
| hsa_circRNA_103080 | 8.17E-05 | 5.35994 | 2.51616 | 1.22138 |
| hsa_circRNA_101076 | 4.48E-03 | 3.31005 | -2.28042 | 1.22122 |
| hsa_circRNA_102396 | 1.05E-04 | 5.21638 | 2.18076 | 1.22046 |
| hsa_circRNA_103584 | 5.93E-04 | 4.28634 | -0.01364 | 1.22014 |
| hsa_circRNA_101365 | 5.02E-06 | 7.19972 | 6.62497 | 1.2184 |
| hsa_circRNA_104021 | 1.87E-04 | 4.89059 | 1.41492 | 1.21829 |
| hsa_circRNA_100565 | 1.09E-05 | 6.65454 | 5.45037 | 1.21802 |
| hsa_circRNA_103710 | 6.97E-05 | 5.45614 | 2.74004 | 1.21751 |
| hsa_circRNA_102105 | 3.13E-04 | 4.62057 | 0.77692 | 1.2171 |
| hsa_circRNA_101159 | 6.51E-04 | 4.2368 | -0.13062 | 1.21699 |
| hsa_circRNA_101032 | 1.43E-06 | 8.26127 | 8.7939 | 1.21689 |
| hsa_circRNA_100069 | 4.95E-04 | 4.3735 | 0.19239 | 1.21617 |
| hsa_circRNA_100640 | 1.32E-05 | 6.51625 | 5.14619 | 1.21529 |
| hsa_circRNA_102205 | 1.16E-02 | 2.86779 | -3.25213 | 1.21516 |
| hsa_circRNA_102682 | 7.58E-04 | 4.15753 | -0.31765 | 1.21491 |
| hsa_circRNA_102713 | 1.65E-04 | 4.959 | 1.57617 | 1.21455 |
| hsa_circRNA_104395 | 3.63E-03 | 3.40974 | -2.05492 | 1.21453 |
| hsa_circRNA_101843 | 2.72E-05 | 6.02518 | 4.04691 | 1.21436 |
| hsa_circRNA_103919 | 3.42E-05 | 5.87986 | 3.71628 | 1.21431 |
| hsa_circRNA_102255 | 5.87E-06 | 7.0679 | 6.34464 | 1.21297 |
| hsa_circRNA_101232 | 4.62E-04 | 4.41179 | 0.28294 | 1.21295 |
| hsa_circRNA_400095 | 4.71E-04 | 4.40014 | 0.2554 | 1.21209 |
| hsa_circRNA_100123 | 4.75E-06 | 7.24643 | 6.72372 | 1.21004 |
| hsa_circRNA_100439 | 2.19E-05 | 6.15532 | 4.34103 | 1.20894 |
| hsa_circRNA_103687 | 9.31E-04 | 4.0576 | -0.55296 | 1.20878 |
| hsa_circRNA_103125 | 4.18E-06 | 7.34993 | 6.94148 | 1.20874 |
| hsa_circRNA_104719 | 2.88E-06 | 7.61914 | 7.50087 | 1.20858 |
| hsa_circRNA_104860 | 3.16E-04 | 4.61498 | 0.76369 | 1.20786 |
| hsa_circRNA_102086 | 1.18E-05 | 6.59676 | 5.32359 | 1.20748 |
| hsa_circRNA_100674 | 1.24E-02 | 2.83694 | -3.31781 | 1.20712 |
| hsa_circRNA_104466 | 1.04E-04 | 5.22226 | 2.19453 | 1.2069 |
| hsa_circRNA_103277 | 5.54E-05 | 5.58825 | 3.04621 | 1.20684 |
| hsa_circRNA_103481 | 8.13E-06 | 6.8587 | 5.89491 | 1.2067 |
| hsa_circRNA_103382 | 1.93E-05 | 6.24889 | 4.55127 | 1.20627 |
| hsa_circRNA_001914 | 1.43E-06 | 8.26318 | 8.79768 | 1.2061 |
| hsa_circRNA_102504 | 8.13E-04 | 4.12416 | -0.39628 | 1.20597 |
| hsa_circRNA_000711 | 1.67E-03 | 3.77695 | -1.20976 | 1.20593 |
| hsa_circRNA_102795 | 4.12E-05 | 5.77126 | 3.46771 | 1.20585 |
| hsa_circRNA_101503 | 1.96E-03 | 3.69916 | -1.39042 | 1.20583 |
| hsa_circRNA_102256 | 1.51E-06 | 8.2099 | 8.6926 | 1.20565 |
| hsa_circRNA_104003 | 4.64E-03 | 3.29349 | -2.31767 | 1.20531 |
| hsa_circRNA_101249 | 9.03E-04 | 4.07261 | -0.51765 | 1.20446 |
| hsa_circRNA_100526 | 5.12E-05 | 5.63923 | 3.16396 | 1.20374 |
| hsa_circRNA_101645 | 1.32E-05 | 6.51232 | 5.13751 | 1.20369 |
| hsa_circRNA_400063 | 8.01E-04 | 4.1315 | -0.37899 | 1.20328 |
| hsa_circRNA_102826 | 1.63E-04 | 4.96622 | 1.59317 | 1.20298 |
| hsa_circRNA_103122 | 6.40E-06 | 7.0137 | 6.2287 | 1.20277 |
| hsa_circRNA_102028 | 2.86E-04 | 4.66676 | 0.88616 | 1.20256 |
| hsa_circRNA_101527 | 1.56E-04 | 4.99271 | 1.65556 | 1.20246 |
| hsa_circRNA_100344 | 3.10E-05 | 5.94507 | 3.86492 | 1.20238 |
| hsa_circRNA_103754 | 4.86E-05 | 5.66564 | 3.22484 | 1.20052 |
| hsa_circRNA_104965 | 7.41E-06 | 6.92115 | 6.02979 | 1.20041 |
| hsa_circRNA_100490 | 2.12E-05 | 6.18022 | 4.39706 | 1.19974 |
| hsa_circRNA_103144 | 1.50E-05 | 6.431 | 4.95745 | 1.19898 |
| hsa_circRNA_102200 | 8.46E-05 | 5.33776 | 2.46443 | 1.19898 |
| hsa_circRNA_104038 | 5.28E-05 | 5.61979 | 3.11908 | 1.19859 |
| hsa_circRNA_100915 | 1.69E-05 | 6.33911 | 4.753 | 1.19824 |
| hsa_circRNA_100977 | 3.69E-03 | 3.4018 | -2.07296 | 1.198 |
| hsa_circRNA_104519 | 5.60E-05 | 5.58215 | 3.0321 | 1.19644 |
| hsa_circRNA_104071 | 5.41E-05 | 5.60404 | 3.0827 | 1.19641 |
| hsa_circRNA_104948 | 3.74E-03 | 3.39499 | -2.0884 | 1.19473 |
| hsa_circRNA_101782 | 4.28E-05 | 5.746 | 3.40972 | 1.19384 |
| hsa_circRNA_104305 | 3.41E-05 | 5.88234 | 3.72193 | 1.19365 |
| hsa_circRNA_100427 | 1.26E-05 | 6.54936 | 5.21925 | 1.19332 |
| hsa_circRNA_101318 | 1.82E-02 | 2.65811 | -3.69208 | 1.19313 |
| hsa_circRNA_100759 | 4.99E-04 | 4.37037 | 0.18499 | 1.19274 |
| hsa_circRNA_101349 | 1.91E-05 | 6.25729 | 4.57009 | 1.19232 |
| hsa_circRNA_101490 | 6.47E-04 | 4.24076 | -0.12129 | 1.19188 |
| hsa_circRNA_103935 | 1.13E-04 | 5.17293 | 2.07898 | 1.19175 |
| hsa_circRNA_104752 | 4.32E-03 | 3.32736 | -2.24141 | 1.19175 |
| hsa_circRNA_104444 | 2.30E-04 | 4.77976 | 1.15327 | 1.19095 |
| hsa_circRNA_104759 | 2.13E-03 | 3.65975 | -1.48164 | 1.19058 |
| hsa_circRNA_103680 | 7.60E-05 | 5.40516 | 2.62149 | 1.19033 |
| hsa_circRNA_104584 | 5.76E-05 | 5.56498 | 2.99239 | 1.18949 |
| hsa_circRNA_102512 | 1.60E-05 | 6.37988 | 4.84384 | 1.18902 |
| hsa_circRNA_104364 | 1.56E-05 | 6.40152 | 4.89197 | 1.189 |
| hsa_circRNA_101736 | 2.78E-04 | 4.68133 | 0.92061 | 1.18787 |
| hsa_circRNA_101974 | 1.56E-06 | 8.16647 | 8.60668 | 1.18784 |
| hsa_circRNA_001583 | 1.08E-04 | 5.20412 | 2.15205 | 1.18723 |
| hsa_circRNA_104823 | 4.24E-05 | 5.75342 | 3.42678 | 1.18699 |
| hsa_circRNA_103367 | 2.12E-05 | 6.1816 | 4.40017 | 1.18685 |
| hsa_circRNA_103000 | 7.61E-05 | 5.40451 | 2.61997 | 1.18659 |
| hsa_circRNA_102249 | 9.69E-06 | 6.73803 | 5.63282 | 1.18524 |
| hsa_circRNA_102583 | 2.85E-05 | 5.99445 | 3.97719 | 1.18522 |
| hsa_circRNA_000754 | 3.09E-04 | 4.6267 | 0.79142 | 1.18401 |
| hsa_circRNA_101184 | 8.28E-03 | 3.02769 | -2.90691 | 1.18359 |
| hsa_circRNA_101274 | 3.72E-05 | 5.829 | 3.60003 | 1.18355 |
| hsa_circRNA_103390 | 1.76E-03 | 3.75098 | -1.27017 | 1.18355 |
| hsa_circRNA_104810 | 9.13E-04 | 4.06749 | -0.5297 | 1.18269 |
| hsa_circRNA_002117 | 3.16E-03 | 3.47374 | -1.90915 | 1.18234 |
| hsa_circRNA_103719 | 4.27E-05 | 5.74812 | 3.4146 | 1.18023 |
| hsa_circRNA_101075 | 5.18E-05 | 5.63184 | 3.1469 | 1.18015 |
| hsa_circRNA_000010 | 5.96E-05 | 5.54668 | 2.95004 | 1.17995 |
| hsa_circRNA_103765 | 1.25E-05 | 6.55213 | 5.22534 | 1.17975 |
| hsa_circRNA_101693 | 1.56E-06 | 8.17891 | 8.63132 | 1.17956 |
| hsa_circRNA_100292 | 3.88E-05 | 5.80418 | 3.54318 | 1.17933 |
| hsa_circRNA_102585 | 1.58E-04 | 4.98805 | 1.6446 | 1.17911 |
| hsa_circRNA_101702 | 3.88E-04 | 4.50551 | 0.50467 | 1.17851 |
| hsa_circRNA_102796 | 1.87E-04 | 4.8888 | 1.4107 | 1.1771 |
| hsa_circRNA_100003 | 2.89E-03 | 3.51537 | -1.81396 | 1.17705 |
| hsa_circRNA_101019 | 7.49E-04 | 4.16468 | -0.30078 | 1.17678 |
| hsa_circRNA_100511 | 6.18E-06 | 7.03896 | 6.28279 | 1.17665 |
| hsa_circRNA_101090 | 4.10E-05 | 5.77347 | 3.47278 | 1.17581 |
| hsa_circRNA_104980 | 6.12E-03 | 3.16646 | -2.60146 | 1.17577 |
| hsa_circRNA_103291 | 4.19E-04 | 4.46129 | 0.40004 | 1.17565 |
| hsa_circRNA_103263 | 2.35E-05 | 6.11381 | 4.24741 | 1.1749 |
| hsa_circRNA_103903 | 3.21E-05 | 5.92142 | 3.81106 | 1.17432 |
| hsa_circRNA_001820 | 1.18E-03 | 3.9443 | -0.81894 | 1.1743 |
| hsa_circRNA_102481 | 3.87E-04 | 4.50698 | 0.50815 | 1.17422 |
| hsa_circRNA_102039 | 1.80E-04 | 4.91065 | 1.46223 | 1.17421 |
| hsa_circRNA_102444 | 4.58E-05 | 5.7071 | 3.32031 | 1.17358 |
| hsa_circRNA_104774 | 3.30E-05 | 5.90032 | 3.76296 | 1.17306 |
| hsa_circRNA_102876 | 3.02E-04 | 4.63726 | 0.81638 | 1.17206 |
| hsa_circRNA_104101 | 2.78E-04 | 4.68111 | 0.9201 | 1.17125 |
| hsa_circRNA_103540 | 1.91E-04 | 4.87903 | 1.38765 | 1.17122 |
| hsa_circRNA_103096 | 3.16E-02 | 2.38803 | -4.23345 | 1.1705 |
| hsa_circRNA_104591 | 5.70E-04 | 4.30633 | 0.03361 | 1.16996 |
| hsa_circRNA_101165 | 6.43E-05 | 5.50344 | 2.84982 | 1.16964 |
| hsa_circRNA_102521 | 9.44E-05 | 5.27655 | 2.32152 | 1.16956 |
| hsa_circRNA_101353 | 2.93E-04 | 4.6536 | 0.85505 | 1.16942 |
| hsa_circRNA_103736 | 5.97E-04 | 4.28249 | -0.02274 | 1.1683 |
| hsa_circRNA_101867 | 1.35E-03 | 3.8789 | -0.97199 | 1.16768 |
| hsa_circRNA_100262 | 1.56E-06 | 8.14906 | 8.57215 | 1.16732 |
| hsa_circRNA_104391 | 9.56E-05 | 5.26801 | 2.30155 | 1.16694 |
| hsa_circRNA_101281 | 3.56E-05 | 5.86009 | 3.67112 | 1.16635 |
| hsa_circRNA_104588 | 8.10E-05 | 5.366 | 2.53028 | 1.16633 |
| hsa_circRNA_101756 | 4.80E-04 | 4.3892 | 0.22953 | 1.16609 |
| hsa_circRNA_102466 | 6.64E-03 | 3.12842 | -2.6857 | 1.16551 |
| hsa_circRNA_101633 | 9.58E-04 | 4.04535 | -0.58175 | 1.16549 |
| hsa_circRNA_100530 | 2.87E-06 | 7.62575 | 7.51448 | 1.16547 |
| hsa_circRNA_103269 | 1.38E-03 | 3.86891 | -0.99536 | 1.16447 |
| hsa_circRNA_104485 | 5.74E-05 | 5.56665 | 2.99625 | 1.16424 |
| hsa_circRNA_101975 | 1.14E-02 | 2.87569 | -3.23527 | 1.16307 |
| hsa_circRNA_102297 | 2.85E-04 | 4.6691 | 0.8917 | 1.16298 |
| hsa_circRNA_100644 | 1.14E-03 | 3.96042 | -0.78115 | 1.16223 |
| hsa_circRNA_104881 | 1.67E-03 | 3.77766 | -1.20812 | 1.16122 |
| hsa_circRNA_102797 | 4.82E-05 | 5.67191 | 3.23929 | 1.16071 |
| hsa_circRNA_100071 | 2.64E-05 | 6.04161 | 4.08415 | 1.15992 |
| hsa_circRNA_100299 | 4.67E-04 | 4.40459 | 0.26591 | 1.15918 |
| hsa_circRNA_400049 | 1.04E-02 | 2.91915 | -3.1421 | 1.15915 |
| hsa_circRNA_102975 | 1.83E-05 | 6.28666 | 4.63585 | 1.15863 |
| hsa_circRNA_100156 | 2.13E-05 | 6.17271 | 4.38017 | 1.15846 |
| hsa_circRNA_100614 | 4.24E-05 | 5.7534 | 3.42672 | 1.15833 |
| hsa_circRNA_105049 | 3.80E-03 | 3.38729 | -2.10588 | 1.15829 |
| hsa_circRNA_103287 | 5.32E-06 | 7.14304 | 6.50474 | 1.15792 |
| hsa_circRNA_100873 | 1.60E-05 | 6.38228 | 4.84919 | 1.15756 |
| hsa_circRNA_101647 | 4.72E-05 | 5.68574 | 3.27115 | 1.15749 |
| hsa_circRNA_102377 | 1.11E-04 | 5.18089 | 2.09762 | 1.1569 |
| hsa_circRNA_103411 | 6.04E-04 | 4.27671 | -0.03638 | 1.15536 |
| hsa_circRNA_102786 | 1.37E-03 | 3.87375 | -0.98404 | 1.15536 |
| hsa_circRNA_103488 | 3.04E-05 | 5.95488 | 3.88724 | 1.15451 |
| hsa_circRNA_101749 | 7.83E-03 | 3.05308 | -2.85141 | 1.15352 |
| hsa_circRNA_103264 | 2.64E-06 | 7.70372 | 7.67452 | 1.15321 |
| hsa_circRNA_101726 | 8.26E-05 | 5.3538 | 2.50183 | 1.15291 |
| hsa_circRNA_100106 | 2.61E-04 | 4.71333 | 0.99628 | 1.15251 |
| hsa_circRNA_102624 | 1.11E-04 | 5.18163 | 2.09937 | 1.1524 |
| hsa_circRNA_104835 | 8.72E-06 | 6.81319 | 5.79629 | 1.15213 |
| hsa_circRNA_104663 | 6.84E-05 | 5.46502 | 2.76066 | 1.15179 |
| hsa_circRNA_102135 | 6.53E-03 | 3.13627 | -2.66835 | 1.15131 |
| hsa_circRNA_105051 | 1.21E-05 | 6.57849 | 5.2834 | 1.1512 |
| hsa_circRNA_100653 | 1.49E-04 | 5.0189 | 1.71719 | 1.15056 |
| hsa_circRNA_100726 | 4.49E-05 | 5.71707 | 3.34326 | 1.15033 |
| hsa_circRNA_104239 | 1.11E-04 | 5.18056 | 2.09686 | 1.15011 |
| hsa_circRNA_100812 | 4.88E-05 | 5.66257 | 3.21776 | 1.14959 |
| hsa_circRNA_100815 | 2.15E-03 | 3.65454 | -1.49369 | 1.14931 |
| hsa_circRNA_102736 | 2.01E-03 | 3.68554 | -1.42197 | 1.14918 |
| hsa_circRNA_103690 | 7.01E-03 | 3.1029 | -2.74199 | 1.14914 |
| hsa_circRNA_104470 | 2.64E-06 | 7.70507 | 7.67727 | 1.14909 |
| hsa_circRNA_100029 | 2.37E-05 | 6.10542 | 4.22847 | 1.14909 |
| hsa_circRNA_103683 | 1.11E-04 | 5.18606 | 2.10975 | 1.14868 |
| hsa_circRNA_103053 | 6.97E-06 | 6.95818 | 6.10951 | 1.14858 |
| hsa_circRNA_104951 | 2.29E-04 | 4.78247 | 1.15969 | 1.14843 |
| hsa_circRNA_101177 | 9.23E-06 | 6.7741 | 5.71136 | 1.14838 |
| hsa_circRNA_101796 | 1.19E-03 | 3.93963 | -0.82988 | 1.14834 |
| hsa_circRNA_104837 | 2.12E-05 | 6.18137 | 4.39967 | 1.14824 |
| hsa_circRNA_100366 | 2.59E-06 | 7.72548 | 7.71902 | 1.14756 |
| hsa_circRNA_101921 | 5.24E-06 | 7.15785 | 6.53619 | 1.14714 |
| hsa_circRNA_000095 | 2.51E-05 | 6.0725 | 4.15408 | 1.14686 |
| hsa_circRNA_100241 | 9.15E-04 | 4.06613 | -0.5329 | 1.14594 |
| hsa_circRNA_102734 | 2.42E-05 | 6.09295 | 4.20032 | 1.14587 |
| hsa_circRNA_100046 | 1.35E-04 | 5.07026 | 1.83797 | 1.14555 |
| hsa_circRNA_103084 | 4.77E-06 | 7.24313 | 6.71676 | 1.14553 |
| hsa_circRNA_101035 | 1.64E-04 | 4.96349 | 1.58676 | 1.14436 |
| hsa_circRNA_101625 | 7.45E-04 | 4.16661 | -0.29624 | 1.14434 |
| hsa_circRNA_101311 | 1.84E-02 | 2.65173 | -3.70521 | 1.14424 |
| hsa_circRNA_102558 | 5.64E-05 | 5.57695 | 3.02008 | 1.14382 |
| hsa_circRNA_102091 | 8.73E-03 | 3.00204 | -2.96282 | 1.1431 |
| hsa_circRNA_100933 | 3.23E-04 | 4.60177 | 0.73244 | 1.14289 |
| hsa_circRNA_101279 | 2.82E-03 | 3.52678 | -1.7878 | 1.14281 |
| hsa_circRNA_101477 | 4.22E-06 | 7.34343 | 6.92785 | 1.14219 |
| hsa_circRNA_101485 | 5.13E-05 | 5.63685 | 3.15845 | 1.14175 |
| hsa_circRNA_101300 | 2.61E-04 | 4.71213 | 0.99345 | 1.14101 |
| hsa_circRNA_103974 | 1.19E-05 | 6.58941 | 5.30742 | 1.14094 |
| hsa_circRNA_101932 | 1.25E-04 | 5.1158 | 1.94495 | 1.1408 |
| hsa_circRNA_104005 | 9.22E-05 | 5.29183 | 2.35721 | 1.14001 |
| hsa_circRNA_103758 | 6.69E-05 | 5.47707 | 2.78865 | 1.13966 |
| hsa_circRNA_100103 | 6.20E-04 | 4.26383 | -0.06681 | 1.13948 |
| hsa_circRNA_104834 | 4.25E-05 | 5.75142 | 3.42217 | 1.13855 |
| hsa_circRNA_101457 | 7.54E-04 | 4.16033 | -0.31106 | 1.13851 |
| hsa_circRNA_101866 | 1.56E-04 | 4.99583 | 1.6629 | 1.13822 |
| hsa_circRNA_103258 | 1.31E-05 | 6.52522 | 5.166 | 1.1378 |
| hsa_circRNA_103187 | 1.73E-05 | 6.32081 | 4.71217 | 1.13771 |
| hsa_circRNA_102027 | 2.39E-04 | 4.761 | 1.10896 | 1.1376 |
| hsa_circRNA_101370 | 1.35E-04 | 5.07307 | 1.84457 | 1.13722 |
| hsa_circRNA_101581 | 1.32E-06 | 8.3587 | 8.98501 | 1.13665 |
| hsa_circRNA_102971 | 1.05E-05 | 6.67562 | 5.49652 | 1.13637 |
| hsa_circRNA_103666 | 4.28E-05 | 5.74547 | 3.40851 | 1.13624 |
| hsa_circRNA_101586 | 2.43E-05 | 6.09064 | 4.19509 | 1.13608 |
| hsa_circRNA_100906 | 1.68E-04 | 4.95007 | 1.55514 | 1.13575 |
| hsa_circRNA_101445 | 1.80E-04 | 4.91028 | 1.46135 | 1.13545 |
| hsa_circRNA_102077 | 2.87E-05 | 5.99219 | 3.97206 | 1.13487 |
| hsa_circRNA_101392 | 4.69E-04 | 4.40188 | 0.2595 | 1.13467 |
| hsa_circRNA_104923 | 1.34E-04 | 5.0763 | 1.85217 | 1.1346 |
| hsa_circRNA_103654 | 1.72E-03 | 3.76312 | -1.24194 | 1.13254 |
| hsa_circRNA_103737 | 1.09E-03 | 3.98268 | -0.72895 | 1.13221 |
| hsa_circRNA_100158 | 4.49E-06 | 7.28902 | 6.81351 | 1.1322 |
| hsa_circRNA_103670 | 9.22E-05 | 5.29174 | 2.35702 | 1.13208 |
| hsa_circRNA_101146 | 8.68E-04 | 4.09261 | -0.47059 | 1.13168 |
| hsa_circRNA_104226 | 1.44E-03 | 3.84795 | -1.04429 | 1.13148 |
| hsa_circRNA_101484 | 6.47E-05 | 5.49912 | 2.83982 | 1.13131 |
| hsa_circRNA_101864 | 1.69E-04 | 4.94575 | 1.54496 | 1.13103 |
| hsa_circRNA_002143 | 1.07E-03 | 3.99291 | -0.70495 | 1.12984 |
| hsa_circRNA_101348 | 3.29E-05 | 5.90383 | 3.77096 | 1.12977 |
| hsa_circRNA_102180 | 4.29E-05 | 5.74254 | 3.40178 | 1.12926 |
| hsa_circRNA_100380 | 3.17E-04 | 4.61055 | 0.75319 | 1.12863 |
| hsa_circRNA_100037 | 6.65E-04 | 4.22611 | -0.15588 | 1.12807 |
| hsa_circRNA_100949 | 6.83E-04 | 4.21169 | -0.1899 | 1.12801 |
| hsa_circRNA_100403 | 8.50E-05 | 5.33453 | 2.45691 | 1.12778 |
| hsa_circRNA_100669 | 1.99E-03 | 3.69141 | -1.40837 | 1.12745 |
| hsa_circRNA_104816 | 1.84E-05 | 6.28087 | 4.62288 | 1.12681 |
| hsa_circRNA_102208 | 1.97E-05 | 6.23748 | 4.52568 | 1.12636 |
| hsa_circRNA_100611 | 2.40E-04 | 4.75866 | 1.10342 | 1.12629 |
| hsa_circRNA_104741 | 5.67E-05 | 5.57406 | 3.01339 | 1.1256 |
| hsa_circRNA_100119 | 9.07E-03 | 2.98353 | -3.00302 | 1.12499 |
| hsa_circRNA_103984 | 1.38E-04 | 5.05926 | 1.81212 | 1.12493 |
| hsa_circRNA_400014 | 1.72E-03 | 3.76406 | -1.23975 | 1.12481 |
| hsa_circRNA_100571 | 5.57E-04 | 4.31797 | 0.06111 | 1.12474 |
| hsa_circRNA_100246 | 1.49E-02 | 2.75198 | -3.49705 | 1.1243 |
| hsa_circRNA_101922 | 6.69E-05 | 5.47856 | 2.79209 | 1.12429 |
| hsa_circRNA_101817 | 4.86E-05 | 5.6657 | 3.22498 | 1.12386 |
| hsa_circRNA_104641 | 5.50E-05 | 5.59414 | 3.05983 | 1.12379 |
| hsa_circRNA_103559 | 3.06E-02 | 2.40452 | -4.20132 | 1.1233 |
| hsa_circRNA_103524 | 1.31E-05 | 6.52339 | 5.16195 | 1.12294 |
| hsa_circRNA_101328 | 5.64E-04 | 4.31152 | 0.04586 | 1.12236 |
| hsa_circRNA_104354 | 1.02E-04 | 5.23177 | 2.21679 | 1.122 |
| hsa_circRNA_103127 | 4.32E-04 | 4.44566 | 0.36308 | 1.12196 |
| hsa_circRNA_100487 | 1.06E-05 | 6.66792 | 5.47968 | 1.12176 |
| hsa_circRNA_101508 | 9.33E-05 | 5.28359 | 2.33797 | 1.12141 |
| hsa_circRNA_102154 | 4.00E-06 | 7.3872 | 7.01953 | 1.12128 |
| hsa_circRNA_104201 | 2.48E-05 | 6.0789 | 4.16856 | 1.12063 |
| hsa_circRNA_001409 | 2.12E-02 | 2.58313 | -3.84543 | 1.12039 |
| hsa_circRNA_104478 | 1.44E-05 | 6.45868 | 5.01883 | 1.12038 |
| hsa_circRNA_103828 | 6.08E-05 | 5.53417 | 2.92106 | 1.11952 |
| hsa_circRNA_101910 | 1.43E-04 | 5.04053 | 1.76809 | 1.11935 |
| hsa_circRNA_103250 | 2.59E-05 | 6.05573 | 4.11613 | 1.119 |
| hsa_circRNA_100527 | 1.11E-04 | 5.1878 | 2.11382 | 1.11872 |
| hsa_circRNA_101481 | 7.70E-04 | 4.14977 | -0.33594 | 1.11864 |
| hsa_circRNA_103629 | 1.86E-04 | 4.89468 | 1.42456 | 1.11853 |
| hsa_circRNA_103228 | 1.96E-04 | 4.86158 | 1.34646 | 1.11787 |
| hsa_circRNA_104677 | 4.77E-05 | 5.67944 | 3.25664 | 1.11747 |
| hsa_circRNA_102289 | 2.14E-04 | 4.81483 | 1.23612 | 1.11716 |
| hsa_circRNA_101043 | 2.22E-02 | 2.56148 | -3.88929 | 1.11697 |
| hsa_circRNA_103111 | 6.89E-04 | 4.20757 | -0.19962 | 1.11603 |
| hsa_circRNA_101648 | 2.59E-04 | 4.71716 | 1.00534 | 1.11544 |
| hsa_circRNA_102427 | 1.58E-05 | 6.39351 | 4.87418 | 1.11529 |
| hsa_circRNA_103317 | 3.17E-05 | 5.9298 | 3.83017 | 1.11497 |
| hsa_circRNA_103035 | 2.98E-04 | 4.64532 | 0.83544 | 1.11491 |
| hsa_circRNA_102407 | 8.33E-05 | 5.34887 | 2.49036 | 1.11475 |
| hsa_circRNA_104832 | 1.22E-04 | 5.13054 | 1.97954 | 1.11446 |
| hsa_circRNA_104362 | 8.42E-06 | 6.83899 | 5.85224 | 1.11375 |
| hsa_circRNA_104329 | 1.70E-04 | 4.94097 | 1.53369 | 1.11242 |
| hsa_circRNA_104221 | 2.87E-06 | 7.62444 | 7.51178 | 1.11211 |
| hsa_circRNA_104120 | 4.35E-06 | 7.30922 | 6.856 | 1.11117 |
| hsa_circRNA_103410 | 3.71E-02 | 2.30855 | -4.38657 | 1.11086 |
| hsa_circRNA_101140 | 4.00E-06 | 7.38521 | 7.01536 | 1.11064 |
| hsa_circRNA_101323 | 9.62E-05 | 5.26429 | 2.29286 | 1.11021 |
| hsa_circRNA_103200 | 3.86E-05 | 5.80945 | 3.55527 | 1.11 |
| hsa_circRNA_100853 | 3.02E-04 | 4.63669 | 0.81504 | 1.10968 |
| hsa_circRNA_104366 | 2.13E-05 | 6.17609 | 4.38778 | 1.10954 |
| hsa_circRNA_100088 | 4.82E-05 | 5.67341 | 3.24274 | 1.10948 |
| hsa_circRNA_102538 | 4.48E-04 | 4.42711 | 0.31919 | 1.10939 |
| hsa_circRNA_100318 | 3.76E-03 | 3.39241 | -2.09426 | 1.10877 |
| hsa_circRNA_102386 | 4.47E-04 | 4.4277 | 0.32058 | 1.10846 |
| hsa_circRNA_103964 | 3.43E-06 | 7.48214 | 7.21746 | 1.10719 |
| hsa_circRNA_102164 | 1.08E-04 | 5.19894 | 2.13991 | 1.10708 |
| hsa_circRNA_102315 | 2.78E-03 | 3.53351 | -1.77237 | 1.10669 |
| hsa_circRNA_102591 | 8.80E-05 | 5.31681 | 2.41555 | 1.10625 |
| hsa_circRNA_101602 | 1.63E-05 | 6.36419 | 4.8089 | 1.10624 |
| hsa_circRNA_104699 | 3.10E-05 | 5.94535 | 3.86556 | 1.10606 |
| hsa_circRNA_101260 | 1.50E-03 | 3.82744 | -1.09216 | 1.10572 |
| hsa_circRNA_100601 | 1.60E-04 | 4.98071 | 1.62731 | 1.10549 |
| hsa_circRNA_104142 | 1.65E-04 | 4.95956 | 1.5775 | 1.10469 |
| hsa_circRNA_102475 | 1.01E-05 | 6.70918 | 5.56989 | 1.10393 |
| hsa_circRNA_103953 | 3.98E-05 | 5.79004 | 3.51078 | 1.10301 |
| hsa_circRNA_100615 | 1.19E-03 | 3.94129 | -0.826 | 1.10284 |
| hsa_circRNA_100189 | 4.01E-04 | 4.48735 | 0.46171 | 1.10281 |
| hsa_circRNA_100671 | 4.07E-04 | 4.47755 | 0.43852 | 1.10262 |
| hsa_circRNA_104431 | 7.07E-05 | 5.44949 | 2.72458 | 1.10256 |
| hsa_circRNA_102457 | 1.23E-04 | 5.12747 | 1.97235 | 1.10221 |
| hsa_circRNA_001896 | 7.83E-04 | 4.14201 | -0.35422 | 1.10159 |
| hsa_circRNA_102801 | 3.64E-04 | 4.53855 | 0.58284 | 1.10156 |
| hsa_circRNA_101294 | 3.09E-03 | 3.4835 | -1.88687 | 1.10138 |
| hsa_circRNA_101878 | 2.12E-05 | 6.17852 | 4.39325 | 1.10021 |
| hsa_circRNA_104952 | 5.37E-05 | 5.60851 | 3.09302 | 1.09956 |
| hsa_circRNA_103755 | 5.92E-05 | 5.5514 | 2.96097 | 1.09939 |
| hsa_circRNA_103462 | 1.41E-05 | 6.47396 | 5.05267 | 1.09933 |
| hsa_circRNA_101655 | 1.58E-05 | 6.39384 | 4.8749 | 1.09924 |
| hsa_circRNA_103497 | 1.79E-04 | 4.91493 | 1.4723 | 1.09915 |
| hsa_circRNA_102766 | 1.75E-03 | 3.75403 | -1.26308 | 1.09912 |
| hsa_circRNA_102951 | 1.77E-04 | 4.91917 | 1.48232 | 1.09861 |
| hsa_circRNA_103788 | 1.41E-05 | 6.47279 | 5.05009 | 1.09856 |
| hsa_circRNA_104745 | 9.53E-03 | 2.96023 | -3.05351 | 1.09843 |
| hsa_circRNA_100328 | 1.16E-03 | 3.95239 | -0.79998 | 1.09838 |
| hsa_circRNA_104357 | 5.31E-05 | 5.61626 | 3.11092 | 1.0978 |
| hsa_circRNA_103254 | 1.60E-02 | 2.71815 | -3.56772 | 1.09763 |
| hsa_circRNA_101715 | 6.81E-04 | 4.21353 | -0.18556 | 1.09761 |
| hsa_circRNA_100124 | 1.58E-04 | 4.98769 | 1.64375 | 1.09719 |
| hsa_circRNA_102397 | 1.04E-02 | 2.91812 | -3.14431 | 1.09719 |
| hsa_circRNA_102524 | 1.53E-04 | 5.00687 | 1.6889 | 1.09709 |
| hsa_circRNA_101684 | 1.13E-03 | 3.96751 | -0.76452 | 1.09686 |
| hsa_circRNA_104775 | 1.27E-04 | 5.10415 | 1.9176 | 1.09673 |
| hsa_circRNA_002050 | 8.04E-06 | 6.86612 | 5.91095 | 1.09626 |
| hsa_circRNA_101321 | 6.74E-05 | 5.47328 | 2.77985 | 1.09615 |
| hsa_circRNA_104333 | 1.50E-05 | 6.42862 | 4.95218 | 1.09611 |
| hsa_circRNA_103766 | 9.17E-04 | 4.06484 | -0.53592 | 1.09611 |
| hsa_circRNA_101206 | 3.37E-04 | 4.57934 | 0.67936 | 1.09578 |
| hsa_circRNA_100120 | 3.21E-02 | 2.38167 | -4.24583 | 1.09554 |
| hsa_circRNA_101064 | 2.85E-03 | 3.52098 | -1.8011 | 1.09498 |
| hsa_circRNA_100720 | 1.69E-03 | 3.7701 | -1.22571 | 1.09481 |
| hsa_circRNA_104014 | 1.39E-05 | 6.48199 | 5.07044 | 1.0947 |
| hsa_circRNA_103171 | 8.04E-05 | 5.37156 | 2.54323 | 1.09436 |
| hsa_circRNA_102637 | 2.06E-05 | 6.20479 | 4.4523 | 1.09415 |
| hsa_circRNA_400031 | 4.58E-03 | 3.29962 | -2.3039 | 1.09409 |
| hsa_circRNA_100195 | 9.29E-05 | 5.28688 | 2.34566 | 1.09404 |
| hsa_circRNA_102536 | 2.48E-06 | 7.77488 | 7.81983 | 1.09368 |
| hsa_circRNA_102616 | 1.61E-05 | 6.37463 | 4.83217 | 1.09357 |
| hsa_circRNA_104213 | 1.82E-04 | 4.90385 | 1.44618 | 1.09351 |
| hsa_circRNA_101467 | 3.59E-05 | 5.85446 | 3.65826 | 1.09295 |
| hsa_circRNA_100978 | 7.75E-03 | 3.0577 | -2.84131 | 1.09275 |
| hsa_circRNA_101098 | 9.66E-06 | 6.74156 | 5.64053 | 1.09185 |
| hsa_circRNA_101651 | 5.13E-05 | 5.63713 | 3.15911 | 1.09177 |
| hsa_circRNA_102525 | 2.23E-04 | 4.79639 | 1.19257 | 1.09122 |
| hsa_circRNA_104953 | 1.07E-05 | 6.6646 | 5.4724 | 1.09086 |
| hsa_circRNA_101979 | 1.96E-05 | 6.23966 | 4.53058 | 1.09032 |
| hsa_circRNA_102197 | 8.33E-05 | 5.34817 | 2.48872 | 1.08941 |
| hsa_circRNA_100782 | 1.07E-02 | 2.90698 | -3.16824 | 1.08915 |
| hsa_circRNA_103112 | 4.22E-03 | 3.33835 | -2.2166 | 1.08898 |
| hsa_circRNA_001701 | 6.66E-05 | 5.4808 | 2.79729 | 1.08877 |
| hsa_circRNA_104309 | 2.48E-04 | 4.74023 | 1.05986 | 1.08869 |
| hsa_circRNA_100762 | 1.35E-03 | 3.8778 | -0.97457 | 1.08857 |
| hsa_circRNA_101834 | 8.43E-06 | 6.83305 | 5.83935 | 1.08782 |
| hsa_circRNA_104861 | 4.04E-04 | 4.48218 | 0.44947 | 1.08724 |
| hsa_circRNA_100166 | 4.07E-03 | 3.35629 | -2.17608 | 1.08718 |
| hsa_circRNA_104753 | 7.29E-04 | 4.17807 | -0.26921 | 1.08652 |
| hsa_circRNA_102913 | 1.70E-04 | 4.94313 | 1.53877 | 1.08614 |
| hsa_circRNA_104702 | 3.97E-04 | 4.49238 | 0.4736 | 1.08571 |
| hsa_circRNA_100649 | 1.17E-05 | 6.60779 | 5.34781 | 1.08529 |
| hsa_circRNA_100950 | 1.32E-04 | 5.08388 | 1.86997 | 1.08526 |
| hsa_circRNA_100845 | 1.91E-02 | 2.63299 | -3.7437 | 1.08512 |
| hsa_circRNA_100811 | 4.37E-05 | 5.73199 | 3.37753 | 1.08493 |
| hsa_circRNA_104695 | 1.12E-03 | 3.97217 | -0.75361 | 1.0848 |
| hsa_circRNA_102529 | 3.39E-04 | 4.57603 | 0.67153 | 1.08452 |
| hsa_circRNA_400015 | 1.04E-02 | 2.91719 | -3.14631 | 1.08429 |
| hsa_circRNA_102507 | 4.35E-06 | 7.31418 | 6.86644 | 1.08407 |
| hsa_circRNA_100605 | 7.41E-05 | 5.42349 | 2.66413 | 1.08329 |
| hsa_circRNA_101882 | 2.10E-02 | 2.58964 | -3.83221 | 1.08282 |
| hsa_circRNA_100058 | 7.80E-04 | 4.14408 | -0.34935 | 1.08218 |
| hsa_circRNA_100809 | 8.41E-05 | 5.34282 | 2.47624 | 1.08142 |
| hsa_circRNA_104417 | 1.75E-04 | 4.92666 | 1.49997 | 1.08047 |
| hsa_circRNA_103351 | 4.05E-04 | 4.48052 | 0.44556 | 1.08018 |
| hsa_circRNA_103625 | 3.02E-04 | 4.63811 | 0.81841 | 1.07946 |
| hsa_circRNA_100880 | 1.58E-05 | 6.39439 | 4.87612 | 1.07899 |
| hsa_circRNA_104154 | 8.39E-06 | 6.84218 | 5.85914 | 1.07874 |
| hsa_circRNA_104471 | 1.15E-03 | 3.95772 | -0.78749 | 1.07779 |
| hsa_circRNA_101617 | 1.02E-03 | 4.01356 | -0.65646 | 1.07775 |
| hsa_circRNA_103981 | 2.07E-04 | 4.83234 | 1.27745 | 1.07768 |
| hsa_circRNA_100152 | 1.86E-04 | 4.89483 | 1.4249 | 1.07751 |
| hsa_circRNA_103589 | 1.83E-05 | 6.28806 | 4.63897 | 1.07746 |
| hsa_circRNA_104999 | 8.95E-05 | 5.30772 | 2.39432 | 1.07738 |
| hsa_circRNA_104520 | 1.23E-04 | 5.12792 | 1.9734 | 1.07684 |
| hsa_circRNA_104077 | 3.78E-04 | 4.51911 | 0.53684 | 1.07648 |
| hsa_circRNA_100757 | 2.13E-05 | 6.17731 | 4.39052 | 1.07645 |
| hsa_circRNA_000621 | 5.35E-05 | 5.61186 | 3.10076 | 1.07588 |
| hsa_circRNA_100432 | 1.60E-03 | 3.79666 | -1.16388 | 1.07556 |
| hsa_circRNA_101435 | 3.33E-06 | 7.51163 | 7.2787 | 1.07523 |
| hsa_circRNA_103623 | 6.21E-04 | 4.26201 | -0.07109 | 1.07485 |
| hsa_circRNA_101033 | 9.37E-03 | 2.96815 | -3.03636 | 1.07455 |
| hsa_circRNA_102037 | 2.87E-04 | 4.6646 | 0.88105 | 1.07454 |
| hsa_circRNA_103435 | 1.09E-02 | 2.89698 | -3.18971 | 1.07418 |
| hsa_circRNA_103048 | 1.25E-04 | 5.11543 | 1.94409 | 1.07375 |
| hsa_circRNA_104548 | 4.19E-04 | 4.46107 | 0.39953 | 1.07321 |
| hsa_circRNA_104521 | 1.31E-05 | 6.52138 | 5.15751 | 1.07308 |
| hsa_circRNA_103050 | 6.70E-05 | 5.47634 | 2.78695 | 1.07286 |
| hsa_circRNA_104840 | 4.19E-05 | 5.75963 | 3.44104 | 1.07199 |
| hsa_circRNA_103370 | 1.38E-04 | 5.05858 | 1.81053 | 1.07195 |
| hsa_circRNA_101413 | 2.33E-04 | 4.77278 | 1.13679 | 1.0715 |
| hsa_circRNA_104629 | 9.82E-06 | 6.72914 | 5.61345 | 1.07121 |
| hsa_circRNA_100438 | 7.79E-03 | 3.05534 | -2.84647 | 1.07064 |
| hsa_circRNA_100587 | 2.40E-03 | 3.60231 | -1.61421 | 1.07061 |
| hsa_circRNA_105050 | 7.12E-03 | 3.09547 | -2.75835 | 1.07046 |
| hsa_circRNA_100756 | 7.89E-05 | 5.38453 | 2.57344 | 1.07038 |
| hsa_circRNA_103412 | 1.25E-03 | 3.91652 | -0.88401 | 1.07034 |
| hsa_circRNA_101283 | 1.91E-05 | 6.25331 | 4.56117 | 1.07012 |
| hsa_circRNA_103204 | 6.88E-04 | 4.20839 | -0.19769 | 1.06972 |
| hsa_circRNA_100242 | 1.18E-03 | 3.94304 | -0.8219 | 1.06936 |
| hsa_circRNA_102330 | 3.40E-04 | 4.57335 | 0.6652 | 1.06786 |
| hsa_circRNA_102204 | 1.35E-04 | 5.07061 | 1.83881 | 1.06761 |
| hsa_circRNA_100096 | 4.70E-05 | 5.68979 | 3.28048 | 1.06749 |
| hsa_circRNA_104853 | 3.30E-05 | 5.90149 | 3.76563 | 1.06742 |
| hsa_circRNA_104660 | 3.24E-05 | 5.91357 | 3.79317 | 1.06705 |
| hsa_circRNA_001090 | 6.34E-04 | 4.25193 | -0.0949 | 1.06675 |
| hsa_circRNA_101510 | 6.27E-06 | 7.02939 | 6.2623 | 1.06663 |
| hsa_circRNA_104941 | 2.94E-05 | 5.97619 | 3.93571 | 1.0663 |
| hsa_circRNA_103226 | 1.12E-04 | 5.17871 | 2.09253 | 1.06616 |
| hsa_circRNA_105028 | 1.91E-03 | 3.71079 | -1.36346 | 1.06609 |
| hsa_circRNA_103450 | 2.10E-06 | 7.91765 | 8.10922 | 1.06603 |
| hsa_circRNA_100243 | 1.89E-03 | 3.71702 | -1.34901 | 1.06594 |
| hsa_circRNA_102973 | 3.05E-05 | 5.95364 | 3.88442 | 1.06576 |
| hsa_circRNA_102623 | 5.26E-06 | 7.15501 | 6.53017 | 1.06556 |
| hsa_circRNA_101119 | 1.20E-03 | 3.93426 | -0.84247 | 1.06526 |
| hsa_circRNA_101687 | 2.07E-05 | 6.1978 | 4.43661 | 1.06508 |
| hsa_circRNA_000569 | 2.28E-03 | 3.62577 | -1.56012 | 1.06447 |
| hsa_circRNA_101334 | 1.93E-04 | 4.8708 | 1.36823 | 1.06404 |
| hsa_circRNA_101431 | 5.30E-05 | 5.61787 | 3.11464 | 1.064 |
| hsa_circRNA_104537 | 1.19E-05 | 6.58864 | 5.30571 | 1.06395 |
| hsa_circRNA_100512 | 1.43E-05 | 6.46105 | 5.02409 | 1.06362 |
| hsa_circRNA_102666 | 1.17E-05 | 6.60722 | 5.34657 | 1.06269 |
| hsa_circRNA_100239 | 6.20E-03 | 3.16051 | -2.61467 | 1.06256 |
| hsa_circRNA_101607 | 4.29E-04 | 4.44901 | 0.37099 | 1.06217 |
| hsa_circRNA_001216 | 1.69E-02 | 2.6923 | -3.62141 | 1.06142 |
| hsa_circRNA_104875 | 1.65E-04 | 4.95778 | 1.5733 | 1.06002 |
| hsa_circRNA_103489 | 3.29E-05 | 5.90392 | 3.77117 | 1.05992 |
| hsa_circRNA_102218 | 7.55E-05 | 5.40992 | 2.63256 | 1.05975 |
| hsa_circRNA_100280 | 1.43E-04 | 5.04195 | 1.77141 | 1.05944 |
| hsa_circRNA_102949 | 4.28E-04 | 4.44993 | 0.37316 | 1.05944 |
| hsa_circRNA_101800 | 1.21E-06 | 8.43397 | 9.13175 | 1.05938 |
| hsa_circRNA_102982 | 1.31E-05 | 6.52508 | 5.16567 | 1.05886 |
| hsa_circRNA_100204 | 1.13E-05 | 6.63095 | 5.39867 | 1.05816 |
| hsa_circRNA_102425 | 1.64E-05 | 6.36234 | 4.8048 | 1.05791 |
| hsa_circRNA_103916 | 3.13E-04 | 4.61995 | 0.77545 | 1.05772 |
| hsa_circRNA_103650 | 1.14E-04 | 5.16777 | 2.06687 | 1.05763 |
| hsa_circRNA_103887 | 1.74E-02 | 2.67749 | -3.65207 | 1.0575 |
| hsa_circRNA_104035 | 1.60E-04 | 4.97933 | 1.62407 | 1.05731 |
| hsa_circRNA_103350 | 4.40E-05 | 5.72673 | 3.36546 | 1.05715 |
| hsa_circRNA_001296 | 7.49E-05 | 5.41706 | 2.64917 | 1.05694 |
| hsa_circRNA_101023 | 1.03E-05 | 6.69114 | 5.53048 | 1.05578 |
| hsa_circRNA_100842 | 1.79E-04 | 4.91466 | 1.47168 | 1.05488 |
| hsa_circRNA_100562 | 8.78E-04 | 4.08637 | -0.48527 | 1.05479 |
| hsa_circRNA_100479 | 1.21E-05 | 6.57766 | 5.28157 | 1.05472 |
| hsa_circRNA_104747 | 1.12E-05 | 6.63716 | 5.41227 | 1.05454 |
| hsa_circRNA_100835 | 9.49E-05 | 5.27317 | 2.3136 | 1.05452 |
| hsa_circRNA_400030 | 8.54E-04 | 4.10112 | -0.45055 | 1.05444 |
| hsa_circRNA_101474 | 1.82E-03 | 3.73562 | -1.30583 | 1.0544 |
| hsa_circRNA_102055 | 7.26E-04 | 4.18094 | -0.26245 | 1.05402 |
| hsa_circRNA_100150 | 1.53E-04 | 5.00706 | 1.68933 | 1.05401 |
| hsa_circRNA_103142 | 1.97E-04 | 4.85707 | 1.33582 | 1.05401 |
| hsa_circRNA_103215 | 2.87E-06 | 7.63178 | 7.52689 | 1.05389 |
| hsa_circRNA_100248 | 2.65E-05 | 6.03929 | 4.0789 | 1.05314 |
| hsa_circRNA_100678 | 7.52E-04 | 4.16126 | -0.30885 | 1.05309 |
| hsa_circRNA_102723 | 3.37E-03 | 3.44412 | -1.97671 | 1.05291 |
| hsa_circRNA_100659 | 1.61E-04 | 4.97707 | 1.61874 | 1.05285 |
| hsa_circRNA_103594 | 4.64E-03 | 3.29261 | -2.31966 | 1.05257 |
| hsa_circRNA_103095 | 1.87E-04 | 4.8921 | 1.41846 | 1.05254 |
| hsa_circRNA_104855 | 1.11E-04 | 5.18746 | 2.11303 | 1.05194 |
| hsa_circRNA_104428 | 1.26E-04 | 5.11153 | 1.93493 | 1.05179 |
| hsa_circRNA_102958 | 2.01E-05 | 6.22315 | 4.49354 | 1.05165 |
| hsa_circRNA_102959 | 5.83E-06 | 7.07517 | 6.36017 | 1.05152 |
| hsa_circRNA_104095 | 4.35E-06 | 7.31566 | 6.86954 | 1.05142 |
| hsa_circRNA_102394 | 7.72E-06 | 6.89295 | 5.96895 | 1.05119 |
| hsa_circRNA_102955 | 1.09E-04 | 5.19567 | 2.13225 | 1.05077 |
| hsa_circRNA_102157 | 1.73E-04 | 4.9325 | 1.51374 | 1.05076 |
| hsa_circRNA_102066 | 6.94E-04 | 4.20291 | -0.21061 | 1.05039 |
| hsa_circRNA_103971 | 2.41E-05 | 6.09606 | 4.20734 | 1.05037 |
| hsa_circRNA_000962 | 2.56E-05 | 6.06139 | 4.12894 | 1.04998 |
| hsa_circRNA_104367 | 6.13E-05 | 5.52842 | 2.90774 | 1.04966 |
| hsa_circRNA_103021 | 7.26E-06 | 6.93292 | 6.05515 | 1.04959 |
| hsa_circRNA_104382 | 2.03E-04 | 4.84089 | 1.29763 | 1.04923 |
| hsa_circRNA_101937 | 8.93E-05 | 5.30864 | 2.39648 | 1.04877 |
| hsa_circRNA_101869 | 1.72E-03 | 3.76301 | -1.2422 | 1.04857 |
| hsa_circRNA_104807 | 1.31E-03 | 3.89419 | -0.93624 | 1.04794 |
| hsa_circRNA_103099 | 7.17E-04 | 4.18733 | -0.24738 | 1.04763 |
| hsa_circRNA_104949 | 2.63E-04 | 4.7083 | 0.9844 | 1.04685 |
| hsa_circRNA_101919 | 1.16E-03 | 3.9543 | -0.79551 | 1.04637 |
| hsa_circRNA_100874 | 2.84E-05 | 5.99764 | 3.98442 | 1.04636 |
| hsa_circRNA_103642 | 1.70E-02 | 2.68984 | -3.62653 | 1.04599 |
| hsa_circRNA_100186 | 3.74E-03 | 3.39472 | -2.08902 | 1.04521 |
| hsa_circRNA_104618 | 2.04E-05 | 6.21363 | 4.47216 | 1.04519 |
| hsa_circRNA_101832 | 3.19E-05 | 5.92648 | 3.82258 | 1.04438 |
| hsa_circRNA_103472 | 2.82E-03 | 3.52622 | -1.78909 | 1.04408 |
| hsa_circRNA_101034 | 1.71E-03 | 3.76694 | -1.23306 | 1.04373 |
| hsa_circRNA_104319 | 2.15E-04 | 4.81221 | 1.22993 | 1.04356 |
| hsa_circRNA_100810 | 1.08E-03 | 3.98753 | -0.71757 | 1.04336 |
| hsa_circRNA_100543 | 1.56E-04 | 4.99497 | 1.66087 | 1.04262 |
| hsa_circRNA_104413 | 2.05E-05 | 6.20802 | 4.45957 | 1.04235 |
| hsa_circRNA_000162 | 5.69E-04 | 4.30757 | 0.03653 | 1.04143 |
| hsa_circRNA_100108 | 1.12E-02 | 2.88416 | -3.21716 | 1.04131 |
| hsa_circRNA_103049 | 7.52E-04 | 4.16173 | -0.30775 | 1.04085 |
| hsa_circRNA_101723 | 2.64E-05 | 6.04331 | 4.088 | 1.04068 |
| hsa_circRNA_102874 | 5.20E-05 | 5.62954 | 3.14159 | 1.04034 |
| hsa_circRNA_101632 | 4.76E-04 | 4.39361 | 0.23996 | 1.04011 |
| hsa_circRNA_103667 | 3.95E-04 | 4.49688 | 0.48426 | 1.03992 |
| hsa_circRNA_100728 | 9.94E-06 | 6.72027 | 5.59409 | 1.03965 |
| hsa_circRNA_101573 | 1.31E-04 | 5.09101 | 1.88673 | 1.03957 |
| hsa_circRNA_104248 | 1.18E-05 | 6.60232 | 5.33581 | 1.03953 |
| hsa_circRNA_103972 | 4.38E-04 | 4.43745 | 0.34366 | 1.0395 |
| hsa_circRNA_102210 | 3.09E-06 | 7.56068 | 7.38026 | 1.03947 |
| hsa_circRNA_102656 | 1.56E-04 | 4.99508 | 1.66115 | 1.03888 |
| hsa_circRNA_001579 | 1.01E-03 | 4.01981 | -0.64177 | 1.03885 |
| hsa_circRNA_103517 | 1.93E-03 | 3.70575 | -1.37513 | 1.03835 |
| hsa_circRNA_102497 | 6.38E-06 | 7.01615 | 6.23394 | 1.03815 |
| hsa_circRNA_100856 | 3.39E-05 | 5.88599 | 3.73027 | 1.03813 |
| hsa_circRNA_100240 | 1.41E-03 | 3.85875 | -1.01908 | 1.03691 |
| hsa_circRNA_101254 | 7.87E-05 | 5.38628 | 2.57752 | 1.03651 |
| hsa_circRNA_104007 | 3.18E-04 | 4.60876 | 0.74897 | 1.03625 |
| hsa_circRNA_104263 | 2.43E-04 | 4.7504 | 1.08391 | 1.03622 |
| hsa_circRNA_104372 | 1.20E-03 | 3.93524 | -0.84017 | 1.03608 |
| hsa_circRNA_102138 | 7.26E-04 | 4.18062 | -0.26321 | 1.03591 |
| hsa_circRNA_103448 | 6.84E-03 | 3.11415 | -2.71719 | 1.03585 |
| hsa_circRNA_100600 | 4.03E-04 | 4.48337 | 0.4523 | 1.03574 |
| hsa_circRNA_102101 | 1.11E-02 | 2.89144 | -3.20157 | 1.03494 |
| hsa_circRNA_104915 | 1.59E-05 | 6.38385 | 4.85269 | 1.03484 |
| hsa_circRNA_102837 | 5.12E-05 | 5.63899 | 3.16339 | 1.0348 |
| hsa_circRNA_102681 | 3.21E-04 | 4.60375 | 0.73711 | 1.03424 |
| hsa_circRNA_102136 | 4.81E-03 | 3.27587 | -2.35725 | 1.03379 |
| hsa_circRNA_103943 | 7.59E-05 | 5.40646 | 2.62451 | 1.03376 |
| hsa_circRNA_100416 | 4.16E-05 | 5.76456 | 3.45235 | 1.03347 |
| hsa_circRNA_101794 | 6.22E-05 | 5.52 | 2.88823 | 1.03312 |
| hsa_circRNA_102014 | 2.29E-04 | 4.78079 | 1.1557 | 1.03287 |
| hsa_circRNA_104281 | 6.58E-05 | 5.48923 | 2.81686 | 1.03279 |
| hsa_circRNA_104994 | 1.52E-04 | 5.01061 | 1.69769 | 1.03278 |
| hsa_circRNA_101821 | 1.76E-04 | 4.92379 | 1.4932 | 1.03207 |
| hsa_circRNA_101578 | 3.99E-04 | 4.48971 | 0.46729 | 1.03195 |
| hsa_circRNA_100201 | 3.41E-05 | 5.88296 | 3.72334 | 1.03122 |
| hsa_circRNA_103478 | 2.04E-02 | 2.6023 | -3.80645 | 1.0312 |
| hsa_circRNA_104442 | 2.28E-02 | 2.5469 | -3.91874 | 1.03107 |
| hsa_circRNA_101595 | 1.22E-03 | 3.92726 | -0.85885 | 1.03104 |
| hsa_circRNA_101257 | 1.74E-04 | 4.92932 | 1.50624 | 1.03097 |
| hsa_circRNA_103126 | 3.95E-04 | 4.49688 | 0.48425 | 1.03014 |
| hsa_circRNA_000006 | 1.19E-05 | 6.59092 | 5.31073 | 1.02908 |
| hsa_circRNA_104972 | 3.64E-04 | 4.53771 | 0.58086 | 1.02901 |
| hsa_circRNA_102379 | 5.81E-03 | 3.19173 | -2.5453 | 1.02822 |
| hsa_circRNA_104112 | 7.58E-05 | 5.40785 | 2.62775 | 1.02704 |
| hsa_circRNA_104427 | 1.11E-03 | 3.97698 | -0.74232 | 1.02688 |
| hsa_circRNA_104559 | 1.65E-05 | 6.35536 | 4.78924 | 1.02581 |
| hsa_circRNA_102251 | 9.56E-05 | 5.26767 | 2.30076 | 1.02568 |
| hsa_circRNA_104133 | 1.37E-03 | 3.87385 | -0.98379 | 1.02557 |
| hsa_circRNA_103013 | 8.42E-05 | 5.34063 | 2.47112 | 1.02492 |
| hsa_circRNA_400079 | 6.14E-05 | 5.52708 | 2.90464 | 1.0244 |
| hsa_circRNA_101738 | 3.56E-04 | 4.55096 | 0.61222 | 1.02422 |
| hsa_circRNA_101290 | 7.36E-03 | 3.08084 | -2.79053 | 1.02342 |
| hsa_circRNA_102338 | 1.18E-03 | 3.94472 | -0.81796 | 1.02322 |
| hsa_circRNA_100677 | 1.33E-04 | 5.08119 | 1.86367 | 1.02316 |
| hsa_circRNA_104233 | 2.59E-04 | 4.71592 | 1.0024 | 1.02309 |
| hsa_circRNA_101394 | 1.56E-04 | 4.99626 | 1.66393 | 1.02195 |
| hsa_circRNA_101604 | 2.23E-04 | 4.79469 | 1.18853 | 1.02147 |
| hsa_circRNA_100019 | 3.05E-04 | 4.6326 | 0.80537 | 1.02129 |
| hsa_circRNA_102024 | 1.39E-04 | 5.0565 | 1.80564 | 1.0211 |
| hsa_circRNA_101638 | 1.94E-04 | 4.86933 | 1.36476 | 1.02108 |
| hsa_circRNA_102679 | 9.66E-03 | 2.95391 | -3.06716 | 1.02068 |
| hsa_circRNA_104269 | 1.21E-04 | 5.13619 | 1.99281 | 1.02053 |
| hsa_circRNA_101322 | 9.85E-03 | 2.94482 | -3.0868 | 1.02025 |
| hsa_circRNA_100668 | 9.79E-03 | 2.94757 | -3.08085 | 1.02016 |
| hsa_circRNA_104474 | 8.78E-04 | 4.08673 | -0.48442 | 1.02014 |
| hsa_circRNA_104093 | 1.45E-04 | 5.03396 | 1.75264 | 1.01944 |
| hsa_circRNA_100755 | 6.93E-04 | 4.20357 | -0.20907 | 1.01905 |
| hsa_circRNA_100107 | 2.31E-04 | 4.77592 | 1.14421 | 1.01851 |
| hsa_circRNA_102418 | 1.39E-05 | 6.48251 | 5.07159 | 1.01816 |
| hsa_circRNA_100982 | 2.58E-04 | 4.71881 | 1.00924 | 1.01794 |
| hsa_circRNA_102752 | 1.49E-04 | 5.01847 | 1.71619 | 1.01788 |
| hsa_circRNA_400098 | 2.12E-05 | 6.18212 | 4.40135 | 1.01761 |
| hsa_circRNA_102074 | 2.23E-05 | 6.14213 | 4.31131 | 1.01707 |
| hsa_circRNA_100149 | 2.41E-04 | 4.75576 | 1.09656 | 1.01702 |
| hsa_circRNA_101847 | 2.30E-04 | 4.77798 | 1.14906 | 1.01649 |
| hsa_circRNA_104865 | 4.33E-03 | 3.32604 | -2.24438 | 1.01639 |
| hsa_circRNA_103509 | 4.08E-03 | 3.35466 | -2.17976 | 1.01632 |
| hsa_circRNA_103648 | 7.07E-05 | 5.44848 | 2.72222 | 1.01623 |
| hsa_circRNA_102422 | 1.59E-04 | 4.98365 | 1.63424 | 1.01537 |
| hsa_circRNA_400039 | 1.65E-05 | 6.35489 | 4.78819 | 1.01493 |
| hsa_circRNA_100305 | 2.61E-05 | 6.04894 | 4.10074 | 1.01403 |
| hsa_circRNA_104555 | 1.98E-03 | 3.69315 | -1.40433 | 1.01377 |
| hsa_circRNA_104321 | 1.64E-03 | 3.7856 | -1.18964 | 1.01364 |
| hsa_circRNA_000581 | 8.32E-04 | 4.11346 | -0.42148 | 1.01346 |
| hsa_circRNA_102859 | 1.29E-03 | 3.9022 | -0.9175 | 1.01264 |
| hsa_circRNA_105007 | 2.15E-05 | 6.16579 | 4.3646 | 1.01221 |
| hsa_circRNA_102345 | 1.77E-04 | 4.92019 | 1.4847 | 1.01219 |
| hsa_circRNA_101207 | 2.70E-05 | 6.0285 | 4.05443 | 1.0121 |
| hsa_circRNA_100372 | 2.50E-05 | 6.07585 | 4.16164 | 1.01204 |
| hsa_circRNA_101208 | 4.15E-04 | 4.46671 | 0.41288 | 1.01168 |
| hsa_circRNA_104664 | 5.37E-05 | 5.60924 | 3.09471 | 1.0115 |
| hsa_circRNA_102146 | 8.74E-06 | 6.80998 | 5.78933 | 1.01146 |
| hsa_circRNA_102214 | 7.33E-05 | 5.42982 | 2.67886 | 1.01028 |
| hsa_circRNA_101081 | 1.71E-05 | 6.33038 | 4.73353 | 1.00905 |
| hsa_circRNA_400062 | 2.79E-05 | 6.01267 | 4.01855 | 1.00884 |
| hsa_circRNA_104560 | 7.45E-04 | 4.1666 | -0.29626 | 1.00876 |
| hsa_circRNA_104563 | 9.14E-05 | 5.29676 | 2.36874 | 1.00861 |
| hsa_circRNA_104805 | 1.11E-03 | 3.97284 | -0.75202 | 1.00816 |
| hsa_circRNA_103119 | 4.59E-05 | 5.70469 | 3.31477 | 1.00805 |
| hsa_circRNA_101123 | 3.57E-04 | 4.54855 | 0.60652 | 1.0077 |
| hsa_circRNA_103113 | 1.90E-04 | 4.88175 | 1.39405 | 1.00767 |
| hsa_circRNA_102584 | 2.66E-05 | 6.03611 | 4.07169 | 1.00755 |
| hsa_circRNA_102026 | 1.25E-03 | 3.91562 | -0.8861 | 1.00723 |
| hsa_circRNA_102112 | 1.30E-04 | 5.09224 | 1.88963 | 1.00645 |
| hsa_circRNA_104246 | 5.02E-06 | 7.19932 | 6.62413 | 1.00638 |
| hsa_circRNA_101501 | 6.69E-05 | 5.47839 | 2.7917 | 1.00636 |
| hsa_circRNA_101597 | 1.63E-03 | 3.78837 | -1.1832 | 1.00625 |
| hsa_circRNA_100548 | 1.18E-04 | 5.15206 | 2.03003 | 1.0062 |
| hsa_circRNA_102038 | 4.15E-04 | 4.46576 | 0.41061 | 1.00583 |
| hsa_circRNA_104363 | 1.69E-04 | 4.94685 | 1.54756 | 1.00531 |
| hsa_circRNA_104416 | 1.23E-04 | 5.12587 | 1.96859 | 1.00506 |
| hsa_circRNA_102705 | 1.49E-04 | 5.01941 | 1.7184 | 1.00477 |
| hsa_circRNA_102058 | 4.64E-04 | 4.40921 | 0.27685 | 1.00468 |
| hsa_circRNA_101930 | 5.34E-04 | 4.33823 | 0.10899 | 1.00463 |
| hsa_circRNA_100857 | 9.53E-06 | 6.74946 | 5.65774 | 1.0037 |
| hsa_circRNA_103881 | 2.69E-02 | 2.46754 | -4.07734 | 1.00334 |
| hsa_circRNA_100983 | 5.25E-05 | 5.62365 | 3.128 | 1.0033 |
| hsa_circRNA_103337 | 1.01E-04 | 5.23841 | 2.23232 | 1.00328 |
| hsa_circRNA_103599 | 6.52E-04 | 4.23655 | -0.13122 | 1.00306 |
| hsa_circRNA_103902 | 1.21E-02 | 2.84696 | -3.29652 | 1.00295 |
| hsa_circRNA_100229 | 5.73E-04 | 4.30263 | 0.02486 | 1.00265 |
| hsa_circRNA_103369 | 9.36E-05 | 5.28184 | 2.33388 | 1.00236 |
| hsa_circRNA_000487 | 6.20E-04 | 4.26365 | -0.06723 | 1.00232 |
| hsa_circRNA_104424 | 5.48E-05 | 5.59599 | 3.06411 | 1.00219 |
| hsa_circRNA_100822 | 4.60E-05 | 5.70299 | 3.31087 | 1.00174 |
| hsa_circRNA_101923 | 3.92E-05 | 5.79663 | 3.52589 | 1.00142 |
| hsa_circRNA_100337 | 1.59E-05 | 6.38505 | 4.85534 | 1.00137 |
| hsa_circRNA_101580 | 2.73E-06 | 7.6724 | 7.61033 | 1.00107 |
| hsa_circRNA_104152 | 3.08E-03 | -3.48529 | -1.88277 | -1.01374 |
| hsa_circRNA_101852 | 4.35E-02 | -2.22888 | -4.53694 | -1.05826 |
| hsa_circRNA_104313 | 6.22E-03 | -3.15868 | -2.61872 | -1.09382 |
| hsa_circRNA_102459 | 3.83E-02 | -2.29209 | -4.41791 | -1.10763 |
| hsa_circRNA_103345 | 6.63E-04 | -4.2277 | -0.15212 | -1.36444 |
| hsa_circRNA_103225 | 9.86E-06 | -6.72603 | 5.60665 | -1.37268 |
| hsa_circRNA_104374 | 4.64E-03 | -3.29292 | -2.31897 | -1.50352 |
| hsa_circRNA_400068 | 7.07E-03 | -3.09921 | -2.75013 | -1.67021 |
| hsa_circRNA_100719 | 1.17E-02 | -2.86269 | -3.26301 | -1.67247 |
| hsa_circRNA_100319 | 3.68E-02 | -2.31327 | -4.37757 | -1.84077 |
| hsa_circRNA_102119 | 1.20E-02 | -2.85225 | -3.28525 | -1.89566 |
| hsa_circRNA_100433 | 1.27E-02 | -2.82678 | -3.33938 | -2.2894 |
| hsa_circRNA_101004 | 3.25E-06 | -7.52506 | 7.30654 | -2.33609 |
| hsa_circRNA_100989 | 3.81E-02 | -2.29498 | -4.41242 | -2.5011 |
| hsa_circRNA_101531 | 3.44E-03 | -3.43381 | -2.00019 | -2.92925 |
| hsa_circRNA_104270 | 9.99E-05 | -5.24407 | 2.24557 | -4.27188 |
| hsa_circRNA_102051 | 7.85E-06 | -6.88193 | 5.94513 | -4.8718 |
| hsa_circRNA_102619 | 5.21E-05 | -5.62755 | 3.137 | -5.20904 |
| hsa_circRNA_102049 | 3.59E-07 | -11.621 | 14.65136 | -6.44344 |
