# Supplementary material for: Whole-transcriptome analysis reveals a potential hsa_circ_0001955/hsa_circ_0000977-mediated miRNA-mRNA regulatory sub-network in colorectal cancer
Source: Aging (Albany NY). 2020 Mar 28;12(6):5259–79. doi: 10.18632/aging.102945 (PMC7138558; doi:10.18632/aging.102945)
Supplement: Supplementary Table 2 [file aging-12-102945-s001..doc]

**Supplementary Table 2. The binding miRNAs of circRNAs predicted by starBase.**

| circRNA ID | miRNA ID |
| --- | --- |
| hsa_circ_0072088 | hsa-miR-377-3p |
| hsa_circ_0072088 | hsa-miR-620 |
| hsa_circ_0072088 | hsa-miR-1270 |
| hsa_circ_0072088 | hsa-miR-3164 |
| hsa_circ_0072088 | hsa-miR-6820-3p |
| hsa_circ_0072088 | hsa-miR-944 |
| hsa_circ_0072088 | hsa-miR-545-3p |
| hsa_circ_0072088 | hsa-miR-624-3p |
| hsa_circ_0072088 | hsa-miR-323b-3p |
| hsa_circ_0072088 | hsa-miR-532-3p |
| hsa_circ_0072088 | hsa-miR-642a-5p |
| hsa_circ_0072088 | hsa-miR-6835-3p |
| hsa_circ_0072088 | hsa-miR-522-3p |
| hsa_circ_0072088 | hsa-miR-224-3p |
| hsa_circ_0000512 | hsa-miR-627-5p |
| hsa_circ_0000512 | hsa-miR-650 |
| hsa_circ_0000512 | hsa-miR-3612 |
| hsa_circ_0000512 | hsa-miR-296-5p |
| hsa_circ_0000512 | hsa-miR-663a |
| hsa_circ_0000512 | hsa-miR-6787-5p |
| hsa_circ_0000512 | hsa-miR-1908-5p |
| hsa_circ_0000512 | hsa-miR-326 |
| hsa_circ_0000512 | hsa-miR-330-5p |
| hsa_circ_0000511 | hsa-miR-627-5p |
| hsa_circ_0000511 | hsa-miR-650 |
| hsa_circ_0000511 | hsa-miR-3612 |
| hsa_circ_0000511 | hsa-miR-296-5p |
| hsa_circ_0000511 | hsa-miR-663a |
| hsa_circ_0000511 | hsa-miR-6787-5p |
| hsa_circ_0000511 | hsa-miR-1908-5p |
| hsa_circ_0000511 | hsa-miR-326 |
| hsa_circ_0000511 | hsa-miR-330-5p |
| hsa_circ_0001955 | hsa-miR-147a |
| hsa_circ_0001955 | hsa-miR-516a-5p |
| hsa_circ_0001955 | hsa-miR-501-3p |
| hsa_circ_0001955 | hsa-miR-502-3p |
| hsa_circ_0001955 | hsa-miR-150-5p |
| hsa_circ_0001955 | hsa-miR-3611 |
| hsa_circ_0001955 | hsa-miR-485-5p |
| hsa_circ_0001955 | hsa-miR-6884-5p |
| hsa_circ_0001955 | hsa-miR-1252-5p |
| hsa_circ_0001955 | hsa-miR-5195-3p |
| hsa_circ_0001955 | hsa-miR-145-5p |
| hsa_circ_0001955 | hsa-miR-642b-5p |
| hsa_circ_0001955 | hsa-miR-212-5p |
| hsa_circ_0001955 | hsa-miR-455-3p |
| hsa_circ_0001955 | hsa-miR-511-3p |
| hsa_circ_0001955 | hsa-miR-489-3p |
| hsa_circ_0001955 | hsa-miR-1296-5p |
| hsa_circ_0001955 | hsa-miR-28-5p |
| hsa_circ_0001955 | hsa-miR-708-5p |
| hsa_circ_0001955 | hsa-miR-3139 |
| hsa_circ_0001955 | hsa-miR-29a-3p |
| hsa_circ_0001955 | hsa-miR-29c-3p |
| hsa_circ_0001955 | hsa-miR-29b-3p |
| hsa_circ_0001955 | hsa-miR-374c-5p |
| hsa_circ_0001955 | hsa-miR-655-3p |
| hsa_circ_0008274 | hsa-miR-487a-3p |
| hsa_circ_0008274 | hsa-miR-154-3p |
| hsa_circ_0008274 | hsa-miR-660-5p |
| hsa_circ_0008274 | hsa-miR-140-3p |
| hsa_circ_0008274 | hsa-miR-526b-5p |
| hsa_circ_0008274 | hsa-miR-656-3p |
| hsa_circ_0001666 | hsa-miR-362-5p |
| hsa_circ_0001666 | hsa-miR-500b-5p |
| hsa_circ_0001666 | hsa-miR-299-3p |
| hsa_circ_0001666 | hsa-miR-493-5p |
| hsa_circ_0001666 | hsa-miR-3200-5p |
| hsa_circ_0001666 | hsa-miR-516b-5p |
| hsa_circ_0001666 | hsa-miR-22-3p |
| hsa_circ_0001666 | hsa-miR-380-3p |
| hsa_circ_0001666 | hsa-miR-3164 |
| hsa_circ_0001666 | hsa-miR-6820-3p |
| hsa_circ_0001666 | hsa-miR-3064-5p |
| hsa_circ_0001666 | hsa-miR-6504-5p |
| hsa_circ_0001666 | hsa-miR-125a-5p |
| hsa_circ_0001666 | hsa-miR-125b-5p |
| hsa_circ_0001666 | hsa-miR-4319 |
| hsa_circ_0001666 | hsa-miR-30b-5p |
| hsa_circ_0001666 | hsa-miR-30a-5p |
| hsa_circ_0001666 | hsa-miR-30c-5p |
| hsa_circ_0001666 | hsa-miR-30d-5p |
| hsa_circ_0001666 | hsa-miR-30e-5p |
| hsa_circ_0001666 | hsa-miR-199b-5p |
| hsa_circ_0001666 | hsa-miR-199a-5p |
| hsa_circ_0001666 | hsa-miR-5094 |
| hsa_circ_0001666 | hsa-miR-6509-5p |
| hsa_circ_0001666 | hsa-miR-376b-3p |
| hsa_circ_0001666 | hsa-miR-376a-3p |
| hsa_circ_0001666 | hsa-miR-493-3p |
| hsa_circ_0001666 | hsa-miR-3612 |
| hsa_circ_0001666 | hsa-miR-650 |
| hsa_circ_0001666 | hsa-miR-330-5p |
| hsa_circ_0001666 | hsa-miR-326 |
| hsa_circ_0001666 | hsa-miR-9-3p |
| hsa_circ_0001666 | hsa-miR-381-3p |
| hsa_circ_0001666 | hsa-miR-300 |
| hsa_circ_0001666 | hsa-miR-576-5p |
| hsa_circ_0001666 | hsa-miR-889-3p |
| hsa_circ_0001666 | hsa-miR-133a-3p |
| hsa_circ_0001666 | hsa-miR-133b |
| hsa_circ_0001666 | hsa-miR-1276 |
| hsa_circ_0001666 | hsa-miR-2355-3p |
| hsa_circ_0001666 | hsa-miR-1270 |
| hsa_circ_0001666 | hsa-miR-620 |
| hsa_circ_0001666 | hsa-miR-486-5p |
| hsa_circ_0001666 | hsa-miR-3918 |
| hsa_circ_0001666 | hsa-miR-3127-5p |
| hsa_circ_0001666 | hsa-miR-506-5p |
| hsa_circ_0001666 | hsa-miR-5579-3p |
| hsa_circ_0001666 | hsa-miR-193a-5p |
| hsa_circ_0001666 | hsa-miR-3613-5p |
| hsa_circ_0001666 | hsa-miR-433-3p |
| hsa_circ_0001666 | hsa-miR-6509-3p |
| hsa_circ_0001666 | hsa-miR-216b-5p |
| hsa_circ_0006220 | hsa-miR-221-3p |
| hsa_circ_0006220 | hsa-miR-222-3p |
| hsa_circ_0006220 | hsa-miR-483-3p |
| hsa_circ_0006220 | hsa-miR-342-3p |
| hsa_circ_0006220 | hsa-miR-520f-3p |
| hsa_circ_0006220 | hsa-miR-214-3p |
| hsa_circ_0006220 | hsa-miR-761 |
| hsa_circ_0006220 | hsa-miR-3619-5p |
| hsa_circ_0000977 | hsa-miR-887-3p |
| hsa_circ_0000977 | hsa-miR-4766-3p |
| hsa_circ_0000977 | hsa-miR-135a-5p |
| hsa_circ_0000977 | hsa-miR-135b-5p |
| hsa_circ_0000977 | hsa-miR-374a-3p |
| hsa_circ_0000977 | hsa-miR-361-5p |
| hsa_circ_0000977 | hsa-miR-874-3p |
| hsa_circ_0000977 | hsa-miR-124-3p |
| hsa_circ_0000977 | hsa-miR-506-3p |
| hsa_circ_0000977 | hsa-miR-330-3p |
| hsa_circ_0043278 | hsa-miR-561-5p |
| hsa_circ_0043278 | hsa-miR-455-3p |
| hsa_circ_0043278 | hsa-miR-526b-5p |
| hsa_circ_0043278 | hsa-miR-221-3p |
| hsa_circ_0043278 | hsa-miR-222-3p |
| hsa_circ_0043278 | hsa-miR-483-3p |
| hsa_circ_0043278 | hsa-miR-342-3p |
| hsa_circ_0043278 | hsa-miR-520f-3p |
| hsa_circ_0043278 | hsa-miR-214-3p |
| hsa_circ_0043278 | hsa-miR-761 |
| hsa_circ_0043278 | hsa-miR-3619-5p |
