# Supplementary material for: Whole-transcriptome analysis reveals a potential hsa_circ_0001955/hsa_circ_0000977-mediated miRNA-mRNA regulatory sub-network in colorectal cancer
Source: Aging (Albany NY). 2020 Mar 28;12(6):5259–79. doi: 10.18632/aging.102945 (PMC7138558; doi:10.18632/aging.102945)
Supplement: Supplementary Table 3 [file aging-12-102945-s002..doc]

**Supplementary Table 3. Identification of differentially expressed miRNAs (DEmiRNAs) between colorectal cancer tissues and adjacent normal tissues.**

| miRNA_ID | adj.P.Val | t | B | Log_2_FC |
| --- | --- | --- | --- | --- |
| hsa-miR-31-5p | 0.000598 | 6.38232 | 4.37305 | 9.153176 |
| hsa-miR-224-5p | 0.000259 | 7.0954 | 5.73669 | 8.99901 |
| hsa-miR-1244 | 3.59E-05 | 9.43153 | 9.67758 | 8.326863 |
| hsa-miR-188-5p | 0.000259 | 7.09302 | 5.73227 | 7.89375 |
| hsa-miR-764 | 0.001075 | 5.84593 | 3.29934 | 7.667327 |
| hsa-miR-301b | 0.000667 | 6.19595 | 4.00447 | 7.589305 |
| hsa-miR-19b-1-5p | 0.001277 | 5.71447 | 3.03032 | 7.257608 |
| hsa-miR-3648 | 0.001333 | 5.65045 | 2.89852 | 7.016826 |
| hsa-miR-452-3p | 0.004439 | 4.83587 | 1.18186 | 6.820998 |
| hsa-miR-3157-3p | 0.00085 | 6.03486 | 3.68201 | 6.796099 |
| hsa-miR-135b-3p | 0.000564 | 6.46267 | 4.53042 | 6.74844 |
| hsa-miR-412 | 0.011995 | 4.19415 | -0.20641 | 6.676802 |
| hsa-miR-19a-5p | 0.001333 | 5.66823 | 2.93516 | 6.589656 |
| hsa-miR-96-5p | 0.009219 | 4.41525 | 0.27383 | 6.575318 |
| hsa-miR-942 | 0.001333 | 5.63551 | 2.86769 | 6.490987 |
| hsa-miR-18a-3p | 0.001094 | 5.82034 | 3.24715 | 6.44523 |
| hsa-miR-135b-5p | 0.003636 | 4.97292 | 1.47518 | 6.269277 |
| hsa-miR-421 | 0.01202 | 4.18832 | -0.21906 | 6.263953 |
| hsa-miR-545-3p | 0.010112 | 4.33951 | 0.10944 | 6.149096 |
| hsa-miR-34a-3p | 0.00019 | 7.79195 | 6.99555 | 6.101058 |
| hsa-miR-4778-3p | 0.009263 | 4.39636 | 0.23284 | 6.05004 |
| hsa-miR-222-5p | 0.001099 | 5.80144 | 3.20854 | 5.992396 |
| hsa-miR-432-3p | 0.003636 | 4.9671 | 1.46276 | 5.927658 |
| hsa-miR-4524a-5p | 0.008098 | 4.49761 | 0.45234 | 5.91181 |
| hsa-miR-4753-3p | 0.009219 | 4.40765 | 0.25733 | 5.885558 |
| hsa-miR-520e | 0.009219 | 4.40452 | 0.25054 | 5.876938 |
| hsa-miR-3650 | 0.011148 | 4.26786 | -0.0462 | 5.870752 |
| hsa-miR-3605-3p | 0.011705 | 4.20998 | -0.17198 | 5.847395 |
| hsa-miR-625-5p | 0.012614 | 4.14777 | -0.30722 | 5.833786 |
| hsa-miR-31-3p | 0.032408 | 3.44671 | -1.82297 | 5.79268 |
| hsa-miR-3609 | 0.009886 | 4.36132 | 0.1568 | 5.706076 |
| hsa-miR-4295 | 0.029155 | 3.56154 | -1.57696 | 5.695541 |
| hsa-miR-4703-3p | 0.036374 | 3.36679 | -1.9933 | 5.686816 |
| hsa-miR-3127-5p | 0.01656 | 3.94564 | -0.74652 | 5.659287 |
| hsa-miR-374a-3p | 0.016316 | 3.96055 | -0.71414 | 5.633182 |
| hsa-miR-155-3p | 0.005275 | 4.72791 | 0.94979 | 5.6194 |
| hsa-miR-3158-3p | 0.014754 | 4.03382 | -0.55494 | 5.511625 |
| hsa-miR-1226-3p | 0.008098 | 4.4941 | 0.44473 | 5.406087 |
| hsa-miR-573 | 0.017595 | 3.87846 | -0.89231 | 5.358664 |
| hsa-miR-4760-5p | 0.029295 | 3.54616 | -1.60997 | 5.310442 |
| hsa-miR-135a-5p | 0.047539 | 3.19524 | -2.35585 | 5.244314 |
| hsa-miR-3130-5p | 0.011148 | 4.25772 | -0.06823 | 5.221793 |
| hsa-miR-433 | 0.028336 | 3.60101 | -1.49209 | 5.18339 |
| hsa-miR-4326 | 0.017138 | 3.90817 | -0.82786 | 5.171544 |
| hsa-miR-3664-5p | 0.031663 | 3.46528 | -1.78328 | 5.158716 |
| hsa-miR-3920 | 0.013808 | 4.09381 | -0.42454 | 5.129842 |
| hsa-miR-3198 | 0.021357 | 3.76355 | -1.14129 | 5.107234 |
| hsa-miR-519b-3p | 0.011148 | 4.27634 | -0.02778 | 5.072691 |
| hsa-miR-744-3p | 0.017521 | 3.89105 | -0.86501 | 5.06762 |
| hsa-miR-3670 | 0.037809 | 3.34713 | -2.03506 | 5.05351 |
| hsa-miR-3116 | 0.035091 | 3.39471 | -1.93389 | 5.020991 |
| hsa-miR-504 | 0.042207 | 3.27725 | -2.18309 | 4.895788 |
| hsa-miR-450b-5p | 0.040006 | 3.31488 | -2.10346 | 4.879503 |
| hsa-miR-1915-5p | 0.044535 | 3.24427 | -2.25269 | 4.865831 |
| hsa-miR-3913-3p | 0.018465 | 3.84303 | -0.96914 | 4.860894 |
| hsa-miR-4495 | 0.017595 | 3.8786 | -0.892 | 4.823268 |
| hsa-miR-3121-5p | 0.029295 | 3.5435 | -1.6157 | 4.821022 |
| hsa-miR-548d-5p | 0.024381 | 3.69302 | -1.29374 | 4.790041 |
| hsa-miR-296-5p | 0.048689 | 3.17435 | -2.39966 | 4.761064 |
| hsa-miR-4254 | 0.024528 | 3.68539 | -1.31022 | 4.709172 |
| hsa-miR-515-3p | 0.016169 | 3.96853 | -0.69679 | 4.676365 |
| hsa-miR-500a-3p | 0.028555 | 3.59452 | -1.50605 | 4.671667 |
| hsa-miR-4664-5p | 0.019783 | 3.80561 | -1.05023 | 4.655108 |
| hsa-miR-4680-3p | 0.049609 | 3.1497 | -2.45126 | 4.606622 |
| hsa-miR-1263 | 0.047539 | 3.19135 | -2.364 | 4.566077 |
| hsa-miR-105-5p | 0.03263 | 3.43937 | -1.83865 | 4.536957 |
| hsa-miR-5009-5p | 0.048639 | 3.17688 | -2.39435 | 4.512451 |
| hsa-miR-4307 | 0.040342 | 3.30884 | -2.11627 | 4.475916 |
| hsa-miR-4736 | 0.041667 | 3.28527 | -2.16614 | 4.450466 |
| hsa-miR-15a-3p | 0.021357 | 3.7616 | -1.14549 | 4.428238 |
| hsa-miR-760 | 0.032408 | 3.44489 | -1.82686 | 4.313043 |
| hsa-miR-5695 | 0.028655 | 3.58621 | -1.52392 | 4.265596 |
| hsa-miR-3681-5p | 0.04351 | 3.25915 | -2.22132 | 4.235457 |
| hsa-miR-1207-3p | 0.039846 | 3.31898 | -2.09478 | 4.213706 |
| hsa-miR-521 | 0.028655 | 3.58492 | -1.5267 | 4.171604 |
| hsa-miR-5692c | 0.029747 | 3.51586 | -1.67499 | 4.170257 |
| hsa-miR-501-3p | 0.041017 | 3.29684 | -2.14166 | 4.15469 |
| hsa-miR-4766-3p | 0.029634 | 3.5276 | -1.6498 | 4.144989 |
| hsa-miR-32-5p | 0.048722 | 3.172 | -2.40459 | 4.1374 |
| hsa-miR-181a-3p | 0.047539 | 3.19221 | -2.36219 | 4.129411 |
| hsa-miR-4517 | 0.04473 | 3.23627 | -2.26956 | 4.121364 |
| hsa-miR-4647 | 0.028771 | 3.57914 | -1.53912 | 4.091355 |
| hsa-miR-182-5p | 0.022402 | 3.73385 | -1.20552 | 3.955943 |
| hsa-miR-18b-5p | 0.029295 | 3.54525 | -1.61194 | 3.639661 |
| hsa-miR-3165 | 0.046329 | 3.21781 | -2.30841 | 3.406193 |
| hsa-miR-20a-3p | 0.021902 | 3.74713 | -1.17681 | 3.123627 |
| hsa-miR-20b-5p | 0.011148 | 4.26091 | -0.0613 | 2.790386 |
| hsa-miRPlus-A1086 | 0.002668 | 5.20469 | 1.96749 | 2.581994 |
| hsa-miR-203 | 0.039326 | 3.32718 | -2.0774 | 2.465661 |
| hsa-miR-142-3p | 0.002384 | 5.29568 | 2.15932 | 2.319546 |
| hsa-miR-3682-5p | 0.014754 | 4.04306 | -0.53486 | 2.220859 |
| hsa-miR-20a-5p | 0.018103 | 3.85872 | -0.93512 | 2.092364 |
| hsa-miR-657 | 0.014754 | 4.03988 | -0.54178 | 2.074754 |
| hsa-miR-4694-5p | 0.030816 | 3.47992 | -1.75196 | 2.069133 |
| hsa-miR-708-5p | 0.00299 | 5.10154 | 1.749 | 1.99028 |
| hsa-miR-424-5p | 0.002668 | 5.20414 | 1.96632 | 1.982967 |
| hsa-miR-4704-5p | 0.004814 | 4.79145 | 1.08647 | 1.976799 |
| hsa-miR-3651 | 0.00096 | 5.91777 | 3.44539 | 1.88966 |
| hsa-miR-16-1-3p | 0.030368 | 3.49797 | -1.71332 | 1.857042 |
| hsa-miR-3120-5p | 0.016968 | 3.92004 | -0.8021 | 1.832926 |
| hsa-miR-3119 | 0.034917 | 3.40176 | -1.91887 | 1.792915 |
| hsa-miR-4645-3p | 0.030608 | 3.49065 | -1.72899 | 1.776187 |
| hsa-miR-5689 | 0.027181 | 3.62726 | -1.43556 | 1.74196 |
| hsa-miR-766-5p | 0.000259 | 7.1608 | 5.85798 | 1.737138 |
| hsa-miR-3152-5p | 0.018044 | 3.86367 | -0.92439 | 1.712773 |
| hsa-miR-4425 | 0.029634 | 3.52628 | -1.65264 | 1.694044 |
| hsa-miR-130b-5p | 0.000639 | 6.29965 | 4.21017 | 1.663375 |
| hsa-miR-4297 | 0.027181 | 3.62257 | -1.44566 | 1.596456 |
| hsa-miR-7-5p | 0.029155 | 3.56345 | -1.57284 | 1.572599 |
| hsa-miR-500a-5p/hsa-miR-500b | 0.027181 | 3.62357 | -1.44352 | 1.499573 |
| hsa-miR-345-5p | 0.009977 | 4.35134 | 0.13513 | 1.497689 |
| hsa-miR-200c-5p | 0.005085 | 4.75175 | 1.00111 | 1.497167 |
| hsa-miR-3160-5p | 0.001719 | 5.48951 | 2.56498 | 1.474143 |
| hsa-miR-452-5p | 0.000639 | 6.26548 | 4.14256 | 1.454915 |
| hsa-miR-761 | 0.003755 | 4.944 | 1.4134 | 1.432136 |
| hsa-miR-330-3p | 0.030341 | 3.5024 | -1.70382 | 1.412499 |
| hsa-miR-223-3p | 0.014688 | 4.05745 | -0.50359 | 1.411617 |
| hsa-miR-3944-5p | 0.029155 | 3.56538 | -1.56871 | 1.408022 |
| hsa-miR-5690 | 0.001658 | 5.51969 | 2.62775 | 1.402476 |
| hsa-miR-342-5p | 0.002384 | 5.28917 | 2.14562 | 1.397874 |
| hsa-miR-4715-3p | 0.02967 | 3.52038 | -1.66529 | 1.367566 |
| hsa-miR-17-3p | 0.029634 | 3.52731 | -1.65042 | 1.356156 |
| hsa-miR-4677-3p | 0.003989 | 4.90826 | 1.33698 | 1.343031 |
| hsa-miR-636 | 0.002434 | 5.26827 | 2.1016 | 1.339295 |
| hsa-miR-17-5p | 0.032055 | 3.45727 | -1.8004 | 1.307585 |
| hsa-miR-5699 | 0.00303 | 5.08592 | 1.71583 | 1.276496 |
| hsa-miR-106a-5p | 0.011279 | 4.23818 | -0.11069 | 1.267884 |
| hsa-miR-552 | 0.015423 | 3.99544 | -0.63833 | 1.25313 |
| hsa-miR-186-3p | 0.003574 | 5.00143 | 1.53601 | 1.240058 |
| hsa-miR-4788 | 0.029295 | 3.54678 | -1.60866 | 1.229843 |
| hsa-miR-19a-3p | 0.024572 | 3.67992 | -1.32201 | 1.218829 |
| hsa-miR-4756-3p | 0.004323 | 4.85652 | 1.22615 | 1.21131 |
| hsa-miR-4659b-5p | 0.011403 | 4.22665 | -0.13575 | 1.200098 |
| hsa-miR-4735-5p | 0.011148 | 4.25727 | -0.06922 | 1.190955 |
| hsa-miR-4436b-3p | 0.028655 | 3.58392 | -1.52885 | 1.171406 |
| hsa-miR-645 | 0.0347 | 3.40944 | -1.90251 | 1.152207 |
| hsa-miR-5006-3p | 0.017164 | 3.90381 | -0.83731 | 1.130226 |
| hsa-miR-4434 | 0.026016 | 3.65141 | -1.38352 | 1.126765 |
| hsa-miR-1285-3p | 0.049322 | 3.15432 | -2.44159 | 1.114946 |
| hsa-miR-183-5p | 0.011279 | 4.23659 | -0.11415 | 1.095285 |
| hsa-miR-622 | 0.008188 | 4.47758 | 0.40895 | 1.072782 |
| hsa-miR-29b-1-5p | 0.014754 | 4.0366 | -0.54889 | 1.072436 |
| hsa-miR-1252 | 0.015158 | 4.00934 | -0.60813 | 1.065881 |
| hsa-miR-659-5p | 0.012485 | 4.15703 | -0.2871 | 1.037224 |
| hsa-miR-1258 | 0.015158 | 4.01076 | -0.60504 | 1.013203 |
| hsa-miR-92b-3p | 0.012983 | 4.13031 | -0.34519 | 1.006851 |
| hsa-miR-4530 | 0.029295 | 3.54239 | -1.61807 | 1.004748 |
| hsa-miR-30c-5p | 0.032327 | -3.45099 | -1.81382 | -1.05813 |
| hsa-miR-371b-5p | 0.000389 | -6.72534 | 5.03837 | -1.10639 |
| hsa-miR-4787-5p | 0.000916 | -5.95895 | 3.52882 | -1.16248 |
| hsa-miR-933 | 0.016617 | -3.93688 | -0.76553 | -1.44821 |
| hsa-miR-195-5p | 0.029295 | -3.54319 | -1.61637 | -1.47186 |
| hsa-miR-28-3p | 0.008098 | -4.49601 | 0.44887 | -1.53795 |
| hsa-miR-26a-5p | 0.002766 | -5.16667 | 1.88706 | -1.6513 |
| hsa-miR-125a-5p | 0.000881 | -5.99753 | 3.60678 | -1.67705 |
| hsa-miR-378d | 0.029634 | -3.52829 | -1.64834 | -1.68283 |
| hsa-miR-100-5p | 0.008073 | -4.5226 | 0.50645 | -1.7255 |
| hsa-miR-943 | 0.001333 | -5.63744 | 2.87166 | -1.74328 |
| hsa-miR-145-5p | 0.000564 | -6.44299 | 4.49195 | -1.77101 |
| hsa-miR-4328 | 0.00019 | -7.4668 | 6.41697 | -1.77448 |
| hsa-miR-99a-5p | 0.010266 | -4.32703 | 0.08235 | -1.79788 |
| hsa-miR-378a-5p | 0.048014 | -3.18479 | -2.37777 | -1.80068 |
| hsa-miR-125b-5p | 0.002163 | -5.35786 | 2.28991 | -1.85079 |
| hsa-miR-4708-3p | 4.99E-05 | -8.82169 | 8.72482 | -1.85594 |
| hsa-miR-29c-5p | 0.005383 | -4.71152 | 0.91449 | -1.86139 |
| hsa-miR-548ap-5p/hsa-miR-548j | 0.00019 | -7.53206 | 6.53436 | -1.93751 |
| hsa-miR-143-3p | 0.002132 | -5.37679 | 2.32958 | -1.95061 |
| hsa-miR-4324 | 0.015158 | -4.01713 | -0.59121 | -1.99105 |
| hsa-miR-497-5p | 0.004967 | -4.76978 | 1.03989 | -2.02043 |
| hsa-miR-548at-5p | 0.000639 | -6.28858 | 4.18828 | -2.42233 |
| hsa-miR-3656 | 0.035191 | -3.38866 | -1.94676 | -2.47814 |
| hsa-miR-145-3p | 0.000642 | -6.23805 | 4.08817 | -2.64975 |
| hsa-miR-133b | 0.00019 | -7.6361 | 6.72019 | -3.43307 |
| hsa-miR-133a | 0.000291 | -6.9795 | 5.52018 | -3.47906 |
| hsa-miR-139-5p | 0.000669 | -6.17235 | 3.95745 | -3.5918 |
| hsa-miR-363-3p | 0.029747 | -3.51384 | -1.67931 | -6.57816 |
