# Supplementary material for: Whole-transcriptome analysis reveals a potential hsa_circ_0001955/hsa_circ_0000977-mediated miRNA-mRNA regulatory sub-network in colorectal cancer
Source: Aging (Albany NY). 2020 Mar 28;12(6):5259–79. doi: 10.18632/aging.102945 (PMC7138558; doi:10.18632/aging.102945)
Supplement: Supplementary Table 4 [file aging-12-102945-s003..doc]

**Supplementary Table 4. The target genes of hsa-miR-145-5p and hsa-miR-135b-5p predicted by miRNet.**

| miRNA ID | Target gene |
| --- | --- |
| hsa-mir-145-5p | ACTB |
| hsa-mir-145-5p | ADD3 |
| hsa-mir-145-5p | AP1G1 |
| hsa-mir-145-5p | JAG1 |
| hsa-mir-145-5p | CRYBG1 |
| hsa-mir-145-5p | ALDH3A1 |
| hsa-mir-145-5p | ALPPL2 |
| hsa-mir-145-5p | ANGPT2 |
| hsa-mir-145-5p | ARF6 |
| hsa-mir-145-5p | BNIP3 |
| hsa-mir-145-5p | BRAF |
| hsa-mir-145-5p | KLF5 |
| hsa-mir-145-5p | BTG1 |
| hsa-mir-145-5p | VPS51 |
| hsa-mir-145-5p | MYRF |
| hsa-mir-145-5p | CBFB |
| hsa-mir-145-5p | CD28 |
| hsa-mir-145-5p | CD40 |
| hsa-mir-145-5p | CD44 |
| hsa-mir-145-5p | CDH2 |
| hsa-mir-145-5p | CDK4 |
| hsa-mir-145-5p | CDK6 |
| hsa-mir-145-5p | CDKN1A |
| hsa-mir-145-5p | CFTR |
| hsa-mir-145-5p | COL5A1 |
| hsa-mir-145-5p | CTGF |
| hsa-mir-145-5p | CTNND1 |
| hsa-mir-145-5p | CYP2C19 |
| hsa-mir-145-5p | DDC |
| hsa-mir-145-5p | DDX6 |
| hsa-mir-145-5p | DMXL1 |
| hsa-mir-145-5p | DFFA |
| hsa-mir-145-5p | SLC26A2 |
| hsa-mir-145-5p | HBEGF |
| hsa-mir-145-5p | DUSP6 |
| hsa-mir-145-5p | E2F3 |
| hsa-mir-145-5p | EGFR |
| hsa-mir-145-5p | EIF4E |
| hsa-mir-145-5p | EPAS1 |
| hsa-mir-145-5p | ERBB4 |
| hsa-mir-145-5p | ERG |
| hsa-mir-145-5p | ESR1 |
| hsa-mir-145-5p | ETS1 |
| hsa-mir-145-5p | FLI1 |
| hsa-mir-145-5p | FXN |
| hsa-mir-145-5p | GMFB |
| hsa-mir-145-5p | HDAC2 |
| hsa-mir-145-5p | HIF1A |
| hsa-mir-145-5p | HOXA9 |
| hsa-mir-145-5p | IFNB1 |
| hsa-mir-145-5p | IGF1R |
| hsa-mir-145-5p | IGFBP5 |
| hsa-mir-145-5p | ILK |
| hsa-mir-145-5p | IRS1 |
| hsa-mir-145-5p | ITGB8 |
| hsa-mir-145-5p | KRT7 |
| hsa-mir-145-5p | SMAD2 |
| hsa-mir-145-5p | SMAD3 |
| hsa-mir-145-5p | SMAD4 |
| hsa-mir-145-5p | SMAD5 |
| hsa-mir-145-5p | MCM2 |
| hsa-mir-145-5p | MDM2 |
| hsa-mir-145-5p | MAP3K3 |
| hsa-mir-145-5p | MEST |
| hsa-mir-145-5p | MAP3K11 |
| hsa-mir-145-5p | MMP1 |
| hsa-mir-145-5p | MMP12 |
| hsa-mir-145-5p | MMP14 |
| hsa-mir-145-5p | ABCC1 |
| hsa-mir-145-5p | MSH3 |
| hsa-mir-145-5p | NUDT1 |
| hsa-mir-145-5p | MUC1 |
| hsa-mir-145-5p | MUC4 |
| hsa-mir-145-5p | MYC |
| hsa-mir-145-5p | MYO5A |
| hsa-mir-145-5p | MYO6 |
| hsa-mir-145-5p | NAIP |
| hsa-mir-145-5p | NDUFA4 |
| hsa-mir-145-5p | NDUFS2 |
| hsa-mir-145-5p | NEDD9 |
| hsa-mir-145-5p | NFATC1 |
| hsa-mir-145-5p | NRAS |
| hsa-mir-145-5p | ORC4 |
| hsa-mir-145-5p | P4HA1 |
| hsa-mir-145-5p | SERPINE1 |
| hsa-mir-145-5p | PIGF |
| hsa-mir-145-5p | PLAGL2 |
| hsa-mir-145-5p | PODXL |
| hsa-mir-145-5p | POU5F1 |
| hsa-mir-145-5p | PPP3CA |
| hsa-mir-145-5p | MAP2K6 |
| hsa-mir-145-5p | PXN |
| hsa-mir-145-5p | MAP4K2 |
| hsa-mir-145-5p | REL |
| hsa-mir-145-5p | ROBO2 |
| hsa-mir-145-5p | ROCK1 |
| hsa-mir-145-5p | RPA1 |
| hsa-mir-145-5p | RPS6KA3 |
| hsa-mir-145-5p | RPS6KB1 |
| hsa-mir-145-5p | RREB1 |
| hsa-mir-145-5p | RTKN |
| hsa-mir-145-5p | MAP2K4 |
| hsa-mir-145-5p | SET |
| hsa-mir-145-5p | HLTF |
| hsa-mir-145-5p | FSCN1 |
| hsa-mir-145-5p | SNTB1 |
| hsa-mir-145-5p | SOX2 |
| hsa-mir-145-5p | SOX9 |
| hsa-mir-145-5p | SOX11 |
| hsa-mir-145-5p | SP1 |
| hsa-mir-145-5p | SPTBN1 |
| hsa-mir-145-5p | STAT1 |
| hsa-mir-145-5p | ADAM17 |
| hsa-mir-145-5p | TGFB2 |
| hsa-mir-145-5p | TGFBI |
| hsa-mir-145-5p | TGFBR2 |
| hsa-mir-145-5p | TSPAN6 |
| hsa-mir-145-5p | TNR |
| hsa-mir-145-5p | TPM3 |
| hsa-mir-145-5p | VEGFA |
| hsa-mir-145-5p | YES1 |
| hsa-mir-145-5p | ZBTB25 |
| hsa-mir-145-5p | PRDM2 |
| hsa-mir-145-5p | DEK |
| hsa-mir-145-5p | PTP4A2 |
| hsa-mir-145-5p | HMGA2 |
| hsa-mir-145-5p | FZD6 |
| hsa-mir-145-5p | FZD7 |
| hsa-mir-145-5p | HIST1H2BF |
| hsa-mir-145-5p | PPM1D |
| hsa-mir-145-5p | NIPSNAP1 |
| hsa-mir-145-5p | AGPS |
| hsa-mir-145-5p | IRS2 |
| hsa-mir-145-5p | TNFSF13 |
| hsa-mir-145-5p | SLC16A5 |
| hsa-mir-145-5p | KLF4 |
| hsa-mir-145-5p | ZFYVE9 |
| hsa-mir-145-5p | CLINT1 |
| hsa-mir-145-5p | VGLL4 |
| hsa-mir-145-5p | AQR |
| hsa-mir-145-5p | PHACTR2 |
| hsa-mir-145-5p | PLEKHM1 |
| hsa-mir-145-5p | NR1D2 |
| hsa-mir-145-5p | PAK4 |
| hsa-mir-145-5p | FAM3C |
| hsa-mir-145-5p | DDX17 |
| hsa-mir-145-5p | ARL6IP5 |
| hsa-mir-145-5p | SPTLC1 |
| hsa-mir-145-5p | IVNS1ABP |
| hsa-mir-145-5p | LYPLA2 |
| hsa-mir-145-5p | SWAP70 |
| hsa-mir-145-5p | ANKRD28 |
| hsa-mir-145-5p | ZNF451 |
| hsa-mir-145-5p | WSB1 |
| hsa-mir-145-5p | SRPX2 |
| hsa-mir-145-5p | SNX24 |
| hsa-mir-145-5p | TMOD3 |
| hsa-mir-145-5p | SENP1 |
| hsa-mir-145-5p | PADI1 |
| hsa-mir-145-5p | PNMA3 |
| hsa-mir-145-5p | PSAT1 |
| hsa-mir-145-5p | SOCS7 |
| hsa-mir-145-5p | F11R |
| hsa-mir-145-5p | APH1A |
| hsa-mir-145-5p | GOLM1 |
| hsa-mir-145-5p | PANK1 |
| hsa-mir-145-5p | DNAJC28 |
| hsa-mir-145-5p | C1orf27 |
| hsa-mir-145-5p | TUG1 |
| hsa-mir-145-5p | UBR7 |
| hsa-mir-145-5p | KIF21A |
| hsa-mir-145-5p | TMEM9B |
| hsa-mir-145-5p | CTNNBIP1 |
| hsa-mir-145-5p | AKR1B10 |
| hsa-mir-145-5p | AGTRAP |
| hsa-mir-145-5p | CAMK1D |
| hsa-mir-145-5p | NDRG2 |
| hsa-mir-145-5p | SRGAP1 |
| hsa-mir-145-5p | NUFIP2 |
| hsa-mir-145-5p | CRAMP1 |
| hsa-mir-145-5p | ABHD17C |
| hsa-mir-145-5p | ABRACL |
| hsa-mir-145-5p | ZFAND3 |
| hsa-mir-145-5p | CLSTN2 |
| hsa-mir-145-5p | RRAGC |
| hsa-mir-145-5p | MTMR14 |
| hsa-mir-145-5p | ZNF426 |
| hsa-mir-145-5p | PARP8 |
| hsa-mir-145-5p | ALG9 |
| hsa-mir-145-5p | HDAC11 |
| hsa-mir-145-5p | NANOG |
| hsa-mir-145-5p | JADE1 |
| hsa-mir-145-5p | CSRNP3 |
| hsa-mir-145-5p | PDGFD |
| hsa-mir-145-5p | CPEB4 |
| hsa-mir-145-5p | MAP1LC3B |
| hsa-mir-145-5p | SESN2 |
| hsa-mir-145-5p | MIXL1 |
| hsa-mir-145-5p | IMMP2L |
| hsa-mir-145-5p | KREMEN1 |
| hsa-mir-145-5p | UTP15 |
| hsa-mir-145-5p | DDI2 |
| hsa-mir-145-5p | LMNB2 |
| hsa-mir-145-5p | CEP19 |
| hsa-mir-145-5p | HIST1H2AH |
| hsa-mir-145-5p | MTDH |
| hsa-mir-145-5p | RBM18 |
| hsa-mir-145-5p | DTD1 |
| hsa-mir-145-5p | MYOCD |
| hsa-mir-145-5p | SLC22A9 |
| hsa-mir-145-5p | TIRAP |
| hsa-mir-145-5p | RAB3IP |
| hsa-mir-145-5p | SLC16A10 |
| hsa-mir-145-5p | SP7 |
| hsa-mir-145-5p | CCDC43 |
| hsa-mir-145-5p | EID2B |
| hsa-mir-145-5p | UNC5D |
| hsa-mir-145-5p | SMIM17 |
| hsa-mir-145-5p | CCDC80 |
| hsa-mir-145-5p | C11orf65 |
| hsa-mir-145-5p | ZNF100 |
| hsa-mir-145-5p | UBXN2A |
| hsa-mir-145-5p | AAED1 |
| hsa-mir-145-5p | THSD7A |
| hsa-mir-145-5p | WASHC2C |
| hsa-mir-145-5p | BCLAF3 |
| hsa-mir-145-5p | SERINC5 |
| hsa-mir-145-5p | MUC19 |
| hsa-mir-145-5p | ZNF660 |
| hsa-mir-145-5p | TPRG1 |
| hsa-mir-145-5p | CCDC85C |
| hsa-mir-145-5p | ZNF678 |
| hsa-mir-145-5p | NUP43 |
| hsa-mir-145-5p | ZNF445 |
| hsa-mir-145-5p | SAMD5 |
| hsa-mir-145-5p | ZNF772 |
| hsa-mir-145-5p | FAM45A |
| hsa-mir-145-5p | CRNDE |
| hsa-mir-135b-5p | ACVR1B |
| hsa-mir-135b-5p | APC |
| hsa-mir-135b-5p | BIRC5 |
| hsa-mir-135b-5p | APOA1 |
| hsa-mir-135b-5p | AZF1 |
| hsa-mir-135b-5p | BGLAP |
| hsa-mir-135b-5p | BMPR2 |
| hsa-mir-135b-5p | CAPZA2 |
| hsa-mir-135b-5p | CASR |
| hsa-mir-135b-5p | RUNX2 |
| hsa-mir-135b-5p | CDR1 |
| hsa-mir-135b-5p | COX6B1 |
| hsa-mir-135b-5p | FOXO1 |
| hsa-mir-135b-5p | GAGE1 |
| hsa-mir-135b-5p | GATA6 |
| hsa-mir-135b-5p | GNL1 |
| hsa-mir-135b-5p | HMGB2 |
| hsa-mir-135b-5p | IBSP |
| hsa-mir-135b-5p | LDHA |
| hsa-mir-135b-5p | MARCKS |
| hsa-mir-135b-5p | SMAD5 |
| hsa-mir-135b-5p | MBNL1 |
| hsa-mir-135b-5p | MEF2C |
| hsa-mir-135b-5p | MID1 |
| hsa-mir-135b-5p | MPL |
| hsa-mir-135b-5p | MYC |
| hsa-mir-135b-5p | NHS |
| hsa-mir-135b-5p | POLH |
| hsa-mir-135b-5p | PPP2R5C |
| hsa-mir-135b-5p | PEX2 |
| hsa-mir-135b-5p | SKIL |
| hsa-mir-135b-5p | STAT6 |
| hsa-mir-135b-5p | TGFBR1 |
| hsa-mir-135b-5p | THBS2 |
| hsa-mir-135b-5p | TRAF6 |
| hsa-mir-135b-5p | XBP1P1 |
| hsa-mir-135b-5p | EVI5 |
| hsa-mir-135b-5p | ADAM12 |
| hsa-mir-135b-5p | PIP5K1A |
| hsa-mir-135b-5p | RECK |
| hsa-mir-135b-5p | KLF4 |
| hsa-mir-135b-5p | CEP135 |
| hsa-mir-135b-5p | TRIM66 |
| hsa-mir-135b-5p | TOX4 |
| hsa-mir-135b-5p | MAFB |
| hsa-mir-135b-5p | NSA2 |
| hsa-mir-135b-5p | TXNIP |
| hsa-mir-135b-5p | LZTS1 |
| hsa-mir-135b-5p | PPM1E |
| hsa-mir-135b-5p | ZNF609 |
| hsa-mir-135b-5p | ARC |
| hsa-mir-135b-5p | IL17RA |
| hsa-mir-135b-5p | MTCH2 |
| hsa-mir-135b-5p | RAB3GAP2 |
| hsa-mir-135b-5p | SLC39A6 |
| hsa-mir-135b-5p | SNED1 |
| hsa-mir-135b-5p | HEYL |
| hsa-mir-135b-5p | TNPO2 |
| hsa-mir-135b-5p | AMOTL2 |
| hsa-mir-135b-5p | ZNF107 |
| hsa-mir-135b-5p | PIAS4 |
| hsa-mir-135b-5p | DPP8 |
| hsa-mir-135b-5p | LAX1 |
| hsa-mir-135b-5p | VNN3 |
| hsa-mir-135b-5p | CENPN |
| hsa-mir-135b-5p | SCYL3 |
| hsa-mir-135b-5p | KIAA1143 |
| hsa-mir-135b-5p | NUFIP2 |
| hsa-mir-135b-5p | PCTP |
| hsa-mir-135b-5p | TTLL7 |
| hsa-mir-135b-5p | SLC19A3 |
| hsa-mir-135b-5p | TRIM4 |
| hsa-mir-135b-5p | ZNF468 |
| hsa-mir-135b-5p | SP7 |
| hsa-mir-135b-5p | LRRC15 |
| hsa-mir-135b-5p | KIAA1958 |
| hsa-mir-135b-5p | ZNF846 |
| hsa-mir-135b-5p | KIF6 |
| hsa-mir-135b-5p | ATXN7L1 |
| hsa-mir-135b-5p | CCDC85C |
| hsa-mir-135b-5p | ZNF805 |
| hsa-mir-135b-5p | PCP4L1 |
| hsa-mir-135b-5p | WDR82P1 |
