# Supplementary material for: Whole-transcriptome analysis reveals a potential hsa_circ_0001955/hsa_circ_0000977-mediated miRNA-mRNA regulatory sub-network in colorectal cancer
Source: Aging (Albany NY). 2020 Mar 28;12(6):5259–79. doi: 10.18632/aging.102945 (PMC7138558; doi:10.18632/aging.102945)
Supplement: Supplementary Table 5 [file aging-12-102945-s005..doc]

**Supplementary Table 5. Identification of differentially expressed genes (DEGs) between colorectal cancer tissues and normal tissues.**

| ID | adj.P.Val | t | B | logFC |
| --- | --- | --- | --- | --- |
| CUST_145122_PI430048170 | 9.76E-08 | 12.63391 | 15.22064 | 8.648813 |
| CUST_141792_PI430048170 | 1.55E-10 | 21.6716 | 23.94251 | 6.158618 |
| CUST_5377_PI430048170 | 3.43E-06 | 9.299367 | 10.2082 | 5.77126 |
| CUST_127586_PI430048170 | 5.03E-07 | 11.03094 | 12.97458 | 5.770365 |
| CUST_128358_PI430048170 | 1.89E-06 | 9.822987 | 11.08508 | 5.650322 |
| CUST_125137_PI430048170 | 1.59E-04 | 6.247141 | 4.336892 | 5.577447 |
| CUST_58761_PI430048170 | 3.74E-04 | 5.697976 | 3.145482 | 5.401148 |
| CUST_1488_PI430048170 | 1.43E-09 | 18.60499 | 21.57345 | 5.304836 |
| CUST_137929_PI430048170 | 8.90E-03 | 3.82212 | -1.09823 | 5.145209 |
| CUST_41403_PI430048170 | 3.89E-06 | 9.168508 | 9.983335 | 5.084703 |
| CUST_45401_PI430048170 | 3.50E-05 | 7.367575 | 6.645373 | 4.738569 |
| CUST_136618_PI430048170 | 6.32E-08 | 13.0061 | 15.70351 | 4.731195 |
| CUST_126853_PI430048170 | 2.47E-04 | 5.951459 | 3.699865 | 4.686458 |
| CUST_100650_PI430048170 | 3.68E-06 | 9.231753 | 10.09231 | 4.673326 |
| CUST_132940_PI430048170 | 4.73E-09 | 16.62732 | 19.76311 | 4.662882 |
| CUST_144827_PI430048170 | 1.46E-06 | 10.02857 | 11.41954 | 4.60254 |
| CUST_19675_PI430048170 | 6.81E-06 | 8.714336 | 9.184695 | 4.597025 |
| CUST_143790_PI430048170 | 1.92E-04 | 6.112464 | 4.048079 | 4.555178 |
| CUST_20154_PI430048170 | 1.10E-03 | 5.033361 | 1.661127 | 4.510617 |
| CUST_48179_PI430048170 | 7.03E-05 | 6.829118 | 5.557601 | 4.487417 |
| CUST_143718_PI430048170 | 1.80E-04 | 6.162671 | 4.156014 | 4.446267 |
| CUST_134531_PI430048170 | 2.32E-06 | 9.659717 | 10.81555 | 4.420711 |
| CUST_128936_PI430048170 | 1.34E-04 | 6.363101 | 4.583711 | 4.410234 |
| CUST_140303_PI430048170 | 1.32E-06 | 10.14406 | 11.60506 | 4.349447 |
| CUST_57830_PI430048170 | 3.14E-06 | 9.380836 | 10.34703 | 4.347582 |
| CUST_134217_PI430048170 | 5.57E-05 | 7.011324 | 5.930269 | 4.33215 |
| CUST_69924_PI430048170 | 3.28E-03 | 4.393845 | 0.205075 | 4.235897 |
| CUST_142934_PI430048170 | 1.47E-06 | 10.01031 | 11.39005 | 4.226445 |
| CUST_133468_PI430048170 | 1.04E-05 | 8.314178 | 8.457197 | 4.206495 |
| CUST_71878_PI430048170 | 2.56E-03 | 4.536347 | 0.530695 | 4.14868 |
| CUST_82133_PI430048170 | 2.90E-08 | 14.0214 | 16.95338 | 4.121411 |
| CUST_50659_PI430048170 | 6.76E-04 | 5.327537 | 2.32303 | 4.094232 |
| CUST_134948_PI430048170 | 3.47E-06 | 9.286302 | 10.18586 | 4.086924 |
| CUST_132589_PI430048170 | 1.32E-05 | 8.127919 | 8.110836 | 4.033176 |
| CUST_122265_PI430048170 | 3.43E-06 | 9.299274 | 10.20804 | 3.981719 |
| CUST_81682_PI430048170 | 5.30E-09 | 16.36625 | 19.50485 | 3.953427 |
| CUST_97232_PI430048170 | 4.69E-08 | 13.38294 | 16.17864 | 3.895768 |
| CUST_139824_PI430048170 | 8.06E-06 | 8.58078 | 8.944394 | 3.858889 |
| CUST_70631_PI430048170 | 3.43E-04 | 5.75477 | 3.270323 | 3.83258 |
| CUST_20074_PI430048170 | 1.61E-05 | 7.967193 | 7.807974 | 3.781498 |
| CUST_140877_PI430048170 | 7.83E-08 | 12.83069 | 15.47765 | 3.759283 |
| CUST_72198_PI430048170 | 8.57E-06 | 8.506887 | 8.810365 | 3.73836 |
| CUST_45943_PI430048170 | 8.66E-03 | 3.837275 | -1.06389 | 3.736866 |
| CUST_111850_PI430048170 | 1.90E-08 | 14.7807 | 17.82796 | 3.699909 |
| CUST_41044_PI430048170 | 1.26E-02 | 3.615825 | -1.56358 | 3.697924 |
| CUST_67818_PI430048170 | 6.61E-06 | 8.738196 | 9.227363 | 3.695375 |
| CUST_121083_PI430048170 | 1.19E-05 | 8.202135 | 8.249438 | 3.671743 |
| CUST_81681_PI430048170 | 3.67E-08 | 13.64645 | 16.50292 | 3.658176 |
| CUST_137495_PI430048170 | 9.79E-05 | 6.586752 | 5.054764 | 3.657072 |
| CUST_20036_PI430048170 | 2.67E-08 | 14.26891 | 17.24391 | 3.636063 |
| CUST_110351_PI430048170 | 1.28E-03 | 4.942429 | 1.455251 | 3.620105 |
| CUST_92499_PI430048170 | 2.22E-04 | 6.015617 | 3.838999 | 3.620065 |
| CUST_136041_PI430048170 | 1.86E-04 | 6.13708 | 4.101038 | 3.589806 |
| CUST_60218_PI430048170 | 1.38E-05 | 8.097493 | 8.053787 | 3.574255 |
| CUST_140466_PI430048170 | 3.04E-07 | 11.56093 | 13.74847 | 3.549184 |
| CUST_27833_PI430048170 | 3.93E-05 | 7.276173 | 6.463623 | 3.540254 |
| CUST_92138_PI430048170 | 7.35E-05 | 6.795789 | 5.488932 | 3.533655 |
| CUST_107270_PI430048170 | 6.68E-04 | 5.336071 | 2.342124 | 3.513335 |
| CUST_68706_PI430048170 | 1.65E-04 | 6.220486 | 4.279913 | 3.507298 |
| CUST_127726_PI430048170 | 1.95E-05 | 7.833194 | 7.552642 | 3.497683 |
| CUST_102074_PI430048170 | 7.16E-05 | 6.814277 | 5.527042 | 3.482274 |
| CUST_7623_PI430048170 | 1.93E-03 | 4.70423 | 0.913744 | 3.474125 |
| CUST_112277_PI430048170 | 2.71E-03 | 4.502372 | 0.453087 | 3.454322 |
| CUST_123662_PI430048170 | 1.36E-04 | 6.350144 | 4.55622 | 3.420465 |
| CUST_76304_PI430048170 | 2.52E-03 | 4.545644 | 0.551928 | 3.418697 |
| CUST_124944_PI430048170 | 1.03E-04 | 6.551341 | 4.980627 | 3.416949 |
| CUST_5123_PI430048170 | 9.16E-04 | 5.144265 | 1.911457 | 3.41316 |
| CUST_7624_PI430048170 | 7.60E-04 | 5.257016 | 2.164991 | 3.411908 |
| CUST_69978_PI430048170 | 1.49E-05 | 8.040486 | 7.946542 | 3.394338 |
| CUST_125385_PI430048170 | 6.68E-03 | 3.984686 | -0.72901 | 3.391724 |
| CUST_105411_PI430048170 | 2.71E-05 | 7.561271 | 7.026575 | 3.38821 |
| CUST_25879_PI430048170 | 1.23E-02 | 3.630604 | -1.53039 | 3.387689 |
| CUST_45195_PI430048170 | 1.33E-06 | 10.10991 | 11.55037 | 3.359769 |
| CUST_137163_PI430048170 | 2.46E-04 | 5.955649 | 3.708968 | 3.34695 |
| CUST_134906_PI430048170 | 6.89E-04 | 5.316318 | 2.297916 | 3.34584 |
| CUST_29609_PI430048170 | 3.10E-04 | 5.815233 | 3.402838 | 3.335935 |
| CUST_66580_PI430048170 | 1.06E-03 | 5.057431 | 1.715532 | 3.33413 |
| CUST_49297_PI430048170 | 3.19E-02 | 3.066855 | -2.77217 | 3.33283 |
| CUST_84309_PI430048170 | 6.04E-05 | 6.952803 | 5.811081 | 3.331936 |
| CUST_20748_PI430048170 | 5.54E-03 | 4.092469 | -0.4834 | 3.321899 |
| CUST_142518_PI430048170 | 1.49E-06 | 9.997543 | 11.36941 | 3.303385 |
| CUST_129341_PI430048170 | 4.45E-02 | 2.861275 | -3.20802 | 3.301457 |
| CUST_5124_PI430048170 | 8.27E-04 | 5.205313 | 2.048857 | 3.278397 |
| CUST_71805_PI430048170 | 1.14E-05 | 8.241263 | 8.322196 | 3.277207 |
| CUST_133568_PI430048170 | 2.53E-03 | 4.543312 | 0.546602 | 3.27437 |
| CUST_145267_PI430048170 | 4.08E-03 | 4.268668 | -0.08104 | 3.270856 |
| CUST_127454_PI430048170 | 4.39E-04 | 5.598423 | 2.92581 | 3.268833 |
| CUST_139024_PI430048170 | 6.21E-05 | 6.928773 | 5.762003 | 3.268174 |
| CUST_55363_PI430048170 | 1.37E-03 | 4.905502 | 1.3715 | 3.258474 |
| CUST_132565_PI430048170 | 6.52E-04 | 5.352 | 2.37775 | 3.247702 |
| CUST_129687_PI430048170 | 1.06E-02 | 3.717259 | -1.3353 | 3.245902 |
| CUST_124868_PI430048170 | 2.06E-05 | 7.789553 | 7.46893 | 3.245631 |
| CUST_113534_PI430048170 | 1.05E-04 | 6.534012 | 4.944285 | 3.238699 |
| CUST_49672_PI430048170 | 1.62E-04 | 6.233292 | 4.307299 | 3.216257 |
| CUST_96116_PI430048170 | 1.04E-02 | 3.731486 | -1.30319 | 3.205384 |
| CUST_129518_PI430048170 | 1.84E-03 | 4.733242 | 0.979842 | 3.201178 |
| CUST_58310_PI430048170 | 1.39E-05 | 8.09208 | 8.043623 | 3.199597 |
| CUST_140326_PI430048170 | 6.05E-04 | 5.396614 | 2.477403 | 3.197943 |
| CUST_127560_PI430048170 | 2.29E-05 | 7.703243 | 7.302562 | 3.166799 |
| CUST_28409_PI430048170 | 4.52E-02 | 2.850349 | -3.23085 | 3.146458 |
| CUST_58103_PI430048170 | 4.04E-04 | 5.646502 | 3.032031 | 3.143517 |
| CUST_141790_PI430048170 | 8.80E-03 | 3.828958 | -1.08273 | 3.140951 |
| CUST_140833_PI430048170 | 5.33E-05 | 7.043435 | 5.995463 | 3.137356 |
| CUST_104053_PI430048170 | 7.86E-04 | 5.237357 | 2.120859 | 3.137089 |
| CUST_139461_PI430048170 | 3.01E-03 | 4.44106 | 0.312989 | 3.115906 |
| CUST_137932_PI430048170 | 1.53E-03 | 4.839318 | 1.221198 | 3.108665 |
| CUST_78373_PI430048170 | 6.50E-05 | 6.883418 | 5.669146 | 3.10224 |
| CUST_86621_PI430048170 | 4.38E-05 | 7.197115 | 6.305458 | 3.09764 |
| CUST_70630_PI430048170 | 4.83E-05 | 7.12227 | 6.154903 | 3.090159 |
| CUST_117853_PI430048170 | 1.32E-03 | 4.926426 | 1.418966 | 3.087489 |
| CUST_109573_PI430048170 | 6.26E-03 | 4.023471 | -0.64069 | 3.086201 |
| CUST_16517_PI430048170 | 2.52E-05 | 7.619432 | 7.139987 | 3.0836 |
| CUST_127557_PI430048170 | 1.46E-04 | 6.299727 | 4.449035 | 3.077392 |
| CUST_37928_PI430048170 | 1.56E-07 | 12.20126 | 14.64171 | 3.077285 |
| CUST_16366_PI430048170 | 3.27E-06 | 9.33351 | 10.26649 | 3.072858 |
| CUST_60643_PI430048170 | 6.66E-06 | 8.729999 | 9.212713 | 3.066653 |
| CUST_134644_PI430048170 | 2.81E-04 | 5.873179 | 3.529445 | 3.061192 |
| CUST_144905_PI430048170 | 2.45E-03 | 4.56064 | 0.586173 | 3.060895 |
| CUST_143267_PI430048170 | 8.94E-04 | 5.158091 | 1.9426 | 3.057842 |
| CUST_48039_PI430048170 | 7.46E-05 | 6.785853 | 5.468432 | 3.054591 |
| CUST_134859_PI430048170 | 2.29E-04 | 5.997681 | 3.800154 | 3.054402 |
| CUST_34332_PI430048170 | 1.33E-03 | 4.919367 | 1.402955 | 3.050129 |
| CUST_60205_PI430048170 | 3.81E-05 | 7.296859 | 6.50486 | 3.038306 |
| CUST_29298_PI430048170 | 3.79E-04 | 5.688315 | 3.124209 | 3.020219 |
| CUST_144691_PI430048170 | 3.61E-08 | 13.77616 | 16.66019 | 3.011451 |
| CUST_127495_PI430048170 | 1.32E-03 | 4.924012 | 1.413491 | 3.00605 |
| CUST_91835_PI430048170 | 3.66E-07 | 11.3945 | 13.50891 | 3.005414 |
| CUST_130371_PI430048170 | 2.57E-03 | 4.533362 | 0.523878 | 3.003824 |
| CUST_12823_PI430048170 | 1.52E-02 | 3.506273 | -1.80875 | 2.999312 |
| CUST_112407_PI430048170 | 4.82E-04 | 5.539466 | 2.79523 | 2.988086 |
| CUST_63112_PI430048170 | 3.61E-08 | 13.74346 | 16.62068 | 2.97261 |
| CUST_95859_PI430048170 | 5.97E-07 | 10.88455 | 12.75508 | 2.969767 |
| CUST_113415_PI430048170 | 2.04E-03 | 4.668661 | 0.832664 | 2.966329 |
| CUST_137850_PI430048170 | 6.14E-04 | 5.387962 | 2.458092 | 2.96344 |
| CUST_84214_PI430048170 | 6.57E-04 | 5.347785 | 2.368323 | 2.962905 |
| CUST_138735_PI430048170 | 4.43E-04 | 5.593143 | 2.91413 | 2.960599 |
| CUST_144194_PI430048170 | 8.38E-05 | 6.703283 | 5.297536 | 2.960452 |
| CUST_17641_PI430048170 | 3.35E-03 | 4.380168 | 0.173811 | 2.948147 |
| CUST_129229_PI430048170 | 1.77E-03 | 4.753581 | 1.026161 | 2.945715 |
| CUST_135152_PI430048170 | 2.09E-02 | 3.318037 | -2.22589 | 2.941752 |
| CUST_139845_PI430048170 | 5.68E-04 | 5.438393 | 2.570559 | 2.941107 |
| CUST_135976_PI430048170 | 5.68E-06 | 8.862661 | 9.448651 | 2.940865 |
| CUST_79252_PI430048170 | 1.30E-03 | 4.936438 | 1.44167 | 2.938321 |
| CUST_116320_PI430048170 | 5.05E-03 | 4.144584 | -0.36448 | 2.932555 |
| CUST_108441_PI430048170 | 1.13E-05 | 8.246975 | 8.332798 | 2.927885 |
| CUST_7444_PI430048170 | 5.76E-04 | 5.429301 | 2.550301 | 2.927223 |
| CUST_135446_PI430048170 | 3.61E-03 | 4.337328 | 0.075888 | 2.920945 |
| CUST_51014_PI430048170 | 4.33E-03 | 4.23418 | -0.15985 | 2.908224 |
| CUST_143141_PI430048170 | 1.86E-03 | 4.725699 | 0.962661 | 2.904236 |
| CUST_60214_PI430048170 | 6.78E-07 | 10.77382 | 12.58737 | 2.904173 |
| CUST_47187_PI430048170 | 5.18E-07 | 11.00485 | 12.93565 | 2.900274 |
| CUST_127137_PI430048170 | 1.70E-03 | 4.777146 | 1.079801 | 2.899697 |
| CUST_80802_PI430048170 | 3.47E-03 | 4.361269 | 0.130612 | 2.898121 |
| CUST_89175_PI430048170 | 3.02E-03 | 4.439744 | 0.309981 | 2.893758 |
| CUST_140468_PI430048170 | 1.22E-02 | 3.636147 | -1.51793 | 2.87277 |
| CUST_29299_PI430048170 | 6.46E-04 | 5.357309 | 2.389617 | 2.871235 |
| CUST_139647_PI430048170 | 7.32E-03 | 3.931658 | -0.84964 | 2.870573 |
| CUST_137546_PI430048170 | 1.89E-02 | 3.380394 | -2.08835 | 2.870177 |
| CUST_145203_PI430048170 | 9.90E-03 | 3.758417 | -1.24237 | 2.864499 |
| CUST_103399_PI430048170 | 4.68E-02 | 2.827005 | -3.27951 | 2.863375 |
| CUST_127972_PI430048170 | 1.39E-02 | 3.561217 | -1.68598 | 2.863241 |
| CUST_54090_PI430048170 | 2.35E-04 | 5.982001 | 3.766161 | 2.862281 |
| CUST_55030_PI430048170 | 9.21E-04 | 5.140374 | 1.902688 | 2.86212 |
| CUST_130696_PI430048170 | 8.80E-06 | 8.480557 | 8.762422 | 2.860449 |
| CUST_92828_PI430048170 | 1.10E-04 | 6.504275 | 4.881828 | 2.853922 |
| CUST_139040_PI430048170 | 2.20E-04 | 6.021071 | 3.850805 | 2.851486 |
| CUST_135652_PI430048170 | 2.99E-03 | 4.444567 | 0.321003 | 2.848892 |
| CUST_135595_PI430048170 | 6.23E-05 | 6.924122 | 5.752493 | 2.846425 |
| CUST_29395_PI430048170 | 1.53E-04 | 6.267475 | 4.380298 | 2.844868 |
| CUST_119941_PI430048170 | 2.26E-05 | 7.720789 | 7.336469 | 2.840691 |
| CUST_21458_PI430048170 | 3.48E-06 | 9.282403 | 10.17918 | 2.840687 |
| CUST_134352_PI430048170 | 2.41E-03 | 4.57048 | 0.60864 | 2.840389 |
| CUST_135248_PI430048170 | 3.09E-03 | 4.425799 | 0.27811 | 2.835993 |
| CUST_129073_PI430048170 | 4.34E-04 | 5.605372 | 2.941178 | 2.833508 |
| CUST_122174_PI430048170 | 1.04E-02 | 3.728685 | -1.30951 | 2.820211 |
| CUST_131701_PI430048170 | 1.13E-03 | 5.01731 | 1.624827 | 2.817661 |
| CUST_109162_PI430048170 | 1.14E-04 | 6.476534 | 4.823455 | 2.807408 |
| CUST_96236_PI430048170 | 2.69E-07 | 11.69012 | 13.93229 | 2.806258 |
| CUST_140502_PI430048170 | 1.09E-02 | 3.702 | -1.36971 | 2.800893 |
| CUST_101425_PI430048170 | 3.93E-05 | 7.276977 | 6.465226 | 2.78743 |
| CUST_116276_PI430048170 | 4.44E-05 | 7.185615 | 6.282377 | 2.785957 |
| CUST_21389_PI430048170 | 1.42E-03 | 4.881212 | 1.316365 | 2.785195 |
| CUST_25883_PI430048170 | 2.66E-02 | 3.175483 | -2.53758 | 2.779015 |
| CUST_132956_PI430048170 | 2.48E-03 | 4.55297 | 0.568658 | 2.773838 |
| CUST_75480_PI430048170 | 8.78E-06 | 8.482939 | 8.766762 | 2.771338 |
| CUST_119935_PI430048170 | 6.46E-05 | 6.890654 | 5.683981 | 2.769421 |
| CUST_137291_PI430048170 | 1.10E-03 | 5.035363 | 1.665654 | 2.768026 |
| CUST_91806_PI430048170 | 2.11E-02 | 3.312771 | -2.23747 | 2.767409 |
| CUST_134340_PI430048170 | 4.70E-06 | 9.021044 | 9.727141 | 2.766763 |
| CUST_63721_PI430048170 | 3.89E-02 | 2.945745 | -3.03033 | 2.765017 |
| CUST_50535_PI430048170 | 2.28E-05 | 7.710244 | 7.316097 | 2.764982 |
| CUST_134954_PI430048170 | 8.25E-06 | 8.539738 | 8.870046 | 2.759055 |
| CUST_52844_PI430048170 | 4.63E-04 | 5.5671 | 2.856477 | 2.749527 |
| CUST_127599_PI430048170 | 2.75E-03 | 4.493229 | 0.4322 | 2.743314 |
| CUST_138546_PI430048170 | 3.68E-03 | 4.327182 | 0.052695 | 2.738247 |
| CUST_30965_PI430048170 | 4.29E-05 | 7.213463 | 6.338236 | 2.72849 |
| CUST_16280_PI430048170 | 3.89E-02 | 2.945735 | -3.03035 | 2.711681 |
| CUST_60253_PI430048170 | 2.49E-05 | 7.629889 | 7.160326 | 2.709631 |
| CUST_20833_PI430048170 | 3.51E-03 | 4.354616 | 0.115405 | 2.704141 |
| CUST_67630_PI430048170 | 3.51E-04 | 5.740884 | 3.239832 | 2.703209 |
| CUST_120073_PI430048170 | 2.13E-04 | 6.0416 | 3.895206 | 2.703149 |
| CUST_49460_PI430048170 | 7.22E-06 | 8.66966 | 9.10459 | 2.701374 |
| CUST_133940_PI430048170 | 1.09E-03 | 5.039335 | 1.674635 | 2.696516 |
| CUST_115797_PI430048170 | 5.39E-05 | 7.033508 | 5.975324 | 2.687997 |
| CUST_91397_PI430048170 | 1.82E-04 | 6.156775 | 4.143355 | 2.677276 |
| CUST_90646_PI430048170 | 1.57E-04 | 6.252207 | 4.347709 | 2.675953 |
| CUST_21432_PI430048170 | 1.12E-03 | 5.025073 | 1.642385 | 2.66958 |
| CUST_99489_PI430048170 | 7.27E-05 | 6.803018 | 5.503839 | 2.668855 |
| CUST_67126_PI430048170 | 4.68E-05 | 7.143345 | 6.197378 | 2.667922 |
| CUST_132164_PI430048170 | 8.39E-03 | 3.854208 | -1.0255 | 2.667597 |
| CUST_103483_PI430048170 | 1.13E-05 | 8.245313 | 8.329713 | 2.665575 |
| CUST_119971_PI430048170 | 2.01E-03 | 4.676518 | 0.850577 | 2.658083 |
| CUST_18357_PI430048170 | 8.52E-04 | 5.188093 | 2.010129 | 2.656202 |
| CUST_136688_PI430048170 | 1.42E-05 | 8.076091 | 8.013578 | 2.653987 |
| CUST_85203_PI430048170 | 1.40E-05 | 8.084879 | 8.030096 | 2.653966 |
| CUST_142975_PI430048170 | 1.46E-02 | 3.53266 | -1.74984 | 2.65044 |
| CUST_144014_PI430048170 | 1.03E-04 | 6.553778 | 4.985735 | 2.649349 |
| CUST_126585_PI430048170 | 7.32E-06 | 8.659802 | 9.086877 | 2.649338 |
| CUST_60212_PI430048170 | 3.76E-05 | 7.305881 | 6.522826 | 2.645849 |
| CUST_10680_PI430048170 | 3.04E-04 | 5.824821 | 3.423814 | 2.642996 |
| CUST_77467_PI430048170 | 9.48E-04 | 5.124915 | 1.867845 | 2.630121 |
| CUST_132902_PI430048170 | 3.45E-04 | 5.751626 | 3.263421 | 2.629951 |
| CUST_115075_PI430048170 | 1.94E-04 | 6.102177 | 4.025925 | 2.617453 |
| CUST_84236_PI430048170 | 1.89E-04 | 6.12175 | 4.068066 | 2.610407 |
| CUST_22087_PI430048170 | 1.17E-03 | 4.999528 | 1.584589 | 2.597788 |
| CUST_137490_PI430048170 | 2.29E-02 | 3.264079 | -2.34434 | 2.597775 |
| CUST_120426_PI430048170 | 4.89E-03 | 4.164263 | -0.31955 | 2.594688 |
| CUST_60193_PI430048170 | 3.29E-05 | 7.423521 | 6.756028 | 2.590241 |
| CUST_77466_PI430048170 | 1.28E-03 | 4.943727 | 1.458194 | 2.585227 |
| CUST_116304_PI430048170 | 8.99E-06 | 8.439788 | 8.687993 | 2.584892 |
| CUST_128776_PI430048170 | 7.25E-05 | 6.805679 | 5.509326 | 2.583248 |
| CUST_42418_PI430048170 | 2.04E-02 | 3.33284 | -2.1933 | 2.58191 |
| CUST_133071_PI430048170 | 1.25E-03 | 4.956804 | 1.487832 | 2.5793 |
| CUST_94319_PI430048170 | 1.08E-05 | 8.2863 | 8.405671 | 2.578801 |
| CUST_138116_PI430048170 | 5.62E-03 | 4.083878 | -0.503 | 2.56085 |
| CUST_19238_PI430048170 | 2.19E-04 | 6.023136 | 3.855272 | 2.559868 |
| CUST_132834_PI430048170 | 2.84E-03 | 4.475016 | 0.390585 | 2.559053 |
| CUST_119649_PI430048170 | 1.38E-06 | 10.08004 | 11.50243 | 2.557651 |
| CUST_123570_PI430048170 | 6.10E-03 | 4.038325 | -0.60685 | 2.554627 |
| CUST_121440_PI430048170 | 5.64E-03 | 4.081916 | -0.50747 | 2.54965 |
| CUST_134665_PI430048170 | 3.10E-02 | 3.083631 | -2.73612 | 2.545227 |
| CUST_130493_PI430048170 | 1.95E-04 | 6.096561 | 4.013825 | 2.545212 |
| CUST_118992_PI430048170 | 1.51E-03 | 4.846759 | 1.238108 | 2.544999 |
| CUST_29802_PI430048170 | 2.31E-07 | 11.83263 | 14.13292 | 2.542523 |
| CUST_133884_PI430048170 | 3.33E-05 | 7.416701 | 6.742562 | 2.538403 |
| CUST_126748_PI430048170 | 3.95E-03 | 4.28652 | -0.04024 | 2.537351 |
| CUST_130405_PI430048170 | 5.01E-04 | 5.514877 | 2.740663 | 2.536121 |
| CUST_131141_PI430048170 | 6.26E-03 | 4.022916 | -0.64196 | 2.532399 |
| CUST_62029_PI430048170 | 2.96E-03 | 4.450933 | 0.335553 | 2.531176 |
| CUST_94794_PI430048170 | 2.36E-02 | 3.246284 | -2.38328 | 2.529769 |
| CUST_142050_PI430048170 | 1.49E-07 | 12.30211 | 14.77839 | 2.525305 |
| CUST_135319_PI430048170 | 9.70E-03 | 3.77077 | -1.21444 | 2.522036 |
| CUST_6366_PI430048170 | 2.75E-07 | 11.64972 | 13.875 | 2.519581 |
| CUST_132598_PI430048170 | 4.06E-02 | 2.918518 | -3.08782 | 2.51792 |
| CUST_136732_PI430048170 | 2.65E-04 | 5.90705 | 3.603273 | 2.512742 |
| CUST_141504_PI430048170 | 1.75E-04 | 6.182658 | 4.198894 | 2.512385 |
| CUST_87647_PI430048170 | 1.09E-02 | 3.70092 | -1.37214 | 2.512137 |
| CUST_19326_PI430048170 | 4.78E-04 | 5.547028 | 2.811996 | 2.511935 |
| CUST_54150_PI430048170 | 8.28E-03 | 3.861871 | -1.00812 | 2.510033 |
| CUST_98433_PI430048170 | 1.19E-05 | 8.196614 | 8.239155 | 2.50965 |
| CUST_26938_PI430048170 | 1.39E-02 | 3.560897 | -1.6867 | 2.509231 |
| CUST_143169_PI430048170 | 2.62E-03 | 4.521843 | 0.497567 | 2.509182 |
| CUST_7862_PI430048170 | 1.56E-07 | 12.20515 | 14.64701 | 2.507485 |
| CUST_17747_PI430048170 | 2.65E-03 | 4.514045 | 0.479755 | 2.506591 |
| CUST_38069_PI430048170 | 2.32E-02 | 3.25673 | -2.36043 | 2.505916 |
| CUST_49529_PI430048170 | 3.31E-02 | 3.044933 | -2.81918 | 2.494721 |
| CUST_141499_PI430048170 | 1.80E-03 | 4.743384 | 1.00294 | 2.488484 |
| CUST_126659_PI430048170 | 5.55E-03 | 4.091432 | -0.48577 | 2.485715 |
| CUST_131715_PI430048170 | 2.57E-03 | 4.534143 | 0.525663 | 2.483314 |
| CUST_23969_PI430048170 | 1.82E-04 | 6.152879 | 4.134988 | 2.47882 |
| CUST_138691_PI430048170 | 8.75E-03 | 3.831887 | -1.0761 | 2.478401 |
| CUST_140236_PI430048170 | 6.17E-03 | 4.030747 | -0.62412 | 2.478254 |
| CUST_118529_PI430048170 | 6.61E-03 | 3.990905 | -0.71486 | 2.47778 |
| CUST_141614_PI430048170 | 4.03E-04 | 5.648147 | 3.035661 | 2.468506 |
| CUST_132774_PI430048170 | 7.39E-04 | 5.274092 | 2.203298 | 2.467811 |
| CUST_112545_PI430048170 | 1.44E-05 | 8.064363 | 7.991517 | 2.461924 |
| CUST_142986_PI430048170 | 1.01E-03 | 5.086738 | 1.781719 | 2.461437 |
| CUST_80908_PI430048170 | 9.30E-06 | 8.408494 | 8.630702 | 2.461131 |
| CUST_140640_PI430048170 | 9.24E-04 | 5.138693 | 1.898902 | 2.458828 |
| CUST_130916_PI430048170 | 4.19E-04 | 5.626331 | 2.987496 | 2.455062 |
| CUST_120041_PI430048170 | 5.55E-04 | 5.454463 | 2.606348 | 2.448471 |
| CUST_129095_PI430048170 | 1.96E-03 | 4.693195 | 0.888594 | 2.446577 |
| CUST_103897_PI430048170 | 1.04E-04 | 6.54348 | 4.964146 | 2.446115 |
| CUST_115550_PI430048170 | 1.64E-03 | 4.799576 | 1.130835 | 2.445616 |
| CUST_140424_PI430048170 | 3.78E-04 | 5.68997 | 3.127855 | 2.442741 |
| CUST_118339_PI430048170 | 8.20E-04 | 5.209967 | 2.05932 | 2.440609 |
| CUST_124586_PI430048170 | 4.26E-02 | 2.888338 | -3.15131 | 2.43987 |
| CUST_74385_PI430048170 | 1.26E-02 | 3.617946 | -1.55882 | 2.437682 |
| CUST_78996_PI430048170 | 8.22E-05 | 6.719528 | 5.331232 | 2.437631 |
| CUST_142575_PI430048170 | 1.71E-04 | 6.196809 | 4.229223 | 2.437401 |
| CUST_98751_PI430048170 | 5.27E-03 | 4.119777 | -0.4211 | 2.437013 |
| CUST_21454_PI430048170 | 6.49E-05 | 6.887014 | 5.67652 | 2.436527 |
| CUST_130673_PI430048170 | 3.83E-07 | 11.35547 | 13.45226 | 2.431356 |
| CUST_126697_PI430048170 | 9.89E-03 | 3.759496 | -1.23993 | 2.431305 |
| CUST_119945_PI430048170 | 7.64E-05 | 6.768126 | 5.431821 | 2.428152 |
| CUST_52897_PI430048170 | 6.82E-03 | 3.972694 | -0.75631 | 2.426748 |
| CUST_60211_PI430048170 | 5.35E-05 | 7.038981 | 5.98643 | 2.421742 |
| CUST_126421_PI430048170 | 5.98E-03 | 4.048711 | -0.58318 | 2.421252 |
| CUST_7894_PI430048170 | 2.26E-05 | 7.71831 | 7.331682 | 2.420542 |
| CUST_136698_PI430048170 | 7.64E-05 | 6.769381 | 5.434414 | 2.416446 |
| CUST_82353_PI430048170 | 2.16E-02 | 3.300527 | -2.26439 | 2.416272 |
| CUST_134277_PI430048170 | 7.81E-04 | 5.241448 | 2.130046 | 2.415084 |
| CUST_88592_PI430048170 | 1.19E-04 | 6.445911 | 4.7589 | 2.414305 |
| CUST_121544_PI430048170 | 7.40E-03 | 3.926322 | -0.86177 | 2.413635 |
| CUST_106550_PI430048170 | 2.47E-04 | 5.951959 | 3.700951 | 2.412028 |
| CUST_27654_PI430048170 | 1.49E-03 | 4.857176 | 1.261775 | 2.410906 |
| CUST_111309_PI430048170 | 1.18E-03 | 4.992242 | 1.568098 | 2.40922 |
| CUST_127205_PI430048170 | 8.66E-03 | 3.837364 | -1.06368 | 2.407513 |
| CUST_39311_PI430048170 | 2.27E-03 | 4.604835 | 0.687062 | 2.406134 |
| CUST_132642_PI430048170 | 7.50E-05 | 6.78264 | 5.461799 | 2.403732 |
| CUST_136691_PI430048170 | 3.91E-02 | 2.942715 | -3.03674 | 2.394423 |
| CUST_136017_PI430048170 | 4.42E-04 | 5.59513 | 2.918525 | 2.393546 |
| CUST_62679_PI430048170 | 8.62E-05 | 6.684726 | 5.259 | 2.392603 |
| CUST_128635_PI430048170 | 9.70E-04 | 5.110854 | 1.836136 | 2.387886 |
| CUST_144841_PI430048170 | 2.38E-04 | 5.974961 | 3.75089 | 2.382748 |
| CUST_19537_PI430048170 | 5.08E-06 | 8.949372 | 9.601547 | 2.380195 |
| CUST_138593_PI430048170 | 1.05E-04 | 6.536042 | 4.948545 | 2.374767 |
| CUST_104592_PI430048170 | 5.01E-04 | 5.515897 | 2.742928 | 2.374364 |
| CUST_142767_PI430048170 | 6.66E-05 | 6.866479 | 5.634393 | 2.37318 |
| CUST_137737_PI430048170 | 2.41E-03 | 4.570594 | 0.6089 | 2.371947 |
| CUST_126845_PI430048170 | 6.73E-03 | 3.980608 | -0.7383 | 2.366456 |
| CUST_124335_PI430048170 | 2.69E-02 | 3.169086 | -2.55146 | 2.365997 |
| CUST_28673_PI430048170 | 1.52E-04 | 6.273222 | 4.392556 | 2.363041 |
| CUST_135571_PI430048170 | 8.07E-05 | 6.730831 | 5.354658 | 2.36085 |
| CUST_68282_PI430048170 | 9.08E-03 | 3.810842 | -1.12377 | 2.359351 |
| CUST_68215_PI430048170 | 2.88E-04 | 5.857269 | 3.494721 | 2.358795 |
| CUST_103598_PI430048170 | 1.45E-03 | 4.871913 | 1.295251 | 2.357979 |
| CUST_129072_PI430048170 | 9.02E-04 | 5.153077 | 1.931308 | 2.353605 |
| CUST_138058_PI430048170 | 2.20E-03 | 4.623385 | 0.729392 | 2.347421 |
| CUST_132798_PI430048170 | 7.06E-03 | 3.953136 | -0.8008 | 2.346428 |
| CUST_10466_PI430048170 | 1.09E-03 | 5.038551 | 1.672861 | 2.345274 |
| CUST_137843_PI430048170 | 4.36E-04 | 5.602918 | 2.935751 | 2.344712 |
| CUST_69249_PI430048170 | 1.49E-03 | 4.854876 | 1.256552 | 2.341986 |
| CUST_48222_PI430048170 | 2.00E-06 | 9.783088 | 11.01953 | 2.341891 |
| CUST_137823_PI430048170 | 9.83E-04 | 5.102399 | 1.817062 | 2.338086 |
| CUST_72543_PI430048170 | 1.28E-03 | 4.944051 | 1.458928 | 2.337561 |
| CUST_873_PI430048170 | 1.01E-02 | 3.748408 | -1.26498 | 2.336976 |
| CUST_98807_PI430048170 | 1.04E-05 | 8.315399 | 8.459452 | 2.33683 |
| CUST_136671_PI430048170 | 1.26E-04 | 6.402507 | 4.667189 | 2.336411 |
| CUST_97152_PI430048170 | 1.50E-02 | 3.516242 | -1.7865 | 2.334984 |
| CUST_133524_PI430048170 | 3.33E-02 | 3.041173 | -2.82723 | 2.333021 |
| CUST_139490_PI430048170 | 3.81E-03 | 4.307439 | 0.007569 | 2.331025 |
| CUST_51074_PI430048170 | 3.19E-04 | 5.798949 | 3.367188 | 2.328049 |
| CUST_128775_PI430048170 | 2.48E-02 | 3.216562 | -2.44818 | 2.324054 |
| CUST_60931_PI430048170 | 4.75E-04 | 5.550607 | 2.819931 | 2.321922 |
| CUST_100788_PI430048170 | 6.23E-05 | 6.92146 | 5.747049 | 2.321746 |
| CUST_71244_PI430048170 | 1.03E-02 | 3.733437 | -1.29879 | 2.318903 |
| CUST_130460_PI430048170 | 5.45E-04 | 5.465801 | 2.631582 | 2.318239 |
| CUST_56683_PI430048170 | 1.17E-03 | 4.999942 | 1.585528 | 2.316932 |
| CUST_129276_PI430048170 | 7.73E-06 | 8.613283 | 9.003106 | 2.310386 |
| CUST_40827_PI430048170 | 3.31E-03 | 4.387824 | 0.191311 | 2.308012 |
| CUST_12811_PI430048170 | 5.17E-04 | 5.494756 | 2.695969 | 2.306937 |
| CUST_132648_PI430048170 | 7.74E-03 | 3.900327 | -0.92083 | 2.306255 |
| CUST_59898_PI430048170 | 1.04E-04 | 6.543591 | 4.964379 | 2.293445 |
| CUST_133270_PI430048170 | 2.35E-03 | 4.585842 | 0.643711 | 2.293419 |
| CUST_122118_PI430048170 | 4.84E-05 | 7.119557 | 6.14943 | 2.292785 |
| CUST_72055_PI430048170 | 8.32E-03 | 3.858936 | -1.01477 | 2.292591 |
| CUST_132032_PI430048170 | 2.88E-03 | 4.467043 | 0.372367 | 2.292242 |
| CUST_112573_PI430048170 | 3.56E-05 | 7.352449 | 6.615378 | 2.289357 |
| CUST_134383_PI430048170 | 5.10E-03 | 4.13978 | -0.37544 | 2.284916 |
| CUST_118866_PI430048170 | 3.06E-03 | 4.431216 | 0.290489 | 2.284708 |
| CUST_115917_PI430048170 | 1.85E-04 | 6.144657 | 4.117323 | 2.283274 |
| CUST_20848_PI430048170 | 7.98E-03 | 3.883774 | -0.95841 | 2.277453 |
| CUST_38240_PI430048170 | 7.40E-03 | 3.925784 | -0.86299 | 2.275867 |
| CUST_58998_PI430048170 | 2.82E-05 | 7.533815 | 6.972866 | 2.274772 |
| CUST_111256_PI430048170 | 2.10E-04 | 6.049653 | 3.912609 | 2.272494 |
| CUST_140427_PI430048170 | 4.97E-02 | 2.790589 | -3.35509 | 2.270513 |
| CUST_144860_PI430048170 | 5.25E-05 | 7.053095 | 6.015048 | 2.267856 |
| CUST_17790_PI430048170 | 2.99E-04 | 5.835172 | 3.446445 | 2.267114 |
| CUST_129932_PI430048170 | 2.56E-05 | 7.601738 | 7.105537 | 2.265801 |
| CUST_100403_PI430048170 | 3.87E-06 | 9.179715 | 10.00268 | 2.265072 |
| CUST_80427_PI430048170 | 5.90E-05 | 6.967889 | 5.841852 | 2.257165 |
| CUST_52959_PI430048170 | 1.51E-05 | 8.020285 | 7.908426 | 2.254231 |
| CUST_109041_PI430048170 | 3.05E-04 | 5.823314 | 3.420518 | 2.249181 |
| CUST_112467_PI430048170 | 2.22E-04 | 6.014742 | 3.837106 | 2.248066 |
| CUST_61061_PI430048170 | 1.33E-02 | 3.587737 | -1.62658 | 2.247066 |
| CUST_83706_PI430048170 | 1.72E-03 | 4.771383 | 1.066685 | 2.245233 |
| CUST_53029_PI430048170 | 8.97E-05 | 6.65639 | 5.200064 | 2.243631 |
| CUST_4403_PI430048170 | 8.17E-03 | 3.869464 | -0.99089 | 2.239641 |
| CUST_77761_PI430048170 | 4.53E-02 | 2.848898 | -3.23388 | 2.239021 |
| CUST_60203_PI430048170 | 1.57E-04 | 6.2531 | 4.349618 | 2.237626 |
| CUST_3206_PI430048170 | 5.74E-08 | 13.14717 | 15.88297 | 2.233575 |
| CUST_89330_PI430048170 | 1.87E-03 | 4.722112 | 0.954489 | 2.23171 |
| CUST_125135_PI430048170 | 5.59E-03 | 4.086551 | -0.4969 | 2.230523 |
| CUST_135534_PI430048170 | 4.93E-04 | 5.52597 | 2.765287 | 2.229343 |
| CUST_124636_PI430048170 | 1.03E-02 | 3.734879 | -1.29553 | 2.22825 |
| CUST_132968_PI430048170 | 4.35E-04 | 5.604209 | 2.938606 | 2.228244 |
| CUST_40646_PI430048170 | 2.99E-04 | 5.834895 | 3.44584 | 2.227477 |
| CUST_144973_PI430048170 | 2.60E-02 | 3.188516 | -2.50925 | 2.226311 |
| CUST_144638_PI430048170 | 3.23E-03 | 4.40153 | 0.222639 | 2.222202 |
| CUST_10830_PI430048170 | 1.38E-04 | 6.341529 | 4.537928 | 2.217241 |
| CUST_56694_PI430048170 | 1.33E-03 | 4.920775 | 1.406148 | 2.216031 |
| CUST_128174_PI430048170 | 9.91E-04 | 5.09797 | 1.80707 | 2.214934 |
| CUST_120972_PI430048170 | 7.69E-05 | 6.762805 | 5.420824 | 2.212401 |
| CUST_137685_PI430048170 | 2.39E-07 | 11.78272 | 14.06291 | 2.211154 |
| CUST_54089_PI430048170 | 1.42E-03 | 4.881548 | 1.317127 | 2.209823 |
| CUST_16282_PI430048170 | 2.83E-02 | 3.137573 | -2.61975 | 2.209462 |
| CUST_48604_PI430048170 | 9.74E-05 | 6.592097 | 5.065939 | 2.208158 |
| CUST_83824_PI430048170 | 1.74E-04 | 6.185119 | 4.20417 | 2.208061 |
| CUST_134106_PI430048170 | 3.76E-03 | 4.31492 | 0.024669 | 2.207406 |
| CUST_138018_PI430048170 | 3.16E-04 | 5.803483 | 3.377118 | 2.203913 |
| CUST_60511_PI430048170 | 9.51E-04 | 5.123128 | 1.863816 | 2.20358 |
| CUST_98999_PI430048170 | 1.46E-03 | 4.868611 | 1.28775 | 2.202632 |
| CUST_124229_PI430048170 | 3.57E-02 | 2.998691 | -2.91794 | 2.200514 |
| CUST_111140_PI430048170 | 6.76E-03 | 3.977815 | -0.74465 | 2.198329 |
| CUST_136648_PI430048170 | 1.35E-04 | 6.359839 | 4.576792 | 2.198303 |
| CUST_136799_PI430048170 | 1.27E-02 | 3.610332 | -1.57591 | 2.195621 |
| CUST_47375_PI430048170 | 1.85E-04 | 6.143083 | 4.11394 | 2.194946 |
| CUST_138813_PI430048170 | 1.26E-04 | 6.404547 | 4.671505 | 2.190035 |
| CUST_63695_PI430048170 | 2.61E-05 | 7.587367 | 7.077522 | 2.187174 |
| CUST_132937_PI430048170 | 3.36E-04 | 5.766848 | 3.296827 | 2.184737 |
| CUST_99607_PI430048170 | 7.80E-03 | 3.896736 | -0.92898 | 2.182166 |
| CUST_127948_PI430048170 | 1.68E-03 | 4.783288 | 1.093778 | 2.179919 |
| CUST_129581_PI430048170 | 8.89E-03 | 3.822768 | -1.09676 | 2.179524 |
| CUST_74671_PI430048170 | 3.01E-04 | 5.831369 | 3.438132 | 2.179177 |
| CUST_118763_PI430048170 | 1.22E-02 | 3.634357 | -1.52196 | 2.175451 |
| CUST_97642_PI430048170 | 8.64E-03 | 3.838479 | -1.06116 | 2.173958 |
| CUST_111455_PI430048170 | 2.86E-04 | 5.86201 | 3.505072 | 2.170909 |
| CUST_30331_PI430048170 | 3.83E-04 | 5.682178 | 3.110692 | 2.169196 |
| CUST_110741_PI430048170 | 1.26E-03 | 4.954728 | 1.483129 | 2.165528 |
| CUST_127633_PI430048170 | 2.27E-04 | 6.001775 | 3.809024 | 2.164168 |
| CUST_128914_PI430048170 | 1.38E-03 | 4.901416 | 1.362227 | 2.163951 |
| CUST_140736_PI430048170 | 7.16E-05 | 6.81427 | 5.527028 | 2.163931 |
| CUST_116455_PI430048170 | 1.26E-04 | 6.408406 | 4.679668 | 2.162219 |
| CUST_99959_PI430048170 | 5.83E-04 | 5.420432 | 2.530531 | 2.161834 |
| CUST_74698_PI430048170 | 9.94E-05 | 6.575478 | 5.03118 | 2.160922 |
| CUST_8239_PI430048170 | 2.64E-04 | 5.911156 | 3.612214 | 2.160291 |
| CUST_96113_PI430048170 | 1.61E-03 | 4.810396 | 1.155444 | 2.158472 |
| CUST_128001_PI430048170 | 1.75E-02 | 3.424668 | -1.99029 | 2.158291 |
| CUST_73413_PI430048170 | 3.53E-03 | 4.351723 | 0.108791 | 2.155818 |
| CUST_39346_PI430048170 | 2.38E-03 | 4.578509 | 0.62697 | 2.154847 |
| CUST_138745_PI430048170 | 1.25E-02 | 3.622983 | -1.54751 | 2.151979 |
| CUST_135854_PI430048170 | 1.82E-04 | 6.15755 | 4.14502 | 2.148601 |
| CUST_49680_PI430048170 | 3.95E-04 | 5.662424 | 3.067152 | 2.146333 |
| CUST_25463_PI430048170 | 1.05E-02 | 3.723266 | -1.32174 | 2.145379 |
| CUST_60093_PI430048170 | 3.77E-04 | 5.691446 | 3.131105 | 2.14525 |
| CUST_33287_PI430048170 | 9.29E-05 | 6.626728 | 5.138255 | 2.142823 |
| CUST_133553_PI430048170 | 6.39E-05 | 6.898426 | 5.699905 | 2.142449 |
| CUST_138027_PI430048170 | 4.88E-03 | 4.165523 | -0.31667 | 2.139463 |
| CUST_68475_PI430048170 | 3.17E-05 | 7.450407 | 6.809046 | 2.137703 |
| CUST_144191_PI430048170 | 1.19E-03 | 4.985104 | 1.551938 | 2.137556 |
| CUST_103636_PI430048170 | 6.95E-06 | 8.69773 | 9.154952 | 2.136527 |
| CUST_49540_PI430048170 | 5.11E-04 | 5.50158 | 2.711131 | 2.133326 |
| CUST_17332_PI430048170 | 1.48E-03 | 4.859405 | 1.26684 | 2.13329 |
| CUST_67509_PI430048170 | 1.44E-03 | 4.875535 | 1.303475 | 2.131105 |
| CUST_81049_PI430048170 | 1.11E-02 | 3.692349 | -1.39146 | 2.131007 |
| CUST_56065_PI430048170 | 1.25E-04 | 6.412487 | 4.688299 | 2.129552 |
| CUST_12852_PI430048170 | 2.23E-02 | 3.280833 | -2.30762 | 2.123443 |
| CUST_134618_PI430048170 | 2.15E-03 | 4.638701 | 0.764334 | 2.121021 |
| CUST_128905_PI430048170 | 8.99E-03 | 3.815903 | -1.11231 | 2.120453 |
| CUST_122031_PI430048170 | 2.09E-05 | 7.779667 | 7.449928 | 2.119748 |
| CUST_65080_PI430048170 | 2.87E-05 | 7.525371 | 6.956329 | 2.118586 |
| CUST_61767_PI430048170 | 3.53E-05 | 7.359968 | 6.63029 | 2.118312 |
| CUST_109965_PI430048170 | 1.85E-02 | 3.3904 | -2.06621 | 2.117785 |
| CUST_80262_PI430048170 | 4.31E-03 | 4.237673 | -0.15187 | 2.116621 |
| CUST_136021_PI430048170 | 8.96E-04 | 5.156836 | 1.939773 | 2.115387 |
| CUST_134136_PI430048170 | 4.63E-04 | 5.56681 | 2.855835 | 2.112078 |
| CUST_16284_PI430048170 | 4.59E-02 | 2.840631 | -3.25113 | 2.109719 |
| CUST_119595_PI430048170 | 3.41E-02 | 3.027369 | -2.85675 | 2.108746 |
| CUST_140911_PI430048170 | 4.86E-03 | 4.167749 | -0.31159 | 2.105996 |
| CUST_127730_PI430048170 | 1.59E-04 | 6.244893 | 4.332088 | 2.104414 |
| CUST_34775_PI430048170 | 1.85E-04 | 6.140873 | 4.109192 | 2.10359 |
| CUST_37528_PI430048170 | 1.17E-02 | 3.662921 | -1.45773 | 2.100707 |
| CUST_120422_PI430048170 | 8.39E-03 | 3.854273 | -1.02535 | 2.098656 |
| CUST_132174_PI430048170 | 2.52E-02 | 3.206722 | -2.46963 | 2.097296 |
| CUST_130403_PI430048170 | 2.57E-03 | 4.53324 | 0.5236 | 2.096248 |
| CUST_77328_PI430048170 | 6.87E-05 | 6.846001 | 5.592328 | 2.093852 |
| CUST_87500_PI430048170 | 1.02E-03 | 5.07698 | 1.759688 | 2.093676 |
| CUST_45763_PI430048170 | 7.76E-05 | 6.755638 | 5.406004 | 2.093556 |
| CUST_127073_PI430048170 | 4.02E-02 | 2.925771 | -3.07253 | 2.093506 |
| CUST_55161_PI430048170 | 6.24E-03 | 4.025339 | -0.63644 | 2.093481 |
| CUST_113313_PI430048170 | 1.55E-02 | 3.497291 | -1.82878 | 2.092503 |
| CUST_125456_PI430048170 | 2.58E-03 | 4.530314 | 0.516916 | 2.091327 |
| CUST_132028_PI430048170 | 2.92E-04 | 5.848965 | 3.476586 | 2.091107 |
| CUST_67913_PI430048170 | 2.01E-05 | 7.81455 | 7.516912 | 2.089642 |
| CUST_100509_PI430048170 | 1.03E-05 | 8.336442 | 8.498268 | 2.089531 |
| CUST_88651_PI430048170 | 9.89E-03 | 3.759994 | -1.2388 | 2.086211 |
| CUST_112007_PI430048170 | 2.64E-05 | 7.581799 | 7.066659 | 2.086039 |
| CUST_127098_PI430048170 | 3.66E-04 | 5.7099 | 3.171722 | 2.085841 |
| CUST_36808_PI430048170 | 7.28E-03 | 3.935052 | -0.84192 | 2.085341 |
| CUST_83936_PI430048170 | 1.93E-03 | 4.703506 | 0.912095 | 2.084956 |
| CUST_116266_PI430048170 | 1.97E-03 | 4.689531 | 0.880244 | 2.084604 |
| CUST_79139_PI430048170 | 6.88E-04 | 5.317536 | 2.300644 | 2.082013 |
| CUST_138259_PI430048170 | 4.85E-03 | 4.168935 | -0.30888 | 2.081824 |
| CUST_128313_PI430048170 | 3.22E-05 | 7.436999 | 6.78262 | 2.080422 |
| CUST_34777_PI430048170 | 2.87E-04 | 5.860955 | 3.502769 | 2.077279 |
| CUST_28526_PI430048170 | 2.06E-02 | 3.326487 | -2.20729 | 2.076851 |
| CUST_5120_PI430048170 | 2.33E-03 | 4.589133 | 0.651222 | 2.076075 |
| CUST_136540_PI430048170 | 3.98E-05 | 7.266717 | 6.444751 | 2.074657 |
| CUST_110999_PI430048170 | 6.75E-04 | 5.329957 | 2.328444 | 2.074357 |
| CUST_141492_PI430048170 | 3.19E-06 | 9.363084 | 10.31686 | 2.074342 |
| CUST_102585_PI430048170 | 5.44E-05 | 7.025956 | 5.959993 | 2.071624 |
| CUST_135608_PI430048170 | 4.29E-02 | 2.883868 | -3.16069 | 2.069375 |
| CUST_12820_PI430048170 | 3.33E-02 | 3.041337 | -2.82688 | 2.06925 |
| CUST_34124_PI430048170 | 1.32E-03 | 4.923333 | 1.411951 | 2.064296 |
| CUST_112211_PI430048170 | 2.04E-04 | 6.067465 | 3.951076 | 2.063444 |
| CUST_93426_PI430048170 | 1.95E-05 | 7.836216 | 7.558428 | 2.062334 |
| CUST_46766_PI430048170 | 1.37E-02 | 3.570424 | -1.66537 | 2.060791 |
| CUST_75522_PI430048170 | 2.52E-03 | 4.544073 | 0.548341 | 2.060187 |
| CUST_137222_PI430048170 | 4.29E-04 | 5.612584 | 2.957119 | 2.057772 |
| CUST_31390_PI430048170 | 4.41E-02 | 2.8662 | -3.19772 | 2.055635 |
| CUST_16290_PI430048170 | 2.36E-02 | 3.246793 | -2.38216 | 2.054735 |
| CUST_138921_PI430048170 | 1.60E-03 | 4.815316 | 1.166632 | 2.054365 |
| CUST_5127_PI430048170 | 2.67E-03 | 4.510143 | 0.470841 | 2.053966 |
| CUST_144617_PI430048170 | 2.00E-03 | 4.682692 | 0.864654 | 2.05011 |
| CUST_30333_PI430048170 | 1.28E-04 | 6.392011 | 4.644974 | 2.047682 |
| CUST_111008_PI430048170 | 8.13E-04 | 5.216827 | 2.074738 | 2.045215 |
| CUST_31632_PI430048170 | 6.83E-03 | 3.971755 | -0.75844 | 2.045028 |
| CUST_142558_PI430048170 | 1.26E-02 | 3.618333 | -1.55795 | 2.044764 |
| CUST_118219_PI430048170 | 4.85E-02 | 2.805616 | -3.32395 | 2.044192 |
| CUST_36589_PI430048170 | 7.63E-03 | 3.908778 | -0.90163 | 2.042548 |
| CUST_56513_PI430048170 | 9.03E-06 | 8.429414 | 8.669015 | 2.042172 |
| CUST_71901_PI430048170 | 2.47E-05 | 7.639107 | 7.178243 | 2.041281 |
| CUST_27057_PI430048170 | 1.14E-03 | 5.01208 | 1.612994 | 2.041118 |
| CUST_62534_PI430048170 | 1.93E-02 | 3.365463 | -2.12134 | 2.041 |
| CUST_81580_PI430048170 | 1.19E-03 | 4.984205 | 1.549902 | 2.038197 |
| CUST_125471_PI430048170 | 4.18E-03 | 4.254485 | -0.11345 | 2.037217 |
| CUST_33031_PI430048170 | 1.41E-02 | 3.553713 | -1.70277 | 2.032679 |
| CUST_11913_PI430048170 | 3.11E-02 | 3.083029 | -2.73742 | 2.03225 |
| CUST_92408_PI430048170 | 3.86E-03 | 4.298362 | -0.01318 | 2.03044 |
| CUST_36524_PI430048170 | 9.62E-03 | 3.776358 | -1.20181 | 2.030186 |
| CUST_116839_PI430048170 | 9.93E-06 | 8.366406 | 8.553432 | 2.030166 |
| CUST_80068_PI430048170 | 1.01E-04 | 6.565555 | 5.010406 | 2.028298 |
| CUST_112326_PI430048170 | 2.10E-04 | 6.050416 | 3.914258 | 2.026207 |
| CUST_63354_PI430048170 | 1.89E-02 | 3.381497 | -2.08591 | 2.024302 |
| CUST_54304_PI430048170 | 7.87E-04 | 5.236735 | 2.119463 | 2.023788 |
| CUST_60252_PI430048170 | 5.16E-03 | 4.132553 | -0.39194 | 2.022793 |
| CUST_96819_PI430048170 | 1.29E-04 | 6.388984 | 4.638565 | 2.021782 |
| CUST_48159_PI430048170 | 7.07E-03 | 3.952298 | -0.80271 | 2.02176 |
| CUST_127826_PI430048170 | 1.11E-03 | 5.030178 | 1.65393 | 2.020542 |
| CUST_127496_PI430048170 | 3.39E-04 | 5.761746 | 3.285634 | 2.018787 |
| CUST_134326_PI430048170 | 1.32E-06 | 10.13077 | 11.5838 | 2.017616 |
| CUST_16406_PI430048170 | 5.29E-04 | 5.481668 | 2.666874 | 2.016839 |
| CUST_16691_PI430048170 | 1.22E-02 | 3.634118 | -1.52249 | 2.016693 |
| CUST_97309_PI430048170 | 1.26E-04 | 6.408426 | 4.67971 | 2.015746 |
| CUST_127037_PI430048170 | 1.66E-02 | 3.457921 | -1.91643 | 2.015566 |
| CUST_82244_PI430048170 | 3.01E-05 | 7.49172 | 6.890313 | 2.01244 |
| CUST_60604_PI430048170 | 1.89E-03 | 4.715994 | 0.94055 | 2.011485 |
| CUST_105721_PI430048170 | 2.37E-04 | 5.97713 | 3.755595 | 2.011476 |
| CUST_123162_PI430048170 | 1.55E-03 | 4.831508 | 1.203446 | 2.009916 |
| CUST_60521_PI430048170 | 1.80E-02 | 3.410389 | -2.02195 | 2.008209 |
| CUST_114415_PI430048170 | 1.93E-04 | 6.107404 | 4.037185 | 2.008189 |
| CUST_87949_PI430048170 | 5.95E-04 | 5.407759 | 2.502271 | 2.00791 |
| CUST_4859_PI430048170 | 4.55E-04 | 5.57673 | 2.877805 | 2.007105 |
| CUST_54094_PI430048170 | 1.46E-04 | 6.300847 | 4.45142 | 2.006945 |
| CUST_129288_PI430048170 | 2.20E-03 | 4.624657 | 0.732295 | 2.006876 |
| CUST_97943_PI430048170 | 1.05E-04 | 6.537096 | 4.950756 | 2.006217 |
| CUST_36602_PI430048170 | 1.07E-02 | 3.715655 | -1.33891 | 2.00611 |
| CUST_45430_PI430048170 | 3.07E-05 | 7.477181 | 6.861742 | 2.003825 |
| CUST_109062_PI430048170 | 1.58E-04 | 6.248578 | 4.33996 | 2.00357 |
| CUST_111896_PI430048170 | 3.78E-06 | 9.214469 | 10.06258 | 2.003151 |
| CUST_100586_PI430048170 | 3.77E-03 | 4.313256 | 0.020866 | 2.001177 |
| CUST_41613_PI430048170 | 2.44E-05 | 7.654516 | 7.208166 | 2.00115 |
| CUST_134850_PI430048170 | 7.27E-07 | 10.65528 | 12.40619 | 2.000553 |
| CUST_87505_PI430048170 | 3.35E-05 | 7.410125 | 6.729572 | 1.996657 |
| CUST_129489_PI430048170 | 1.80E-03 | 4.743557 | 1.003334 | 1.9959 |
| CUST_10884_PI430048170 | 7.51E-05 | 6.781476 | 5.459397 | 1.991772 |
| CUST_37984_PI430048170 | 7.20E-03 | 3.94143 | -0.82742 | 1.990905 |
| CUST_66389_PI430048170 | 2.70E-04 | 5.897642 | 3.582781 | 1.990722 |
| CUST_130121_PI430048170 | 2.09E-02 | 3.319017 | -2.22373 | 1.988995 |
| CUST_143971_PI430048170 | 1.02E-05 | 8.344576 | 8.513257 | 1.987095 |
| CUST_24161_PI430048170 | 1.14E-04 | 6.472546 | 4.815056 | 1.985253 |
| CUST_130680_PI430048170 | 2.43E-02 | 3.228152 | -2.42289 | 1.985087 |
| CUST_44750_PI430048170 | 7.31E-03 | 3.932551 | -0.84761 | 1.982704 |
| CUST_142436_PI430048170 | 2.56E-05 | 7.6007 | 7.103513 | 1.982346 |
| CUST_133291_PI430048170 | 2.52E-02 | 3.207114 | -2.46877 | 1.976647 |
| CUST_128278_PI430048170 | 1.14E-04 | 6.471037 | 4.811876 | 1.974313 |
| CUST_73218_PI430048170 | 2.10E-04 | 6.051775 | 3.917193 | 1.973058 |
| CUST_97706_PI430048170 | 7.96E-06 | 8.590988 | 8.962849 | 1.971523 |
| CUST_70370_PI430048170 | 2.41E-04 | 5.968116 | 3.736035 | 1.968756 |
| CUST_126145_PI430048170 | 5.54E-04 | 5.455199 | 2.607986 | 1.967794 |
| CUST_142630_PI430048170 | 1.74E-03 | 4.763148 | 1.047939 | 1.967573 |
| CUST_137601_PI430048170 | 2.47E-02 | 3.218988 | -2.44289 | 1.967283 |
| CUST_135913_PI430048170 | 3.76E-05 | 7.307371 | 6.525793 | 1.965541 |
| CUST_136264_PI430048170 | 2.88E-03 | 4.465797 | 0.369519 | 1.964736 |
| CUST_140968_PI430048170 | 6.32E-03 | 4.018116 | -0.65289 | 1.964376 |
| CUST_98416_PI430048170 | 3.61E-05 | 7.340973 | 6.592598 | 1.962523 |
| CUST_136673_PI430048170 | 1.09E-03 | 5.04234 | 1.681428 | 1.959588 |
| CUST_6917_PI430048170 | 2.57E-06 | 9.547271 | 10.62788 | 1.959151 |
| CUST_35396_PI430048170 | 2.18E-06 | 9.712225 | 10.90261 | 1.957963 |
| CUST_133327_PI430048170 | 5.76E-06 | 8.838594 | 9.40603 | 1.957208 |
| CUST_140423_PI430048170 | 2.35E-06 | 9.632842 | 10.77085 | 1.954866 |
| CUST_61065_PI430048170 | 1.38E-02 | 3.567121 | -1.67277 | 1.954121 |
| CUST_83440_PI430048170 | 1.71E-03 | 4.775592 | 1.076264 | 1.953179 |
| CUST_136306_PI430048170 | 1.93E-04 | 6.108985 | 4.040588 | 1.952983 |
| CUST_112522_PI430048170 | 1.02E-03 | 5.077842 | 1.761635 | 1.952341 |
| CUST_69768_PI430048170 | 8.74E-03 | 3.832664 | -1.07434 | 1.952246 |
| CUST_134312_PI430048170 | 2.71E-02 | 3.164373 | -2.56169 | 1.9517 |
| CUST_82398_PI430048170 | 4.89E-02 | 2.800186 | -3.33521 | 1.94983 |
| CUST_67091_PI430048170 | 3.75E-04 | 5.696082 | 3.141311 | 1.949525 |
| CUST_143810_PI430048170 | 1.32E-03 | 4.926861 | 1.419952 | 1.947578 |
| CUST_132086_PI430048170 | 7.52E-07 | 10.6284 | 12.36485 | 1.94706 |
| CUST_27095_PI430048170 | 2.26E-02 | 3.272074 | -2.32682 | 1.942531 |
| CUST_46991_PI430048170 | 7.65E-03 | 3.90712 | -0.9054 | 1.941854 |
| CUST_20741_PI430048170 | 1.07E-03 | 5.050366 | 1.699568 | 1.941662 |
| CUST_36918_PI430048170 | 1.21E-02 | 3.640102 | -1.50905 | 1.941439 |
| CUST_86330_PI430048170 | 1.04E-02 | 3.731701 | -1.30271 | 1.940501 |
| CUST_69199_PI430048170 | 4.73E-03 | 4.183609 | -0.27537 | 1.940303 |
| CUST_5035_PI430048170 | 1.08E-04 | 6.518069 | 4.910813 | 1.938224 |
| CUST_57337_PI430048170 | 1.52E-02 | 3.506776 | -1.80763 | 1.937072 |
| CUST_129889_PI430048170 | 4.23E-03 | 4.248137 | -0.12796 | 1.936795 |
| CUST_100130_PI430048170 | 9.75E-05 | 6.590079 | 5.061722 | 1.934991 |
| CUST_89014_PI430048170 | 3.78E-03 | 4.311882 | 0.017725 | 1.934609 |
| CUST_69612_PI430048170 | 3.10E-03 | 4.422832 | 0.271328 | 1.934491 |
| CUST_81663_PI430048170 | 1.85E-04 | 6.141782 | 4.111144 | 1.934349 |
| CUST_80749_PI430048170 | 3.68E-05 | 7.32272 | 6.556329 | 1.933587 |
| CUST_36604_PI430048170 | 1.22E-02 | 3.636712 | -1.51666 | 1.932112 |
| CUST_41271_PI430048170 | 1.04E-03 | 5.066734 | 1.736548 | 1.931771 |
| CUST_92043_PI430048170 | 6.34E-04 | 5.36876 | 2.415207 | 1.931174 |
| CUST_36957_PI430048170 | 1.20E-02 | 3.647403 | -1.49263 | 1.93064 |
| CUST_62699_PI430048170 | 2.06E-03 | 4.661205 | 0.815661 | 1.930607 |
| CUST_108577_PI430048170 | 1.39E-02 | 3.559889 | -1.68895 | 1.928073 |
| CUST_145327_PI430048170 | 1.73E-04 | 6.19019 | 4.21504 | 1.927134 |
| CUST_102920_PI430048170 | 1.29E-02 | 3.601524 | -1.59567 | 1.926433 |
| CUST_135321_PI430048170 | 1.94E-02 | 3.363976 | -2.12462 | 1.925213 |
| CUST_36551_PI430048170 | 1.26E-02 | 3.61531 | -1.56473 | 1.924969 |
| CUST_138844_PI430048170 | 3.63E-05 | 7.33408 | 6.578907 | 1.924658 |
| CUST_74488_PI430048170 | 3.30E-02 | 3.047435 | -2.81382 | 1.92144 |
| CUST_57818_PI430048170 | 1.58E-02 | 3.484322 | -1.85768 | 1.921288 |
| CUST_85995_PI430048170 | 1.81E-03 | 4.742222 | 1.000294 | 1.921028 |
| CUST_254_PI430048170 | 3.31E-03 | 4.387175 | 0.189827 | 1.919778 |
| CUST_79138_PI430048170 | 3.70E-03 | 4.323416 | 0.044087 | 1.918663 |
| CUST_58516_PI430048170 | 1.38E-03 | 4.897943 | 1.354346 | 1.918318 |
| CUST_7861_PI430048170 | 5.67E-04 | 5.440122 | 2.574411 | 1.916396 |
| CUST_78375_PI430048170 | 5.53E-03 | 4.093083 | -0.482 | 1.915678 |
| CUST_98144_PI430048170 | 2.70E-04 | 5.896162 | 3.579555 | 1.914891 |
| CUST_28407_PI430048170 | 4.23E-02 | 2.893981 | -3.13946 | 1.913451 |
| CUST_33959_PI430048170 | 2.09E-03 | 4.654979 | 0.801463 | 1.913207 |
| CUST_129035_PI430048170 | 1.36E-02 | 3.573369 | -1.65878 | 1.913143 |
| CUST_27497_PI430048170 | 2.61E-03 | 4.52426 | 0.503088 | 1.910894 |
| CUST_137105_PI430048170 | 1.89E-02 | 3.380899 | -2.08723 | 1.910032 |
| CUST_36783_PI430048170 | 1.27E-02 | 3.611065 | -1.57426 | 1.909133 |
| CUST_36929_PI430048170 | 1.34E-02 | 3.581671 | -1.64018 | 1.908907 |
| CUST_127130_PI430048170 | 1.49E-03 | 4.854568 | 1.25585 | 1.90871 |
| CUST_36537_PI430048170 | 1.43E-02 | 3.543906 | -1.7247 | 1.908493 |
| CUST_116007_PI430048170 | 1.53E-04 | 6.268793 | 4.383109 | 1.907687 |
| CUST_60602_PI430048170 | 3.61E-02 | 2.99208 | -2.93201 | 1.904058 |
| CUST_34774_PI430048170 | 7.33E-03 | 3.931298 | -0.85046 | 1.903862 |
| CUST_57680_PI430048170 | 3.67E-04 | 5.708336 | 3.16828 | 1.903477 |
| CUST_7652_PI430048170 | 1.03E-03 | 5.071846 | 1.748096 | 1.902236 |
| CUST_115673_PI430048170 | 2.20E-03 | 4.622825 | 0.728114 | 1.901902 |
| CUST_139432_PI430048170 | 8.15E-04 | 5.214849 | 2.070294 | 1.901085 |
| CUST_93781_PI430048170 | 1.77E-03 | 4.753581 | 1.026159 | 1.900105 |
| CUST_91508_PI430048170 | 3.52E-04 | 5.738649 | 3.234922 | 1.899478 |
| CUST_144155_PI430048170 | 8.31E-03 | 3.859754 | -1.01292 | 1.898776 |
| CUST_47890_PI430048170 | 4.96E-05 | 7.099546 | 6.109035 | 1.898507 |
| CUST_5554_PI430048170 | 1.85E-03 | 4.729763 | 0.971919 | 1.894649 |
| CUST_54443_PI430048170 | 3.34E-03 | 4.381154 | 0.176064 | 1.893324 |
| CUST_68376_PI430048170 | 6.36E-04 | 5.366631 | 2.41045 | 1.893242 |
| CUST_53522_PI430048170 | 6.10E-03 | 4.038226 | -0.60707 | 1.891875 |
| CUST_23275_PI430048170 | 6.52E-03 | 3.999101 | -0.6962 | 1.891076 |
| CUST_78610_PI430048170 | 1.46E-06 | 10.01851 | 11.4033 | 1.888265 |
| CUST_136815_PI430048170 | 1.50E-02 | 3.516239 | -1.78651 | 1.887548 |
| CUST_74808_PI430048170 | 5.63E-04 | 5.443985 | 2.583017 | 1.88684 |
| CUST_138714_PI430048170 | 9.56E-03 | 3.780482 | -1.19248 | 1.886491 |
| CUST_122154_PI430048170 | 9.86E-03 | 3.761882 | -1.23453 | 1.8863 |
| CUST_99135_PI430048170 | 1.02E-03 | 5.080911 | 1.768565 | 1.885701 |
| CUST_79123_PI430048170 | 2.44E-03 | 4.563215 | 0.592053 | 1.885518 |
| CUST_131151_PI430048170 | 2.06E-03 | 4.662082 | 0.817662 | 1.885364 |
| CUST_123664_PI430048170 | 3.84E-04 | 5.68116 | 3.108448 | 1.885294 |
| CUST_66106_PI430048170 | 7.26E-03 | 3.936913 | -0.83769 | 1.882222 |
| CUST_24789_PI430048170 | 2.45E-02 | 3.223687 | -2.43264 | 1.881653 |
| CUST_4951_PI430048170 | 1.99E-05 | 7.82037 | 7.528072 | 1.88165 |
| CUST_138334_PI430048170 | 4.13E-05 | 7.243341 | 6.398048 | 1.88115 |
| CUST_128354_PI430048170 | 9.22E-03 | 3.801883 | -1.14405 | 1.880424 |
| CUST_6022_PI430048170 | 1.71E-03 | 4.773549 | 1.071615 | 1.879804 |
| CUST_14274_PI430048170 | 4.23E-03 | 4.247991 | -0.12829 | 1.879696 |
| CUST_29275_PI430048170 | 1.52E-02 | 3.507608 | -1.80577 | 1.879145 |
| CUST_75464_PI430048170 | 4.16E-05 | 7.236123 | 6.383609 | 1.877882 |
| CUST_145687_PI430048170 | 1.29E-03 | 4.938725 | 1.446855 | 1.874513 |
| CUST_25074_PI430048170 | 5.33E-05 | 7.044015 | 5.99664 | 1.873377 |
| CUST_142598_PI430048170 | 1.37E-04 | 6.347377 | 4.550346 | 1.87174 |
| CUST_109263_PI430048170 | 1.32E-06 | 10.12711 | 11.57794 | 1.871489 |
| CUST_366_PI430048170 | 4.51E-04 | 5.581858 | 2.889156 | 1.871442 |
| CUST_19461_PI430048170 | 6.85E-03 | 3.97013 | -0.76214 | 1.8714 |
| CUST_19133_PI430048170 | 2.52E-02 | 3.206998 | -2.46902 | 1.870878 |
| CUST_38537_PI430048170 | 4.89E-04 | 5.532165 | 2.779033 | 1.870815 |
| CUST_59618_PI430048170 | 1.06E-03 | 5.058297 | 1.71749 | 1.870526 |
| CUST_20073_PI430048170 | 2.84E-04 | 5.867093 | 3.516167 | 1.869983 |
| CUST_46678_PI430048170 | 1.85E-03 | 4.729457 | 0.97122 | 1.866695 |
| CUST_49421_PI430048170 | 4.04E-02 | 2.923084 | -3.0782 | 1.864072 |
| CUST_1377_PI430048170 | 2.82E-03 | 4.47848 | 0.398501 | 1.861505 |
| CUST_60075_PI430048170 | 1.65E-04 | 6.220363 | 4.27965 | 1.859763 |
| CUST_14145_PI430048170 | 9.44E-03 | 3.787376 | -1.17688 | 1.859065 |
| CUST_97319_PI430048170 | 2.81E-04 | 5.871892 | 3.526638 | 1.85878 |
| CUST_66387_PI430048170 | 7.11E-04 | 5.295913 | 2.252212 | 1.858486 |
| CUST_105688_PI430048170 | 8.74E-04 | 5.173299 | 1.976838 | 1.858445 |
| CUST_132034_PI430048170 | 2.54E-02 | 3.201076 | -2.48192 | 1.858402 |
| CUST_18823_PI430048170 | 8.90E-04 | 5.160433 | 1.947874 | 1.856292 |
| CUST_127590_PI430048170 | 1.26E-03 | 4.952745 | 1.478633 | 1.854577 |
| CUST_139291_PI430048170 | 9.78E-04 | 5.106088 | 1.825385 | 1.854371 |
| CUST_24181_PI430048170 | 1.92E-04 | 6.11105 | 4.045035 | 1.853653 |
| CUST_112132_PI430048170 | 1.88E-02 | 3.383699 | -2.08104 | 1.853141 |
| CUST_99779_PI430048170 | 5.88E-04 | 5.415467 | 2.519461 | 1.852524 |
| CUST_94470_PI430048170 | 1.08E-02 | 3.710637 | -1.35023 | 1.851962 |
| CUST_125455_PI430048170 | 1.20E-02 | 3.64716 | -1.49318 | 1.851839 |
| CUST_100478_PI430048170 | 1.76E-05 | 7.902201 | 7.684454 | 1.850859 |
| CUST_122590_PI430048170 | 1.71E-02 | 3.440379 | -1.95541 | 1.850129 |
| CUST_137047_PI430048170 | 3.37E-04 | 5.76479 | 3.292312 | 1.849187 |
| CUST_104094_PI430048170 | 2.56E-05 | 7.607275 | 7.116321 | 1.849041 |
| CUST_122038_PI430048170 | 3.40E-03 | 4.372168 | 0.155524 | 1.848026 |
| CUST_64149_PI430048170 | 3.42E-05 | 7.38924 | 6.688277 | 1.847883 |
| CUST_75081_PI430048170 | 3.05E-03 | 4.433489 | 0.295685 | 1.847469 |
| CUST_53136_PI430048170 | 1.49E-04 | 6.284782 | 4.4172 | 1.847015 |
| CUST_131934_PI430048170 | 1.07E-03 | 5.048429 | 1.695191 | 1.845587 |
| CUST_14352_PI430048170 | 3.01E-02 | 3.103302 | -2.69376 | 1.845204 |
| CUST_113140_PI430048170 | 7.32E-04 | 5.278411 | 2.212983 | 1.844176 |
| CUST_31322_PI430048170 | 1.16E-02 | 3.667971 | -1.44636 | 1.843797 |
| CUST_58504_PI430048170 | 4.24E-04 | 5.619065 | 2.971444 | 1.842132 |
| CUST_129390_PI430048170 | 1.42E-02 | 3.546553 | -1.71879 | 1.842099 |
| CUST_13420_PI430048170 | 4.59E-05 | 7.157333 | 6.225534 | 1.839539 |
| CUST_133136_PI430048170 | 1.47E-05 | 8.052284 | 7.968774 | 1.837848 |
| CUST_138614_PI430048170 | 2.58E-03 | 4.530172 | 0.516592 | 1.836933 |
| CUST_19536_PI430048170 | 1.04E-05 | 8.314216 | 8.457268 | 1.836277 |
| CUST_35440_PI430048170 | 1.30E-04 | 6.38213 | 4.624049 | 1.835588 |
| CUST_111815_PI430048170 | 3.27E-07 | 11.50223 | 13.66433 | 1.833168 |
| CUST_135520_PI430048170 | 3.30E-04 | 5.779456 | 3.324477 | 1.832767 |
| CUST_56697_PI430048170 | 1.34E-04 | 6.362501 | 4.582438 | 1.832013 |
| CUST_122710_PI430048170 | 2.85E-03 | 4.472784 | 0.385486 | 1.830325 |
| CUST_142028_PI430048170 | 3.63E-04 | 5.71968 | 3.193231 | 1.830188 |
| CUST_127579_PI430048170 | 3.20E-05 | 7.443882 | 6.79619 | 1.829055 |
| CUST_140360_PI430048170 | 2.99E-02 | 3.105436 | -2.68916 | 1.827899 |
| CUST_50235_PI430048170 | 1.94E-02 | 3.361545 | -2.12999 | 1.827682 |
| CUST_29006_PI430048170 | 2.69E-03 | 4.506083 | 0.461565 | 1.827321 |
| CUST_93724_PI430048170 | 2.17E-03 | 4.633577 | 0.752645 | 1.825884 |
| CUST_106364_PI430048170 | 8.27E-05 | 6.713752 | 5.319256 | 1.825072 |
| CUST_45604_PI430048170 | 4.60E-04 | 5.571394 | 2.865988 | 1.82505 |
| CUST_130797_PI430048170 | 6.41E-03 | 4.008782 | -0.67415 | 1.820724 |
| CUST_122325_PI430048170 | 1.11E-02 | 3.691465 | -1.39345 | 1.820564 |
| CUST_126976_PI430048170 | 1.30E-02 | 3.596459 | -1.60703 | 1.818265 |
| CUST_139014_PI430048170 | 1.31E-03 | 4.931874 | 1.431319 | 1.817237 |
| CUST_35058_PI430048170 | 1.50E-02 | 3.514893 | -1.78951 | 1.817125 |
| CUST_10491_PI430048170 | 1.03E-03 | 5.072629 | 1.749863 | 1.81701 |
| CUST_110193_PI430048170 | 6.25E-05 | 6.919021 | 5.742061 | 1.816236 |
| CUST_17011_PI430048170 | 1.58E-03 | 4.821969 | 1.18176 | 1.814449 |
| CUST_31276_PI430048170 | 1.09E-04 | 6.507152 | 4.887876 | 1.814356 |
| CUST_60708_PI430048170 | 1.26E-04 | 6.408097 | 4.679016 | 1.813391 |
| CUST_138125_PI430048170 | 1.32E-06 | 10.13395 | 11.58889 | 1.812166 |
| CUST_122087_PI430048170 | 1.51E-02 | 3.511313 | -1.7975 | 1.810498 |
| CUST_111783_PI430048170 | 1.23E-04 | 6.42446 | 4.713606 | 1.810015 |
| CUST_59935_PI430048170 | 7.41E-04 | 5.2723 | 2.199279 | 1.808834 |
| CUST_80616_PI430048170 | 1.47E-02 | 3.528552 | -1.75902 | 1.808289 |
| CUST_42146_PI430048170 | 2.55E-04 | 5.933559 | 3.660959 | 1.806604 |
| CUST_127845_PI430048170 | 1.62E-03 | 4.805908 | 1.145238 | 1.804433 |
| CUST_46814_PI430048170 | 5.81E-04 | 5.423254 | 2.536822 | 1.802512 |
| CUST_136313_PI430048170 | 6.16E-03 | 4.032686 | -0.6197 | 1.802093 |
| CUST_96465_PI430048170 | 3.04E-03 | 4.434981 | 0.299096 | 1.80166 |
| CUST_69665_PI430048170 | 3.05E-03 | 4.432656 | 0.293782 | 1.801255 |
| CUST_133566_PI430048170 | 2.27E-03 | 4.606126 | 0.690008 | 1.800523 |
| CUST_89277_PI430048170 | 1.80E-03 | 4.745499 | 1.007758 | 1.80033 |
| CUST_63521_PI430048170 | 2.17E-03 | 4.632271 | 0.749666 | 1.797118 |
| CUST_75959_PI430048170 | 8.94E-04 | 5.158401 | 1.943298 | 1.796296 |
| CUST_61025_PI430048170 | 1.02E-02 | 3.739562 | -1.28496 | 1.794844 |
| CUST_129423_PI430048170 | 2.80E-03 | 4.482977 | 0.408776 | 1.792584 |
| CUST_96937_PI430048170 | 7.66E-05 | 6.764996 | 5.425352 | 1.790537 |
| CUST_2219_PI430048170 | 1.26E-02 | 3.618417 | -1.55776 | 1.788832 |
| CUST_68532_PI430048170 | 2.27E-02 | 3.269163 | -2.3332 | 1.788501 |
| CUST_131120_PI430048170 | 1.13E-02 | 3.680799 | -1.41748 | 1.787617 |
| CUST_97988_PI430048170 | 1.61E-03 | 4.810507 | 1.155697 | 1.787253 |
| CUST_138611_PI430048170 | 1.24E-04 | 6.416943 | 4.697719 | 1.7851 |
| CUST_28377_PI430048170 | 1.27E-03 | 4.946514 | 1.464511 | 1.783474 |
| CUST_141388_PI430048170 | 1.30E-06 | 10.16152 | 11.63296 | 1.783207 |
| CUST_23167_PI430048170 | 5.51E-03 | 4.094434 | -0.47892 | 1.781387 |
| CUST_106624_PI430048170 | 2.54E-05 | 7.613045 | 7.127556 | 1.780641 |
| CUST_81560_PI430048170 | 4.11E-02 | 2.910136 | -3.10548 | 1.780328 |
| CUST_74201_PI430048170 | 4.02E-03 | 4.276731 | -0.06262 | 1.778825 |
| CUST_31090_PI430048170 | 2.77E-02 | 3.150987 | -2.59071 | 1.778075 |
| CUST_142243_PI430048170 | 2.32E-05 | 7.695727 | 7.288023 | 1.77725 |
| CUST_38094_PI430048170 | 6.45E-05 | 6.892261 | 5.687274 | 1.777109 |
| CUST_55284_PI430048170 | 2.82E-03 | 4.479934 | 0.401822 | 1.77463 |
| CUST_138803_PI430048170 | 1.04E-05 | 8.323641 | 8.474662 | 1.774058 |
| CUST_49668_PI430048170 | 4.00E-02 | 2.929541 | -3.06457 | 1.773847 |
| CUST_76048_PI430048170 | 3.99E-05 | 7.26399 | 6.439307 | 1.773398 |
| CUST_118767_PI430048170 | 8.86E-03 | 3.824641 | -1.09252 | 1.773352 |
| CUST_25372_PI430048170 | 4.23E-02 | 2.892946 | -3.14164 | 1.773077 |
| CUST_51460_PI430048170 | 3.51E-02 | 3.009663 | -2.89455 | 1.772914 |
| CUST_137346_PI430048170 | 2.31E-02 | 3.260786 | -2.35155 | 1.772767 |
| CUST_115260_PI430048170 | 1.20E-04 | 6.442692 | 4.752108 | 1.771168 |
| CUST_113982_PI430048170 | 1.52E-04 | 6.273488 | 4.393123 | 1.770522 |
| CUST_109312_PI430048170 | 1.65E-03 | 4.794163 | 1.118521 | 1.770502 |
| CUST_135325_PI430048170 | 1.01E-02 | 3.747422 | -1.26721 | 1.77036 |
| CUST_127989_PI430048170 | 7.45E-03 | 3.922337 | -0.87082 | 1.769841 |
| CUST_70003_PI430048170 | 3.24E-04 | 5.789906 | 3.347379 | 1.768874 |
| CUST_84042_PI430048170 | 2.63E-04 | 5.914004 | 3.618415 | 1.768846 |
| CUST_654_PI430048170 | 1.93E-03 | 4.701709 | 0.907999 | 1.768277 |
| CUST_108483_PI430048170 | 6.11E-05 | 6.941601 | 5.788212 | 1.766934 |
| CUST_25950_PI430048170 | 1.26E-02 | 3.615609 | -1.56406 | 1.765525 |
| CUST_69807_PI430048170 | 1.96E-02 | 3.356554 | -2.14101 | 1.76498 |
| CUST_124918_PI430048170 | 1.01E-02 | 3.748291 | -1.26524 | 1.764562 |
| CUST_127197_PI430048170 | 9.47E-05 | 6.612761 | 5.109109 | 1.76241 |
| CUST_105577_PI430048170 | 4.95E-07 | 11.05062 | 13.0039 | 1.761485 |
| CUST_122160_PI430048170 | 1.23E-03 | 4.966986 | 1.510902 | 1.761144 |
| CUST_128518_PI430048170 | 2.01E-03 | 4.679034 | 0.856313 | 1.760829 |
| CUST_16211_PI430048170 | 4.87E-03 | 4.166803 | -0.31375 | 1.760489 |
| CUST_34773_PI430048170 | 3.91E-04 | 5.668863 | 3.08135 | 1.760042 |
| CUST_37404_PI430048170 | 2.40E-02 | 3.237239 | -2.40305 | 1.759667 |
| CUST_59477_PI430048170 | 7.81E-04 | 5.241376 | 2.129884 | 1.759159 |
| CUST_135046_PI430048170 | 1.73E-02 | 3.43304 | -1.97171 | 1.758046 |
| CUST_11767_PI430048170 | 1.33E-03 | 4.920796 | 1.406197 | 1.758044 |
| CUST_62623_PI430048170 | 9.22E-05 | 6.633789 | 5.15298 | 1.756004 |
| CUST_142509_PI430048170 | 5.55E-04 | 5.454054 | 2.605437 | 1.754574 |
| CUST_19710_PI430048170 | 2.02E-02 | 3.33868 | -2.18043 | 1.752885 |
| CUST_94119_PI430048170 | 8.78E-04 | 5.169385 | 1.96803 | 1.751484 |
| CUST_47203_PI430048170 | 2.79E-03 | 4.484647 | 0.412591 | 1.751116 |
| CUST_66397_PI430048170 | 5.97E-04 | 5.404904 | 2.495901 | 1.750354 |
| CUST_65079_PI430048170 | 5.97E-04 | 5.404959 | 2.496024 | 1.750098 |
| CUST_12171_PI430048170 | 1.36E-02 | 3.574519 | -1.6562 | 1.750034 |
| CUST_91293_PI430048170 | 3.52E-03 | 4.353337 | 0.11248 | 1.748649 |
| CUST_49008_PI430048170 | 7.38E-03 | 3.927564 | -0.85894 | 1.747896 |
| CUST_85240_PI430048170 | 5.15E-04 | 5.496701 | 2.700291 | 1.746646 |
| CUST_12085_PI430048170 | 3.68E-04 | 5.70698 | 3.165297 | 1.746382 |
| CUST_94274_PI430048170 | 5.99E-05 | 6.958569 | 5.822847 | 1.746093 |
| CUST_64217_PI430048170 | 6.04E-05 | 6.952864 | 5.811207 | 1.745906 |
| CUST_123404_PI430048170 | 3.35E-03 | 4.380281 | 0.17407 | 1.745726 |
| CUST_142948_PI430048170 | 1.70E-02 | 3.442551 | -1.95059 | 1.745231 |
| CUST_35848_PI430048170 | 6.16E-05 | 6.937103 | 5.779024 | 1.744752 |
| CUST_33240_PI430048170 | 2.40E-05 | 7.667942 | 7.234208 | 1.744319 |
| CUST_79028_PI430048170 | 8.48E-05 | 6.695818 | 5.28204 | 1.744278 |
| CUST_81181_PI430048170 | 1.77E-03 | 4.753857 | 1.026789 | 1.743714 |
| CUST_78774_PI430048170 | 2.32E-03 | 4.592065 | 0.657916 | 1.743707 |
| CUST_142777_PI430048170 | 1.26E-04 | 6.404971 | 4.672403 | 1.743399 |
| CUST_70385_PI430048170 | 5.86E-03 | 4.060037 | -0.55736 | 1.74246 |
| CUST_22313_PI430048170 | 1.50E-05 | 8.033566 | 7.933492 | 1.741493 |
| CUST_130349_PI430048170 | 3.13E-06 | 9.388698 | 10.36038 | 1.740518 |
| CUST_56695_PI430048170 | 4.96E-03 | 4.155406 | -0.33977 | 1.74029 |
| CUST_141116_PI430048170 | 4.10E-03 | 4.264954 | -0.08953 | 1.739877 |
| CUST_53520_PI430048170 | 1.20E-02 | 3.648576 | -1.48999 | 1.739694 |
| CUST_106808_PI430048170 | 8.88E-04 | 5.162612 | 1.952781 | 1.739247 |
| CUST_59341_PI430048170 | 3.17E-05 | 7.450783 | 6.809787 | 1.73888 |
| CUST_60914_PI430048170 | 5.63E-05 | 7.000762 | 5.908794 | 1.736794 |
| CUST_19437_PI430048170 | 4.33E-05 | 7.204491 | 6.320253 | 1.735444 |
| CUST_25202_PI430048170 | 7.19E-05 | 6.811346 | 5.521004 | 1.73496 |
| CUST_20749_PI430048170 | 1.43E-03 | 4.880043 | 1.313712 | 1.73433 |
| CUST_13437_PI430048170 | 6.69E-05 | 6.862503 | 5.62623 | 1.734155 |
| CUST_22857_PI430048170 | 9.41E-03 | 3.789563 | -1.17193 | 1.732725 |
| CUST_121269_PI430048170 | 1.13E-04 | 6.47848 | 4.827552 | 1.732552 |
| CUST_65565_PI430048170 | 3.79E-06 | 9.207919 | 10.0513 | 1.731664 |
| CUST_9513_PI430048170 | 4.25E-03 | 4.24454 | -0.13618 | 1.729115 |
| CUST_117358_PI430048170 | 1.20E-03 | 4.982443 | 1.545912 | 1.72889 |
| CUST_112009_PI430048170 | 1.15E-04 | 6.46727 | 4.80394 | 1.728178 |
| CUST_18399_PI430048170 | 1.14E-04 | 6.473324 | 4.816694 | 1.726396 |
| CUST_129892_PI430048170 | 4.26E-04 | 5.615764 | 2.964148 | 1.726346 |
| CUST_86718_PI430048170 | 7.64E-05 | 6.768376 | 5.432337 | 1.725486 |
| CUST_5675_PI430048170 | 3.61E-05 | 7.339144 | 6.588966 | 1.724521 |
| CUST_99005_PI430048170 | 1.72E-03 | 4.769883 | 1.063271 | 1.722008 |
| CUST_134245_PI430048170 | 2.70E-02 | 3.165886 | -2.55841 | 1.721602 |
| CUST_69691_PI430048170 | 1.76E-05 | 7.903263 | 7.686478 | 1.721448 |
| CUST_35818_PI430048170 | 8.14E-06 | 8.559278 | 8.905472 | 1.720897 |
| CUST_75154_PI430048170 | 1.56E-02 | 3.491804 | -1.84101 | 1.720194 |
| CUST_102620_PI430048170 | 3.05E-04 | 5.824208 | 3.422472 | 1.720017 |
| CUST_4186_PI430048170 | 1.92E-02 | 3.368611 | -2.11439 | 1.719283 |
| CUST_136139_PI430048170 | 1.17E-02 | 3.661426 | -1.46109 | 1.718686 |
| CUST_143794_PI430048170 | 7.82E-04 | 5.24034 | 2.127559 | 1.718172 |
| CUST_27330_PI430048170 | 7.06E-04 | 5.299851 | 2.261035 | 1.717888 |
| CUST_110288_PI430048170 | 7.99E-04 | 5.227936 | 2.0997 | 1.717092 |
| CUST_56653_PI430048170 | 3.55E-04 | 5.733433 | 3.223462 | 1.715973 |
| CUST_71235_PI430048170 | 3.65E-04 | 5.713933 | 3.180593 | 1.714921 |
| CUST_12628_PI430048170 | 1.12E-03 | 5.025827 | 1.644092 | 1.712589 |
| CUST_125934_PI430048170 | 1.96E-02 | 3.35681 | -2.14045 | 1.709064 |
| CUST_93402_PI430048170 | 5.86E-03 | 4.060738 | -0.55576 | 1.70804 |
| CUST_7804_PI430048170 | 6.97E-05 | 6.835258 | 5.570234 | 1.70745 |
| CUST_102939_PI430048170 | 4.18E-03 | 4.2545 | -0.11342 | 1.707282 |
| CUST_28683_PI430048170 | 2.35E-04 | 5.982197 | 3.766585 | 1.707096 |
| CUST_122084_PI430048170 | 2.10E-02 | 3.315965 | -2.23045 | 1.706397 |
| CUST_4661_PI430048170 | 1.38E-03 | 4.900374 | 1.359863 | 1.706197 |
| CUST_80372_PI430048170 | 3.50E-05 | 7.367766 | 6.645749 | 1.705939 |
| CUST_97129_PI430048170 | 8.06E-06 | 8.580446 | 8.94379 | 1.70458 |
| CUST_91605_PI430048170 | 3.39E-03 | 4.373178 | 0.157835 | 1.703226 |
| CUST_43561_PI430048170 | 6.80E-05 | 6.853554 | 5.607849 | 1.702941 |
| CUST_103161_PI430048170 | 3.37E-05 | 7.402632 | 6.714763 | 1.702802 |
| CUST_79283_PI430048170 | 2.92E-05 | 7.515288 | 6.936565 | 1.700988 |
| CUST_19329_PI430048170 | 1.24E-02 | 3.627071 | -1.53832 | 1.699045 |
| CUST_136290_PI430048170 | 1.44E-03 | 4.873677 | 1.299256 | 1.698871 |
| CUST_111519_PI430048170 | 1.08E-02 | 3.708322 | -1.35545 | 1.698679 |
| CUST_37651_PI430048170 | 1.21E-03 | 4.975605 | 1.530425 | 1.697704 |
| CUST_124228_PI430048170 | 2.35E-02 | 3.249032 | -2.37727 | 1.697429 |
| CUST_74482_PI430048170 | 7.21E-03 | 3.940901 | -0.82862 | 1.697267 |
| CUST_25697_PI430048170 | 3.51E-03 | 4.354996 | 0.116273 | 1.6963 |
| CUST_104325_PI430048170 | 8.55E-05 | 6.690506 | 5.271008 | 1.696197 |
| CUST_117046_PI430048170 | 3.96E-04 | 5.660809 | 3.063591 | 1.695354 |
| CUST_136629_PI430048170 | 2.70E-05 | 7.564255 | 7.032405 | 1.695192 |
| CUST_138052_PI430048170 | 4.52E-03 | 4.209239 | -0.21683 | 1.695014 |
| CUST_44549_PI430048170 | 4.01E-02 | 2.927053 | -3.06982 | 1.694493 |
| CUST_98628_PI430048170 | 5.58E-04 | 5.450738 | 2.598053 | 1.694336 |
| CUST_128696_PI430048170 | 2.50E-03 | 4.549517 | 0.560774 | 1.692356 |
| CUST_56654_PI430048170 | 3.02E-04 | 5.82938 | 3.433784 | 1.691007 |
| CUST_80210_PI430048170 | 2.29E-03 | 4.601373 | 0.679161 | 1.688643 |
| CUST_115714_PI430048170 | 4.54E-04 | 5.578229 | 2.881122 | 1.68707 |
| CUST_38275_PI430048170 | 1.80E-03 | 4.742963 | 1.001982 | 1.686646 |
| CUST_143553_PI430048170 | 9.83E-05 | 6.583752 | 5.048489 | 1.684866 |
| CUST_5387_PI430048170 | 5.41E-04 | 5.470017 | 2.640961 | 1.684669 |
| CUST_19540_PI430048170 | 8.84E-03 | 3.825825 | -1.08983 | 1.684092 |
| CUST_111516_PI430048170 | 1.45E-02 | 3.535992 | -1.7424 | 1.683759 |
| CUST_58301_PI430048170 | 6.42E-03 | 4.007841 | -0.6763 | 1.683243 |
| CUST_127236_PI430048170 | 1.16E-03 | 5.005379 | 1.597832 | 1.683076 |
| CUST_65757_PI430048170 | 2.78E-03 | 4.48777 | 0.419727 | 1.682944 |
| CUST_136521_PI430048170 | 1.55E-03 | 4.834481 | 1.210203 | 1.681047 |
| CUST_22838_PI430048170 | 1.33E-02 | 3.587026 | -1.62818 | 1.679552 |
| CUST_38665_PI430048170 | 3.84E-04 | 5.680192 | 3.106316 | 1.679351 |
| CUST_63413_PI430048170 | 5.05E-05 | 7.083204 | 6.076004 | 1.679035 |
| CUST_23452_PI430048170 | 1.17E-03 | 4.996075 | 1.576774 | 1.67571 |
| CUST_4423_PI430048170 | 4.80E-05 | 7.127413 | 6.165274 | 1.674549 |
| CUST_55851_PI430048170 | 2.87E-06 | 9.465291 | 10.49001 | 1.67408 |
| CUST_73511_PI430048170 | 2.71E-04 | 5.893428 | 3.573598 | 1.673849 |
| CUST_137894_PI430048170 | 9.17E-07 | 10.45156 | 12.09081 | 1.673067 |
| CUST_89958_PI430048170 | 2.67E-03 | 4.511323 | 0.473536 | 1.672792 |
| CUST_92540_PI430048170 | 2.82E-03 | 4.479677 | 0.401235 | 1.672641 |
| CUST_139540_PI430048170 | 5.75E-03 | 4.070749 | -0.53294 | 1.671907 |
| CUST_6967_PI430048170 | 2.22E-05 | 7.734133 | 7.362228 | 1.671639 |
| CUST_134789_PI430048170 | 9.84E-04 | 5.101728 | 1.815549 | 1.670743 |
| CUST_7132_PI430048170 | 2.68E-02 | 3.171272 | -2.54672 | 1.668995 |
| CUST_76521_PI430048170 | 4.78E-03 | 4.177625 | -0.28904 | 1.668304 |
| CUST_100681_PI430048170 | 2.50E-02 | 3.210523 | -2.46134 | 1.666886 |
| CUST_64353_PI430048170 | 6.49E-04 | 5.354184 | 2.382632 | 1.666274 |
| CUST_16404_PI430048170 | 4.05E-03 | 4.271693 | -0.07413 | 1.665535 |
| CUST_35779_PI430048170 | 5.71E-04 | 5.436026 | 2.565287 | 1.665335 |
| CUST_96682_PI430048170 | 2.14E-02 | 3.305386 | -2.25371 | 1.665323 |
| CUST_103117_PI430048170 | 1.28E-04 | 6.395322 | 4.651985 | 1.664589 |
| CUST_126941_PI430048170 | 4.94E-03 | 4.157 | -0.33613 | 1.66418 |
| CUST_79077_PI430048170 | 1.03E-02 | 3.738445 | -1.28748 | 1.661603 |
| CUST_124468_PI430048170 | 2.95E-02 | 3.11479 | -2.66898 | 1.660845 |
| CUST_124541_PI430048170 | 4.54E-02 | 2.846734 | -3.2384 | 1.660001 |
| CUST_42572_PI430048170 | 3.30E-04 | 5.780061 | 3.325801 | 1.659721 |
| CUST_40354_PI430048170 | 6.84E-03 | 3.97033 | -0.76169 | 1.658599 |
| CUST_35667_PI430048170 | 5.88E-04 | 5.415135 | 2.518722 | 1.658591 |
| CUST_55471_PI430048170 | 8.48E-04 | 5.190414 | 2.01535 | 1.657195 |
| CUST_44229_PI430048170 | 2.29E-04 | 5.996308 | 3.797178 | 1.656867 |
| CUST_143237_PI430048170 | 1.39E-03 | 4.892497 | 1.341984 | 1.656439 |
| CUST_27642_PI430048170 | 8.28E-04 | 5.20456 | 2.047163 | 1.653324 |
| CUST_143490_PI430048170 | 7.80E-06 | 8.605307 | 8.988711 | 1.651942 |
| CUST_140755_PI430048170 | 2.29E-04 | 5.997304 | 3.799337 | 1.651432 |
| CUST_135395_PI430048170 | 1.64E-04 | 6.226198 | 4.29213 | 1.650439 |
| CUST_138112_PI430048170 | 1.52E-03 | 4.845374 | 1.23496 | 1.648761 |
| CUST_141220_PI430048170 | 8.89E-03 | 3.822992 | -1.09625 | 1.647514 |
| CUST_39948_PI430048170 | 1.70E-05 | 7.932405 | 7.741934 | 1.646741 |
| CUST_96404_PI430048170 | 3.85E-02 | 2.953559 | -3.01379 | 1.646729 |
| CUST_57093_PI430048170 | 3.49E-03 | 4.357776 | 0.122626 | 1.645798 |
| CUST_132670_PI430048170 | 2.32E-02 | 3.25804 | -2.35756 | 1.644894 |
| CUST_109998_PI430048170 | 9.63E-03 | 3.775546 | -1.20364 | 1.64447 |
| CUST_67758_PI430048170 | 3.40E-05 | 7.39317 | 6.696052 | 1.640817 |
| CUST_145297_PI430048170 | 2.08E-02 | 3.320425 | -2.22063 | 1.639802 |
| CUST_134552_PI430048170 | 3.58E-06 | 9.254507 | 10.13138 | 1.639395 |
| CUST_83682_PI430048170 | 4.53E-04 | 5.579643 | 2.884254 | 1.636523 |
| CUST_94538_PI430048170 | 1.10E-04 | 6.504117 | 4.881495 | 1.636368 |
| CUST_126548_PI430048170 | 2.28E-05 | 7.707159 | 7.310133 | 1.635637 |
| CUST_33617_PI430048170 | 9.03E-06 | 8.429181 | 8.66859 | 1.634748 |
| CUST_142650_PI430048170 | 2.58E-05 | 7.594582 | 7.091589 | 1.633259 |
| CUST_40296_PI430048170 | 3.22E-02 | 3.061021 | -2.78469 | 1.632732 |
| CUST_134360_PI430048170 | 4.32E-03 | 4.235978 | -0.15574 | 1.632027 |
| CUST_139703_PI430048170 | 3.11E-02 | 3.081093 | -2.74158 | 1.631399 |
| CUST_97801_PI430048170 | 4.38E-02 | 2.870671 | -3.18836 | 1.630044 |
| CUST_116029_PI430048170 | 6.67E-04 | 5.338296 | 2.347101 | 1.628511 |
| CUST_143465_PI430048170 | 5.36E-06 | 8.907159 | 9.527243 | 1.627575 |
| CUST_70124_PI430048170 | 2.97E-03 | 4.448115 | 0.329112 | 1.627398 |
| CUST_7665_PI430048170 | 8.58E-04 | 5.184242 | 2.001466 | 1.626385 |
| CUST_59410_PI430048170 | 3.31E-04 | 5.776985 | 3.319057 | 1.626081 |
| CUST_144024_PI430048170 | 1.11E-04 | 6.498098 | 4.868839 | 1.624495 |
| CUST_134712_PI430048170 | 4.42E-04 | 5.593531 | 2.914988 | 1.624433 |
| CUST_7632_PI430048170 | 4.75E-04 | 5.550421 | 2.81952 | 1.623793 |
| CUST_134668_PI430048170 | 3.76E-04 | 5.693214 | 3.134997 | 1.622879 |
| CUST_69396_PI430048170 | 5.06E-03 | 4.143158 | -0.36773 | 1.619858 |
| CUST_113470_PI430048170 | 2.17E-03 | 4.63259 | 0.750393 | 1.618342 |
| CUST_41184_PI430048170 | 1.25E-02 | 3.622842 | -1.54782 | 1.618334 |
| CUST_33424_PI430048170 | 1.02E-02 | 3.741846 | -1.2798 | 1.616365 |
| CUST_131729_PI430048170 | 6.43E-04 | 5.359781 | 2.395142 | 1.615692 |
| CUST_35460_PI430048170 | 5.87E-04 | 5.416448 | 2.521649 | 1.615446 |
| CUST_29125_PI430048170 | 4.54E-03 | 4.206339 | -0.22345 | 1.614309 |
| CUST_121718_PI430048170 | 6.49E-05 | 6.88617 | 5.674789 | 1.614133 |
| CUST_79749_PI430048170 | 1.09E-03 | 5.042001 | 1.680661 | 1.613598 |
| CUST_94220_PI430048170 | 2.07E-03 | 4.660696 | 0.814501 | 1.612145 |
| CUST_91241_PI430048170 | 6.54E-03 | 3.997594 | -0.69963 | 1.610051 |
| CUST_104096_PI430048170 | 1.25E-04 | 6.413636 | 4.690729 | 1.609752 |
| CUST_32600_PI430048170 | 4.43E-03 | 4.220063 | -0.1921 | 1.608493 |
| CUST_78142_PI430048170 | 3.95E-03 | 4.286685 | -0.03987 | 1.607815 |
| CUST_137535_PI430048170 | 3.14E-06 | 9.380019 | 10.34564 | 1.607306 |
| CUST_73364_PI430048170 | 2.83E-03 | 4.4777 | 0.396719 | 1.606946 |
| CUST_65755_PI430048170 | 2.28E-03 | 4.603098 | 0.683096 | 1.606706 |
| CUST_74969_PI430048170 | 1.38E-02 | 3.565398 | -1.67662 | 1.606472 |
| CUST_10994_PI430048170 | 5.33E-03 | 4.112755 | -0.43712 | 1.604449 |
| CUST_126000_PI430048170 | 7.74E-05 | 6.757969 | 5.410824 | 1.604096 |
| CUST_55672_PI430048170 | 5.11E-04 | 5.501817 | 2.711657 | 1.604074 |
| CUST_89255_PI430048170 | 2.00E-04 | 6.081773 | 3.981945 | 1.601359 |
| CUST_86874_PI430048170 | 1.42E-03 | 4.883521 | 1.321609 | 1.601174 |
| CUST_25462_PI430048170 | 1.93E-02 | 3.3666 | -2.11883 | 1.601135 |
| CUST_122120_PI430048170 | 1.03E-04 | 6.549525 | 4.976819 | 1.600654 |
| CUST_59638_PI430048170 | 1.91E-03 | 4.70843 | 0.923316 | 1.600581 |
| CUST_129650_PI430048170 | 5.77E-04 | 5.427426 | 2.546123 | 1.59982 |
| CUST_23952_PI430048170 | 1.19E-05 | 8.197161 | 8.240174 | 1.599446 |
| CUST_72786_PI430048170 | 2.00E-03 | 4.679976 | 0.858462 | 1.599259 |
| CUST_127404_PI430048170 | 6.41E-04 | 5.361253 | 2.398431 | 1.599208 |
| CUST_128612_PI430048170 | 1.38E-06 | 10.08175 | 11.50518 | 1.59919 |
| CUST_135464_PI430048170 | 1.53E-02 | 3.503411 | -1.81513 | 1.599004 |
| CUST_73462_PI430048170 | 7.14E-05 | 6.818052 | 5.534818 | 1.59874 |
| CUST_92175_PI430048170 | 4.38E-03 | 4.227794 | -0.17444 | 1.597978 |
| CUST_143118_PI430048170 | 1.16E-02 | 3.668905 | -1.44426 | 1.597849 |
| CUST_34285_PI430048170 | 8.42E-04 | 5.19446 | 2.024453 | 1.59713 |
| CUST_111996_PI430048170 | 2.30E-02 | 3.262458 | -2.34789 | 1.596557 |
| CUST_46886_PI430048170 | 1.01E-04 | 6.561622 | 5.002169 | 1.596179 |
| CUST_99391_PI430048170 | 1.86E-04 | 6.131789 | 4.089661 | 1.596135 |
| CUST_145077_PI430048170 | 1.53E-02 | 3.501917 | -1.81846 | 1.595412 |
| CUST_52515_PI430048170 | 2.02E-05 | 7.808631 | 7.505559 | 1.594715 |
| CUST_112069_PI430048170 | 1.61E-03 | 4.809348 | 1.153061 | 1.593791 |
| CUST_58252_PI430048170 | 3.05E-03 | 4.434558 | 0.298129 | 1.593343 |
| CUST_105992_PI430048170 | 1.37E-04 | 6.345861 | 4.547126 | 1.592672 |
| CUST_70744_PI430048170 | 1.26E-03 | 4.951046 | 1.474784 | 1.592331 |
| CUST_28976_PI430048170 | 4.39E-04 | 5.597764 | 2.924351 | 1.591191 |
| CUST_5215_PI430048170 | 2.83E-03 | 4.476665 | 0.394354 | 1.590018 |
| CUST_26179_PI430048170 | 1.49E-04 | 6.288608 | 4.425354 | 1.589945 |
| CUST_125559_PI430048170 | 3.66E-04 | 5.711459 | 3.175151 | 1.588511 |
| CUST_126920_PI430048170 | 1.42E-04 | 6.322212 | 4.496878 | 1.588503 |
| CUST_127814_PI430048170 | 7.03E-03 | 3.955515 | -0.79539 | 1.587635 |
| CUST_72869_PI430048170 | 1.21E-03 | 4.978102 | 1.536081 | 1.587232 |
| CUST_135138_PI430048170 | 3.87E-06 | 9.182699 | 10.00783 | 1.587033 |
| CUST_128169_PI430048170 | 1.04E-06 | 10.33479 | 11.90775 | 1.586924 |
| CUST_92610_PI430048170 | 1.51E-03 | 4.847471 | 1.239725 | 1.583764 |
| CUST_132072_PI430048170 | 5.49E-05 | 7.019956 | 5.947808 | 1.583403 |
| CUST_52831_PI430048170 | 7.42E-03 | 3.924048 | -0.86693 | 1.583192 |
| CUST_47753_PI430048170 | 7.01E-05 | 6.831342 | 5.562179 | 1.581898 |
| CUST_110537_PI430048170 | 2.34E-03 | 4.587857 | 0.64831 | 1.580104 |
| CUST_5030_PI430048170 | 1.24E-03 | 4.961828 | 1.499217 | 1.579865 |
| CUST_5876_PI430048170 | 2.61E-02 | 3.18648 | -2.51368 | 1.579158 |
| CUST_127684_PI430048170 | 1.85E-02 | 3.393398 | -2.05958 | 1.578053 |
| CUST_131280_PI430048170 | 3.07E-04 | 5.819646 | 3.412493 | 1.575877 |
| CUST_57338_PI430048170 | 1.84E-05 | 7.8728 | 7.628379 | 1.574262 |
| CUST_143908_PI430048170 | 4.29E-04 | 5.612859 | 2.957728 | 1.573843 |
| CUST_24804_PI430048170 | 3.30E-02 | 3.046544 | -2.81573 | 1.573666 |
| CUST_27675_PI430048170 | 1.84E-03 | 4.732404 | 0.977934 | 1.57268 |
| CUST_55163_PI430048170 | 3.22E-02 | 3.06226 | -2.78203 | 1.571504 |
| CUST_69886_PI430048170 | 6.23E-04 | 5.37902 | 2.438126 | 1.571091 |
| CUST_124232_PI430048170 | 1.99E-02 | 3.34828 | -2.15927 | 1.569141 |
| CUST_120043_PI430048170 | 1.61E-02 | 3.475009 | -1.87842 | 1.567255 |
| CUST_128267_PI430048170 | 3.33E-04 | 5.773375 | 3.311143 | 1.566145 |
| CUST_142860_PI430048170 | 2.28E-05 | 7.712039 | 7.319566 | 1.565983 |
| CUST_46932_PI430048170 | 3.06E-04 | 5.821411 | 3.416354 | 1.56388 |
| CUST_34054_PI430048170 | 2.91E-03 | 4.459601 | 0.355361 | 1.563788 |
| CUST_53159_PI430048170 | 4.38E-03 | 4.227818 | -0.17438 | 1.563452 |
| CUST_76116_PI430048170 | 1.42E-02 | 3.548106 | -1.71531 | 1.562857 |
| CUST_41167_PI430048170 | 4.96E-03 | 4.154731 | -0.34131 | 1.561909 |
| CUST_31154_PI430048170 | 1.18E-02 | 3.656701 | -1.47172 | 1.561442 |
| CUST_41732_PI430048170 | 1.69E-04 | 6.204729 | 4.246186 | 1.561297 |
| CUST_140307_PI430048170 | 2.30E-04 | 5.994417 | 3.79308 | 1.560096 |
| CUST_18824_PI430048170 | 6.45E-03 | 4.005242 | -0.68221 | 1.559649 |
| CUST_11346_PI430048170 | 1.51E-05 | 8.015969 | 7.900275 | 1.559487 |
| CUST_25281_PI430048170 | 2.13E-03 | 4.644077 | 0.776598 | 1.559067 |
| CUST_70399_PI430048170 | 2.85E-04 | 5.864606 | 3.510738 | 1.558317 |
| CUST_78899_PI430048170 | 2.18E-04 | 6.027361 | 3.864415 | 1.557824 |
| CUST_141384_PI430048170 | 2.20E-03 | 4.625126 | 0.733364 | 1.556246 |
| CUST_48950_PI430048170 | 1.67E-02 | 3.45387 | -1.92544 | 1.555978 |
| CUST_141944_PI430048170 | 8.15E-05 | 6.724861 | 5.342286 | 1.555517 |
| CUST_85567_PI430048170 | 1.91E-02 | 3.372489 | -2.10582 | 1.555123 |
| CUST_137766_PI430048170 | 9.88E-04 | 5.09932 | 1.810116 | 1.554731 |
| CUST_102857_PI430048170 | 1.19E-03 | 4.989104 | 1.560994 | 1.554594 |
| CUST_142250_PI430048170 | 4.16E-05 | 7.234557 | 6.380477 | 1.554521 |
| CUST_71372_PI430048170 | 1.14E-04 | 6.477014 | 4.824467 | 1.553932 |
| CUST_66400_PI430048170 | 5.43E-06 | 8.897746 | 9.510641 | 1.553232 |
| CUST_74490_PI430048170 | 6.55E-03 | 3.995713 | -0.70391 | 1.552429 |
| CUST_55606_PI430048170 | 5.49E-05 | 7.019478 | 5.946837 | 1.552123 |
| CUST_24307_PI430048170 | 1.73E-03 | 4.766783 | 1.056214 | 1.550544 |
| CUST_48652_PI430048170 | 1.51E-04 | 6.27812 | 4.403 | 1.550322 |
| CUST_144988_PI430048170 | 1.58E-02 | 3.486008 | -1.85392 | 1.55003 |
| CUST_118438_PI430048170 | 2.56E-03 | 4.536065 | 0.530051 | 1.549245 |
| CUST_142539_PI430048170 | 4.91E-02 | 2.797792 | -3.34017 | 1.549053 |
| CUST_144851_PI430048170 | 3.05E-05 | 7.480593 | 6.868451 | 1.547698 |
| CUST_96625_PI430048170 | 1.36E-04 | 6.351611 | 4.559333 | 1.546554 |
| CUST_60349_PI430048170 | 1.78E-02 | 3.414336 | -2.0132 | 1.546305 |
| CUST_6279_PI430048170 | 1.67E-02 | 3.453265 | -1.92678 | 1.545044 |
| CUST_27932_PI430048170 | 3.21E-03 | 4.405248 | 0.231139 | 1.544497 |
| CUST_104107_PI430048170 | 1.47E-04 | 6.294883 | 4.43872 | 1.544032 |
| CUST_29455_PI430048170 | 2.99E-06 | 9.433126 | 10.43566 | 1.543734 |
| CUST_41038_PI430048170 | 3.01E-02 | 3.101701 | -2.69721 | 1.54282 |
| CUST_116962_PI430048170 | 4.48E-02 | 2.855509 | -3.22007 | 1.5427 |
| CUST_73241_PI430048170 | 3.49E-02 | 3.0125 | -2.8885 | 1.541003 |
| CUST_105527_PI430048170 | 9.78E-04 | 5.106167 | 1.825563 | 1.540837 |
| CUST_16904_PI430048170 | 2.84E-02 | 3.136061 | -2.62302 | 1.539201 |
| CUST_55441_PI430048170 | 2.96E-05 | 7.503633 | 6.913702 | 1.538944 |
| CUST_143876_PI430048170 | 1.86E-04 | 6.13664 | 4.100091 | 1.538733 |
| CUST_143845_PI430048170 | 3.58E-03 | 4.343449 | 0.089878 | 1.537823 |
| CUST_62570_PI430048170 | 2.94E-03 | 4.454536 | 0.343786 | 1.537366 |
| CUST_20198_PI430048170 | 9.35E-04 | 5.132567 | 1.885095 | 1.536944 |
| CUST_115309_PI430048170 | 2.32E-02 | 3.258066 | -2.3575 | 1.536542 |
| CUST_121676_PI430048170 | 2.63E-04 | 5.912807 | 3.615807 | 1.536236 |
| CUST_8104_PI430048170 | 2.01E-04 | 6.075875 | 3.969225 | 1.536178 |
| CUST_28829_PI430048170 | 2.26E-02 | 3.271952 | -2.32709 | 1.535834 |
| CUST_767_PI430048170 | 1.43E-02 | 3.544382 | -1.72364 | 1.534918 |
| CUST_13417_PI430048170 | 3.35E-03 | 4.380768 | 0.175183 | 1.532514 |
| CUST_64850_PI430048170 | 2.20E-02 | 3.289874 | -2.28778 | 1.531714 |
| CUST_82297_PI430048170 | 1.25E-06 | 10.18865 | 11.67624 | 1.531297 |
| CUST_127771_PI430048170 | 2.32E-04 | 5.988407 | 3.780052 | 1.530861 |
| CUST_58709_PI430048170 | 5.55E-03 | 4.090821 | -0.48716 | 1.530379 |
| CUST_128566_PI430048170 | 5.14E-05 | 7.071046 | 6.051404 | 1.530251 |
| CUST_113821_PI430048170 | 2.59E-04 | 5.922043 | 3.63591 | 1.529671 |
| CUST_105277_PI430048170 | 2.06E-05 | 7.792953 | 7.475462 | 1.529334 |
| CUST_109618_PI430048170 | 8.38E-03 | 3.855229 | -1.02318 | 1.529121 |
| CUST_133779_PI430048170 | 5.09E-03 | 4.140246 | -0.37438 | 1.529012 |
| CUST_35621_PI430048170 | 1.55E-03 | 4.832973 | 1.206776 | 1.528773 |
| CUST_86673_PI430048170 | 1.24E-03 | 4.965392 | 1.507292 | 1.527914 |
| CUST_35528_PI430048170 | 2.54E-04 | 5.935068 | 3.664241 | 1.527846 |
| CUST_25933_PI430048170 | 6.91E-04 | 5.314951 | 2.294856 | 1.527789 |
| CUST_104111_PI430048170 | 2.89E-05 | 7.521369 | 6.948486 | 1.527678 |
| CUST_122592_PI430048170 | 2.64E-03 | 4.516571 | 0.485525 | 1.527574 |
| CUST_22163_PI430048170 | 9.52E-03 | 3.78257 | -1.18776 | 1.527483 |
| CUST_15379_PI430048170 | 2.98E-04 | 5.836514 | 3.449379 | 1.526296 |
| CUST_128094_PI430048170 | 2.57E-04 | 5.928756 | 3.650514 | 1.52534 |
| CUST_28741_PI430048170 | 8.18E-05 | 6.721798 | 5.335937 | 1.524655 |
| CUST_80193_PI430048170 | 1.08E-04 | 6.51932 | 4.913442 | 1.524462 |
| CUST_91256_PI430048170 | 6.19E-03 | 4.028956 | -0.6282 | 1.524339 |
| CUST_127600_PI430048170 | 7.09E-04 | 5.296774 | 2.254141 | 1.524042 |
| CUST_130798_PI430048170 | 5.13E-04 | 5.49998 | 2.707576 | 1.523605 |
| CUST_23945_PI430048170 | 1.57E-05 | 7.992254 | 7.855441 | 1.522491 |
| CUST_49461_PI430048170 | 2.24E-02 | 3.277945 | -2.31395 | 1.521581 |
| CUST_6818_PI430048170 | 2.68E-03 | 4.507713 | 0.46529 | 1.521503 |
| CUST_35417_PI430048170 | 1.81E-04 | 6.159908 | 4.150082 | 1.519482 |
| CUST_122658_PI430048170 | 1.25E-05 | 8.167689 | 8.185206 | 1.518188 |
| CUST_137076_PI430048170 | 3.13E-02 | 3.077792 | -2.74868 | 1.517218 |
| CUST_40509_PI430048170 | 6.05E-03 | 4.042423 | -0.59751 | 1.516984 |
| CUST_120729_PI430048170 | 9.78E-04 | 5.106504 | 1.826323 | 1.516309 |
| CUST_25992_PI430048170 | 4.77E-02 | 2.815362 | -3.30372 | 1.515532 |
| CUST_104614_PI430048170 | 1.00E-03 | 5.088429 | 1.785535 | 1.514815 |
| CUST_115373_PI430048170 | 6.97E-05 | 6.835391 | 5.570509 | 1.514022 |
| CUST_135353_PI430048170 | 2.01E-03 | 4.676678 | 0.850943 | 1.513138 |
| CUST_128743_PI430048170 | 1.99E-04 | 6.084576 | 3.987991 | 1.512985 |
| CUST_61146_PI430048170 | 2.06E-02 | 3.327045 | -2.20606 | 1.512693 |
| CUST_102804_PI430048170 | 4.37E-03 | 4.2287 | -0.17237 | 1.512099 |
| CUST_86664_PI430048170 | 1.17E-03 | 4.997527 | 1.580061 | 1.512083 |
| CUST_88745_PI430048170 | 3.44E-05 | 7.382366 | 6.67467 | 1.511916 |
| CUST_63322_PI430048170 | 1.03E-04 | 6.547437 | 4.972444 | 1.511591 |
| CUST_13423_PI430048170 | 4.78E-04 | 5.545903 | 2.809503 | 1.510207 |
| CUST_97951_PI430048170 | 3.84E-05 | 7.29001 | 6.491213 | 1.509849 |
| CUST_129417_PI430048170 | 1.64E-02 | 3.464401 | -1.90202 | 1.509249 |
| CUST_108427_PI430048170 | 1.70E-02 | 3.444102 | -1.94715 | 1.509104 |
| CUST_129487_PI430048170 | 2.24E-03 | 4.613971 | 0.707912 | 1.507899 |
| CUST_123816_PI430048170 | 5.96E-03 | 4.050992 | -0.57798 | 1.507132 |
| CUST_35964_PI430048170 | 2.80E-02 | 3.145017 | -2.60364 | 1.507068 |
| CUST_94467_PI430048170 | 6.23E-05 | 6.920741 | 5.745579 | 1.506043 |
| CUST_2819_PI430048170 | 2.77E-02 | 3.150635 | -2.59147 | 1.505114 |
| CUST_24339_PI430048170 | 3.56E-02 | 3.000447 | -2.9142 | 1.504151 |
| CUST_138497_PI430048170 | 4.72E-02 | 2.822539 | -3.2888 | 1.503839 |
| CUST_144429_PI430048170 | 1.07E-05 | 8.290107 | 8.412713 | 1.502253 |
| CUST_117792_PI430048170 | 1.67E-02 | 3.453536 | -1.92618 | 1.501763 |
| CUST_134935_PI430048170 | 1.23E-03 | 4.967408 | 1.511858 | 1.501601 |
| CUST_97273_PI430048170 | 5.95E-03 | 4.051387 | -0.57708 | 1.501179 |
| CUST_113112_PI430048170 | 6.67E-04 | 5.337188 | 2.344624 | 1.501024 |
| CUST_50332_PI430048170 | 1.41E-02 | 3.552788 | -1.70484 | 1.500846 |
| CUST_138876_PI430048170 | 2.01E-02 | 3.342829 | -2.17129 | 1.500827 |
| CUST_129579_PI430048170 | 5.96E-03 | 4.050558 | -0.57897 | 1.500779 |
| CUST_85683_PI430048170 | 1.12E-02 | 3.684811 | -1.40844 | 1.500506 |
| CUST_71920_PI430048170 | 3.52E-04 | 5.739664 | 3.237153 | 1.500174 |
| CUST_67584_PI430048170 | 2.62E-03 | 4.522018 | 0.497968 | 1.499988 |
| CUST_133021_PI430048170 | 5.33E-03 | 4.113017 | -0.43652 | 1.499247 |
| CUST_128387_PI430048170 | 1.16E-03 | 5.00146 | 1.588963 | 1.499079 |
| CUST_132139_PI430048170 | 2.75E-04 | 5.884117 | 3.553302 | 1.498819 |
| CUST_35784_PI430048170 | 4.30E-03 | 4.238956 | -0.14894 | 1.497862 |
| CUST_128515_PI430048170 | 2.69E-03 | 4.505818 | 0.46096 | 1.497033 |
| CUST_118930_PI430048170 | 2.44E-04 | 5.959418 | 3.717153 | 1.496586 |
| CUST_112209_PI430048170 | 1.83E-03 | 4.73536 | 0.984667 | 1.495419 |
| CUST_124230_PI430048170 | 2.91E-02 | 3.121501 | -2.6545 | 1.49527 |
| CUST_95090_PI430048170 | 1.14E-02 | 3.678196 | -1.42334 | 1.494411 |
| CUST_75958_PI430048170 | 7.46E-03 | 3.921825 | -0.87199 | 1.494049 |
| CUST_45091_PI430048170 | 2.33E-02 | 3.254382 | -2.36557 | 1.493783 |
| CUST_106116_PI430048170 | 6.28E-04 | 5.375036 | 2.429228 | 1.492549 |
| CUST_89156_PI430048170 | 4.16E-05 | 7.236081 | 6.383525 | 1.492355 |
| CUST_118590_PI430048170 | 4.27E-06 | 9.098263 | 9.861668 | 1.490101 |
| CUST_48812_PI430048170 | 2.91E-02 | 3.121936 | -2.65356 | 1.489616 |
| CUST_119874_PI430048170 | 1.41E-03 | 4.885407 | 1.32589 | 1.48919 |
| CUST_107103_PI430048170 | 1.28E-04 | 6.392457 | 4.645919 | 1.488031 |
| CUST_36474_PI430048170 | 4.72E-04 | 5.555058 | 2.829797 | 1.486917 |
| CUST_98722_PI430048170 | 9.25E-04 | 5.138143 | 1.897661 | 1.486413 |
| CUST_118588_PI430048170 | 1.10E-05 | 8.267858 | 8.371524 | 1.48635 |
| CUST_52446_PI430048170 | 2.49E-03 | 4.550674 | 0.563416 | 1.486222 |
| CUST_138156_PI430048170 | 1.31E-03 | 4.929657 | 1.426294 | 1.485598 |
| CUST_111423_PI430048170 | 4.97E-05 | 7.097028 | 6.103948 | 1.48473 |
| CUST_97622_PI430048170 | 2.70E-03 | 4.503287 | 0.455179 | 1.484114 |
| CUST_133647_PI430048170 | 7.07E-07 | 10.73255 | 12.52447 | 1.484085 |
| CUST_74086_PI430048170 | 8.74E-04 | 5.173247 | 1.976722 | 1.484078 |
| CUST_94422_PI430048170 | 4.00E-03 | 4.2803 | -0.05446 | 1.484067 |
| CUST_49830_PI430048170 | 1.47E-03 | 4.865381 | 1.280415 | 1.483814 |
| CUST_142626_PI430048170 | 1.03E-04 | 6.54929 | 4.976327 | 1.483681 |
| CUST_103071_PI430048170 | 1.73E-03 | 4.766945 | 1.056584 | 1.483672 |
| CUST_6660_PI430048170 | 1.87E-02 | 3.384589 | -2.07907 | 1.483635 |
| CUST_120741_PI430048170 | 1.68E-04 | 6.208072 | 4.253345 | 1.482352 |
| CUST_19434_PI430048170 | 2.73E-03 | 4.497629 | 0.442252 | 1.481554 |
| CUST_84195_PI430048170 | 1.63E-03 | 4.801225 | 1.134586 | 1.481009 |
| CUST_131643_PI430048170 | 1.52E-03 | 4.844216 | 1.232328 | 1.480894 |
| CUST_35519_PI430048170 | 2.44E-05 | 7.65407 | 7.207299 | 1.480502 |
| CUST_71090_PI430048170 | 1.15E-06 | 10.25035 | 11.7743 | 1.478652 |
| CUST_37810_PI430048170 | 5.09E-05 | 7.077443 | 6.06435 | 1.478652 |
| CUST_135596_PI430048170 | 2.42E-03 | 4.567447 | 0.601714 | 1.478487 |
| CUST_133500_PI430048170 | 1.09E-02 | 3.700207 | -1.37375 | 1.478382 |
| CUST_57277_PI430048170 | 4.61E-03 | 4.197678 | -0.24324 | 1.477698 |
| CUST_128654_PI430048170 | 9.13E-03 | 3.807601 | -1.13111 | 1.47696 |
| CUST_106327_PI430048170 | 4.26E-02 | 2.889427 | -3.14903 | 1.476035 |
| CUST_21452_PI430048170 | 5.15E-05 | 7.068857 | 6.046974 | 1.475496 |
| CUST_17082_PI430048170 | 1.12E-02 | 3.689381 | -1.39815 | 1.475221 |
| CUST_76240_PI430048170 | 3.57E-06 | 9.262236 | 10.14463 | 1.473932 |
| CUST_74202_PI430048170 | 2.53E-02 | 3.203012 | -2.47771 | 1.473469 |
| CUST_99129_PI430048170 | 4.09E-02 | 2.914032 | -3.09728 | 1.472821 |
| CUST_69148_PI430048170 | 1.13E-04 | 6.481773 | 4.834486 | 1.471049 |
| CUST_144906_PI430048170 | 1.72E-02 | 3.436206 | -1.96468 | 1.471016 |
| CUST_130795_PI430048170 | 2.15E-02 | 3.302523 | -2.26 | 1.470225 |
| CUST_37207_PI430048170 | 1.92E-02 | 3.369347 | -2.11276 | 1.469392 |
| CUST_99243_PI430048170 | 8.76E-05 | 6.673341 | 5.235334 | 1.468553 |
| CUST_94807_PI430048170 | 2.63E-04 | 5.913527 | 3.617375 | 1.467488 |
| CUST_44408_PI430048170 | 6.38E-03 | 4.012223 | -0.66631 | 1.467093 |
| CUST_9158_PI430048170 | 5.20E-05 | 7.060863 | 6.030786 | 1.466417 |
| CUST_81519_PI430048170 | 6.09E-03 | 4.039058 | -0.60518 | 1.46614 |
| CUST_25199_PI430048170 | 2.12E-03 | 4.647225 | 0.783777 | 1.465447 |
| CUST_133278_PI430048170 | 9.14E-05 | 6.641498 | 5.169048 | 1.464784 |
| CUST_73660_PI430048170 | 1.94E-04 | 6.10056 | 4.022442 | 1.464482 |
| CUST_88368_PI430048170 | 2.35E-02 | 3.24991 | -2.37535 | 1.464332 |
| CUST_129972_PI430048170 | 4.63E-04 | 5.567572 | 2.857522 | 1.46424 |
| CUST_49523_PI430048170 | 1.51E-02 | 3.510725 | -1.79882 | 1.463993 |
| CUST_66382_PI430048170 | 2.29E-03 | 4.599727 | 0.675405 | 1.463702 |
| CUST_133926_PI430048170 | 1.03E-05 | 8.335977 | 8.497411 | 1.462695 |
| CUST_80245_PI430048170 | 4.91E-03 | 4.161377 | -0.32614 | 1.462601 |
| CUST_136267_PI430048170 | 6.27E-04 | 5.376486 | 2.432466 | 1.461994 |
| CUST_63477_PI430048170 | 3.04E-06 | 9.413026 | 10.40164 | 1.46126 |
| CUST_21093_PI430048170 | 5.23E-03 | 4.123702 | -0.41214 | 1.459532 |
| CUST_126805_PI430048170 | 4.47E-05 | 7.177373 | 6.265823 | 1.459409 |
| CUST_93628_PI430048170 | 9.42E-03 | 3.78866 | -1.17398 | 1.459362 |
| CUST_3800_PI430048170 | 4.17E-06 | 9.116388 | 9.893125 | 1.458465 |
| CUST_112008_PI430048170 | 1.14E-02 | 3.676624 | -1.42688 | 1.458364 |
| CUST_141249_PI430048170 | 2.88E-04 | 5.857598 | 3.495439 | 1.457427 |
| CUST_134194_PI430048170 | 4.42E-03 | 4.222456 | -0.18664 | 1.457182 |
| CUST_88795_PI430048170 | 5.35E-05 | 7.03829 | 5.985027 | 1.456938 |
| CUST_38074_PI430048170 | 7.28E-04 | 5.281558 | 2.220038 | 1.456067 |
| CUST_140947_PI430048170 | 2.33E-02 | 3.252668 | -2.36932 | 1.455622 |
| CUST_23953_PI430048170 | 1.51E-05 | 8.028363 | 7.923675 | 1.455586 |
| CUST_127410_PI430048170 | 6.23E-03 | 4.026048 | -0.63482 | 1.454871 |
| CUST_97572_PI430048170 | 1.47E-02 | 3.525572 | -1.76567 | 1.45409 |
| CUST_109720_PI430048170 | 1.47E-02 | 3.528103 | -1.76002 | 1.453677 |
| CUST_85192_PI430048170 | 1.25E-03 | 4.958552 | 1.491794 | 1.453101 |
| CUST_142451_PI430048170 | 1.07E-03 | 5.052858 | 1.705199 | 1.453085 |
| CUST_115915_PI430048170 | 1.41E-04 | 6.327163 | 4.507405 | 1.452903 |
| CUST_22354_PI430048170 | 3.61E-03 | 4.338391 | 0.078316 | 1.452304 |
| CUST_88078_PI430048170 | 1.21E-04 | 6.436981 | 4.740051 | 1.452189 |
| CUST_70996_PI430048170 | 1.48E-03 | 4.860193 | 1.26863 | 1.452013 |
| CUST_13292_PI430048170 | 2.14E-04 | 6.036902 | 3.88505 | 1.451516 |
| CUST_42635_PI430048170 | 1.72E-03 | 4.77106 | 1.065951 | 1.451516 |
| CUST_47979_PI430048170 | 3.93E-03 | 4.288905 | -0.03479 | 1.449355 |
| CUST_122130_PI430048170 | 5.35E-03 | 4.110829 | -0.44151 | 1.449145 |
| CUST_117948_PI430048170 | 6.54E-05 | 6.877958 | 5.657949 | 1.449099 |
| CUST_30099_PI430048170 | 4.63E-03 | 4.195397 | -0.24845 | 1.448847 |
| CUST_99605_PI430048170 | 3.97E-03 | 4.284112 | -0.04575 | 1.448812 |
| CUST_130059_PI430048170 | 6.27E-05 | 6.914851 | 5.73353 | 1.448682 |
| CUST_137831_PI430048170 | 3.49E-03 | 4.357205 | 0.121321 | 1.448552 |
| CUST_40340_PI430048170 | 3.52E-04 | 5.738076 | 3.233664 | 1.448282 |
| CUST_126854_PI430048170 | 2.00E-03 | 4.680961 | 0.860707 | 1.448028 |
| CUST_86579_PI430048170 | 4.07E-03 | 4.26982 | -0.07841 | 1.447455 |
| CUST_135694_PI430048170 | 9.40E-03 | 3.790103 | -1.17071 | 1.447309 |
| CUST_7436_PI430048170 | 2.13E-02 | 3.306788 | -2.25063 | 1.447025 |
| CUST_99673_PI430048170 | 6.54E-03 | 3.99733 | -0.70023 | 1.446909 |
| CUST_28830_PI430048170 | 3.69E-02 | 2.978405 | -2.96109 | 1.446639 |
| CUST_132246_PI430048170 | 4.70E-06 | 9.022266 | 9.729276 | 1.446563 |
| CUST_129940_PI430048170 | 1.37E-04 | 6.343648 | 4.542429 | 1.446417 |
| CUST_79851_PI430048170 | 3.47E-05 | 7.376898 | 6.663844 | 1.445433 |
| CUST_64491_PI430048170 | 1.60E-03 | 4.813023 | 1.161418 | 1.444664 |
| CUST_58823_PI430048170 | 2.20E-03 | 4.625047 | 0.733184 | 1.44323 |
| CUST_35459_PI430048170 | 1.85E-04 | 6.145417 | 4.118957 | 1.44309 |
| CUST_131329_PI430048170 | 3.22E-03 | 4.403759 | 0.227736 | 1.442961 |
| CUST_136642_PI430048170 | 1.50E-04 | 6.282456 | 4.412243 | 1.44291 |
| CUST_62700_PI430048170 | 7.29E-03 | 3.934356 | -0.8435 | 1.44252 |
| CUST_17346_PI430048170 | 1.21E-02 | 3.64083 | -1.50741 | 1.442327 |
| CUST_83026_PI430048170 | 9.97E-04 | 5.0938 | 1.797658 | 1.442195 |
| CUST_74239_PI430048170 | 3.20E-02 | 3.065974 | -2.77406 | 1.44192 |
| CUST_141992_PI430048170 | 3.42E-03 | 4.36871 | 0.147621 | 1.439851 |
| CUST_33686_PI430048170 | 3.69E-03 | 4.325661 | 0.049218 | 1.439783 |
| CUST_53174_PI430048170 | 6.00E-03 | 4.046974 | -0.58714 | 1.438728 |
| CUST_136485_PI430048170 | 3.66E-05 | 7.325267 | 6.561393 | 1.43842 |
| CUST_67292_PI430048170 | 8.15E-04 | 5.215576 | 2.071926 | 1.438084 |
| CUST_111520_PI430048170 | 1.88E-02 | 3.38295 | -2.08269 | 1.438036 |
| CUST_131242_PI430048170 | 1.90E-03 | 4.713268 | 0.934339 | 1.437812 |
| CUST_35402_PI430048170 | 8.22E-05 | 6.717797 | 5.327643 | 1.437801 |
| CUST_114996_PI430048170 | 1.72E-03 | 4.770852 | 1.065476 | 1.437762 |
| CUST_36661_PI430048170 | 7.66E-03 | 3.906752 | -0.90623 | 1.436169 |
| CUST_46738_PI430048170 | 1.49E-02 | 3.517284 | -1.78418 | 1.435693 |
| CUST_127733_PI430048170 | 2.56E-05 | 7.600494 | 7.103112 | 1.43554 |
| CUST_123319_PI430048170 | 7.30E-03 | 3.933593 | -0.84524 | 1.435496 |
| CUST_87659_PI430048170 | 4.04E-02 | 2.921645 | -3.08123 | 1.435296 |
| CUST_15087_PI430048170 | 1.81E-02 | 3.403739 | -2.03668 | 1.434391 |
| CUST_70512_PI430048170 | 8.74E-04 | 5.17241 | 1.974839 | 1.433871 |
| CUST_114040_PI430048170 | 5.01E-05 | 7.090839 | 6.091442 | 1.43364 |
| CUST_115535_PI430048170 | 1.43E-04 | 6.315161 | 4.481882 | 1.43303 |
| CUST_140851_PI430048170 | 1.43E-07 | 12.34851 | 14.84091 | 1.431753 |
| CUST_23854_PI430048170 | 8.22E-03 | 3.866364 | -0.99792 | 1.431454 |
| CUST_125676_PI430048170 | 2.16E-05 | 7.75333 | 7.399238 | 1.431344 |
| CUST_68154_PI430048170 | 2.37E-03 | 4.580599 | 0.631743 | 1.431153 |
| CUST_95873_PI430048170 | 9.48E-03 | 3.784831 | -1.18264 | 1.430493 |
| CUST_58728_PI430048170 | 1.17E-02 | 3.661843 | -1.46015 | 1.429974 |
| CUST_19870_PI430048170 | 2.63E-04 | 5.912763 | 3.615712 | 1.428571 |
| CUST_24091_PI430048170 | 6.13E-05 | 6.939975 | 5.784891 | 1.428419 |
| CUST_19531_PI430048170 | 6.75E-03 | 3.979342 | -0.74118 | 1.426866 |
| CUST_33579_PI430048170 | 2.26E-02 | 3.271524 | -2.32803 | 1.426857 |
| CUST_64853_PI430048170 | 1.84E-02 | 3.394766 | -2.05655 | 1.426702 |
| CUST_20220_PI430048170 | 1.49E-02 | 3.518176 | -1.78219 | 1.423107 |
| CUST_42603_PI430048170 | 4.00E-02 | 2.929799 | -3.06403 | 1.422123 |
| CUST_28812_PI430048170 | 1.99E-03 | 4.685553 | 0.871175 | 1.422046 |
| CUST_30100_PI430048170 | 9.16E-04 | 5.143576 | 1.909905 | 1.421628 |
| CUST_127019_PI430048170 | 3.91E-04 | 5.66954 | 3.082842 | 1.421378 |
| CUST_69903_PI430048170 | 2.58E-03 | 4.531888 | 0.520512 | 1.421301 |
| CUST_35876_PI430048170 | 1.08E-03 | 5.04669 | 1.69126 | 1.420017 |
| CUST_103011_PI430048170 | 1.58E-02 | 3.484613 | -1.85703 | 1.419549 |
| CUST_117211_PI430048170 | 1.08E-04 | 6.513549 | 4.901318 | 1.418831 |
| CUST_74238_PI430048170 | 3.63E-02 | 2.989236 | -2.93806 | 1.418125 |
| CUST_70917_PI430048170 | 1.58E-02 | 3.485407 | -1.85526 | 1.417347 |
| CUST_137807_PI430048170 | 1.98E-02 | 3.351304 | -2.15259 | 1.416392 |
| CUST_68177_PI430048170 | 7.41E-04 | 5.271704 | 2.197943 | 1.415853 |
| CUST_33539_PI430048170 | 9.16E-03 | 3.805636 | -1.13556 | 1.415522 |
| CUST_74705_PI430048170 | 9.05E-06 | 8.425533 | 8.661912 | 1.4151 |
| CUST_77879_PI430048170 | 2.63E-04 | 5.911421 | 3.61279 | 1.415084 |
| CUST_2278_PI430048170 | 3.07E-02 | 3.090366 | -2.72163 | 1.414446 |
| CUST_58784_PI430048170 | 4.13E-05 | 7.242095 | 6.395555 | 1.414018 |
| CUST_129499_PI430048170 | 1.59E-05 | 7.979046 | 7.830434 | 1.413974 |
| CUST_71077_PI430048170 | 3.95E-05 | 7.273082 | 6.457455 | 1.413605 |
| CUST_34382_PI430048170 | 2.67E-02 | 3.17352 | -2.54184 | 1.413434 |
| CUST_117954_PI430048170 | 2.26E-02 | 3.272649 | -2.32556 | 1.413066 |
| CUST_11159_PI430048170 | 3.38E-02 | 3.032476 | -2.84584 | 1.411349 |
| CUST_42338_PI430048170 | 4.44E-02 | 2.862562 | -3.20533 | 1.410078 |
| CUST_39334_PI430048170 | 1.26E-04 | 6.403244 | 4.668748 | 1.409807 |
| CUST_77311_PI430048170 | 3.72E-02 | 2.973179 | -2.97219 | 1.40919 |
| CUST_61521_PI430048170 | 1.43E-04 | 6.314527 | 4.480533 | 1.409097 |
| CUST_118016_PI430048170 | 4.75E-02 | 2.817817 | -3.29862 | 1.408419 |
| CUST_137857_PI430048170 | 1.11E-03 | 5.028733 | 1.650663 | 1.407377 |
| CUST_62376_PI430048170 | 2.43E-03 | 4.565987 | 0.598382 | 1.406827 |
| CUST_126983_PI430048170 | 4.73E-02 | 2.820794 | -3.29243 | 1.406465 |
| CUST_126480_PI430048170 | 1.41E-02 | 3.551127 | -1.70856 | 1.405617 |
| CUST_111449_PI430048170 | 9.50E-04 | 5.123592 | 1.864863 | 1.40507 |
| CUST_90586_PI430048170 | 5.89E-05 | 6.970339 | 5.846847 | 1.40497 |
| CUST_127805_PI430048170 | 6.72E-03 | 3.98138 | -0.73654 | 1.4019 |
| CUST_142676_PI430048170 | 4.51E-04 | 5.581808 | 2.889046 | 1.401622 |
| CUST_111081_PI430048170 | 2.56E-05 | 7.605285 | 7.112445 | 1.401087 |
| CUST_136561_PI430048170 | 1.47E-02 | 3.526845 | -1.76283 | 1.401049 |
| CUST_77482_PI430048170 | 6.17E-04 | 5.385811 | 2.45329 | 1.400918 |
| CUST_2822_PI430048170 | 3.95E-03 | 4.287125 | -0.03886 | 1.400027 |
| CUST_5049_PI430048170 | 1.58E-03 | 4.82282 | 1.183695 | 1.400014 |
| CUST_28558_PI430048170 | 7.90E-03 | 3.889223 | -0.94604 | 1.399948 |
| CUST_11758_PI430048170 | 3.28E-04 | 5.783294 | 3.332889 | 1.399413 |
| CUST_31026_PI430048170 | 6.97E-04 | 5.308085 | 2.279481 | 1.399137 |
| CUST_62253_PI430048170 | 4.15E-04 | 5.631603 | 2.999139 | 1.398565 |
| CUST_100516_PI430048170 | 1.20E-04 | 6.439925 | 4.746266 | 1.398106 |
| CUST_55162_PI430048170 | 3.91E-02 | 2.943268 | -3.03557 | 1.397487 |
| CUST_137859_PI430048170 | 1.70E-03 | 4.778935 | 1.083872 | 1.397473 |
| CUST_25698_PI430048170 | 8.02E-03 | 3.879929 | -0.96714 | 1.395768 |
| CUST_138507_PI430048170 | 3.53E-03 | 4.350812 | 0.106709 | 1.39576 |
| CUST_36407_PI430048170 | 3.55E-02 | 3.002848 | -2.90908 | 1.395372 |
| CUST_136868_PI430048170 | 2.81E-04 | 5.873085 | 3.529241 | 1.395268 |
| CUST_134159_PI430048170 | 9.03E-06 | 8.431424 | 8.672693 | 1.394704 |
| CUST_4586_PI430048170 | 1.03E-02 | 3.73322 | -1.29928 | 1.394553 |
| CUST_115117_PI430048170 | 4.32E-02 | 2.879632 | -3.16958 | 1.394188 |
| CUST_82250_PI430048170 | 1.46E-02 | 3.533489 | -1.74799 | 1.393037 |
| CUST_111718_PI430048170 | 4.95E-02 | 2.79284 | -3.35043 | 1.392174 |
| CUST_70386_PI430048170 | 4.42E-02 | 2.865837 | -3.19848 | 1.392009 |
| CUST_1220_PI430048170 | 1.09E-03 | 5.039858 | 1.675817 | 1.391947 |
| CUST_105155_PI430048170 | 3.26E-04 | 5.785759 | 3.338292 | 1.391672 |
| CUST_120927_PI430048170 | 3.50E-05 | 7.366706 | 6.64365 | 1.391388 |
| CUST_60714_PI430048170 | 2.61E-03 | 4.522886 | 0.49995 | 1.391151 |
| CUST_21249_PI430048170 | 5.35E-03 | 4.110947 | -0.44124 | 1.390258 |
| CUST_82828_PI430048170 | 9.83E-05 | 6.583435 | 5.047828 | 1.389606 |
| CUST_127017_PI430048170 | 1.10E-05 | 8.270546 | 8.376505 | 1.389245 |
| CUST_132089_PI430048170 | 3.22E-03 | 4.403382 | 0.226873 | 1.388613 |
| CUST_20787_PI430048170 | 2.52E-03 | 4.545486 | 0.551567 | 1.388313 |
| CUST_126699_PI430048170 | 2.01E-04 | 6.078427 | 3.97473 | 1.387883 |
| CUST_46373_PI430048170 | 2.52E-02 | 3.205314 | -2.47269 | 1.387219 |
| CUST_11990_PI430048170 | 3.52E-03 | 4.353272 | 0.112333 | 1.386631 |
| CUST_145422_PI430048170 | 1.36E-03 | 4.908855 | 1.379107 | 1.386225 |
| CUST_104064_PI430048170 | 1.09E-03 | 5.042845 | 1.682568 | 1.385974 |
| CUST_5205_PI430048170 | 8.85E-04 | 5.164692 | 1.957465 | 1.384938 |
| CUST_29300_PI430048170 | 8.02E-03 | 3.880701 | -0.96539 | 1.384513 |
| CUST_141274_PI430048170 | 1.34E-04 | 6.364627 | 4.586948 | 1.383849 |
| CUST_87619_PI430048170 | 1.64E-03 | 4.79789 | 1.127001 | 1.383418 |
| CUST_1760_PI430048170 | 2.44E-02 | 3.226196 | -2.42716 | 1.382642 |
| CUST_67016_PI430048170 | 5.79E-04 | 5.425412 | 2.541633 | 1.38186 |
| CUST_120940_PI430048170 | 1.96E-03 | 4.69521 | 0.893189 | 1.381684 |
| CUST_43449_PI430048170 | 1.33E-02 | 3.584372 | -1.63413 | 1.381666 |
| CUST_29918_PI430048170 | 5.46E-03 | 4.100038 | -0.46613 | 1.38161 |
| CUST_103149_PI430048170 | 4.78E-05 | 7.131806 | 6.174129 | 1.381075 |
| CUST_142432_PI430048170 | 2.72E-03 | 4.499877 | 0.447388 | 1.380178 |
| CUST_112946_PI430048170 | 4.22E-03 | 4.248686 | -0.1267 | 1.380075 |
| CUST_116573_PI430048170 | 5.55E-03 | 4.09046 | -0.48798 | 1.37997 |
| CUST_145525_PI430048170 | 2.23E-02 | 3.279766 | -2.30996 | 1.37996 |
| CUST_119321_PI430048170 | 1.15E-04 | 6.466375 | 4.802053 | 1.379804 |
| CUST_63596_PI430048170 | 9.79E-05 | 6.586482 | 5.054199 | 1.378931 |
| CUST_125865_PI430048170 | 2.00E-03 | 4.680421 | 0.859477 | 1.378528 |
| CUST_143094_PI430048170 | 1.14E-04 | 6.475432 | 4.821134 | 1.378197 |
| CUST_134818_PI430048170 | 8.99E-06 | 8.439116 | 8.686765 | 1.378188 |
| CUST_35722_PI430048170 | 8.76E-04 | 5.171092 | 1.971872 | 1.377937 |
| CUST_17012_PI430048170 | 2.60E-02 | 3.188649 | -2.50896 | 1.377637 |
| CUST_130483_PI430048170 | 4.92E-04 | 5.526407 | 2.766258 | 1.377011 |
| CUST_29608_PI430048170 | 4.38E-03 | 4.227455 | -0.17522 | 1.376377 |
| CUST_52116_PI430048170 | 8.53E-06 | 8.512321 | 8.820248 | 1.376155 |
| CUST_93745_PI430048170 | 1.98E-04 | 6.087069 | 3.993366 | 1.375881 |
| CUST_16554_PI430048170 | 4.54E-03 | 4.206391 | -0.22334 | 1.375425 |
| CUST_81483_PI430048170 | 7.42E-05 | 6.78967 | 5.476308 | 1.375291 |
| CUST_58052_PI430048170 | 1.62E-05 | 7.962074 | 7.798267 | 1.374845 |
| CUST_33416_PI430048170 | 1.85E-04 | 6.140011 | 4.107337 | 1.37437 |
| CUST_132154_PI430048170 | 2.94E-03 | 4.453446 | 0.341294 | 1.374204 |
| CUST_35406_PI430048170 | 3.57E-05 | 7.350333 | 6.611177 | 1.373954 |
| CUST_88354_PI430048170 | 1.10E-03 | 5.033437 | 1.6613 | 1.372448 |
| CUST_125558_PI430048170 | 3.54E-02 | 3.003835 | -2.90698 | 1.372336 |
| CUST_83872_PI430048170 | 5.41E-05 | 7.030373 | 5.968961 | 1.371981 |
| CUST_34184_PI430048170 | 1.59E-02 | 3.480412 | -1.86639 | 1.371828 |
| CUST_113570_PI430048170 | 4.47E-02 | 2.856715 | -3.21755 | 1.371597 |
| CUST_36666_PI430048170 | 8.83E-04 | 5.165902 | 1.960188 | 1.370349 |
| CUST_35954_PI430048170 | 2.54E-06 | 9.567089 | 10.66108 | 1.369544 |
| CUST_41150_PI430048170 | 7.54E-04 | 5.261888 | 2.175922 | 1.369451 |
| CUST_114042_PI430048170 | 3.63E-04 | 5.718231 | 3.190046 | 1.369341 |
| CUST_104251_PI430048170 | 1.22E-03 | 4.971311 | 1.520699 | 1.369207 |
| CUST_55850_PI430048170 | 1.77E-02 | 3.418018 | -2.00503 | 1.369176 |
| CUST_135369_PI430048170 | 8.83E-04 | 5.166339 | 1.961171 | 1.369074 |
| CUST_116337_PI430048170 | 1.29E-04 | 6.388849 | 4.63828 | 1.36832 |
| CUST_103867_PI430048170 | 3.88E-03 | 4.296414 | -0.01763 | 1.368303 |
| CUST_135649_PI430048170 | 6.21E-04 | 5.381436 | 2.44352 | 1.3672 |
| CUST_95557_PI430048170 | 4.23E-05 | 7.224235 | 6.359816 | 1.366667 |
| CUST_70322_PI430048170 | 2.74E-03 | 4.496326 | 0.439275 | 1.366238 |
| CUST_25986_PI430048170 | 2.26E-03 | 4.608717 | 0.695921 | 1.365952 |
| CUST_127170_PI430048170 | 2.12E-03 | 4.645315 | 0.779422 | 1.365949 |
| CUST_96635_PI430048170 | 3.10E-02 | 3.084353 | -2.73457 | 1.365904 |
| CUST_21696_PI430048170 | 1.05E-03 | 5.063798 | 1.729917 | 1.365764 |
| CUST_144566_PI430048170 | 3.64E-03 | 4.333367 | 0.066833 | 1.365644 |
| CUST_88912_PI430048170 | 7.92E-04 | 5.232803 | 2.110631 | 1.365465 |
| CUST_124912_PI430048170 | 1.58E-04 | 6.248233 | 4.339223 | 1.365076 |
| CUST_13184_PI430048170 | 1.27E-02 | 3.614149 | -1.56734 | 1.364646 |
| CUST_139129_PI430048170 | 2.27E-04 | 6.002858 | 3.81137 | 1.364018 |
| CUST_116085_PI430048170 | 2.08E-02 | 3.321189 | -2.21895 | 1.363275 |
| CUST_128079_PI430048170 | 3.89E-02 | 2.946495 | -3.02874 | 1.36316 |
| CUST_33414_PI430048170 | 1.10E-03 | 5.031894 | 1.657812 | 1.363062 |
| CUST_42661_PI430048170 | 2.82E-02 | 3.141499 | -2.61126 | 1.362281 |
| CUST_128292_PI430048170 | 2.26E-05 | 7.719493 | 7.333967 | 1.362259 |
| CUST_97834_PI430048170 | 2.75E-02 | 3.155982 | -2.57989 | 1.362246 |
| CUST_134235_PI430048170 | 8.09E-05 | 6.729124 | 5.35112 | 1.362012 |
| CUST_7841_PI430048170 | 9.95E-04 | 5.094948 | 1.800249 | 1.360899 |
| CUST_137786_PI430048170 | 8.14E-06 | 8.558945 | 8.904869 | 1.360586 |
| CUST_82961_PI430048170 | 1.54E-04 | 6.263249 | 4.371281 | 1.360454 |
| CUST_94329_PI430048170 | 4.51E-05 | 7.169959 | 6.250923 | 1.360176 |
| CUST_35652_PI430048170 | 1.32E-04 | 6.370499 | 4.5994 | 1.359678 |
| CUST_142659_PI430048170 | 4.41E-02 | 2.867361 | -3.19529 | 1.359636 |
| CUST_125398_PI430048170 | 1.20E-03 | 4.9813 | 1.543323 | 1.359504 |
| CUST_9516_PI430048170 | 7.78E-04 | 5.24358 | 2.134833 | 1.359126 |
| CUST_11794_PI430048170 | 6.71E-04 | 5.333589 | 2.336571 | 1.358813 |
| CUST_55607_PI430048170 | 2.18E-03 | 4.628579 | 0.741241 | 1.358549 |
| CUST_53042_PI430048170 | 1.40E-02 | 3.556022 | -1.69761 | 1.358419 |
| CUST_136482_PI430048170 | 9.67E-03 | 3.772494 | -1.21054 | 1.358337 |
| CUST_46408_PI430048170 | 1.51E-04 | 6.280038 | 4.407089 | 1.35772 |
| CUST_10490_PI430048170 | 1.52E-03 | 4.843775 | 1.231327 | 1.357684 |
| CUST_92465_PI430048170 | 1.90E-03 | 4.712655 | 0.932942 | 1.357341 |
| CUST_142970_PI430048170 | 2.33E-02 | 3.254637 | -2.36501 | 1.356953 |
| CUST_127186_PI430048170 | 2.35E-02 | 3.249877 | -2.37542 | 1.356898 |
| CUST_65537_PI430048170 | 6.23E-05 | 6.920799 | 5.745698 | 1.35686 |
| CUST_33574_PI430048170 | 1.52E-04 | 6.274325 | 4.394908 | 1.35644 |
| CUST_96949_PI430048170 | 6.80E-04 | 5.324755 | 2.316804 | 1.355508 |
| CUST_67251_PI430048170 | 5.88E-05 | 6.972871 | 5.852008 | 1.354131 |
| CUST_135807_PI430048170 | 1.04E-06 | 10.33145 | 11.90248 | 1.353976 |
| CUST_64678_PI430048170 | 1.27E-04 | 6.400228 | 4.662366 | 1.353533 |
| CUST_131566_PI430048170 | 1.19E-05 | 8.206978 | 8.258455 | 1.353411 |
| CUST_126972_PI430048170 | 6.94E-04 | 5.311324 | 2.286733 | 1.35337 |
| CUST_133345_PI430048170 | 4.46E-02 | 2.859026 | -3.21272 | 1.352797 |
| CUST_140969_PI430048170 | 2.52E-02 | 3.207438 | -2.46806 | 1.352601 |
| CUST_134648_PI430048170 | 1.70E-02 | 3.443116 | -1.94933 | 1.352549 |
| CUST_136043_PI430048170 | 2.23E-06 | 9.688493 | 10.86331 | 1.352439 |
| CUST_91139_PI430048170 | 2.99E-03 | 4.44467 | 0.321238 | 1.351666 |
| CUST_138598_PI430048170 | 4.57E-02 | 2.842469 | -3.2473 | 1.351429 |
| CUST_67423_PI430048170 | 2.35E-06 | 9.642403 | 10.78676 | 1.351326 |
| CUST_58708_PI430048170 | 9.22E-03 | 3.801491 | -1.14494 | 1.351151 |
| CUST_126307_PI430048170 | 2.96E-02 | 3.113309 | -2.67218 | 1.350765 |
| CUST_120914_PI430048170 | 1.26E-02 | 3.61849 | -1.5576 | 1.349708 |
| CUST_106477_PI430048170 | 8.81E-04 | 5.167464 | 1.963705 | 1.349395 |
| CUST_63681_PI430048170 | 6.86E-03 | 3.969215 | -0.76422 | 1.349315 |
| CUST_128041_PI430048170 | 4.90E-03 | 4.162382 | -0.32384 | 1.348351 |
| CUST_30744_PI430048170 | 5.63E-03 | 4.082535 | -0.50606 | 1.347968 |
| CUST_112667_PI430048170 | 6.75E-03 | 3.978708 | -0.74262 | 1.34792 |
| CUST_103018_PI430048170 | 1.16E-03 | 5.004509 | 1.595862 | 1.347838 |
| CUST_35706_PI430048170 | 1.07E-04 | 6.520598 | 4.916126 | 1.346801 |
| CUST_41302_PI430048170 | 1.86E-03 | 4.722402 | 0.95515 | 1.346776 |
| CUST_135465_PI430048170 | 3.71E-05 | 7.317907 | 6.546757 | 1.346707 |
| CUST_141578_PI430048170 | 3.63E-04 | 5.717338 | 3.188082 | 1.346419 |
| CUST_1326_PI430048170 | 1.67E-02 | 3.454007 | -1.92513 | 1.346208 |
| CUST_7901_PI430048170 | 6.23E-07 | 10.84878 | 12.70107 | 1.346192 |
| CUST_46823_PI430048170 | 1.42E-04 | 6.320099 | 4.492385 | 1.346091 |
| CUST_120507_PI430048170 | 1.36E-03 | 4.909221 | 1.379939 | 1.345875 |
| CUST_2814_PI430048170 | 3.03E-02 | 3.098105 | -2.70496 | 1.345466 |
| CUST_24799_PI430048170 | 1.75E-02 | 3.425691 | -1.98802 | 1.345282 |
| CUST_36415_PI430048170 | 1.05E-04 | 6.533175 | 4.942529 | 1.345007 |
| CUST_80503_PI430048170 | 4.16E-05 | 7.238474 | 6.388312 | 1.344625 |
| CUST_96865_PI430048170 | 7.78E-04 | 5.243888 | 2.135524 | 1.344579 |
| CUST_73023_PI430048170 | 3.77E-05 | 7.303043 | 6.517176 | 1.344435 |
| CUST_35976_PI430048170 | 2.65E-03 | 4.513918 | 0.479465 | 1.344263 |
| CUST_71089_PI430048170 | 5.26E-06 | 8.921192 | 9.551971 | 1.34361 |
| CUST_99415_PI430048170 | 1.50E-04 | 6.283512 | 4.414493 | 1.342545 |
| CUST_36214_PI430048170 | 6.04E-04 | 5.397164 | 2.478632 | 1.342483 |
| CUST_50994_PI430048170 | 8.90E-04 | 5.16072 | 1.94852 | 1.340895 |
| CUST_25994_PI430048170 | 2.34E-02 | 3.250748 | -2.37352 | 1.34075 |
| CUST_142725_PI430048170 | 1.72E-05 | 7.921547 | 7.721285 | 1.340604 |
| CUST_23537_PI430048170 | 3.40E-03 | 4.372344 | 0.155926 | 1.340186 |
| CUST_34394_PI430048170 | 2.94E-02 | 3.117095 | -2.66401 | 1.339217 |
| CUST_13596_PI430048170 | 4.62E-02 | 2.836666 | -3.25939 | 1.338918 |
| CUST_69479_PI430048170 | 5.25E-03 | 4.121668 | -0.41678 | 1.338173 |
| CUST_35734_PI430048170 | 3.59E-04 | 5.727173 | 3.209705 | 1.338056 |
| CUST_41464_PI430048170 | 1.22E-03 | 4.973836 | 1.526418 | 1.337903 |
| CUST_137896_PI430048170 | 1.96E-02 | 3.356459 | -2.14122 | 1.33779 |
| CUST_87757_PI430048170 | 1.00E-02 | 3.752088 | -1.25667 | 1.337046 |
| CUST_135357_PI430048170 | 5.05E-04 | 5.508613 | 2.726754 | 1.33684 |
| CUST_14502_PI430048170 | 2.02E-03 | 4.675923 | 0.849223 | 1.336585 |
| CUST_76575_PI430048170 | 3.79E-03 | 4.310195 | 0.013868 | 1.336504 |
| CUST_108144_PI430048170 | 1.20E-04 | 6.441055 | 4.748652 | 1.336232 |
| CUST_122911_PI430048170 | 4.91E-03 | 4.16122 | -0.3265 | 1.33552 |
| CUST_126142_PI430048170 | 9.51E-05 | 6.608453 | 5.100114 | 1.335464 |
| CUST_51060_PI430048170 | 4.43E-02 | 2.863204 | -3.20398 | 1.335338 |
| CUST_99694_PI430048170 | 1.57E-03 | 4.823977 | 1.186326 | 1.335067 |
| CUST_33270_PI430048170 | 1.04E-05 | 8.323535 | 8.474468 | 1.334953 |
| CUST_119536_PI430048170 | 6.10E-05 | 6.945599 | 5.796376 | 1.334218 |
| CUST_34377_PI430048170 | 3.52E-02 | 3.006561 | -2.90117 | 1.333384 |
| CUST_90686_PI430048170 | 5.44E-03 | 4.101264 | -0.46334 | 1.333327 |
| CUST_88605_PI430048170 | 1.65E-02 | 3.459021 | -1.91399 | 1.333069 |
| CUST_26894_PI430048170 | 2.67E-02 | 3.173443 | -2.542 | 1.332364 |
| CUST_7470_PI430048170 | 1.77E-03 | 4.754308 | 1.027816 | 1.332168 |
| CUST_78500_PI430048170 | 2.10E-05 | 7.772781 | 7.436684 | 1.331818 |
| CUST_39121_PI430048170 | 4.40E-02 | 2.8689 | -3.19206 | 1.331816 |
| CUST_114140_PI430048170 | 4.48E-04 | 5.586449 | 2.899317 | 1.331419 |
| CUST_110805_PI430048170 | 2.93E-05 | 7.510196 | 6.92658 | 1.331038 |
| CUST_60561_PI430048170 | 1.39E-03 | 4.895824 | 1.349535 | 1.330983 |
| CUST_144912_PI430048170 | 1.37E-04 | 6.347819 | 4.551283 | 1.330785 |
| CUST_12861_PI430048170 | 2.26E-03 | 4.609481 | 0.697663 | 1.329785 |
| CUST_32962_PI430048170 | 1.31E-02 | 3.595449 | -1.60929 | 1.329405 |
| CUST_73473_PI430048170 | 5.76E-06 | 8.840814 | 9.409964 | 1.329346 |
| CUST_29760_PI430048170 | 3.99E-02 | 2.930983 | -3.06153 | 1.329282 |
| CUST_79120_PI430048170 | 4.57E-05 | 7.160517 | 6.231939 | 1.32906 |
| CUST_101825_PI430048170 | 2.55E-03 | 4.538841 | 0.536392 | 1.328738 |
| CUST_94035_PI430048170 | 1.48E-02 | 3.522581 | -1.77235 | 1.328585 |
| CUST_42716_PI430048170 | 3.63E-04 | 5.717742 | 3.188969 | 1.328447 |
| CUST_105047_PI430048170 | 8.61E-04 | 5.181911 | 1.996221 | 1.328311 |
| CUST_107503_PI430048170 | 4.78E-05 | 7.130784 | 6.17207 | 1.327548 |
| CUST_81043_PI430048170 | 4.58E-02 | 2.841424 | -3.24948 | 1.327443 |
| CUST_35399_PI430048170 | 1.32E-03 | 4.924451 | 1.414486 | 1.326479 |
| CUST_118600_PI430048170 | 2.98E-02 | 3.107916 | -2.68381 | 1.32453 |
| CUST_23946_PI430048170 | 3.61E-05 | 7.341237 | 6.593122 | 1.324444 |
| CUST_144047_PI430048170 | 3.11E-05 | 7.466604 | 6.840937 | 1.324269 |
| CUST_131418_PI430048170 | 1.87E-02 | 3.38502 | -2.07811 | 1.323995 |
| CUST_95282_PI430048170 | 1.30E-05 | 8.136473 | 8.126852 | 1.323678 |
| CUST_39331_PI430048170 | 4.38E-03 | 4.226789 | -0.17674 | 1.323638 |
| CUST_26405_PI430048170 | 8.33E-03 | 3.858332 | -1.01614 | 1.323363 |
| CUST_144744_PI430048170 | 2.64E-04 | 5.910335 | 3.610426 | 1.322624 |
| CUST_49074_PI430048170 | 7.69E-03 | 3.904109 | -0.91224 | 1.322388 |
| CUST_99820_PI430048170 | 2.28E-03 | 4.602639 | 0.682051 | 1.32213 |
| CUST_130195_PI430048170 | 3.01E-02 | 3.102944 | -2.69453 | 1.322001 |
| CUST_34879_PI430048170 | 2.23E-03 | 4.616909 | 0.714615 | 1.321421 |
| CUST_34929_PI430048170 | 1.73E-02 | 3.431947 | -1.97413 | 1.320879 |
| CUST_109596_PI430048170 | 9.75E-05 | 6.589489 | 5.060486 | 1.320589 |
| CUST_102167_PI430048170 | 1.24E-04 | 6.420597 | 4.705443 | 1.320466 |
| CUST_85100_PI430048170 | 2.55E-04 | 5.933567 | 3.660978 | 1.320351 |
| CUST_7271_PI430048170 | 8.54E-05 | 6.691691 | 5.27347 | 1.319623 |
| CUST_137939_PI430048170 | 6.36E-03 | 4.01474 | -0.66058 | 1.318377 |
| CUST_25659_PI430048170 | 1.89E-03 | 4.713921 | 0.935828 | 1.318346 |
| CUST_141700_PI430048170 | 2.29E-02 | 3.263645 | -2.34529 | 1.317978 |
| CUST_89313_PI430048170 | 2.26E-02 | 3.272862 | -2.32509 | 1.317062 |
| CUST_87406_PI430048170 | 3.33E-05 | 7.415416 | 6.740025 | 1.316589 |
| CUST_103782_PI430048170 | 1.09E-02 | 3.7021 | -1.36948 | 1.315972 |
| CUST_113973_PI430048170 | 2.35E-06 | 9.63329 | 10.77159 | 1.315323 |
| CUST_76756_PI430048170 | 3.93E-03 | 4.289911 | -0.03249 | 1.315221 |
| CUST_75688_PI430048170 | 1.97E-03 | 4.691313 | 0.884304 | 1.314701 |
| CUST_49936_PI430048170 | 2.02E-03 | 4.673699 | 0.844151 | 1.314559 |
| CUST_24651_PI430048170 | 2.58E-03 | 4.531316 | 0.519204 | 1.313938 |
| CUST_132583_PI430048170 | 1.70E-02 | 3.444333 | -1.94663 | 1.313872 |
| CUST_140697_PI430048170 | 1.92E-04 | 6.111195 | 4.045347 | 1.313655 |
| CUST_23166_PI430048170 | 7.94E-03 | 3.886834 | -0.95147 | 1.313602 |
| CUST_77269_PI430048170 | 6.76E-03 | 3.977813 | -0.74466 | 1.3133 |
| CUST_71300_PI430048170 | 1.29E-05 | 8.1465 | 8.145611 | 1.313245 |
| CUST_127487_PI430048170 | 6.25E-05 | 6.918569 | 5.741137 | 1.312946 |
| CUST_139437_PI430048170 | 1.86E-03 | 4.724214 | 0.959277 | 1.312699 |
| CUST_82154_PI430048170 | 1.51E-02 | 3.511959 | -1.79606 | 1.31254 |
| CUST_56035_PI430048170 | 1.57E-03 | 4.827097 | 1.193418 | 1.312507 |
| CUST_20874_PI430048170 | 1.00E-02 | 3.750925 | -1.25929 | 1.312456 |
| CUST_68798_PI430048170 | 2.84E-04 | 5.866443 | 3.514748 | 1.312438 |
| CUST_114098_PI430048170 | 7.80E-03 | 3.896349 | -0.92986 | 1.312297 |
| CUST_131214_PI430048170 | 5.33E-04 | 5.477029 | 2.656558 | 1.312065 |
| CUST_133174_PI430048170 | 4.83E-02 | 2.80788 | -3.31926 | 1.310994 |
| CUST_119371_PI430048170 | 7.63E-05 | 6.770676 | 5.43709 | 1.310042 |
| CUST_92407_PI430048170 | 6.37E-05 | 6.901815 | 5.706846 | 1.309627 |
| CUST_16069_PI430048170 | 2.75E-02 | 3.155262 | -2.58145 | 1.309386 |
| CUST_29943_PI430048170 | 1.44E-02 | 3.540148 | -1.73311 | 1.309022 |
| CUST_74704_PI430048170 | 7.60E-04 | 5.257075 | 2.165124 | 1.308698 |
| CUST_137312_PI430048170 | 2.81E-03 | 4.481257 | 0.404846 | 1.308666 |
| CUST_95667_PI430048170 | 1.93E-02 | 3.367117 | -2.11769 | 1.308626 |
| CUST_75152_PI430048170 | 2.65E-02 | 3.177456 | -2.53329 | 1.308616 |
| CUST_99355_PI430048170 | 6.67E-04 | 5.337826 | 2.346051 | 1.307811 |
| CUST_88353_PI430048170 | 6.57E-03 | 3.994538 | -0.70659 | 1.307476 |
| CUST_42873_PI430048170 | 3.24E-03 | 4.399815 | 0.218719 | 1.307031 |
| CUST_16913_PI430048170 | 8.47E-03 | 3.848889 | -1.03756 | 1.306881 |
| CUST_113643_PI430048170 | 3.16E-03 | 4.412885 | 0.248593 | 1.306653 |
| CUST_101084_PI430048170 | 2.00E-03 | 4.682133 | 0.863378 | 1.306009 |
| CUST_130505_PI430048170 | 1.01E-02 | 3.747007 | -1.26814 | 1.306001 |
| CUST_145230_PI430048170 | 1.29E-02 | 3.60342 | -1.59142 | 1.305669 |
| CUST_38178_PI430048170 | 5.22E-05 | 7.058226 | 6.025444 | 1.305648 |
| CUST_12476_PI430048170 | 1.24E-03 | 4.961038 | 1.497425 | 1.305533 |
| CUST_35969_PI430048170 | 7.90E-05 | 6.744191 | 5.38232 | 1.305355 |
| CUST_118819_PI430048170 | 6.42E-03 | 4.008487 | -0.67482 | 1.305342 |
| CUST_137448_PI430048170 | 4.97E-04 | 5.520599 | 2.753366 | 1.304777 |
| CUST_145494_PI430048170 | 1.19E-02 | 3.653227 | -1.47954 | 1.304617 |
| CUST_35684_PI430048170 | 1.38E-04 | 6.340016 | 4.534715 | 1.304052 |
| CUST_82212_PI430048170 | 2.82E-03 | 4.478478 | 0.398496 | 1.303989 |
| CUST_54400_PI430048170 | 1.17E-02 | 3.66282 | -1.45795 | 1.303867 |
| CUST_89214_PI430048170 | 1.14E-02 | 3.678865 | -1.42183 | 1.303816 |
| CUST_24173_PI430048170 | 2.58E-02 | 3.192142 | -2.50136 | 1.303802 |
| CUST_26046_PI430048170 | 2.86E-02 | 3.133041 | -2.62956 | 1.303645 |
| CUST_108275_PI430048170 | 5.49E-03 | 4.096919 | -0.47325 | 1.303642 |
| CUST_70909_PI430048170 | 1.22E-03 | 4.972192 | 1.522694 | 1.303058 |
| CUST_66401_PI430048170 | 1.48E-03 | 4.858737 | 1.265323 | 1.303006 |
| CUST_119088_PI430048170 | 2.47E-05 | 7.6422 | 7.184251 | 1.302729 |
| CUST_83027_PI430048170 | 9.19E-05 | 6.635707 | 5.156979 | 1.302654 |
| CUST_4968_PI430048170 | 1.46E-04 | 6.299743 | 4.44907 | 1.301662 |
| CUST_94460_PI430048170 | 1.54E-03 | 4.835815 | 1.213237 | 1.301557 |
| CUST_34793_PI430048170 | 2.55E-03 | 4.53799 | 0.534448 | 1.301408 |
| CUST_25759_PI430048170 | 8.94E-03 | 3.819409 | -1.10437 | 1.301303 |
| CUST_20480_PI430048170 | 3.96E-04 | 5.659263 | 3.060182 | 1.301242 |
| CUST_110782_PI430048170 | 5.21E-04 | 5.491166 | 2.687991 | 1.301215 |
| CUST_61935_PI430048170 | 3.91E-02 | 2.943442 | -3.0352 | 1.300753 |
| CUST_106527_PI430048170 | 1.07E-04 | 6.524066 | 4.923408 | 1.300499 |
| CUST_13430_PI430048170 | 1.02E-02 | 3.742516 | -1.27829 | 1.299645 |
| CUST_54796_PI430048170 | 1.70E-04 | 6.200425 | 4.23697 | 1.299065 |
| CUST_101454_PI430048170 | 1.64E-03 | 4.79778 | 1.126748 | 1.297914 |
| CUST_60913_PI430048170 | 1.73E-04 | 6.189799 | 4.214201 | 1.297844 |
| CUST_26897_PI430048170 | 2.02E-02 | 3.3379 | -2.18215 | 1.297359 |
| CUST_130480_PI430048170 | 2.31E-02 | 3.259862 | -2.35357 | 1.296374 |
| CUST_129450_PI430048170 | 6.48E-03 | 4.002717 | -0.68796 | 1.296141 |
| CUST_9489_PI430048170 | 3.94E-04 | 5.663035 | 3.068501 | 1.294938 |
| CUST_73087_PI430048170 | 1.74E-02 | 3.430222 | -1.97796 | 1.293992 |
| CUST_5000_PI430048170 | 5.59E-05 | 7.008812 | 5.925163 | 1.293964 |
| CUST_128076_PI430048170 | 1.14E-04 | 6.47657 | 4.823532 | 1.293524 |
| CUST_121530_PI430048170 | 8.27E-04 | 5.205566 | 2.049427 | 1.293348 |
| CUST_117300_PI430048170 | 6.23E-03 | 4.026038 | -0.63484 | 1.29272 |
| CUST_52573_PI430048170 | 9.80E-03 | 3.765479 | -1.2264 | 1.292553 |
| CUST_67825_PI430048170 | 1.86E-03 | 4.726726 | 0.965 | 1.292101 |
| CUST_114644_PI430048170 | 3.97E-02 | 2.933768 | -3.05565 | 1.291566 |
| CUST_119760_PI430048170 | 8.36E-04 | 5.199198 | 2.035108 | 1.290832 |
| CUST_6922_PI430048170 | 6.38E-07 | 10.81844 | 12.65513 | 1.290627 |
| CUST_69435_PI430048170 | 9.32E-04 | 5.134233 | 1.888849 | 1.290346 |
| CUST_136601_PI430048170 | 2.44E-04 | 5.960004 | 3.718425 | 1.289469 |
| CUST_39361_PI430048170 | 2.46E-04 | 5.954753 | 3.70702 | 1.289167 |
| CUST_117394_PI430048170 | 1.14E-03 | 5.012957 | 1.61498 | 1.288963 |
| CUST_90460_PI430048170 | 1.45E-03 | 4.870208 | 1.291377 | 1.288704 |
| CUST_132756_PI430048170 | 2.24E-05 | 7.728134 | 7.350651 | 1.288432 |
| CUST_89709_PI430048170 | 7.56E-03 | 3.913876 | -0.89005 | 1.287714 |
| CUST_33417_PI430048170 | 4.75E-04 | 5.55101 | 2.820824 | 1.287019 |
| CUST_74755_PI430048170 | 1.81E-02 | 3.405415 | -2.03297 | 1.286984 |
| CUST_70878_PI430048170 | 1.85E-02 | 3.392279 | -2.06205 | 1.286282 |
| CUST_96739_PI430048170 | 7.17E-04 | 5.290569 | 2.240238 | 1.285293 |
| CUST_29015_PI430048170 | 7.55E-03 | 3.915086 | -0.8873 | 1.284727 |
| CUST_143902_PI430048170 | 1.99E-05 | 7.822488 | 7.532131 | 1.284698 |
| CUST_76194_PI430048170 | 5.11E-03 | 4.137294 | -0.38112 | 1.284662 |
| CUST_113123_PI430048170 | 2.48E-02 | 3.216349 | -2.44864 | 1.283862 |
| CUST_56076_PI430048170 | 5.20E-04 | 5.491618 | 2.688994 | 1.283795 |
| CUST_74531_PI430048170 | 1.08E-02 | 3.710202 | -1.35121 | 1.283604 |
| CUST_64359_PI430048170 | 3.49E-03 | 4.357511 | 0.122022 | 1.283468 |
| CUST_134812_PI430048170 | 1.95E-03 | 4.69607 | 0.895147 | 1.283301 |
| CUST_133992_PI430048170 | 4.90E-04 | 5.529572 | 2.77328 | 1.282295 |
| CUST_122035_PI430048170 | 1.85E-03 | 4.728496 | 0.969033 | 1.281336 |
| CUST_142859_PI430048170 | 1.38E-02 | 3.563621 | -1.6806 | 1.281311 |
| CUST_58824_PI430048170 | 2.38E-04 | 5.973704 | 3.748163 | 1.281034 |
| CUST_129256_PI430048170 | 2.51E-05 | 7.621706 | 7.144411 | 1.280944 |
| CUST_18740_PI430048170 | 4.04E-03 | 4.273336 | -0.07037 | 1.27973 |
| CUST_133638_PI430048170 | 6.31E-03 | 4.019356 | -0.65007 | 1.279615 |
| CUST_72569_PI430048170 | 1.29E-04 | 6.388088 | 4.636668 | 1.279515 |
| CUST_92238_PI430048170 | 7.85E-05 | 6.748485 | 5.391207 | 1.279061 |
| CUST_78390_PI430048170 | 1.06E-03 | 5.057368 | 1.715389 | 1.279004 |
| CUST_58787_PI430048170 | 2.00E-03 | 4.681297 | 0.861473 | 1.278698 |
| CUST_90737_PI430048170 | 2.26E-03 | 4.607031 | 0.692074 | 1.27835 |
| CUST_82324_PI430048170 | 1.05E-03 | 5.063259 | 1.728699 | 1.277973 |
| CUST_134605_PI430048170 | 1.83E-02 | 3.399574 | -2.04591 | 1.277967 |
| CUST_138660_PI430048170 | 7.23E-03 | 3.93861 | -0.83383 | 1.277642 |
| CUST_136204_PI430048170 | 1.94E-04 | 6.101284 | 4.024001 | 1.27756 |
| CUST_106885_PI430048170 | 1.25E-05 | 8.168513 | 8.186745 | 1.277423 |
| CUST_128581_PI430048170 | 1.51E-04 | 6.277799 | 4.402315 | 1.276711 |
| CUST_71313_PI430048170 | 2.34E-02 | 3.250657 | -2.37371 | 1.276255 |
| CUST_106276_PI430048170 | 1.13E-04 | 6.481942 | 4.834842 | 1.276032 |
| CUST_128007_PI430048170 | 7.27E-07 | 10.66273 | 12.41762 | 1.275693 |
| CUST_13431_PI430048170 | 1.10E-02 | 3.699576 | -1.37517 | 1.275248 |
| CUST_98249_PI430048170 | 9.80E-03 | 3.765297 | -1.22681 | 1.275063 |
| CUST_21200_PI430048170 | 1.77E-02 | 3.419443 | -2.00188 | 1.27467 |
| CUST_93122_PI430048170 | 4.56E-02 | 2.844483 | -3.2431 | 1.273373 |
| CUST_140673_PI430048170 | 2.71E-05 | 7.560116 | 7.024318 | 1.27241 |
| CUST_42007_PI430048170 | 2.46E-02 | 3.22131 | -2.43782 | 1.272109 |
| CUST_140666_PI430048170 | 3.31E-03 | 4.387361 | 0.190253 | 1.272007 |
| CUST_107137_PI430048170 | 6.23E-04 | 5.378842 | 2.437727 | 1.271907 |
| CUST_2594_PI430048170 | 1.17E-03 | 4.995313 | 1.57505 | 1.271899 |
| CUST_73025_PI430048170 | 7.16E-05 | 6.813544 | 5.525533 | 1.271263 |
| CUST_35579_PI430048170 | 2.17E-05 | 7.752096 | 7.39686 | 1.270986 |
| CUST_106096_PI430048170 | 2.46E-05 | 7.647455 | 7.194458 | 1.270791 |
| CUST_4967_PI430048170 | 1.38E-04 | 6.337927 | 4.530278 | 1.270171 |
| CUST_136395_PI430048170 | 4.09E-03 | 4.266664 | -0.08562 | 1.269735 |
| CUST_13493_PI430048170 | 1.62E-03 | 4.807736 | 1.149395 | 1.269735 |
| CUST_60869_PI430048170 | 2.70E-03 | 4.50308 | 0.454706 | 1.268597 |
| CUST_35423_PI430048170 | 9.97E-05 | 6.572176 | 5.024267 | 1.267831 |
| CUST_119847_PI430048170 | 4.12E-04 | 5.635068 | 3.006791 | 1.26753 |
| CUST_137131_PI430048170 | 7.01E-05 | 6.831346 | 5.562186 | 1.267444 |
| CUST_142296_PI430048170 | 2.20E-02 | 3.289472 | -2.28866 | 1.266947 |
| CUST_29495_PI430048170 | 9.98E-04 | 5.093056 | 1.795979 | 1.266261 |
| CUST_29449_PI430048170 | 1.01E-03 | 5.085557 | 1.779053 | 1.264775 |
| CUST_76965_PI430048170 | 1.32E-06 | 10.11802 | 11.56338 | 1.264136 |
| CUST_134888_PI430048170 | 1.55E-02 | 3.497083 | -1.82924 | 1.26344 |
| CUST_120967_PI430048170 | 3.39E-02 | 3.030107 | -2.8509 | 1.263365 |
| CUST_70818_PI430048170 | 1.24E-04 | 6.42094 | 4.706167 | 1.263331 |
| CUST_129370_PI430048170 | 2.47E-02 | 3.218492 | -2.44397 | 1.263174 |
| CUST_135967_PI430048170 | 1.84E-02 | 3.393641 | -2.05904 | 1.262986 |
| CUST_7860_PI430048170 | 1.31E-03 | 4.932557 | 1.43287 | 1.262914 |
| CUST_104826_PI430048170 | 2.15E-02 | 3.301788 | -2.26162 | 1.262738 |
| CUST_110194_PI430048170 | 1.17E-03 | 4.999508 | 1.584545 | 1.262737 |
| CUST_35709_PI430048170 | 8.07E-05 | 6.730764 | 5.354517 | 1.261542 |
| CUST_60330_PI430048170 | 1.12E-04 | 6.486828 | 4.845126 | 1.260966 |
| CUST_90650_PI430048170 | 1.27E-03 | 4.948276 | 1.468506 | 1.26019 |
| CUST_91094_PI430048170 | 9.44E-03 | 3.787663 | -1.17624 | 1.260026 |
| CUST_132036_PI430048170 | 1.12E-03 | 5.023469 | 1.638757 | 1.259681 |
| CUST_79854_PI430048170 | 1.57E-03 | 4.8256 | 1.190017 | 1.259145 |
| CUST_76680_PI430048170 | 6.23E-05 | 6.925728 | 5.755777 | 1.259002 |
| CUST_37686_PI430048170 | 7.51E-05 | 6.78183 | 5.460127 | 1.258871 |
| CUST_113483_PI430048170 | 1.52E-03 | 4.844923 | 1.233937 | 1.258717 |
| CUST_120037_PI430048170 | 1.68E-04 | 6.208 | 4.25319 | 1.257781 |
| CUST_21_PI430048170 | 6.93E-04 | 5.313344 | 2.291257 | 1.257602 |
| CUST_56810_PI430048170 | 2.86E-02 | 3.131246 | -2.63344 | 1.25754 |
| CUST_101891_PI430048170 | 1.68E-02 | 3.448462 | -1.93746 | 1.256771 |
| CUST_62603_PI430048170 | 8.97E-05 | 6.656332 | 5.199944 | 1.256727 |
| CUST_95969_PI430048170 | 3.64E-04 | 5.715914 | 3.18495 | 1.256558 |
| CUST_34781_PI430048170 | 6.81E-05 | 6.851579 | 5.603792 | 1.256491 |
| CUST_109536_PI430048170 | 3.89E-06 | 9.167148 | 9.980987 | 1.255952 |
| CUST_119377_PI430048170 | 5.63E-05 | 7.000911 | 5.909096 | 1.255751 |
| CUST_100357_PI430048170 | 9.58E-03 | 3.778826 | -1.19623 | 1.25531 |
| CUST_71748_PI430048170 | 3.30E-04 | 5.778177 | 3.321673 | 1.255209 |
| CUST_74367_PI430048170 | 9.96E-03 | 3.755493 | -1.24897 | 1.254693 |
| CUST_4997_PI430048170 | 8.89E-05 | 6.663238 | 5.214318 | 1.254651 |
| CUST_94297_PI430048170 | 1.29E-02 | 3.603122 | -1.59209 | 1.254413 |
| CUST_54035_PI430048170 | 1.35E-03 | 4.913439 | 1.389508 | 1.254404 |
| CUST_115666_PI430048170 | 3.26E-06 | 9.34246 | 10.28175 | 1.254163 |
| CUST_40238_PI430048170 | 9.31E-03 | 3.79533 | -1.15889 | 1.254108 |
| CUST_128918_PI430048170 | 1.10E-04 | 6.50604 | 4.885538 | 1.253362 |
| CUST_109412_PI430048170 | 3.17E-04 | 5.802871 | 3.375777 | 1.253275 |
| CUST_55726_PI430048170 | 9.68E-04 | 5.112076 | 1.838894 | 1.252781 |
| CUST_55957_PI430048170 | 2.55E-03 | 4.539298 | 0.537435 | 1.2525 |
| CUST_84294_PI430048170 | 1.13E-04 | 6.483728 | 4.838602 | 1.252053 |
| CUST_49734_PI430048170 | 1.43E-02 | 3.544394 | -1.72361 | 1.251871 |
| CUST_103959_PI430048170 | 4.46E-02 | 2.859301 | -3.21215 | 1.251708 |
| CUST_59901_PI430048170 | 1.05E-03 | 5.063572 | 1.729407 | 1.250425 |
| CUST_137254_PI430048170 | 3.23E-02 | 3.060566 | -2.78567 | 1.249886 |
| CUST_63524_PI430048170 | 2.21E-03 | 4.620456 | 0.72271 | 1.249833 |
| CUST_139855_PI430048170 | 6.19E-06 | 8.787715 | 9.315661 | 1.249618 |
| CUST_79496_PI430048170 | 3.37E-02 | 3.033713 | -2.84319 | 1.249353 |
| CUST_113445_PI430048170 | 1.33E-04 | 6.365867 | 4.589578 | 1.249097 |
| CUST_92294_PI430048170 | 3.96E-04 | 5.65917 | 3.059977 | 1.249063 |
| CUST_33361_PI430048170 | 1.86E-02 | 3.387733 | -2.07211 | 1.248999 |
| CUST_103904_PI430048170 | 1.97E-02 | 3.353586 | -2.14756 | 1.248831 |
| CUST_131310_PI430048170 | 2.61E-02 | 3.184704 | -2.51754 | 1.248709 |
| CUST_91319_PI430048170 | 2.50E-04 | 5.944841 | 3.685485 | 1.248574 |
| CUST_103320_PI430048170 | 1.11E-02 | 3.69359 | -1.38866 | 1.248371 |
| CUST_143787_PI430048170 | 2.39E-05 | 7.673861 | 7.245681 | 1.248236 |
| CUST_139971_PI430048170 | 2.29E-04 | 5.998381 | 3.80167 | 1.247555 |
| CUST_98816_PI430048170 | 3.23E-02 | 3.059489 | -2.78798 | 1.246955 |
| CUST_10639_PI430048170 | 1.23E-04 | 6.42316 | 4.710859 | 1.246371 |
| CUST_89822_PI430048170 | 8.60E-05 | 6.687389 | 5.264532 | 1.245985 |
| CUST_114748_PI430048170 | 5.76E-06 | 8.843985 | 9.415584 | 1.245451 |
| CUST_95487_PI430048170 | 1.55E-02 | 3.495337 | -1.83313 | 1.245387 |
| CUST_137334_PI430048170 | 2.85E-04 | 5.864436 | 3.510368 | 1.245048 |
| CUST_110228_PI430048170 | 9.97E-05 | 6.572266 | 5.024457 | 1.244799 |
| CUST_26654_PI430048170 | 3.08E-02 | 3.088873 | -2.72484 | 1.244699 |
| CUST_125519_PI430048170 | 4.84E-05 | 7.119804 | 6.149929 | 1.24454 |
| CUST_137020_PI430048170 | 7.25E-05 | 6.80568 | 5.509328 | 1.243531 |
| CUST_123321_PI430048170 | 2.33E-02 | 3.254717 | -2.36483 | 1.243521 |
| CUST_131718_PI430048170 | 1.78E-02 | 3.417291 | -2.00665 | 1.242847 |
| CUST_119853_PI430048170 | 3.28E-04 | 5.781781 | 3.329574 | 1.242487 |
| CUST_142928_PI430048170 | 5.38E-05 | 7.034199 | 5.976726 | 1.242484 |
| CUST_141344_PI430048170 | 3.52E-05 | 7.363675 | 6.637641 | 1.242384 |
| CUST_120757_PI430048170 | 8.01E-04 | 5.225741 | 2.094767 | 1.242267 |
| CUST_7826_PI430048170 | 1.00E-05 | 8.358434 | 8.538769 | 1.242173 |
| CUST_95940_PI430048170 | 6.03E-03 | 4.04395 | -0.59403 | 1.242006 |
| CUST_25076_PI430048170 | 9.41E-03 | 3.789587 | -1.17188 | 1.241833 |
| CUST_97728_PI430048170 | 7.55E-04 | 5.260742 | 2.173351 | 1.241691 |
| CUST_105241_PI430048170 | 8.85E-05 | 6.666741 | 5.221607 | 1.241486 |
| CUST_98851_PI430048170 | 5.33E-05 | 7.042207 | 5.992974 | 1.241123 |
| CUST_230_PI430053867 | 1.26E-02 | 3.61833 | -1.55796 | 1.240944 |
| CUST_133649_PI430048170 | 8.72E-06 | 8.488064 | 8.776101 | 1.240277 |
| CUST_42043_PI430048170 | 8.11E-03 | 3.873748 | -0.98117 | 1.240201 |
| CUST_132271_PI430048170 | 2.04E-03 | 4.667867 | 0.830853 | 1.239317 |
| CUST_38880_PI430048170 | 2.23E-02 | 3.280097 | -2.30923 | 1.239197 |
| CUST_63853_PI430048170 | 2.44E-02 | 3.22522 | -2.42929 | 1.239097 |
| CUST_70879_PI430048170 | 2.51E-03 | 4.54795 | 0.557196 | 1.238987 |
| CUST_78782_PI430048170 | 2.24E-03 | 4.613978 | 0.707927 | 1.238523 |
| CUST_14988_PI430048170 | 4.78E-04 | 5.545691 | 2.809033 | 1.238256 |
| CUST_67596_PI430048170 | 5.55E-03 | 4.090751 | -0.48732 | 1.237832 |
| CUST_106023_PI430048170 | 7.21E-04 | 5.287272 | 2.232846 | 1.237255 |
| CUST_136879_PI430048170 | 3.64E-05 | 7.330048 | 6.570895 | 1.237138 |
| CUST_112544_PI430048170 | 2.49E-05 | 7.62911 | 7.158813 | 1.237107 |
| CUST_13798_PI430048170 | 1.20E-02 | 3.646512 | -1.49464 | 1.237055 |
| CUST_111285_PI430048170 | 2.62E-03 | 4.522452 | 0.498959 | 1.23674 |
| CUST_38857_PI430048170 | 7.34E-03 | 3.930326 | -0.85267 | 1.236421 |
| CUST_73975_PI430048170 | 2.88E-02 | 3.127715 | -2.64107 | 1.236181 |
| CUST_89020_PI430048170 | 1.88E-03 | 4.717178 | 0.943249 | 1.235974 |
| CUST_116413_PI430048170 | 4.40E-03 | 4.22464 | -0.18164 | 1.235832 |
| CUST_95594_PI430048170 | 2.30E-03 | 4.599222 | 0.674252 | 1.235787 |
| CUST_108315_PI430048170 | 3.42E-05 | 7.386465 | 6.682785 | 1.234983 |
| CUST_135483_PI430048170 | 1.61E-03 | 4.810994 | 1.156804 | 1.234872 |
| CUST_145236_PI430048170 | 1.43E-04 | 6.315669 | 4.482963 | 1.23421 |
| CUST_99150_PI430048170 | 3.63E-04 | 5.719302 | 3.1924 | 1.233627 |
| CUST_71079_PI430048170 | 9.45E-04 | 5.126483 | 1.87138 | 1.233567 |
| CUST_97127_PI430048170 | 2.53E-02 | 3.20298 | -2.47777 | 1.233225 |
| CUST_122872_PI430048170 | 1.87E-03 | 4.719878 | 0.949399 | 1.233185 |
| CUST_106387_PI430048170 | 1.99E-04 | 6.084068 | 3.986895 | 1.232816 |
| CUST_52083_PI430048170 | 3.78E-03 | 4.312062 | 0.018135 | 1.232126 |
| CUST_144012_PI430048170 | 3.48E-02 | 3.013688 | -2.88597 | 1.232113 |
| CUST_143061_PI430048170 | 3.22E-04 | 5.792774 | 3.353663 | 1.231931 |
| CUST_64637_PI430048170 | 7.77E-04 | 5.244556 | 2.137024 | 1.231837 |
| CUST_27451_PI430048170 | 2.57E-03 | 4.534587 | 0.526676 | 1.231227 |
| CUST_27805_PI430048170 | 2.29E-02 | 3.265486 | -2.34126 | 1.23117 |
| CUST_116518_PI430048170 | 5.62E-06 | 8.870237 | 9.462052 | 1.231145 |
| CUST_76330_PI430048170 | 1.08E-04 | 6.518336 | 4.911375 | 1.231085 |
| CUST_139523_PI430048170 | 3.59E-02 | 2.995301 | -2.92516 | 1.23098 |
| CUST_86490_PI430048170 | 9.81E-04 | 5.103511 | 1.819572 | 1.230967 |
| CUST_126918_PI430048170 | 3.90E-03 | 4.29362 | -0.02402 | 1.230643 |
| CUST_77820_PI430048170 | 9.67E-05 | 6.598246 | 5.078791 | 1.229987 |
| CUST_38367_PI430048170 | 2.25E-02 | 3.275439 | -2.31945 | 1.22964 |
| CUST_91542_PI430048170 | 4.34E-03 | 4.233308 | -0.16184 | 1.229275 |
| CUST_127463_PI430048170 | 8.48E-05 | 6.696059 | 5.28254 | 1.229259 |
| CUST_73061_PI430048170 | 2.38E-04 | 5.974197 | 3.749231 | 1.228844 |
| CUST_121431_PI430048170 | 8.14E-06 | 8.56907 | 8.923206 | 1.228713 |
| CUST_104819_PI430048170 | 3.43E-02 | 3.023511 | -2.865 | 1.228668 |
| CUST_109966_PI430048170 | 2.06E-03 | 4.663281 | 0.820395 | 1.228598 |
| CUST_124092_PI430048170 | 1.92E-02 | 3.370891 | -2.10935 | 1.226742 |
| CUST_92863_PI430048170 | 2.81E-03 | 4.48046 | 0.403025 | 1.22657 |
| CUST_140749_PI430048170 | 2.80E-03 | 4.483863 | 0.410799 | 1.225722 |
| CUST_119939_PI430048170 | 1.37E-04 | 6.344184 | 4.543566 | 1.224971 |
| CUST_98066_PI430048170 | 3.85E-03 | 4.299809 | -0.00987 | 1.224756 |
| CUST_117745_PI430048170 | 3.25E-03 | 4.39826 | 0.215164 | 1.224747 |
| CUST_72255_PI430048170 | 1.22E-02 | 3.636514 | -1.51711 | 1.224155 |
| CUST_69774_PI430048170 | 4.66E-04 | 5.563333 | 2.848134 | 1.224096 |
| CUST_46167_PI430048170 | 2.25E-02 | 3.276458 | -2.31721 | 1.223673 |
| CUST_35432_PI430048170 | 3.53E-03 | 4.351192 | 0.107577 | 1.223596 |
| CUST_49031_PI430048170 | 8.43E-04 | 5.193982 | 2.023376 | 1.223562 |
| CUST_90428_PI430048170 | 9.34E-05 | 6.623163 | 5.13082 | 1.223281 |
| CUST_111101_PI430048170 | 3.69E-02 | 2.978233 | -2.96145 | 1.223232 |
| CUST_21558_PI430048170 | 1.91E-02 | 3.374293 | -2.10183 | 1.222607 |
| CUST_55970_PI430048170 | 1.92E-03 | 4.704976 | 0.915444 | 1.222431 |
| CUST_33912_PI430048170 | 2.83E-02 | 3.139081 | -2.61649 | 1.222077 |
| CUST_132257_PI430048170 | 3.10E-03 | 4.424467 | 0.275065 | 1.220981 |
| CUST_56458_PI430048170 | 2.06E-04 | 6.063798 | 3.943159 | 1.220841 |
| CUST_132887_PI430048170 | 1.44E-02 | 3.540517 | -1.73228 | 1.220821 |
| CUST_25007_PI430048170 | 4.54E-03 | 4.206487 | -0.22312 | 1.220741 |
| CUST_40795_PI430048170 | 2.34E-02 | 3.252255 | -2.37022 | 1.220697 |
| CUST_134858_PI430048170 | 6.09E-04 | 5.392866 | 2.469038 | 1.220636 |
| CUST_71998_PI430048170 | 7.92E-05 | 6.742185 | 5.378168 | 1.220549 |
| CUST_106339_PI430048170 | 6.85E-03 | 3.969532 | -0.7635 | 1.21971 |
| CUST_33600_PI430048170 | 3.66E-02 | 2.983855 | -2.9495 | 1.219503 |
| CUST_141245_PI430048170 | 8.24E-03 | 3.864331 | -1.00253 | 1.219314 |
| CUST_14944_PI430048170 | 2.17E-02 | 3.298328 | -2.26922 | 1.21846 |
| CUST_35747_PI430048170 | 5.29E-04 | 5.480708 | 2.664742 | 1.218187 |
| CUST_138571_PI430048170 | 6.54E-03 | 3.997207 | -0.70051 | 1.217628 |
| CUST_104527_PI430048170 | 6.52E-04 | 5.351963 | 2.377667 | 1.217107 |
| CUST_97346_PI430048170 | 3.69E-03 | 4.325825 | 0.049594 | 1.216895 |
| CUST_97920_PI430048170 | 1.23E-03 | 4.967996 | 1.513191 | 1.216637 |
| CUST_122453_PI430048170 | 2.04E-02 | 3.332975 | -2.193 | 1.216249 |
| CUST_127594_PI430048170 | 1.02E-02 | 3.742726 | -1.27781 | 1.215911 |
| CUST_71108_PI430048170 | 4.85E-04 | 5.536593 | 2.788857 | 1.215853 |
| CUST_45083_PI430048170 | 3.94E-02 | 2.938005 | -3.04669 | 1.214829 |
| CUST_111875_PI430048170 | 5.32E-04 | 5.47814 | 2.65903 | 1.214647 |
| CUST_53633_PI430048170 | 3.30E-03 | 4.390015 | 0.196318 | 1.21452 |
| CUST_63297_PI430048170 | 2.23E-02 | 3.281506 | -2.30614 | 1.214445 |
| CUST_57077_PI430048170 | 2.36E-02 | 3.247274 | -2.38111 | 1.214384 |
| CUST_98181_PI430048170 | 8.95E-03 | 3.818554 | -1.1063 | 1.214296 |
| CUST_21709_PI430048170 | 1.84E-02 | 3.394717 | -2.05666 | 1.213804 |
| CUST_53176_PI430048170 | 1.55E-02 | 3.49683 | -1.82981 | 1.213787 |
| CUST_130011_PI430048170 | 1.26E-04 | 6.409287 | 4.681531 | 1.213402 |
| CUST_128225_PI430048170 | 1.33E-03 | 4.920646 | 1.405857 | 1.213238 |
| CUST_35419_PI430048170 | 1.20E-05 | 8.191823 | 8.230228 | 1.21288 |
| CUST_34523_PI430048170 | 2.12E-02 | 3.311502 | -2.24026 | 1.212377 |
| CUST_65211_PI430048170 | 2.31E-02 | 3.259092 | -2.35526 | 1.211928 |
| CUST_53810_PI430048170 | 3.13E-05 | 7.461811 | 6.831505 | 1.211599 |
| CUST_119446_PI430048170 | 3.54E-04 | 5.734823 | 3.226518 | 1.211464 |
| CUST_120514_PI430048170 | 4.47E-02 | 2.857338 | -3.21625 | 1.210785 |
| CUST_135423_PI430048170 | 2.34E-02 | 3.250895 | -2.37319 | 1.210231 |
| CUST_9309_PI430048170 | 1.38E-02 | 3.564202 | -1.6793 | 1.210095 |
| CUST_42259_PI430048170 | 6.55E-03 | 3.995963 | -0.70334 | 1.210058 |
| CUST_127172_PI430048170 | 1.32E-03 | 4.924999 | 1.41573 | 1.209311 |
| CUST_36373_PI430048170 | 1.76E-02 | 3.422248 | -1.99565 | 1.209194 |
| CUST_43740_PI430048170 | 1.39E-03 | 4.893821 | 1.344991 | 1.209164 |
| CUST_144375_PI430048170 | 3.32E-02 | 3.043469 | -2.82231 | 1.209007 |
| CUST_110482_PI430048170 | 2.01E-03 | 4.678757 | 0.855682 | 1.208504 |
| CUST_144918_PI430048170 | 5.06E-06 | 8.953372 | 9.608575 | 1.208477 |
| CUST_66121_PI430048170 | 2.47E-04 | 5.951068 | 3.699016 | 1.208382 |
| CUST_17827_PI430048170 | 1.11E-02 | 3.69175 | -1.39281 | 1.208215 |
| CUST_58620_PI430048170 | 4.24E-04 | 5.618922 | 2.971127 | 1.207918 |
| CUST_7184_PI430048170 | 1.97E-03 | 4.688485 | 0.877859 | 1.207629 |
| CUST_57741_PI430048170 | 6.92E-05 | 6.841433 | 5.582935 | 1.207482 |
| CUST_102171_PI430048170 | 1.52E-03 | 4.842356 | 1.228103 | 1.207351 |
| CUST_73558_PI430048170 | 9.97E-04 | 5.093526 | 1.797041 | 1.207066 |
| CUST_49937_PI430048170 | 3.40E-03 | 4.371547 | 0.154104 | 1.206624 |
| CUST_57692_PI430048170 | 3.27E-03 | 4.395721 | 0.209361 | 1.206517 |
| CUST_52636_PI430048170 | 2.91E-04 | 5.850064 | 3.478986 | 1.206058 |
| CUST_74534_PI430048170 | 1.38E-03 | 4.899824 | 1.358614 | 1.205905 |
| CUST_130977_PI430048170 | 1.74E-03 | 4.764547 | 1.051125 | 1.205686 |
| CUST_25524_PI430048170 | 9.18E-03 | 3.804078 | -1.13908 | 1.205558 |
| CUST_114106_PI430048170 | 9.93E-05 | 6.576323 | 5.032948 | 1.204992 |
| CUST_110087_PI430048170 | 8.60E-05 | 6.687455 | 5.264669 | 1.204918 |
| CUST_55558_PI430048170 | 3.31E-04 | 5.776223 | 3.317387 | 1.204528 |
| CUST_128095_PI430048170 | 6.96E-04 | 5.308959 | 2.281437 | 1.204272 |
| CUST_134302_PI430048170 | 9.89E-03 | 3.759551 | -1.2398 | 1.204143 |
| CUST_29352_PI430048170 | 1.02E-03 | 5.077012 | 1.759761 | 1.20397 |
| CUST_136458_PI430048170 | 6.49E-05 | 6.887026 | 5.676543 | 1.203386 |
| CUST_125504_PI430048170 | 1.16E-02 | 3.66574 | -1.45138 | 1.203158 |
| CUST_47229_PI430048170 | 1.56E-02 | 3.491087 | -1.84261 | 1.203136 |
| CUST_100120_PI430048170 | 2.82E-02 | 3.140136 | -2.61421 | 1.202814 |
| CUST_34675_PI430048170 | 1.72E-02 | 3.434626 | -1.96819 | 1.20212 |
| CUST_75435_PI430048170 | 3.92E-04 | 5.666439 | 3.076007 | 1.202008 |
| CUST_133508_PI430048170 | 2.10E-02 | 3.315964 | -2.23045 | 1.201974 |
| CUST_107946_PI430048170 | 1.31E-05 | 8.132999 | 8.120349 | 1.201803 |
| CUST_58591_PI430048170 | 1.08E-02 | 3.709993 | -1.35169 | 1.200841 |
| CUST_64519_PI430048170 | 1.95E-04 | 6.098327 | 4.017631 | 1.200774 |
| CUST_38553_PI430048170 | 2.78E-03 | 4.487233 | 0.418501 | 1.200603 |
| CUST_138262_PI430048170 | 7.67E-06 | 8.625023 | 9.024276 | 1.20045 |
| CUST_59870_PI430048170 | 1.20E-04 | 6.441008 | 4.748553 | 1.200279 |
| CUST_95085_PI430048170 | 2.37E-02 | 3.245121 | -2.38582 | 1.200214 |
| CUST_66355_PI430048170 | 1.38E-02 | 3.567109 | -1.67279 | 1.2001 |
| CUST_81006_PI430048170 | 1.80E-02 | 3.408965 | -2.0251 | 1.199607 |
| CUST_25315_PI430048170 | 2.44E-02 | 3.226416 | -2.42668 | 1.199362 |
| CUST_28770_PI430048170 | 3.38E-05 | 7.399579 | 6.708727 | 1.199171 |
| CUST_72765_PI430048170 | 5.98E-04 | 5.403842 | 2.493532 | 1.199064 |
| CUST_108652_PI430048170 | 3.76E-04 | 5.694603 | 3.138055 | 1.198709 |
| CUST_111538_PI430048170 | 4.68E-02 | 2.828136 | -3.27716 | 1.198266 |
| CUST_125423_PI430048170 | 8.83E-03 | 3.827324 | -1.08644 | 1.198089 |
| CUST_138464_PI430048170 | 1.35E-04 | 6.360122 | 4.577393 | 1.197983 |
| CUST_135241_PI430048170 | 3.61E-03 | 4.337651 | 0.076624 | 1.197909 |
| CUST_34192_PI430048170 | 2.15E-02 | 3.301312 | -2.26266 | 1.197859 |
| CUST_53919_PI430048170 | 6.43E-04 | 5.359548 | 2.394621 | 1.197704 |
| CUST_54278_PI430048170 | 4.13E-02 | 2.907396 | -3.11125 | 1.197574 |
| CUST_8279_PI430048170 | 8.22E-03 | 3.865839 | -0.99911 | 1.19754 |
| CUST_93420_PI430048170 | 1.53E-02 | 3.50279 | -1.81652 | 1.1973 |
| CUST_31023_PI430048170 | 1.29E-03 | 4.940547 | 1.450986 | 1.197101 |
| CUST_57616_PI430048170 | 6.34E-04 | 5.368316 | 2.414214 | 1.196972 |
| CUST_78213_PI430048170 | 3.88E-02 | 2.947376 | -3.02688 | 1.196419 |
| CUST_91413_PI430048170 | 1.34E-05 | 8.116343 | 8.089146 | 1.196332 |
| CUST_126182_PI430048170 | 1.30E-02 | 3.597526 | -1.60464 | 1.196278 |
| CUST_96876_PI430048170 | 5.10E-03 | 4.13917 | -0.37683 | 1.195879 |
| CUST_71344_PI430048170 | 3.26E-05 | 7.429085 | 6.767009 | 1.195706 |
| CUST_59581_PI430048170 | 9.60E-04 | 5.116767 | 1.849472 | 1.195345 |
| CUST_35469_PI430048170 | 3.64E-04 | 5.71528 | 3.183555 | 1.19491 |
| CUST_142280_PI430048170 | 1.13E-03 | 5.017581 | 1.625439 | 1.19428 |
| CUST_115131_PI430048170 | 1.53E-02 | 3.50436 | -1.81301 | 1.193658 |
| CUST_88691_PI430048170 | 1.39E-03 | 4.893566 | 1.34441 | 1.193259 |
| CUST_20025_PI430048170 | 9.09E-03 | 3.809731 | -1.12629 | 1.192818 |
| CUST_78226_PI430048170 | 4.91E-03 | 4.16157 | -0.3257 | 1.191925 |
| CUST_133616_PI430048170 | 4.65E-02 | 2.831878 | -3.26937 | 1.191895 |
| CUST_88613_PI430048170 | 1.43E-04 | 6.313011 | 4.477308 | 1.191844 |
| CUST_33436_PI430048170 | 5.70E-03 | 4.076061 | -0.52082 | 1.191787 |
| CUST_49850_PI430048170 | 3.97E-05 | 7.268551 | 6.448412 | 1.191752 |
| CUST_36108_PI430048170 | 8.71E-03 | 3.834488 | -1.0702 | 1.191322 |
| CUST_4974_PI430048170 | 1.44E-04 | 6.309126 | 4.469041 | 1.191245 |
| CUST_65740_PI430048170 | 9.09E-04 | 5.149196 | 1.922565 | 1.191221 |
| CUST_111009_PI430048170 | 1.32E-02 | 3.591557 | -1.61802 | 1.191147 |
| CUST_128690_PI430048170 | 4.83E-03 | 4.171286 | -0.30351 | 1.191117 |
| CUST_59239_PI430048170 | 6.25E-05 | 6.91776 | 5.739482 | 1.190244 |
| CUST_134381_PI430048170 | 3.42E-04 | 5.756721 | 3.274605 | 1.19002 |
| CUST_88709_PI430048170 | 2.49E-05 | 7.628349 | 7.157333 | 1.189862 |
| CUST_112596_PI430048170 | 2.38E-03 | 4.578403 | 0.626729 | 1.189797 |
| CUST_93826_PI430048170 | 1.35E-02 | 3.579618 | -1.64478 | 1.189491 |
| CUST_19859_PI430048170 | 1.48E-04 | 6.29297 | 4.434646 | 1.189438 |
| CUST_144909_PI430048170 | 4.58E-02 | 2.841829 | -3.24863 | 1.189031 |
| CUST_54550_PI430048170 | 1.56E-04 | 6.256121 | 4.356068 | 1.188708 |
| CUST_129516_PI430048170 | 3.70E-03 | 4.323365 | 0.04397 | 1.188613 |
| CUST_58750_PI430048170 | 1.73E-02 | 3.432276 | -1.9734 | 1.188381 |
| CUST_100278_PI430048170 | 4.05E-02 | 2.92006 | -3.08457 | 1.188101 |
| CUST_64269_PI430048170 | 1.55E-03 | 4.831953 | 1.204457 | 1.187731 |
| CUST_19057_PI430048170 | 2.90E-03 | 4.462288 | 0.3615 | 1.187179 |
| CUST_25070_PI430048170 | 3.98E-02 | 2.931772 | -3.05986 | 1.187055 |
| CUST_122342_PI430048170 | 4.02E-02 | 2.925101 | -3.07394 | 1.186872 |
| CUST_93798_PI430048170 | 2.19E-03 | 4.628166 | 0.740299 | 1.185628 |
| CUST_131572_PI430048170 | 1.17E-03 | 4.995451 | 1.575362 | 1.185386 |
| CUST_120469_PI430048170 | 1.17E-02 | 3.662535 | -1.4586 | 1.184709 |
| CUST_132556_PI430048170 | 5.32E-06 | 8.913004 | 9.537546 | 1.184513 |
| CUST_113466_PI430048170 | 1.03E-05 | 8.336748 | 8.498833 | 1.184384 |
| CUST_138964_PI430048170 | 5.64E-04 | 5.442849 | 2.580484 | 1.184074 |
| CUST_82087_PI430048170 | 3.07E-05 | 7.474304 | 6.856085 | 1.184048 |
| CUST_26704_PI430048170 | 5.70E-03 | 4.075587 | -0.5219 | 1.18337 |
| CUST_145212_PI430048170 | 4.33E-03 | 4.233854 | -0.1606 | 1.183144 |
| CUST_16906_PI430048170 | 1.80E-02 | 3.410289 | -2.02217 | 1.182777 |
| CUST_71197_PI430048170 | 5.77E-04 | 5.42732 | 2.545885 | 1.182382 |
| CUST_61889_PI430048170 | 1.46E-05 | 8.058881 | 7.981198 | 1.182359 |
| CUST_44874_PI430048170 | 6.33E-03 | 4.017662 | -0.65393 | 1.1822 |
| CUST_124467_PI430048170 | 3.13E-05 | 7.462623 | 6.833104 | 1.182181 |
| CUST_55032_PI430048170 | 2.45E-05 | 7.652077 | 7.203431 | 1.181988 |
| CUST_137322_PI430048170 | 6.07E-04 | 5.394937 | 2.473662 | 1.181956 |
| CUST_136053_PI430048170 | 2.69E-03 | 4.506173 | 0.461772 | 1.181572 |
| CUST_109490_PI430048170 | 2.18E-02 | 3.295119 | -2.27627 | 1.181498 |
| CUST_141355_PI430048170 | 7.66E-03 | 3.906574 | -0.90664 | 1.181161 |
| CUST_34794_PI430048170 | 3.56E-04 | 5.731488 | 3.219189 | 1.180402 |
| CUST_35447_PI430048170 | 2.31E-02 | 3.260746 | -2.35164 | 1.179531 |
| CUST_120992_PI430048170 | 9.80E-03 | 3.764982 | -1.22753 | 1.179362 |
| CUST_77741_PI430048170 | 1.85E-04 | 6.139054 | 4.105282 | 1.17902 |
| CUST_65316_PI430048170 | 2.04E-03 | 4.669224 | 0.833946 | 1.178297 |
| CUST_144989_PI430048170 | 1.70E-02 | 3.442368 | -1.951 | 1.178128 |
| CUST_5051_PI430048170 | 1.22E-04 | 6.431649 | 4.728793 | 1.17803 |
| CUST_13598_PI430048170 | 2.74E-02 | 3.157851 | -2.57584 | 1.177804 |
| CUST_125817_PI430048170 | 1.13E-04 | 6.482376 | 4.835756 | 1.177524 |
| CUST_103475_PI430048170 | 3.45E-03 | 4.36442 | 0.137815 | 1.177183 |
| CUST_70412_PI430048170 | 1.95E-04 | 6.097937 | 4.016792 | 1.177007 |
| CUST_100362_PI430048170 | 3.73E-03 | 4.31911 | 0.034246 | 1.176517 |
| CUST_124231_PI430048170 | 3.34E-03 | 4.381111 | 0.175968 | 1.175693 |
| CUST_97882_PI430048170 | 6.44E-03 | 4.005798 | -0.68095 | 1.175212 |
| CUST_145530_PI430048170 | 2.58E-04 | 5.925966 | 3.644444 | 1.174633 |
| CUST_119376_PI430048170 | 1.73E-04 | 6.190349 | 4.215381 | 1.17419 |
| CUST_4966_PI430048170 | 1.43E-04 | 6.314824 | 4.481166 | 1.174069 |
| CUST_119266_PI430048170 | 2.88E-04 | 5.859282 | 3.499118 | 1.173938 |
| CUST_83798_PI430048170 | 2.62E-03 | 4.520422 | 0.494322 | 1.173771 |
| CUST_138509_PI430048170 | 2.35E-04 | 5.982505 | 3.767255 | 1.173731 |
| CUST_6032_PI430048170 | 2.01E-02 | 3.342011 | -2.17309 | 1.173498 |
| CUST_118934_PI430048170 | 2.71E-04 | 5.893919 | 3.574667 | 1.173076 |
| CUST_93672_PI430048170 | 1.20E-03 | 4.981582 | 1.543961 | 1.172888 |
| CUST_132039_PI430048170 | 2.52E-04 | 5.940868 | 3.676851 | 1.172846 |
| CUST_82338_PI430048170 | 3.28E-03 | 4.393747 | 0.20485 | 1.172546 |
| CUST_3452_PI430048170 | 9.22E-05 | 6.633237 | 5.151829 | 1.172387 |
| CUST_58602_PI430048170 | 9.94E-07 | 10.39153 | 11.9969 | 1.17217 |
| CUST_33364_PI430048170 | 1.67E-02 | 3.454432 | -1.92419 | 1.171871 |
| CUST_17284_PI430048170 | 5.29E-03 | 4.118285 | -0.4245 | 1.171752 |
| CUST_139423_PI430048170 | 6.51E-04 | 5.352769 | 2.379467 | 1.171412 |
| CUST_26964_PI430048170 | 5.75E-04 | 5.430632 | 2.553267 | 1.171253 |
| CUST_87660_PI430048170 | 5.84E-03 | 4.062165 | -0.55251 | 1.170809 |
| CUST_25189_PI430048170 | 1.06E-04 | 6.526022 | 4.927515 | 1.170418 |
| CUST_122604_PI430048170 | 1.42E-02 | 3.547712 | -1.7162 | 1.17031 |
| CUST_69625_PI430048170 | 1.36E-04 | 6.352858 | 4.561979 | 1.17013 |
| CUST_11653_PI430048170 | 2.20E-03 | 4.624625 | 0.73222 | 1.170118 |
| CUST_70904_PI430048170 | 7.32E-05 | 6.798751 | 5.495042 | 1.169957 |
| CUST_68113_PI430048170 | 2.83E-03 | 4.476481 | 0.393933 | 1.169923 |
| CUST_122363_PI430048170 | 1.11E-02 | 3.690751 | -1.39506 | 1.169735 |
| CUST_132842_PI430048170 | 1.25E-04 | 6.41194 | 4.687142 | 1.169734 |
| CUST_91953_PI430048170 | 2.77E-02 | 3.150703 | -2.59133 | 1.16952 |
| CUST_95661_PI430048170 | 7.32E-03 | 3.931856 | -0.84919 | 1.169342 |
| CUST_102331_PI430048170 | 1.38E-03 | 4.901784 | 1.363063 | 1.16901 |
| CUST_71027_PI430048170 | 3.37E-05 | 7.403798 | 6.717068 | 1.168687 |
| CUST_81336_PI430048170 | 3.40E-03 | 4.37184 | 0.154775 | 1.168604 |
| CUST_109793_PI430048170 | 8.09E-04 | 5.219707 | 2.081211 | 1.168565 |
| CUST_144699_PI430048170 | 1.99E-05 | 7.818701 | 7.524871 | 1.167462 |
| CUST_141706_PI430048170 | 5.57E-04 | 5.451827 | 2.600478 | 1.16744 |
| CUST_93471_PI430048170 | 4.51E-02 | 2.850863 | -3.22978 | 1.167219 |
| CUST_63436_PI430048170 | 5.91E-04 | 5.411544 | 2.510713 | 1.166743 |
| CUST_140103_PI430048170 | 1.93E-03 | 4.702723 | 0.91031 | 1.166215 |
| CUST_83104_PI430048170 | 8.37E-04 | 5.198459 | 2.033445 | 1.166211 |
| CUST_93456_PI430048170 | 7.34E-05 | 6.796449 | 5.490294 | 1.166 |
| CUST_135821_PI430048170 | 8.99E-06 | 8.44663 | 8.7005 | 1.165642 |
| CUST_125274_PI430048170 | 6.21E-05 | 6.931085 | 5.766727 | 1.165374 |
| CUST_72490_PI430048170 | 3.19E-04 | 5.798652 | 3.366539 | 1.165136 |
| CUST_96017_PI430048170 | 1.91E-03 | 4.709471 | 0.925688 | 1.165028 |
| CUST_103416_PI430048170 | 1.89E-02 | 3.380699 | -2.08767 | 1.16474 |
| CUST_124996_PI430048170 | 1.54E-03 | 4.836928 | 1.215766 | 1.163952 |
| CUST_131720_PI430048170 | 2.76E-04 | 5.881773 | 3.548191 | 1.16372 |
| CUST_122964_PI430048170 | 1.26E-04 | 6.406351 | 4.675322 | 1.163637 |
| CUST_118174_PI430048170 | 2.38E-03 | 4.577404 | 0.624447 | 1.163475 |
| CUST_97029_PI430048170 | 1.87E-02 | 3.38655 | -2.07473 | 1.163393 |
| CUST_135417_PI430048170 | 1.41E-03 | 4.887009 | 1.329528 | 1.163094 |
| CUST_97869_PI430048170 | 4.50E-04 | 5.582737 | 2.891101 | 1.162759 |
| CUST_98461_PI430048170 | 1.27E-05 | 8.153666 | 8.15901 | 1.16201 |
| CUST_56232_PI430048170 | 8.93E-03 | 3.820253 | -1.10246 | 1.161882 |
| CUST_120669_PI430048170 | 2.64E-05 | 7.578805 | 7.060817 | 1.161829 |
| CUST_100294_PI430048170 | 1.16E-05 | 8.228284 | 8.298086 | 1.161698 |
| CUST_71846_PI430048170 | 4.15E-04 | 5.630602 | 2.996929 | 1.161565 |
| CUST_110296_PI430048170 | 6.49E-05 | 6.884377 | 5.671112 | 1.161372 |
| CUST_143792_PI430048170 | 2.56E-04 | 5.931376 | 3.656213 | 1.161072 |
| CUST_36255_PI430048170 | 8.42E-04 | 5.194229 | 2.023931 | 1.161061 |
| CUST_26333_PI430048170 | 6.30E-04 | 5.371784 | 2.421964 | 1.161011 |
| CUST_116647_PI430048170 | 2.03E-03 | 4.670372 | 0.836565 | 1.160857 |
| CUST_131973_PI430048170 | 2.92E-03 | 4.45923 | 0.354513 | 1.160846 |
| CUST_10641_PI430048170 | 3.60E-03 | 4.340148 | 0.082333 | 1.160657 |
| CUST_101440_PI430048170 | 6.22E-04 | 5.380184 | 2.440726 | 1.160402 |
| CUST_59338_PI430048170 | 2.49E-03 | 4.551169 | 0.564546 | 1.15981 |
| CUST_78494_PI430048170 | 7.16E-06 | 8.676162 | 9.116266 | 1.15953 |
| CUST_93689_PI430048170 | 3.81E-03 | 4.306483 | 0.005383 | 1.159516 |
| CUST_17982_PI430048170 | 1.44E-02 | 3.538398 | -1.73702 | 1.159351 |
| CUST_137883_PI430048170 | 2.60E-03 | 4.526734 | 0.50874 | 1.159234 |
| CUST_57652_PI430048170 | 4.51E-05 | 7.168762 | 6.248518 | 1.158806 |
| CUST_3302_PI430048170 | 1.23E-02 | 3.632838 | -1.52537 | 1.158558 |
| CUST_23439_PI430048170 | 1.35E-04 | 6.359053 | 4.575126 | 1.158529 |
| CUST_121537_PI430048170 | 6.52E-03 | 3.998605 | -0.69733 | 1.158514 |
| CUST_129699_PI430048170 | 3.54E-03 | 4.349235 | 0.103105 | 1.158252 |
| CUST_71321_PI430048170 | 2.78E-02 | 3.149013 | -2.59499 | 1.158091 |
| CUST_101445_PI430048170 | 4.05E-04 | 5.64424 | 3.027038 | 1.157448 |
| CUST_51886_PI430048170 | 4.25E-03 | 4.245076 | -0.13495 | 1.15704 |
| CUST_59158_PI430048170 | 2.54E-06 | 9.560953 | 10.65081 | 1.15687 |
| CUST_36741_PI430048170 | 7.23E-05 | 6.807975 | 5.514058 | 1.15638 |
| CUST_21341_PI430048170 | 3.12E-02 | 3.080299 | -2.74329 | 1.15613 |
| CUST_6790_PI430048170 | 3.26E-05 | 7.429553 | 6.767933 | 1.155648 |
| CUST_136477_PI430048170 | 3.36E-02 | 3.035464 | -2.83945 | 1.155335 |
| CUST_65718_PI430048170 | 8.86E-04 | 5.164176 | 1.956301 | 1.155236 |
| CUST_5055_PI430048170 | 2.46E-05 | 7.647681 | 7.194898 | 1.155228 |
| CUST_76567_PI430048170 | 3.14E-02 | 3.076261 | -2.75197 | 1.15498 |
| CUST_119159_PI430048170 | 1.46E-04 | 6.302371 | 4.454665 | 1.154705 |
| CUST_119260_PI430048170 | 2.29E-06 | 9.670982 | 10.83426 | 1.154515 |
| CUST_87126_PI430048170 | 6.30E-04 | 5.372247 | 2.422997 | 1.15444 |
| CUST_66572_PI430048170 | 4.50E-02 | 2.852052 | -3.2273 | 1.154105 |
| CUST_59514_PI430048170 | 1.08E-02 | 3.706345 | -1.35991 | 1.154051 |
| CUST_29441_PI430048170 | 4.43E-04 | 5.592603 | 2.912934 | 1.153853 |
| CUST_76977_PI430048170 | 3.33E-02 | 3.040894 | -2.82783 | 1.153773 |
| CUST_50052_PI430048170 | 1.71E-02 | 3.439944 | -1.95638 | 1.152485 |
| CUST_92543_PI430048170 | 1.90E-02 | 3.375739 | -2.09864 | 1.152198 |
| CUST_73570_PI430048170 | 4.50E-04 | 5.583461 | 2.892703 | 1.151877 |
| CUST_66392_PI430048170 | 1.78E-03 | 4.75175 | 1.02199 | 1.151606 |
| CUST_75240_PI430048170 | 1.03E-02 | 3.7385 | -1.28736 | 1.151395 |
| CUST_23379_PI430048170 | 1.84E-02 | 3.394305 | -2.05757 | 1.151102 |
| CUST_137112_PI430048170 | 9.50E-03 | 3.783358 | -1.18597 | 1.151063 |
| CUST_94821_PI430048170 | 2.93E-03 | 4.456216 | 0.347624 | 1.150388 |
| CUST_111834_PI430048170 | 3.62E-02 | 2.989495 | -2.93751 | 1.150338 |
| CUST_131354_PI430048170 | 3.21E-06 | 9.356026 | 10.30485 | 1.150212 |
| CUST_73213_PI430048170 | 8.48E-05 | 6.695548 | 5.281478 | 1.149658 |
| CUST_60574_PI430048170 | 2.58E-05 | 7.593649 | 7.089771 | 1.149296 |
| CUST_75991_PI430048170 | 8.98E-04 | 5.155854 | 1.937562 | 1.148867 |
| CUST_19332_PI430048170 | 4.57E-02 | 2.843531 | -3.24508 | 1.148864 |
| CUST_143357_PI430048170 | 3.58E-06 | 9.255824 | 10.13364 | 1.148806 |
| CUST_95199_PI430048170 | 1.17E-03 | 5.00009 | 1.585861 | 1.148662 |
| CUST_39565_PI430048170 | 4.55E-02 | 2.846012 | -3.2399 | 1.148444 |
| CUST_99986_PI430048170 | 1.12E-02 | 3.687235 | -1.40298 | 1.14838 |
| CUST_126721_PI430048170 | 1.93E-03 | 4.703955 | 0.913119 | 1.148257 |
| CUST_137505_PI430048170 | 1.00E-02 | 3.751512 | -1.25797 | 1.147975 |
| CUST_15448_PI430048170 | 1.80E-02 | 3.410676 | -2.02131 | 1.147888 |
| CUST_59725_PI430048170 | 5.11E-03 | 4.137762 | -0.38005 | 1.147757 |
| CUST_127036_PI430048170 | 3.67E-04 | 5.709422 | 3.170669 | 1.147426 |
| CUST_126818_PI430048170 | 6.83E-03 | 3.971444 | -0.75915 | 1.147405 |
| CUST_62199_PI430048170 | 2.03E-03 | 4.672865 | 0.842249 | 1.14728 |
| CUST_126412_PI430048170 | 2.58E-02 | 3.192422 | -2.50076 | 1.146992 |
| CUST_35596_PI430048170 | 2.88E-03 | 4.466411 | 0.370923 | 1.146869 |
| CUST_104695_PI430048170 | 3.04E-02 | 3.096695 | -2.708 | 1.14683 |
| CUST_26492_PI430048170 | 2.60E-03 | 4.525888 | 0.506808 | 1.146652 |
| CUST_135517_PI430048170 | 6.83E-06 | 8.71118 | 9.179045 | 1.146546 |
| CUST_46475_PI430048170 | 4.72E-04 | 5.555002 | 2.829674 | 1.14629 |
| CUST_59521_PI430048170 | 1.64E-03 | 4.797611 | 1.126364 | 1.146066 |
| CUST_139153_PI430048170 | 2.65E-03 | 4.515358 | 0.482753 | 1.146047 |
| CUST_91362_PI430048170 | 6.57E-04 | 5.347128 | 2.366855 | 1.145906 |
| CUST_67275_PI430048170 | 5.31E-03 | 4.115045 | -0.43189 | 1.145579 |
| CUST_118286_PI430048170 | 1.95E-04 | 6.0986 | 4.01822 | 1.145524 |
| CUST_139858_PI430048170 | 4.73E-04 | 5.553431 | 2.826192 | 1.145164 |
| CUST_107900_PI430048170 | 9.83E-03 | 3.763196 | -1.23156 | 1.145026 |
| CUST_99082_PI430048170 | 3.48E-02 | 3.014851 | -2.88349 | 1.144835 |
| CUST_37747_PI430048170 | 6.94E-03 | 3.962291 | -0.77998 | 1.144782 |
| CUST_35551_PI430048170 | 5.98E-03 | 4.049365 | -0.58169 | 1.144504 |
| CUST_100430_PI430048170 | 5.36E-03 | 4.109317 | -0.44496 | 1.14441 |
| CUST_137649_PI430048170 | 3.63E-02 | 2.988997 | -2.93857 | 1.144173 |
| CUST_133997_PI430048170 | 1.01E-03 | 5.087019 | 1.782354 | 1.144137 |
| CUST_47703_PI430048170 | 4.21E-02 | 2.896706 | -3.13374 | 1.14411 |
| CUST_130404_PI430048170 | 4.04E-03 | 4.273621 | -0.06972 | 1.143908 |
| CUST_20454_PI430048170 | 4.61E-02 | 2.837921 | -3.25678 | 1.143818 |
| CUST_73681_PI430048170 | 1.57E-02 | 3.486492 | -1.85284 | 1.143581 |
| CUST_143698_PI430048170 | 5.30E-05 | 7.04749 | 6.003685 | 1.143491 |
| CUST_96003_PI430048170 | 9.93E-05 | 6.576436 | 5.033184 | 1.143103 |
| CUST_7134_PI430048170 | 1.97E-03 | 4.691693 | 0.885172 | 1.141544 |
| CUST_78940_PI430048170 | 2.20E-02 | 3.289347 | -2.28894 | 1.141406 |
| CUST_124274_PI430048170 | 8.97E-03 | 3.817675 | -1.1083 | 1.141261 |
| CUST_50256_PI430048170 | 1.13E-02 | 3.683034 | -1.41245 | 1.141111 |
| CUST_116722_PI430048170 | 5.23E-04 | 5.48808 | 2.68113 | 1.14095 |
| CUST_73242_PI430048170 | 8.87E-03 | 3.824201 | -1.09351 | 1.140012 |
| CUST_115322_PI430048170 | 5.66E-05 | 6.997023 | 5.901187 | 1.139247 |
| CUST_129486_PI430048170 | 5.22E-06 | 8.92933 | 9.5663 | 1.138755 |
| CUST_59339_PI430048170 | 1.69E-03 | 4.779441 | 1.085024 | 1.138474 |
| CUST_40476_PI430048170 | 2.62E-02 | 3.182762 | -2.52176 | 1.138044 |
| CUST_114681_PI430048170 | 6.27E-03 | 4.022192 | -0.64361 | 1.137926 |
| CUST_91130_PI430048170 | 4.54E-03 | 4.206875 | -0.22223 | 1.137783 |
| CUST_92405_PI430048170 | 8.60E-06 | 8.500123 | 8.798058 | 1.13772 |
| CUST_35543_PI430048170 | 3.90E-04 | 5.670671 | 3.085334 | 1.137395 |
| CUST_95530_PI430048170 | 4.43E-02 | 2.864306 | -3.20168 | 1.137333 |
| CUST_20394_PI430048170 | 2.08E-03 | 4.657413 | 0.807014 | 1.137325 |
| CUST_116226_PI430048170 | 4.32E-04 | 5.608893 | 2.94896 | 1.136971 |
| CUST_44243_PI430048170 | 1.40E-02 | 3.557219 | -1.69493 | 1.136803 |
| CUST_90792_PI430048170 | 1.97E-02 | 3.352732 | -2.14944 | 1.136724 |
| CUST_144492_PI430048170 | 3.73E-03 | 4.31888 | 0.03372 | 1.136602 |
| CUST_139235_PI430048170 | 1.16E-03 | 5.003606 | 1.593819 | 1.136578 |
| CUST_134477_PI430048170 | 8.30E-03 | 3.860333 | -1.0116 | 1.135875 |
| CUST_104048_PI430048170 | 1.71E-02 | 3.438099 | -1.96048 | 1.135795 |
| CUST_135409_PI430048170 | 1.19E-05 | 8.206003 | 8.25664 | 1.13541 |
| CUST_143153_PI430048170 | 3.38E-04 | 5.763077 | 3.288552 | 1.135294 |
| CUST_35544_PI430048170 | 3.74E-05 | 7.310863 | 6.532742 | 1.134901 |
| CUST_60201_PI430048170 | 7.85E-05 | 6.748304 | 5.390833 | 1.134358 |
| CUST_54537_PI430048170 | 9.90E-05 | 6.578756 | 5.038038 | 1.134243 |
| CUST_19863_PI430048170 | 9.19E-05 | 6.635975 | 5.157538 | 1.133984 |
| CUST_91947_PI430048170 | 4.73E-03 | 4.183098 | -0.27654 | 1.133885 |
| CUST_22533_PI430048170 | 9.99E-05 | 6.570448 | 5.02065 | 1.133567 |
| CUST_62817_PI430048170 | 8.85E-06 | 8.46729 | 8.738227 | 1.133563 |
| CUST_6006_PI430048170 | 1.55E-03 | 4.833177 | 1.20724 | 1.132701 |
| CUST_126946_PI430048170 | 4.88E-02 | 2.802251 | -3.33093 | 1.132663 |
| CUST_26406_PI430048170 | 3.86E-04 | 5.677799 | 3.101043 | 1.132358 |
| CUST_103145_PI430048170 | 1.82E-04 | 6.152889 | 4.135008 | 1.132343 |
| CUST_74584_PI430048170 | 3.38E-02 | 3.031211 | -2.84854 | 1.132298 |
| CUST_17360_PI430048170 | 8.17E-04 | 5.213247 | 2.066692 | 1.132258 |
| CUST_130921_PI430048170 | 2.55E-03 | 4.537927 | 0.534305 | 1.132065 |
| CUST_130644_PI430048170 | 6.17E-05 | 6.934609 | 5.773929 | 1.132004 |
| CUST_78435_PI430048170 | 2.10E-05 | 7.773712 | 7.438476 | 1.131387 |
| CUST_107553_PI430048170 | 2.97E-03 | 4.448947 | 0.331014 | 1.13133 |
| CUST_119373_PI430048170 | 9.59E-06 | 8.389238 | 8.595382 | 1.131234 |
| CUST_94314_PI430048170 | 1.21E-02 | 3.64121 | -1.50656 | 1.131058 |
| CUST_93748_PI430048170 | 1.06E-02 | 3.717585 | -1.33456 | 1.130698 |
| CUST_12182_PI430048170 | 1.61E-02 | 3.473584 | -1.88159 | 1.130694 |
| CUST_105429_PI430048170 | 1.12E-04 | 6.486501 | 4.844439 | 1.13044 |
| Mouse-PGK1_5 | 1.38E-04 | 6.340549 | 4.535845 | 1.129841 |
| CUST_94439_PI430048170 | 4.21E-02 | 2.896301 | -3.13459 | 1.129692 |
| CUST_70165_PI430048170 | 1.72E-03 | 4.771338 | 1.066583 | 1.12961 |
| CUST_88746_PI430048170 | 1.92E-03 | 4.704946 | 0.915375 | 1.129578 |
| CUST_115936_PI430048170 | 4.92E-05 | 7.105628 | 6.121319 | 1.129283 |
| CUST_49975_PI430048170 | 3.24E-02 | 3.057054 | -2.7932 | 1.129134 |
| CUST_136705_PI430048170 | 1.07E-06 | 10.30059 | 11.85381 | 1.128998 |
| CUST_27768_PI430048170 | 1.51E-02 | 3.511753 | -1.79652 | 1.128451 |
| CUST_56153_PI430048170 | 8.99E-06 | 8.443337 | 8.694482 | 1.128362 |
| CUST_142752_PI430048170 | 4.02E-04 | 5.650033 | 3.039822 | 1.128224 |
| CUST_99463_PI430048170 | 2.09E-02 | 3.319222 | -2.22328 | 1.12767 |
| CUST_54198_PI430048170 | 2.54E-04 | 5.935795 | 3.665821 | 1.127639 |
| CUST_19418_PI430048170 | 2.24E-04 | 6.010963 | 3.828923 | 1.127431 |
| CUST_113980_PI430048170 | 3.53E-03 | 4.351676 | 0.108683 | 1.127279 |
| CUST_15987_PI430048170 | 5.03E-05 | 7.087516 | 6.084722 | 1.127032 |
| CUST_51178_PI430048170 | 1.17E-02 | 3.664202 | -1.45484 | 1.126761 |
| CUST_544_PI430048170 | 4.94E-02 | 2.793623 | -3.34881 | 1.126718 |
| CUST_29378_PI430048170 | 1.71E-03 | 4.774163 | 1.073012 | 1.126622 |
| CUST_135023_PI430048170 | 1.60E-02 | 3.477577 | -1.8727 | 1.126615 |
| CUST_100420_PI430048170 | 9.98E-04 | 5.092577 | 1.794898 | 1.126518 |
| CUST_31218_PI430048170 | 2.75E-02 | 3.155009 | -2.582 | 1.126479 |
| CUST_29313_PI430048170 | 1.19E-03 | 4.986744 | 1.55565 | 1.126444 |
| CUST_144227_PI430048170 | 1.80E-04 | 6.162409 | 4.155451 | 1.12582 |
| CUST_189_PI430053867 | 1.23E-02 | 3.630792 | -1.52997 | 1.125446 |
| CUST_57667_PI430048170 | 1.25E-02 | 3.621466 | -1.55091 | 1.125424 |
| CUST_26029_PI430048170 | 1.04E-02 | 3.729228 | -1.30829 | 1.12524 |
| CUST_27683_PI430048170 | 1.63E-03 | 4.803526 | 1.13982 | 1.125105 |
| CUST_143034_PI430048170 | 1.97E-02 | 3.354439 | -2.14568 | 1.124681 |
| CUST_138113_PI430048170 | 6.54E-04 | 5.350225 | 2.37378 | 1.124236 |
| CUST_109969_PI430048170 | 6.06E-04 | 5.395142 | 2.47412 | 1.124102 |
| CUST_82929_PI430048170 | 6.95E-04 | 5.310636 | 2.285192 | 1.123844 |
| CUST_110903_PI430048170 | 4.00E-05 | 7.261765 | 6.434864 | 1.123362 |
| CUST_60577_PI430048170 | 1.66E-04 | 6.215389 | 4.269005 | 1.123229 |
| CUST_77207_PI430048170 | 8.20E-04 | 5.210671 | 2.060903 | 1.122956 |
| CUST_42486_PI430048170 | 6.52E-04 | 5.35242 | 2.378687 | 1.122929 |
| CUST_125093_PI430048170 | 2.12E-03 | 4.647733 | 0.784936 | 1.122889 |
| CUST_142772_PI430048170 | 3.61E-05 | 7.341729 | 6.594099 | 1.122579 |
| CUST_109404_PI430048170 | 6.92E-05 | 6.841218 | 5.582493 | 1.122567 |
| CUST_59021_PI430048170 | 1.04E-02 | 3.73008 | -1.30636 | 1.122465 |
| CUST_38516_PI430048170 | 6.39E-03 | 4.01066 | -0.66987 | 1.12234 |
| CUST_103474_PI430048170 | 1.92E-03 | 4.705579 | 0.916819 | 1.121679 |
| CUST_115369_PI430048170 | 7.86E-03 | 3.892352 | -0.93894 | 1.121322 |
| CUST_139109_PI430048170 | 2.03E-02 | 3.335709 | -2.18698 | 1.120292 |
| CUST_49824_PI430048170 | 9.71E-04 | 5.1106 | 1.835564 | 1.120033 |
| CUST_94736_PI430048170 | 4.32E-02 | 2.879542 | -3.16977 | 1.1193 |
| CUST_75625_PI430048170 | 2.57E-02 | 3.195232 | -2.49464 | 1.119147 |
| CUST_82086_PI430048170 | 4.33E-05 | 7.206554 | 6.324389 | 1.119102 |
| CUST_126284_PI430048170 | 2.18E-03 | 4.628364 | 0.740752 | 1.118715 |
| CUST_129822_PI430048170 | 9.74E-03 | 3.768705 | -1.21911 | 1.118535 |
| CUST_102008_PI430048170 | 7.21E-03 | 3.941085 | -0.82821 | 1.118222 |
| CUST_462_PI430048170 | 1.17E-03 | 4.996472 | 1.577674 | 1.118176 |
| CUST_84165_PI430048170 | 7.79E-04 | 5.243175 | 2.133924 | 1.118007 |
| CUST_81605_PI430048170 | 7.00E-05 | 6.832266 | 5.564079 | 1.117781 |
| CUST_74149_PI430048170 | 7.81E-03 | 3.895395 | -0.93203 | 1.117752 |
| CUST_142853_PI430048170 | 1.80E-03 | 4.744176 | 1.004743 | 1.116536 |
| CUST_4981_PI430048170 | 5.68E-06 | 8.860633 | 9.445063 | 1.116516 |
| CUST_350_PI430048170 | 4.08E-03 | 4.267918 | -0.08276 | 1.115684 |
| CUST_125400_PI430048170 | 4.45E-05 | 7.180919 | 6.272946 | 1.115662 |
| Rat-PGK1_5 | 1.10E-04 | 6.50531 | 4.884003 | 1.115512 |
| CUST_67571_PI430048170 | 1.08E-02 | 3.7069 | -1.35866 | 1.115377 |
| CUST_84748_PI430048170 | 1.14E-02 | 3.674816 | -1.43095 | 1.115224 |
| CUST_70998_PI430048170 | 1.68E-03 | 4.783588 | 1.094461 | 1.11518 |
| CUST_4644_PI430048170 | 4.40E-06 | 9.07409 | 9.819642 | 1.115006 |
| CUST_110209_PI430048170 | 1.76E-05 | 7.9064 | 7.692453 | 1.114791 |
| CUST_51718_PI430048170 | 9.46E-03 | 3.786286 | -1.17935 | 1.114744 |
| CUST_134565_PI430048170 | 3.12E-05 | 7.463926 | 6.835667 | 1.113934 |
| CUST_84577_PI430048170 | 1.21E-02 | 3.644426 | -1.49933 | 1.113547 |
| CUST_119322_PI430048170 | 1.93E-04 | 6.107513 | 4.037418 | 1.113303 |
| CUST_64381_PI430048170 | 1.20E-02 | 3.649256 | -1.48847 | 1.113023 |
| CUST_69896_PI430048170 | 4.72E-06 | 9.01603 | 9.718378 | 1.112967 |
| CUST_34132_PI430048170 | 3.15E-02 | 3.074865 | -2.75497 | 1.112783 |
| CUST_19330_PI430048170 | 2.92E-02 | 3.119867 | -2.65802 | 1.112137 |
| CUST_62344_PI430048170 | 1.13E-02 | 3.684494 | -1.40916 | 1.111928 |
| CUST_63494_PI430048170 | 2.71E-04 | 5.893867 | 3.574555 | 1.111332 |
| CUST_130917_PI430048170 | 3.68E-06 | 9.231921 | 10.09259 | 1.111316 |
| CUST_8902_PI430048170 | 1.50E-02 | 3.5137 | -1.79218 | 1.111249 |
| CUST_63493_PI430048170 | 6.31E-04 | 5.371049 | 2.420321 | 1.111041 |
| CUST_41587_PI430048170 | 1.21E-02 | 3.6401 | -1.50905 | 1.110775 |
| CUST_35803_PI430048170 | 7.45E-04 | 5.268806 | 2.191441 | 1.110714 |
| CUST_112418_PI430048170 | 3.48E-02 | 3.0141 | -2.88509 | 1.110694 |
| CUST_130935_PI430048170 | 2.35E-05 | 7.684822 | 7.266916 | 1.110542 |
| CUST_133535_PI430048170 | 1.96E-03 | 4.693193 | 0.888591 | 1.11053 |
| CUST_101679_PI430048170 | 4.30E-02 | 2.881868 | -3.16489 | 1.110032 |
| CUST_62535_PI430048170 | 3.98E-05 | 7.26578 | 6.442881 | 1.109873 |
| CUST_9131_PI430048170 | 2.96E-02 | 3.111793 | -2.67545 | 1.109076 |
| CUST_145749_PI430048170 | 1.55E-03 | 4.831826 | 1.204168 | 1.108609 |
| CUST_141639_PI430048170 | 3.50E-02 | 3.010062 | -2.8937 | 1.108605 |
| CUST_96908_PI430048170 | 1.06E-03 | 5.057081 | 1.714742 | 1.107349 |
| CUST_104918_PI430048170 | 8.14E-06 | 8.561621 | 8.909716 | 1.107124 |
| CUST_61443_PI430048170 | 3.42E-02 | 3.023814 | -2.86435 | 1.10653 |
| CUST_94626_PI430048170 | 2.79E-02 | 3.146 | -2.60151 | 1.106388 |
| CUST_67823_PI430048170 | 5.57E-04 | 5.452207 | 2.601324 | 1.10568 |
| CUST_92506_PI430048170 | 4.11E-02 | 2.911599 | -3.1024 | 1.105475 |
| CUST_65423_PI430048170 | 1.10E-05 | 8.265319 | 8.366819 | 1.104999 |
| CUST_105901_PI430048170 | 4.95E-02 | 2.792294 | -3.35156 | 1.104882 |
| CUST_62823_PI430048170 | 3.81E-03 | 4.307431 | 0.007551 | 1.104769 |
| CUST_110979_PI430048170 | 2.48E-03 | 4.554311 | 0.571719 | 1.104705 |
| CUST_111911_PI430048170 | 1.80E-04 | 6.163842 | 4.158527 | 1.104692 |
| CUST_63426_PI430048170 | 5.47E-03 | 4.098668 | -0.46926 | 1.104515 |
| CUST_137401_PI430048170 | 2.21E-04 | 6.017728 | 3.843569 | 1.10435 |
| CUST_19535_PI430048170 | 1.80E-02 | 3.409714 | -2.02344 | 1.10375 |
| CUST_44455_PI430048170 | 1.06E-02 | 3.722052 | -1.32448 | 1.103682 |
| CUST_24014_PI430048170 | 1.85E-03 | 4.727257 | 0.966208 | 1.103582 |
| CUST_64923_PI430048170 | 4.42E-04 | 5.594354 | 2.916808 | 1.103201 |
| CUST_35913_PI430048170 | 9.99E-03 | 3.753147 | -1.25427 | 1.103179 |
| CUST_57343_PI430048170 | 3.40E-02 | 3.029 | -2.85327 | 1.102321 |
| CUST_59448_PI430048170 | 1.26E-04 | 6.404409 | 4.671213 | 1.101932 |
| CUST_91048_PI430048170 | 8.30E-04 | 5.203418 | 2.044596 | 1.101708 |
| CUST_123017_PI430048170 | 4.72E-04 | 5.554322 | 2.828166 | 1.101699 |
| CUST_82339_PI430048170 | 2.83E-02 | 3.138472 | -2.61781 | 1.101539 |
| CUST_132579_PI430048170 | 5.75E-04 | 5.431568 | 2.555354 | 1.101468 |
| CUST_90416_PI430048170 | 8.90E-04 | 5.161054 | 1.949273 | 1.101417 |
| CUST_116219_PI430048170 | 4.46E-03 | 4.217234 | -0.19857 | 1.101091 |
| CUST_24801_PI430048170 | 1.05E-02 | 3.726661 | -1.31408 | 1.101076 |
| CUST_87636_PI430048170 | 1.14E-03 | 5.014612 | 1.618724 | 1.100988 |
| CUST_23918_PI430048170 | 1.15E-02 | 3.670348 | -1.44101 | 1.100856 |
| CUST_81717_PI430048170 | 1.31E-04 | 6.37904 | 4.617501 | 1.09991 |
| CUST_131263_PI430048170 | 1.37E-04 | 6.34857 | 4.552879 | 1.0998 |
| CUST_31704_PI430048170 | 7.23E-03 | 3.939048 | -0.83284 | 1.09975 |
| CUST_26581_PI430048170 | 3.42E-04 | 5.756033 | 3.273095 | 1.09973 |
| CUST_28771_PI430048170 | 1.12E-04 | 6.485741 | 4.842839 | 1.099344 |
| CUST_60942_PI430048170 | 1.55E-02 | 3.49733 | -1.82869 | 1.099004 |
| CUST_23944_PI430048170 | 1.02E-05 | 8.347951 | 8.519471 | 1.098867 |
| CUST_125210_PI430048170 | 2.35E-02 | 3.249283 | -2.37672 | 1.098644 |
| CUST_86077_PI430048170 | 1.72E-04 | 6.193408 | 4.221935 | 1.098364 |
| CUST_16962_PI430048170 | 1.61E-02 | 3.472449 | -1.88411 | 1.098249 |
| CUST_110569_PI430048170 | 1.96E-05 | 7.830971 | 7.548385 | 1.097949 |
| CUST_31414_PI430048170 | 3.74E-02 | 2.970329 | -2.97823 | 1.097758 |
| CUST_10821_PI430048170 | 7.32E-03 | 3.931905 | -0.84908 | 1.097418 |
| CUST_24786_PI430048170 | 4.46E-02 | 2.859703 | -3.21131 | 1.097402 |
| CUST_99_PI430053867 | 1.84E-02 | 3.396278 | -2.0532 | 1.0969 |
| CUST_142536_PI430048170 | 5.19E-03 | 4.129268 | -0.39944 | 1.096852 |
| CUST_97990_PI430048170 | 8.41E-04 | 5.195873 | 2.027629 | 1.09642 |
| CUST_88149_PI430048170 | 1.65E-03 | 4.797022 | 1.125024 | 1.096389 |
| CUST_120773_PI430048170 | 1.61E-04 | 6.234986 | 4.31092 | 1.096097 |
| CUST_55969_PI430048170 | 2.69E-02 | 3.167651 | -2.55458 | 1.096089 |
| CUST_70459_PI430048170 | 2.07E-04 | 6.060277 | 3.935558 | 1.096048 |
| CUST_45667_PI430048170 | 1.24E-03 | 4.964816 | 1.505985 | 1.09597 |
| CUST_28820_PI430048170 | 8.66E-03 | 3.837724 | -1.06287 | 1.095836 |
| CUST_39439_PI430048170 | 3.99E-03 | 4.281521 | -0.05167 | 1.0958 |
| CUST_23937_PI430048170 | 6.40E-05 | 6.897214 | 5.697421 | 1.095777 |
| CUST_23941_PI430048170 | 1.41E-05 | 8.080226 | 8.021353 | 1.095711 |
| CUST_80363_PI430048170 | 5.13E-03 | 4.135671 | -0.38482 | 1.095634 |
| CUST_78931_PI430048170 | 1.50E-02 | 3.51558 | -1.78798 | 1.094911 |
| CUST_57631_PI430048170 | 1.66E-03 | 4.792662 | 1.115105 | 1.094536 |
| CUST_141932_PI430048170 | 2.30E-02 | 3.26095 | -2.35119 | 1.094478 |
| CUST_43563_PI430048170 | 9.84E-03 | 3.763072 | -1.23184 | 1.094282 |
| CUST_78811_PI430048170 | 7.33E-03 | 3.93124 | -0.85059 | 1.093909 |
| CUST_81045_PI430048170 | 3.82E-02 | 2.957966 | -3.00445 | 1.0936 |
| CUST_55605_PI430048170 | 1.01E-02 | 3.747194 | -1.26772 | 1.093396 |
| CUST_51475_PI430048170 | 1.22E-03 | 4.974024 | 1.526845 | 1.093014 |
| CUST_82279_PI430048170 | 1.95E-02 | 3.359772 | -2.13391 | 1.092343 |
| CUST_127116_PI430048170 | 7.32E-04 | 5.279005 | 2.214314 | 1.092301 |
| CUST_110886_PI430048170 | 3.43E-02 | 3.023551 | -2.86491 | 1.092256 |
| CUST_96081_PI430048170 | 1.95E-03 | 4.695708 | 0.894322 | 1.092031 |
| CUST_25310_PI430048170 | 3.77E-04 | 5.691709 | 3.131682 | 1.091836 |
| CUST_134631_PI430048170 | 7.32E-06 | 8.658949 | 9.085344 | 1.091498 |
| CUST_79639_PI430048170 | 1.25E-02 | 3.620711 | -1.55261 | 1.091464 |
| CUST_57427_PI430048170 | 3.82E-02 | 2.957396 | -3.00566 | 1.091026 |
| CUST_45193_PI430048170 | 2.65E-02 | 3.177998 | -2.53211 | 1.091015 |
| CUST_100445_PI430048170 | 7.05E-04 | 5.300589 | 2.262688 | 1.090695 |
| CUST_94023_PI430048170 | 4.16E-05 | 7.237449 | 6.386262 | 1.090202 |
| CUST_74345_PI430048170 | 2.33E-02 | 3.253611 | -2.36725 | 1.090188 |
| CUST_126619_PI430048170 | 4.78E-04 | 5.546185 | 2.810128 | 1.090178 |
| CUST_88640_PI430048170 | 1.18E-05 | 8.216746 | 8.276634 | 1.090136 |
| CUST_119286_PI430048170 | 6.39E-03 | 4.011565 | -0.66781 | 1.089466 |
| CUST_134694_PI430048170 | 1.51E-02 | 3.5114 | -1.79731 | 1.089277 |
| CUST_142142_PI430048170 | 8.85E-05 | 6.666534 | 5.221175 | 1.08918 |
| CUST_118610_PI430048170 | 7.16E-03 | 3.945324 | -0.81857 | 1.088926 |
| CUST_34234_PI430048170 | 2.17E-03 | 4.630847 | 0.746417 | 1.088883 |
| CUST_34122_PI430048170 | 1.11E-03 | 5.028478 | 1.650087 | 1.088711 |
| CUST_71788_PI430048170 | 1.21E-02 | 3.641013 | -1.507 | 1.088642 |
| CUST_120062_PI430048170 | 2.14E-03 | 4.640158 | 0.767659 | 1.08755 |
| CUST_121137_PI430048170 | 1.79E-02 | 3.413211 | -2.01569 | 1.086746 |
| CUST_135392_PI430048170 | 4.03E-04 | 5.649403 | 3.038431 | 1.086695 |
| CUST_144508_PI430048170 | 1.87E-02 | 3.386851 | -2.07406 | 1.086609 |
| CUST_89506_PI430048170 | 3.10E-02 | 3.083951 | -2.73543 | 1.086013 |
| CUST_137409_PI430048170 | 1.89E-02 | 3.380583 | -2.08793 | 1.085941 |
| CUST_35520_PI430048170 | 1.41E-02 | 3.552689 | -1.70506 | 1.085165 |
| CUST_123018_PI430048170 | 6.75E-04 | 5.329037 | 2.326387 | 1.084986 |
| CUST_80370_PI430048170 | 4.02E-02 | 2.925553 | -3.07299 | 1.084752 |
| CUST_74121_PI430048170 | 1.32E-03 | 4.923799 | 1.413007 | 1.084606 |
| CUST_122477_PI430048170 | 4.43E-05 | 7.189251 | 6.289676 | 1.084427 |
| CUST_90489_PI430048170 | 1.47E-02 | 3.527128 | -1.7622 | 1.084171 |
| CUST_70927_PI430048170 | 1.20E-03 | 4.980153 | 1.540726 | 1.083892 |
| CUST_35995_PI430048170 | 9.14E-05 | 6.641751 | 5.169575 | 1.083615 |
| CUST_81310_PI430048170 | 2.09E-04 | 6.054526 | 3.923136 | 1.083563 |
| CUST_33992_PI430048170 | 1.06E-02 | 3.719936 | -1.32926 | 1.083516 |
| CUST_104333_PI430048170 | 6.41E-03 | 4.009174 | -0.67326 | 1.083157 |
| CUST_113825_PI430048170 | 1.70E-03 | 4.775806 | 1.076751 | 1.082855 |
| CUST_37286_PI430048170 | 2.97E-02 | 3.110943 | -2.67729 | 1.082581 |
| CUST_58497_PI430048170 | 6.90E-03 | 3.965976 | -0.77159 | 1.082577 |
| CUST_93418_PI430048170 | 6.66E-05 | 6.866023 | 5.633459 | 1.082427 |
| CUST_78177_PI430048170 | 4.66E-02 | 2.8301 | -3.27307 | 1.081692 |
| CUST_5002_PI430048170 | 4.78E-05 | 7.130168 | 6.170828 | 1.081653 |
| CUST_8988_PI430048170 | 9.95E-04 | 5.09459 | 1.799441 | 1.081551 |
| CUST_48625_PI430048170 | 4.99E-02 | 2.787982 | -3.36048 | 1.081409 |
| CUST_79258_PI430048170 | 4.96E-02 | 2.792045 | -3.35207 | 1.081071 |
| CUST_126_PI430048170 | 4.19E-02 | 2.898802 | -3.12933 | 1.080805 |
| CUST_35425_PI430048170 | 5.57E-05 | 7.012244 | 5.932139 | 1.080442 |
| CUST_31093_PI430048170 | 6.87E-03 | 3.968083 | -0.7668 | 1.080393 |
| CUST_86404_PI430048170 | 3.91E-02 | 2.942984 | -3.03617 | 1.080353 |
| CUST_125596_PI430048170 | 2.40E-02 | 3.236473 | -2.40472 | 1.080174 |
| CUST_54188_PI430048170 | 4.05E-03 | 4.272053 | -0.07331 | 1.079912 |
| CUST_11765_PI430048170 | 2.25E-03 | 4.611028 | 0.701196 | 1.079595 |
| CUST_118423_PI430048170 | 5.35E-05 | 7.039098 | 5.986667 | 1.079506 |
| CUST_69650_PI430048170 | 3.05E-04 | 5.823422 | 3.420753 | 1.078899 |
| CUST_7918_PI430048170 | 1.51E-05 | 8.028051 | 7.923086 | 1.078534 |
| CUST_123322_PI430048170 | 4.07E-02 | 2.918194 | -3.08851 | 1.078436 |
| CUST_69631_PI430048170 | 7.45E-04 | 5.268366 | 2.190454 | 1.07769 |
| CUST_104991_PI430048170 | 2.23E-03 | 4.615704 | 0.711866 | 1.077618 |
| CUST_99505_PI430048170 | 4.93E-02 | 2.795967 | -3.34395 | 1.07754 |
| CUST_126019_PI430048170 | 2.46E-04 | 5.956091 | 3.709927 | 1.077439 |
| CUST_139067_PI430048170 | 2.14E-04 | 6.038057 | 3.887546 | 1.077227 |
| CUST_70606_PI430048170 | 1.06E-04 | 6.527431 | 4.930473 | 1.077144 |
| CUST_60536_PI430048170 | 1.69E-04 | 6.206011 | 4.248931 | 1.076998 |
| CUST_20755_PI430048170 | 1.51E-02 | 3.511559 | -1.79695 | 1.076793 |
| CUST_58298_PI430048170 | 3.83E-03 | 4.303662 | -0.00106 | 1.076117 |
| CUST_109556_PI430048170 | 1.14E-03 | 5.013525 | 1.616264 | 1.075896 |
| CUST_125605_PI430048170 | 3.76E-05 | 7.306669 | 6.524395 | 1.075786 |
| CUST_130574_PI430048170 | 7.57E-03 | 3.913011 | -0.89202 | 1.07558 |
| CUST_22649_PI430048170 | 9.25E-04 | 5.138032 | 1.897411 | 1.075572 |
| CUST_129262_PI430048170 | 6.39E-03 | 4.011634 | -0.66766 | 1.075467 |
| CUST_17474_PI430048170 | 2.82E-03 | 4.479869 | 0.401674 | 1.075427 |
| CUST_66851_PI430048170 | 1.83E-02 | 3.400263 | -2.04438 | 1.075287 |
| CUST_125757_PI430048170 | 1.94E-03 | 4.701171 | 0.906773 | 1.075121 |
| CUST_53511_PI430048170 | 1.82E-04 | 6.153362 | 4.136025 | 1.075023 |
| CUST_131759_PI430048170 | 1.05E-05 | 8.308603 | 8.446902 | 1.074471 |
| CUST_102776_PI430048170 | 1.02E-03 | 5.076107 | 1.757717 | 1.07441 |
| CUST_71900_PI430048170 | 1.26E-04 | 6.401733 | 4.665552 | 1.074136 |
| CUST_24459_PI430048170 | 4.88E-03 | 4.165184 | -0.31745 | 1.073952 |
| CUST_92111_PI430048170 | 2.00E-03 | 4.680366 | 0.85935 | 1.073935 |
| CUST_97055_PI430048170 | 3.15E-02 | 3.073874 | -2.7571 | 1.073781 |
| CUST_34671_PI430048170 | 2.41E-02 | 3.234771 | -2.40844 | 1.073776 |
| CUST_141668_PI430048170 | 7.99E-04 | 5.227937 | 2.099702 | 1.073524 |
| CUST_52477_PI430048170 | 9.80E-04 | 5.104797 | 1.822473 | 1.073334 |
| CUST_61934_PI430048170 | 4.60E-02 | 2.838486 | -3.2556 | 1.073033 |
| CUST_143315_PI430048170 | 2.48E-03 | 4.554556 | 0.572281 | 1.072965 |
| CUST_126795_PI430048170 | 5.44E-05 | 7.026913 | 5.961937 | 1.072911 |
| CUST_77655_PI430048170 | 7.10E-03 | 3.949341 | -0.80943 | 1.072801 |
| CUST_66269_PI430048170 | 3.90E-02 | 2.944626 | -3.03269 | 1.072202 |
| CUST_34017_PI430048170 | 3.73E-03 | 4.318998 | 0.033988 | 1.072043 |
| CUST_82690_PI430048170 | 2.27E-02 | 3.270759 | -2.3297 | 1.071993 |
| CUST_135509_PI430048170 | 5.66E-03 | 4.08054 | -0.51061 | 1.071768 |
| CUST_128627_PI430048170 | 1.49E-04 | 6.288745 | 4.425645 | 1.071201 |
| CUST_125867_PI430048170 | 3.58E-04 | 5.728649 | 3.212949 | 1.070922 |
| CUST_119025_PI430048170 | 3.54E-04 | 5.735465 | 3.227928 | 1.070638 |
| CUST_10647_PI430048170 | 1.94E-04 | 6.103775 | 4.029368 | 1.07046 |
| CUST_12106_PI430048170 | 2.48E-02 | 3.215252 | -2.45103 | 1.070449 |
| CUST_17437_PI430048170 | 1.70E-02 | 3.443893 | -1.94761 | 1.070227 |
| CUST_45192_PI430048170 | 4.23E-02 | 2.893272 | -3.14095 | 1.070061 |
| CUST_131473_PI430048170 | 1.11E-03 | 5.027782 | 1.648513 | 1.069881 |
| CUST_110016_PI430048170 | 1.16E-03 | 5.005301 | 1.597655 | 1.069532 |
| CUST_52576_PI430048170 | 1.19E-02 | 3.649756 | -1.48734 | 1.06939 |
| CUST_69123_PI430048170 | 9.16E-04 | 5.143597 | 1.909951 | 1.069187 |
| CUST_70485_PI430048170 | 6.96E-04 | 5.309376 | 2.28237 | 1.069125 |
| CUST_138056_PI430048170 | 1.30E-03 | 4.936571 | 1.44197 | 1.069101 |
| CUST_127607_PI430048170 | 1.15E-04 | 6.467487 | 4.804397 | 1.068625 |
| CUST_98192_PI430048170 | 4.17E-02 | 2.902278 | -3.12202 | 1.068354 |
| CUST_24469_PI430048170 | 6.77E-03 | 3.977558 | -0.74524 | 1.068256 |
| CUST_127769_PI430048170 | 6.10E-03 | 4.037674 | -0.60833 | 1.067961 |
| CUST_31064_PI430048170 | 3.82E-02 | 2.957688 | -3.00504 | 1.0676 |
| CUST_14783_PI430048170 | 1.48E-02 | 3.524931 | -1.76711 | 1.067288 |
| CUST_58808_PI430048170 | 6.99E-06 | 8.690908 | 9.142723 | 1.067212 |
| CUST_119937_PI430048170 | 2.12E-04 | 6.04397 | 3.900329 | 1.066232 |
| CUST_102747_PI430048170 | 1.14E-03 | 5.012231 | 1.613336 | 1.066221 |
| CUST_36459_PI430048170 | 2.61E-04 | 5.918678 | 3.628587 | 1.066097 |
| CUST_42183_PI430048170 | 6.18E-05 | 6.933892 | 5.772464 | 1.066026 |
| CUST_89030_PI430048170 | 1.06E-02 | 3.720202 | -1.32866 | 1.065616 |
| CUST_79327_PI430048170 | 8.99E-06 | 8.441198 | 8.690571 | 1.065506 |
| CUST_126521_PI430048170 | 1.56E-02 | 3.491394 | -1.84192 | 1.065235 |
| CUST_38193_PI430048170 | 1.44E-02 | 3.538502 | -1.73679 | 1.06491 |
| CUST_44796_PI430048170 | 1.18E-02 | 3.658309 | -1.4681 | 1.064776 |
| CUST_45232_PI430048170 | 4.66E-03 | 4.191302 | -0.2578 | 1.06477 |
| CUST_94569_PI430048170 | 1.74E-05 | 7.911276 | 7.701739 | 1.064673 |
| CUST_101319_PI430048170 | 1.21E-02 | 3.643345 | -1.50176 | 1.064372 |
| CUST_69557_PI430048170 | 3.68E-04 | 5.707681 | 3.166838 | 1.064371 |
| CUST_54305_PI430048170 | 1.95E-02 | 3.358423 | -2.13689 | 1.063773 |
| CUST_123344_PI430048170 | 5.22E-05 | 7.05794 | 6.024865 | 1.06373 |
| CUST_34386_PI430048170 | 4.80E-02 | 2.812468 | -3.30973 | 1.063498 |
| CUST_63135_PI430048170 | 4.14E-02 | 2.905612 | -3.11501 | 1.063281 |
| CUST_35586_PI430048170 | 4.02E-02 | 2.926401 | -3.0712 | 1.062515 |
| CUST_133832_PI430048170 | 5.35E-05 | 7.038628 | 5.985712 | 1.062514 |
| CUST_126720_PI430048170 | 4.34E-03 | 4.232552 | -0.16357 | 1.062415 |
| CUST_129973_PI430048170 | 3.52E-04 | 5.737922 | 3.233326 | 1.062378 |
| CUST_39061_PI430048170 | 4.87E-03 | 4.167201 | -0.31284 | 1.062352 |
| CUST_27041_PI430048170 | 8.22E-04 | 5.208723 | 2.056523 | 1.062341 |
| CUST_139326_PI430048170 | 1.25E-02 | 3.62196 | -1.5498 | 1.062051 |
| CUST_127480_PI430048170 | 1.50E-02 | 3.514104 | -1.79128 | 1.061641 |
| CUST_104685_PI430048170 | 1.54E-03 | 4.83548 | 1.212475 | 1.061394 |
| CUST_125924_PI430048170 | 2.59E-04 | 5.923232 | 3.638497 | 1.061289 |
| CUST_52819_PI430048170 | 6.00E-04 | 5.401633 | 2.488605 | 1.060427 |
| CUST_101189_PI430048170 | 8.67E-04 | 5.177896 | 1.987186 | 1.060346 |
| CUST_47588_PI430048170 | 5.18E-03 | 4.129468 | -0.39898 | 1.060278 |
| CUST_7913_PI430048170 | 1.61E-04 | 6.237747 | 4.316819 | 1.060215 |
| CUST_106737_PI430048170 | 4.07E-03 | 4.269809 | -0.07843 | 1.06012 |
| CUST_34380_PI430048170 | 4.45E-02 | 2.860397 | -3.20985 | 1.05991 |
| CUST_132838_PI430048170 | 9.44E-04 | 5.12672 | 1.871913 | 1.059896 |
| CUST_60094_PI430048170 | 5.12E-03 | 4.136927 | -0.38195 | 1.05986 |
| CUST_135948_PI430048170 | 3.89E-02 | 2.945793 | -3.03023 | 1.059783 |
| CUST_130992_PI430048170 | 4.93E-06 | 8.98098 | 9.657021 | 1.059703 |
| CUST_124872_PI430048170 | 1.60E-02 | 3.478123 | -1.87148 | 1.059699 |
| CUST_135407_PI430048170 | 3.51E-03 | 4.355306 | 0.116981 | 1.059655 |
| CUST_19435_PI430048170 | 6.28E-04 | 5.374333 | 2.427658 | 1.059351 |
| CUST_137534_PI430048170 | 8.46E-03 | 3.849699 | -1.03572 | 1.05924 |
| CUST_92060_PI430048170 | 1.84E-03 | 4.733247 | 0.979854 | 1.059202 |
| CUST_97852_PI430048170 | 5.44E-04 | 5.466207 | 2.632485 | 1.059178 |
| CUST_37774_PI430048170 | 2.71E-02 | 3.163319 | -2.56398 | 1.059112 |
| CUST_7877_PI430048170 | 7.49E-04 | 5.265292 | 2.183559 | 1.058987 |
| CUST_136781_PI430048170 | 1.09E-04 | 6.509587 | 4.892992 | 1.058901 |
| CUST_25043_PI430048170 | 1.85E-02 | 3.392634 | -2.06127 | 1.058565 |
| CUST_114985_PI430048170 | 7.20E-03 | 3.941286 | -0.82775 | 1.058527 |
| CUST_30969_PI430048170 | 2.16E-03 | 4.63654 | 0.759404 | 1.058407 |
| CUST_103332_PI430048170 | 1.08E-03 | 5.047587 | 1.693286 | 1.058384 |
| CUST_144098_PI430048170 | 2.39E-03 | 4.575803 | 0.620793 | 1.058227 |
| CUST_63594_PI430048170 | 4.97E-04 | 5.51978 | 2.751549 | 1.058217 |
| CUST_27328_PI430048170 | 8.34E-05 | 6.707806 | 5.306922 | 1.057614 |
| CUST_99307_PI430048170 | 2.01E-03 | 4.676627 | 0.850827 | 1.057444 |
| CUST_87437_PI430048170 | 3.70E-03 | 4.323958 | 0.045325 | 1.057175 |
| CUST_55499_PI430048170 | 2.98E-02 | 3.108456 | -2.68265 | 1.056921 |
| CUST_121032_PI430048170 | 1.19E-02 | 3.654419 | -1.47685 | 1.056898 |
| CUST_58695_PI430048170 | 3.01E-02 | 3.101301 | -2.69808 | 1.056885 |
| CUST_36020_PI430048170 | 1.50E-04 | 6.284065 | 4.415672 | 1.056285 |
| CUST_91646_PI430048170 | 3.85E-06 | 9.194296 | 10.02783 | 1.05623 |
| CUST_128823_PI430048170 | 1.22E-04 | 6.430919 | 4.72725 | 1.056169 |
| CUST_4950_PI430048170 | 4.72E-04 | 5.554749 | 2.829113 | 1.055917 |
| CUST_69067_PI430048170 | 2.38E-03 | 4.577854 | 0.625476 | 1.055118 |
| CUST_137264_PI430048170 | 7.57E-04 | 5.258877 | 2.169166 | 1.054837 |
| CUST_7867_PI430048170 | 1.02E-04 | 6.558698 | 4.996044 | 1.054335 |
| CUST_90758_PI430048170 | 3.68E-02 | 2.980062 | -2.95757 | 1.053773 |
| CUST_144883_PI430048170 | 3.35E-03 | 4.380444 | 0.174442 | 1.053646 |
| CUST_94397_PI430048170 | 1.02E-02 | 3.742083 | -1.27926 | 1.053632 |
| CUST_100096_PI430048170 | 6.86E-04 | 5.3194 | 2.304816 | 1.053476 |
| CUST_49789_PI430048170 | 4.83E-02 | 2.807659 | -3.31971 | 1.053409 |
| CUST_121735_PI430048170 | 9.34E-04 | 5.133017 | 1.886108 | 1.052994 |
| CUST_135683_PI430048170 | 6.32E-05 | 6.907082 | 5.71763 | 1.052874 |
| CUST_65941_PI430048170 | 3.09E-03 | 4.426449 | 0.279596 | 1.052658 |
| CUST_120718_PI430048170 | 4.58E-03 | 4.201957 | -0.23346 | 1.052616 |
| CUST_76974_PI430048170 | 6.92E-03 | 3.963848 | -0.77643 | 1.05255 |
| CUST_127824_PI430048170 | 2.12E-02 | 3.310529 | -2.2424 | 1.05203 |
| CUST_36500_PI430048170 | 2.01E-04 | 6.076798 | 3.971216 | 1.051496 |
| CUST_137314_PI430048170 | 6.38E-03 | 4.011729 | -0.66744 | 1.051492 |
| CUST_126498_PI430048170 | 3.40E-02 | 3.029096 | -2.85306 | 1.051246 |
| CUST_104769_PI430048170 | 1.13E-04 | 6.483877 | 4.838916 | 1.051122 |
| CUST_59663_PI430048170 | 1.57E-03 | 4.825699 | 1.190241 | 1.051019 |
| CUST_61022_PI430048170 | 9.80E-03 | 3.765202 | -1.22703 | 1.050855 |
| CUST_10784_PI430048170 | 3.73E-02 | 2.971728 | -2.97527 | 1.05052 |
| CUST_55469_PI430048170 | 5.84E-04 | 5.419178 | 2.527736 | 1.050485 |
| CUST_94133_PI430048170 | 5.65E-04 | 5.44227 | 2.579195 | 1.050456 |
| CUST_95817_PI430048170 | 1.15E-03 | 5.008706 | 1.60536 | 1.050276 |
| CUST_136170_PI430048170 | 1.82E-04 | 6.154314 | 4.138069 | 1.050005 |
| CUST_49931_PI430048170 | 9.50E-03 | 3.783773 | -1.18504 | 1.04995 |
| CUST_78813_PI430048170 | 1.38E-03 | 4.8976 | 1.353566 | 1.049792 |
| CUST_23940_PI430048170 | 3.77E-05 | 7.302702 | 6.516498 | 1.049545 |
| CUST_19677_PI430048170 | 1.20E-02 | 3.646659 | -1.49431 | 1.04919 |
| CUST_132472_PI430048170 | 3.49E-02 | 3.012403 | -2.88871 | 1.048557 |
| CUST_140092_PI430048170 | 3.09E-03 | 4.424999 | 0.276281 | 1.0483 |
| CUST_134246_PI430048170 | 1.08E-02 | 3.706471 | -1.35963 | 1.048092 |
| CUST_58051_PI430048170 | 8.34E-04 | 5.20018 | 2.037316 | 1.047749 |
| Human-PGK1_5 | 1.05E-04 | 6.53774 | 4.952105 | 1.047589 |
| CUST_42150_PI430048170 | 8.62E-03 | 3.840382 | -1.05684 | 1.047563 |
| CUST_6298_PI430048170 | 1.12E-04 | 6.489396 | 4.850531 | 1.047478 |
| CUST_126078_PI430048170 | 1.57E-02 | 3.487237 | -1.85118 | 1.047463 |
| CUST_126471_PI430048170 | 1.67E-03 | 4.788981 | 1.106732 | 1.046456 |
| CUST_62527_PI430048170 | 3.13E-03 | 4.418723 | 0.261937 | 1.046366 |
| CUST_115928_PI430048170 | 4.02E-04 | 5.650669 | 3.041225 | 1.046364 |
| CUST_127622_PI430048170 | 9.79E-04 | 5.105469 | 1.823989 | 1.046259 |
| CUST_29454_PI430048170 | 2.30E-02 | 3.262952 | -2.34681 | 1.045362 |
| CUST_115020_PI430048170 | 1.73E-04 | 6.19132 | 4.217462 | 1.045362 |
| CUST_24800_PI430048170 | 2.28E-02 | 3.26822 | -2.33527 | 1.045177 |
| CUST_137918_PI430048170 | 2.20E-03 | 4.622451 | 0.72726 | 1.044966 |
| CUST_107315_PI430048170 | 4.09E-03 | 4.26639 | -0.08625 | 1.044858 |
| CUST_39330_PI430048170 | 1.03E-02 | 3.736276 | -1.29238 | 1.044647 |
| CUST_114659_PI430048170 | 1.71E-04 | 6.198642 | 4.233148 | 1.044568 |
| CUST_78208_PI430048170 | 1.77E-03 | 4.753737 | 1.026516 | 1.044564 |
| CUST_36281_PI430048170 | 9.09E-04 | 5.149275 | 1.922742 | 1.044541 |
| CUST_70371_PI430048170 | 3.30E-03 | 4.389122 | 0.194279 | 1.04441 |
| CUST_132326_PI430048170 | 5.44E-03 | 4.101238 | -0.4634 | 1.044384 |
| CUST_17959_PI430048170 | 1.97E-03 | 4.690358 | 0.882129 | 1.044295 |
| CUST_63323_PI430048170 | 1.95E-04 | 6.097777 | 4.016446 | 1.044159 |
| CUST_69195_PI430048170 | 6.69E-03 | 3.983943 | -0.73071 | 1.044025 |
| CUST_104914_PI430048170 | 2.19E-03 | 4.627589 | 0.738983 | 1.04379 |
| CUST_37688_PI430048170 | 1.86E-03 | 4.724874 | 0.96078 | 1.043752 |
| CUST_137693_PI430048170 | 2.90E-04 | 5.853231 | 3.485904 | 1.043721 |
| CUST_120664_PI430048170 | 4.33E-03 | 4.233804 | -0.16071 | 1.043675 |
| CUST_84038_PI430048170 | 2.48E-02 | 3.215695 | -2.45007 | 1.042977 |
| CUST_108397_PI430048170 | 2.25E-02 | 3.276425 | -2.31728 | 1.042892 |
| CUST_111897_PI430048170 | 2.89E-03 | 4.465403 | 0.368618 | 1.042859 |
| CUST_75218_PI430048170 | 2.99E-02 | 3.107425 | -2.68487 | 1.042089 |
| CUST_78436_PI430048170 | 3.75E-04 | 5.695608 | 3.140267 | 1.042075 |
| CUST_59884_PI430048170 | 6.73E-04 | 5.332263 | 2.333604 | 1.041895 |
| CUST_29636_PI430048170 | 2.33E-02 | 3.255356 | -2.36343 | 1.041814 |
| CUST_138923_PI430048170 | 5.95E-04 | 5.407937 | 2.502668 | 1.04168 |
| CUST_132553_PI430048170 | 3.92E-04 | 5.667244 | 3.077781 | 1.041656 |
| CUST_136068_PI430048170 | 2.43E-03 | 4.565487 | 0.59724 | 1.041197 |
| CUST_81021_PI430048170 | 2.62E-02 | 3.183291 | -2.52061 | 1.040683 |
| CUST_24536_PI430048170 | 5.70E-03 | 4.075989 | -0.52099 | 1.04059 |
| CUST_81005_PI430048170 | 4.93E-02 | 2.796001 | -3.34388 | 1.040144 |
| CUST_70496_PI430048170 | 1.03E-02 | 3.733173 | -1.29938 | 1.039905 |
| CUST_142474_PI430048170 | 1.47E-05 | 8.04928 | 7.963116 | 1.039503 |
| CUST_73510_PI430048170 | 3.34E-05 | 7.413223 | 6.735693 | 1.039417 |
| CUST_115336_PI430048170 | 3.11E-02 | 3.082461 | -2.73864 | 1.039151 |
| CUST_11310_PI430048170 | 3.85E-03 | 4.300427 | -0.00846 | 1.038954 |
| CUST_57633_PI430048170 | 2.49E-02 | 3.212833 | -2.45631 | 1.038907 |
| CUST_30083_PI430048170 | 3.34E-03 | 4.383092 | 0.180494 | 1.038692 |
| CUST_5116_PI430048170 | 8.24E-05 | 6.716421 | 5.324791 | 1.038498 |
| CUST_56897_PI430048170 | 8.30E-04 | 5.202887 | 2.043402 | 1.038152 |
| CUST_51410_PI430048170 | 6.21E-03 | 4.027704 | -0.63105 | 1.038102 |
| CUST_105658_PI430048170 | 7.71E-04 | 5.248455 | 2.145777 | 1.037838 |
| CUST_131563_PI430048170 | 1.24E-04 | 6.416864 | 4.697552 | 1.037817 |
| CUST_137250_PI430048170 | 7.08E-03 | 3.951039 | -0.80557 | 1.037583 |
| CUST_59414_PI430048170 | 2.05E-03 | 4.665741 | 0.826005 | 1.03752 |
| CUST_143291_PI430048170 | 3.67E-03 | 4.329142 | 0.057175 | 1.037484 |
| CUST_32931_PI430048170 | 7.40E-04 | 5.273525 | 2.202027 | 1.037281 |
| CUST_101071_PI430048170 | 2.12E-05 | 7.76612 | 7.423868 | 1.036713 |
| CUST_140089_PI430048170 | 2.56E-02 | 3.197643 | -2.48939 | 1.036598 |
| CUST_34880_PI430048170 | 7.37E-03 | 3.92798 | -0.858 | 1.036284 |
| CUST_118794_PI430048170 | 4.59E-03 | 4.200554 | -0.23667 | 1.036132 |
| CUST_5073_PI430048170 | 2.09E-03 | 4.652749 | 0.796378 | 1.03584 |
| CUST_134411_PI430048170 | 3.72E-02 | 2.973486 | -2.97153 | 1.035515 |
| CUST_80156_PI430048170 | 1.85E-04 | 6.140847 | 4.109135 | 1.035174 |
| CUST_122060_PI430048170 | 1.31E-04 | 6.377647 | 4.61455 | 1.035088 |
| CUST_65848_PI430048170 | 1.38E-02 | 3.564287 | -1.67911 | 1.035066 |
| CUST_57829_PI430048170 | 9.51E-05 | 6.609877 | 5.103088 | 1.034941 |
| CUST_45907_PI430048170 | 3.01E-02 | 3.102741 | -2.69497 | 1.034909 |
| CUST_5134_PI430048170 | 4.05E-03 | 4.272959 | -0.07124 | 1.034492 |
| CUST_24144_PI430048170 | 3.05E-03 | 4.43295 | 0.294454 | 1.033719 |
| CUST_120441_PI430048170 | 2.92E-03 | 4.458813 | 0.353559 | 1.032744 |
| CUST_109642_PI430048170 | 3.29E-03 | 4.392219 | 0.201358 | 1.03265 |
| CUST_127540_PI430048170 | 1.99E-02 | 3.348038 | -2.1598 | 1.032646 |
| CUST_102449_PI430048170 | 3.53E-05 | 7.360362 | 6.631073 | 1.032581 |
| CUST_100360_PI430048170 | 3.15E-02 | 3.074804 | -2.7551 | 1.031916 |
| CUST_137275_PI430048170 | 1.61E-05 | 7.969169 | 7.81172 | 1.031766 |
| CUST_3733_PI430048170 | 1.12E-02 | 3.68596 | -1.40586 | 1.03163 |
| CUST_97666_PI430048170 | 6.04E-05 | 6.952853 | 5.811184 | 1.031047 |
| CUST_112447_PI430048170 | 3.02E-04 | 5.828758 | 3.432422 | 1.030482 |
| CUST_102855_PI430048170 | 2.73E-02 | 3.160716 | -2.56962 | 1.030415 |
| CUST_62021_PI430048170 | 4.01E-04 | 5.65264 | 3.045574 | 1.030086 |
| CUST_34275_PI430048170 | 1.64E-02 | 3.463233 | -1.90462 | 1.030079 |
| CUST_101290_PI430048170 | 3.10E-02 | 3.084622 | -2.73399 | 1.02929 |
| CUST_91732_PI430048170 | 1.39E-03 | 4.896276 | 1.350562 | 1.028998 |
| CUST_79232_PI430048170 | 1.77E-02 | 3.419836 | -2.001 | 1.02868 |
| CUST_106746_PI430048170 | 6.36E-05 | 6.90269 | 5.708637 | 1.028674 |
| CUST_97004_PI430048170 | 4.21E-03 | 4.250946 | -0.12154 | 1.028584 |
| CUST_124687_PI430048170 | 4.04E-04 | 5.646338 | 3.031669 | 1.028152 |
| CUST_87196_PI430048170 | 2.97E-02 | 3.111063 | -2.67703 | 1.028063 |
| CUST_95596_PI430048170 | 7.22E-03 | 3.94005 | -0.83056 | 1.028021 |
| CUST_89657_PI430048170 | 1.13E-03 | 5.018492 | 1.627501 | 1.027911 |
| CUST_69643_PI430048170 | 1.09E-03 | 5.041459 | 1.679436 | 1.027878 |
| CUST_91066_PI430048170 | 2.70E-03 | 4.504939 | 0.458952 | 1.027773 |
| CUST_101404_PI430048170 | 1.36E-03 | 4.909468 | 1.380498 | 1.027706 |
| CUST_134593_PI430048170 | 5.29E-05 | 7.048553 | 6.005842 | 1.027691 |
| CUST_95452_PI430048170 | 2.18E-06 | 9.71529 | 10.90768 | 1.027654 |
| CUST_94266_PI430048170 | 2.30E-02 | 3.261187 | -2.35067 | 1.027328 |
| CUST_122857_PI430048170 | 2.46E-05 | 7.644132 | 7.188005 | 1.027184 |
| CUST_112257_PI430048170 | 5.05E-05 | 7.085486 | 6.080619 | 1.026466 |
| CUST_47104_PI430048170 | 4.46E-03 | 4.215936 | -0.20153 | 1.026264 |
| CUST_63203_PI430048170 | 2.48E-03 | 4.554162 | 0.57138 | 1.026221 |
| CUST_83015_PI430048170 | 8.01E-04 | 5.226003 | 2.095357 | 1.026123 |
| CUST_68919_PI430048170 | 1.46E-04 | 6.303152 | 4.456328 | 1.025956 |
| CUST_70102_PI430048170 | 1.58E-02 | 3.485968 | -1.85401 | 1.024834 |
| CUST_131554_PI430048170 | 5.57E-04 | 5.452028 | 2.600925 | 1.024815 |
| CUST_54869_PI430048170 | 1.66E-02 | 3.455691 | -1.92139 | 1.024664 |
| CUST_143437_PI430048170 | 4.35E-02 | 2.874718 | -3.17988 | 1.024583 |
| CUST_72483_PI430048170 | 3.13E-03 | 4.41831 | 0.260993 | 1.024479 |
| CUST_142668_PI430048170 | 1.26E-04 | 6.40254 | 4.66726 | 1.024459 |
| CUST_129034_PI430048170 | 9.15E-03 | 3.806261 | -1.13414 | 1.024315 |
| CUST_91100_PI430048170 | 2.53E-02 | 3.204222 | -2.47507 | 1.023439 |
| CUST_129595_PI430048170 | 1.14E-03 | 5.01252 | 1.613989 | 1.023182 |
| CUST_71725_PI430048170 | 4.67E-04 | 5.561624 | 2.844346 | 1.023019 |
| CUST_60141_PI430048170 | 9.86E-03 | 3.76155 | -1.23529 | 1.022978 |
| CUST_31162_PI430048170 | 6.62E-05 | 6.870724 | 5.643108 | 1.022803 |
| CUST_39435_PI430048170 | 5.09E-03 | 4.140737 | -0.37326 | 1.022718 |
| CUST_110739_PI430048170 | 6.92E-04 | 5.313815 | 2.292312 | 1.02246 |
| CUST_5214_PI430048170 | 2.20E-03 | 4.625001 | 0.733079 | 1.022442 |
| CUST_34_PI430048170 | 1.37E-03 | 4.905734 | 1.372025 | 1.02243 |
| CUST_105129_PI430048170 | 2.30E-03 | 4.599187 | 0.674171 | 1.022254 |
| CUST_119079_PI430048170 | 3.85E-03 | 4.301149 | -0.00681 | 1.021511 |
| CUST_57378_PI430048170 | 2.31E-02 | 3.258914 | -2.35565 | 1.021146 |
| CUST_132745_PI430048170 | 5.64E-04 | 5.443652 | 2.582273 | 1.021138 |
| CUST_86267_PI430048170 | 1.60E-03 | 4.815772 | 1.167671 | 1.020647 |
| CUST_145635_PI430048170 | 3.43E-03 | 4.368121 | 0.146274 | 1.020556 |
| CUST_124074_PI430048170 | 2.04E-05 | 7.801251 | 7.491396 | 1.020286 |
| CUST_95071_PI430048170 | 1.40E-02 | 3.55468 | -1.70061 | 1.020096 |
| CUST_112108_PI430048170 | 1.94E-02 | 3.361483 | -2.13013 | 1.019809 |
| CUST_53829_PI430048170 | 6.94E-03 | 3.961889 | -0.78089 | 1.019183 |
| CUST_125886_PI430048170 | 6.67E-04 | 5.337637 | 2.345627 | 1.019154 |
| CUST_46953_PI430048170 | 1.39E-02 | 3.560894 | -1.6867 | 1.018552 |
| CUST_49880_PI430048170 | 2.34E-03 | 4.588332 | 0.649394 | 1.018309 |
| CUST_94853_PI430048170 | 3.64E-03 | 4.332808 | 0.065554 | 1.018033 |
| CUST_100277_PI430048170 | 2.64E-03 | 4.517737 | 0.488188 | 1.017523 |
| CUST_32012_PI430048170 | 1.21E-02 | 3.639545 | -1.5103 | 1.016824 |
| CUST_135441_PI430048170 | 1.05E-03 | 5.060702 | 1.722923 | 1.016776 |
| CUST_35675_PI430048170 | 1.68E-03 | 4.783322 | 1.093856 | 1.016635 |
| CUST_123812_PI430048170 | 1.40E-02 | 3.558091 | -1.69298 | 1.01639 |
| CUST_144791_PI430048170 | 4.09E-02 | 2.915457 | -3.09427 | 1.016303 |
| CUST_83645_PI430048170 | 1.55E-05 | 8.00129 | 7.872534 | 1.016152 |
| CUST_109272_PI430048170 | 5.78E-03 | 4.067735 | -0.53981 | 1.014926 |
| CUST_70183_PI430048170 | 2.21E-03 | 4.62058 | 0.722992 | 1.014831 |
| CUST_57913_PI430048170 | 4.90E-04 | 5.529713 | 2.773594 | 1.014809 |
| CUST_11759_PI430048170 | 4.04E-04 | 5.64644 | 3.031894 | 1.014701 |
| CUST_4783_PI430048170 | 3.31E-03 | 4.387706 | 0.191042 | 1.01447 |
| CUST_87742_PI430048170 | 6.60E-03 | 3.991625 | -0.71322 | 1.014218 |
| CUST_106342_PI430048170 | 2.99E-02 | 3.10704 | -2.6857 | 1.01393 |
| CUST_12832_PI430048170 | 1.18E-02 | 3.655924 | -1.47347 | 1.013844 |
| CUST_20703_PI430048170 | 1.37E-02 | 3.569687 | -1.66702 | 1.013693 |
| CUST_127942_PI430048170 | 1.86E-04 | 6.135056 | 4.096686 | 1.013226 |
| CUST_131771_PI430048170 | 1.48E-02 | 3.522717 | -1.77205 | 1.013107 |
| CUST_109546_PI430048170 | 9.07E-03 | 3.811282 | -1.12277 | 1.012966 |
| CUST_71514_PI430048170 | 2.00E-04 | 6.079806 | 3.977703 | 1.012855 |
| CUST_7668_PI430048170 | 6.31E-05 | 6.908787 | 5.721121 | 1.012676 |
| CUST_58175_PI430048170 | 8.48E-03 | 3.848626 | -1.03815 | 1.012584 |
| CUST_50844_PI430048170 | 4.35E-02 | 2.875019 | -3.17925 | 1.012444 |
| CUST_141133_PI430048170 | 3.56E-02 | 2.999767 | -2.91565 | 1.012436 |
| CUST_123502_PI430048170 | 6.49E-05 | 6.887025 | 5.676542 | 1.012282 |
| CUST_132112_PI430048170 | 1.10E-02 | 3.699528 | -1.37528 | 1.011611 |
| CUST_24812_PI430048170 | 1.26E-02 | 3.617447 | -1.55994 | 1.011445 |
| CUST_121910_PI430048170 | 7.09E-05 | 6.822534 | 5.544048 | 1.011205 |
| CUST_111092_PI430048170 | 4.47E-03 | 4.21536 | -0.20285 | 1.011058 |
| CUST_72002_PI430048170 | 2.85E-03 | 4.472658 | 0.385198 | 1.010836 |
| CUST_16289_PI430048170 | 4.19E-02 | 2.899991 | -3.12683 | 1.010604 |
| CUST_130299_PI430048170 | 1.84E-03 | 4.731156 | 0.975091 | 1.010499 |
| CUST_117032_PI430048170 | 8.19E-04 | 5.211532 | 2.062838 | 1.010485 |
| CUST_136450_PI430048170 | 6.71E-05 | 6.860256 | 5.621617 | 1.010187 |
| CUST_60370_PI430048170 | 1.14E-04 | 6.477567 | 4.825631 | 1.009953 |
| CUST_31006_PI430048170 | 2.32E-02 | 3.258198 | -2.35721 | 1.009887 |
| CUST_92431_PI430048170 | 1.08E-02 | 3.710054 | -1.35155 | 1.009667 |
| CUST_117888_PI430048170 | 6.18E-04 | 5.384792 | 2.451014 | 1.008288 |
| CUST_140415_PI430048170 | 3.74E-02 | 2.969947 | -2.97905 | 1.008201 |
| CUST_60455_PI430048170 | 4.26E-02 | 2.888211 | -3.15158 | 1.007976 |
| CUST_103172_PI430048170 | 4.81E-04 | 5.540766 | 2.798112 | 1.007911 |
| CUST_77577_PI430048170 | 1.36E-03 | 4.909804 | 1.38126 | 1.007895 |
| CUST_97169_PI430048170 | 4.47E-02 | 2.857795 | -3.21529 | 1.007142 |
| CUST_74368_PI430048170 | 2.62E-02 | 3.182572 | -2.52217 | 1.00705 |
| CUST_80628_PI430048170 | 4.20E-04 | 5.623799 | 2.981902 | 1.006602 |
| CUST_130742_PI430048170 | 9.23E-03 | 3.801215 | -1.14556 | 1.006481 |
| CUST_13881_PI430048170 | 1.67E-02 | 3.452366 | -1.92878 | 1.006414 |
| CUST_136367_PI430048170 | 4.35E-05 | 7.201431 | 6.314115 | 1.006407 |
| CUST_127829_PI430048170 | 1.12E-05 | 8.256088 | 8.349706 | 1.006392 |
| CUST_73173_PI430048170 | 1.08E-02 | 3.70988 | -1.35194 | 1.006241 |
| CUST_33564_PI430048170 | 9.76E-03 | 3.767573 | -1.22167 | 1.00624 |
| CUST_132525_PI430048170 | 1.68E-04 | 6.207442 | 4.251995 | 1.006235 |
| CUST_60125_PI430048170 | 2.97E-02 | 3.110458 | -2.67833 | 1.005829 |
| CUST_78066_PI430048170 | 4.44E-05 | 7.186804 | 6.284765 | 1.005125 |
| CUST_17031_PI430048170 | 8.97E-03 | 3.817683 | -1.10828 | 1.004831 |
| CUST_121879_PI430048170 | 1.13E-02 | 3.680831 | -1.41741 | 1.004424 |
| CUST_109742_PI430048170 | 1.03E-03 | 5.07071 | 1.74553 | 1.004055 |
| CUST_137788_PI430048170 | 3.35E-03 | 4.380698 | 0.175023 | 1.004026 |
| CUST_132990_PI430048170 | 6.62E-04 | 5.342365 | 2.356204 | 1.003901 |
| CUST_59587_PI430048170 | 9.44E-05 | 6.615087 | 5.113966 | 1.003505 |
| CUST_136773_PI430048170 | 1.10E-03 | 5.034857 | 1.66451 | 1.003304 |
| CUST_104209_PI430048170 | 5.01E-05 | 7.09088 | 6.091523 | 1.003156 |
| CUST_11297_PI430048170 | 1.48E-02 | 3.52444 | -1.7682 | 1.00276 |
| CUST_123542_PI430048170 | 1.74E-02 | 3.42846 | -1.98187 | 1.002135 |
| CUST_52034_PI430048170 | 2.19E-02 | 3.291636 | -2.28391 | 1.00181 |
| CUST_142027_PI430048170 | 9.81E-04 | 5.103845 | 1.820326 | 1.001623 |
| CUST_94690_PI430048170 | 3.04E-03 | 4.435454 | 0.300176 | 1.001559 |
| CUST_17014_PI430048170 | 1.18E-02 | 3.657357 | -1.47025 | 1.001465 |
| CUST_88988_PI430048170 | 2.38E-05 | 7.676571 | 7.250935 | 1.001317 |
| CUST_123342_PI430048170 | 3.27E-03 | 4.395492 | 0.208838 | 1.000894 |
| CUST_128338_PI430048170 | 3.48E-04 | 5.745523 | 3.250022 | 1.000732 |
| CUST_90324_PI430048170 | 5.59E-04 | 5.449026 | 2.594241 | 1.000043 |
| CUST_142831_PI430048170 | 3.92E-05 | -7.27863 | 6.468529 | -6.80553 |
| CUST_135819_PI430048170 | 7.44E-06 | -8.64467 | 9.059667 | -6.41732 |
| CUST_130000_PI430048170 | 7.27E-07 | -10.6768 | 12.43915 | -6.29696 |
| CUST_69980_PI430048170 | 1.74E-06 | -9.88734 | 11.19037 | -6.29083 |
| CUST_141915_PI430048170 | 4.69E-07 | -11.1093 | 13.09106 | -6.23829 |
| CUST_138708_PI430048170 | 2.44E-06 | -9.60078 | 10.71739 | -6.21982 |
| CUST_132744_PI430048170 | 3.86E-07 | -11.3404 | 13.43038 | -6.20868 |
| CUST_106947_PI430048170 | 3.43E-06 | -9.29526 | 10.20117 | -6.2045 |
| CUST_21824_PI430048170 | 1.50E-06 | -9.98919 | 11.3559 | -6.12849 |
| CUST_127509_PI430048170 | 8.24E-09 | -15.5492 | 18.66474 | -6.11604 |
| CUST_14871_PI430048170 | 4.99E-06 | -8.96746 | 9.633303 | -6.08604 |
| CUST_128809_PI430048170 | 2.23E-06 | -9.68985 | 10.86555 | -5.96326 |
| CUST_133607_PI430048170 | 5.74E-08 | -13.1425 | 15.87708 | -5.85642 |
| CUST_136524_PI430048170 | 6.28E-05 | -6.91343 | 5.730616 | -5.842 |
| CUST_137943_PI430048170 | 5.97E-07 | -10.8854 | 12.75631 | -5.78338 |
| CUST_66802_PI430048170 | 1.72E-07 | -12.1041 | 14.50897 | -5.75781 |
| CUST_16758_PI430048170 | 1.77E-05 | -7.89831 | 7.67704 | -5.63752 |
| CUST_132566_PI430048170 | 2.56E-05 | -7.60527 | 7.112419 | -5.56166 |
| CUST_32499_PI430048170 | 2.87E-06 | -9.47527 | 10.50684 | -5.55839 |
| CUST_128020_PI430048170 | 5.75E-04 | -5.42991 | 2.551649 | -5.53498 |
| CUST_132048_PI430048170 | 3.14E-05 | -7.45769 | 6.823385 | -5.49063 |
| CUST_75411_PI430048170 | 4.02E-07 | -11.274 | 13.33352 | -5.47519 |
| CUST_145585_PI430048170 | 8.62E-05 | -6.68424 | 5.257986 | -5.45338 |
| CUST_135275_PI430048170 | 1.67E-09 | -18.0592 | 21.0983 | -5.44644 |
| CUST_136046_PI430048170 | 3.04E-06 | -9.41862 | 10.41111 | -5.40121 |
| CUST_137336_PI430048170 | 5.56E-07 | -10.9515 | 12.85572 | -5.39382 |
| CUST_130802_PI430048170 | 5.93E-08 | -13.0956 | 15.81754 | -5.36611 |
| CUST_73380_PI430048170 | 5.70E-09 | -16.1678 | 19.30532 | -5.355 |
| CUST_107691_PI430048170 | 4.59E-07 | -11.1322 | 13.12489 | -5.34525 |
| CUST_145298_PI430048170 | 1.91E-07 | -11.9869 | 14.34762 | -5.32287 |
| CUST_141226_PI430048170 | 1.19E-05 | -8.20234 | 8.249821 | -5.2546 |
| CUST_104824_PI430048170 | 1.28E-04 | -6.39446 | 4.650155 | -5.24059 |
| CUST_129162_PI430048170 | 2.21E-08 | -14.5289 | 17.54337 | -5.23651 |
| CUST_128303_PI430048170 | 4.58E-07 | -11.1628 | 13.17013 | -5.22846 |
| CUST_112218_PI430048170 | 2.87E-06 | -9.46635 | 10.49179 | -5.22559 |
| CUST_144806_PI430048170 | 1.74E-05 | -7.91557 | 7.709906 | -5.21321 |
| CUST_144000_PI430048170 | 3.66E-07 | -11.3978 | 13.51369 | -5.15765 |
| CUST_139590_PI430048170 | 2.26E-08 | -14.452 | 17.45544 | -5.15294 |
| CUST_105903_PI430048170 | 1.78E-06 | -9.8634 | 11.15126 | -5.11976 |
| CUST_115024_PI430048170 | 3.65E-04 | -5.71277 | 3.178035 | -5.10687 |
| CUST_142247_PI430048170 | 1.09E-05 | -8.27941 | 8.392914 | -5.10558 |
| CUST_81979_PI430048170 | 1.85E-05 | -7.86603 | 7.615447 | -5.10102 |
| CUST_134239_PI430048170 | 7.22E-03 | -3.93976 | -0.83122 | -5.10056 |
| CUST_107439_PI430048170 | 2.50E-06 | -9.58322 | 10.68807 | -5.09893 |
| CUST_139910_PI430048170 | 3.10E-06 | -9.39616 | 10.37305 | -5.09068 |
| CUST_40750_PI430048170 | 3.40E-04 | -5.76046 | 3.282819 | -5.07227 |
| CUST_136679_PI430048170 | 2.22E-05 | -7.73576 | 7.365365 | -5.06103 |
| CUST_94052_PI430048170 | 3.67E-08 | -13.6482 | 16.50506 | -5.01138 |
| CUST_16770_PI430048170 | 1.10E-05 | -8.27048 | 8.376381 | -4.99727 |
| CUST_14868_PI430048170 | 5.15E-05 | -7.06919 | 6.047647 | -4.97863 |
| CUST_135722_PI430048170 | 5.22E-05 | -7.0585 | 6.026005 | -4.97279 |
| CUST_134376_PI430048170 | 8.14E-06 | -8.55775 | 8.902712 | -4.97002 |
| CUST_144613_PI430048170 | 9.03E-06 | -8.43306 | 8.675683 | -4.95043 |
| CUST_28889_PI430048170 | 5.15E-05 | -7.06855 | 6.046348 | -4.93739 |
| CUST_139601_PI430048170 | 8.50E-05 | -6.69415 | 5.278575 | -4.92787 |
| CUST_71338_PI430048170 | 4.28E-07 | -11.2231 | 13.25887 | -4.92598 |
| CUST_142712_PI430048170 | 1.32E-06 | -10.1166 | 11.56112 | -4.91772 |
| CUST_136012_PI430048170 | 8.75E-04 | -5.17183 | 1.973536 | -4.90517 |
| CUST_103814_PI430048170 | 4.86E-05 | -7.11445 | 6.139136 | -4.90109 |
| CUST_117836_PI430048170 | 5.16E-06 | -8.9387 | 9.582776 | -4.88402 |
| CUST_126788_PI430048170 | 4.77E-06 | -9.0088 | 9.705728 | -4.87832 |
| CUST_28555_PI430048170 | 3.73E-05 | -7.31332 | 6.537632 | -4.8697 |
| CUST_140270_PI430048170 | 7.81E-06 | -8.60304 | 8.984614 | -4.86292 |
| CUST_105841_PI430048170 | 2.51E-06 | -9.57849 | 10.68015 | -4.85654 |
| CUST_126724_PI430048170 | 4.69E-08 | -13.3654 | 16.15686 | -4.84875 |
| CUST_14869_PI430048170 | 2.71E-05 | -7.56143 | 7.026881 | -4.84827 |
| CUST_138527_PI430048170 | 6.51E-05 | -6.88173 | 5.66568 | -4.83772 |
| CUST_137402_PI430048170 | 8.51E-06 | -8.51519 | 8.825467 | -4.80778 |
| CUST_137509_PI430048170 | 6.32E-08 | -13.0155 | 15.71555 | -4.79533 |
| CUST_9352_PI430048170 | 8.31E-06 | -8.53124 | 8.854614 | -4.777 |
| CUST_145196_PI430048170 | 2.75E-07 | -11.6402 | 13.86153 | -4.77356 |
| CUST_73748_PI430048170 | 7.27E-07 | -10.6569 | 12.40862 | -4.73022 |
| CUST_19150_PI430048170 | 1.65E-04 | -6.22119 | 4.281408 | -4.69079 |
| CUST_139591_PI430048170 | 4.73E-09 | -16.7972 | 19.92865 | -4.66278 |
| CUST_145111_PI430048170 | 2.86E-02 | -3.13143 | -2.63303 | -4.64547 |
| CUST_136022_PI430048170 | 4.21E-05 | -7.22775 | 6.366862 | -4.6444 |
| CUST_112217_PI430048170 | 1.81E-05 | -7.88305 | 7.647949 | -4.61421 |
| CUST_136055_PI430048170 | 4.66E-05 | -7.14797 | 6.206684 | -4.61285 |
| CUST_69937_PI430048170 | 8.69E-06 | -8.49218 | 8.7836 | -4.60583 |
| CUST_142015_PI430048170 | 4.88E-05 | -7.11146 | 6.133091 | -4.6053 |
| CUST_93174_PI430048170 | 6.39E-05 | -6.89864 | 5.700335 | -4.6027 |
| CUST_8776_PI430048170 | 2.06E-05 | -7.79201 | 7.473641 | -4.59619 |
| CUST_139175_PI430048170 | 7.27E-07 | -10.6715 | 12.43107 | -4.58585 |
| CUST_135577_PI430048170 | 2.48E-05 | -7.63614 | 7.172487 | -4.56032 |
| CUST_25171_PI430048170 | 3.61E-04 | -5.7238 | 3.202296 | -4.5551 |
| CUST_6475_PI430048170 | 9.75E-05 | -6.59021 | 5.062001 | -4.54033 |
| CUST_138690_PI430048170 | 3.64E-05 | -7.32905 | 6.568914 | -4.53833 |
| CUST_21820_PI430048170 | 3.50E-06 | -9.27677 | 10.16953 | -4.48495 |
| CUST_133889_PI430048170 | 4.96E-05 | -7.10021 | 6.110369 | -4.48404 |
| CUST_141894_PI430048170 | 3.53E-05 | -7.35892 | 6.628204 | -4.48136 |
| CUST_73386_PI430048170 | 2.90E-08 | -14.0449 | 16.98121 | -4.46025 |
| CUST_95438_PI430048170 | 3.00E-05 | -7.49452 | 6.89581 | -4.45391 |
| CUST_113494_PI430048170 | 1.78E-06 | -9.86836 | 11.15936 | -4.43642 |
| CUST_128068_PI430048170 | 4.52E-05 | -7.16679 | 6.244562 | -4.42538 |
| CUST_136252_PI430048170 | 2.16E-05 | -7.75396 | 7.400461 | -4.40243 |
| CUST_133286_PI430048170 | 1.87E-07 | -12.0301 | 14.40725 | -4.36265 |
| CUST_144757_PI430048170 | 1.53E-07 | -12.2497 | 14.7075 | -4.34578 |
| CUST_120498_PI430048170 | 1.45E-04 | -6.30695 | 4.464414 | -4.33895 |
| CUST_22816_PI430048170 | 3.86E-07 | -11.3294 | 13.41436 | -4.33158 |
| CUST_131935_PI430048170 | 8.34E-05 | -6.70808 | 5.307496 | -4.3157 |
| CUST_28890_PI430048170 | 1.81E-04 | -6.16041 | 4.151167 | -4.29581 |
| CUST_38420_PI430048170 | 3.44E-04 | -5.75268 | 3.265739 | -4.27412 |
| CUST_134734_PI430048170 | 1.08E-03 | -5.04511 | 1.687699 | -4.2568 |
| CUST_118948_PI430048170 | 1.86E-03 | -4.72581 | 0.962905 | -4.24672 |
| CUST_118329_PI430048170 | 2.32E-06 | -9.65621 | 10.80972 | -4.24431 |
| CUST_138391_PI430048170 | 5.90E-06 | -8.81963 | 9.372392 | -4.21374 |
| CUST_142573_PI430048170 | 3.95E-06 | -9.15665 | 9.962839 | -4.19553 |
| CUST_50416_PI430048170 | 6.38E-07 | -10.8263 | 12.66705 | -4.18487 |
| CUST_135352_PI430048170 | 1.50E-05 | -8.03338 | 7.933132 | -4.18186 |
| CUST_84037_PI430048170 | 9.55E-04 | -5.1197 | 1.856093 | -4.18184 |
| CUST_130540_PI430048170 | 9.98E-07 | -10.3817 | 11.98145 | -4.15346 |
| CUST_138206_PI430048170 | 1.05E-05 | -8.30127 | 8.433357 | -4.12839 |
| CUST_131239_PI430048170 | 3.26E-06 | -9.33865 | 10.27526 | -4.1185 |
| CUST_127595_PI430048170 | 1.30E-04 | -6.38097 | 4.621596 | -4.11439 |
| CUST_115612_PI430048170 | 2.57E-03 | -4.53247 | 0.521849 | -4.11307 |
| CUST_77362_PI430048170 | 1.15E-04 | -6.46751 | 4.804439 | -4.10978 |
| CUST_88333_PI430048170 | 1.86E-04 | -6.1362 | 4.099153 | -4.10353 |
| CUST_119440_PI430048170 | 7.49E-04 | -5.26539 | 2.183777 | -4.09905 |
| CUST_121942_PI430048170 | 3.50E-04 | -5.74318 | 3.244864 | -4.09711 |
| CUST_49692_PI430048170 | 8.22E-05 | -6.71897 | 5.33007 | -4.09376 |
| CUST_145481_PI430048170 | 1.51E-07 | -12.2764 | 14.74363 | -4.07462 |
| CUST_56253_PI430048170 | 8.18E-06 | -8.55022 | 8.889064 | -4.07334 |
| CUST_121726_PI430048170 | 1.19E-02 | -3.65006 | -1.48666 | -4.07327 |
| CUST_47956_PI430048170 | 6.80E-03 | -3.97428 | -0.75269 | -4.05511 |
| CUST_94767_PI430048170 | 4.06E-06 | -9.13691 | 9.92869 | -4.05105 |
| CUST_127298_PI430048170 | 1.44E-04 | -6.31124 | 4.473533 | -4.0487 |
| CUST_134337_PI430048170 | 6.44E-06 | -8.75605 | 9.259236 | -4.04783 |
| CUST_748_PI430048170 | 5.68E-04 | -5.43905 | 2.572022 | -4.0385 |
| CUST_118741_PI430048170 | 9.17E-07 | -10.4462 | 12.08243 | -4.02509 |
| CUST_133099_PI430048170 | 5.81E-06 | -8.82986 | 9.390549 | -4.01099 |
| CUST_134525_PI430048170 | 1.41E-05 | -8.08179 | 8.0243 | -4.00815 |
| CUST_131218_PI430048170 | 9.87E-05 | -6.58113 | 5.043002 | -4.00648 |
| CUST_132981_PI430048170 | 2.39E-07 | -11.7865 | 14.06828 | -4.0025 |
| CUST_25474_PI430048170 | 7.13E-04 | -5.29416 | 2.248281 | -4.00132 |
| CUST_136573_PI430048170 | 8.83E-07 | -10.4924 | 12.15446 | -4.00036 |
| CUST_62483_PI430048170 | 5.53E-05 | -7.01549 | 5.938731 | -3.99258 |
| CUST_127126_PI430048170 | 8.93E-08 | -12.7164 | 15.3288 | -3.97739 |
| CUST_13556_PI430048170 | 1.58E-05 | -7.98607 | 7.843734 | -3.97522 |
| CUST_138314_PI430048170 | 3.04E-06 | -9.41694 | 10.40826 | -3.97133 |
| CUST_144776_PI430048170 | 4.33E-03 | -4.23441 | -0.15932 | -3.96766 |
| CUST_51053_PI430048170 | 3.43E-04 | -5.75556 | 3.272053 | -3.96636 |
| CUST_131504_PI430048170 | 2.56E-05 | -7.60536 | 7.112588 | -3.96355 |
| CUST_98778_PI430048170 | 1.19E-05 | -8.19814 | 8.241989 | -3.96265 |
| CUST_59297_PI430048170 | 4.91E-07 | -11.0713 | 13.03468 | -3.94555 |
| CUST_129307_PI430048170 | 5.70E-04 | -5.43664 | 2.566659 | -3.93159 |
| CUST_69260_PI430048170 | 8.85E-06 | -8.46654 | 8.736857 | -3.9301 |
| CUST_131828_PI430048170 | 1.36E-04 | -6.35237 | 4.560949 | -3.91995 |
| CUST_127420_PI430048170 | 1.32E-06 | -10.1217 | 11.56929 | -3.91657 |
| CUST_129419_PI430048170 | 2.44E-04 | -5.95901 | 3.716261 | -3.90556 |
| CUST_98692_PI430048170 | 2.64E-05 | -7.58031 | 7.063748 | -3.90253 |
| CUST_137179_PI430048170 | 1.51E-05 | -8.02063 | 7.909079 | -3.90154 |
| CUST_370_PI430048170 | 5.79E-05 | -6.98314 | 5.872924 | -3.88641 |
| CUST_139975_PI430048170 | 3.15E-05 | -7.45612 | 6.820302 | -3.8829 |
| CUST_129776_PI430048170 | 8.09E-06 | -8.57519 | 8.934289 | -3.87421 |
| CUST_130871_PI430048170 | 1.38E-05 | -8.0972 | 8.053237 | -3.86628 |
| CUST_14877_PI430048170 | 4.87E-06 | -8.9925 | 9.677209 | -3.86547 |
| CUST_145547_PI430048170 | 1.42E-06 | -10.0564 | 11.46442 | -3.85054 |
| CUST_139060_PI430048170 | 3.56E-05 | -7.35273 | 6.615934 | -3.84596 |
| CUST_131817_PI430048170 | 1.23E-04 | -6.42506 | 4.714881 | -3.84484 |
| CUST_58236_PI430048170 | 2.06E-04 | -6.06139 | 3.937961 | -3.83698 |
| CUST_126496_PI430048170 | 9.80E-05 | -6.58608 | 5.053361 | -3.7993 |
| CUST_102881_PI430048170 | 1.59E-07 | -12.1747 | 14.60552 | -3.79118 |
| CUST_21711_PI430048170 | 1.86E-03 | -4.72602 | 0.963384 | -3.78965 |
| CUST_112666_PI430048170 | 1.02E-06 | -10.3529 | 11.93624 | -3.78635 |
| CUST_125652_PI430048170 | 2.43E-03 | -4.56616 | 0.598766 | -3.77958 |
| CUST_122799_PI430048170 | 1.81E-05 | -7.87973 | 7.641614 | -3.77558 |
| CUST_17992_PI430048170 | 5.93E-05 | -6.96493 | 5.835825 | -3.77116 |
| CUST_127144_PI430048170 | 4.40E-06 | -9.07686 | 9.824457 | -3.76582 |
| CUST_143157_PI430048170 | 7.46E-04 | -5.26793 | 2.189479 | -3.75242 |
| CUST_134423_PI430048170 | 2.50E-02 | -3.20994 | -2.46262 | -3.74458 |
| CUST_142864_PI430048170 | 7.41E-05 | -6.79109 | 5.479232 | -3.74089 |
| CUST_58235_PI430048170 | 1.53E-04 | -6.27029 | 4.386311 | -3.73701 |
| CUST_145173_PI430048170 | 1.74E-04 | -6.18398 | 4.201719 | -3.73685 |
| CUST_137323_PI430048170 | 4.70E-06 | -9.02234 | 9.729399 | -3.72465 |
| CUST_66504_PI430048170 | 4.44E-05 | -7.18227 | 6.275663 | -3.72057 |
| CUST_141473_PI430048170 | 4.89E-04 | -5.53128 | 2.777074 | -3.69085 |
| CUST_135420_PI430048170 | 7.73E-06 | -8.61352 | 9.003535 | -3.68603 |
| CUST_145392_PI430048170 | 9.18E-05 | -6.63716 | 5.159999 | -3.68601 |
| CUST_104494_PI430048170 | 9.35E-05 | -6.62236 | 5.129149 | -3.68317 |
| CUST_140344_PI430048170 | 4.56E-04 | -5.57516 | 2.874329 | -3.67827 |
| CUST_66800_PI430048170 | 1.89E-04 | -6.12172 | 4.06801 | -3.67731 |
| CUST_16267_PI430048170 | 4.51E-08 | -13.4505 | 16.26244 | -3.67593 |
| CUST_122115_PI430048170 | 2.34E-04 | -5.98384 | 3.770147 | -3.66563 |
| CUST_142995_PI430048170 | 1.46E-06 | -10.0255 | 11.4146 | -3.6631 |
| CUST_140599_PI430048170 | 3.64E-07 | -11.4206 | 13.54672 | -3.66166 |
| CUST_137492_PI430048170 | 1.82E-04 | -6.1524 | 4.133949 | -3.66164 |
| CUST_144934_PI430048170 | 1.54E-03 | -4.83737 | 1.216774 | -3.66075 |
| CUST_55340_PI430048170 | 9.44E-05 | -6.61567 | 5.115178 | -3.65842 |
| CUST_48304_PI430048170 | 6.76E-03 | -3.97786 | -0.74456 | -3.6553 |
| CUST_141076_PI430048170 | 5.22E-03 | -4.12553 | -0.40797 | -3.64257 |
| CUST_107922_PI430048170 | 1.82E-04 | -6.15628 | 4.142289 | -3.63992 |
| CUST_130235_PI430048170 | 7.74E-05 | -6.75782 | 5.41052 | -3.62233 |
| CUST_6627_PI430048170 | 5.89E-03 | -4.05742 | -0.56333 | -3.61783 |
| CUST_136692_PI430048170 | 5.67E-05 | -6.9963 | 5.899709 | -3.61694 |
| CUST_99580_PI430048170 | 6.11E-05 | -6.94431 | 5.79374 | -3.6145 |
| CUST_114837_PI430048170 | 3.17E-05 | -7.45002 | 6.808278 | -3.61285 |
| CUST_74728_PI430048170 | 2.50E-05 | -7.62527 | 7.151349 | -3.60662 |
| CUST_95858_PI430048170 | 2.60E-06 | -9.53805 | 10.61242 | -3.59894 |
| CUST_117376_PI430048170 | 8.24E-09 | -15.5896 | 18.70746 | -3.59419 |
| CUST_88644_PI430048170 | 4.44E-05 | -7.1836 | 6.278333 | -3.58498 |
| CUST_121800_PI430048170 | 1.49E-02 | -3.52009 | -1.77792 | -3.58458 |
| CUST_93134_PI430048170 | 6.34E-05 | -6.90431 | 5.711959 | -3.58227 |
| CUST_128604_PI430048170 | 1.43E-03 | -4.87951 | 1.312494 | -3.57081 |
| CUST_127999_PI430048170 | 4.36E-05 | -7.19944 | 6.310125 | -3.55429 |
| CUST_134957_PI430048170 | 1.92E-04 | -6.11032 | 4.043461 | -3.5523 |
| CUST_130312_PI430048170 | 2.01E-04 | -6.0786 | 3.975106 | -3.53834 |
| CUST_137739_PI430048170 | 4.97E-04 | -5.51911 | 2.750063 | -3.53192 |
| CUST_69649_PI430048170 | 3.23E-04 | -5.7917 | 3.351311 | -3.52592 |
| CUST_17534_PI430048170 | 1.09E-05 | -8.27717 | 8.388776 | -3.52442 |
| CUST_125360_PI430048170 | 2.56E-06 | -9.55268 | 10.63694 | -3.51845 |
| CUST_60014_PI430048170 | 2.46E-05 | -7.64733 | 7.194214 | -3.50993 |
| CUST_97141_PI430048170 | 8.57E-06 | -8.50799 | 8.812363 | -3.50675 |
| CUST_58628_PI430048170 | 8.27E-05 | -6.7144 | 5.320596 | -3.50445 |
| CUST_97885_PI430048170 | 4.07E-05 | -7.25184 | 6.415032 | -3.49886 |
| CUST_119000_PI430048170 | 9.44E-05 | -6.61545 | 5.114717 | -3.49614 |
| CUST_141505_PI430048170 | 6.22E-05 | -6.92762 | 5.759639 | -3.49441 |
| CUST_134076_PI430048170 | 4.13E-06 | -9.12401 | 9.906337 | -3.49116 |
| CUST_99141_PI430048170 | 4.51E-05 | -7.16835 | 6.247683 | -3.48816 |
| CUST_132570_PI430048170 | 8.69E-06 | -8.49148 | 8.782317 | -3.48399 |
| CUST_68699_PI430048170 | 3.01E-05 | -7.49136 | 6.889616 | -3.48339 |
| CUST_77709_PI430048170 | 9.44E-05 | -6.61613 | 5.116141 | -3.48297 |
| CUST_824_PI430048170 | 5.01E-05 | -7.09034 | 6.090436 | -3.47535 |
| CUST_52571_PI430048170 | 1.37E-04 | -6.3459 | 4.547204 | -3.47439 |
| CUST_3180_PI430048170 | 5.09E-05 | -7.07756 | 6.064578 | -3.4737 |
| CUST_131666_PI430048170 | 1.89E-04 | -6.12325 | 4.071296 | -3.47176 |
| CUST_135795_PI430048170 | 2.97E-05 | -7.50101 | 6.908549 | -3.46866 |
| CUST_78765_PI430048170 | 5.96E-03 | -4.05051 | -0.57908 | -3.45173 |
| CUST_80759_PI430048170 | 1.51E-05 | -8.01574 | 7.899837 | -3.44814 |
| CUST_79205_PI430048170 | 2.31E-03 | -4.59655 | 0.668144 | -3.44439 |
| CUST_88678_PI430048170 | 5.90E-05 | -6.96901 | 5.844135 | -3.44381 |
| CUST_136201_PI430048170 | 1.09E-03 | -5.03917 | 1.674265 | -3.44209 |
| CUST_130926_PI430048170 | 7.65E-07 | -10.6111 | 12.33815 | -3.43988 |
| CUST_128362_PI430048170 | 1.53E-04 | -6.26776 | 4.380906 | -3.43825 |
| CUST_90147_PI430048170 | 2.38E-05 | -7.6775 | 7.252729 | -3.43748 |
| CUST_134307_PI430048170 | 2.25E-04 | -6.00769 | 3.821827 | -3.4268 |
| CUST_125440_PI430048170 | 4.81E-04 | -5.54044 | 2.797385 | -3.42458 |
| CUST_17228_PI430048170 | 4.92E-05 | -7.10603 | 6.122136 | -3.42441 |
| CUST_22498_PI430048170 | 5.76E-06 | -8.84041 | 9.409252 | -3.42416 |
| CUST_7715_PI430048170 | 3.35E-04 | -5.76932 | 3.302239 | -3.42302 |
| CUST_52520_PI430048170 | 3.61E-05 | -7.33823 | 6.587151 | -3.42001 |
| CUST_136777_PI430048170 | 1.80E-03 | -4.74365 | 1.003551 | -3.41935 |
| CUST_22500_PI430048170 | 2.29E-04 | -5.99697 | 3.798616 | -3.41219 |
| CUST_127412_PI430048170 | 2.58E-05 | -7.59629 | 7.09492 | -3.39866 |
| CUST_16743_PI430048170 | 2.45E-04 | -5.95802 | 3.714106 | -3.39839 |
| CUST_69663_PI430048170 | 9.47E-05 | -6.6128 | 5.109189 | -3.39757 |
| CUST_73148_PI430048170 | 2.63E-04 | -5.91357 | 3.617479 | -3.39666 |
| CUST_80721_PI430048170 | 1.19E-05 | -8.20931 | 8.262791 | -3.39305 |
| CUST_44425_PI430048170 | 2.90E-08 | -14.0801 | 17.02277 | -3.38915 |
| CUST_131098_PI430048170 | 3.98E-04 | -5.65591 | 3.052785 | -3.38256 |
| CUST_137051_PI430048170 | 2.18E-06 | -9.71018 | 10.89922 | -3.37928 |
| CUST_130289_PI430048170 | 6.84E-05 | -6.84898 | 5.598457 | -3.37906 |
| CUST_64676_PI430048170 | 3.09E-08 | -13.9339 | 16.84934 | -3.37651 |
| CUST_137500_PI430048170 | 1.20E-02 | -3.64838 | -1.49043 | -3.37349 |
| CUST_131331_PI430048170 | 5.89E-04 | -5.41398 | 2.516135 | -3.37149 |
| CUST_133721_PI430048170 | 1.74E-06 | -9.88633 | 11.18872 | -3.37117 |
| CUST_132841_PI430048170 | 6.17E-05 | -6.93556 | 5.775869 | -3.36944 |
| CUST_132146_PI430048170 | 4.46E-05 | -7.17938 | 6.269858 | -3.35796 |
| CUST_136016_PI430048170 | 9.94E-03 | -3.75655 | -1.24658 | -3.35599 |
| CUST_140358_PI430048170 | 9.70E-05 | -6.59671 | 5.075586 | -3.35241 |
| CUST_138893_PI430048170 | 5.06E-04 | -5.50782 | 2.724989 | -3.34557 |
| CUST_138926_PI430048170 | 7.11E-05 | -6.82079 | 5.540464 | -3.34197 |
| CUST_63395_PI430048170 | 8.03E-04 | -5.22452 | 2.092022 | -3.33925 |
| CUST_143989_PI430048170 | 8.48E-05 | -6.69563 | 5.281642 | -3.33724 |
| CUST_140493_PI430048170 | 8.77E-05 | -6.67257 | 5.233724 | -3.33535 |
| CUST_57373_PI430048170 | 3.05E-05 | -7.48017 | 6.867626 | -3.33175 |
| CUST_7719_PI430048170 | 3.31E-04 | -5.77726 | 3.319672 | -3.32901 |
| CUST_135755_PI430048170 | 1.03E-05 | -8.33479 | 8.495216 | -3.32618 |
| CUST_74640_PI430048170 | 6.34E-05 | -6.90539 | 5.714163 | -3.31047 |
| CUST_127206_PI430048170 | 3.89E-06 | -9.17407 | 9.992937 | -3.31033 |
| CUST_125531_PI430048170 | 9.54E-04 | -5.12031 | 1.857472 | -3.30887 |
| CUST_52976_PI430048170 | 6.37E-04 | -5.36515 | 2.407135 | -3.30628 |
| CUST_128462_PI430048170 | 2.14E-03 | -4.6415 | 0.770723 | -3.30176 |
| CUST_89745_PI430048170 | 9.00E-05 | -6.65158 | 5.190044 | -3.30133 |
| CUST_144318_PI430048170 | 8.85E-06 | -8.4667 | 8.737143 | -3.29437 |
| CUST_7708_PI430048170 | 4.48E-04 | -5.58597 | 2.898268 | -3.29229 |
| CUST_57632_PI430048170 | 1.46E-04 | -6.29938 | 4.448306 | -3.28023 |
| CUST_140067_PI430048170 | 7.23E-05 | -6.80746 | 5.512997 | -3.27953 |
| CUST_95319_PI430048170 | 7.40E-04 | -5.27365 | 2.202314 | -3.27687 |
| CUST_93951_PI430048170 | 1.00E-03 | -5.08978 | 1.788584 | -3.27591 |
| CUST_121940_PI430048170 | 3.26E-06 | -9.33983 | 10.27727 | -3.27586 |
| CUST_10974_PI430048170 | 2.14E-04 | -6.03683 | 3.884894 | -3.27559 |
| CUST_138811_PI430048170 | 6.04E-05 | -6.95176 | 5.808963 | -3.27299 |
| CUST_95225_PI430048170 | 4.35E-04 | -5.60416 | 2.938506 | -3.27141 |
| CUST_24589_PI430048170 | 1.24E-04 | -6.41684 | 4.697492 | -3.2708 |
| CUST_113509_PI430048170 | 2.72E-05 | -7.55802 | 7.020216 | -3.2671 |
| CUST_117114_PI430048170 | 2.85E-03 | -4.47234 | 0.384461 | -3.26645 |
| CUST_16741_PI430048170 | 2.73E-04 | -5.8881 | 3.561996 | -3.26604 |
| CUST_7714_PI430048170 | 5.75E-04 | -5.43096 | 2.554001 | -3.26195 |
| CUST_133490_PI430048170 | 5.76E-06 | -8.84085 | 9.410029 | -3.25925 |
| CUST_142443_PI430048170 | 2.28E-05 | -7.70722 | 7.310242 | -3.25897 |
| CUST_99130_PI430048170 | 7.08E-05 | -6.82366 | 5.546364 | -3.25643 |
| CUST_143462_PI430048170 | 2.83E-03 | -4.47557 | 0.391842 | -3.24859 |
| CUST_133602_PI430048170 | 7.66E-05 | -6.76533 | 5.426052 | -3.24765 |
| CUST_134315_PI430048170 | 4.88E-06 | -8.98866 | 9.670486 | -3.24747 |
| CUST_52319_PI430048170 | 2.18E-04 | -6.02726 | 3.864189 | -3.24627 |
| CUST_133605_PI430048170 | 1.61E-04 | -6.23614 | 4.313377 | -3.24568 |
| CUST_140138_PI430048170 | 1.14E-05 | -8.24328 | 8.325937 | -3.2428 |
| CUST_40569_PI430048170 | 8.97E-05 | -6.65776 | 5.202922 | -3.24066 |
| CUST_25343_PI430048170 | 2.10E-04 | -6.05068 | 3.914823 | -3.22435 |
| CUST_141472_PI430048170 | 1.78E-04 | -6.1717 | 4.175381 | -3.21872 |
| CUST_140396_PI430048170 | 6.97E-05 | -6.83579 | 5.571322 | -3.21271 |
| CUST_98176_PI430048170 | 2.44E-04 | -5.95907 | 3.71639 | -3.20967 |
| CUST_61731_PI430048170 | 2.97E-04 | -5.83906 | 3.454954 | -3.20283 |
| CUST_93699_PI430048170 | 6.21E-05 | -6.92901 | 5.762494 | -3.19902 |
| CUST_128320_PI430048170 | 1.04E-05 | -8.32072 | 8.469274 | -3.19759 |
| CUST_137327_PI430048170 | 4.30E-05 | -7.21189 | 6.335089 | -3.19557 |
| CUST_14663_PI430048170 | 8.14E-06 | -8.55614 | 8.899791 | -3.19491 |
| CUST_140133_PI430048170 | 1.70E-04 | -6.20324 | 4.242991 | -3.19224 |
| CUST_72015_PI430048170 | 8.99E-06 | -8.4412 | 8.69058 | -3.19048 |
| CUST_137340_PI430048170 | 8.21E-03 | -3.86709 | -0.99627 | -3.18886 |
| CUST_38199_PI430048170 | 1.57E-05 | -7.98989 | 7.850968 | -3.18727 |
| CUST_134388_PI430048170 | 3.42E-04 | -5.75791 | 3.277214 | -3.18541 |
| CUST_131032_PI430048170 | 5.87E-05 | -6.97449 | 5.855298 | -3.18327 |
| CUST_144424_PI430048170 | 6.37E-05 | -6.9011 | 5.705374 | -3.18204 |
| CUST_98678_PI430048170 | 1.66E-04 | -6.21612 | 4.270561 | -3.18054 |
| CUST_135560_PI430048170 | 1.30E-05 | -8.13592 | 8.125812 | -3.17896 |
| CUST_33996_PI430048170 | 3.76E-02 | -2.96781 | -2.98358 | -3.16992 |
| CUST_127255_PI430048170 | 3.87E-07 | -11.3182 | 13.39799 | -3.16643 |
| CUST_95634_PI430048170 | 1.49E-05 | -8.03788 | 7.941624 | -3.16615 |
| CUST_140859_PI430048170 | 8.30E-06 | -8.53329 | 8.858352 | -3.16517 |
| CUST_143960_PI430048170 | 1.46E-03 | -4.86636 | 1.282634 | -3.16456 |
| CUST_34827_PI430048170 | 1.02E-04 | -6.55456 | 4.987381 | -3.16271 |
| CUST_89389_PI430048170 | 5.52E-03 | -4.09426 | -0.47933 | -3.16176 |
| CUST_138986_PI430048170 | 5.72E-05 | -6.98994 | 5.886781 | -3.15843 |
| CUST_137962_PI430048170 | 1.66E-04 | -6.21815 | 4.274907 | -3.15459 |
| CUST_127270_PI430048170 | 3.36E-05 | -7.40704 | 6.72347 | -3.15423 |
| CUST_134339_PI430048170 | 6.84E-04 | -5.32109 | 2.308601 | -3.15241 |
| CUST_70144_PI430048170 | 2.28E-04 | -6.00053 | 3.806316 | -3.14935 |
| CUST_7713_PI430048170 | 5.62E-04 | -5.44658 | 2.588796 | -3.14463 |
| CUST_143805_PI430048170 | 9.25E-04 | -5.13802 | 1.897389 | -3.13809 |
| CUST_139341_PI430048170 | 5.15E-05 | -7.06934 | 6.047948 | -3.13782 |
| CUST_111469_PI430048170 | 3.96E-05 | -7.27154 | 6.454384 | -3.13718 |
| CUST_114679_PI430048170 | 1.26E-03 | -4.95387 | 1.48119 | -3.13115 |
| CUST_7716_PI430048170 | 7.88E-04 | -5.23621 | 2.118291 | -3.12999 |
| CUST_142269_PI430048170 | 3.13E-03 | -4.4187 | 0.261895 | -3.12838 |
| CUST_52949_PI430048170 | 1.83E-02 | -3.3985 | -2.04829 | -3.12822 |
| CUST_131955_PI430048170 | 3.06E-04 | -5.82211 | 3.417885 | -3.12785 |
| CUST_69919_PI430048170 | 6.91E-04 | -5.31524 | 2.295508 | -3.11944 |
| CUST_68430_PI430048170 | 2.63E-04 | -5.91184 | 3.613706 | -3.11911 |
| CUST_130927_PI430048170 | 4.68E-05 | -7.14358 | 6.19786 | -3.11889 |
| CUST_135072_PI430048170 | 1.39E-03 | -4.89239 | 1.341732 | -3.11378 |
| CUST_9629_PI430048170 | 5.95E-04 | -5.40744 | 2.501569 | -3.11194 |
| CUST_91681_PI430048170 | 4.44E-05 | -7.1874 | 6.285955 | -3.10553 |
| CUST_52465_PI430048170 | 2.56E-04 | -5.93059 | 3.654499 | -3.10466 |
| CUST_36784_PI430048170 | 5.92E-04 | -5.41072 | 2.508864 | -3.10094 |
| CUST_97862_PI430048170 | 2.13E-04 | -6.04235 | 3.896832 | -3.09893 |
| CUST_133980_PI430048170 | 6.30E-05 | -6.9111 | 5.725858 | -3.09827 |
| CUST_94583_PI430048170 | 5.40E-04 | -5.47051 | 2.642051 | -3.09654 |
| CUST_134567_PI430048170 | 4.51E-05 | -7.1701 | 6.251203 | -3.08539 |
| CUST_14411_PI430048170 | 7.27E-04 | -5.28288 | 2.223007 | -3.08478 |
| CUST_95775_PI430048170 | 2.19E-04 | -6.02432 | 3.857839 | -3.08119 |
| CUST_125811_PI430048170 | 4.97E-04 | -5.5195 | 2.750927 | -3.07848 |
| CUST_58632_PI430048170 | 4.54E-06 | -9.0479 | 9.774022 | -3.07538 |
| CUST_130734_PI430048170 | 6.23E-05 | -6.92361 | 5.751449 | -3.06856 |
| CUST_2323_PI430048170 | 3.41E-02 | -3.02713 | -2.85727 | -3.06731 |
| CUST_56380_PI430048170 | 2.94E-03 | -4.45442 | 0.343531 | -3.0645 |
| CUST_134104_PI430048170 | 6.61E-06 | -8.74015 | 9.230846 | -3.05778 |
| CUST_119831_PI430048170 | 2.48E-05 | -7.63651 | 7.173204 | -3.05607 |
| CUST_58630_PI430048170 | 6.22E-06 | -8.77726 | 9.297042 | -3.05199 |
| CUST_58770_PI430048170 | 7.08E-05 | -6.82453 | 5.548155 | -3.05174 |
| CUST_140591_PI430048170 | 8.85E-05 | -6.6674 | 5.222973 | -3.04871 |
| CUST_118749_PI430048170 | 9.74E-05 | -6.59138 | 5.064439 | -3.04754 |
| CUST_14878_PI430048170 | 4.46E-06 | -9.06421 | 9.802435 | -3.04417 |
| CUST_127623_PI430048170 | 8.85E-06 | -8.47387 | 8.750223 | -3.04203 |
| CUST_114688_PI430048170 | 9.97E-06 | -8.36319 | 8.547525 | -3.04101 |
| CUST_22869_PI430048170 | 2.10E-05 | -7.77588 | 7.442655 | -3.04071 |
| CUST_63092_PI430048170 | 2.92E-03 | -4.4594 | 0.354905 | -3.03831 |
| CUST_7726_PI430048170 | 2.03E-03 | -4.67266 | 0.841787 | -3.03271 |
| CUST_119542_PI430048170 | 5.56E-06 | -8.88011 | 9.479497 | -3.03116 |
| CUST_134390_PI430048170 | 9.80E-04 | -5.10471 | 1.822266 | -3.02834 |
| CUST_118379_PI430048170 | 2.87E-06 | -9.46665 | 10.4923 | -3.02801 |
| CUST_36774_PI430048170 | 1.53E-03 | -4.8397 | 1.22207 | -3.02733 |
| CUST_13001_PI430048170 | 2.59E-04 | -5.92326 | 3.638558 | -3.0259 |
| CUST_128039_PI430048170 | 5.98E-05 | -6.96072 | 5.827243 | -3.02502 |
| CUST_137106_PI430048170 | 6.55E-04 | -5.34938 | 2.371896 | -3.02169 |
| CUST_38217_PI430048170 | 7.32E-06 | -8.65567 | 9.079441 | -3.02012 |
| CUST_133892_PI430048170 | 7.33E-05 | -6.79796 | 5.493406 | -3.01861 |
| CUST_137387_PI430048170 | 2.25E-03 | -4.61181 | 0.702989 | -3.0185 |
| CUST_131407_PI430048170 | 9.47E-05 | -6.61255 | 5.108674 | -3.01723 |
| CUST_137333_PI430048170 | 5.97E-07 | -10.8836 | 12.75362 | -3.01691 |
| CUST_102040_PI430048170 | 6.09E-04 | -5.39296 | 2.469247 | -3.01643 |
| CUST_127111_PI430048170 | 8.33E-07 | -10.5404 | 12.22897 | -3.01432 |
| CUST_16771_PI430048170 | 4.13E-05 | -7.24252 | 6.396402 | -3.01277 |
| CUST_136871_PI430048170 | 2.45E-03 | -4.56199 | 0.589257 | -3.00599 |
| CUST_63386_PI430048170 | 4.58E-07 | -11.1541 | 13.15727 | -3.0049 |
| CUST_58757_PI430048170 | 7.27E-07 | -10.6656 | 12.42199 | -2.99914 |
| CUST_100452_PI430048170 | 1.88E-04 | -6.12538 | 4.075877 | -2.99824 |
| CUST_15339_PI430048170 | 1.40E-04 | -6.33002 | 4.513466 | -2.99487 |
| CUST_128575_PI430048170 | 3.26E-05 | -7.42976 | 6.768339 | -2.99321 |
| CUST_140938_PI430048170 | 7.27E-04 | -5.28277 | 2.22275 | -2.98103 |
| CUST_48278_PI430048170 | 1.08E-04 | -6.514 | 4.902263 | -2.97975 |
| CUST_143110_PI430048170 | 5.77E-04 | -5.42792 | 2.547232 | -2.9766 |
| CUST_23647_PI430048170 | 6.76E-04 | -5.32847 | 2.325121 | -2.97638 |
| CUST_61052_PI430048170 | 4.51E-06 | -9.05497 | 9.786353 | -2.97623 |
| CUST_73512_PI430048170 | 1.50E-06 | -9.98702 | 11.35238 | -2.97477 |
| CUST_11909_PI430048170 | 5.41E-05 | -7.03031 | 5.968836 | -2.97051 |
| CUST_73608_PI430048170 | 1.92E-02 | -3.36975 | -2.11186 | -2.96505 |
| CUST_70464_PI430048170 | 1.46E-03 | -4.86868 | 1.287908 | -2.96244 |
| CUST_137830_PI430048170 | 5.48E-05 | -7.02205 | 5.952068 | -2.95929 |
| CUST_128096_PI430048170 | 3.57E-05 | -7.35062 | 6.61174 | -2.95601 |
| CUST_1683_PI430048170 | 6.21E-06 | -8.78177 | 9.305071 | -2.95319 |
| CUST_51009_PI430048170 | 2.50E-03 | -4.54995 | 0.561758 | -2.94861 |
| CUST_141451_PI430048170 | 1.21E-04 | -6.43506 | 4.735989 | -2.94589 |
| CUST_135850_PI430048170 | 4.89E-04 | -5.53221 | 2.779129 | -2.94373 |
| CUST_139366_PI430048170 | 2.90E-08 | -14.1383 | 17.09125 | -2.94274 |
| CUST_56203_PI430048170 | 1.13E-04 | -6.48371 | 4.83856 | -2.9387 |
| CUST_139651_PI430048170 | 2.54E-03 | -4.54014 | 0.539349 | -2.93645 |
| CUST_73728_PI430048170 | 1.75E-02 | -3.42586 | -1.98763 | -2.93393 |
| CUST_58087_PI430048170 | 1.56E-04 | -6.2565 | 4.356876 | -2.93375 |
| CUST_135871_PI430048170 | 3.69E-02 | -2.97963 | -2.95849 | -2.93168 |
| CUST_105825_PI430048170 | 6.16E-06 | -8.79216 | 9.323566 | -2.93074 |
| CUST_136124_PI430048170 | 3.93E-04 | -5.66477 | 3.072323 | -2.93024 |
| CUST_134257_PI430048170 | 4.84E-05 | -7.11906 | 6.148419 | -2.92753 |
| CUST_121013_PI430048170 | 2.14E-04 | -6.03743 | 3.886184 | -2.92742 |
| CUST_132344_PI430048170 | 5.72E-05 | -6.99057 | 5.888054 | -2.92716 |
| CUST_17991_PI430048170 | 1.09E-04 | -6.50735 | 4.888282 | -2.92694 |
| CUST_54163_PI430048170 | 5.88E-04 | -5.41522 | 2.518921 | -2.92518 |
| CUST_91153_PI430048170 | 5.81E-06 | -8.83152 | 9.393487 | -2.92463 |
| CUST_145034_PI430048170 | 7.70E-05 | -6.76201 | 5.419173 | -2.92371 |
| CUST_36567_PI430048170 | 1.62E-03 | -4.80582 | 1.145032 | -2.92286 |
| CUST_7702_PI430048170 | 7.99E-04 | -5.2279 | 2.099629 | -2.92261 |
| CUST_51516_PI430048170 | 4.20E-06 | -9.11005 | 9.88213 | -2.92006 |
| CUST_144028_PI430048170 | 9.98E-07 | -10.3781 | 11.97583 | -2.91873 |
| CUST_27740_PI430048170 | 1.74E-04 | -6.18381 | 4.201363 | -2.91805 |
| CUST_17513_PI430048170 | 8.95E-04 | -5.15725 | 1.940697 | -2.91412 |
| CUST_73280_PI430048170 | 2.31E-07 | -11.847 | 14.153 | -2.90948 |
| CUST_129320_PI430048170 | 3.61E-05 | -7.33913 | 6.588934 | -2.90878 |
| CUST_68281_PI430048170 | 7.67E-06 | -8.62305 | 9.020715 | -2.90645 |
| CUST_42929_PI430048170 | 3.01E-05 | -7.49128 | 6.889456 | -2.90266 |
| CUST_52843_PI430048170 | 1.19E-03 | -4.98591 | 1.553753 | -2.90134 |
| CUST_11328_PI430048170 | 1.15E-03 | -5.00759 | 1.602846 | -2.89911 |
| CUST_141348_PI430048170 | 1.04E-04 | -6.54183 | 4.960692 | -2.89825 |
| CUST_137428_PI430048170 | 1.90E-04 | -6.11857 | 4.061219 | -2.89678 |
| CUST_137298_PI430048170 | 5.01E-05 | -7.08997 | 6.089693 | -2.89652 |
| CUST_108080_PI430048170 | 4.89E-04 | -5.53045 | 2.775234 | -2.8918 |
| CUST_139116_PI430048170 | 4.04E-04 | -5.64588 | 3.030652 | -2.89104 |
| CUST_7723_PI430048170 | 2.91E-04 | -5.85035 | 3.479612 | -2.8868 |
| CUST_25262_PI430048170 | 9.93E-05 | -6.57602 | 5.032311 | -2.87953 |
| CUST_99490_PI430048170 | 8.24E-09 | -15.6072 | 18.72606 | -2.87809 |
| CUST_144551_PI430048170 | 3.50E-05 | -7.36815 | 6.646511 | -2.87515 |
| CUST_78165_PI430048170 | 9.63E-05 | -6.60126 | 5.085095 | -2.87418 |
| CUST_145707_PI430048170 | 6.46E-04 | -5.35655 | 2.387925 | -2.87359 |
| CUST_126640_PI430048170 | 2.67E-04 | -5.90243 | 3.593206 | -2.87217 |
| CUST_96634_PI430048170 | 3.32E-05 | -7.41755 | 6.744244 | -2.87153 |
| CUST_129051_PI430048170 | 2.13E-02 | -3.30795 | -2.24808 | -2.87057 |
| CUST_129418_PI430048170 | 5.81E-04 | -5.42282 | 2.535857 | -2.86822 |
| CUST_134126_PI430048170 | 5.46E-03 | -4.09987 | -0.46651 | -2.86816 |
| CUST_96316_PI430048170 | 2.94E-04 | -5.84557 | 3.469161 | -2.86366 |
| CUST_126851_PI430048170 | 3.28E-03 | -4.39389 | 0.205172 | -2.86125 |
| CUST_114016_PI430048170 | 5.44E-04 | -5.46606 | 2.632162 | -2.86097 |
| CUST_110827_PI430048170 | 1.72E-04 | -6.1935 | 4.222131 | -2.86086 |
| CUST_48122_PI430048170 | 1.07E-03 | -5.05064 | 1.700192 | -2.85795 |
| CUST_73151_PI430048170 | 1.04E-03 | -5.07038 | 1.744777 | -2.85228 |
| CUST_103323_PI430048170 | 2.44E-05 | -7.65316 | 7.205529 | -2.85082 |
| CUST_7741_PI430048170 | 8.63E-04 | -5.18003 | 1.991989 | -2.84992 |
| CUST_29827_PI430048170 | 8.18E-06 | -8.54779 | 8.884656 | -2.84902 |
| CUST_32918_PI430048170 | 2.05E-04 | -6.06572 | 3.947305 | -2.84869 |
| CUST_94912_PI430048170 | 5.63E-05 | -7.00204 | 5.911384 | -2.84805 |
| CUST_129090_PI430048170 | 6.31E-05 | -6.90933 | 5.722227 | -2.84483 |
| CUST_127677_PI430048170 | 1.41E-04 | -6.32354 | 4.499698 | -2.83629 |
| CUST_134804_PI430048170 | 5.29E-04 | -5.48196 | 2.667527 | -2.83616 |
| CUST_129750_PI430048170 | 1.99E-03 | -4.68466 | 0.869138 | -2.83056 |
| CUST_136800_PI430048170 | 4.06E-06 | -9.13586 | 9.926879 | -2.82781 |
| CUST_22640_PI430048170 | 2.20E-02 | -3.29005 | -2.28739 | -2.82552 |
| CUST_99541_PI430048170 | 6.82E-04 | -5.32351 | 2.314011 | -2.82458 |
| CUST_145205_PI430048170 | 8.35E-05 | -6.70608 | 5.30334 | -2.81885 |
| CUST_33710_PI430048170 | 5.26E-04 | -5.48397 | 2.672001 | -2.81766 |
| CUST_132270_PI430048170 | 4.90E-04 | -5.52965 | 2.773457 | -2.81702 |
| CUST_145478_PI430048170 | 1.16E-04 | -6.45808 | 4.78456 | -2.81598 |
| CUST_60143_PI430048170 | 1.04E-05 | -8.3263 | 8.479568 | -2.81402 |
| CUST_112075_PI430048170 | 1.61E-05 | -7.96998 | 7.813252 | -2.8111 |
| CUST_136269_PI430048170 | 4.93E-03 | -4.1582 | -0.33338 | -2.80879 |
| CUST_139495_PI430048170 | 2.58E-05 | -7.59463 | 7.091677 | -2.80548 |
| CUST_111095_PI430048170 | 5.06E-04 | -5.50805 | 2.725494 | -2.80486 |
| CUST_16768_PI430048170 | 3.62E-04 | -5.72133 | 3.196855 | -2.80407 |
| CUST_12993_PI430048170 | 1.38E-04 | -6.34017 | 4.535051 | -2.8033 |
| CUST_70199_PI430048170 | 7.64E-05 | -6.76866 | 5.432928 | -2.80319 |
| CUST_11006_PI430048170 | 1.24E-04 | -6.42104 | 4.706384 | -2.80146 |
| CUST_118541_PI430048170 | 4.44E-05 | -7.18488 | 6.280905 | -2.80103 |
| CUST_41497_PI430048170 | 3.86E-03 | -4.29895 | -0.01184 | -2.79811 |
| CUST_144583_PI430048170 | 7.18E-04 | -5.29003 | 2.23902 | -2.79733 |
| CUST_139597_PI430048170 | 1.25E-04 | -6.41106 | 4.685275 | -2.79608 |
| CUST_82589_PI430048170 | 6.39E-04 | -5.36306 | 2.402478 | -2.78241 |
| CUST_69337_PI430048170 | 1.89E-07 | -12.011 | 14.38093 | -2.78126 |
| CUST_145274_PI430048170 | 1.21E-05 | -8.18718 | 8.221566 | -2.7808 |
| CUST_73229_PI430048170 | 6.77E-06 | -8.7198 | 9.194475 | -2.77951 |
| CUST_687_PI430048170 | 2.51E-03 | -4.54663 | 0.554178 | -2.77834 |
| CUST_132185_PI430048170 | 2.15E-04 | -6.03503 | 3.881007 | -2.77723 |
| CUST_57372_PI430048170 | 2.11E-04 | -6.04817 | 3.909398 | -2.77658 |
| CUST_134753_PI430048170 | 1.05E-05 | -8.30345 | 8.437386 | -2.77636 |
| CUST_103963_PI430048170 | 7.19E-03 | -3.94231 | -0.82543 | -2.77434 |
| CUST_126046_PI430048170 | 1.43E-04 | -6.31306 | 4.477422 | -2.77351 |
| CUST_82202_PI430048170 | 9.42E-04 | -5.12821 | 1.875267 | -2.76938 |
| CUST_127884_PI430048170 | 8.87E-06 | -8.46384 | 8.731932 | -2.76782 |
| CUST_143313_PI430048170 | 9.05E-05 | -6.64791 | 5.182405 | -2.76442 |
| CUST_134145_PI430048170 | 8.38E-05 | -6.70311 | 5.297175 | -2.76191 |
| CUST_117450_PI430048170 | 2.72E-03 | -4.49902 | 0.445438 | -2.76087 |
| CUST_132683_PI430048170 | 9.22E-05 | -6.63037 | 5.145855 | -2.76075 |
| CUST_60551_PI430048170 | 2.00E-04 | -6.08117 | 3.980644 | -2.76066 |
| CUST_103471_PI430048170 | 6.11E-05 | -6.94415 | 5.793422 | -2.75638 |
| CUST_133015_PI430048170 | 4.95E-07 | -11.0495 | 13.00221 | -2.75633 |
| CUST_132183_PI430048170 | 4.47E-04 | -5.58721 | 2.901003 | -2.7515 |
| CUST_50687_PI430048170 | 1.57E-03 | -4.82535 | 1.18944 | -2.75043 |
| CUST_34752_PI430048170 | 2.19E-02 | -3.29111 | -2.28507 | -2.74951 |
| CUST_135188_PI430048170 | 6.95E-05 | -6.83831 | 5.576516 | -2.74927 |
| CUST_138839_PI430048170 | 3.37E-05 | -7.40247 | 6.714452 | -2.7491 |
| CUST_6522_PI430048170 | 9.59E-06 | -8.39004 | 8.596857 | -2.74644 |
| CUST_40264_PI430048170 | 1.30E-05 | -8.1357 | 8.125412 | -2.74343 |
| CUST_135166_PI430048170 | 2.06E-03 | -4.66433 | 0.822786 | -2.74256 |
| CUST_89564_PI430048170 | 1.96E-03 | -4.69264 | 0.887325 | -2.74103 |
| CUST_17532_PI430048170 | 7.80E-07 | -10.5867 | 12.30054 | -2.74021 |
| CUST_137528_PI430048170 | 4.46E-02 | -2.85936 | -3.21203 | -2.73868 |
| CUST_47264_PI430048170 | 1.39E-04 | -6.3345 | 4.523006 | -2.73829 |
| CUST_70063_PI430048170 | 2.00E-04 | -6.08066 | 3.979556 | -2.738 |
| CUST_24487_PI430048170 | 8.58E-04 | -5.18447 | 2.001971 | -2.73663 |
| CUST_59070_PI430048170 | 9.07E-05 | -6.64624 | 5.178935 | -2.736 |
| CUST_123234_PI430048170 | 1.67E-03 | -4.78912 | 1.107055 | -2.73479 |
| CUST_132659_PI430048170 | 3.98E-05 | -7.26597 | 6.443258 | -2.7339 |
| CUST_5020_PI430048170 | 7.69E-06 | -8.61967 | 9.014617 | -2.73369 |
| CUST_42657_PI430048170 | 6.12E-03 | -4.03599 | -0.61217 | -2.73264 |
| CUST_124224_PI430048170 | 3.67E-08 | -13.6329 | 16.48641 | -2.73176 |
| CUST_109446_PI430048170 | 2.70E-02 | -3.16638 | -2.55733 | -2.72931 |
| CUST_66333_PI430048170 | 1.09E-04 | -6.51028 | 4.894455 | -2.72869 |
| CUST_137420_PI430048170 | 5.77E-04 | -5.42691 | 2.544974 | -2.72789 |
| CUST_76167_PI430048170 | 2.29E-04 | -5.99616 | 3.796866 | -2.72753 |
| CUST_24597_PI430048170 | 2.07E-03 | -4.65999 | 0.81289 | -2.72738 |
| CUST_134868_PI430048170 | 2.49E-05 | -7.62984 | 7.160229 | -2.72378 |
| CUST_119464_PI430048170 | 7.60E-04 | -5.25675 | 2.164393 | -2.7214 |
| CUST_143222_PI430048170 | 9.16E-06 | -8.41796 | 8.648045 | -2.71975 |
| CUST_142924_PI430048170 | 3.64E-04 | -5.71618 | 3.185528 | -2.71841 |
| CUST_15072_PI430048170 | 1.41E-04 | -6.32599 | 4.504909 | -2.71813 |
| CUST_144642_PI430048170 | 2.14E-04 | -6.03661 | 3.884424 | -2.71781 |
| CUST_57537_PI430048170 | 1.79E-04 | -6.16857 | 4.168677 | -2.71677 |
| CUST_99064_PI430048170 | 1.16E-03 | -5.00185 | 1.589837 | -2.71253 |
| CUST_73310_PI430048170 | 7.60E-04 | -5.25633 | 2.163452 | -2.70736 |
| CUST_111412_PI430048170 | 3.66E-04 | -5.71248 | 3.177403 | -2.70352 |
| CUST_130428_PI430048170 | 6.11E-05 | -6.94195 | 5.788931 | -2.70341 |
| CUST_24489_PI430048170 | 7.09E-04 | -5.29722 | 2.255149 | -2.69769 |
| CUST_139530_PI430048170 | 2.19E-04 | -6.02412 | 3.857398 | -2.69625 |
| CUST_14666_PI430048170 | 4.86E-06 | -8.99602 | 9.68337 | -2.6946 |
| CUST_18479_PI430048170 | 2.19E-04 | -6.02294 | 3.854848 | -2.69315 |
| CUST_121889_PI430048170 | 2.18E-02 | -3.29361 | -2.27959 | -2.69311 |
| CUST_16772_PI430048170 | 1.12E-04 | -6.4903 | 4.852444 | -2.69268 |
| CUST_83922_PI430048170 | 3.82E-04 | -5.68393 | 3.114558 | -2.6925 |
| CUST_104602_PI430048170 | 1.09E-04 | -6.50719 | 4.887948 | -2.68556 |
| CUST_139284_PI430048170 | 1.68E-03 | -4.784 | 1.095387 | -2.68375 |
| CUST_28230_PI430048170 | 5.59E-04 | -5.44967 | 2.595677 | -2.67679 |
| CUST_112408_PI430048170 | 2.00E-04 | -6.08292 | 3.984428 | -2.67388 |
| CUST_135098_PI430048170 | 2.05E-02 | -3.32926 | -2.20119 | -2.67353 |
| CUST_81553_PI430048170 | 2.73E-04 | -5.88845 | 3.562751 | -2.67298 |
| CUST_104095_PI430048170 | 2.49E-03 | -4.55129 | 0.564828 | -2.67278 |
| CUST_126436_PI430048170 | 7.77E-06 | -8.60895 | 8.995285 | -2.6707 |
| CUST_52826_PI430048170 | 4.11E-05 | -7.24618 | 6.403728 | -2.67033 |
| CUST_138501_PI430048170 | 2.08E-05 | -7.78441 | 7.45905 | -2.6669 |
| CUST_136487_PI430048170 | 1.05E-05 | -8.30281 | 8.436196 | -2.665 |
| CUST_20761_PI430048170 | 2.75E-03 | -4.49336 | 0.432488 | -2.6634 |
| CUST_145396_PI430048170 | 2.31E-04 | -5.99047 | 3.784519 | -2.66256 |
| CUST_133705_PI430048170 | 1.57E-05 | -7.99048 | 7.852074 | -2.66112 |
| CUST_92064_PI430048170 | 8.10E-03 | -3.87519 | -0.97791 | -2.65999 |
| CUST_17990_PI430048170 | 5.80E-04 | -5.42365 | 2.537711 | -2.65954 |
| CUST_111997_PI430048170 | 2.33E-03 | -4.58968 | 0.652474 | -2.65913 |
| CUST_92435_PI430048170 | 1.51E-05 | -8.01905 | 7.906097 | -2.65478 |
| CUST_95633_PI430048170 | 8.60E-04 | -5.18284 | 1.998317 | -2.65314 |
| CUST_42575_PI430048170 | 1.01E-03 | -5.08522 | 1.778293 | -2.64333 |
| CUST_116658_PI430048170 | 3.62E-04 | -5.72095 | 3.196033 | -2.64163 |
| CUST_134198_PI430048170 | 6.73E-04 | -5.33231 | 2.333707 | -2.63586 |
| CUST_136415_PI430048170 | 2.16E-04 | -6.03263 | 3.875819 | -2.63574 |
| CUST_2341_PI430048170 | 2.21E-02 | -3.28538 | -2.29764 | -2.63526 |
| CUST_134432_PI430048170 | 2.88E-03 | -4.46717 | 0.372649 | -2.63372 |
| CUST_139654_PI430048170 | 5.55E-04 | -5.45437 | 2.606146 | -2.63303 |
| CUST_136614_PI430048170 | 8.99E-05 | -6.65308 | 5.193166 | -2.6319 |
| CUST_135242_PI430048170 | 1.62E-04 | -6.23221 | 4.304995 | -2.63147 |
| CUST_125047_PI430048170 | 8.14E-06 | -8.56083 | 8.908276 | -2.63009 |
| CUST_131528_PI430048170 | 6.49E-05 | -6.88633 | 5.675127 | -2.62827 |
| CUST_129937_PI430048170 | 2.02E-03 | -4.67447 | 0.845907 | -2.62767 |
| CUST_111617_PI430048170 | 5.32E-04 | -5.47753 | 2.657672 | -2.6273 |
| CUST_129025_PI430048170 | 2.58E-02 | -3.1929 | -2.49971 | -2.62654 |
| CUST_12331_PI430048170 | 2.26E-04 | -6.00622 | 3.818642 | -2.62485 |
| CUST_104280_PI430048170 | 5.01E-04 | -5.51504 | 2.741029 | -2.62411 |
| CUST_19087_PI430048170 | 4.58E-07 | -11.152 | 13.15427 | -2.62185 |
| CUST_78711_PI430048170 | 1.03E-04 | -6.54766 | 4.972912 | -2.62127 |
| CUST_17533_PI430048170 | 8.14E-06 | -8.56114 | 8.908849 | -2.62035 |
| CUST_51245_PI430048170 | 1.61E-04 | -6.23634 | 4.313806 | -2.61939 |
| CUST_104671_PI430048170 | 2.47E-05 | -7.64111 | 7.182134 | -2.61649 |
| CUST_134598_PI430048170 | 3.05E-05 | -7.48 | 6.867281 | -2.61643 |
| CUST_117707_PI430048170 | 7.03E-04 | -5.30211 | 2.266102 | -2.61565 |
| CUST_132777_PI430048170 | 1.35E-05 | -8.11286 | 8.082613 | -2.61546 |
| CUST_70836_PI430048170 | 1.37E-02 | -3.57115 | -1.66375 | -2.61455 |
| CUST_143486_PI430048170 | 8.15E-03 | -3.87086 | -0.98771 | -2.61374 |
| CUST_64563_PI430048170 | 4.05E-03 | -4.27306 | -0.071 | -2.61148 |
| CUST_144913_PI430048170 | 8.88E-05 | -6.66432 | 5.216562 | -2.61037 |
| CUST_127882_PI430048170 | 2.24E-05 | -7.72903 | 7.352377 | -2.61013 |
| CUST_126055_PI430048170 | 4.29E-05 | -7.21315 | 6.337601 | -2.60942 |
| CUST_83989_PI430048170 | 3.93E-07 | -11.2982 | 13.36891 | -2.60859 |
| CUST_137510_PI430048170 | 7.91E-05 | -6.7432 | 5.380266 | -2.60523 |
| CUST_25903_PI430048170 | 2.08E-03 | -4.65701 | 0.806094 | -2.60396 |
| CUST_24829_PI430048170 | 1.81E-05 | -7.88358 | 7.648944 | -2.59789 |
| CUST_16783_PI430048170 | 9.67E-05 | -6.59879 | 5.079934 | -2.5958 |
| CUST_138256_PI430048170 | 2.33E-03 | -4.58999 | 0.653178 | -2.59528 |
| CUST_16933_PI430048170 | 8.14E-06 | -8.5636 | 8.913309 | -2.59508 |
| CUST_131664_PI430048170 | 3.15E-06 | -9.37326 | 10.33417 | -2.59079 |
| CUST_46236_PI430048170 | 1.43E-03 | -4.87714 | 1.307126 | -2.59074 |
| CUST_79755_PI430048170 | 3.74E-04 | -5.69842 | 3.146453 | -2.58892 |
| CUST_119319_PI430048170 | 6.72E-05 | -6.8596 | 5.620274 | -2.58849 |
| CUST_79928_PI430048170 | 2.02E-06 | -9.77385 | 11.00433 | -2.58752 |
| CUST_35248_PI430048170 | 1.22E-03 | -4.97398 | 1.52675 | -2.58554 |
| CUST_24436_PI430048170 | 2.06E-05 | -7.78976 | 7.469318 | -2.58305 |
| CUST_131736_PI430048170 | 2.04E-03 | -4.67008 | 0.835897 | -2.58284 |
| CUST_136763_PI430048170 | 2.72E-04 | -5.89164 | 3.569706 | -2.58032 |
| CUST_77415_PI430048170 | 2.49E-05 | -7.6314 | 7.163274 | -2.57818 |
| CUST_73427_PI430048170 | 3.29E-03 | -4.39124 | 0.199117 | -2.57815 |
| CUST_141825_PI430048170 | 8.61E-05 | -6.68593 | 5.261503 | -2.57582 |
| CUST_55239_PI430048170 | 4.26E-04 | -5.61522 | 2.962957 | -2.57512 |
| CUST_29958_PI430048170 | 7.74E-07 | -10.5976 | 12.31742 | -2.57193 |
| CUST_59516_PI430048170 | 8.35E-05 | -6.70575 | 5.302649 | -2.56925 |
| CUST_128488_PI430048170 | 2.08E-04 | -6.05798 | 3.930602 | -2.56643 |
| CUST_142917_PI430048170 | 3.20E-05 | -7.44323 | 6.794905 | -2.56431 |
| CUST_26851_PI430048170 | 3.24E-04 | -5.7888 | 3.344957 | -2.56312 |
| CUST_55017_PI430048170 | 2.29E-02 | -3.26385 | -2.34484 | -2.5618 |
| CUST_134195_PI430048170 | 3.40E-05 | -7.39309 | 6.695888 | -2.55827 |
| CUST_144822_PI430048170 | 3.01E-05 | -7.49064 | 6.888184 | -2.55761 |
| CUST_114037_PI430048170 | 2.65E-04 | -5.90773 | 3.604751 | -2.55304 |
| CUST_78360_PI430048170 | 7.42E-05 | -6.79012 | 5.47724 | -2.55251 |
| CUST_7736_PI430048170 | 2.58E-02 | -3.19325 | -2.49896 | -2.54826 |
| CUST_143636_PI430048170 | 7.05E-04 | -5.30047 | 2.262416 | -2.54729 |
| CUST_27554_PI430048170 | 4.31E-03 | -4.23677 | -0.15394 | -2.54602 |
| CUST_19906_PI430048170 | 1.81E-04 | -6.15882 | 4.147749 | -2.54391 |
| CUST_41623_PI430048170 | 1.10E-03 | -5.03353 | 1.661508 | -2.54358 |
| CUST_110518_PI430048170 | 1.86E-04 | -6.13558 | 4.09782 | -2.5423 |
| CUST_18103_PI430048170 | 5.22E-03 | -4.12523 | -0.40865 | -2.54147 |
| CUST_119474_PI430048170 | 5.74E-08 | -13.1802 | 15.92469 | -2.5398 |
| CUST_89779_PI430048170 | 5.81E-04 | -5.42236 | 2.534836 | -2.53834 |
| CUST_70764_PI430048170 | 1.69E-04 | -6.20652 | 4.250031 | -2.53781 |
| CUST_31211_PI430048170 | 3.68E-04 | -5.70788 | 3.167267 | -2.53283 |
| CUST_138678_PI430048170 | 1.69E-03 | -4.77915 | 1.084369 | -2.53132 |
| CUST_127372_PI430048170 | 6.23E-05 | -6.92145 | 5.747028 | -2.5302 |
| CUST_63690_PI430048170 | 7.83E-05 | -6.75065 | 5.395696 | -2.52783 |
| CUST_73790_PI430048170 | 2.04E-05 | -7.80112 | 7.491146 | -2.52754 |
| CUST_120116_PI430048170 | 3.43E-06 | -9.2966 | 10.20346 | -2.52444 |
| CUST_124156_PI430048170 | 6.48E-03 | -4.00227 | -0.68897 | -2.52335 |
| CUST_121012_PI430048170 | 2.27E-05 | -7.71588 | 7.326982 | -2.52041 |
| CUST_126992_PI430048170 | 7.43E-04 | -5.27003 | 2.194187 | -2.52022 |
| CUST_132402_PI430048170 | 1.88E-02 | -3.38196 | -2.08489 | -2.51874 |
| CUST_95280_PI430048170 | 9.03E-05 | -6.6491 | 5.184884 | -2.51622 |
| CUST_71577_PI430048170 | 4.88E-03 | -4.16521 | -0.31738 | -2.51569 |
| CUST_123183_PI430048170 | 8.20E-04 | -5.21075 | 2.061075 | -2.51432 |
| CUST_57084_PI430048170 | 5.79E-04 | -5.42502 | 2.540761 | -2.51294 |
| CUST_94590_PI430048170 | 4.05E-03 | -4.27167 | -0.07419 | -2.51169 |
| CUST_127450_PI430048170 | 1.87E-04 | -6.13008 | 4.085975 | -2.51108 |
| CUST_129844_PI430048170 | 1.58E-05 | -7.98712 | 7.845725 | -2.50916 |
| CUST_141495_PI430048170 | 1.44E-05 | -8.06438 | 7.991554 | -2.50863 |
| CUST_68233_PI430048170 | 2.21E-04 | -6.01834 | 3.844902 | -2.5076 |
| CUST_57701_PI430048170 | 6.52E-03 | -3.99915 | -0.69608 | -2.506 |
| CUST_135833_PI430048170 | 3.53E-05 | -7.36048 | 6.6313 | -2.50321 |
| CUST_114007_PI430048170 | 1.36E-04 | -6.34951 | 4.554864 | -2.50282 |
| CUST_134479_PI430048170 | 1.18E-03 | -4.99203 | 1.567628 | -2.50164 |
| CUST_18777_PI430048170 | 1.46E-05 | -8.05844 | 7.980371 | -2.49583 |
| CUST_139531_PI430048170 | 8.74E-03 | -3.83277 | -1.0741 | -2.49352 |
| CUST_100267_PI430048170 | 1.85E-04 | -6.14152 | 4.110581 | -2.49316 |
| CUST_120406_PI430048170 | 2.58E-03 | -4.52956 | 0.515195 | -2.49313 |
| CUST_141719_PI430048170 | 1.66E-03 | -4.79272 | 1.115239 | -2.48936 |
| CUST_143139_PI430048170 | 9.03E-06 | -8.42827 | 8.666927 | -2.48846 |
| CUST_93214_PI430048170 | 6.56E-05 | -6.87546 | 5.652823 | -2.48678 |
| CUST_126760_PI430048170 | 1.74E-03 | -4.76215 | 1.045671 | -2.48602 |
| CUST_135805_PI430048170 | 3.68E-06 | -9.23564 | 10.09898 | -2.48428 |
| CUST_126960_PI430048170 | 2.39E-05 | -7.67161 | 7.241323 | -2.48419 |
| CUST_90215_PI430048170 | 4.40E-06 | -9.07898 | 9.828142 | -2.48317 |
| CUST_65917_PI430048170 | 3.85E-04 | -5.67858 | 3.102759 | -2.48275 |
| CUST_141996_PI430048170 | 7.60E-04 | -5.25647 | 2.163762 | -2.48253 |
| CUST_89404_PI430048170 | 3.02E-04 | -5.8301 | 3.435355 | -2.4814 |
| CUST_29789_PI430048170 | 5.80E-03 | -4.06583 | -0.54416 | -2.47975 |
| CUST_133782_PI430048170 | 2.91E-04 | -5.85198 | 3.483174 | -2.47882 |
| CUST_80736_PI430048170 | 6.63E-05 | -6.86939 | 5.640378 | -2.47617 |
| CUST_113651_PI430048170 | 9.87E-06 | -8.37271 | 8.565031 | -2.47531 |
| CUST_100198_PI430048170 | 2.77E-06 | -9.49815 | 10.54538 | -2.47466 |
| CUST_102245_PI430048170 | 8.99E-06 | -8.44607 | 8.69948 | -2.47307 |
| CUST_55464_PI430048170 | 3.14E-03 | -4.41657 | 0.257026 | -2.47259 |
| CUST_58002_PI430048170 | 4.05E-04 | -5.64538 | 3.029563 | -2.47214 |
| CUST_126474_PI430048170 | 3.10E-06 | -9.3994 | 10.37853 | -2.46997 |
| CUST_75496_PI430048170 | 1.26E-02 | -3.61692 | -1.56112 | -2.46963 |
| CUST_128713_PI430048170 | 3.62E-04 | -5.72209 | 3.198521 | -2.4696 |
| CUST_128014_PI430048170 | 4.44E-05 | -7.18532 | 6.28178 | -2.4682 |
| CUST_133102_PI430048170 | 2.18E-06 | -9.71954 | 10.91471 | -2.4676 |
| CUST_42850_PI430048170 | 2.35E-03 | -4.58401 | 0.639535 | -2.46715 |
| CUST_134544_PI430048170 | 2.95E-06 | -9.44249 | 10.4515 | -2.46657 |
| CUST_52073_PI430048170 | 1.08E-05 | -8.28455 | 8.402431 | -2.46579 |
| CUST_136381_PI430048170 | 5.63E-04 | -5.44452 | 2.58421 | -2.46515 |
| CUST_82264_PI430048170 | 2.84E-03 | -4.47502 | 0.390589 | -2.46333 |
| CUST_126888_PI430048170 | 2.70E-02 | -3.16663 | -2.5568 | -2.46237 |
| CUST_65261_PI430048170 | 4.68E-05 | -7.1449 | 6.20051 | -2.46221 |
| CUST_119318_PI430048170 | 1.61E-03 | -4.80878 | 1.151765 | -2.46209 |
| CUST_138603_PI430048170 | 1.91E-05 | -7.84631 | 7.577748 | -2.46056 |
| CUST_82959_PI430048170 | 3.72E-04 | -5.70092 | 3.151967 | -2.46047 |
| CUST_126925_PI430048170 | 1.29E-03 | -4.93731 | 1.443645 | -2.4598 |
| CUST_135185_PI430048170 | 8.18E-06 | -8.54792 | 8.88489 | -2.45707 |
| CUST_143743_PI430048170 | 5.05E-03 | -4.14553 | -0.36231 | -2.45258 |
| CUST_92142_PI430048170 | 1.65E-03 | -4.79485 | 1.120087 | -2.45195 |
| CUST_134427_PI430048170 | 3.44E-05 | -7.38115 | 6.672269 | -2.45063 |
| CUST_14167_PI430048170 | 6.19E-03 | -4.02895 | -0.62822 | -2.45054 |
| CUST_86871_PI430048170 | 1.03E-03 | -5.07066 | 1.745411 | -2.44911 |
| CUST_96914_PI430048170 | 1.07E-03 | -5.04833 | 1.694963 | -2.44812 |
| CUST_14638_PI430048170 | 2.80E-04 | -5.87448 | 3.53229 | -2.447 |
| CUST_94003_PI430048170 | 3.46E-03 | -4.36318 | 0.134982 | -2.44647 |
| CUST_138453_PI430048170 | 2.35E-05 | -7.68491 | 7.267084 | -2.44583 |
| CUST_145060_PI430048170 | 3.82E-04 | -5.6839 | 3.114482 | -2.44418 |
| CUST_99620_PI430048170 | 1.74E-05 | -7.91242 | 7.703911 | -2.44184 |
| CUST_56856_PI430048170 | 1.50E-03 | -4.85264 | 1.251462 | -2.43992 |
| CUST_118563_PI430048170 | 3.66E-04 | -5.71131 | 3.174828 | -2.43926 |
| CUST_142834_PI430048170 | 8.90E-05 | -6.66232 | 5.212411 | -2.43906 |
| CUST_127067_PI430048170 | 5.96E-04 | -5.40647 | 2.499387 | -2.43804 |
| CUST_121811_PI430048170 | 3.82E-03 | -4.30488 | 0.001712 | -2.4378 |
| CUST_136315_PI430048170 | 1.22E-03 | -4.9729 | 1.524302 | -2.43754 |
| CUST_129836_PI430048170 | 7.49E-04 | -5.26549 | 2.184006 | -2.43562 |
| CUST_7720_PI430048170 | 1.04E-02 | -3.73158 | -1.30299 | -2.43501 |
| CUST_95309_PI430048170 | 6.62E-06 | -8.73593 | 9.223312 | -2.43489 |
| CUST_86560_PI430048170 | 9.14E-07 | -10.4599 | 12.10376 | -2.43439 |
| CUST_80849_PI430048170 | 1.66E-05 | -7.9498 | 7.774984 | -2.43418 |
| CUST_64306_PI430048170 | 3.37E-05 | -7.40272 | 6.714945 | -2.42924 |
| CUST_129021_PI430048170 | 1.74E-03 | -4.7644 | 1.0508 | -2.42726 |
| CUST_130124_PI430048170 | 4.42E-02 | -2.86588 | -3.1984 | -2.42414 |
| CUST_137743_PI430048170 | 5.89E-05 | -6.97076 | 5.847695 | -2.42259 |
| CUST_111918_PI430048170 | 3.62E-05 | -7.33586 | 6.582435 | -2.42206 |
| CUST_95603_PI430048170 | 5.01E-04 | -5.5149 | 2.740722 | -2.42164 |
| CUST_20344_PI430048170 | 6.61E-03 | -3.99028 | -0.71628 | -2.41997 |
| CUST_89274_PI430048170 | 1.60E-02 | -3.47563 | -1.87702 | -2.41993 |
| CUST_127603_PI430048170 | 4.79E-03 | -4.17627 | -0.29212 | -2.41962 |
| CUST_70887_PI430048170 | 6.23E-05 | -6.92181 | 5.747776 | -2.41907 |
| CUST_127831_PI430048170 | 9.98E-04 | -5.09288 | 1.795577 | -2.41809 |
| CUST_137967_PI430048170 | 1.49E-05 | -8.04102 | 7.947553 | -2.41606 |
| CUST_129319_PI430048170 | 1.01E-03 | -5.08202 | 1.771078 | -2.41596 |
| CUST_84033_PI430048170 | 5.78E-04 | -5.42583 | 2.54257 | -2.41595 |
| CUST_116194_PI430048170 | 1.59E-03 | -4.81866 | 1.174244 | -2.41585 |
| CUST_128649_PI430048170 | 3.38E-05 | -7.39779 | 6.705198 | -2.41476 |
| CUST_134083_PI430048170 | 6.23E-05 | -6.92144 | 5.747001 | -2.41437 |
| CUST_54502_PI430048170 | 6.10E-05 | -6.94653 | 5.798276 | -2.41182 |
| CUST_99971_PI430048170 | 8.16E-04 | -5.21402 | 2.068424 | -2.41137 |
| CUST_75099_PI430048170 | 9.80E-04 | -5.10494 | 1.82279 | -2.41076 |
| CUST_54492_PI430048170 | 1.30E-02 | -3.59891 | -1.60153 | -2.40939 |
| CUST_12996_PI430048170 | 3.61E-03 | -4.33787 | 0.077129 | -2.40807 |
| CUST_126738_PI430048170 | 1.98E-04 | -6.08753 | 3.994363 | -2.40764 |
| CUST_121156_PI430048170 | 3.96E-04 | -5.66022 | 3.062294 | -2.40742 |
| CUST_36953_PI430048170 | 1.64E-04 | -6.22582 | 4.291331 | -2.40718 |
| CUST_27605_PI430048170 | 3.44E-04 | -5.75357 | 3.267691 | -2.40661 |
| CUST_96911_PI430048170 | 1.28E-03 | -4.94542 | 1.462021 | -2.4045 |
| CUST_88152_PI430048170 | 2.59E-04 | -5.92425 | 3.640715 | -2.40336 |
| CUST_143002_PI430048170 | 1.51E-05 | -8.02515 | 7.917602 | -2.4026 |
| CUST_4417_PI430048170 | 3.63E-04 | -5.71854 | 3.190716 | -2.40165 |
| CUST_73346_PI430048170 | 1.86E-04 | -6.13531 | 4.097234 | -2.40108 |
| CUST_55036_PI430048170 | 5.33E-05 | -7.04232 | 5.993202 | -2.39974 |
| CUST_104701_PI430048170 | 3.04E-03 | -4.43667 | 0.302952 | -2.39529 |
| CUST_25538_PI430048170 | 1.49E-02 | -3.51735 | -1.78402 | -2.3938 |
| CUST_109225_PI430048170 | 6.95E-06 | -8.69628 | 9.152353 | -2.3938 |
| CUST_15961_PI430048170 | 1.28E-03 | -4.94546 | 1.462112 | -2.39305 |
| CUST_58234_PI430048170 | 8.96E-05 | -6.65829 | 5.204022 | -2.38749 |
| CUST_4650_PI430048170 | 7.11E-04 | -5.29521 | 2.250636 | -2.38569 |
| CUST_134976_PI430048170 | 4.39E-04 | -5.59787 | 2.924583 | -2.38504 |
| CUST_18774_PI430048170 | 4.51E-05 | -7.16894 | 6.248882 | -2.38451 |
| CUST_145239_PI430048170 | 1.53E-04 | -6.27108 | 4.387996 | -2.38419 |
| CUST_128407_PI430048170 | 1.11E-04 | -6.49628 | 4.865018 | -2.38418 |
| CUST_81439_PI430048170 | 3.12E-02 | -3.08046 | -2.74294 | -2.38283 |
| CUST_123496_PI430048170 | 1.07E-03 | -5.05298 | 1.705483 | -2.38215 |
| CUST_133655_PI430048170 | 1.48E-03 | -4.85956 | 1.267188 | -2.3813 |
| CUST_18773_PI430048170 | 1.46E-04 | -6.2995 | 4.448546 | -2.38066 |
| CUST_70131_PI430048170 | 7.82E-04 | -5.24014 | 2.127113 | -2.37683 |
| CUST_108041_PI430048170 | 2.48E-04 | -5.94998 | 3.696654 | -2.37625 |
| CUST_142594_PI430048170 | 1.52E-06 | -9.97484 | 11.33266 | -2.37509 |
| CUST_118723_PI430048170 | 9.22E-05 | -6.63023 | 5.145569 | -2.37365 |
| CUST_5647_PI430048170 | 3.76E-04 | -5.69454 | 3.137906 | -2.37138 |
| CUST_142926_PI430048170 | 6.97E-04 | -5.30835 | 2.280063 | -2.37136 |
| CUST_139677_PI430048170 | 4.63E-02 | -2.83514 | -3.26258 | -2.36925 |
| CUST_49451_PI430048170 | 4.79E-04 | -5.54327 | 2.803666 | -2.36717 |
| CUST_118383_PI430048170 | 3.96E-04 | -5.66098 | 3.063976 | -2.36635 |
| CUST_66894_PI430048170 | 2.33E-02 | -3.25285 | -2.36892 | -2.36391 |
| CUST_51405_PI430048170 | 8.07E-04 | -5.22149 | 2.085218 | -2.36367 |
| CUST_139737_PI430048170 | 3.45E-03 | -4.36429 | 0.137509 | -2.36286 |
| CUST_120582_PI430048170 | 6.91E-05 | -6.8433 | 5.586773 | -2.36226 |
| CUST_137054_PI430048170 | 1.33E-03 | -4.92165 | 1.408127 | -2.36194 |
| CUST_134662_PI430048170 | 4.86E-05 | -7.11502 | 6.140278 | -2.36091 |
| CUST_123427_PI430048170 | 1.46E-05 | -8.05739 | 7.978387 | -2.35908 |
| CUST_130782_PI430048170 | 2.91E-03 | -4.46109 | 0.358764 | -2.35895 |
| CUST_122370_PI430048170 | 1.95E-04 | -6.09678 | 4.014304 | -2.35759 |
| CUST_73577_PI430048170 | 4.28E-02 | -2.88503 | -3.15826 | -2.35652 |
| CUST_110471_PI430048170 | 1.39E-04 | -6.33564 | 4.525417 | -2.35544 |
| CUST_52607_PI430048170 | 4.32E-04 | -5.6084 | 2.947872 | -2.35443 |
| CUST_16769_PI430048170 | 2.70E-04 | -5.89715 | 3.58171 | -2.35063 |
| CUST_139868_PI430048170 | 4.94E-04 | -5.52473 | 2.76254 | -2.34848 |
| CUST_75304_PI430048170 | 5.20E-05 | -7.06219 | 6.033469 | -2.34829 |
| CUST_31033_PI430048170 | 1.30E-04 | -6.38187 | 4.623488 | -2.344 |
| CUST_121404_PI430048170 | 4.92E-05 | -7.10668 | 6.123439 | -2.34253 |
| CUST_142580_PI430048170 | 2.79E-03 | -4.48528 | 0.414045 | -2.34108 |
| CUST_123670_PI430048170 | 1.67E-03 | -4.78949 | 1.107881 | -2.34092 |
| CUST_62306_PI430048170 | 8.37E-05 | -6.70429 | 5.299628 | -2.3407 |
| CUST_136951_PI430048170 | 9.14E-07 | -10.4627 | 12.10819 | -2.34049 |
| CUST_112303_PI430048170 | 3.89E-03 | -4.29498 | -0.0209 | -2.33911 |
| CUST_142587_PI430048170 | 2.35E-03 | -4.5844 | 0.640416 | -2.33797 |
| CUST_97105_PI430048170 | 1.05E-04 | -6.53354 | 4.943293 | -2.33739 |
| CUST_108290_PI430048170 | 9.54E-04 | -5.12028 | 1.857397 | -2.33538 |
| CUST_5810_PI430048170 | 8.62E-05 | -6.68497 | 5.259502 | -2.33484 |
| CUST_65250_PI430048170 | 7.44E-05 | -6.78781 | 5.472481 | -2.33467 |
| CUST_50524_PI430048170 | 2.27E-03 | -4.60584 | 0.689364 | -2.33339 |
| CUST_80031_PI430048170 | 1.00E-02 | -3.75218 | -1.25645 | -2.3328 |
| CUST_107953_PI430048170 | 1.10E-03 | -5.03188 | 1.657771 | -2.33051 |
| CUST_127065_PI430048170 | 2.59E-04 | -5.92211 | 3.636063 | -2.32893 |
| CUST_21592_PI430048170 | 8.85E-05 | -6.66717 | 5.2225 | -2.32782 |
| CUST_123445_PI430048170 | 3.50E-05 | -7.37088 | 6.651926 | -2.32702 |
| CUST_140745_PI430048170 | 1.41E-04 | -6.32308 | 4.498727 | -2.32549 |
| CUST_125045_PI430048170 | 6.29E-04 | -5.37292 | 2.424509 | -2.3248 |
| CUST_61897_PI430048170 | 1.47E-03 | -4.86429 | 1.277947 | -2.32365 |
| CUST_110565_PI430048170 | 8.42E-06 | -8.52278 | 8.839262 | -2.32333 |
| CUST_92479_PI430048170 | 3.87E-06 | -9.18178 | 10.00626 | -2.32331 |
| CUST_109319_PI430048170 | 1.16E-02 | -3.66572 | -1.45144 | -2.32151 |
| CUST_138165_PI430048170 | 1.25E-04 | -6.41453 | 4.692614 | -2.32057 |
| CUST_124876_PI430048170 | 3.78E-04 | -5.68967 | 3.127184 | -2.32055 |
| CUST_32967_PI430048170 | 6.54E-05 | -6.87738 | 5.656757 | -2.32033 |
| CUST_57180_PI430048170 | 3.31E-02 | -3.04487 | -2.81931 | -2.31624 |
| CUST_120285_PI430048170 | 4.00E-03 | -4.28036 | -0.05433 | -2.31469 |
| CUST_15523_PI430048170 | 1.46E-03 | -4.86924 | 1.289179 | -2.31394 |
| CUST_57069_PI430048170 | 6.54E-04 | -5.3503 | 2.373947 | -2.31161 |
| CUST_134589_PI430048170 | 6.25E-03 | -4.02418 | -0.63907 | -2.31104 |
| CUST_88363_PI430048170 | 2.21E-05 | -7.73849 | 7.370626 | -2.31047 |
| CUST_39879_PI430048170 | 1.65E-03 | -4.79443 | 1.11912 | -2.30981 |
| CUST_106393_PI430048170 | 1.10E-03 | -5.0349 | 1.664617 | -2.30953 |
| CUST_5314_PI430048170 | 4.26E-04 | -5.61555 | 2.963675 | -2.30823 |
| CUST_129628_PI430048170 | 2.52E-05 | -7.61708 | 7.135401 | -2.30795 |
| CUST_141963_PI430048170 | 8.85E-06 | -8.47431 | 8.751026 | -2.3058 |
| CUST_142070_PI430048170 | 1.05E-03 | -5.06516 | 1.732996 | -2.305 |
| CUST_64816_PI430048170 | 3.25E-04 | -5.78681 | 3.340595 | -2.30499 |
| CUST_38763_PI430048170 | 1.82E-04 | -6.15442 | 4.138296 | -2.30358 |
| CUST_58295_PI430048170 | 7.16E-05 | -6.81417 | 5.526822 | -2.30242 |
| CUST_40251_PI430048170 | 5.38E-05 | -7.03523 | 5.978823 | -2.29844 |
| CUST_56299_PI430048170 | 5.20E-05 | -7.0634 | 6.035923 | -2.29804 |
| CUST_67776_PI430048170 | 1.90E-04 | -6.11675 | 4.057313 | -2.29758 |
| CUST_141950_PI430048170 | 1.28E-04 | -6.39273 | 4.646488 | -2.29213 |
| CUST_135570_PI430048170 | 6.71E-04 | -5.33396 | 2.337409 | -2.29138 |
| CUST_24533_PI430048170 | 6.86E-04 | -5.31913 | 2.304214 | -2.29128 |
| CUST_137828_PI430048170 | 3.87E-06 | -9.18793 | 10.01686 | -2.29122 |
| CUST_101115_PI430048170 | 7.40E-04 | -5.27349 | 2.201948 | -2.29115 |
| CUST_104408_PI430048170 | 1.32E-04 | -6.37097 | 4.600397 | -2.29017 |
| CUST_54740_PI430048170 | 2.84E-04 | -5.86712 | 3.51622 | -2.28854 |
| CUST_27583_PI430048170 | 3.13E-04 | -5.81058 | 3.392643 | -2.28843 |
| CUST_136720_PI430048170 | 1.95E-03 | -4.69698 | 0.897211 | -2.28714 |
| CUST_144174_PI430048170 | 3.63E-04 | -5.71776 | 3.189006 | -2.28595 |
| CUST_118843_PI430048170 | 6.84E-04 | -5.32161 | 2.309769 | -2.28527 |
| CUST_41541_PI430048170 | 4.61E-05 | -7.15451 | 6.21985 | -2.28502 |
| CUST_136507_PI430048170 | 3.62E-04 | -5.72279 | 3.200069 | -2.2844 |
| CUST_50239_PI430048170 | 2.62E-04 | -5.91614 | 3.623065 | -2.28155 |
| CUST_63460_PI430048170 | 3.07E-05 | -7.47385 | 6.855188 | -2.27828 |
| CUST_27585_PI430048170 | 5.13E-05 | -7.0728 | 6.054958 | -2.27773 |
| CUST_93845_PI430048170 | 3.79E-03 | -4.31031 | 0.014123 | -2.27757 |
| CUST_71781_PI430048170 | 6.46E-04 | -5.35695 | 2.388822 | -2.2766 |
| CUST_46637_PI430048170 | 1.18E-04 | -6.44867 | 4.764731 | -2.27602 |
| CUST_29078_PI430048170 | 3.11E-03 | -4.42075 | 0.26657 | -2.2752 |
| CUST_116897_PI430048170 | 1.19E-05 | -8.20609 | 8.256806 | -2.27376 |
| CUST_118845_PI430048170 | 9.81E-04 | -5.10391 | 1.820471 | -2.27148 |
| CUST_140794_PI430048170 | 5.24E-04 | -5.48749 | 2.679811 | -2.26788 |
| CUST_56144_PI430048170 | 5.68E-03 | -4.07821 | -0.51593 | -2.26715 |
| CUST_131496_PI430048170 | 1.11E-03 | -5.02767 | 1.64827 | -2.26685 |
| CUST_139865_PI430048170 | 5.68E-04 | -5.43902 | 2.571948 | -2.2667 |
| CUST_86625_PI430048170 | 1.39E-03 | -4.89466 | 1.346891 | -2.2656 |
| CUST_11495_PI430048170 | 3.36E-04 | -5.76836 | 3.300152 | -2.2651 |
| CUST_132115_PI430048170 | 8.85E-05 | -6.66642 | 5.220937 | -2.26444 |
| CUST_28313_PI430048170 | 7.03E-05 | -6.82841 | 5.556144 | -2.2642 |
| CUST_88177_PI430048170 | 1.77E-04 | -6.17484 | 4.182136 | -2.26341 |
| CUST_56114_PI430048170 | 8.47E-03 | -3.84934 | -1.03654 | -2.26191 |
| CUST_137742_PI430048170 | 4.86E-02 | -2.80417 | -3.32695 | -2.2615 |
| CUST_42318_PI430048170 | 2.18E-02 | -3.29342 | -2.27999 | -2.26107 |
| CUST_143922_PI430048170 | 2.48E-05 | -7.63682 | 7.173799 | -2.26037 |
| CUST_138369_PI430048170 | 6.92E-05 | -6.84164 | 5.583357 | -2.25782 |
| CUST_94062_PI430048170 | 6.16E-06 | -8.79254 | 9.324254 | -2.25528 |
| CUST_129790_PI430048170 | 5.30E-04 | -5.47974 | 2.662585 | -2.25488 |
| CUST_86466_PI430048170 | 7.01E-04 | -5.30423 | 2.270848 | -2.25198 |
| CUST_132534_PI430048170 | 4.59E-05 | -7.15785 | 6.226571 | -2.25167 |
| CUST_66112_PI430048170 | 6.66E-05 | -6.86564 | 5.632672 | -2.25153 |
| CUST_142773_PI430048170 | 1.03E-05 | -8.3284 | 8.483433 | -2.24597 |
| CUST_23299_PI430048170 | 1.10E-03 | -5.03553 | 1.666029 | -2.24578 |
| CUST_59148_PI430048170 | 3.33E-03 | -4.38448 | 0.183666 | -2.24519 |
| CUST_138656_PI430048170 | 7.94E-04 | -5.23161 | 2.107955 | -2.24273 |
| CUST_59182_PI430048170 | 8.96E-06 | -8.45412 | 8.714178 | -2.24213 |
| CUST_67340_PI430048170 | 2.73E-03 | -4.49832 | 0.443838 | -2.24075 |
| CUST_27314_PI430048170 | 4.78E-05 | -7.13142 | 6.173354 | -2.23999 |
| CUST_112036_PI430048170 | 5.59E-05 | -7.00798 | 5.923481 | -2.2388 |
| CUST_142949_PI430048170 | 3.64E-05 | -7.33076 | 6.572318 | -2.23733 |
| CUST_67938_PI430048170 | 1.39E-04 | -6.3337 | 4.521288 | -2.23723 |
| CUST_127306_PI430048170 | 6.23E-05 | -6.92159 | 5.747323 | -2.23645 |
| CUST_71680_PI430048170 | 6.80E-05 | -6.85292 | 5.606547 | -2.23263 |
| CUST_95407_PI430048170 | 9.14E-04 | -5.14554 | 1.914335 | -2.22812 |
| CUST_89565_PI430048170 | 6.95E-03 | -3.96165 | -0.78144 | -2.22731 |
| CUST_78792_PI430048170 | 3.72E-05 | -7.31443 | 6.539842 | -2.22397 |
| CUST_113442_PI430048170 | 1.93E-04 | -6.10788 | 4.038211 | -2.22393 |
| CUST_44907_PI430048170 | 2.01E-04 | -6.07835 | 3.974563 | -2.22174 |
| CUST_31088_PI430048170 | 1.34E-02 | -3.58073 | -1.64229 | -2.22153 |
| CUST_33837_PI430048170 | 9.65E-03 | -3.77434 | -1.20637 | -2.2205 |
| CUST_40009_PI430048170 | 1.86E-03 | -4.72655 | 0.96459 | -2.22044 |
| CUST_134871_PI430048170 | 5.52E-04 | -5.45742 | 2.612934 | -2.21916 |
| CUST_66893_PI430048170 | 1.11E-02 | -3.6929 | -1.39021 | -2.21814 |
| CUST_139080_PI430048170 | 8.07E-05 | -6.73046 | 5.353889 | -2.21756 |
| CUST_113867_PI430048170 | 1.31E-04 | -6.37709 | 4.61337 | -2.21644 |
| CUST_77414_PI430048170 | 1.13E-04 | -6.47935 | 4.829387 | -2.21544 |
| CUST_138521_PI430048170 | 8.16E-05 | -6.7242 | 5.340918 | -2.21457 |
| CUST_144898_PI430048170 | 4.93E-03 | -4.15899 | -0.3316 | -2.21358 |
| CUST_145548_PI430048170 | 8.87E-04 | -5.16333 | 1.954404 | -2.21318 |
| CUST_49533_PI430048170 | 4.38E-03 | -4.2282 | -0.17351 | -2.21179 |
| CUST_131084_PI430048170 | 9.27E-04 | -5.13686 | 1.89477 | -2.20963 |
| CUST_144873_PI430048170 | 2.45E-03 | -4.56241 | 0.590213 | -2.20927 |
| CUST_103055_PI430048170 | 1.14E-03 | -5.01142 | 1.611492 | -2.2073 |
| CUST_53139_PI430048170 | 1.44E-03 | -4.87491 | 1.302058 | -2.2067 |
| CUST_73677_PI430048170 | 4.78E-04 | -5.54697 | 2.811865 | -2.2062 |
| CUST_24728_PI430048170 | 3.68E-04 | -5.706 | 3.163131 | -2.20419 |
| CUST_86666_PI430048170 | 9.90E-03 | -3.75839 | -1.24242 | -2.20357 |
| CUST_123142_PI430048170 | 6.32E-05 | -6.90692 | 5.717288 | -2.20278 |
| CUST_134063_PI430048170 | 3.23E-02 | -3.05958 | -2.78779 | -2.20237 |
| CUST_80850_PI430048170 | 2.35E-05 | -7.68764 | 7.272381 | -2.2023 |
| CUST_117592_PI430048170 | 1.37E-04 | -6.34382 | 4.542786 | -2.2003 |
| CUST_32904_PI430048170 | 5.22E-05 | -7.05771 | 6.024409 | -2.19755 |
| CUST_132182_PI430048170 | 4.67E-04 | -5.56097 | 2.842893 | -2.19661 |
| CUST_126347_PI430048170 | 4.28E-02 | -2.88653 | -3.1551 | -2.19481 |
| CUST_22289_PI430048170 | 1.11E-04 | -6.49578 | 4.863973 | -2.19397 |
| CUST_65838_PI430048170 | 2.22E-04 | -6.0144 | 3.836362 | -2.19175 |
| CUST_125692_PI430048170 | 2.84E-04 | -5.86763 | 3.517338 | -2.1901 |
| CUST_93414_PI430048170 | 4.97E-03 | -4.15344 | -0.34427 | -2.18935 |
| CUST_100533_PI430048170 | 1.87E-02 | -3.38456 | -2.07913 | -2.18675 |
| CUST_16262_PI430048170 | 8.65E-07 | -10.5114 | 12.1839 | -2.18445 |
| CUST_31718_PI430048170 | 3.32E-03 | -4.38606 | 0.187289 | -2.18002 |
| CUST_11956_PI430048170 | 2.56E-04 | -5.93098 | 3.655344 | -2.17996 |
| CUST_137355_PI430048170 | 1.06E-02 | -3.72115 | -1.32652 | -2.1785 |
| CUST_27316_PI430048170 | 5.86E-05 | -6.97538 | 5.85712 | -2.17689 |
| CUST_24873_PI430048170 | 3.58E-04 | -5.72896 | 3.213637 | -2.17616 |
| CUST_139045_PI430048170 | 5.57E-04 | -5.45257 | 2.602128 | -2.17558 |
| CUST_47271_PI430048170 | 4.97E-03 | -4.15403 | -0.34291 | -2.17446 |
| CUST_42970_PI430048170 | 3.90E-02 | -2.94408 | -3.03384 | -2.17308 |
| CUST_138023_PI430048170 | 1.43E-02 | -3.54229 | -1.72832 | -2.17296 |
| CUST_145305_PI430048170 | 2.09E-03 | -4.65458 | 0.800557 | -2.17266 |
| CUST_138852_PI430048170 | 1.46E-06 | -10.0189 | 11.40387 | -2.17192 |
| CUST_68117_PI430048170 | 4.94E-04 | -5.52382 | 2.760518 | -2.17175 |
| CUST_138906_PI430048170 | 3.15E-05 | -7.45525 | 6.818594 | -2.17136 |
| CUST_123351_PI430048170 | 2.43E-04 | -5.96362 | 3.726275 | -2.17117 |
| CUST_32923_PI430048170 | 2.48E-04 | -5.94994 | 3.696563 | -2.17094 |
| CUST_19086_PI430048170 | 2.74E-07 | -11.6674 | 13.90011 | -2.16749 |
| CUST_142041_PI430048170 | 1.22E-02 | -3.63563 | -1.51909 | -2.16698 |
| CUST_135355_PI430048170 | 3.14E-05 | -7.45801 | 6.824013 | -2.16597 |
| CUST_137206_PI430048170 | 3.38E-03 | -4.37543 | 0.162983 | -2.16395 |
| CUST_34029_PI430048170 | 2.34E-04 | -5.98472 | 3.772068 | -2.16351 |
| CUST_77209_PI430048170 | 2.48E-03 | -4.55342 | 0.569682 | -2.1631 |
| CUST_46046_PI430048170 | 5.70E-04 | -5.4368 | 2.567001 | -2.16104 |
| CUST_55798_PI430048170 | 6.26E-05 | -6.91656 | 5.737017 | -2.15609 |
| CUST_73783_PI430048170 | 1.78E-02 | -3.41537 | -2.01091 | -2.15598 |
| CUST_119126_PI430048170 | 4.44E-02 | -2.86194 | -3.20663 | -2.15492 |
| CUST_47914_PI430048170 | 2.36E-05 | -7.6818 | 7.261071 | -2.15472 |
| CUST_143676_PI430048170 | 6.33E-03 | -4.01706 | -0.6553 | -2.15437 |
| CUST_64736_PI430048170 | 5.07E-05 | -7.0808 | 6.071151 | -2.1503 |
| CUST_76440_PI430048170 | 2.24E-03 | -4.614 | 0.707976 | -2.15017 |
| CUST_137460_PI430048170 | 1.52E-03 | -4.84257 | 1.228583 | -2.14881 |
| CUST_143531_PI430048170 | 3.99E-05 | -7.26379 | 6.438907 | -2.14678 |
| CUST_83988_PI430048170 | 1.11E-04 | -6.49563 | 4.863657 | -2.14663 |
| CUST_37360_PI430048170 | 9.17E-04 | -5.14266 | 1.907847 | -2.14594 |
| CUST_88748_PI430048170 | 3.02E-03 | -4.43965 | 0.309769 | -2.14547 |
| CUST_140533_PI430048170 | 3.76E-04 | -5.6943 | 3.137387 | -2.14506 |
| CUST_126811_PI430048170 | 1.97E-04 | -6.09238 | 4.004815 | -2.14362 |
| CUST_132907_PI430048170 | 3.65E-05 | -7.32689 | 6.56462 | -2.14315 |
| CUST_65771_PI430048170 | 1.82E-04 | -6.15631 | 4.142352 | -2.14251 |
| CUST_83196_PI430048170 | 1.24E-03 | -4.96459 | 1.505485 | -2.13895 |
| CUST_63285_PI430048170 | 4.57E-02 | -2.84312 | -3.24594 | -2.13822 |
| CUST_75161_PI430048170 | 6.30E-03 | -4.0199 | -0.64882 | -2.13801 |
| CUST_58629_PI430048170 | 1.15E-03 | -5.01052 | 1.609458 | -2.13779 |
| CUST_142854_PI430048170 | 1.83E-02 | -3.39764 | -2.05018 | -2.13712 |
| CUST_137547_PI430048170 | 2.33E-02 | -3.25413 | -2.36612 | -2.13706 |
| CUST_56642_PI430048170 | 9.88E-04 | -5.09912 | 1.809665 | -2.13686 |
| CUST_55796_PI430048170 | 3.62E-04 | -5.72026 | 3.194511 | -2.1367 |
| CUST_47655_PI430048170 | 4.24E-05 | -7.22167 | 6.354675 | -2.13584 |
| CUST_93695_PI430048170 | 3.44E-05 | -7.38314 | 6.676202 | -2.13483 |
| CUST_133820_PI430048170 | 8.46E-03 | -3.84989 | -1.0353 | -2.13247 |
| CUST_40458_PI430048170 | 4.96E-05 | -7.09868 | 6.10728 | -2.1323 |
| CUST_63559_PI430048170 | 1.80E-04 | -6.16417 | 4.15923 | -2.13176 |
| CUST_18771_PI430048170 | 2.49E-05 | -7.63246 | 7.165318 | -2.13171 |
| CUST_104910_PI430048170 | 7.21E-04 | -5.28704 | 2.232319 | -2.13093 |
| CUST_73279_PI430048170 | 6.92E-06 | -8.70233 | 9.16319 | -2.13072 |
| CUST_132051_PI430048170 | 7.27E-07 | -10.682 | 12.44719 | -2.13041 |
| CUST_134892_PI430048170 | 9.98E-07 | -10.3727 | 11.96733 | -2.12815 |
| CUST_121091_PI430048170 | 5.64E-05 | -6.99964 | 5.906511 | -2.12694 |
| CUST_118378_PI430048170 | 1.91E-05 | -7.84779 | 7.580586 | -2.1268 |
| CUST_18202_PI430048170 | 2.51E-03 | -4.54728 | 0.555654 | -2.12362 |
| CUST_129092_PI430048170 | 1.30E-04 | -6.3817 | 4.623147 | -2.12318 |
| CUST_124891_PI430048170 | 2.04E-03 | -4.66951 | 0.834604 | -2.12227 |
| CUST_120170_PI430048170 | 2.85E-04 | -5.86387 | 3.509122 | -2.12215 |
| CUST_128263_PI430048170 | 1.83E-03 | -4.73709 | 0.988604 | -2.12087 |
| CUST_54132_PI430048170 | 5.50E-04 | -5.46024 | 2.619196 | -2.11938 |
| CUST_65919_PI430048170 | 1.35E-03 | -4.91452 | 1.391956 | -2.11928 |
| CUST_136185_PI430048170 | 1.72E-05 | -7.92592 | 7.729601 | -2.11867 |
| CUST_32517_PI430048170 | 2.91E-04 | -5.85136 | 3.481825 | -2.1185 |
| CUST_131503_PI430048170 | 1.56E-04 | -6.25549 | 4.354724 | -2.11708 |
| CUST_138496_PI430048170 | 1.96E-03 | -4.69463 | 0.891859 | -2.11645 |
| CUST_95949_PI430048170 | 4.97E-04 | -5.51985 | 2.75171 | -2.1163 |
| CUST_78158_PI430048170 | 1.60E-04 | -6.23886 | 4.319206 | -2.11515 |
| CUST_50119_PI430048170 | 1.54E-04 | -6.26648 | 4.378185 | -2.11208 |
| CUST_95214_PI430048170 | 4.63E-04 | -5.56716 | 2.856605 | -2.11161 |
| CUST_125755_PI430048170 | 1.32E-03 | -4.9261 | 1.418234 | -2.11118 |
| CUST_57235_PI430048170 | 2.65E-05 | -7.57752 | 7.0583 | -2.1103 |
| CUST_24438_PI430048170 | 6.40E-05 | -6.89682 | 5.696611 | -2.10936 |
| CUST_91858_PI430048170 | 7.72E-03 | -3.90222 | -0.91652 | -2.10717 |
| CUST_125492_PI430048170 | 7.27E-04 | -5.28242 | 2.221968 | -2.10561 |
| CUST_14646_PI430048170 | 5.72E-03 | -4.0741 | -0.52529 | -2.10209 |
| CUST_120367_PI430048170 | 1.02E-03 | -5.07925 | 1.764817 | -2.10157 |
| CUST_120596_PI430048170 | 1.07E-06 | -10.3018 | 11.85567 | -2.10138 |
| CUST_7710_PI430048170 | 3.43E-04 | -5.7558 | 3.272579 | -2.0971 |
| CUST_135724_PI430048170 | 3.04E-03 | -4.4352 | 0.299593 | -2.09585 |
| CUST_75101_PI430048170 | 8.42E-04 | -5.195 | 2.025667 | -2.09529 |
| CUST_82958_PI430048170 | 1.11E-04 | -6.4969 | 4.866327 | -2.09521 |
| CUST_107203_PI430048170 | 5.86E-05 | -6.9757 | 5.857778 | -2.09454 |
| CUST_130937_PI430048170 | 2.56E-05 | -7.60487 | 7.111637 | -2.09447 |
| CUST_29856_PI430048170 | 1.71E-04 | -6.19802 | 4.231826 | -2.0924 |
| CUST_119564_PI430048170 | 1.20E-03 | -4.98227 | 1.545524 | -2.09229 |
| CUST_100136_PI430048170 | 1.72E-03 | -4.77164 | 1.067259 | -2.08925 |
| CUST_61083_PI430048170 | 5.10E-03 | -4.13888 | -0.37751 | -2.08774 |
| CUST_129708_PI430048170 | 4.66E-05 | -7.14795 | 6.206656 | -2.08751 |
| CUST_127039_PI430048170 | 1.45E-04 | -6.30438 | 4.458935 | -2.08669 |
| CUST_105590_PI430048170 | 1.15E-03 | -5.00973 | 1.607667 | -2.08651 |
| CUST_61648_PI430048170 | 1.83E-03 | -4.73562 | 0.985258 | -2.08541 |
| CUST_64091_PI430048170 | 3.93E-05 | -7.27677 | 6.464805 | -2.08484 |
| CUST_66111_PI430048170 | 3.04E-04 | -5.82609 | 3.426582 | -2.08265 |
| CUST_35792_PI430048170 | 4.87E-04 | -5.53443 | 2.784061 | -2.08225 |
| CUST_92940_PI430048170 | 4.44E-03 | -4.2193 | -0.19385 | -2.08151 |
| CUST_49501_PI430048170 | 7.52E-03 | -3.91744 | -0.88194 | -2.08144 |
| CUST_126340_PI430048170 | 1.14E-04 | -6.46996 | 4.8096 | -2.07967 |
| CUST_31863_PI430048170 | 4.97E-04 | -5.51932 | 2.750524 | -2.07838 |
| CUST_63760_PI430048170 | 1.56E-04 | -6.25562 | 4.355 | -2.07836 |
| CUST_14064_PI430048170 | 1.97E-04 | -6.09137 | 4.002631 | -2.0772 |
| CUST_142832_PI430048170 | 3.07E-05 | -7.47573 | 6.858894 | -2.07342 |
| CUST_125684_PI430048170 | 8.65E-04 | -5.17901 | 1.989682 | -2.07304 |
| CUST_7725_PI430048170 | 4.67E-04 | -5.56252 | 2.84633 | -2.0727 |
| CUST_88254_PI430048170 | 8.51E-04 | -5.18879 | 2.011693 | -2.07222 |
| CUST_69989_PI430048170 | 1.49E-03 | -4.85415 | 1.254911 | -2.06814 |
| CUST_74581_PI430048170 | 9.43E-04 | -5.12768 | 1.87408 | -2.06786 |
| CUST_18077_PI430048170 | 7.23E-03 | -3.93893 | -0.83311 | -2.06715 |
| CUST_73528_PI430048170 | 1.61E-03 | -4.81208 | 1.159278 | -2.06653 |
| CUST_36969_PI430048170 | 3.15E-06 | -9.37359 | 10.33472 | -2.06482 |
| CUST_93398_PI430048170 | 8.09E-06 | -8.57684 | 8.937265 | -2.06388 |
| CUST_343_PI430048170 | 2.66E-04 | -5.90587 | 3.600695 | -2.06176 |
| CUST_111435_PI430048170 | 9.71E-05 | -6.59599 | 5.074076 | -2.06061 |
| CUST_104648_PI430048170 | 8.77E-04 | -5.16999 | 1.969402 | -2.06009 |
| CUST_55275_PI430048170 | 1.06E-02 | -3.72064 | -1.32767 | -2.05978 |
| CUST_71571_PI430048170 | 8.32E-04 | -5.20196 | 2.041325 | -2.05849 |
| CUST_47249_PI430048170 | 2.92E-03 | -4.45779 | 0.351213 | -2.05787 |
| CUST_117441_PI430048170 | 1.25E-03 | -4.95936 | 1.493621 | -2.05745 |
| CUST_111071_PI430048170 | 9.03E-06 | -8.4317 | 8.673203 | -2.05732 |
| CUST_9690_PI430048170 | 2.84E-03 | -4.4746 | 0.389636 | -2.05688 |
| CUST_139864_PI430048170 | 3.79E-04 | -5.6889 | 3.125487 | -2.05638 |
| CUST_75031_PI430048170 | 8.97E-05 | -6.65588 | 5.198997 | -2.05515 |
| CUST_64737_PI430048170 | 3.42E-05 | -7.38615 | 6.682156 | -2.05492 |
| CUST_7727_PI430048170 | 6.44E-04 | -5.35917 | 2.393773 | -2.05402 |
| CUST_68256_PI430048170 | 5.64E-04 | -5.4436 | 2.58216 | -2.05281 |
| CUST_68236_PI430048170 | 3.13E-04 | -5.80898 | 3.38916 | -2.05262 |
| CUST_144815_PI430048170 | 3.86E-03 | -4.29836 | -0.01317 | -2.05242 |
| CUST_7692_PI430048170 | 5.48E-04 | -5.46237 | 2.623939 | -2.05153 |
| CUST_75164_PI430048170 | 1.10E-02 | -3.6993 | -1.37579 | -2.05084 |
| CUST_7694_PI430048170 | 6.20E-04 | -5.38197 | 2.444714 | -2.04983 |
| CUST_70168_PI430048170 | 2.98E-03 | -4.44644 | 0.325289 | -2.04814 |
| CUST_119171_PI430048170 | 2.01E-04 | -6.07654 | 3.970663 | -2.04801 |
| CUST_52761_PI430048170 | 8.68E-04 | -5.17683 | 1.984797 | -2.04722 |
| CUST_71679_PI430048170 | 5.22E-06 | -8.92745 | 9.562986 | -2.04667 |
| CUST_50883_PI430048170 | 9.80E-03 | -3.76556 | -1.22621 | -2.04634 |
| CUST_35979_PI430048170 | 1.78E-04 | -6.17116 | 4.174237 | -2.04534 |
| CUST_106617_PI430048170 | 1.16E-03 | -5.00454 | 1.595922 | -2.04474 |
| CUST_124893_PI430048170 | 2.21E-02 | -3.28573 | -2.29688 | -2.04183 |
| CUST_7684_PI430048170 | 6.19E-04 | -5.38341 | 2.447923 | -2.0398 |
| CUST_44798_PI430048170 | 1.71E-03 | -4.77331 | 1.07106 | -2.03937 |
| CUST_75699_PI430048170 | 3.50E-05 | -7.36637 | 6.642982 | -2.03908 |
| CUST_35407_PI430048170 | 5.07E-04 | -5.50656 | 2.722204 | -2.03855 |
| CUST_58794_PI430048170 | 6.63E-04 | -5.34161 | 2.35451 | -2.03796 |
| CUST_55388_PI430048170 | 1.85E-04 | -6.14295 | 4.113649 | -2.03609 |
| CUST_35711_PI430048170 | 4.14E-04 | -5.63302 | 3.002268 | -2.03456 |
| CUST_113672_PI430048170 | 6.94E-04 | -5.31146 | 2.287041 | -2.03432 |
| CUST_145437_PI430048170 | 1.49E-03 | -4.85481 | 1.256411 | -2.0333 |
| CUST_137541_PI430048170 | 1.80E-04 | -6.16379 | 4.158423 | -2.03023 |
| CUST_131852_PI430048170 | 4.26E-03 | -4.24363 | -0.13825 | -2.02995 |
| CUST_134357_PI430048170 | 2.08E-04 | -6.05708 | 3.928654 | -2.02961 |
| CUST_137658_PI430048170 | 3.99E-02 | -2.93012 | -3.06335 | -2.0289 |
| CUST_97698_PI430048170 | 3.78E-03 | -4.31264 | 0.019451 | -2.02848 |
| CUST_103499_PI430048170 | 2.42E-02 | -3.23032 | -2.41815 | -2.02719 |
| CUST_40661_PI430048170 | 9.87E-03 | -3.7612 | -1.23607 | -2.02679 |
| CUST_134186_PI430048170 | 1.60E-04 | -6.24104 | 4.323864 | -2.02636 |
| CUST_141933_PI430048170 | 9.91E-04 | -5.09783 | 1.806758 | -2.02571 |
| CUST_44825_PI430048170 | 4.26E-02 | -2.88808 | -3.15186 | -2.02363 |
| CUST_28811_PI430048170 | 6.44E-04 | -5.35855 | 2.392385 | -2.02345 |
| CUST_142227_PI430048170 | 4.42E-04 | -5.5939 | 2.915815 | -2.02221 |
| CUST_135835_PI430048170 | 9.12E-04 | -5.14716 | 1.91798 | -2.02016 |
| CUST_103719_PI430048170 | 9.89E-03 | -3.75998 | -1.23883 | -2.01999 |
| CUST_112352_PI430048170 | 1.68E-03 | -4.78273 | 1.092513 | -2.01756 |
| CUST_136605_PI430048170 | 2.05E-05 | -7.79712 | 7.483473 | -2.01727 |
| CUST_108505_PI430048170 | 3.34E-03 | -4.38145 | 0.176732 | -2.01708 |
| CUST_115803_PI430048170 | 9.22E-05 | -6.63285 | 5.151027 | -2.01703 |
| CUST_20369_PI430048170 | 2.04E-02 | -3.33319 | -2.19252 | -2.01592 |
| CUST_24244_PI430048170 | 5.08E-04 | -5.50475 | 2.718164 | -2.01551 |
| CUST_120368_PI430048170 | 6.40E-04 | -5.36241 | 2.401013 | -2.01547 |
| CUST_86640_PI430048170 | 1.16E-04 | -6.46167 | 4.792145 | -2.0111 |
| CUST_79431_PI430048170 | 4.56E-03 | -4.20396 | -0.22889 | -2.01094 |
| CUST_136620_PI430048170 | 1.67E-05 | -7.94634 | 7.768409 | -2.0096 |
| CUST_142192_PI430048170 | 9.40E-05 | -6.61957 | 5.12332 | -2.00878 |
| CUST_24258_PI430048170 | 4.18E-04 | -5.62739 | 2.989838 | -2.00825 |
| CUST_142033_PI430048170 | 1.90E-08 | -14.7124 | 17.75129 | -2.00816 |
| CUST_12669_PI430048170 | 4.83E-03 | -4.17217 | -0.3015 | -2.0081 |
| CUST_7686_PI430048170 | 7.02E-04 | -5.30298 | 2.268056 | -2.00782 |
| CUST_37749_PI430048170 | 2.40E-03 | -4.57272 | 0.613765 | -2.0076 |
| CUST_61592_PI430048170 | 6.02E-04 | -5.39948 | 2.483794 | -2.00694 |
| CUST_51641_PI430048170 | 1.63E-04 | -6.22869 | 4.297454 | -2.00512 |
| CUST_143106_PI430048170 | 4.11E-02 | -2.91118 | -3.10328 | -2.00321 |
| CUST_130636_PI430048170 | 3.88E-04 | -5.67408 | 3.092855 | -2.00278 |
| CUST_89946_PI430048170 | 9.29E-04 | -5.13536 | 1.891387 | -2.00258 |
| CUST_119870_PI430048170 | 1.36E-02 | -3.5761 | -1.65265 | -2.00066 |
| CUST_83137_PI430048170 | 1.48E-03 | -4.85828 | 1.264289 | -2.00033 |
| CUST_122357_PI430048170 | 1.25E-03 | -4.95639 | 1.486898 | -2.00023 |
| CUST_69828_PI430048170 | 6.03E-04 | -5.39917 | 2.483115 | -1.9967 |
| CUST_14738_PI430048170 | 7.83E-05 | -6.75099 | 5.3964 | -1.99527 |
| CUST_133936_PI430048170 | 5.90E-03 | -4.05671 | -0.56494 | -1.99381 |
| CUST_136390_PI430048170 | 1.89E-04 | -6.12183 | 4.068237 | -1.99367 |
| CUST_111098_PI430048170 | 5.97E-04 | -5.40556 | 2.497365 | -1.99246 |
| CUST_129942_PI430048170 | 3.84E-03 | -4.30181 | -0.00529 | -1.99083 |
| CUST_138154_PI430048170 | 2.28E-03 | -4.60419 | 0.685593 | -1.98775 |
| CUST_27582_PI430048170 | 6.27E-04 | -5.37582 | 2.430972 | -1.98653 |
| CUST_7681_PI430048170 | 6.58E-04 | -5.34622 | 2.364826 | -1.98651 |
| CUST_58729_PI430048170 | 1.36E-03 | -4.90925 | 1.379994 | -1.9862 |
| CUST_33831_PI430048170 | 1.36E-02 | -3.57338 | -1.65876 | -1.98603 |
| CUST_95016_PI430048170 | 2.65E-03 | -4.51323 | 0.477888 | -1.98578 |
| CUST_86940_PI430048170 | 3.53E-03 | -4.35112 | 0.107408 | -1.98476 |
| CUST_75420_PI430048170 | 9.60E-04 | -5.11716 | 1.850351 | -1.98431 |
| CUST_94171_PI430048170 | 4.56E-03 | -4.20395 | -0.2289 | -1.9825 |
| CUST_134872_PI430048170 | 6.75E-04 | -5.32896 | 2.326205 | -1.98248 |
| CUST_89221_PI430048170 | 3.75E-04 | -5.69703 | 3.143398 | -1.98233 |
| CUST_142765_PI430048170 | 2.22E-03 | -4.61843 | 0.718079 | -1.9809 |
| CUST_115429_PI430048170 | 1.76E-04 | -6.17785 | 4.188574 | -1.98049 |
| CUST_24499_PI430048170 | 3.31E-03 | -4.38743 | 0.190401 | -1.98013 |
| CUST_94377_PI430048170 | 4.45E-02 | -2.86108 | -3.20843 | -1.97635 |
| CUST_50938_PI430048170 | 3.04E-04 | -5.82625 | 3.426935 | -1.97635 |
| CUST_107792_PI430048170 | 8.38E-03 | -3.85524 | -1.02315 | -1.97592 |
| CUST_10434_PI430048170 | 5.98E-05 | -6.95966 | 5.825077 | -1.97583 |
| CUST_63512_PI430048170 | 2.07E-04 | -6.06048 | 3.935985 | -1.97548 |
| CUST_95570_PI430048170 | 8.02E-04 | -5.22546 | 2.09413 | -1.97474 |
| CUST_134754_PI430048170 | 1.72E-03 | -4.76994 | 1.063399 | -1.97412 |
| CUST_63714_PI430048170 | 6.02E-03 | -4.04589 | -0.5896 | -1.97331 |
| CUST_11090_PI430048170 | 2.12E-03 | -4.64642 | 0.781932 | -1.97268 |
| CUST_58196_PI430048170 | 1.65E-03 | -4.7969 | 1.124752 | -1.9717 |
| CUST_136848_PI430048170 | 3.01E-05 | -7.49018 | 6.887287 | -1.97067 |
| CUST_55034_PI430048170 | 1.53E-04 | -6.27197 | 4.389887 | -1.97028 |
| CUST_69502_PI430048170 | 1.59E-04 | -6.2427 | 4.3274 | -1.97005 |
| CUST_96577_PI430048170 | 7.17E-04 | -5.29042 | 2.2399 | -1.96893 |
| CUST_145094_PI430048170 | 1.75E-02 | -3.42446 | -1.99075 | -1.96892 |
| CUST_137633_PI430048170 | 1.11E-04 | -6.49613 | 4.864694 | -1.96857 |
| CUST_137348_PI430048170 | 8.97E-05 | -6.65702 | 5.201381 | -1.96631 |
| CUST_10435_PI430048170 | 2.50E-05 | -7.62665 | 7.154035 | -1.96473 |
| CUST_127216_PI430048170 | 2.68E-05 | -7.56886 | 7.041399 | -1.96404 |
| CUST_102721_PI430048170 | 1.30E-02 | -3.59798 | -1.60362 | -1.96188 |
| CUST_133189_PI430048170 | 4.51E-06 | -9.05353 | 9.783841 | -1.96122 |
| CUST_48076_PI430048170 | 6.06E-04 | -5.39548 | 2.474864 | -1.96054 |
| CUST_129008_PI430048170 | 4.37E-04 | -5.60091 | 2.931307 | -1.95952 |
| CUST_63741_PI430048170 | 6.76E-04 | -5.32784 | 2.323702 | -1.95902 |
| CUST_130790_PI430048170 | 3.02E-02 | -3.09912 | -2.70278 | -1.95595 |
| CUST_133094_PI430048170 | 2.72E-03 | -4.4987 | 0.444693 | -1.95572 |
| CUST_133283_PI430048170 | 1.49E-03 | -4.85569 | 1.258391 | -1.95557 |
| CUST_37650_PI430048170 | 1.39E-04 | -6.3353 | 4.524701 | -1.95433 |
| CUST_135193_PI430048170 | 1.05E-03 | -5.06438 | 1.73124 | -1.95364 |
| CUST_12481_PI430048170 | 5.05E-04 | -5.5102 | 2.730268 | -1.9536 |
| CUST_35975_PI430048170 | 6.89E-04 | -5.31633 | 2.297945 | -1.95331 |
| CUST_46710_PI430048170 | 2.10E-03 | -4.65138 | 0.79325 | -1.95289 |
| CUST_121520_PI430048170 | 5.60E-04 | -5.44813 | 2.592249 | -1.95251 |
| CUST_121518_PI430048170 | 5.62E-03 | -4.08369 | -0.50343 | -1.95211 |
| CUST_138722_PI430048170 | 3.50E-05 | -7.37013 | 6.65043 | -1.94677 |
| CUST_73637_PI430048170 | 3.40E-05 | -7.39277 | 6.69527 | -1.94629 |
| CUST_133510_PI430048170 | 8.90E-04 | -5.16082 | 1.948737 | -1.94607 |
| CUST_13636_PI430048170 | 2.33E-04 | -5.98544 | 3.773625 | -1.94593 |
| CUST_125805_PI430048170 | 2.11E-03 | -4.64907 | 0.78799 | -1.94564 |
| CUST_134530_PI430048170 | 4.88E-05 | -7.11136 | 6.132892 | -1.94546 |
| CUST_142009_PI430048170 | 1.26E-03 | -4.95487 | 1.483443 | -1.94529 |
| CUST_79776_PI430048170 | 4.22E-04 | -5.6218 | 2.977489 | -1.94376 |
| CUST_23271_PI430048170 | 4.52E-04 | -5.58109 | 2.88746 | -1.94373 |
| CUST_134330_PI430048170 | 2.04E-05 | -7.80255 | 7.493884 | -1.94292 |
| CUST_25219_PI430048170 | 1.72E-05 | -7.92184 | 7.721849 | -1.94265 |
| CUST_16599_PI430048170 | 2.57E-02 | -3.19455 | -2.49612 | -1.94203 |
| CUST_132716_PI430048170 | 1.56E-03 | -4.82784 | 1.195106 | -1.94128 |
| CUST_143146_PI430048170 | 9.17E-04 | -5.14248 | 1.907434 | -1.94112 |
| CUST_132380_PI430048170 | 6.04E-04 | -5.39773 | 2.479903 | -1.94004 |
| CUST_125170_PI430048170 | 1.51E-03 | -4.84719 | 1.239094 | -1.93903 |
| CUST_143053_PI430048170 | 1.14E-03 | -5.01526 | 1.620199 | -1.93862 |
| CUST_89776_PI430048170 | 8.87E-06 | -8.46103 | 8.726798 | -1.93805 |
| CUST_100383_PI430048170 | 6.36E-04 | -5.36596 | 2.40895 | -1.93679 |
| CUST_93998_PI430048170 | 1.86E-04 | -6.13321 | 4.092707 | -1.93627 |
| CUST_77424_PI430048170 | 5.47E-04 | -5.46361 | 2.626713 | -1.9355 |
| CUST_103450_PI430048170 | 7.43E-04 | -5.27047 | 2.19517 | -1.93536 |
| CUST_139119_PI430048170 | 4.33E-02 | -2.87857 | -3.17181 | -1.93298 |
| CUST_45710_PI430048170 | 2.73E-05 | -7.55473 | 7.013798 | -1.93287 |
| CUST_31591_PI430048170 | 1.42E-04 | -6.31964 | 4.491412 | -1.93184 |
| CUST_129020_PI430048170 | 4.03E-04 | -5.6486 | 3.036658 | -1.93138 |
| CUST_17464_PI430048170 | 8.19E-04 | -5.2117 | 2.063208 | -1.92989 |
| CUST_123517_PI430048170 | 9.90E-05 | -6.57857 | 5.03764 | -1.92895 |
| CUST_62092_PI430048170 | 2.70E-02 | -3.16681 | -2.55641 | -1.92797 |
| CUST_133953_PI430048170 | 1.08E-03 | -5.04369 | 1.684489 | -1.92767 |
| CUST_20738_PI430048170 | 4.04E-04 | -5.6463 | 3.031575 | -1.92765 |
| CUST_135156_PI430048170 | 3.35E-05 | -7.40905 | 6.727448 | -1.92752 |
| CUST_99485_PI430048170 | 4.45E-03 | -4.21842 | -0.19587 | -1.92694 |
| CUST_129574_PI430048170 | 9.64E-04 | -5.11428 | 1.843866 | -1.9266 |
| CUST_121947_PI430048170 | 3.72E-04 | -5.70027 | 3.15052 | -1.9266 |
| CUST_143623_PI430048170 | 4.26E-04 | -5.61697 | 2.96681 | -1.92644 |
| CUST_11330_PI430048170 | 4.19E-03 | -4.25334 | -0.11608 | -1.92571 |
| CUST_55627_PI430048170 | 9.66E-05 | -6.59954 | 5.081496 | -1.9254 |
| CUST_120597_PI430048170 | 6.17E-05 | -6.93537 | 5.775485 | -1.92482 |
| CUST_46033_PI430048170 | 3.38E-04 | -5.76335 | 3.289142 | -1.9242 |
| CUST_130786_PI430048170 | 2.60E-02 | -3.18746 | -2.51155 | -1.92416 |
| CUST_103373_PI430048170 | 1.19E-03 | -4.98548 | 1.552782 | -1.92374 |
| CUST_145747_PI430048170 | 1.42E-04 | -6.32075 | 4.493769 | -1.92366 |
| CUST_49112_PI430048170 | 1.16E-03 | -5.00454 | 1.595936 | -1.92298 |
| CUST_25425_PI430048170 | 4.30E-05 | -7.21093 | 6.333151 | -1.92289 |
| CUST_137028_PI430048170 | 2.41E-04 | -5.96769 | 3.735111 | -1.92006 |
| CUST_5968_PI430048170 | 3.13E-03 | -4.4184 | 0.261197 | -1.91963 |
| CUST_128711_PI430048170 | 5.63E-05 | -7.00184 | 5.910978 | -1.91959 |
| CUST_24673_PI430048170 | 1.48E-04 | -6.29068 | 4.429768 | -1.91879 |
| CUST_55545_PI430048170 | 4.98E-03 | -4.15217 | -0.34717 | -1.91846 |
| CUST_90706_PI430048170 | 1.61E-04 | -6.23731 | 4.315887 | -1.91845 |
| CUST_94975_PI430048170 | 1.27E-05 | -8.15645 | 8.164207 | -1.91682 |
| CUST_88708_PI430048170 | 4.46E-03 | -4.2164 | -0.20046 | -1.91671 |
| CUST_139485_PI430048170 | 5.70E-05 | -6.99258 | 5.892156 | -1.91657 |
| CUST_139167_PI430048170 | 1.36E-03 | -4.90973 | 1.381093 | -1.91552 |
| CUST_113621_PI430048170 | 1.48E-03 | -4.85795 | 1.263542 | -1.91386 |
| CUST_43271_PI430048170 | 1.77E-05 | -7.8961 | 7.672838 | -1.91347 |
| CUST_120364_PI430048170 | 1.85E-04 | -6.13875 | 4.104617 | -1.91237 |
| CUST_134975_PI430048170 | 6.94E-03 | -3.96228 | -0.78001 | -1.91223 |
| CUST_141360_PI430048170 | 1.89E-04 | -6.12453 | 4.074056 | -1.91216 |
| CUST_130836_PI430048170 | 5.39E-05 | -7.0325 | 5.97328 | -1.91144 |
| CUST_127118_PI430048170 | 7.21E-03 | -3.94074 | -0.82898 | -1.91034 |
| CUST_110164_PI430048170 | 2.12E-03 | -4.64692 | 0.783089 | -1.90846 |
| CUST_97050_PI430048170 | 1.39E-03 | -4.89543 | 1.348635 | -1.90799 |
| CUST_62460_PI430048170 | 6.73E-04 | -5.332 | 2.333011 | -1.9073 |
| CUST_121932_PI430048170 | 4.06E-02 | -2.91938 | -3.08602 | -1.90642 |
| CUST_140891_PI430048170 | 3.14E-02 | -3.0763 | -2.75187 | -1.90605 |
| CUST_141328_PI430048170 | 6.34E-04 | -5.36809 | 2.413707 | -1.90435 |
| CUST_32518_PI430048170 | 3.25E-04 | -5.78768 | 3.342497 | -1.90397 |
| CUST_70513_PI430048170 | 4.22E-02 | -2.89486 | -3.13761 | -1.90293 |
| CUST_102374_PI430048170 | 3.91E-03 | -4.29244 | -0.02672 | -1.90213 |
| CUST_12842_PI430048170 | 1.42E-04 | -6.32223 | 4.496925 | -1.90068 |
| CUST_145658_PI430048170 | 9.51E-04 | -5.12277 | 1.863008 | -1.90051 |
| CUST_108758_PI430048170 | 1.59E-04 | -6.24263 | 4.327251 | -1.90007 |
| CUST_96663_PI430048170 | 5.51E-04 | -5.45848 | 2.615282 | -1.89891 |
| CUST_140443_PI430048170 | 3.31E-02 | -3.04535 | -2.81828 | -1.89857 |
| CUST_136102_PI430048170 | 5.17E-03 | -4.13119 | -0.39504 | -1.89844 |
| CUST_134831_PI430048170 | 5.46E-05 | -7.02421 | 5.956452 | -1.89667 |
| CUST_94191_PI430048170 | 5.96E-04 | -5.40675 | 2.500025 | -1.89663 |
| CUST_137158_PI430048170 | 1.26E-04 | -6.40677 | 4.676206 | -1.89629 |
| CUST_73711_PI430048170 | 9.22E-05 | -6.63161 | 5.148438 | -1.89514 |
| CUST_70012_PI430048170 | 5.50E-04 | -5.45968 | 2.617963 | -1.89495 |
| CUST_74180_PI430048170 | 1.86E-04 | -6.13226 | 4.090669 | -1.89439 |
| CUST_130474_PI430048170 | 3.87E-03 | -4.29811 | -0.01375 | -1.89428 |
| CUST_73676_PI430048170 | 2.32E-03 | -4.59207 | 0.65792 | -1.89364 |
| CUST_136562_PI430048170 | 5.33E-03 | -4.11241 | -0.43792 | -1.89292 |
| CUST_125376_PI430048170 | 8.98E-04 | -5.15543 | 1.936614 | -1.89219 |
| CUST_118434_PI430048170 | 1.39E-02 | -3.55919 | -1.69053 | -1.89184 |
| CUST_139296_PI430048170 | 2.03E-03 | -4.67031 | 0.836431 | -1.89162 |
| CUST_32913_PI430048170 | 5.36E-04 | -5.47416 | 2.650169 | -1.89153 |
| CUST_54618_PI430048170 | 3.34E-04 | -5.77077 | 3.305436 | -1.89145 |
| CUST_93713_PI430048170 | 1.49E-03 | -4.85729 | 1.262036 | -1.89085 |
| CUST_108251_PI430048170 | 2.32E-02 | -3.25563 | -2.36283 | -1.8906 |
| CUST_64789_PI430048170 | 2.70E-03 | -4.50525 | 0.459668 | -1.8899 |
| CUST_135846_PI430048170 | 4.13E-03 | -4.26108 | -0.09839 | -1.88988 |
| CUST_56534_PI430048170 | 9.99E-04 | -5.09204 | 1.793677 | -1.88927 |
| CUST_120213_PI430048170 | 1.66E-03 | -4.79306 | 1.116003 | -1.8889 |
| CUST_77745_PI430048170 | 5.22E-05 | -7.05678 | 6.022508 | -1.88772 |
| CUST_120905_PI430048170 | 1.38E-03 | -4.89875 | 1.356168 | -1.88743 |
| CUST_105095_PI430048170 | 8.20E-04 | -5.21102 | 2.061676 | -1.8867 |
| CUST_136838_PI430048170 | 7.18E-07 | -10.7167 | 12.50032 | -1.8862 |
| CUST_88484_PI430048170 | 1.49E-03 | -4.85601 | 1.259116 | -1.88605 |
| CUST_31105_PI430048170 | 1.86E-03 | -4.72436 | 0.959602 | -1.88572 |
| CUST_139589_PI430048170 | 2.52E-05 | -7.61776 | 7.136726 | -1.88568 |
| CUST_7645_PI430048170 | 4.81E-03 | -4.17411 | -0.29705 | -1.88489 |
| CUST_73162_PI430048170 | 1.03E-02 | -3.73478 | -1.29576 | -1.88437 |
| CUST_135970_PI430048170 | 2.26E-05 | -7.72294 | 7.340626 | -1.88159 |
| CUST_140004_PI430048170 | 1.84E-02 | -3.39523 | -2.05552 | -1.881 |
| CUST_21300_PI430048170 | 3.33E-03 | -4.38452 | 0.18375 | -1.87935 |
| CUST_42667_PI430048170 | 4.70E-03 | -4.18708 | -0.26745 | -1.8791 |
| CUST_43918_PI430048170 | 3.65E-04 | -5.71319 | 3.178951 | -1.87843 |
| CUST_130991_PI430048170 | 8.35E-05 | -6.70636 | 5.30392 | -1.87783 |
| CUST_136419_PI430048170 | 8.39E-04 | -5.19707 | 2.03033 | -1.87745 |
| CUST_17185_PI430048170 | 3.82E-02 | -2.95776 | -3.00489 | -1.8755 |
| CUST_118960_PI430048170 | 8.63E-05 | -6.68342 | 5.25629 | -1.87521 |
| CUST_23035_PI430048170 | 1.25E-03 | -4.96067 | 1.496585 | -1.8742 |
| CUST_134202_PI430048170 | 6.97E-07 | -10.7485 | 12.54877 | -1.87405 |
| CUST_138705_PI430048170 | 5.26E-04 | -5.4844 | 2.672944 | -1.87322 |
| CUST_73593_PI430048170 | 3.26E-03 | -4.39618 | 0.210414 | -1.87314 |
| CUST_139059_PI430048170 | 5.57E-04 | -5.45234 | 2.601627 | -1.87259 |
| CUST_31210_PI430048170 | 3.35E-04 | -5.77044 | 3.304712 | -1.87098 |
| CUST_137274_PI430048170 | 6.73E-04 | -5.33137 | 2.331602 | -1.87093 |
| CUST_79572_PI430048170 | 9.99E-05 | -6.56987 | 5.01944 | -1.86899 |
| CUST_29869_PI430048170 | 3.13E-03 | -4.4189 | 0.262341 | -1.86896 |
| CUST_27611_PI430048170 | 1.42E-04 | -6.31972 | 4.491586 | -1.86835 |
| CUST_139254_PI430048170 | 1.99E-04 | -6.08445 | 3.987716 | -1.86524 |
| CUST_141003_PI430048170 | 4.68E-03 | -4.18931 | -0.26234 | -1.86415 |
| CUST_130924_PI430048170 | 2.53E-03 | -4.54238 | 0.544476 | -1.86375 |
| CUST_114192_PI430048170 | 6.98E-04 | -5.30636 | 2.275609 | -1.8632 |
| CUST_13646_PI430048170 | 4.51E-05 | -7.17005 | 6.251099 | -1.86291 |
| CUST_28476_PI430048170 | 7.73E-03 | -3.90156 | -0.91803 | -1.86079 |
| CUST_126244_PI430048170 | 2.17E-02 | -3.29836 | -2.26916 | -1.85925 |
| CUST_136633_PI430048170 | 2.15E-03 | -4.6383 | 0.76343 | -1.85914 |
| CUST_136137_PI430048170 | 2.17E-04 | -6.0296 | 3.869263 | -1.85912 |
| CUST_138618_PI430048170 | 4.15E-04 | -5.63079 | 2.997337 | -1.85826 |
| CUST_18792_PI430048170 | 1.94E-04 | -6.10506 | 4.03214 | -1.85665 |
| CUST_89604_PI430048170 | 7.05E-03 | -3.9535 | -0.79997 | -1.85596 |
| CUST_143335_PI430048170 | 2.99E-03 | -4.44431 | 0.320427 | -1.8559 |
| CUST_137053_PI430048170 | 1.46E-02 | -3.52971 | -1.75642 | -1.85502 |
| CUST_144117_PI430048170 | 1.34E-03 | -4.91662 | 1.39672 | -1.85442 |
| CUST_141276_PI430048170 | 3.75E-05 | -7.30963 | 6.530281 | -1.85346 |
| CUST_83314_PI430048170 | 2.29E-03 | -4.60048 | 0.677122 | -1.85324 |
| CUST_20062_PI430048170 | 6.27E-05 | -6.91561 | 5.735075 | -1.85271 |
| CUST_81506_PI430048170 | 9.91E-06 | -8.36894 | 8.558084 | -1.85257 |
| CUST_93802_PI430048170 | 1.07E-03 | -5.0515 | 1.702129 | -1.85241 |
| CUST_139299_PI430048170 | 2.54E-03 | -4.53958 | 0.538073 | -1.85208 |
| CUST_1720_PI430048170 | 2.45E-02 | -3.22232 | -2.43563 | -1.85182 |
| CUST_88358_PI430048170 | 4.62E-04 | -5.56856 | 2.859715 | -1.85132 |
| CUST_109603_PI430048170 | 8.63E-05 | -6.68282 | 5.255034 | -1.85121 |
| CUST_100266_PI430048170 | 6.57E-04 | -5.34758 | 2.367856 | -1.85066 |
| CUST_136923_PI430048170 | 5.76E-06 | -8.84262 | 9.413167 | -1.85026 |
| CUST_56558_PI430048170 | 7.57E-06 | -8.63375 | 9.040001 | -1.84937 |
| CUST_140359_PI430048170 | 3.62E-04 | -5.72139 | 3.196991 | -1.84825 |
| CUST_140044_PI430048170 | 2.61E-02 | -3.18675 | -2.51309 | -1.84708 |
| CUST_139505_PI430048170 | 4.57E-03 | -4.20315 | -0.23074 | -1.84689 |
| CUST_143459_PI430048170 | 1.90E-03 | -4.712 | 0.931455 | -1.84622 |
| CUST_11661_PI430048170 | 1.38E-02 | -3.56305 | -1.68188 | -1.84566 |
| CUST_54559_PI430048170 | 3.42E-04 | -5.75723 | 3.275729 | -1.84557 |
| CUST_130031_PI430048170 | 1.69E-03 | -4.78142 | 1.089527 | -1.84549 |
| CUST_28866_PI430048170 | 1.03E-05 | -8.33429 | 8.49431 | -1.84506 |
| CUST_105842_PI430048170 | 9.78E-04 | -5.10624 | 1.825732 | -1.84503 |
| CUST_38014_PI430048170 | 1.95E-02 | -3.36132 | -2.13049 | -1.84485 |
| CUST_144095_PI430048170 | 3.17E-03 | -4.41117 | 0.244667 | -1.84431 |
| CUST_27570_PI430048170 | 3.58E-04 | -5.72859 | 3.212825 | -1.84407 |
| CUST_59302_PI430048170 | 1.03E-03 | -5.07285 | 1.750366 | -1.84364 |
| CUST_142658_PI430048170 | 2.46E-02 | -3.21966 | -2.44141 | -1.84268 |
| CUST_129058_PI430048170 | 5.58E-03 | -4.08801 | -0.49357 | -1.84266 |
| CUST_114898_PI430048170 | 3.84E-05 | -7.29157 | 6.494326 | -1.84222 |
| CUST_71267_PI430048170 | 1.42E-02 | -3.54803 | -1.71548 | -1.84189 |
| CUST_57459_PI430048170 | 3.05E-03 | -4.43467 | 0.298393 | -1.8413 |
| CUST_55352_PI430048170 | 3.68E-04 | -5.7066 | 3.164468 | -1.84118 |
| CUST_7629_PI430048170 | 1.10E-03 | -5.03235 | 1.658835 | -1.84112 |
| CUST_118335_PI430048170 | 3.81E-04 | -5.68531 | 3.117592 | -1.8408 |
| CUST_142830_PI430048170 | 1.85E-04 | -6.143 | 4.113757 | -1.84061 |
| CUST_126809_PI430048170 | 1.48E-03 | -4.85904 | 1.266012 | -1.84046 |
| CUST_41992_PI430048170 | 1.27E-02 | -3.61253 | -1.57098 | -1.83918 |
| CUST_56255_PI430048170 | 1.51E-05 | -8.02601 | 7.919231 | -1.83849 |
| CUST_134714_PI430048170 | 6.22E-03 | -4.02675 | -0.63323 | -1.8383 |
| CUST_136675_PI430048170 | 8.03E-03 | -3.87937 | -0.96841 | -1.83793 |
| CUST_63676_PI430048170 | 8.24E-04 | -5.20722 | 2.053149 | -1.83617 |
| CUST_64703_PI430048170 | 1.01E-04 | -6.56561 | 5.010512 | -1.83393 |
| CUST_131696_PI430048170 | 4.59E-03 | -4.2005 | -0.23678 | -1.83224 |
| CUST_127384_PI430048170 | 1.76E-03 | -4.75636 | 1.032483 | -1.83217 |
| CUST_27817_PI430048170 | 2.47E-03 | -4.55655 | 0.576828 | -1.83123 |
| CUST_131005_PI430048170 | 1.19E-03 | -4.98568 | 1.553234 | -1.83105 |
| CUST_50618_PI430048170 | 1.67E-02 | -3.45194 | -1.92973 | -1.83105 |
| CUST_30777_PI430048170 | 4.13E-03 | -4.26162 | -0.09714 | -1.82985 |
| CUST_97520_PI430048170 | 3.85E-04 | -5.67825 | 3.102048 | -1.82935 |
| CUST_118776_PI430048170 | 4.66E-04 | -5.56338 | 2.848243 | -1.82858 |
| CUST_61732_PI430048170 | 8.99E-04 | -5.15486 | 1.935321 | -1.82846 |
| CUST_137750_PI430048170 | 7.29E-05 | -6.801 | 5.499683 | -1.82693 |
| CUST_134372_PI430048170 | 3.19E-03 | -4.40828 | 0.238074 | -1.82643 |
| CUST_40546_PI430048170 | 1.55E-04 | -6.25999 | 4.36432 | -1.82622 |
| CUST_102226_PI430048170 | 7.87E-04 | -5.23656 | 2.119077 | -1.82527 |
| CUST_97739_PI430048170 | 1.60E-04 | -6.24024 | 4.322156 | -1.82506 |
| CUST_38731_PI430048170 | 5.05E-04 | -5.50939 | 2.728475 | -1.82427 |
| CUST_58795_PI430048170 | 1.30E-03 | -4.93572 | 1.44005 | -1.82352 |
| CUST_68263_PI430048170 | 2.01E-03 | -4.67693 | 0.851511 | -1.82344 |
| CUST_8105_PI430048170 | 6.23E-05 | -6.92384 | 5.751914 | -1.82341 |
| CUST_127652_PI430048170 | 7.65E-03 | -3.90718 | -0.90526 | -1.823 |
| CUST_90228_PI430048170 | 1.53E-02 | -3.50535 | -1.8108 | -1.8229 |
| CUST_111623_PI430048170 | 2.58E-03 | -4.52929 | 0.514573 | -1.82179 |
| CUST_140348_PI430048170 | 5.66E-03 | -4.07992 | -0.51201 | -1.82136 |
| CUST_7783_PI430048170 | 4.09E-02 | -2.91465 | -3.09597 | -1.82128 |
| CUST_127548_PI430048170 | 1.68E-03 | -4.78344 | 1.094125 | -1.81924 |
| CUST_41259_PI430048170 | 1.86E-04 | -6.13416 | 4.094759 | -1.81924 |
| CUST_135600_PI430048170 | 6.62E-04 | -5.34239 | 2.356253 | -1.81918 |
| CUST_134156_PI430048170 | 3.00E-03 | -4.44379 | 0.319217 | -1.81918 |
| CUST_55861_PI430048170 | 4.16E-05 | -7.23583 | 6.383018 | -1.8184 |
| CUST_92323_PI430048170 | 5.53E-06 | -8.8857 | 9.489372 | -1.81626 |
| CUST_17337_PI430048170 | 1.12E-02 | -3.6877 | -1.40193 | -1.81577 |
| CUST_135215_PI430048170 | 3.27E-02 | -3.05259 | -2.80278 | -1.81378 |
| CUST_142541_PI430048170 | 2.30E-04 | -5.99295 | 3.789896 | -1.81362 |
| CUST_128226_PI430048170 | 2.34E-03 | -4.58705 | 0.646465 | -1.81355 |
| CUST_61585_PI430048170 | 1.88E-04 | -6.12633 | 4.07791 | -1.81222 |
| CUST_121776_PI430048170 | 1.49E-04 | -6.28775 | 4.423518 | -1.81127 |
| CUST_75097_PI430048170 | 5.10E-03 | -4.13845 | -0.37847 | -1.81114 |
| CUST_126628_PI430048170 | 2.35E-03 | -4.584 | 0.639513 | -1.81077 |
| CUST_117542_PI430048170 | 6.84E-04 | -5.32168 | 2.30992 | -1.81066 |
| CUST_61601_PI430048170 | 6.94E-04 | -5.31119 | 2.286441 | -1.81043 |
| CUST_143772_PI430048170 | 5.32E-03 | -4.1136 | -0.43518 | -1.80938 |
| CUST_120844_PI430048170 | 1.81E-02 | -3.40351 | -2.03718 | -1.8083 |
| CUST_25443_PI430048170 | 1.11E-03 | -5.02689 | 1.646493 | -1.80641 |
| CUST_128059_PI430048170 | 2.98E-04 | -5.83713 | 3.450736 | -1.80619 |
| CUST_108103_PI430048170 | 1.49E-04 | -6.28493 | 4.417513 | -1.80614 |
| CUST_142642_PI430048170 | 1.35E-04 | -6.35598 | 4.568614 | -1.8055 |
| CUST_131642_PI430048170 | 4.34E-03 | -4.2321 | -0.1646 | -1.80373 |
| CUST_131042_PI430048170 | 1.78E-05 | -7.89251 | 7.665991 | -1.80149 |
| CUST_132415_PI430048170 | 3.66E-03 | -4.33 | 0.059144 | -1.80079 |
| CUST_95893_PI430048170 | 9.98E-05 | -6.57173 | 5.023331 | -1.80033 |
| CUST_133562_PI430048170 | 1.55E-04 | -6.26265 | 4.369996 | -1.79934 |
| CUST_131184_PI430048170 | 1.24E-03 | -4.96311 | 1.502119 | -1.79697 |
| CUST_32902_PI430048170 | 3.59E-03 | -4.34249 | 0.087682 | -1.7968 |
| CUST_13650_PI430048170 | 5.21E-04 | -5.49075 | 2.687068 | -1.79585 |
| CUST_29865_PI430048170 | 2.06E-03 | -4.66423 | 0.822562 | -1.79523 |
| CUST_29857_PI430048170 | 2.49E-03 | -4.55258 | 0.56777 | -1.79503 |
| CUST_128009_PI430048170 | 8.13E-04 | -5.21667 | 2.074387 | -1.79372 |
| CUST_75157_PI430048170 | 1.61E-05 | -7.97111 | 7.815397 | -1.79311 |
| CUST_136836_PI430048170 | 2.12E-03 | -4.64544 | 0.779713 | -1.79269 |
| CUST_127350_PI430048170 | 7.21E-03 | -3.94023 | -0.83014 | -1.79194 |
| CUST_52963_PI430048170 | 1.08E-03 | -5.04377 | 1.684658 | -1.79166 |
| CUST_139115_PI430048170 | 9.41E-05 | -6.61848 | 5.12104 | -1.79129 |
| CUST_12109_PI430048170 | 3.60E-02 | -2.99372 | -2.92851 | -1.78839 |
| CUST_35614_PI430048170 | 5.70E-03 | -4.07653 | -0.51974 | -1.78686 |
| CUST_58493_PI430048170 | 9.99E-03 | -3.75352 | -1.25343 | -1.78643 |
| CUST_99074_PI430048170 | 2.14E-03 | -4.64118 | 0.769989 | -1.7858 |
| CUST_80351_PI430048170 | 6.66E-03 | -3.98674 | -0.72434 | -1.78574 |
| CUST_139679_PI430048170 | 6.75E-04 | -5.32906 | 2.326446 | -1.78569 |
| CUST_20896_PI430048170 | 6.36E-03 | -4.01437 | -0.66143 | -1.78522 |
| CUST_13119_PI430048170 | 1.19E-03 | -4.98922 | 1.561256 | -1.78415 |
| CUST_137359_PI430048170 | 7.88E-04 | -5.2359 | 2.117594 | -1.78249 |
| CUST_91532_PI430048170 | 3.97E-04 | -5.65781 | 3.056973 | -1.78103 |
| CUST_82784_PI430048170 | 6.96E-04 | -5.30952 | 2.282696 | -1.78042 |
| CUST_68459_PI430048170 | 1.58E-04 | -6.24791 | 4.338535 | -1.77897 |
| CUST_126624_PI430048170 | 3.81E-04 | -5.68586 | 3.1188 | -1.77867 |
| CUST_133543_PI430048170 | 4.64E-04 | -5.56541 | 2.852745 | -1.77827 |
| CUST_65154_PI430048170 | 9.57E-05 | -6.60491 | 5.092724 | -1.77706 |
| CUST_137442_PI430048170 | 1.86E-02 | -3.3893 | -2.06864 | -1.77676 |
| CUST_88370_PI430048170 | 1.71E-02 | -3.4389 | -1.9587 | -1.776 |
| CUST_105233_PI430048170 | 1.86E-03 | -4.72424 | 0.959344 | -1.77586 |
| CUST_35606_PI430048170 | 2.19E-03 | -4.62693 | 0.737471 | -1.77491 |
| CUST_121584_PI430048170 | 3.48E-05 | -7.37456 | 6.659219 | -1.77485 |
| CUST_110219_PI430048170 | 3.06E-03 | -4.43208 | 0.292454 | -1.77396 |
| CUST_139899_PI430048170 | 4.57E-04 | -5.57407 | 2.871911 | -1.77396 |
| CUST_35945_PI430048170 | 1.18E-03 | -4.98997 | 1.562952 | -1.77376 |
| CUST_135106_PI430048170 | 4.59E-03 | -4.20072 | -0.23628 | -1.77346 |
| CUST_126572_PI430048170 | 2.43E-04 | -5.96285 | 3.724607 | -1.77243 |
| CUST_135328_PI430048170 | 1.56E-02 | -3.49072 | -1.84342 | -1.77187 |
| CUST_109534_PI430048170 | 2.15E-03 | -4.63866 | 0.764232 | -1.77063 |
| CUST_129342_PI430048170 | 1.88E-03 | -4.71913 | 0.947686 | -1.77062 |
| CUST_137945_PI430048170 | 1.94E-03 | -4.69882 | 0.901419 | -1.77058 |
| CUST_123947_PI430048170 | 5.29E-03 | -4.11823 | -0.42462 | -1.76994 |
| CUST_112205_PI430048170 | 1.04E-05 | -8.31481 | 8.45836 | -1.76894 |
| CUST_131431_PI430048170 | 8.04E-04 | -5.2234 | 2.089517 | -1.76782 |
| CUST_27609_PI430048170 | 2.45E-02 | -3.22281 | -2.43455 | -1.76469 |
| CUST_139122_PI430048170 | 1.12E-02 | -3.68989 | -1.39701 | -1.76447 |
| CUST_49755_PI430048170 | 2.56E-05 | -7.60054 | 7.103205 | -1.76371 |
| CUST_103390_PI430048170 | 1.27E-02 | -3.61412 | -1.56741 | -1.76351 |
| CUST_22246_PI430048170 | 2.70E-03 | -4.50442 | 0.457762 | -1.76184 |
| CUST_133446_PI430048170 | 7.03E-05 | -6.82855 | 5.556435 | -1.76126 |
| CUST_132582_PI430048170 | 1.33E-02 | -3.58494 | -1.63285 | -1.76 |
| CUST_137680_PI430048170 | 3.71E-02 | -2.97557 | -2.96711 | -1.75958 |
| CUST_46182_PI430048170 | 4.78E-04 | -5.54543 | 2.808465 | -1.75842 |
| CUST_103156_PI430048170 | 5.17E-03 | -4.13099 | -0.39551 | -1.75749 |
| CUST_127976_PI430048170 | 5.48E-04 | -5.46154 | 2.622093 | -1.75608 |
| CUST_127885_PI430048170 | 1.39E-02 | -3.55994 | -1.68883 | -1.75568 |
| CUST_125409_PI430048170 | 3.37E-04 | -5.76518 | 3.293165 | -1.75558 |
| CUST_56528_PI430048170 | 2.90E-03 | -4.4627 | 0.36244 | -1.75545 |
| CUST_125390_PI430048170 | 6.03E-03 | -4.04484 | -0.592 | -1.75513 |
| CUST_32937_PI430048170 | 3.14E-05 | -7.45769 | 6.823393 | -1.75296 |
| CUST_130431_PI430048170 | 4.14E-03 | -4.26049 | -0.09973 | -1.75253 |
| CUST_70462_PI430048170 | 1.87E-02 | -3.38666 | -2.0745 | -1.75252 |
| CUST_135000_PI430048170 | 3.11E-03 | -4.42066 | 0.266375 | -1.75237 |
| CUST_125420_PI430048170 | 1.00E-02 | -3.75279 | -1.25509 | -1.75175 |
| CUST_7905_PI430048170 | 9.73E-05 | -6.59309 | 5.068009 | -1.75172 |
| CUST_126759_PI430048170 | 2.09E-02 | -3.3199 | -2.22179 | -1.75121 |
| CUST_9536_PI430048170 | 2.17E-03 | -4.63229 | 0.749714 | -1.75092 |
| CUST_75042_PI430048170 | 7.41E-04 | -5.2717 | 2.197942 | -1.74801 |
| CUST_124207_PI430048170 | 8.34E-05 | -6.70793 | 5.307185 | -1.74577 |
| CUST_74063_PI430048170 | 6.08E-03 | -4.03988 | -0.60331 | -1.74423 |
| CUST_58207_PI430048170 | 1.86E-03 | -4.72659 | 0.964689 | -1.74347 |
| CUST_129962_PI430048170 | 2.51E-03 | -4.54746 | 0.556066 | -1.7431 |
| CUST_127057_PI430048170 | 1.37E-04 | -6.34853 | 4.552783 | -1.74134 |
| CUST_49141_PI430048170 | 1.00E-03 | -5.08914 | 1.787134 | -1.74053 |
| CUST_60001_PI430048170 | 4.58E-04 | -5.57355 | 2.870757 | -1.74046 |
| CUST_126922_PI430048170 | 1.28E-02 | -3.6063 | -1.58496 | -1.74028 |
| CUST_144926_PI430048170 | 5.91E-04 | -5.41138 | 2.510355 | -1.73946 |
| CUST_52461_PI430048170 | 1.58E-03 | -4.8212 | 1.180004 | -1.73471 |
| CUST_57115_PI430048170 | 6.48E-03 | -4.00209 | -0.68939 | -1.73459 |
| CUST_143052_PI430048170 | 1.47E-02 | -3.52682 | -1.76289 | -1.73403 |
| CUST_91035_PI430048170 | 3.68E-02 | -2.98039 | -2.95687 | -1.73377 |
| CUST_76144_PI430048170 | 1.32E-02 | -3.59059 | -1.62018 | -1.73311 |
| CUST_133487_PI430048170 | 1.31E-04 | -6.37851 | 4.616384 | -1.73211 |
| CUST_144612_PI430048170 | 5.97E-03 | -4.05013 | -0.57995 | -1.73169 |
| CUST_127367_PI430048170 | 7.13E-03 | -3.94748 | -0.81366 | -1.73008 |
| CUST_130554_PI430048170 | 4.07E-03 | -4.26939 | -0.07939 | -1.72952 |
| CUST_137159_PI430048170 | 1.46E-02 | -3.53059 | -1.75446 | -1.72923 |
| CUST_98384_PI430048170 | 9.92E-04 | -5.09641 | 1.803544 | -1.72866 |
| CUST_18070_PI430048170 | 3.16E-02 | -3.07237 | -2.76032 | -1.72811 |
| CUST_47686_PI430048170 | 2.63E-02 | -3.18207 | -2.52326 | -1.7263 |
| CUST_36907_PI430048170 | 2.86E-03 | -4.47092 | 0.381219 | -1.72618 |
| CUST_110859_PI430048170 | 1.95E-02 | -3.3603 | -2.13275 | -1.72559 |
| CUST_127810_PI430048170 | 9.94E-04 | -5.09544 | 1.801359 | -1.72425 |
| CUST_125335_PI430048170 | 1.03E-03 | -5.07393 | 1.752802 | -1.72317 |
| CUST_55799_PI430048170 | 5.72E-04 | -5.43434 | 2.561533 | -1.72231 |
| CUST_120242_PI430048170 | 4.96E-04 | -5.52216 | 2.756834 | -1.7223 |
| CUST_138380_PI430048170 | 1.61E-02 | -3.47493 | -1.87859 | -1.72216 |
| CUST_6124_PI430048170 | 1.04E-05 | -8.31342 | 8.455804 | -1.72079 |
| CUST_28858_PI430048170 | 2.62E-02 | -3.18284 | -2.52159 | -1.72043 |
| CUST_134918_PI430048170 | 3.93E-04 | -5.66471 | 3.072203 | -1.71738 |
| CUST_117715_PI430048170 | 6.12E-03 | -4.03591 | -0.61234 | -1.71672 |
| CUST_85968_PI430048170 | 3.17E-04 | -5.80299 | 3.376036 | -1.71576 |
| CUST_130693_PI430048170 | 1.48E-02 | -3.52214 | -1.77333 | -1.7151 |
| CUST_126725_PI430048170 | 2.81E-05 | -7.53691 | 6.978933 | -1.71482 |
| CUST_135793_PI430048170 | 3.83E-03 | -4.30282 | -0.003 | -1.71475 |
| CUST_89782_PI430048170 | 4.96E-04 | -5.52182 | 2.756081 | -1.71462 |
| CUST_136897_PI430048170 | 3.94E-02 | -2.93799 | -3.04672 | -1.71454 |
| CUST_125945_PI430048170 | 1.87E-03 | -4.71963 | 0.948832 | -1.71386 |
| CUST_137181_PI430048170 | 9.28E-03 | -3.798 | -1.15284 | -1.71339 |
| CUST_33999_PI430048170 | 1.83E-02 | -3.39947 | -2.04613 | -1.71251 |
| CUST_993_PI430048170 | 9.12E-04 | -5.14683 | 1.91723 | -1.71217 |
| CUST_8021_PI430048170 | 5.06E-04 | -5.50839 | 2.726252 | -1.71198 |
| CUST_64746_PI430048170 | 2.91E-04 | -5.85012 | 3.479115 | -1.71118 |
| CUST_136685_PI430048170 | 1.04E-03 | -5.06742 | 1.738099 | -1.7101 |
| CUST_7682_PI430048170 | 1.48E-02 | -3.52341 | -1.77051 | -1.70756 |
| CUST_127022_PI430048170 | 3.54E-05 | -7.35663 | 6.623664 | -1.70674 |
| CUST_12240_PI430048170 | 1.82E-04 | -6.15566 | 4.140955 | -1.70584 |
| CUST_131139_PI430048170 | 3.51E-02 | -3.00861 | -2.8968 | -1.70557 |
| CUST_11832_PI430048170 | 1.26E-03 | -4.95329 | 1.479859 | -1.70456 |
| CUST_145622_PI430048170 | 9.36E-04 | -5.13144 | 1.882544 | -1.70417 |
| CUST_111177_PI430048170 | 3.07E-03 | -4.42905 | 0.28553 | -1.70335 |
| CUST_76727_PI430048170 | 6.51E-03 | -4.00041 | -0.69322 | -1.70327 |
| CUST_134647_PI430048170 | 7.23E-03 | -3.93899 | -0.83298 | -1.70323 |
| CUST_97172_PI430048170 | 1.29E-05 | -8.14761 | 8.147685 | -1.70262 |
| CUST_28492_PI430048170 | 3.17E-05 | -7.44885 | 6.805988 | -1.70254 |
| CUST_38848_PI430048170 | 3.32E-03 | -4.38537 | 0.185703 | -1.70155 |
| CUST_97283_PI430048170 | 1.12E-03 | -5.02586 | 1.644164 | -1.70154 |
| CUST_73405_PI430048170 | 8.02E-03 | -3.8807 | -0.96539 | -1.70149 |
| CUST_96779_PI430048170 | 4.80E-03 | -4.17517 | -0.29464 | -1.70092 |
| CUST_136686_PI430048170 | 3.76E-03 | -4.31451 | 0.023738 | -1.70018 |
| CUST_140810_PI430048170 | 5.05E-05 | -7.0833 | 6.076192 | -1.69963 |
| CUST_122801_PI430048170 | 9.04E-03 | -3.81302 | -1.11884 | -1.69681 |
| CUST_103478_PI430048170 | 6.17E-03 | -4.03117 | -0.62315 | -1.69599 |
| CUST_44732_PI430048170 | 4.45E-02 | -2.86145 | -3.20765 | -1.69441 |
| CUST_138086_PI430048170 | 1.31E-02 | -3.59299 | -1.61481 | -1.6936 |
| CUST_69771_PI430048170 | 2.00E-03 | -4.68228 | 0.863717 | -1.69283 |
| CUST_112440_PI430048170 | 1.21E-03 | -4.97895 | 1.537995 | -1.69179 |
| CUST_51821_PI430048170 | 5.78E-03 | -4.06776 | -0.53975 | -1.69133 |
| CUST_126565_PI430048170 | 2.71E-04 | -5.89446 | 3.575839 | -1.69105 |
| CUST_109321_PI430048170 | 5.22E-03 | -4.12505 | -0.40907 | -1.69002 |
| CUST_119605_PI430048170 | 1.04E-06 | -10.3285 | 11.89783 | -1.68806 |
| CUST_124171_PI430048170 | 4.81E-02 | -2.81055 | -3.31372 | -1.68612 |
| CUST_140186_PI430048170 | 1.39E-02 | -3.5615 | -1.68535 | -1.68547 |
| CUST_72755_PI430048170 | 2.47E-03 | -4.55589 | 0.575323 | -1.68536 |
| CUST_134541_PI430048170 | 4.40E-05 | -7.19443 | 6.300072 | -1.68498 |
| CUST_118360_PI430048170 | 4.11E-04 | -5.63674 | 3.010492 | -1.68482 |
| CUST_136231_PI430048170 | 2.42E-05 | -7.66421 | 7.226971 | -1.68403 |
| CUST_13361_PI430048170 | 1.46E-02 | -3.53143 | -1.75259 | -1.68384 |
| CUST_137927_PI430048170 | 2.71E-04 | -5.893 | 3.572666 | -1.68314 |
| CUST_93649_PI430048170 | 9.83E-03 | -3.76354 | -1.23079 | -1.68268 |
| CUST_61423_PI430048170 | 4.45E-03 | -4.21832 | -0.19608 | -1.68156 |
| CUST_126672_PI430048170 | 1.06E-02 | -3.71712 | -1.33561 | -1.68062 |
| CUST_58480_PI430048170 | 1.65E-03 | -4.79453 | 1.119352 | -1.68025 |
| CUST_135278_PI430048170 | 1.19E-05 | -8.20236 | 8.249864 | -1.67965 |
| CUST_17462_PI430048170 | 9.73E-05 | -6.59379 | 5.06948 | -1.67942 |
| CUST_62540_PI430048170 | 2.75E-02 | -3.1548 | -2.58245 | -1.67896 |
| CUST_137662_PI430048170 | 5.54E-03 | -4.09178 | -0.48498 | -1.67787 |
| CUST_136455_PI430048170 | 5.11E-03 | -4.13814 | -0.37918 | -1.67675 |
| CUST_94426_PI430048170 | 1.95E-02 | -3.35887 | -2.13591 | -1.67566 |
| CUST_96212_PI430048170 | 4.73E-03 | -4.18337 | -0.27591 | -1.67539 |
| CUST_110424_PI430048170 | 2.02E-03 | -4.6736 | 0.84393 | -1.6752 |
| CUST_130562_PI430048170 | 6.16E-03 | -4.03205 | -0.62114 | -1.67507 |
| CUST_100796_PI430048170 | 3.36E-04 | -5.76739 | 3.29801 | -1.67363 |
| CUST_32078_PI430048170 | 1.19E-02 | -3.65184 | -1.48266 | -1.67258 |
| CUST_4913_PI430048170 | 2.69E-03 | -4.5062 | 0.461843 | -1.67051 |
| CUST_26141_PI430048170 | 3.61E-03 | -4.33827 | 0.078045 | -1.6705 |
| CUST_136525_PI430048170 | 5.75E-04 | -5.43015 | 2.552188 | -1.66946 |
| CUST_60440_PI430048170 | 3.01E-04 | -5.83091 | 3.43713 | -1.66804 |
| CUST_116780_PI430048170 | 1.42E-03 | -4.88343 | 1.321398 | -1.66737 |
| CUST_127072_PI430048170 | 3.50E-05 | -7.368 | 6.646221 | -1.66729 |
| CUST_103873_PI430048170 | 4.31E-03 | -4.23668 | -0.15413 | -1.66679 |
| CUST_112102_PI430048170 | 9.51E-03 | -3.78325 | -1.18622 | -1.66656 |
| CUST_140416_PI430048170 | 1.85E-04 | -6.14034 | 4.10805 | -1.66614 |
| CUST_136054_PI430048170 | 3.17E-03 | -4.41205 | 0.24668 | -1.66448 |
| CUST_130355_PI430048170 | 1.39E-03 | -4.89403 | 1.345458 | -1.66434 |
| CUST_83726_PI430048170 | 2.27E-03 | -4.60655 | 0.690986 | -1.66415 |
| CUST_129615_PI430048170 | 1.31E-04 | -6.3763 | 4.611696 | -1.66361 |
| CUST_140728_PI430048170 | 1.33E-02 | -3.58442 | -1.63402 | -1.66339 |
| CUST_138896_PI430048170 | 9.25E-03 | -3.79986 | -1.14864 | -1.6633 |
| CUST_19397_PI430048170 | 7.07E-04 | -5.29873 | 2.258521 | -1.66237 |
| CUST_157_PI430048170 | 8.15E-04 | -5.21542 | 2.071567 | -1.66207 |
| CUST_110400_PI430048170 | 2.73E-04 | -5.88939 | 3.564792 | -1.66132 |
| CUST_135636_PI430048170 | 1.46E-04 | -6.30104 | 4.451835 | -1.66115 |
| CUST_135787_PI430048170 | 1.94E-03 | -4.69948 | 0.902916 | -1.66078 |
| CUST_142461_PI430048170 | 9.07E-04 | -5.15064 | 1.925813 | -1.66022 |
| CUST_125355_PI430048170 | 4.71E-04 | -5.55672 | 2.83347 | -1.65981 |
| CUST_94561_PI430048170 | 7.49E-03 | -3.91919 | -0.87797 | -1.65892 |
| CUST_42075_PI430048170 | 1.09E-02 | -3.70274 | -1.36804 | -1.65683 |
| CUST_7695_PI430048170 | 3.88E-02 | -2.94776 | -3.02606 | -1.65614 |
| CUST_127128_PI430048170 | 8.42E-04 | -5.19485 | 2.025322 | -1.65595 |
| CUST_80720_PI430048170 | 1.82E-04 | -6.15588 | 4.141439 | -1.65529 |
| CUST_72691_PI430048170 | 1.09E-02 | -3.70066 | -1.37273 | -1.65513 |
| CUST_125068_PI430048170 | 7.05E-04 | -5.30085 | 2.263266 | -1.65503 |
| CUST_38886_PI430048170 | 1.18E-05 | -8.21298 | 8.269635 | -1.65497 |
| CUST_100548_PI430048170 | 4.26E-02 | -2.88912 | -3.14968 | -1.65468 |
| CUST_21918_PI430048170 | 6.05E-03 | -4.0428 | -0.59664 | -1.65313 |
| CUST_86806_PI430048170 | 6.03E-03 | -4.04494 | -0.59177 | -1.65271 |
| CUST_90882_PI430048170 | 4.45E-04 | -5.58938 | 2.905798 | -1.65263 |
| CUST_5620_PI430048170 | 7.08E-04 | -5.29804 | 2.256988 | -1.65186 |
| CUST_40882_PI430048170 | 1.33E-02 | -3.58666 | -1.62899 | -1.6518 |
| CUST_9165_PI430048170 | 9.93E-04 | -5.09607 | 1.802785 | -1.65175 |
| CUST_118581_PI430048170 | 4.16E-03 | -4.2572 | -0.10726 | -1.65139 |
| CUST_130521_PI430048170 | 1.18E-03 | -4.99025 | 1.563582 | -1.65007 |
| CUST_114327_PI430048170 | 6.03E-05 | -6.95502 | 5.815603 | -1.64882 |
| CUST_127141_PI430048170 | 1.45E-02 | -3.53767 | -1.73864 | -1.64835 |
| CUST_28572_PI430048170 | 1.26E-04 | -6.40652 | 4.675689 | -1.64751 |
| CUST_24726_PI430048170 | 5.31E-03 | -4.11585 | -0.43005 | -1.64721 |
| CUST_13651_PI430048170 | 2.86E-04 | -5.86207 | 3.505203 | -1.64688 |
| CUST_69532_PI430048170 | 4.25E-04 | -5.61808 | 2.969261 | -1.64657 |
| CUST_138231_PI430048170 | 5.42E-03 | -4.10348 | -0.45829 | -1.64626 |
| CUST_111557_PI430048170 | 7.30E-04 | -5.28059 | 2.217866 | -1.64607 |
| CUST_131938_PI430048170 | 2.24E-03 | -4.61395 | 0.707853 | -1.64603 |
| CUST_102803_PI430048170 | 1.08E-02 | -3.71117 | -1.34903 | -1.6452 |
| CUST_138053_PI430048170 | 1.19E-06 | -10.222 | 11.72922 | -1.64508 |
| CUST_109526_PI430048170 | 6.48E-03 | -4.00212 | -0.68933 | -1.64339 |
| CUST_80866_PI430048170 | 2.28E-05 | -7.7073 | 7.310412 | -1.64291 |
| CUST_56802_PI430048170 | 5.13E-04 | -5.49944 | 2.706373 | -1.64286 |
| CUST_77416_PI430048170 | 2.68E-03 | -4.50877 | 0.467714 | -1.64172 |
| CUST_103656_PI430048170 | 9.48E-04 | -5.12436 | 1.866602 | -1.6409 |
| CUST_95425_PI430048170 | 3.76E-04 | -5.69374 | 3.136166 | -1.64075 |
| CUST_127734_PI430048170 | 4.30E-04 | -5.6105 | 2.952514 | -1.64073 |
| CUST_117103_PI430048170 | 1.83E-04 | -6.14937 | 4.12746 | -1.64034 |
| CUST_90043_PI430048170 | 1.25E-02 | -3.62094 | -1.55209 | -1.63902 |
| CUST_18819_PI430048170 | 8.76E-04 | -5.17089 | 1.971408 | -1.63899 |
| CUST_126847_PI430048170 | 3.30E-02 | -3.04766 | -2.81334 | -1.63882 |
| CUST_52407_PI430048170 | 5.01E-05 | -7.09229 | 6.094372 | -1.63831 |
| CUST_104885_PI430048170 | 3.33E-04 | -5.77286 | 3.310019 | -1.637 |
| CUST_88793_PI430048170 | 6.82E-04 | -5.32273 | 2.312274 | -1.63689 |
| CUST_54063_PI430048170 | 9.00E-05 | -6.65134 | 5.189557 | -1.63652 |
| CUST_16607_PI430048170 | 2.12E-02 | -3.3106 | -2.24225 | -1.63315 |
| CUST_122981_PI430048170 | 2.79E-04 | -5.8776 | 3.539096 | -1.63307 |
| CUST_84085_PI430048170 | 2.53E-02 | -3.20367 | -2.47627 | -1.63267 |
| CUST_73890_PI430048170 | 1.16E-04 | -6.46154 | 4.791858 | -1.63208 |
| CUST_92446_PI430048170 | 1.92E-02 | -3.36965 | -2.11209 | -1.63155 |
| CUST_142261_PI430048170 | 3.59E-03 | -4.34116 | 0.08464 | -1.63152 |
| CUST_15017_PI430048170 | 1.68E-02 | -3.44963 | -1.93486 | -1.63147 |
| CUST_136203_PI430048170 | 7.12E-03 | -3.9478 | -0.81294 | -1.63109 |
| CUST_113878_PI430048170 | 6.32E-03 | -4.01855 | -0.6519 | -1.63062 |
| CUST_86971_PI430048170 | 1.60E-03 | -4.81586 | 1.167862 | -1.63052 |
| CUST_75207_PI430048170 | 3.52E-03 | -4.35255 | 0.110675 | -1.62938 |
| CUST_99521_PI430048170 | 7.57E-03 | -3.91355 | -0.89078 | -1.62916 |
| CUST_63459_PI430048170 | 6.96E-04 | -5.30906 | 2.281674 | -1.62913 |
| CUST_83679_PI430048170 | 9.67E-03 | -3.77262 | -1.21026 | -1.62898 |
| CUST_142495_PI430048170 | 1.24E-02 | -3.62562 | -1.54159 | -1.62869 |
| CUST_61599_PI430048170 | 4.78E-04 | -5.54588 | 2.809451 | -1.62816 |
| CUST_81454_PI430048170 | 3.11E-03 | -4.4224 | 0.270347 | -1.62805 |
| CUST_18772_PI430048170 | 1.62E-05 | -7.96256 | 7.799191 | -1.62805 |
| CUST_140314_PI430048170 | 2.11E-05 | -7.76909 | 7.429583 | -1.62764 |
| CUST_77632_PI430048170 | 6.82E-03 | -3.97248 | -0.7568 | -1.62758 |
| CUST_101370_PI430048170 | 5.84E-04 | -5.41898 | 2.527298 | -1.62679 |
| CUST_143244_PI430048170 | 4.88E-03 | -4.16504 | -0.31778 | -1.62614 |
| CUST_26142_PI430048170 | 1.35E-04 | -6.35593 | 4.568508 | -1.62597 |
| CUST_44540_PI430048170 | 7.19E-03 | -3.94196 | -0.82622 | -1.62592 |
| CUST_15444_PI430048170 | 1.17E-03 | -4.99558 | 1.575663 | -1.6258 |
| CUST_110951_PI430048170 | 2.31E-03 | -4.5949 | 0.664391 | -1.62507 |
| CUST_129657_PI430048170 | 7.07E-03 | -3.95192 | -0.80356 | -1.62451 |
| CUST_125529_PI430048170 | 8.18E-03 | -3.86908 | -0.99176 | -1.62425 |
| CUST_111269_PI430048170 | 8.03E-04 | -5.22437 | 2.091683 | -1.6239 |
| CUST_143211_PI430048170 | 2.37E-02 | -3.24477 | -2.3866 | -1.62336 |
| CUST_122249_PI430048170 | 3.47E-04 | -5.74698 | 3.253229 | -1.62242 |
| CUST_18766_PI430048170 | 6.14E-04 | -5.3884 | 2.459068 | -1.62174 |
| CUST_12637_PI430048170 | 8.40E-04 | -5.19617 | 2.028295 | -1.62014 |
| CUST_110002_PI430048170 | 1.61E-02 | -3.47248 | -1.88404 | -1.62014 |
| CUST_23523_PI430048170 | 2.50E-04 | -5.94426 | 3.684231 | -1.62002 |
| CUST_122851_PI430048170 | 8.24E-03 | -3.86439 | -1.0024 | -1.61971 |
| CUST_12991_PI430048170 | 4.02E-03 | -4.27736 | -0.06118 | -1.6195 |
| CUST_36993_PI430048170 | 1.62E-03 | -4.80469 | 1.142457 | -1.61919 |
| CUST_29886_PI430048170 | 2.36E-02 | -3.24696 | -2.3818 | -1.61915 |
| CUST_55235_PI430048170 | 5.61E-03 | -4.08467 | -0.50118 | -1.6189 |
| CUST_128894_PI430048170 | 4.33E-05 | -7.20476 | 6.320789 | -1.61803 |
| CUST_145678_PI430048170 | 2.72E-05 | -7.5583 | 7.020771 | -1.61742 |
| CUST_41529_PI430048170 | 1.20E-02 | -3.64796 | -1.49138 | -1.61705 |
| CUST_18075_PI430048170 | 4.94E-02 | -2.79469 | -3.3466 | -1.61639 |
| CUST_24592_PI430048170 | 1.50E-03 | -4.85044 | 1.246466 | -1.61573 |
| CUST_118375_PI430048170 | 4.66E-05 | -7.14737 | 6.205487 | -1.61571 |
| CUST_46539_PI430048170 | 6.30E-03 | -4.01955 | -0.64963 | -1.61476 |
| CUST_61572_PI430048170 | 5.60E-03 | -4.0855 | -0.49929 | -1.61384 |
| CUST_128890_PI430048170 | 7.57E-05 | -6.77519 | 5.446406 | -1.61317 |
| CUST_71476_PI430048170 | 2.05E-03 | -4.66565 | 0.825807 | -1.61287 |
| CUST_127339_PI430048170 | 4.09E-04 | -5.63959 | 3.016779 | -1.61252 |
| CUST_91321_PI430048170 | 1.60E-03 | -4.81571 | 1.167537 | -1.61234 |
| CUST_57924_PI430048170 | 1.14E-04 | -6.47211 | 4.814134 | -1.61216 |
| CUST_15357_PI430048170 | 4.02E-03 | -4.27633 | -0.06354 | -1.6121 |
| CUST_140868_PI430048170 | 2.99E-02 | -3.10737 | -2.685 | -1.61193 |
| CUST_5648_PI430048170 | 2.29E-03 | -4.60132 | 0.679046 | -1.6117 |
| CUST_3209_PI430048170 | 3.21E-02 | -3.0635 | -2.77938 | -1.61046 |
| CUST_127561_PI430048170 | 2.48E-03 | -4.55505 | 0.573412 | -1.60969 |
| CUST_35441_PI430048170 | 4.29E-02 | -2.88377 | -3.1609 | -1.60879 |
| CUST_88277_PI430048170 | 1.11E-04 | -6.49461 | 4.861492 | -1.60828 |
| CUST_101377_PI430048170 | 2.45E-02 | -3.22403 | -2.43189 | -1.60818 |
| CUST_33548_PI430048170 | 1.24E-03 | -4.96275 | 1.501307 | -1.60794 |
| CUST_89815_PI430048170 | 3.61E-03 | -4.33885 | 0.079371 | -1.60768 |
| CUST_105144_PI430048170 | 4.40E-03 | -4.22387 | -0.18341 | -1.60741 |
| CUST_67175_PI430048170 | 2.77E-03 | -4.49032 | 0.42555 | -1.60736 |
| CUST_66740_PI430048170 | 4.71E-04 | -5.55658 | 2.83316 | -1.6072 |
| CUST_24245_PI430048170 | 3.60E-03 | -4.34031 | 0.082698 | -1.6062 |
| CUST_93173_PI430048170 | 6.09E-04 | -5.39238 | 2.467957 | -1.60561 |
| CUST_130173_PI430048170 | 5.91E-03 | -4.05582 | -0.56698 | -1.60547 |
| CUST_114582_PI430048170 | 5.84E-04 | -5.41841 | 2.526021 | -1.60417 |
| CUST_16560_PI430048170 | 2.40E-03 | -4.57414 | 0.616993 | -1.60415 |
| CUST_51686_PI430048170 | 2.10E-02 | -3.31499 | -2.23258 | -1.60379 |
| CUST_58176_PI430048170 | 1.96E-03 | -4.69304 | 0.888245 | -1.60375 |
| CUST_136632_PI430048170 | 1.57E-02 | -3.48925 | -1.84669 | -1.6023 |
| CUST_135444_PI430048170 | 1.37E-03 | -4.90461 | 1.369473 | -1.60183 |
| CUST_137700_PI430048170 | 2.67E-02 | -3.1723 | -2.54449 | -1.60075 |
| CUST_141222_PI430048170 | 3.35E-03 | -4.37956 | 0.172426 | -1.60027 |
| CUST_74585_PI430048170 | 6.62E-04 | -5.34271 | 2.356968 | -1.59898 |
| CUST_133826_PI430048170 | 1.84E-02 | -3.39545 | -2.05503 | -1.59762 |
| CUST_28318_PI430048170 | 1.18E-04 | -6.44977 | 4.767035 | -1.59695 |
| CUST_90907_PI430048170 | 4.78E-03 | -4.1772 | -0.29002 | -1.59599 |
| CUST_120587_PI430048170 | 3.61E-05 | -7.34412 | 6.598845 | -1.59593 |
| CUST_118751_PI430048170 | 8.39E-03 | -3.85451 | -1.02481 | -1.5951 |
| CUST_131195_PI430048170 | 8.95E-03 | -3.8185 | -1.10643 | -1.59501 |
| CUST_129949_PI430048170 | 6.14E-03 | -4.03425 | -0.61614 | -1.59496 |
| CUST_36636_PI430048170 | 4.21E-03 | -4.2501 | -0.12347 | -1.59494 |
| CUST_145332_PI430048170 | 1.57E-02 | -3.48709 | -1.85151 | -1.59327 |
| CUST_119365_PI430048170 | 8.46E-03 | -3.85019 | -1.0346 | -1.59287 |
| CUST_134556_PI430048170 | 3.48E-02 | -3.01526 | -2.8826 | -1.59275 |
| CUST_67363_PI430048170 | 9.58E-03 | -3.77874 | -1.19641 | -1.5922 |
| CUST_143728_PI430048170 | 2.17E-02 | -3.29681 | -2.27254 | -1.59218 |
| CUST_132172_PI430048170 | 1.77E-02 | -3.41868 | -2.00357 | -1.59205 |
| CUST_47888_PI430048170 | 1.45E-03 | -4.87144 | 1.294166 | -1.59171 |
| CUST_142352_PI430048170 | 1.38E-03 | -4.89721 | 1.352688 | -1.59019 |
| CUST_135304_PI430048170 | 3.87E-04 | -5.6755 | 3.095971 | -1.5901 |
| CUST_28500_PI430048170 | 1.26E-03 | -4.95511 | 1.483985 | -1.58989 |
| CUST_110468_PI430048170 | 1.95E-04 | -6.09926 | 4.019651 | -1.58954 |
| CUST_125946_PI430048170 | 1.65E-02 | -3.45916 | -1.91369 | -1.58862 |
| CUST_36744_PI430048170 | 1.12E-03 | -5.023 | 1.637695 | -1.5884 |
| CUST_113195_PI430048170 | 2.38E-04 | -5.97589 | 3.752898 | -1.58797 |
| CUST_129895_PI430048170 | 5.79E-03 | -4.06694 | -0.54161 | -1.58671 |
| CUST_56141_PI430048170 | 1.29E-02 | -3.60317 | -1.59197 | -1.58649 |
| CUST_131332_PI430048170 | 8.57E-03 | -3.84341 | -1.04997 | -1.58597 |
| CUST_31433_PI430048170 | 1.64E-02 | -3.46233 | -1.90663 | -1.58461 |
| CUST_98574_PI430048170 | 2.84E-03 | -4.47439 | 0.389164 | -1.58425 |
| CUST_81939_PI430048170 | 9.16E-04 | -5.1438 | 1.910408 | -1.58382 |
| CUST_63155_PI430048170 | 2.20E-03 | -4.62353 | 0.729732 | -1.58375 |
| CUST_104416_PI430048170 | 2.96E-05 | -7.50374 | 6.913921 | -1.5837 |
| CUST_126344_PI430048170 | 1.37E-02 | -3.57104 | -1.66399 | -1.58345 |
| CUST_125329_PI430048170 | 1.17E-02 | -3.6606 | -1.46295 | -1.58329 |
| CUST_135516_PI430048170 | 1.99E-03 | -4.685 | 0.869915 | -1.58318 |
| CUST_20491_PI430048170 | 6.60E-03 | -3.99107 | -0.71449 | -1.58182 |
| CUST_136224_PI430048170 | 9.46E-03 | -3.78625 | -1.17942 | -1.5813 |
| CUST_66369_PI430048170 | 4.74E-03 | -4.18163 | -0.27988 | -1.58052 |
| CUST_139929_PI430048170 | 1.24E-04 | -6.42132 | 4.706973 | -1.58026 |
| CUST_134721_PI430048170 | 1.41E-04 | -6.32556 | 4.503998 | -1.58021 |
| CUST_55511_PI430048170 | 2.02E-02 | -3.33822 | -2.18144 | -1.58019 |
| CUST_13472_PI430048170 | 7.98E-04 | -5.22865 | 2.101307 | -1.58017 |
| CUST_137102_PI430048170 | 1.67E-02 | -3.4545 | -1.92404 | -1.57994 |
| CUST_65107_PI430048170 | 9.41E-04 | -5.12853 | 1.875999 | -1.57991 |
| CUST_75842_PI430048170 | 2.41E-06 | -9.61335 | 10.73837 | -1.57923 |
| CUST_16784_PI430048170 | 2.17E-02 | -3.29612 | -2.27407 | -1.57918 |
| CUST_135996_PI430048170 | 2.12E-02 | -3.31108 | -2.24119 | -1.57843 |
| CUST_130292_PI430048170 | 1.20E-03 | -4.98339 | 1.548065 | -1.57827 |
| CUST_126185_PI430048170 | 1.33E-03 | -4.91924 | 1.402671 | -1.57796 |
| CUST_128980_PI430048170 | 9.22E-05 | -6.63107 | 5.147311 | -1.5778 |
| CUST_129898_PI430048170 | 6.03E-03 | -4.04425 | -0.59335 | -1.57677 |
| CUST_16316_PI430048170 | 3.28E-04 | -5.78303 | 3.332301 | -1.5766 |
| CUST_34450_PI430048170 | 1.26E-04 | -6.40878 | 4.680461 | -1.57613 |
| CUST_53248_PI430048170 | 1.36E-04 | -6.35349 | 4.56332 | -1.57596 |
| CUST_81792_PI430048170 | 8.30E-04 | -5.20354 | 2.04488 | -1.57438 |
| CUST_142603_PI430048170 | 8.67E-04 | -5.1778 | 1.98697 | -1.57336 |
| CUST_88328_PI430048170 | 2.73E-03 | -4.49698 | 0.440772 | -1.57288 |
| CUST_135144_PI430048170 | 2.04E-02 | -3.33226 | -2.19458 | -1.57119 |
| CUST_159_PI430053867 | 2.87E-04 | -5.86073 | 3.502268 | -1.57118 |
| CUST_95311_PI430048170 | 1.47E-02 | -3.52872 | -1.75865 | -1.57114 |
| CUST_144405_PI430048170 | 1.51E-03 | -4.8488 | 1.242734 | -1.57094 |
| CUST_135575_PI430048170 | 1.26E-03 | -4.9515 | 1.475801 | -1.57094 |
| CUST_61942_PI430048170 | 1.32E-02 | -3.58998 | -1.62156 | -1.5709 |
| CUST_120611_PI430048170 | 2.37E-02 | -3.24431 | -2.3876 | -1.57067 |
| CUST_127455_PI430048170 | 8.39E-03 | -3.85454 | -1.02475 | -1.57066 |
| CUST_129763_PI430048170 | 2.69E-04 | -5.89964 | 3.587138 | -1.5703 |
| CUST_120654_PI430048170 | 5.11E-04 | -5.50172 | 2.711439 | -1.57025 |
| CUST_78575_PI430048170 | 3.99E-04 | -5.65494 | 3.050647 | -1.56986 |
| CUST_118159_PI430048170 | 1.25E-04 | -6.41324 | 4.689881 | -1.56903 |
| CUST_126389_PI430048170 | 4.28E-03 | -4.24121 | -0.14378 | -1.56877 |
| CUST_26585_PI430048170 | 2.12E-04 | -6.04564 | 3.903933 | -1.56871 |
| CUST_49562_PI430048170 | 1.22E-02 | -3.63519 | -1.52008 | -1.56829 |
| CUST_138480_PI430048170 | 6.38E-03 | -4.01275 | -0.66512 | -1.56682 |
| CUST_133232_PI430048170 | 2.46E-02 | -3.22154 | -2.43731 | -1.56616 |
| CUST_130110_PI430048170 | 7.87E-03 | -3.89155 | -0.94076 | -1.56573 |
| CUST_123052_PI430048170 | 7.43E-04 | -5.27061 | 2.195491 | -1.56557 |
| CUST_14936_PI430048170 | 1.14E-04 | -6.46902 | 4.807624 | -1.56534 |
| CUST_4458_PI430048170 | 5.36E-03 | -4.10985 | -0.44376 | -1.56514 |
| CUST_139004_PI430048170 | 7.27E-05 | -6.80404 | 5.505939 | -1.56514 |
| CUST_129001_PI430048170 | 4.10E-04 | -5.6388 | 3.015034 | -1.56479 |
| CUST_105230_PI430048170 | 1.61E-03 | -4.81214 | 1.159407 | -1.56451 |
| CUST_125773_PI430048170 | 8.00E-03 | -3.88219 | -0.96201 | -1.56418 |
| CUST_127211_PI430048170 | 1.90E-03 | -4.71364 | 0.935193 | -1.56302 |
| CUST_122537_PI430048170 | 8.23E-03 | -3.86527 | -1.00039 | -1.56217 |
| CUST_98644_PI430048170 | 1.09E-02 | -3.70225 | -1.36915 | -1.56206 |
| CUST_119423_PI430048170 | 2.46E-05 | -7.64657 | 7.192748 | -1.562 |
| CUST_100556_PI430048170 | 2.42E-04 | -5.96463 | 3.728465 | -1.56113 |
| CUST_125539_PI430048170 | 7.07E-03 | -3.95211 | -0.80314 | -1.56109 |
| CUST_75893_PI430048170 | 7.97E-05 | -6.73764 | 5.368752 | -1.56098 |
| CUST_82623_PI430048170 | 3.47E-04 | -5.74823 | 3.255968 | -1.56029 |
| CUST_92972_PI430048170 | 2.88E-03 | -4.46673 | 0.371645 | -1.5601 |
| CUST_8293_PI430048170 | 4.14E-03 | -4.26002 | -0.10081 | -1.55995 |
| CUST_55567_PI430048170 | 2.55E-03 | -4.53728 | 0.532819 | -1.55949 |
| CUST_107696_PI430048170 | 5.47E-04 | -5.4633 | 2.626017 | -1.55947 |
| CUST_133768_PI430048170 | 5.81E-04 | -5.42187 | 2.533737 | -1.55855 |
| CUST_136079_PI430048170 | 8.90E-04 | -5.16097 | 1.949077 | -1.55752 |
| CUST_75156_PI430048170 | 5.12E-05 | -7.07408 | 6.057546 | -1.55746 |
| CUST_16766_PI430048170 | 9.91E-04 | -5.09763 | 1.806299 | -1.55712 |
| CUST_118197_PI430048170 | 5.31E-03 | -4.11522 | -0.4315 | -1.55627 |
| CUST_69215_PI430048170 | 1.01E-02 | -3.74554 | -1.27146 | -1.55455 |
| CUST_26411_PI430048170 | 1.07E-03 | -5.04873 | 1.695868 | -1.55311 |
| CUST_47277_PI430048170 | 8.97E-05 | -6.65455 | 5.196227 | -1.5531 |
| CUST_109959_PI430048170 | 2.13E-04 | -6.04037 | 3.892548 | -1.55285 |
| CUST_135651_PI430048170 | 7.93E-05 | -6.74128 | 5.376299 | -1.55219 |
| CUST_54352_PI430048170 | 3.20E-04 | -5.79676 | 3.362398 | -1.55214 |
| CUST_81249_PI430048170 | 2.91E-04 | -5.85083 | 3.480652 | -1.55209 |
| CUST_11735_PI430048170 | 1.03E-04 | -6.54809 | 4.973819 | -1.55179 |
| CUST_74541_PI430048170 | 1.70E-03 | -4.777 | 1.079476 | -1.55099 |
| CUST_105830_PI430048170 | 1.78E-03 | -4.74935 | 1.016533 | -1.5507 |
| CUST_123111_PI430048170 | 6.42E-03 | -4.00794 | -0.67607 | -1.55053 |
| CUST_131016_PI430048170 | 3.49E-03 | -4.35817 | 0.123519 | -1.55039 |
| CUST_12269_PI430048170 | 5.63E-05 | -7.00279 | 5.91292 | -1.55031 |
| CUST_28016_PI430048170 | 1.02E-02 | -3.74153 | -1.28051 | -1.54906 |
| CUST_122568_PI430048170 | 5.79E-03 | -4.06704 | -0.54139 | -1.54905 |
| CUST_118939_PI430048170 | 4.48E-03 | -4.21317 | -0.20785 | -1.54853 |
| CUST_94082_PI430048170 | 1.52E-05 | -8.01206 | 7.892897 | -1.5481 |
| CUST_32531_PI430048170 | 1.26E-02 | -3.61619 | -1.56275 | -1.54701 |
| CUST_129428_PI430048170 | 1.57E-02 | -3.48851 | -1.84835 | -1.54672 |
| CUST_119692_PI430048170 | 3.92E-04 | -5.66708 | 3.077412 | -1.54565 |
| CUST_19959_PI430048170 | 7.89E-03 | -3.89012 | -0.944 | -1.54538 |
| CUST_18079_PI430048170 | 4.70E-02 | -2.82429 | -3.28517 | -1.54528 |
| CUST_24382_PI430048170 | 9.78E-03 | -3.76664 | -1.22378 | -1.54454 |
| CUST_88114_PI430048170 | 1.21E-03 | -4.97858 | 1.537168 | -1.5436 |
| CUST_45370_PI430048170 | 1.28E-02 | -3.60915 | -1.57856 | -1.54328 |
| CUST_17523_PI430048170 | 1.90E-04 | -6.11993 | 4.064159 | -1.54264 |
| CUST_97521_PI430048170 | 1.02E-04 | -6.55591 | 4.990203 | -1.54185 |
| CUST_93277_PI430048170 | 1.13E-04 | -6.48009 | 4.83094 | -1.54039 |
| CUST_134937_PI430048170 | 5.70E-03 | -4.07598 | -0.521 | -1.53897 |
| CUST_75897_PI430048170 | 8.56E-03 | -3.84369 | -1.04935 | -1.53863 |
| CUST_134720_PI430048170 | 1.60E-03 | -4.8129 | 1.161142 | -1.5385 |
| CUST_100189_PI430048170 | 5.86E-04 | -5.41707 | 2.523031 | -1.53822 |
| CUST_95578_PI430048170 | 5.88E-05 | -6.97349 | 5.853261 | -1.53737 |
| CUST_140670_PI430048170 | 2.26E-03 | -4.60723 | 0.692532 | -1.53675 |
| CUST_65149_PI430048170 | 6.34E-04 | -5.3691 | 2.415972 | -1.5354 |
| CUST_57617_PI430048170 | 8.85E-06 | -8.46725 | 8.738153 | -1.53265 |
| CUST_124196_PI430048170 | 3.64E-05 | -7.3305 | 6.571792 | -1.53245 |
| CUST_134844_PI430048170 | 6.08E-03 | -4.04005 | -0.60291 | -1.5319 |
| CUST_55387_PI430048170 | 1.11E-04 | -6.49608 | 4.864603 | -1.5309 |
| CUST_134480_PI430048170 | 9.51E-04 | -5.12223 | 1.861793 | -1.52967 |
| CUST_19912_PI430048170 | 2.26E-05 | -7.7191 | 7.3332 | -1.52953 |
| CUST_129203_PI430048170 | 4.22E-03 | -4.24911 | -0.12573 | -1.52909 |
| CUST_122742_PI430048170 | 1.08E-04 | -6.51402 | 4.902307 | -1.52869 |
| CUST_81956_PI430048170 | 6.38E-04 | -5.36397 | 2.404515 | -1.52825 |
| CUST_43524_PI430048170 | 5.57E-04 | -5.45155 | 2.599866 | -1.52773 |
| CUST_63154_PI430048170 | 2.43E-03 | -4.566 | 0.598408 | -1.52671 |
| CUST_50970_PI430048170 | 5.71E-03 | -4.07444 | -0.52451 | -1.52586 |
| CUST_143377_PI430048170 | 4.44E-05 | -7.18377 | 6.278666 | -1.52483 |
| CUST_74540_PI430048170 | 6.38E-04 | -5.36448 | 2.40565 | -1.52431 |
| CUST_65155_PI430048170 | 4.96E-05 | -7.09891 | 6.107754 | -1.52422 |
| CUST_139742_PI430048170 | 2.21E-03 | -4.62114 | 0.72426 | -1.5239 |
| CUST_9979_PI430048170 | 1.60E-03 | -4.81429 | 1.164292 | -1.52353 |
| CUST_67295_PI430048170 | 2.98E-03 | -4.44712 | 0.326847 | -1.52349 |
| CUST_131259_PI430048170 | 3.77E-04 | -5.69155 | 3.131343 | -1.52295 |
| CUST_131137_PI430048170 | 4.45E-02 | -2.86157 | -3.2074 | -1.52284 |
| CUST_985_PI430048170 | 5.14E-03 | -4.13469 | -0.38707 | -1.52228 |
| CUST_96680_PI430048170 | 1.21E-02 | -3.64364 | -1.50108 | -1.52211 |
| CUST_25885_PI430048170 | 3.77E-03 | -4.31373 | 0.021946 | -1.52188 |
| CUST_41301_PI430048170 | 2.71E-04 | -5.89482 | 3.576628 | -1.52138 |
| CUST_97097_PI430048170 | 1.11E-03 | -5.02763 | 1.648175 | -1.52092 |
| CUST_78476_PI430048170 | 7.60E-05 | -6.77329 | 5.442498 | -1.52066 |
| CUST_85891_PI430048170 | 1.59E-04 | -6.24336 | 4.328814 | -1.52035 |
| CUST_81630_PI430048170 | 6.03E-03 | -4.04449 | -0.59281 | -1.51983 |
| CUST_114952_PI430048170 | 1.12E-04 | -6.48589 | 4.843156 | -1.51977 |
| CUST_109000_PI430048170 | 8.02E-03 | -3.88026 | -0.9664 | -1.51936 |
| CUST_31880_PI430048170 | 3.14E-03 | -4.41613 | 0.256003 | -1.51926 |
| CUST_74169_PI430048170 | 7.57E-04 | -5.2592 | 2.169889 | -1.51827 |
| CUST_23284_PI430048170 | 1.78E-02 | -3.41693 | -2.00746 | -1.51811 |
| CUST_103504_PI430048170 | 8.15E-04 | -5.21531 | 2.071331 | -1.51723 |
| CUST_141444_PI430048170 | 2.32E-02 | -3.25661 | -2.36068 | -1.5169 |
| CUST_72668_PI430048170 | 2.32E-03 | -4.59393 | 0.662165 | -1.51679 |
| CUST_58111_PI430048170 | 1.14E-03 | -5.01144 | 1.611548 | -1.51623 |
| CUST_47790_PI430048170 | 1.92E-02 | -3.36815 | -2.1154 | -1.51604 |
| CUST_79299_PI430048170 | 2.17E-03 | -4.6309 | 0.746545 | -1.51578 |
| CUST_134822_PI430048170 | 7.73E-03 | -3.9015 | -0.91816 | -1.51387 |
| CUST_28604_PI430048170 | 2.10E-04 | -6.04902 | 3.911241 | -1.51279 |
| CUST_135443_PI430048170 | 4.16E-03 | -4.2575 | -0.10657 | -1.51184 |
| CUST_121515_PI430048170 | 7.53E-03 | -3.91656 | -0.88395 | -1.51183 |
| CUST_54025_PI430048170 | 7.69E-03 | -3.90463 | -0.91105 | -1.51084 |
| CUST_17843_PI430048170 | 1.14E-02 | -3.67849 | -1.42267 | -1.51069 |
| CUST_24531_PI430048170 | 8.52E-04 | -5.18798 | 2.009881 | -1.50992 |
| CUST_29212_PI430048170 | 3.81E-03 | -4.30782 | 0.00845 | -1.50991 |
| CUST_117651_PI430048170 | 1.73E-02 | -3.43305 | -1.97168 | -1.50897 |
| CUST_55447_PI430048170 | 1.50E-02 | -3.51335 | -1.79296 | -1.50837 |
| CUST_84329_PI430048170 | 2.77E-02 | -3.15071 | -2.5913 | -1.50757 |
| CUST_94543_PI430048170 | 2.20E-02 | -3.28724 | -2.29356 | -1.50622 |
| CUST_129200_PI430048170 | 1.56E-02 | -3.49084 | -1.84316 | -1.50615 |
| CUST_127643_PI430048170 | 6.09E-03 | -4.03927 | -0.60469 | -1.5055 |
| CUST_127704_PI430048170 | 2.52E-03 | -4.54551 | 0.551627 | -1.50533 |
| CUST_145607_PI430048170 | 1.43E-03 | -4.87985 | 1.313272 | -1.50514 |
| CUST_93026_PI430048170 | 2.13E-04 | -6.04184 | 3.895716 | -1.50507 |
| CUST_132396_PI430048170 | 1.80E-03 | -4.74455 | 1.005595 | -1.50365 |
| CUST_120634_PI430048170 | 1.78E-03 | -4.74963 | 1.017161 | -1.50339 |
| CUST_12098_PI430048170 | 7.50E-04 | -5.26471 | 2.182248 | -1.5032 |
| CUST_82123_PI430048170 | 2.77E-03 | -4.48914 | 0.422867 | -1.50241 |
| CUST_142476_PI430048170 | 1.53E-03 | -4.84189 | 1.227048 | -1.50235 |
| CUST_58756_PI430048170 | 4.48E-04 | -5.58562 | 2.897476 | -1.50139 |
| CUST_144500_PI430048170 | 2.35E-05 | -7.6886 | 7.274226 | -1.5012 |
| CUST_96733_PI430048170 | 7.17E-05 | -6.81293 | 5.524271 | -1.50096 |
| CUST_128104_PI430048170 | 3.69E-04 | -5.70556 | 3.162167 | -1.50088 |
| CUST_51383_PI430048170 | 2.84E-02 | -3.13663 | -2.6218 | -1.50046 |
| CUST_132969_PI430048170 | 1.53E-02 | -3.50464 | -1.81238 | -1.49927 |
| CUST_21632_PI430048170 | 1.07E-03 | -5.05212 | 1.703539 | -1.49859 |
| CUST_95015_PI430048170 | 1.05E-04 | -6.53266 | 4.941437 | -1.4979 |
| CUST_138137_PI430048170 | 1.80E-03 | -4.74268 | 1.001348 | -1.49744 |
| CUST_11915_PI430048170 | 2.10E-04 | -6.05044 | 3.914313 | -1.49713 |
| CUST_59345_PI430048170 | 1.24E-02 | -3.62509 | -1.54277 | -1.49681 |
| CUST_75885_PI430048170 | 1.24E-02 | -3.62587 | -1.54103 | -1.49669 |
| CUST_144051_PI430048170 | 1.31E-04 | -6.37653 | 4.612189 | -1.49633 |
| CUST_124974_PI430048170 | 9.86E-04 | -5.10112 | 1.814185 | -1.4963 |
| CUST_119036_PI430048170 | 6.65E-04 | -5.33983 | 2.350527 | -1.49574 |
| CUST_142959_PI430048170 | 6.76E-03 | -3.97779 | -0.74471 | -1.4947 |
| CUST_145211_PI430048170 | 4.75E-04 | -5.55106 | 2.820935 | -1.49414 |
| CUST_56443_PI430048170 | 1.11E-03 | -5.02648 | 1.645563 | -1.49362 |
| CUST_44818_PI430048170 | 6.06E-03 | -4.04129 | -0.60009 | -1.49339 |
| CUST_25168_PI430048170 | 1.26E-02 | -3.61535 | -1.56465 | -1.49244 |
| CUST_139027_PI430048170 | 3.10E-03 | -4.42422 | 0.274492 | -1.4921 |
| CUST_47769_PI430048170 | 7.51E-05 | -6.78085 | 5.458097 | -1.49204 |
| CUST_32015_PI430048170 | 1.32E-03 | -4.9276 | 1.42163 | -1.49154 |
| CUST_69300_PI430048170 | 7.40E-04 | -5.27281 | 2.200424 | -1.48923 |
| CUST_78674_PI430048170 | 1.77E-03 | -4.75359 | 1.026177 | -1.48714 |
| CUST_81965_PI430048170 | 2.15E-03 | -4.63695 | 0.760335 | -1.48641 |
| CUST_57622_PI430048170 | 3.79E-06 | -9.20775 | 10.05102 | -1.48611 |
| CUST_112935_PI430048170 | 1.21E-02 | -3.64434 | -1.49952 | -1.48611 |
| CUST_23361_PI430048170 | 2.36E-03 | -4.58206 | 0.635069 | -1.48577 |
| CUST_142885_PI430048170 | 3.24E-02 | -3.05762 | -2.792 | -1.48566 |
| CUST_114951_PI430048170 | 5.01E-05 | -7.09295 | 6.095698 | -1.48549 |
| CUST_128831_PI430048170 | 5.58E-03 | -4.08845 | -0.49257 | -1.48445 |
| CUST_94212_PI430048170 | 7.96E-03 | -3.88552 | -0.95444 | -1.48422 |
| CUST_130079_PI430048170 | 2.63E-02 | -3.18174 | -2.52398 | -1.48353 |
| CUST_47569_PI430048170 | 5.80E-03 | -4.06623 | -0.54323 | -1.48254 |
| CUST_126810_PI430048170 | 3.56E-03 | -4.34616 | 0.096082 | -1.48154 |
| CUST_136428_PI430048170 | 8.77E-04 | -5.17022 | 1.969903 | -1.48134 |
| CUST_128188_PI430048170 | 1.44E-02 | -3.53993 | -1.73359 | -1.48131 |
| CUST_24746_PI430048170 | 3.47E-03 | -4.36164 | 0.131471 | -1.48052 |
| CUST_145423_PI430048170 | 5.75E-04 | -5.43024 | 2.552388 | -1.48049 |
| CUST_87253_PI430048170 | 4.78E-04 | -5.54507 | 2.80766 | -1.48002 |
| CUST_27405_PI430048170 | 2.17E-02 | -3.29767 | -2.27065 | -1.47936 |
| CUST_89866_PI430048170 | 9.16E-04 | -5.14326 | 1.909186 | -1.47918 |
| CUST_76953_PI430048170 | 1.04E-03 | -5.06639 | 1.735782 | -1.47794 |
| CUST_93382_PI430048170 | 6.91E-04 | -5.31499 | 2.294944 | -1.47789 |
| CUST_93046_PI430048170 | 1.29E-02 | -3.60156 | -1.59559 | -1.47781 |
| CUST_64890_PI430048170 | 8.73E-04 | -5.17386 | 1.978109 | -1.47608 |
| CUST_33726_PI430048170 | 1.21E-02 | -3.64019 | -1.50885 | -1.47505 |
| CUST_125460_PI430048170 | 5.26E-04 | -5.4849 | 2.674051 | -1.47399 |
| CUST_19273_PI430048170 | 1.68E-03 | -4.78471 | 1.097011 | -1.47314 |
| CUST_109493_PI430048170 | 9.16E-04 | -5.14411 | 1.911096 | -1.47305 |
| CUST_130278_PI430048170 | 5.19E-03 | -4.12894 | -0.40018 | -1.47248 |
| CUST_73378_PI430048170 | 2.93E-02 | -3.11785 | -2.66237 | -1.46927 |
| CUST_69755_PI430048170 | 1.93E-04 | -6.10789 | 4.038229 | -1.46828 |
| CUST_55797_PI430048170 | 1.22E-02 | -3.63852 | -1.5126 | -1.46817 |
| CUST_125980_PI430048170 | 8.90E-03 | -3.82191 | -1.0987 | -1.4681 |
| CUST_60422_PI430048170 | 1.50E-02 | -3.51565 | -1.78783 | -1.46714 |
| CUST_26155_PI430048170 | 5.01E-03 | -4.14934 | -0.35362 | -1.46713 |
| CUST_113753_PI430048170 | 1.52E-02 | -3.50683 | -1.80751 | -1.46696 |
| CUST_93476_PI430048170 | 4.87E-04 | -5.53352 | 2.782046 | -1.46678 |
| CUST_113571_PI430048170 | 4.33E-02 | -2.8781 | -3.17279 | -1.46602 |
| CUST_105744_PI430048170 | 6.00E-04 | -5.40213 | 2.48971 | -1.46573 |
| CUST_126923_PI430048170 | 2.87E-02 | -3.13001 | -2.63611 | -1.46546 |
| CUST_27668_PI430048170 | 2.89E-04 | -5.85567 | 3.491237 | -1.46522 |
| CUST_73001_PI430048170 | 2.08E-03 | -4.65729 | 0.806732 | -1.46492 |
| CUST_52913_PI430048170 | 2.17E-02 | -3.29655 | -2.27313 | -1.46473 |
| CUST_48272_PI430048170 | 1.05E-03 | -5.0644 | 1.731284 | -1.46468 |
| CUST_14747_PI430048170 | 1.86E-03 | -4.72376 | 0.958254 | -1.46454 |
| CUST_126039_PI430048170 | 1.74E-02 | -3.42811 | -1.98266 | -1.46447 |
| CUST_132329_PI430048170 | 6.08E-05 | -6.94853 | 5.802351 | -1.46399 |
| CUST_46245_PI430048170 | 8.08E-03 | -3.87601 | -0.97604 | -1.46391 |
| CUST_42967_PI430048170 | 3.09E-02 | -3.08658 | -2.72977 | -1.46377 |
| CUST_32474_PI430048170 | 1.81E-02 | -3.40644 | -2.0307 | -1.46358 |
| CUST_26449_PI430048170 | 1.63E-03 | -4.80262 | 1.137753 | -1.46353 |
| CUST_121801_PI430048170 | 6.52E-03 | -3.99891 | -0.69663 | -1.463 |
| CUST_81790_PI430048170 | 5.73E-03 | -4.07249 | -0.52896 | -1.46193 |
| CUST_28477_PI430048170 | 1.26E-02 | -3.61591 | -1.56338 | -1.46076 |
| CUST_87057_PI430048170 | 2.32E-02 | -3.25719 | -2.35942 | -1.46059 |
| CUST_28044_PI430048170 | 2.87E-03 | -4.46863 | 0.375994 | -1.46032 |
| CUST_1654_PI430048170 | 2.13E-03 | -4.64381 | 0.775991 | -1.45818 |
| CUST_95914_PI430048170 | 9.64E-04 | -5.11475 | 1.844916 | -1.45754 |
| CUST_101192_PI430048170 | 2.23E-03 | -4.61725 | 0.715386 | -1.45652 |
| CUST_48147_PI430048170 | 7.00E-04 | -5.30479 | 2.272094 | -1.45642 |
| CUST_64761_PI430048170 | 2.59E-04 | -5.92452 | 3.641304 | -1.45596 |
| CUST_143481_PI430048170 | 8.76E-03 | -3.83145 | -1.07708 | -1.45594 |
| CUST_127080_PI430048170 | 1.85E-02 | -3.3927 | -2.06111 | -1.45539 |
| CUST_140674_PI430048170 | 3.82E-03 | -4.3048 | 0.001546 | -1.45513 |
| CUST_119495_PI430048170 | 3.08E-02 | -3.08769 | -2.72738 | -1.45455 |
| CUST_140253_PI430048170 | 5.71E-04 | -5.43533 | 2.563738 | -1.45407 |
| CUST_137516_PI430048170 | 2.84E-04 | -5.86612 | 3.514046 | -1.45361 |
| CUST_62093_PI430048170 | 1.58E-03 | -4.82336 | 1.184912 | -1.45344 |
| CUST_125673_PI430048170 | 5.21E-03 | -4.12706 | -0.40449 | -1.45312 |
| CUST_40639_PI430048170 | 2.62E-04 | -5.91587 | 3.622478 | -1.45267 |
| CUST_67854_PI430048170 | 4.00E-02 | -2.92958 | -3.0645 | -1.45242 |
| CUST_90424_PI430048170 | 3.06E-02 | -3.09219 | -2.71771 | -1.45238 |
| CUST_41384_PI430048170 | 8.20E-04 | -5.21031 | 2.060082 | -1.45159 |
| CUST_132836_PI430048170 | 6.35E-04 | -5.36734 | 2.412041 | -1.45128 |
| CUST_107246_PI430048170 | 2.10E-03 | -4.65199 | 0.794652 | -1.45088 |
| CUST_119684_PI430048170 | 1.94E-02 | -3.36196 | -2.12907 | -1.45013 |
| CUST_126118_PI430048170 | 1.23E-02 | -3.63085 | -1.52985 | -1.4487 |
| CUST_143055_PI430048170 | 3.37E-05 | -7.4039 | 6.717274 | -1.44869 |
| CUST_16259_PI430048170 | 3.07E-03 | -4.42939 | 0.286314 | -1.44833 |
| CUST_103049_PI430048170 | 1.06E-03 | -5.05738 | 1.715415 | -1.44831 |
| CUST_81899_PI430048170 | 1.71E-05 | -7.9281 | 7.733755 | -1.44826 |
| CUST_142568_PI430048170 | 1.86E-03 | -4.72443 | 0.959781 | -1.44779 |
| CUST_128402_PI430048170 | 1.43E-02 | -3.54229 | -1.72832 | -1.44754 |
| CUST_70537_PI430048170 | 1.41E-02 | -3.553 | -1.70437 | -1.44726 |
| CUST_126561_PI430048170 | 2.97E-04 | -5.83963 | 3.456192 | -1.44724 |
| CUST_75467_PI430048170 | 1.15E-04 | -6.46566 | 4.80055 | -1.44648 |
| CUST_6216_PI430048170 | 4.63E-02 | -2.83502 | -3.26283 | -1.44633 |
| CUST_71976_PI430048170 | 7.15E-03 | -3.94553 | -0.81811 | -1.44544 |
| CUST_74651_PI430048170 | 1.77E-02 | -3.41771 | -2.00572 | -1.44349 |
| CUST_48948_PI430048170 | 3.60E-04 | -5.72579 | 3.206667 | -1.44308 |
| CUST_128728_PI430048170 | 3.55E-03 | -4.34775 | 0.099714 | -1.44294 |
| CUST_97294_PI430048170 | 3.34E-03 | -4.38275 | 0.179713 | -1.44281 |
| CUST_65102_PI430048170 | 1.71E-02 | -3.43941 | -1.95757 | -1.44156 |
| CUST_136696_PI430048170 | 4.74E-04 | -5.55244 | 2.823984 | -1.44148 |
| CUST_121151_PI430048170 | 1.05E-02 | -3.72643 | -1.31459 | -1.44055 |
| CUST_55599_PI430048170 | 4.89E-04 | -5.53198 | 2.778634 | -1.43974 |
| CUST_93712_PI430048170 | 2.18E-02 | -3.293 | -2.28092 | -1.43745 |
| CUST_39395_PI430048170 | 1.08E-03 | -5.04655 | 1.690951 | -1.43737 |
| CUST_136045_PI430048170 | 1.99E-04 | -6.08454 | 3.987911 | -1.43712 |
| CUST_57699_PI430048170 | 1.95E-03 | -4.69799 | 0.899519 | -1.43684 |
| CUST_129713_PI430048170 | 1.82E-04 | -6.15281 | 4.134829 | -1.43672 |
| CUST_138408_PI430048170 | 9.29E-03 | -3.7971 | -1.15489 | -1.43621 |
| CUST_54668_PI430048170 | 6.39E-03 | -4.01064 | -0.66992 | -1.43619 |
| CUST_14934_PI430048170 | 7.24E-04 | -5.2848 | 2.227296 | -1.43548 |
| CUST_84366_PI430048170 | 1.47E-02 | -3.52579 | -1.76518 | -1.43441 |
| CUST_18964_PI430048170 | 4.66E-04 | -5.5632 | 2.847847 | -1.43437 |
| CUST_50041_PI430048170 | 7.94E-04 | -5.23119 | 2.107002 | -1.43272 |
| CUST_135145_PI430048170 | 1.17E-03 | -4.99977 | 1.585148 | -1.43255 |
| CUST_108784_PI430048170 | 1.12E-02 | -3.68644 | -1.40478 | -1.43253 |
| CUST_145069_PI430048170 | 3.55E-03 | -4.34735 | 0.098807 | -1.4325 |
| CUST_137998_PI430048170 | 1.38E-02 | -3.5651 | -1.67728 | -1.43231 |
| CUST_57384_PI430048170 | 6.73E-03 | -3.98088 | -0.73769 | -1.4318 |
| CUST_79316_PI430048170 | 4.71E-02 | -2.82295 | -3.28794 | -1.43161 |
| CUST_118096_PI430048170 | 1.49E-02 | -3.52014 | -1.77781 | -1.43064 |
| CUST_102754_PI430048170 | 7.58E-04 | -5.25821 | 2.167659 | -1.4291 |
| CUST_27650_PI430048170 | 8.66E-05 | -6.68029 | 5.249779 | -1.42883 |
| CUST_62985_PI430048170 | 1.05E-04 | -6.53762 | 4.951861 | -1.42846 |
| CUST_61759_PI430048170 | 2.58E-03 | -4.52955 | 0.515165 | -1.42832 |
| CUST_16261_PI430048170 | 1.12E-05 | -8.25444 | 8.346654 | -1.42705 |
| CUST_17986_PI430048170 | 4.04E-04 | -5.64695 | 3.033025 | -1.42646 |
| CUST_43575_PI430048170 | 7.70E-04 | -5.24968 | 2.148534 | -1.42546 |
| CUST_113890_PI430048170 | 1.21E-03 | -4.97698 | 1.53355 | -1.42499 |
| CUST_29843_PI430048170 | 2.69E-02 | -3.16915 | -2.55131 | -1.42424 |
| CUST_113349_PI430048170 | 1.19E-03 | -4.98887 | 1.560471 | -1.42366 |
| CUST_125884_PI430048170 | 4.06E-03 | -4.27119 | -0.07527 | -1.42357 |
| CUST_135885_PI430048170 | 3.42E-02 | -3.0239 | -2.86417 | -1.42326 |
| CUST_106836_PI430048170 | 4.40E-05 | -7.1936 | 6.298408 | -1.42312 |
| CUST_26905_PI430048170 | 8.92E-04 | -5.15919 | 1.945076 | -1.42221 |
| CUST_41953_PI430048170 | 3.34E-03 | -4.3824 | 0.17891 | -1.42199 |
| CUST_109887_PI430048170 | 1.76E-04 | -6.17706 | 4.186897 | -1.42182 |
| CUST_137110_PI430048170 | 1.01E-02 | -3.74476 | -1.27322 | -1.42083 |
| CUST_126568_PI430048170 | 3.77E-02 | -2.96569 | -2.98809 | -1.41868 |
| CUST_130548_PI430048170 | 4.07E-03 | -4.2697 | -0.07868 | -1.41858 |
| CUST_70261_PI430048170 | 2.06E-03 | -4.66442 | 0.822991 | -1.41787 |
| CUST_15425_PI430048170 | 2.58E-02 | -3.19138 | -2.50303 | -1.41768 |
| CUST_73308_PI430048170 | 2.02E-03 | -4.67424 | 0.845376 | -1.41693 |
| CUST_123063_PI430048170 | 5.49E-03 | -4.09689 | -0.47331 | -1.41666 |
| CUST_143111_PI430048170 | 9.22E-05 | -6.63022 | 5.145544 | -1.41658 |
| CUST_107782_PI430048170 | 1.94E-03 | -4.70026 | 0.904693 | -1.4159 |
| CUST_37905_PI430048170 | 7.41E-04 | -5.27224 | 2.199144 | -1.41565 |
| CUST_134961_PI430048170 | 2.19E-02 | -3.29166 | -2.28387 | -1.41556 |
| CUST_103601_PI430048170 | 4.25E-05 | -7.21956 | 6.350456 | -1.41475 |
| CUST_4865_PI430048170 | 1.14E-03 | -5.01382 | 1.616928 | -1.41469 |
| CUST_103775_PI430048170 | 2.48E-03 | -4.55433 | 0.571754 | -1.41431 |
| CUST_117780_PI430048170 | 9.09E-03 | -3.80975 | -1.12625 | -1.41334 |
| CUST_108124_PI430048170 | 1.01E-03 | -5.0868 | 1.781857 | -1.41314 |
| CUST_104684_PI430048170 | 9.09E-04 | -5.14897 | 1.922056 | -1.41219 |
| CUST_109986_PI430048170 | 2.52E-02 | -3.20622 | -2.47072 | -1.41212 |
| CUST_27416_PI430048170 | 2.84E-02 | -3.1356 | -2.62402 | -1.41191 |
| CUST_10846_PI430048170 | 9.10E-04 | -5.14826 | 1.920447 | -1.41171 |
| CUST_49259_PI430048170 | 1.23E-02 | -3.63022 | -1.53126 | -1.41064 |
| CUST_123144_PI430048170 | 2.78E-02 | -3.14867 | -2.59572 | -1.41042 |
| CUST_136048_PI430048170 | 3.63E-02 | -2.98774 | -2.94124 | -1.41034 |
| CUST_63156_PI430048170 | 4.19E-03 | -4.25285 | -0.11718 | -1.40986 |
| CUST_41523_PI430048170 | 9.13E-04 | -5.1463 | 1.91603 | -1.40866 |
| CUST_94959_PI430048170 | 4.71E-04 | -5.55683 | 2.833723 | -1.40859 |
| CUST_24494_PI430048170 | 3.82E-03 | -4.30558 | 0.003314 | -1.40798 |
| CUST_118851_PI430048170 | 6.28E-04 | -5.37412 | 2.427187 | -1.40787 |
| CUST_141558_PI430048170 | 1.00E-02 | -3.7518 | -1.25731 | -1.40763 |
| CUST_137923_PI430048170 | 8.99E-06 | -8.44614 | 8.699599 | -1.40753 |
| CUST_96019_PI430048170 | 3.83E-03 | -4.30279 | -0.00305 | -1.40698 |
| CUST_103148_PI430048170 | 4.30E-05 | -7.21005 | 6.3314 | -1.40666 |
| CUST_20659_PI430048170 | 2.75E-03 | -4.49356 | 0.432966 | -1.40653 |
| CUST_11561_PI430048170 | 2.17E-03 | -4.63279 | 0.750844 | -1.40633 |
| CUST_18800_PI430048170 | 9.30E-03 | -3.79611 | -1.15712 | -1.40514 |
| CUST_66215_PI430048170 | 9.22E-03 | -3.80172 | -1.14443 | -1.40508 |
| CUST_61279_PI430048170 | 5.77E-03 | -4.06919 | -0.5365 | -1.40502 |
| CUST_25557_PI430048170 | 4.30E-04 | -5.61121 | 2.954091 | -1.40482 |
| CUST_113654_PI430048170 | 2.31E-02 | -3.26057 | -2.35203 | -1.40472 |
| CUST_73159_PI430048170 | 1.79E-02 | -3.41174 | -2.01896 | -1.4046 |
| CUST_50338_PI430048170 | 1.37E-03 | -4.90474 | 1.369766 | -1.40317 |
| CUST_11494_PI430048170 | 1.84E-03 | -4.73145 | 0.975771 | -1.40259 |
| CUST_136026_PI430048170 | 8.80E-03 | -3.82934 | -1.08187 | -1.4025 |
| CUST_39912_PI430048170 | 6.77E-03 | -3.97702 | -0.74646 | -1.40187 |
| CUST_52337_PI430048170 | 4.02E-02 | -2.92686 | -3.07023 | -1.40136 |
| CUST_96853_PI430048170 | 8.33E-03 | -3.85851 | -1.01574 | -1.39942 |
| CUST_51614_PI430048170 | 1.24E-02 | -3.62884 | -1.53435 | -1.39849 |
| CUST_134687_PI430048170 | 4.08E-03 | -4.26736 | -0.08403 | -1.39801 |
| CUST_88330_PI430048170 | 5.46E-04 | -5.46412 | 2.627851 | -1.39711 |
| CUST_19587_PI430048170 | 1.26E-02 | -3.61689 | -1.56119 | -1.397 |
| CUST_70175_PI430048170 | 2.56E-03 | -4.53663 | 0.53135 | -1.396 |
| CUST_26575_PI430048170 | 2.34E-03 | -4.58702 | 0.646396 | -1.39585 |
| CUST_71710_PI430048170 | 8.11E-03 | -3.87383 | -0.98098 | -1.39577 |
| CUST_70741_PI430048170 | 1.21E-05 | -8.18867 | 8.224356 | -1.3957 |
| CUST_6592_PI430048170 | 2.02E-03 | -4.67426 | 0.845433 | -1.39484 |
| CUST_120939_PI430048170 | 1.93E-02 | -3.36728 | -2.11732 | -1.39444 |
| CUST_138014_PI430048170 | 5.20E-05 | -7.06265 | 6.034406 | -1.39402 |
| CUST_108346_PI430048170 | 1.94E-02 | -3.36286 | -2.12708 | -1.39399 |
| CUST_2057_PI430048170 | 2.54E-03 | -4.54006 | 0.539172 | -1.39329 |
| CUST_103892_PI430048170 | 3.46E-04 | -5.74892 | 3.257478 | -1.39258 |
| CUST_113793_PI430048170 | 9.71E-05 | -6.59547 | 5.072985 | -1.39204 |
| CUST_63235_PI430048170 | 2.80E-03 | -4.48388 | 0.41085 | -1.39138 |
| CUST_96805_PI430048170 | 7.95E-03 | -3.88615 | -0.95302 | -1.3903 |
| CUST_14455_PI430048170 | 9.51E-04 | -5.1229 | 1.86331 | -1.39003 |
| CUST_136121_PI430048170 | 1.71E-03 | -4.77529 | 1.075575 | -1.38954 |
| CUST_117004_PI430048170 | 3.16E-04 | -5.80456 | 3.379478 | -1.38949 |
| CUST_136853_PI430048170 | 2.94E-02 | -3.11744 | -2.66326 | -1.3892 |
| CUST_127285_PI430048170 | 5.63E-04 | -5.44395 | 2.582948 | -1.38878 |
| CUST_67274_PI430048170 | 1.05E-03 | -5.06357 | 1.729405 | -1.38875 |
| CUST_143037_PI430048170 | 3.79E-02 | -2.96332 | -2.99309 | -1.38843 |
| CUST_82472_PI430048170 | 1.45E-02 | -3.53758 | -1.73885 | -1.38806 |
| CUST_98018_PI430048170 | 7.16E-04 | -5.29146 | 2.242242 | -1.38804 |
| CUST_7728_PI430048170 | 1.14E-02 | -3.6765 | -1.42716 | -1.38701 |
| CUST_135079_PI430048170 | 9.67E-03 | -3.77245 | -1.21064 | -1.38639 |
| CUST_124839_PI430048170 | 1.59E-04 | -6.24303 | 4.328114 | -1.38573 |
| CUST_136412_PI430048170 | 4.13E-02 | -2.90705 | -3.11199 | -1.38474 |
| CUST_61911_PI430048170 | 7.72E-04 | -5.24803 | 2.14482 | -1.38465 |
| CUST_120818_PI430048170 | 2.93E-03 | -4.45633 | 0.34788 | -1.38408 |
| CUST_44605_PI430048170 | 9.14E-03 | -3.80661 | -1.13335 | -1.38388 |
| CUST_68025_PI430048170 | 9.05E-03 | -3.81265 | -1.11967 | -1.38232 |
| CUST_97752_PI430048170 | 1.81E-02 | -3.40607 | -2.03152 | -1.38218 |
| CUST_47072_PI430048170 | 7.16E-04 | -5.29202 | 2.243479 | -1.38213 |
| CUST_93754_PI430048170 | 1.35E-02 | -3.57865 | -1.64694 | -1.38095 |
| CUST_126528_PI430048170 | 4.22E-03 | -4.24868 | -0.12671 | -1.37859 |
| CUST_118709_PI430048170 | 2.55E-02 | -3.19915 | -2.48612 | -1.37775 |
| CUST_133728_PI430048170 | 1.40E-02 | -3.55629 | -1.69702 | -1.37773 |
| CUST_139459_PI430048170 | 3.14E-03 | -4.41589 | 0.25546 | -1.37759 |
| CUST_70540_PI430048170 | 8.30E-04 | -5.20373 | 2.045308 | -1.37568 |
| CUST_102508_PI430048170 | 7.95E-03 | -3.88575 | -0.95394 | -1.37536 |
| CUST_77423_PI430048170 | 9.97E-03 | -3.75504 | -1.25 | -1.37519 |
| CUST_121476_PI430048170 | 2.12E-02 | -3.3103 | -2.24292 | -1.3751 |
| CUST_101732_PI430048170 | 1.77E-03 | -4.75466 | 1.028623 | -1.37497 |
| CUST_104456_PI430048170 | 7.98E-03 | -3.88379 | -0.95837 | -1.3744 |
| CUST_129921_PI430048170 | 1.44E-02 | -3.5407 | -1.73187 | -1.3741 |
| CUST_9316_PI430048170 | 2.41E-04 | -5.96676 | 3.733084 | -1.37344 |
| CUST_145002_PI430048170 | 5.48E-03 | -4.09741 | -0.47214 | -1.37319 |
| CUST_67436_PI430048170 | 1.46E-02 | -3.53198 | -1.75137 | -1.37253 |
| CUST_97269_PI430048170 | 1.18E-02 | -3.65922 | -1.46605 | -1.37214 |
| CUST_13649_PI430048170 | 6.55E-03 | -3.99628 | -0.70263 | -1.37169 |
| CUST_7699_PI430048170 | 8.54E-03 | -3.84519 | -1.04593 | -1.37057 |
| CUST_135585_PI430048170 | 3.07E-03 | -4.42962 | 0.286848 | -1.37005 |
| CUST_126709_PI430048170 | 1.75E-02 | -3.42424 | -1.99123 | -1.36986 |
| CUST_50873_PI430048170 | 2.51E-04 | -5.94266 | 3.680743 | -1.36974 |
| CUST_106419_PI430048170 | 1.83E-02 | -3.39711 | -2.05135 | -1.36965 |
| CUST_37988_PI430048170 | 2.59E-03 | -4.5289 | 0.513689 | -1.36928 |
| CUST_80467_PI430048170 | 2.15E-03 | -4.6386 | 0.76411 | -1.36917 |
| CUST_90793_PI430048170 | 3.11E-02 | -3.08279 | -2.73793 | -1.36801 |
| CUST_82512_PI430048170 | 6.67E-04 | -5.33733 | 2.344931 | -1.3673 |
| CUST_142640_PI430048170 | 3.37E-02 | -3.03399 | -2.8426 | -1.36716 |
| CUST_95835_PI430048170 | 9.94E-05 | -6.57503 | 5.03025 | -1.36708 |
| CUST_129604_PI430048170 | 5.71E-03 | -4.07534 | -0.52246 | -1.36669 |
| CUST_43737_PI430048170 | 1.21E-03 | -4.97767 | 1.535109 | -1.36643 |
| CUST_119135_PI430048170 | 4.87E-04 | -5.53355 | 2.782115 | -1.3661 |
| CUST_132438_PI430048170 | 7.97E-03 | -3.88469 | -0.95634 | -1.36488 |
| CUST_55033_PI430048170 | 8.19E-03 | -3.86814 | -0.99389 | -1.36488 |
| CUST_142047_PI430048170 | 2.15E-02 | -3.30123 | -2.26284 | -1.36482 |
| CUST_116954_PI430048170 | 6.67E-04 | -5.33816 | 2.346795 | -1.36451 |
| CUST_35474_PI430048170 | 6.69E-03 | -3.98419 | -0.73013 | -1.36413 |
| CUST_44268_PI430048170 | 6.58E-03 | -3.99315 | -0.70976 | -1.3638 |
| CUST_98993_PI430048170 | 8.15E-04 | -5.21462 | 2.069787 | -1.36296 |
| CUST_13959_PI430048170 | 3.16E-02 | -3.0726 | -2.75984 | -1.36235 |
| CUST_84268_PI430048170 | 1.42E-02 | -3.54683 | -1.71817 | -1.36232 |
| CUST_28871_PI430048170 | 1.12E-02 | -3.68735 | -1.40272 | -1.36153 |
| CUST_54552_PI430048170 | 9.67E-03 | -3.77242 | -1.21071 | -1.36151 |
| CUST_6018_PI430048170 | 8.05E-03 | -3.87813 | -0.97122 | -1.36097 |
| CUST_109290_PI430048170 | 2.09E-03 | -4.65486 | 0.801199 | -1.36089 |
| CUST_32296_PI430048170 | 2.05E-05 | -7.79949 | 7.488014 | -1.36076 |
| CUST_84540_PI430048170 | 7.72E-03 | -3.90221 | -0.91656 | -1.36067 |
| CUST_132419_PI430048170 | 2.10E-02 | -3.31583 | -2.23075 | -1.36058 |
| CUST_95048_PI430048170 | 2.71E-03 | -4.5019 | 0.452002 | -1.36056 |
| CUST_109585_PI430048170 | 1.47E-03 | -4.86396 | 1.277179 | -1.36015 |
| CUST_111109_PI430048170 | 5.18E-04 | -5.49397 | 2.694218 | -1.36014 |
| CUST_123564_PI430048170 | 2.83E-02 | -3.13736 | -2.6202 | -1.35991 |
| CUST_140215_PI430048170 | 3.95E-03 | -4.28715 | -0.03881 | -1.35978 |
| CUST_31715_PI430048170 | 3.19E-02 | -3.06771 | -2.77034 | -1.35971 |
| CUST_51973_PI430048170 | 2.08E-02 | -3.32123 | -2.21886 | -1.35964 |
| CUST_142415_PI430048170 | 2.69E-03 | -4.5065 | 0.462513 | -1.35963 |
| CUST_135701_PI430048170 | 9.96E-05 | -6.57356 | 5.027157 | -1.35955 |
| CUST_2087_PI430048170 | 5.05E-05 | -7.08426 | 6.078143 | -1.35824 |
| CUST_7688_PI430048170 | 6.73E-03 | -3.98054 | -0.73846 | -1.35822 |
| CUST_126348_PI430048170 | 3.62E-02 | -2.99023 | -2.93594 | -1.35747 |
| CUST_100520_PI430048170 | 1.46E-04 | -6.2978 | 4.444942 | -1.35679 |
| CUST_75850_PI430048170 | 4.63E-02 | -2.83543 | -3.26198 | -1.35648 |
| CUST_75471_PI430048170 | 1.71E-04 | -6.19697 | 4.229573 | -1.35625 |
| CUST_79681_PI430048170 | 1.93E-02 | -3.36727 | -2.11734 | -1.35602 |
| CUST_114757_PI430048170 | 1.03E-02 | -3.73832 | -1.28777 | -1.35511 |
| CUST_13048_PI430048170 | 1.15E-04 | -6.46485 | 4.798829 | -1.35506 |
| CUST_120989_PI430048170 | 2.39E-03 | -4.57644 | 0.622256 | -1.3548 |
| CUST_119779_PI430048170 | 1.40E-03 | -4.89155 | 1.339828 | -1.35426 |
| CUST_16594_PI430048170 | 1.01E-02 | -3.74531 | -1.27199 | -1.35423 |
| CUST_70992_PI430048170 | 1.86E-03 | -4.72449 | 0.959906 | -1.35412 |
| CUST_14679_PI430048170 | 1.74E-03 | -4.76412 | 1.050146 | -1.35272 |
| CUST_11590_PI430048170 | 1.14E-02 | -3.67441 | -1.43187 | -1.3514 |
| CUST_127605_PI430048170 | 1.81E-03 | -4.74045 | 0.996256 | -1.35137 |
| CUST_118891_PI430048170 | 3.05E-03 | -4.43262 | 0.293705 | -1.35094 |
| CUST_49938_PI430048170 | 1.83E-03 | -4.73418 | 0.981985 | -1.35061 |
| CUST_77687_PI430048170 | 1.25E-03 | -4.95719 | 1.488699 | -1.35014 |
| CUST_130478_PI430048170 | 4.47E-02 | -2.85663 | -3.21773 | -1.3496 |
| CUST_70303_PI430048170 | 8.97E-05 | -6.65455 | 5.196244 | -1.34909 |
| CUST_117863_PI430048170 | 3.25E-03 | -4.39785 | 0.214224 | -1.34876 |
| CUST_30987_PI430048170 | 6.84E-04 | -5.32108 | 2.308568 | -1.34802 |
| CUST_60011_PI430048170 | 1.48E-02 | -3.52406 | -1.76906 | -1.34762 |
| CUST_134343_PI430048170 | 1.11E-03 | -5.02851 | 1.650169 | -1.34745 |
| CUST_17972_PI430048170 | 9.19E-03 | -3.80354 | -1.1403 | -1.34742 |
| CUST_5677_PI430048170 | 1.63E-02 | -3.46648 | -1.8974 | -1.34674 |
| CUST_102314_PI430048170 | 6.87E-03 | -3.96829 | -0.76633 | -1.3465 |
| CUST_105484_PI430048170 | 1.80E-03 | -4.74256 | 1.001074 | -1.34647 |
| CUST_224_PI430053867 | 2.33E-02 | -3.25504 | -2.36412 | -1.34566 |
| CUST_136982_PI430048170 | 1.78E-02 | -3.41536 | -2.01092 | -1.34532 |
| CUST_143774_PI430048170 | 2.59E-02 | -3.18937 | -2.5074 | -1.34462 |
| CUST_135011_PI430048170 | 1.17E-03 | -4.99474 | 1.573749 | -1.34458 |
| CUST_139605_PI430048170 | 1.38E-02 | -3.56444 | -1.67877 | -1.34386 |
| CUST_142246_PI430048170 | 4.88E-03 | -4.16546 | -0.31681 | -1.34374 |
| CUST_31580_PI430048170 | 1.52E-02 | -3.50661 | -1.808 | -1.34371 |
| CUST_42866_PI430048170 | 7.59E-03 | -3.9119 | -0.89455 | -1.3434 |
| CUST_69184_PI430048170 | 1.83E-04 | -6.14997 | 4.128737 | -1.3426 |
| CUST_129303_PI430048170 | 1.92E-04 | -6.11001 | 4.042801 | -1.34194 |
| CUST_14712_PI430048170 | 2.21E-03 | -4.62133 | 0.724696 | -1.34163 |
| CUST_100369_PI430048170 | 3.64E-04 | -5.71525 | 3.183495 | -1.34134 |
| CUST_89993_PI430048170 | 8.99E-06 | -8.44655 | 8.700349 | -1.34043 |
| CUST_138440_PI430048170 | 6.62E-04 | -5.34271 | 2.356973 | -1.33971 |
| CUST_92599_PI430048170 | 8.97E-05 | -6.6551 | 5.197388 | -1.33941 |
| CUST_134993_PI430048170 | 1.46E-02 | -3.53344 | -1.74811 | -1.33923 |
| CUST_130585_PI430048170 | 5.66E-03 | -4.08001 | -0.51181 | -1.33903 |
| CUST_137719_PI430048170 | 3.57E-02 | -2.99804 | -2.91932 | -1.33864 |
| CUST_144532_PI430048170 | 4.46E-05 | -7.17868 | 6.268442 | -1.33798 |
| CUST_143885_PI430048170 | 6.35E-04 | -5.36757 | 2.412557 | -1.3378 |
| CUST_24501_PI430048170 | 2.52E-03 | -4.54515 | 0.550795 | -1.33752 |
| CUST_12758_PI430048170 | 1.65E-03 | -4.79685 | 1.124623 | -1.33631 |
| CUST_137723_PI430048170 | 2.12E-02 | -3.31103 | -2.24131 | -1.33585 |
| CUST_12503_PI430048170 | 6.35E-03 | -4.0157 | -0.65838 | -1.33542 |
| CUST_80737_PI430048170 | 2.77E-03 | -4.49058 | 0.426141 | -1.33403 |
| CUST_82878_PI430048170 | 2.51E-03 | -4.54742 | 0.555986 | -1.33352 |
| CUST_69728_PI430048170 | 1.74E-02 | -3.43036 | -1.97766 | -1.33351 |
| CUST_94475_PI430048170 | 1.60E-03 | -4.81246 | 1.160135 | -1.33309 |
| CUST_72834_PI430048170 | 2.48E-03 | -4.55322 | 0.569233 | -1.33245 |
| CUST_113025_PI430048170 | 1.99E-02 | -3.34723 | -2.16157 | -1.33187 |
| CUST_144066_PI430048170 | 2.12E-03 | -4.64552 | 0.779888 | -1.33102 |
| CUST_103632_PI430048170 | 1.90E-04 | -6.11954 | 4.063312 | -1.33048 |
| CUST_105311_PI430048170 | 2.78E-03 | -4.48766 | 0.419479 | -1.32971 |
| CUST_29956_PI430048170 | 3.39E-05 | -7.3957 | 6.701062 | -1.32958 |
| CUST_98338_PI430048170 | 4.01E-04 | -5.65158 | 3.043229 | -1.32921 |
| CUST_75892_PI430048170 | 3.23E-02 | -3.05926 | -2.78847 | -1.32904 |
| CUST_114393_PI430048170 | 3.84E-02 | -2.95405 | -3.01274 | -1.32876 |
| CUST_67324_PI430048170 | 1.59E-03 | -4.81737 | 1.171296 | -1.32869 |
| CUST_56139_PI430048170 | 4.60E-03 | -4.19867 | -0.24097 | -1.32811 |
| CUST_16602_PI430048170 | 2.54E-02 | -3.20109 | -2.48188 | -1.32751 |
| CUST_15382_PI430048170 | 1.56E-02 | -3.49275 | -1.8389 | -1.32725 |
| CUST_6894_PI430048170 | 2.72E-02 | -3.16113 | -2.56872 | -1.32713 |
| CUST_117765_PI430048170 | 3.62E-03 | -4.33659 | 0.074205 | -1.32683 |
| CUST_81372_PI430048170 | 6.45E-04 | -5.35814 | 2.39148 | -1.32668 |
| CUST_118923_PI430048170 | 4.37E-04 | -5.60096 | 2.931414 | -1.32664 |
| CUST_46774_PI430048170 | 3.13E-04 | -5.80855 | 3.388203 | -1.32649 |
| CUST_1631_PI430048170 | 1.90E-03 | -4.71349 | 0.93485 | -1.32554 |
| CUST_139688_PI430048170 | 8.32E-03 | -3.85949 | -1.01352 | -1.32413 |
| CUST_137099_PI430048170 | 1.68E-02 | -3.45024 | -1.9335 | -1.32398 |
| CUST_134298_PI430048170 | 1.27E-03 | -4.9461 | 1.463579 | -1.32329 |
| CUST_58198_PI430048170 | 4.53E-04 | -5.57945 | 2.883828 | -1.32205 |
| CUST_102958_PI430048170 | 1.69E-03 | -4.78023 | 1.086811 | -1.32196 |
| CUST_122409_PI430048170 | 2.76E-03 | -4.49181 | 0.428955 | -1.32142 |
| CUST_138348_PI430048170 | 2.18E-06 | -9.72037 | 10.91609 | -1.3212 |
| CUST_25746_PI430048170 | 9.34E-03 | -3.79356 | -1.1629 | -1.32106 |
| CUST_120474_PI430048170 | 1.30E-03 | -4.93321 | 1.434341 | -1.31976 |
| CUST_11672_PI430048170 | 2.85E-03 | -4.47136 | 0.382224 | -1.31965 |
| CUST_45771_PI430048170 | 4.28E-03 | -4.24173 | -0.14261 | -1.31945 |
| CUST_95273_PI430048170 | 3.30E-02 | -3.0468 | -2.81519 | -1.31884 |
| CUST_142007_PI430048170 | 2.06E-05 | -7.79436 | 7.478169 | -1.3185 |
| CUST_82543_PI430048170 | 1.03E-02 | -3.73809 | -1.28827 | -1.31848 |
| CUST_138822_PI430048170 | 1.28E-02 | -3.60895 | -1.57901 | -1.31826 |
| CUST_143228_PI430048170 | 3.13E-04 | -5.80862 | 3.388361 | -1.31808 |
| CUST_141471_PI430048170 | 4.59E-07 | -11.1355 | 13.12984 | -1.31788 |
| CUST_119741_PI430048170 | 2.52E-03 | -4.54415 | 0.548512 | -1.31725 |
| CUST_63374_PI430048170 | 7.89E-04 | -5.2352 | 2.116017 | -1.31719 |
| CUST_127766_PI430048170 | 1.04E-02 | -3.73152 | -1.30313 | -1.31673 |
| CUST_26090_PI430048170 | 9.16E-04 | -5.1436 | 1.909955 | -1.31669 |
| CUST_104181_PI430048170 | 6.16E-03 | -4.03252 | -0.62007 | -1.31588 |
| CUST_9710_PI430048170 | 2.07E-03 | -4.6585 | 0.809502 | -1.31536 |
| CUST_45922_PI430048170 | 9.67E-03 | -3.77256 | -1.2104 | -1.31456 |
| CUST_135385_PI430048170 | 1.08E-02 | -3.70779 | -1.35666 | -1.31439 |
| CUST_132265_PI430048170 | 1.57E-03 | -4.82386 | 1.18606 | -1.31439 |
| CUST_13989_PI430048170 | 2.33E-02 | -3.25489 | -2.36446 | -1.31369 |
| CUST_27762_PI430048170 | 1.20E-02 | -3.6481 | -1.49106 | -1.31309 |
| CUST_135386_PI430048170 | 1.39E-02 | -3.55985 | -1.68903 | -1.31285 |
| CUST_752_PI430048170 | 5.85E-05 | -6.97718 | 5.860783 | -1.31235 |
| CUST_85187_PI430048170 | 1.74E-02 | -3.42735 | -1.98434 | -1.312 |
| CUST_130658_PI430048170 | 3.81E-03 | -4.30807 | 0.009011 | -1.31104 |
| CUST_94588_PI430048170 | 1.58E-02 | -3.48553 | -1.85499 | -1.31098 |
| CUST_2056_PI430048170 | 2.74E-03 | -4.49589 | 0.43829 | -1.31094 |
| CUST_119035_PI430048170 | 2.81E-03 | -4.4819 | 0.406307 | -1.31082 |
| CUST_55767_PI430048170 | 1.79E-04 | -6.16747 | 4.166323 | -1.31038 |
| CUST_21808_PI430048170 | 6.55E-03 | -3.99585 | -0.70359 | -1.30967 |
| CUST_145436_PI430048170 | 7.80E-03 | -3.89677 | -0.9289 | -1.30961 |
| CUST_65103_PI430048170 | 1.36E-03 | -4.90652 | 1.373802 | -1.30949 |
| CUST_32853_PI430048170 | 2.98E-03 | -4.44632 | 0.325014 | -1.30913 |
| CUST_63999_PI430048170 | 2.43E-02 | -3.22911 | -2.42079 | -1.3089 |
| CUST_88707_PI430048170 | 4.48E-03 | -4.21288 | -0.2085 | -1.30876 |
| CUST_64101_PI430048170 | 8.87E-03 | -3.82391 | -1.09418 | -1.30856 |
| CUST_46470_PI430048170 | 3.04E-03 | -4.43518 | 0.29954 | -1.30821 |
| CUST_48312_PI430048170 | 4.03E-02 | -2.92343 | -3.07747 | -1.30799 |
| CUST_135315_PI430048170 | 8.42E-03 | -3.85256 | -1.02924 | -1.30788 |
| CUST_66778_PI430048170 | 4.98E-02 | -2.78846 | -3.3595 | -1.30739 |
| CUST_122696_PI430048170 | 1.26E-03 | -4.95534 | 1.484516 | -1.30601 |
| CUST_121536_PI430048170 | 4.63E-03 | -4.19468 | -0.25008 | -1.30548 |
| CUST_137741_PI430048170 | 2.54E-06 | -9.56329 | 10.65471 | -1.30543 |
| CUST_90287_PI430048170 | 4.96E-02 | -2.7915 | -3.3532 | -1.30524 |
| CUST_58636_PI430048170 | 2.21E-03 | -4.62208 | 0.726421 | -1.30485 |
| CUST_80565_PI430048170 | 4.18E-03 | -4.25477 | -0.11281 | -1.3042 |
| CUST_131075_PI430048170 | 9.57E-03 | -3.77944 | -1.19484 | -1.30418 |
| CUST_98662_PI430048170 | 5.36E-03 | -4.10977 | -0.44392 | -1.30341 |
| CUST_17691_PI430048170 | 4.24E-03 | -4.24659 | -0.1315 | -1.30324 |
| CUST_123025_PI430048170 | 1.78E-02 | -3.41709 | -2.00709 | -1.30242 |
| CUST_83971_PI430048170 | 1.41E-02 | -3.55343 | -1.7034 | -1.30214 |
| CUST_128568_PI430048170 | 1.58E-02 | -3.4836 | -1.85929 | -1.30195 |
| CUST_86803_PI430048170 | 9.29E-03 | -3.79715 | -1.15477 | -1.30122 |
| CUST_133336_PI430048170 | 5.79E-04 | -5.4247 | 2.540049 | -1.30108 |
| CUST_13006_PI430048170 | 1.43E-03 | -4.87853 | 1.310279 | -1.3009 |
| CUST_125642_PI430048170 | 9.88E-03 | -3.7609 | -1.23675 | -1.30011 |
| CUST_145704_PI430048170 | 9.22E-05 | -6.63062 | 5.146382 | -1.29988 |
| CUST_100287_PI430048170 | 1.54E-02 | -3.49993 | -1.82289 | -1.29919 |
| CUST_125978_PI430048170 | 4.25E-03 | -4.24492 | -0.13531 | -1.299 |
| CUST_126105_PI430048170 | 1.31E-02 | -3.59564 | -1.60887 | -1.29882 |
| CUST_109360_PI430048170 | 1.41E-04 | -6.32443 | 4.501584 | -1.29881 |
| CUST_35774_PI430048170 | 1.89E-02 | -3.38 | -2.08922 | -1.29881 |
| CUST_88694_PI430048170 | 2.12E-04 | -6.0455 | 3.903633 | -1.29876 |
| CUST_137482_PI430048170 | 2.54E-04 | -5.93608 | 3.666452 | -1.29851 |
| CUST_133916_PI430048170 | 5.32E-03 | -4.11378 | -0.43478 | -1.29796 |
| CUST_125453_PI430048170 | 2.47E-04 | -5.95285 | 3.702881 | -1.29771 |
| CUST_126056_PI430048170 | 9.60E-05 | -6.60301 | 5.088735 | -1.29708 |
| CUST_57318_PI430048170 | 2.42E-02 | -3.23171 | -2.41513 | -1.2967 |
| CUST_118827_PI430048170 | 1.17E-04 | -6.45288 | 4.773596 | -1.29644 |
| CUST_67444_PI430048170 | 3.61E-03 | -4.33712 | 0.075409 | -1.29596 |
| CUST_50815_PI430048170 | 8.42E-03 | -3.85198 | -1.03055 | -1.29446 |
| CUST_140402_PI430048170 | 1.12E-03 | -5.02586 | 1.644156 | -1.29393 |
| CUST_105279_PI430048170 | 1.42E-04 | -6.32011 | 4.492398 | -1.2935 |
| CUST_136062_PI430048170 | 2.65E-03 | -4.51553 | 0.48315 | -1.29262 |
| CUST_144380_PI430048170 | 1.68E-05 | -7.9384 | 7.75333 | -1.29242 |
| CUST_134797_PI430048170 | 7.47E-04 | -5.26742 | 2.188332 | -1.29206 |
| CUST_80795_PI430048170 | 8.46E-03 | -3.84988 | -1.03532 | -1.29114 |
| CUST_43890_PI430048170 | 6.59E-03 | -3.99244 | -0.71137 | -1.29036 |
| CUST_47920_PI430048170 | 1.08E-02 | -3.71059 | -1.35033 | -1.2898 |
| CUST_98140_PI430048170 | 1.70E-03 | -4.77878 | 1.083512 | -1.28978 |
| CUST_109084_PI430048170 | 8.92E-03 | -3.82085 | -1.10111 | -1.28948 |
| CUST_54565_PI430048170 | 4.33E-03 | -4.23409 | -0.16006 | -1.2892 |
| CUST_132953_PI430048170 | 9.57E-03 | -3.7797 | -1.19424 | -1.28759 |
| CUST_127979_PI430048170 | 2.27E-02 | -3.26998 | -2.33141 | -1.28737 |
| CUST_7683_PI430048170 | 9.80E-03 | -3.76501 | -1.22747 | -1.28702 |
| CUST_19316_PI430048170 | 1.39E-02 | -3.55877 | -1.69147 | -1.28685 |
| CUST_83516_PI430048170 | 2.34E-02 | -3.25163 | -2.37159 | -1.28639 |
| CUST_39505_PI430048170 | 6.05E-04 | -5.39659 | 2.477357 | -1.28628 |
| CUST_103561_PI430048170 | 3.15E-02 | -3.07415 | -2.75651 | -1.28605 |
| CUST_138846_PI430048170 | 4.07E-05 | -7.25174 | 6.414841 | -1.28596 |
| CUST_139416_PI430048170 | 6.98E-04 | -5.30716 | 2.277397 | -1.28569 |
| CUST_83525_PI430048170 | 4.56E-02 | -2.8449 | -3.24224 | -1.28409 |
| CUST_122011_PI430048170 | 1.81E-04 | -6.16121 | 4.152868 | -1.2834 |
| CUST_11362_PI430048170 | 6.02E-03 | -4.04584 | -0.58973 | -1.28221 |
| CUST_119298_PI430048170 | 2.02E-03 | -4.67427 | 0.845464 | -1.28197 |
| CUST_16603_PI430048170 | 2.27E-02 | -3.26983 | -2.33174 | -1.28108 |
| CUST_133491_PI430048170 | 4.32E-03 | -4.23619 | -0.15526 | -1.28096 |
| CUST_55016_PI430048170 | 1.80E-02 | -3.40803 | -2.02717 | -1.2803 |
| CUST_83088_PI430048170 | 6.75E-04 | -5.33002 | 2.32858 | -1.27973 |
| CUST_134639_PI430048170 | 4.61E-02 | -2.83714 | -3.2584 | -1.27924 |
| CUST_27608_PI430048170 | 1.16E-03 | -5.00445 | 1.595735 | -1.27876 |
| CUST_29041_PI430048170 | 1.14E-03 | -5.01179 | 1.612341 | -1.27839 |
| CUST_103421_PI430048170 | 6.17E-03 | -4.03065 | -0.62433 | -1.27827 |
| CUST_28098_PI430048170 | 3.20E-03 | -4.40662 | 0.23428 | -1.27794 |
| CUST_17197_PI430048170 | 3.03E-02 | -3.09876 | -2.70355 | -1.27758 |
| CUST_56498_PI430048170 | 1.26E-02 | -3.61897 | -1.55652 | -1.27731 |
| CUST_73281_PI430048170 | 3.37E-03 | -4.37722 | 0.167068 | -1.27718 |
| CUST_142205_PI430048170 | 1.37E-04 | -6.34663 | 4.548763 | -1.27659 |
| CUST_98884_PI430048170 | 2.25E-05 | -7.72611 | 7.346745 | -1.27653 |
| CUST_24850_PI430048170 | 1.58E-02 | -3.48247 | -1.86181 | -1.27649 |
| CUST_24924_PI430048170 | 1.30E-02 | -3.60092 | -1.59701 | -1.2762 |
| CUST_26596_PI430048170 | 3.08E-03 | -4.42786 | 0.282826 | -1.27571 |
| CUST_12173_PI430048170 | 1.06E-03 | -5.05455 | 1.709013 | -1.27564 |
| CUST_29904_PI430048170 | 1.65E-02 | -3.45881 | -1.91445 | -1.2753 |
| CUST_74574_PI430048170 | 1.04E-02 | -3.73241 | -1.30111 | -1.27524 |
| CUST_82590_PI430048170 | 6.81E-03 | -3.97357 | -0.7543 | -1.27481 |
| CUST_47268_PI430048170 | 4.21E-03 | -4.25012 | -0.12344 | -1.27477 |
| CUST_54952_PI430048170 | 5.65E-04 | -5.44175 | 2.578047 | -1.27434 |
| CUST_87458_PI430048170 | 2.56E-02 | -3.19718 | -2.4904 | -1.27427 |
| CUST_28872_PI430048170 | 2.14E-02 | -3.30502 | -2.25452 | -1.27377 |
| CUST_55287_PI430048170 | 1.16E-02 | -3.66536 | -1.45224 | -1.27345 |
| CUST_142899_PI430048170 | 2.84E-03 | -4.47469 | 0.389842 | -1.27344 |
| CUST_123521_PI430048170 | 2.65E-03 | -4.5133 | 0.478061 | -1.27326 |
| CUST_136447_PI430048170 | 9.72E-04 | -5.10946 | 1.832988 | -1.27263 |
| CUST_142945_PI430048170 | 4.08E-02 | -2.91566 | -3.09385 | -1.27229 |
| CUST_15551_PI430048170 | 2.44E-02 | -3.22625 | -2.42704 | -1.27212 |
| CUST_128737_PI430048170 | 1.25E-02 | -3.62045 | -1.5532 | -1.27199 |
| CUST_48084_PI430048170 | 8.17E-05 | -6.72311 | 5.33866 | -1.2712 |
| CUST_78747_PI430048170 | 3.33E-04 | -5.77275 | 3.309762 | -1.27107 |
| CUST_109_PI430053867 | 3.27E-03 | -4.39417 | 0.205813 | -1.2699 |
| CUST_78836_PI430048170 | 3.02E-03 | -4.43984 | 0.310206 | -1.26981 |
| CUST_73574_PI430048170 | 2.84E-03 | -4.47494 | 0.390414 | -1.26951 |
| CUST_103396_PI430048170 | 5.51E-04 | -5.45814 | 2.614529 | -1.2691 |
| CUST_129804_PI430048170 | 3.66E-04 | -5.71201 | 3.176367 | -1.26855 |
| CUST_143051_PI430048170 | 1.03E-03 | -5.07063 | 1.745354 | -1.26837 |
| CUST_113122_PI430048170 | 9.56E-03 | -3.77997 | -1.19364 | -1.26833 |
| CUST_120014_PI430048170 | 2.45E-02 | -3.22393 | -2.43211 | -1.26769 |
| CUST_100983_PI430048170 | 1.88E-02 | -3.38309 | -2.08239 | -1.26719 |
| CUST_55052_PI430048170 | 1.39E-02 | -3.56099 | -1.68649 | -1.26688 |
| CUST_132429_PI430048170 | 2.83E-03 | -4.47737 | 0.39596 | -1.26509 |
| CUST_40_PI430048170 | 1.42E-02 | -3.54929 | -1.71266 | -1.26418 |
| CUST_63924_PI430048170 | 3.92E-04 | -5.66715 | 3.077571 | -1.26362 |
| CUST_142641_PI430048170 | 2.52E-03 | -4.54391 | 0.547968 | -1.26342 |
| CUST_144741_PI430048170 | 2.50E-02 | -3.2101 | -2.46226 | -1.26334 |
| CUST_32771_PI430048170 | 1.54E-02 | -3.50006 | -1.8226 | -1.26328 |
| CUST_71647_PI430048170 | 3.16E-02 | -3.07274 | -2.75952 | -1.2625 |
| CUST_55612_PI430048170 | 5.22E-03 | -4.12456 | -0.41018 | -1.26217 |
| CUST_65949_PI430048170 | 2.23E-04 | -6.01171 | 3.830532 | -1.26113 |
| CUST_22358_PI430048170 | 1.32E-03 | -4.92671 | 1.419619 | -1.26101 |
| CUST_93291_PI430048170 | 4.21E-03 | -4.25018 | -0.1233 | -1.26085 |
| CUST_143617_PI430048170 | 8.34E-04 | -5.20011 | 2.037167 | -1.26056 |
| CUST_106498_PI430048170 | 1.63E-04 | -6.22894 | 4.297989 | -1.26033 |
| CUST_127978_PI430048170 | 5.72E-03 | -4.07401 | -0.5255 | -1.26031 |
| CUST_113029_PI430048170 | 1.08E-02 | -3.70884 | -1.35429 | -1.25941 |
| CUST_99872_PI430048170 | 1.97E-02 | -3.35471 | -2.14509 | -1.25881 |
| CUST_44077_PI430048170 | 8.33E-03 | -3.85838 | -1.01604 | -1.25839 |
| CUST_145716_PI430048170 | 7.02E-04 | -5.30326 | 2.268678 | -1.2576 |
| CUST_24133_PI430048170 | 3.61E-03 | -4.33768 | 0.076683 | -1.25741 |
| CUST_58596_PI430048170 | 1.05E-02 | -3.72314 | -1.32203 | -1.25612 |
| CUST_24885_PI430048170 | 5.59E-03 | -4.08673 | -0.49649 | -1.25603 |
| CUST_134476_PI430048170 | 9.80E-03 | -3.76534 | -1.22673 | -1.25564 |
| CUST_109333_PI430048170 | 8.22E-04 | -5.20912 | 2.057413 | -1.25563 |
| CUST_134442_PI430048170 | 1.68E-02 | -3.44928 | -1.93563 | -1.25466 |
| CUST_58441_PI430048170 | 1.21E-03 | -4.9753 | 1.529735 | -1.25461 |
| CUST_46538_PI430048170 | 3.04E-03 | -4.43507 | 0.299293 | -1.2545 |
| CUST_19759_PI430048170 | 6.30E-03 | -4.01977 | -0.64913 | -1.2542 |
| CUST_30826_PI430048170 | 1.80E-02 | -3.4095 | -2.02391 | -1.25279 |
| CUST_90145_PI430048170 | 2.89E-02 | -3.12598 | -2.64481 | -1.25266 |
| CUST_130186_PI430048170 | 3.94E-04 | -5.66384 | 3.070281 | -1.25247 |
| CUST_7384_PI430048170 | 4.22E-03 | -4.24824 | -0.12773 | -1.25244 |
| CUST_56832_PI430048170 | 4.04E-02 | -2.92241 | -3.07962 | -1.25203 |
| CUST_13648_PI430048170 | 1.76E-02 | -3.42148 | -1.99736 | -1.25158 |
| CUST_129167_PI430048170 | 2.54E-04 | -5.93645 | 3.667239 | -1.25107 |
| CUST_57252_PI430048170 | 1.99E-03 | -4.68306 | 0.865496 | -1.25091 |
| CUST_36147_PI430048170 | 1.04E-03 | -5.06887 | 1.741374 | -1.25088 |
| CUST_132761_PI430048170 | 1.34E-04 | -6.36517 | 4.58809 | -1.25078 |
| CUST_25779_PI430048170 | 4.26E-03 | -4.24318 | -0.13927 | -1.25069 |
| CUST_39624_PI430048170 | 2.83E-03 | -4.47627 | 0.39344 | -1.25007 |
| CUST_122311_PI430048170 | 1.31E-02 | -3.59263 | -1.61562 | -1.24978 |
| CUST_63796_PI430048170 | 1.12E-02 | -3.68529 | -1.40736 | -1.24956 |
| CUST_32582_PI430048170 | 9.35E-04 | -5.13215 | 1.884161 | -1.24948 |
| CUST_137868_PI430048170 | 1.36E-04 | -6.35019 | 4.556319 | -1.24876 |
| CUST_132264_PI430048170 | 7.94E-04 | -5.2317 | 2.108146 | -1.24779 |
| CUST_114703_PI430048170 | 4.21E-04 | -5.62267 | 2.979406 | -1.24644 |
| CUST_125898_PI430048170 | 1.30E-02 | -3.59844 | -1.60258 | -1.24607 |
| CUST_143024_PI430048170 | 8.92E-04 | -5.15931 | 1.945354 | -1.24537 |
| CUST_127391_PI430048170 | 1.72E-02 | -3.43482 | -1.96775 | -1.24523 |
| CUST_68169_PI430048170 | 8.24E-04 | -5.2071 | 2.052869 | -1.24487 |
| CUST_56159_PI430048170 | 8.19E-03 | -3.86814 | -0.99389 | -1.24429 |
| CUST_93415_PI430048170 | 1.44E-02 | -3.54172 | -1.7296 | -1.24391 |
| CUST_118479_PI430048170 | 5.85E-05 | -6.97772 | 5.861895 | -1.2434 |
| CUST_13993_PI430048170 | 2.81E-03 | -4.48015 | 0.402323 | -1.2431 |
| CUST_128556_PI430048170 | 2.95E-04 | -5.84264 | 3.462772 | -1.24268 |
| CUST_119743_PI430048170 | 2.24E-03 | -4.61461 | 0.709363 | -1.24228 |
| CUST_135450_PI430048170 | 2.07E-02 | -3.32522 | -2.21008 | -1.24192 |
| CUST_127914_PI430048170 | 6.17E-03 | -4.03066 | -0.62432 | -1.24134 |
| CUST_119107_PI430048170 | 7.79E-03 | -3.89724 | -0.92784 | -1.24093 |
| CUST_11096_PI430048170 | 4.53E-02 | -2.84927 | -3.23311 | -1.24004 |
| CUST_134633_PI430048170 | 1.85E-02 | -3.39302 | -2.06041 | -1.23973 |
| CUST_142815_PI430048170 | 2.25E-04 | -6.00875 | 3.824131 | -1.23876 |
| CUST_115568_PI430048170 | 4.58E-03 | -4.20176 | -0.2339 | -1.23836 |
| CUST_115028_PI430048170 | 6.26E-03 | -4.02331 | -0.64106 | -1.23829 |
| CUST_114885_PI430048170 | 1.17E-02 | -3.6636 | -1.4562 | -1.2376 |
| CUST_75279_PI430048170 | 2.44E-02 | -3.2252 | -2.42934 | -1.23747 |
| CUST_111307_PI430048170 | 4.35E-02 | -2.87544 | -3.17837 | -1.23638 |
| CUST_130724_PI430048170 | 4.75E-03 | -4.18078 | -0.28182 | -1.23632 |
| CUST_137294_PI430048170 | 6.27E-05 | -6.9146 | 5.733007 | -1.23592 |
| CUST_58214_PI430048170 | 7.69E-03 | -3.90452 | -0.9113 | -1.23572 |
| CUST_103412_PI430048170 | 4.57E-03 | -4.20344 | -0.23008 | -1.23537 |
| CUST_51606_PI430048170 | 5.70E-03 | -4.07622 | -0.52047 | -1.23514 |
| CUST_65430_PI430048170 | 4.74E-03 | -4.18198 | -0.2791 | -1.23503 |
| CUST_113450_PI430048170 | 5.31E-03 | -4.11511 | -0.43174 | -1.23408 |
| CUST_118382_PI430048170 | 6.00E-03 | -4.04706 | -0.58694 | -1.23374 |
| CUST_121101_PI430048170 | 2.76E-02 | -3.15263 | -2.58714 | -1.2333 |
| CUST_136683_PI430048170 | 2.11E-02 | -3.31407 | -2.23462 | -1.23321 |
| CUST_35408_PI430048170 | 2.22E-02 | -3.28247 | -2.30402 | -1.23319 |
| CUST_143020_PI430048170 | 4.83E-03 | -4.17189 | -0.30213 | -1.23299 |
| CUST_34007_PI430048170 | 2.24E-02 | -3.27802 | -2.31379 | -1.2329 |
| CUST_125860_PI430048170 | 1.35E-04 | -6.35795 | 4.572795 | -1.2325 |
| CUST_58183_PI430048170 | 1.74E-03 | -4.76367 | 1.049136 | -1.23203 |
| CUST_97660_PI430048170 | 4.87E-03 | -4.16721 | -0.31281 | -1.23175 |
| CUST_128270_PI430048170 | 2.32E-02 | -3.25737 | -2.35902 | -1.23132 |
| CUST_28_PI430048170 | 1.35E-04 | -6.35916 | 4.575359 | -1.23037 |
| CUST_42528_PI430048170 | 2.25E-02 | -3.27523 | -2.31989 | -1.23014 |
| CUST_118554_PI430048170 | 3.26E-02 | -3.05472 | -2.79822 | -1.22994 |
| CUST_139706_PI430048170 | 3.02E-02 | -3.09978 | -2.70135 | -1.22991 |
| CUST_145502_PI430048170 | 4.02E-03 | -4.27629 | -0.06362 | -1.22986 |
| CUST_45035_PI430048170 | 8.15E-04 | -5.21491 | 2.070432 | -1.22959 |
| CUST_140529_PI430048170 | 3.72E-02 | -2.97415 | -2.97011 | -1.22939 |
| CUST_58107_PI430048170 | 2.37E-02 | -3.24292 | -2.39064 | -1.22885 |
| CUST_127179_PI430048170 | 1.59E-04 | -6.24406 | 4.330316 | -1.22874 |
| CUST_113221_PI430048170 | 4.75E-03 | -4.18015 | -0.28326 | -1.22851 |
| CUST_137161_PI430048170 | 4.30E-02 | -2.88245 | -3.16367 | -1.22842 |
| CUST_133076_PI430048170 | 5.41E-03 | -4.10418 | -0.45667 | -1.22837 |
| CUST_87439_PI430048170 | 4.84E-03 | -4.17011 | -0.30619 | -1.22812 |
| CUST_50109_PI430048170 | 3.61E-05 | -7.33972 | 6.590113 | -1.22808 |
| CUST_18757_PI430048170 | 2.12E-03 | -4.64561 | 0.780105 | -1.22808 |
| CUST_5915_PI430048170 | 2.82E-02 | -3.14108 | -2.61216 | -1.22774 |
| CUST_127812_PI430048170 | 2.64E-03 | -4.51746 | 0.487567 | -1.22627 |
| CUST_130909_PI430048170 | 1.32E-03 | -4.92472 | 1.415086 | -1.22626 |
| CUST_19074_PI430048170 | 3.66E-04 | -5.70997 | 3.171882 | -1.22619 |
| CUST_24447_PI430048170 | 6.91E-03 | -3.96494 | -0.77395 | -1.22601 |
| CUST_130824_PI430048170 | 2.91E-03 | -4.45964 | 0.355441 | -1.22585 |
| CUST_141906_PI430048170 | 2.85E-02 | -3.13452 | -2.62635 | -1.22516 |
| CUST_9684_PI430048170 | 1.01E-04 | -6.56043 | 4.999678 | -1.225 |
| CUST_120827_PI430048170 | 5.10E-03 | -4.13849 | -0.37839 | -1.22463 |
| CUST_80858_PI430048170 | 1.24E-03 | -4.96253 | 1.500802 | -1.22414 |
| CUST_19553_PI430048170 | 2.39E-02 | -3.23835 | -2.40062 | -1.2237 |
| CUST_5021_PI430048170 | 1.15E-03 | -5.00767 | 1.603015 | -1.22368 |
| CUST_72897_PI430048170 | 1.34E-03 | -4.91735 | 1.398385 | -1.22354 |
| CUST_84270_PI430048170 | 1.76E-02 | -3.42324 | -1.99345 | -1.22348 |
| CUST_49697_PI430048170 | 8.17E-03 | -3.86982 | -0.99008 | -1.22266 |
| CUST_73367_PI430048170 | 9.88E-04 | -5.09942 | 1.810341 | -1.22256 |
| CUST_138709_PI430048170 | 3.17E-04 | -5.80283 | 3.375681 | -1.22204 |
| CUST_99632_PI430048170 | 5.13E-03 | -4.13511 | -0.3861 | -1.22171 |
| CUST_73369_PI430048170 | 7.62E-04 | -5.25455 | 2.159448 | -1.22135 |
| CUST_143870_PI430048170 | 4.79E-04 | -5.54377 | 2.804782 | -1.22126 |
| CUST_93161_PI430048170 | 1.71E-02 | -3.44065 | -1.95481 | -1.22022 |
| CUST_141105_PI430048170 | 2.82E-03 | -4.47807 | 0.397556 | -1.21964 |
| CUST_140768_PI430048170 | 4.38E-03 | -4.22816 | -0.17361 | -1.21962 |
| CUST_102506_PI430048170 | 2.32E-03 | -4.59372 | 0.6617 | -1.21927 |
| CUST_124796_PI430048170 | 3.50E-04 | -5.74312 | 3.244739 | -1.21926 |
| CUST_134243_PI430048170 | 2.81E-02 | -3.14236 | -2.60939 | -1.21923 |
| CUST_142417_PI430048170 | 2.12E-04 | -6.04514 | 3.902856 | -1.21901 |
| CUST_132942_PI430048170 | 1.10E-03 | -5.03207 | 1.658203 | -1.21831 |
| CUST_9473_PI430048170 | 3.73E-02 | -2.97245 | -2.97373 | -1.21779 |
| CUST_89158_PI430048170 | 1.45E-02 | -3.53416 | -1.74649 | -1.21778 |
| CUST_141962_PI430048170 | 6.00E-04 | -5.40194 | 2.489294 | -1.21772 |
| CUST_133754_PI430048170 | 1.86E-04 | -6.13508 | 4.096747 | -1.2174 |
| CUST_74883_PI430048170 | 5.71E-06 | -8.85554 | 9.436052 | -1.21734 |
| CUST_136413_PI430048170 | 2.63E-03 | -4.51864 | 0.490243 | -1.21649 |
| CUST_92667_PI430048170 | 6.32E-03 | -4.0185 | -0.65202 | -1.21576 |
| CUST_63090_PI430048170 | 4.09E-03 | -4.2667 | -0.08553 | -1.21569 |
| CUST_82067_PI430048170 | 3.64E-03 | -4.3334 | 0.066911 | -1.21565 |
| CUST_122785_PI430048170 | 7.80E-03 | -3.89609 | -0.93046 | -1.21542 |
| CUST_127449_PI430048170 | 6.88E-03 | -3.96753 | -0.76805 | -1.21536 |
| CUST_12627_PI430048170 | 1.11E-02 | -3.69179 | -1.39272 | -1.21535 |
| CUST_66233_PI430048170 | 2.04E-02 | -3.3317 | -2.1958 | -1.21532 |
| CUST_93324_PI430048170 | 2.03E-04 | -6.07057 | 3.957777 | -1.2147 |
| CUST_16737_PI430048170 | 8.25E-03 | -3.86363 | -1.00412 | -1.21422 |
| CUST_141437_PI430048170 | 2.58E-04 | -5.92627 | 3.645098 | -1.21419 |
| CUST_144481_PI430048170 | 3.80E-03 | -4.30899 | 0.011121 | -1.21412 |
| CUST_55636_PI430048170 | 1.57E-03 | -4.82683 | 1.192814 | -1.21364 |
| CUST_125722_PI430048170 | 2.14E-03 | -4.64063 | 0.768744 | -1.21343 |
| CUST_51278_PI430048170 | 2.71E-03 | -4.50207 | 0.452399 | -1.21274 |
| CUST_92594_PI430048170 | 2.01E-02 | -3.34266 | -2.17166 | -1.21238 |
| CUST_37862_PI430048170 | 1.14E-02 | -3.67625 | -1.42772 | -1.21215 |
| CUST_46045_PI430048170 | 5.01E-03 | -4.14873 | -0.35502 | -1.21197 |
| CUST_21864_PI430048170 | 6.69E-03 | -3.98389 | -0.73083 | -1.21176 |
| CUST_66958_PI430048170 | 1.96E-04 | -6.0943 | 4.008961 | -1.21165 |
| CUST_145533_PI430048170 | 6.99E-04 | -5.30615 | 2.27514 | -1.21147 |
| CUST_40033_PI430048170 | 4.89E-02 | -2.80065 | -3.33424 | -1.21141 |
| CUST_24241_PI430048170 | 3.47E-02 | -3.01564 | -2.8818 | -1.21123 |
| CUST_8007_PI430048170 | 3.50E-02 | -3.01023 | -2.89335 | -1.21117 |
| CUST_18943_PI430048170 | 2.09E-04 | -6.05285 | 3.919522 | -1.21051 |
| CUST_129676_PI430048170 | 1.36E-02 | -3.57439 | -1.6565 | -1.21049 |
| CUST_138863_PI430048170 | 7.34E-03 | -3.93032 | -0.85269 | -1.21041 |
| CUST_84208_PI430048170 | 3.74E-03 | -4.31837 | 0.032556 | -1.21018 |
| CUST_80548_PI430048170 | 6.76E-03 | -3.97776 | -0.74477 | -1.21013 |
| CUST_29508_PI430048170 | 1.51E-03 | -4.84789 | 1.240689 | -1.21012 |
| CUST_94667_PI430048170 | 4.02E-02 | -2.92581 | -3.07246 | -1.20938 |
| CUST_12625_PI430048170 | 1.60E-02 | -3.47752 | -1.87282 | -1.209 |
| CUST_20324_PI430048170 | 1.04E-02 | -3.73092 | -1.30446 | -1.20893 |
| CUST_111301_PI430048170 | 2.54E-04 | -5.9348 | 3.66365 | -1.20873 |
| CUST_142323_PI430048170 | 8.68E-04 | -5.17704 | 1.985261 | -1.20846 |
| CUST_140913_PI430048170 | 6.89E-03 | -3.96676 | -0.7698 | -1.20817 |
| CUST_21535_PI430048170 | 4.45E-03 | -4.21739 | -0.1982 | -1.20737 |
| CUST_128642_PI430048170 | 4.16E-03 | -4.25683 | -0.1081 | -1.20727 |
| CUST_123232_PI430048170 | 2.23E-02 | -3.2795 | -2.31054 | -1.20706 |
| CUST_128305_PI430048170 | 1.46E-02 | -3.53223 | -1.7508 | -1.20682 |
| CUST_110369_PI430048170 | 1.06E-02 | -3.71934 | -1.3306 | -1.20657 |
| CUST_135473_PI430048170 | 4.74E-05 | -7.13657 | 6.183723 | -1.20623 |
| CUST_30303_PI430048170 | 1.85E-04 | -6.14234 | 4.112338 | -1.20604 |
| CUST_35873_PI430048170 | 2.10E-03 | -4.65121 | 0.792857 | -1.20557 |
| CUST_121595_PI430048170 | 1.95E-02 | -3.36112 | -2.13092 | -1.20547 |
| CUST_136787_PI430048170 | 6.27E-04 | -5.37546 | 2.430181 | -1.20545 |
| CUST_61587_PI430048170 | 2.00E-03 | -4.67969 | 0.857804 | -1.20495 |
| CUST_97096_PI430048170 | 7.28E-05 | -6.80217 | 5.502097 | -1.20378 |
| CUST_139147_PI430048170 | 1.14E-04 | -6.47613 | 4.822609 | -1.20365 |
| CUST_145596_PI430048170 | 7.56E-04 | -5.26013 | 2.171976 | -1.20338 |
| CUST_127173_PI430048170 | 3.56E-03 | -4.34678 | 0.097492 | -1.20223 |
| CUST_960_PI430048170 | 8.01E-03 | -3.88143 | -0.96374 | -1.20196 |
| CUST_49212_PI430048170 | 3.67E-03 | -4.32891 | 0.056643 | -1.20194 |
| CUST_142761_PI430048170 | 4.25E-03 | -4.24512 | -0.13486 | -1.20151 |
| CUST_82182_PI430048170 | 1.26E-02 | -3.6191 | -1.55623 | -1.20048 |
| CUST_43468_PI430048170 | 2.49E-03 | -4.55212 | 0.566719 | -1.2003 |
| CUST_7309_PI430048170 | 6.79E-03 | -3.9755 | -0.74991 | -1.20001 |
| CUST_92481_PI430048170 | 4.40E-03 | -4.22382 | -0.18352 | -1.19951 |
| CUST_25872_PI430048170 | 5.16E-03 | -4.13214 | -0.39288 | -1.19931 |
| CUST_54174_PI430048170 | 9.89E-03 | -3.75939 | -1.24017 | -1.19904 |
| CUST_88375_PI430048170 | 2.17E-03 | -4.63371 | 0.752942 | -1.19884 |
| CUST_113639_PI430048170 | 6.05E-03 | -4.04221 | -0.598 | -1.19854 |
| CUST_53013_PI430048170 | 6.38E-03 | -4.01263 | -0.66538 | -1.19807 |
| CUST_130326_PI430048170 | 2.04E-02 | -3.33325 | -2.1924 | -1.19789 |
| CUST_128072_PI430048170 | 3.21E-02 | -3.06335 | -2.77969 | -1.19737 |
| CUST_24246_PI430048170 | 9.88E-03 | -3.76015 | -1.23844 | -1.19641 |
| CUST_17935_PI430048170 | 5.90E-03 | -4.05608 | -0.56638 | -1.19624 |
| CUST_137901_PI430048170 | 1.17E-03 | -4.99824 | 1.58168 | -1.1962 |
| CUST_66180_PI430048170 | 1.33E-03 | -4.92089 | 1.406412 | -1.19618 |
| CUST_87386_PI430048170 | 3.55E-02 | -3.00162 | -2.91169 | -1.19603 |
| CUST_20326_PI430048170 | 3.90E-02 | -2.94397 | -3.03407 | -1.19558 |
| CUST_94245_PI430048170 | 7.24E-03 | -3.93809 | -0.83501 | -1.19513 |
| CUST_42025_PI430048170 | 1.76E-04 | -6.17665 | 4.186008 | -1.19453 |
| CUST_97647_PI430048170 | 4.88E-03 | -4.16494 | -0.318 | -1.1939 |
| CUST_28496_PI430048170 | 2.41E-04 | -5.96816 | 3.73614 | -1.19297 |
| CUST_69354_PI430048170 | 2.09E-04 | -6.05459 | 3.923276 | -1.1924 |
| CUST_113730_PI430048170 | 2.88E-04 | -5.85909 | 3.498701 | -1.19221 |
| CUST_92934_PI430048170 | 1.54E-02 | -3.49864 | -1.82577 | -1.19194 |
| CUST_107030_PI430048170 | 2.19E-03 | -4.62671 | 0.736986 | -1.19188 |
| CUST_104467_PI430048170 | 5.43E-03 | -4.10316 | -0.459 | -1.19087 |
| CUST_140999_PI430048170 | 1.13E-02 | -3.68107 | -1.41687 | -1.18933 |
| CUST_66653_PI430048170 | 2.63E-04 | -5.91331 | 3.61691 | -1.18895 |
| CUST_18646_PI430048170 | 3.47E-02 | -3.01565 | -2.88179 | -1.18891 |
| CUST_135233_PI430048170 | 4.59E-03 | -4.20053 | -0.23673 | -1.18886 |
| CUST_136417_PI430048170 | 1.76E-02 | -3.42147 | -1.99737 | -1.18751 |
| CUST_40764_PI430048170 | 2.15E-02 | -3.30352 | -2.25781 | -1.18733 |
| CUST_68541_PI430048170 | 3.13E-04 | -5.80942 | 3.390111 | -1.18615 |
| CUST_5630_PI430048170 | 8.62E-04 | -5.18106 | 1.994296 | -1.18587 |
| CUST_122653_PI430048170 | 1.36E-02 | -3.57172 | -1.66248 | -1.18571 |
| CUST_63392_PI430048170 | 1.93E-04 | -6.10816 | 4.038809 | -1.18535 |
| CUST_69487_PI430048170 | 2.52E-02 | -3.20654 | -2.47003 | -1.1847 |
| CUST_50867_PI430048170 | 5.63E-04 | -5.44419 | 2.583468 | -1.18426 |
| CUST_126907_PI430048170 | 8.87E-06 | -8.46155 | 8.727743 | -1.18405 |
| CUST_37000_PI430048170 | 2.16E-02 | -3.30052 | -2.2644 | -1.18333 |
| CUST_53404_PI430048170 | 4.02E-02 | -2.92561 | -3.07287 | -1.18311 |
| CUST_34601_PI430048170 | 1.83E-04 | -6.15002 | 4.128855 | -1.1823 |
| CUST_45337_PI430048170 | 2.29E-04 | -5.99568 | 3.795825 | -1.18226 |
| CUST_57162_PI430048170 | 6.00E-04 | -5.40226 | 2.489999 | -1.18218 |
| CUST_141412_PI430048170 | 2.42E-02 | -3.2303 | -2.41821 | -1.18191 |
| CUST_140001_PI430048170 | 2.57E-02 | -3.1954 | -2.49428 | -1.18181 |
| CUST_51302_PI430048170 | 3.31E-03 | -4.38821 | 0.19219 | -1.18155 |
| CUST_118910_PI430048170 | 7.97E-03 | -3.8847 | -0.95631 | -1.18138 |
| CUST_24668_PI430048170 | 1.04E-03 | -5.06895 | 1.741545 | -1.18093 |
| CUST_143132_PI430048170 | 2.26E-03 | -4.60919 | 0.697006 | -1.17969 |
| CUST_109052_PI430048170 | 5.80E-04 | -5.42434 | 2.539236 | -1.17843 |
| CUST_111120_PI430048170 | 1.70E-03 | -4.77738 | 1.080336 | -1.17816 |
| CUST_27845_PI430048170 | 4.64E-02 | -2.83309 | -3.26684 | -1.17777 |
| CUST_124910_PI430048170 | 4.24E-05 | -7.22119 | 6.353713 | -1.17727 |
| CUST_27634_PI430048170 | 6.16E-03 | -4.03244 | -0.62026 | -1.17673 |
| CUST_122998_PI430048170 | 4.19E-02 | -2.89976 | -3.12731 | -1.17629 |
| CUST_58532_PI430048170 | 1.81E-03 | -4.74066 | 0.996727 | -1.17625 |
| CUST_144844_PI430048170 | 1.07E-02 | -3.71501 | -1.34036 | -1.17567 |
| CUST_92460_PI430048170 | 4.04E-02 | -2.92234 | -3.07977 | -1.17539 |
| CUST_74279_PI430048170 | 2.52E-02 | -3.20541 | -2.47249 | -1.17526 |
| CUST_73002_PI430048170 | 8.94E-03 | -3.81953 | -1.10409 | -1.17499 |
| CUST_127028_PI430048170 | 1.35E-02 | -3.57951 | -1.64503 | -1.17449 |
| CUST_141145_PI430048170 | 4.89E-02 | -2.80039 | -3.33479 | -1.17435 |
| CUST_58968_PI430048170 | 1.01E-03 | -5.0844 | 1.776438 | -1.17401 |
| CUST_28618_PI430048170 | 1.21E-02 | -3.64158 | -1.50573 | -1.17378 |
| CUST_126897_PI430048170 | 1.11E-02 | -3.69499 | -1.38552 | -1.17369 |
| CUST_58447_PI430048170 | 3.06E-02 | -3.09246 | -2.71713 | -1.17346 |
| CUST_38022_PI430048170 | 1.38E-02 | -3.56671 | -1.67368 | -1.17327 |
| CUST_135276_PI430048170 | 1.68E-04 | -6.20775 | 4.252661 | -1.17321 |
| CUST_134208_PI430048170 | 2.71E-04 | -5.89414 | 3.575145 | -1.17253 |
| CUST_109489_PI430048170 | 7.57E-04 | -5.25865 | 2.168658 | -1.17234 |
| CUST_17971_PI430048170 | 2.57E-02 | -3.19571 | -2.49359 | -1.17224 |
| CUST_117752_PI430048170 | 8.00E-03 | -3.88166 | -0.96321 | -1.17103 |
| CUST_89814_PI430048170 | 5.36E-03 | -4.10905 | -0.44557 | -1.17037 |
| CUST_121025_PI430048170 | 2.03E-04 | -6.07166 | 3.96012 | -1.17027 |
| CUST_29999_PI430048170 | 4.30E-02 | -2.88235 | -3.16388 | -1.17026 |
| CUST_57861_PI430048170 | 6.34E-03 | -4.0163 | -0.65703 | -1.17019 |
| CUST_127147_PI430048170 | 5.20E-04 | -5.4919 | 2.689611 | -1.17009 |
| CUST_137248_PI430048170 | 9.67E-03 | -3.77344 | -1.2084 | -1.16996 |
| CUST_145587_PI430048170 | 9.67E-03 | -3.77339 | -1.20851 | -1.16953 |
| CUST_77378_PI430048170 | 1.73E-02 | -3.43205 | -1.97391 | -1.16878 |
| CUST_145306_PI430048170 | 6.99E-03 | -3.95852 | -0.78856 | -1.16869 |
| CUST_145354_PI430048170 | 9.63E-03 | -3.77564 | -1.20342 | -1.16861 |
| CUST_71775_PI430048170 | 6.63E-03 | -3.98923 | -0.71867 | -1.16825 |
| CUST_135749_PI430048170 | 3.32E-03 | -4.38578 | 0.18663 | -1.16809 |
| CUST_68011_PI430048170 | 1.76E-04 | -6.17728 | 4.187353 | -1.16727 |
| CUST_116192_PI430048170 | 2.82E-02 | -3.14078 | -2.61281 | -1.16709 |
| CUST_118145_PI430048170 | 2.74E-02 | -3.15746 | -2.57668 | -1.16698 |
| CUST_52814_PI430048170 | 3.54E-02 | -3.00321 | -2.9083 | -1.16669 |
| CUST_73658_PI430048170 | 5.50E-04 | -5.45945 | 2.61744 | -1.16568 |
| CUST_138668_PI430048170 | 1.07E-03 | -5.05006 | 1.698873 | -1.16484 |
| CUST_105785_PI430048170 | 7.30E-03 | -3.93394 | -0.84444 | -1.16397 |
| CUST_73810_PI430048170 | 5.45E-03 | -4.10063 | -0.46479 | -1.16389 |
| CUST_18718_PI430048170 | 3.92E-02 | -2.94119 | -3.03997 | -1.16361 |
| CUST_19446_PI430048170 | 1.67E-02 | -3.45138 | -1.93097 | -1.16346 |
| CUST_33549_PI430048170 | 5.01E-03 | -4.14873 | -0.355 | -1.16337 |
| CUST_114261_PI430048170 | 4.09E-02 | -2.91395 | -3.09745 | -1.16333 |
| CUST_46789_PI430048170 | 6.62E-03 | -3.98987 | -0.71722 | -1.16331 |
| CUST_115605_PI430048170 | 2.78E-03 | -4.48701 | 0.418 | -1.16311 |
| CUST_104059_PI430048170 | 3.28E-05 | -7.42555 | 6.760034 | -1.16281 |
| CUST_142714_PI430048170 | 4.35E-02 | -2.8748 | -3.1797 | -1.1627 |
| CUST_101202_PI430048170 | 3.74E-03 | -4.31845 | 0.032733 | -1.16161 |
| CUST_14933_PI430048170 | 2.01E-02 | -3.34297 | -2.17096 | -1.16132 |
| CUST_53407_PI430048170 | 4.84E-02 | -2.80672 | -3.32165 | -1.16113 |
| CUST_99825_PI430048170 | 3.25E-02 | -3.05504 | -2.79752 | -1.16097 |
| CUST_82041_PI430048170 | 1.20E-04 | -6.44306 | 4.752874 | -1.16095 |
| CUST_112242_PI430048170 | 2.03E-03 | -4.67143 | 0.838975 | -1.16082 |
| CUST_139247_PI430048170 | 1.25E-02 | -3.62333 | -1.54674 | -1.15955 |
| CUST_141186_PI430048170 | 9.07E-03 | -3.8111 | -1.12319 | -1.15862 |
| CUST_98395_PI430048170 | 1.53E-03 | -4.84108 | 1.225211 | -1.15842 |
| CUST_56670_PI430048170 | 3.36E-03 | -4.37767 | 0.168109 | -1.15821 |
| CUST_73190_PI430048170 | 6.89E-04 | -5.31663 | 2.298616 | -1.158 |
| CUST_90453_PI430048170 | 2.28E-02 | -3.267 | -2.33794 | -1.15791 |
| CUST_141602_PI430048170 | 4.25E-05 | -7.21929 | 6.349921 | -1.15714 |
| CUST_132545_PI430048170 | 3.24E-03 | -4.39933 | 0.2176 | -1.15674 |
| CUST_113297_PI430048170 | 4.56E-04 | -5.57537 | 2.874802 | -1.15616 |
| CUST_58043_PI430048170 | 4.15E-02 | -2.90446 | -3.11744 | -1.15557 |
| CUST_118384_PI430048170 | 4.04E-02 | -2.92266 | -3.07908 | -1.15544 |
| CUST_98562_PI430048170 | 6.74E-04 | -5.3306 | 2.329883 | -1.15539 |
| CUST_43465_PI430048170 | 7.44E-04 | -5.26935 | 2.192661 | -1.15517 |
| CUST_107167_PI430048170 | 1.70E-04 | -6.20268 | 4.24179 | -1.15485 |
| CUST_135194_PI430048170 | 8.01E-03 | -3.88107 | -0.96456 | -1.15443 |
| CUST_32207_PI430048170 | 2.18E-02 | -3.29515 | -2.2762 | -1.15441 |
| CUST_111628_PI430048170 | 1.51E-03 | -4.84808 | 1.241099 | -1.15421 |
| CUST_12755_PI430048170 | 5.26E-03 | -4.12137 | -0.41745 | -1.15418 |
| CUST_125249_PI430048170 | 1.80E-03 | -4.74594 | 1.008763 | -1.15407 |
| CUST_20267_PI430048170 | 4.81E-04 | -5.54016 | 2.796767 | -1.15397 |
| CUST_118421_PI430048170 | 4.30E-03 | -4.23829 | -0.15046 | -1.1536 |
| CUST_72511_PI430048170 | 1.30E-02 | -3.59823 | -1.60307 | -1.15291 |
| CUST_110036_PI430048170 | 1.15E-03 | -5.00654 | 1.600463 | -1.15206 |
| CUST_19562_PI430048170 | 2.62E-04 | -5.91659 | 3.624032 | -1.15178 |
| CUST_100181_PI430048170 | 8.16E-04 | -5.2138 | 2.067938 | -1.14865 |
| CUST_90500_PI430048170 | 1.58E-02 | -3.48281 | -1.86104 | -1.14851 |
| CUST_29507_PI430048170 | 1.09E-02 | -3.70469 | -1.36363 | -1.14827 |
| CUST_118619_PI430048170 | 3.55E-02 | -3.00135 | -2.91228 | -1.14748 |
| CUST_4749_PI430048170 | 5.91E-03 | -4.05499 | -0.56885 | -1.14729 |
| CUST_79167_PI430048170 | 2.24E-02 | -3.27722 | -2.31555 | -1.14686 |
| CUST_111657_PI430048170 | 4.96E-05 | -7.09883 | 6.107586 | -1.14678 |
| CUST_75294_PI430048170 | 8.24E-03 | -3.86485 | -1.00135 | -1.14582 |
| CUST_87467_PI430048170 | 1.94E-02 | -3.36253 | -2.12782 | -1.14569 |
| CUST_31375_PI430048170 | 6.97E-04 | -5.30811 | 2.279546 | -1.14531 |
| CUST_81180_PI430048170 | 8.22E-03 | -3.86622 | -0.99826 | -1.1447 |
| CUST_134247_PI430048170 | 7.56E-05 | -6.77648 | 5.449081 | -1.14421 |
| CUST_122624_PI430048170 | 2.47E-02 | -3.21737 | -2.44641 | -1.14385 |
| CUST_41621_PI430048170 | 1.06E-03 | -5.05642 | 1.713256 | -1.14377 |
| CUST_118298_PI430048170 | 4.89E-03 | -4.16424 | -0.31961 | -1.14373 |
| CUST_137802_PI430048170 | 5.10E-03 | -4.13936 | -0.37641 | -1.14369 |
| CUST_78000_PI430048170 | 3.10E-02 | -3.0838 | -2.73576 | -1.14251 |
| CUST_95560_PI430048170 | 1.35E-02 | -3.57617 | -1.6525 | -1.14247 |
| CUST_123546_PI430048170 | 1.30E-02 | -3.5988 | -1.60178 | -1.1423 |
| CUST_19801_PI430048170 | 8.89E-03 | -3.82259 | -1.09717 | -1.14224 |
| CUST_70267_PI430048170 | 1.80E-04 | -6.16299 | 4.156693 | -1.14186 |
| CUST_73946_PI430048170 | 3.03E-03 | -4.4381 | 0.306231 | -1.14174 |
| CUST_170_PI430053867 | 1.91E-02 | -3.37397 | -2.10254 | -1.14169 |
| CUST_77533_PI430048170 | 1.09E-02 | -3.70237 | -1.36886 | -1.1414 |
| CUST_129981_PI430048170 | 1.78E-02 | -3.4156 | -2.0104 | -1.14078 |
| CUST_49230_PI430048170 | 3.84E-02 | -2.95506 | -3.0106 | -1.14048 |
| CUST_129508_PI430048170 | 1.63E-02 | -3.46507 | -1.90053 | -1.14037 |
| CUST_136598_PI430048170 | 2.57E-03 | -4.53443 | 0.526318 | -1.14034 |
| CUST_104508_PI430048170 | 2.76E-04 | -5.8831 | 3.551089 | -1.1401 |
| CUST_98166_PI430048170 | 1.43E-02 | -3.54489 | -1.7225 | -1.13982 |
| CUST_74781_PI430048170 | 4.05E-03 | -4.27311 | -0.07089 | -1.13951 |
| CUST_135492_PI430048170 | 2.68E-03 | -4.5087 | 0.467544 | -1.13947 |
| CUST_138996_PI430048170 | 4.45E-04 | -5.59029 | 2.907828 | -1.13907 |
| CUST_55594_PI430048170 | 1.90E-03 | -4.71131 | 0.92987 | -1.13787 |
| CUST_58789_PI430048170 | 7.04E-03 | -3.9546 | -0.79746 | -1.13787 |
| CUST_82957_PI430048170 | 3.31E-03 | -4.38788 | 0.191437 | -1.13726 |
| CUST_32046_PI430048170 | 1.99E-03 | -4.68562 | 0.871337 | -1.13723 |
| CUST_24452_PI430048170 | 2.43E-02 | -3.23002 | -2.41881 | -1.13713 |
| CUST_136718_PI430048170 | 1.68E-02 | -3.44936 | -1.93547 | -1.1369 |
| CUST_103687_PI430048170 | 2.99E-03 | -4.44538 | 0.322863 | -1.13624 |
| CUST_80181_PI430048170 | 1.73E-03 | -4.76781 | 1.058562 | -1.13615 |
| CUST_74292_PI430048170 | 6.52E-03 | -3.99887 | -0.69672 | -1.13571 |
| CUST_74607_PI430048170 | 4.97E-03 | -4.15435 | -0.34218 | -1.1348 |
| CUST_8457_PI430048170 | 1.99E-02 | -3.3495 | -2.15656 | -1.13439 |
| CUST_142366_PI430048170 | 4.80E-02 | -2.81243 | -3.30982 | -1.1343 |
| CUST_137240_PI430048170 | 4.57E-02 | -2.84235 | -3.24755 | -1.13382 |
| CUST_30246_PI430048170 | 8.09E-03 | -3.87568 | -0.97677 | -1.1338 |
| CUST_118634_PI430048170 | 1.30E-03 | -4.93596 | 1.440577 | -1.1337 |
| CUST_22454_PI430048170 | 2.62E-03 | -4.52068 | 0.494917 | -1.1335 |
| CUST_119180_PI430048170 | 4.52E-02 | -2.85029 | -3.23097 | -1.13299 |
| CUST_12053_PI430048170 | 2.57E-03 | -4.53394 | 0.525199 | -1.13288 |
| CUST_15003_PI430048170 | 8.42E-04 | -5.19474 | 2.025075 | -1.13231 |
| CUST_18765_PI430048170 | 6.10E-03 | -4.0374 | -0.60895 | -1.13205 |
| CUST_111072_PI430048170 | 2.18E-03 | -4.63006 | 0.744628 | -1.13204 |
| CUST_114521_PI430048170 | 1.35E-02 | -3.57868 | -1.64688 | -1.13202 |
| CUST_25173_PI430048170 | 1.74E-03 | -4.76278 | 1.047111 | -1.13139 |
| CUST_137154_PI430048170 | 4.85E-03 | -4.16902 | -0.30868 | -1.13137 |
| CUST_23226_PI430048170 | 5.82E-03 | -4.06422 | -0.54783 | -1.13023 |
| CUST_21675_PI430048170 | 1.05E-03 | -5.05991 | 1.72113 | -1.12994 |
| CUST_131039_PI430048170 | 1.56E-02 | -3.49164 | -1.84137 | -1.12943 |
| CUST_45853_PI430048170 | 3.55E-03 | -4.34875 | 0.101986 | -1.12939 |
| CUST_30086_PI430048170 | 1.11E-02 | -3.69487 | -1.38577 | -1.12887 |
| CUST_130117_PI430048170 | 3.11E-03 | -4.42076 | 0.266587 | -1.12873 |
| CUST_24142_PI430048170 | 1.98E-03 | -4.68618 | 0.872601 | -1.12869 |
| CUST_133488_PI430048170 | 1.74E-02 | -3.42713 | -1.98483 | -1.1284 |
| CUST_131775_PI430048170 | 7.57E-03 | -3.91291 | -0.89224 | -1.12737 |
| CUST_78111_PI430048170 | 1.38E-03 | -4.89894 | 1.356616 | -1.12724 |
| CUST_1979_PI430048170 | 1.22E-02 | -3.63744 | -1.51503 | -1.1269 |
| CUST_128656_PI430048170 | 1.12E-04 | -6.49177 | 4.855534 | -1.12676 |
| CUST_22849_PI430048170 | 4.67E-03 | -4.19028 | -0.26013 | -1.12635 |
| CUST_137315_PI430048170 | 6.21E-03 | -4.02776 | -0.63093 | -1.12612 |
| CUST_37386_PI430048170 | 2.02E-02 | -3.33858 | -2.18066 | -1.12604 |
| CUST_61056_PI430048170 | 1.74E-05 | -7.91163 | 7.702404 | -1.12462 |
| CUST_106033_PI430048170 | 4.48E-04 | -5.58558 | 2.8974 | -1.12452 |
| CUST_83518_PI430048170 | 3.27E-02 | -3.05317 | -2.80153 | -1.12433 |
| CUST_68464_PI430048170 | 1.81E-02 | -3.40562 | -2.03251 | -1.12431 |
| CUST_50113_PI430048170 | 3.40E-03 | -4.37152 | 0.154052 | -1.12429 |
| CUST_93765_PI430048170 | 6.73E-03 | -3.98028 | -0.73905 | -1.1242 |
| CUST_109095_PI430048170 | 1.46E-04 | -6.30034 | 4.450345 | -1.12364 |
| CUST_39854_PI430048170 | 2.45E-02 | -3.22278 | -2.43461 | -1.12332 |
| CUST_61456_PI430048170 | 3.93E-02 | -2.93962 | -3.04327 | -1.12325 |
| CUST_142791_PI430048170 | 3.26E-02 | -3.05475 | -2.79813 | -1.12268 |
| CUST_119204_PI430048170 | 4.33E-04 | -5.60636 | 2.943363 | -1.12237 |
| CUST_135774_PI430048170 | 7.79E-03 | -3.89696 | -0.92847 | -1.12226 |
| CUST_52405_PI430048170 | 1.02E-03 | -5.08035 | 1.7673 | -1.1222 |
| CUST_130512_PI430048170 | 9.68E-04 | -5.1122 | 1.839166 | -1.12208 |
| CUST_137567_PI430048170 | 3.95E-03 | -4.2864 | -0.04051 | -1.12189 |
| CUST_107133_PI430048170 | 2.06E-02 | -3.32697 | -2.20624 | -1.12182 |
| CUST_32562_PI430048170 | 3.70E-04 | -5.70407 | 3.158885 | -1.12149 |
| CUST_130023_PI430048170 | 2.70E-02 | -3.16687 | -2.55628 | -1.12122 |
| CUST_25254_PI430048170 | 1.83E-02 | -3.39878 | -2.04766 | -1.12089 |
| CUST_58337_PI430048170 | 7.96E-03 | -3.88517 | -0.95525 | -1.12075 |
| CUST_117850_PI430048170 | 1.16E-02 | -3.66808 | -1.44611 | -1.12038 |
| CUST_89873_PI430048170 | 4.07E-02 | -2.91769 | -3.08957 | -1.12035 |
| CUST_137015_PI430048170 | 1.69E-03 | -4.78255 | 1.0921 | -1.11994 |
| CUST_70170_PI430048170 | 1.34E-02 | -3.58091 | -1.64187 | -1.1195 |
| CUST_95834_PI430048170 | 3.03E-03 | -4.43781 | 0.305552 | -1.11945 |
| CUST_93709_PI430048170 | 1.76E-03 | -4.75682 | 1.033539 | -1.11881 |
| CUST_36075_PI430048170 | 3.05E-02 | -3.0932 | -2.71552 | -1.11875 |
| CUST_31145_PI430048170 | 3.89E-02 | -2.94661 | -3.0285 | -1.11857 |
| CUST_15883_PI430048170 | 9.19E-04 | -5.1412 | 1.904545 | -1.11823 |
| CUST_16215_PI430048170 | 3.49E-02 | -3.01268 | -2.88811 | -1.11809 |
| CUST_68390_PI430048170 | 7.42E-05 | -6.79013 | 5.477252 | -1.11791 |
| CUST_139834_PI430048170 | 5.25E-04 | -5.48611 | 2.676758 | -1.11779 |
| CUST_141842_PI430048170 | 6.96E-05 | -6.83771 | 5.575285 | -1.11769 |
| CUST_137646_PI430048170 | 1.07E-02 | -3.71543 | -1.33941 | -1.11692 |
| CUST_132221_PI430048170 | 8.98E-05 | -6.65392 | 5.194917 | -1.1167 |
| CUST_59227_PI430048170 | 1.48E-02 | -3.52308 | -1.77124 | -1.11607 |
| CUST_142595_PI430048170 | 3.47E-02 | -3.01543 | -2.88224 | -1.1158 |
| CUST_128120_PI430048170 | 5.00E-03 | -4.15014 | -0.3518 | -1.1154 |
| CUST_94600_PI430048170 | 6.22E-04 | -5.38005 | 2.44043 | -1.11525 |
| CUST_130427_PI430048170 | 3.88E-04 | -5.67417 | 3.093044 | -1.11508 |
| CUST_119670_PI430048170 | 7.76E-05 | -6.75623 | 5.407222 | -1.11493 |
| CUST_10675_PI430048170 | 1.69E-03 | -4.7803 | 1.086967 | -1.1147 |
| CUST_27223_PI430048170 | 1.23E-02 | -3.63292 | -1.52519 | -1.11433 |
| CUST_77024_PI430048170 | 1.07E-03 | -5.05203 | 1.703322 | -1.11407 |
| CUST_33491_PI430048170 | 2.26E-02 | -3.27324 | -2.32426 | -1.11407 |
| CUST_144561_PI430048170 | 4.51E-03 | -4.21038 | -0.21423 | -1.11404 |
| CUST_60968_PI430048170 | 3.75E-02 | -2.96922 | -2.9806 | -1.11397 |
| CUST_55862_PI430048170 | 3.69E-02 | -2.97813 | -2.96167 | -1.1138 |
| CUST_24654_PI430048170 | 5.75E-04 | -5.42999 | 2.551835 | -1.11335 |
| CUST_26571_PI430048170 | 2.32E-02 | -3.25703 | -2.35977 | -1.11307 |
| CUST_111873_PI430048170 | 4.07E-03 | -4.26975 | -0.07856 | -1.11298 |
| CUST_72268_PI430048170 | 2.99E-03 | -4.44534 | 0.322763 | -1.11269 |
| CUST_82076_PI430048170 | 7.20E-03 | -3.94178 | -0.82663 | -1.11267 |
| CUST_52392_PI430048170 | 3.60E-02 | -2.99379 | -2.92837 | -1.11232 |
| CUST_135025_PI430048170 | 1.24E-03 | -4.96231 | 1.500305 | -1.11231 |
| CUST_47897_PI430048170 | 4.04E-04 | -5.64764 | 3.034546 | -1.11221 |
| CUST_61206_PI430048170 | 1.97E-02 | -3.35332 | -2.14814 | -1.11215 |
| CUST_42815_PI430048170 | 2.24E-02 | -3.2781 | -2.31361 | -1.1121 |
| CUST_137_PI430053867 | 6.29E-03 | -4.02111 | -0.64608 | -1.11193 |
| CUST_102009_PI430048170 | 4.93E-02 | -2.79547 | -3.34498 | -1.11153 |
| CUST_74985_PI430048170 | 1.92E-04 | -6.11353 | 4.050379 | -1.11097 |
| CUST_17872_PI430048170 | 6.33E-03 | -4.01727 | -0.65482 | -1.11086 |
| CUST_57460_PI430048170 | 4.32E-03 | -4.23635 | -0.15489 | -1.11043 |
| CUST_101318_PI430048170 | 4.32E-04 | -5.60841 | 2.947885 | -1.11031 |
| CUST_114223_PI430048170 | 5.77E-04 | -5.4282 | 2.547837 | -1.10983 |
| CUST_116123_PI430048170 | 2.83E-03 | -4.47542 | 0.391515 | -1.10948 |
| CUST_97498_PI430048170 | 2.44E-02 | -3.22501 | -2.42975 | -1.1092 |
| CUST_140838_PI430048170 | 7.77E-04 | -5.24454 | 2.136979 | -1.10876 |
| CUST_71465_PI430048170 | 9.24E-03 | -3.80039 | -1.14744 | -1.10871 |
| CUST_139870_PI430048170 | 3.05E-03 | -4.43337 | 0.295421 | -1.10844 |
| CUST_134046_PI430048170 | 3.67E-03 | -4.32848 | 0.055665 | -1.10842 |
| CUST_134833_PI430048170 | 7.15E-05 | -6.81682 | 5.532281 | -1.10804 |
| CUST_68092_PI430048170 | 1.78E-02 | -3.41688 | -2.00756 | -1.10791 |
| CUST_21273_PI430048170 | 4.73E-02 | -2.82134 | -3.2913 | -1.10787 |
| CUST_131111_PI430048170 | 1.31E-03 | -4.92927 | 1.425418 | -1.10757 |
| CUST_144975_PI430048170 | 1.11E-03 | -5.03072 | 1.655162 | -1.10711 |
| CUST_12487_PI430048170 | 2.34E-03 | -4.58823 | 0.649165 | -1.10677 |
| CUST_64892_PI430048170 | 1.20E-02 | -3.64664 | -1.49434 | -1.10663 |
| CUST_41283_PI430048170 | 4.68E-02 | -2.82793 | -3.27759 | -1.10644 |
| CUST_13691_PI430048170 | 1.17E-02 | -3.65956 | -1.46528 | -1.10616 |
[truncated: 28,617 more chars]
